# Progesterone, cerclage, pessary, or acetylsalicylic acid for prevention of preterm birth in singleton and multifetal pregnancies – A systematic review and meta-analyses

**DOI:** 10.3389/fmed.2023.1111315

**Published:** 2023-02-28

**Authors:** Ulla-Britt Wennerholm, Lina Bergman, Pihla Kuusela, Elin Ljungström, Anna C. Möller, Cecilie Hongslo Vala, Ann-Catrin Ekelund, Ann Liljegren, Max Petzold, Petteri Sjögren, Mikael Svensson, Annika Strandell, Bo Jacobsson

**Affiliations:** ^1^Region Västra Götaland, Sahlgrenska University Hospital, Department of Obstetrics and Gynecology, Gothenburg, Sweden; ^2^Department of Obstetrics and Gynecology, Institute of Clinical Sciences, Sahlgrenska Academy, University of Gothenburg, Gothenburg, Sweden; ^3^Department of Obstetrics and Gynecology, Stellenbosch University, Cape Town, South Africa; ^4^Department of Women’s and Children’s Health, Uppsala University, Uppsala, Sweden; ^5^Region Västra Götaland, Södra Älvsborg Hospital, Department of Obstetrics and Gynecology, Borås, Sweden; ^6^Region Västra Götaland, HTA-centrum, Gothenburg, Sweden; ^7^Region Västra Götaland, Skaraborg Hospital, Medical Library, Skövde, Sweden; ^8^Region Västra Götaland, Sahlgrenska University Hospital, Medical Library, Gothenburg, Sweden; ^9^School of Public Health and Community Medicine, Institute of Medicine, University of Gothenburg, Gothenburg, Sweden; ^10^Department of Pharmaceutical Outcomes & Policy, College of Pharmacy, University of Florida, Gainesville, FL, United States; ^11^Department of Genetics and Bioinformatics, Division of Health Data and Digitalization, Institute of Public Health, Oslo, Norway

**Keywords:** preterm birth, perinatal morbidity and mortality, progesterone, cerclage, pessary, acetylsalicylic acid, systematic review

## Abstract

**Background:**

Preterm birth is the leading cause of childhood mortality and morbidity. We aimed to provide a comprehensive systematic review on randomized controlled trials (RCTs) on progesterone, cerclage, pessary, and acetylsalicylic acid (ASA) to prevent preterm birth in asymptomatic women with singleton pregnancies defined as risk of preterm birth and multifetal pregnancies.

**Methods:**

Six databases (including PubMed, Embase, Medline, the Cochrane Library) were searched up to February 2022. RCTs published in English or Scandinavian languages were included through a consensus process. Abstracts and duplicates were excluded. The trials were critically appraised by pairs of reviewers. The Cochrane risk-of-bias tool was used for risk of bias assessment. Predefined outcomes including preterm birth, perinatal/neonatal/maternal mortality and morbidity, were pooled in meta-analyses using RevMan 5.4, stratified for high and low risk of bias trials. The certainty of evidence was assessed using the GRADE approach. The systematic review followed the PRISMA guideline.

**Results:**

The search identified 2,309 articles, of which 87 were included in the assessment: 71 original RCTs and 16 secondary publications with 23,886 women and 32,893 offspring. Conclusions were based solely on trials with low risk of bias (*n* = 50). Singleton pregnancies: Progesterone compared with placebo, reduced the risk of preterm birth <37 weeks 26.7% vs. 30.3% [risk ratio (RR) 0.82 (95% confidence interval [CI] 0.71–0.96)] (high certainty of evidence, 13 trials) thereby reducing neonatal mortality and respiratory distress syndrome. Cerclage probably reduced the risk of preterm birth <37 gestational weeks: 29.0% vs. 37.6% (RR 0.78 [95% CI 0.69 to 0.88]) (moderate certainty of evidence, four open trials). In addition, perinatal mortality may be reduced by cerclage. Pessary did not demonstrate any overall effect. ASA did not affect any outcome, but evidence was based on one underpowered study. Multifetal pregnancies: The effect of progesterone, cerclage, or pessary was minimal, if any. No study supported improved long-term outcome of the children.

**Conclusion:**

Progesterone and probably also cerclage have a protective effect against preterm birth in asymptomatic women with a singleton pregnancy at risk of preterm birth. Further trials of ASA are needed. Prevention of preterm birth requires screening programs to identify women at risk of preterm birth.

**Systematic Review Registration:**

[https://www.crd.york.ac.uk/prospero/], identifier [CRD42021234946].

## Introduction

Preterm birth, defined by the World Health Organization as a birth of a liveborn child before 37 gestational weeks, is the leading global cause of childhood mortality and morbidity. About 15 million children worldwide are born preterm each year, and one million will die due to preterm birth complications. Furthermore, 40% of children born preterm will be subjected to an increased risk of various short- and long-term complications, potentially reducing their quality of life and raising healthcare costs (1). The estimates of preterm birth rate vary worldwide between global regions, countries, and within countries. The preterm birth rate varies between five to 15 percent and is estimated to be even higher in some low- and middle-income countries (2, 3).

Preterm birth is either a spontaneous or a physician-initiated birth. Spontaneous preterm birth accounts for two-thirds of all preterm births and starts with either contractions or preterm prelabor rupture of membranes (PPROM). The underlying causes of spontaneous preterm birth are multifactorial, including infection, inflammation, vascular disease, or distention of the uterus, but the cause often remains elusive (4–6). Another third of preterm births is physician-initiated, often due to maternal or fetal complications such as preeclampsia or intrauterine growth restriction (3, 7). Previous preterm birth or late miscarriage, cervical surgery, multifetal pregnancy, assisted reproductive technology, socioeconomic status, country of birth, ethnicity, smoking, and other lifestyle choices confer increased risk of preterm birth (4, 8–11). In addition, short cervix measured by transvaginal ultrasound in the mid-trimester in women with or without preterm birth risk factors predicts spontaneous preterm birth (12, 13). However, identifying a short cervix in women without risk factors requires a universal or targeted screening program (14).

Both pharmacological and mechanical intervention strategies have been suggested to prevent preterm birth. Progesterone, vaginal cerclage, pessary, and more recently, acetylsalicylic acid (ASA) are examples of commonly studied such interventions.

Progesterone is an essential hormone to maintain a pregnancy until term. It modulates the immune response (15) and is also suggested to reduce the production of prostaglandins, thereby inhibiting myometrial contractions and cervical ripening (16–18). The available administration routes are vaginal, oral, or intramuscular (im) injection.

Cervical cerclage offers mechanical support to the cervix by placing a stitch around the cervix by a vaginal (most common) or transabdominal approach, requiring anesthesia and the resources of an operating theatre (19). Cerclage may also be used as an emergency intervention to close the cervical os if the woman presents with a threatening late miscarriage or preterm birth (rescue cerclage) (20).

There are different techniques for cerclage where the two most commonly used are the McDonald and the Shirodkar technique. Both are applied vaginally. The McDonald cerclage is placed like a purse-string fashion and tied anteriorly. It requires no dissection into paracervical tissue. The Shirodkar cerclage starts with a transverse incision in the vaginal mucosa anterior and posterior to allow upward displacement of the bladder and rectum in order to achieve a higher insertion point of the cerclage. The knot is tied posteriorly, and the mucosa is sutured anteriorly and posteriorly. Complications associated with the procedure are iatrogenic rupture of the membranes, vaginal bleeding, and intraamniotic infection. The transabdominal approach is mainly used after a failed vaginal cerclage or in women who have undergone a trachelectomy (radical surgery after cervical cancer) (21).

The cervical pessary is suggested to change the position of the cervix to a more posterior angle and thus change the weight of the pregnancy towards the anterior lower segment of the uterus. Also, the pessary can prevent the cervix from dilating and prevent the amnion and chorion from dissociation from the uterine wall. It is placed and removed during outpatient visits (22). Thus, the intervention is less invasive and requires less resources than cerclage (23). Side effects are pelvic discomfort and increased vaginal discharge after insertion.

Acetylsalicylic acid has an anti-inflammatory effect, and an impact on preterm birth might be due to decreasing uterine contractility through inhibition of the cyclooxygenase (COX)-dependent prostaglandin synthesis (24). ASA is a conventional treatment to prevent preeclampsia, even though the exact mechanism behind its effect is yet to be uncovered. Even so, low-dose ASA treatment reduces the incidence of preterm births in women at risk of preeclampsia (25). This is likely due to prevention of physician-initiated preterm delivery. It has been suggested that ASA potentially also could reduce spontaneous preterm birth rates in women at risk, but with conflicting results (26).

There have been several contributions to preterm birth prevention in the last few years. The aim of this systematic review was to provide a comprehensive, up-to-date review on randomized trials on progesterone, cerclage, pessary, and ASA for the prevention of preterm birth in asymptomatic women with singleton pregnancies defined as having an increased risk of preterm birth or women with a multifetal pregnancy. The focus on a North European setting is based on the lack of guidelines in several countries in this region, and thus the need for a systematic review explicitly applicable to this population.

## Methods

### Study registration

The protocol for this review was registered at the International Prospective Register of Systematic Reviews (PROSPERO March 26th, 2021, registration ID: CRD42021234946) before data extraction. The Preferred Reporting Items for Systematic Reviews and Meta-analysis (PRISMA) was followed (27).

### Inclusion criteria

The research question was framed as the following:

Will the interventions progesterone, cerclage, pessary, or ASA, alone or in combinations, decrease the risk of preterm birth, neonatal and maternal mortality/morbidity, and long-term child morbidity in asymptomatic women with a singleton pregnancy at risk of preterm birth or in asymptomatic women with a multifetal pregnancy with or without additional risk factor(s)?

Asymptomatic women with previous preterm birth, previous spontaneous late miscarriage between 16 and 22 gestational weeks, short cervix, previous cervical surgical treatment for cervical intraepithelial neoplasia, or with other risk factors as defined by the trial authors were considered to be at risk of preterm birth.

We used the PICO model to define the Populations, Interventions, Comparisons, and Outcomes.

Populations (P) were asymptomatic (without symptoms indicating risk of preterm birth) women with a singleton pregnancy at increased risk of preterm birth <37 + 0 gestational weeks or asymptomatic women with a multifetal pregnancy, irrespective of other risk factors.

Types of interventions (I) were:

- Progesterone, any type initiated in the second trimester, alone or in combination with the other specified interventions in the PICO. Comparisons between different dosage and administration routes were not included.
- Cerclage, any type applied before pregnancy, in the first or second trimester, alone or in combination with the other specified interventions in the PICO.
- Pessary, any type applied in the first or second trimester, alone or in combination with the other specified interventions in the PICO.
- ASA initiated in the first or second trimester, alone or in combination with the other specified interventions in the PICO.

Types of comparisons (C) were no intervention, placebo, or other intervention (progesterone, cerclage, pessary, ASA).

Main outcome (O) measures included preterm birth: Any preterm birth <37 weeks and early preterm birth <34 weeks. We assessed adverse neonatal outcomes associated with preterm birth: perinatal mortality (intrauterine fetal death and neonatal mortality <7 or < 28 days), neonatal mortality <7, <28 days, serious neonatal morbidity (such as bronchopulmonary dysplasia, severe intraventricular hemorrhage, necrotizing enterocolitis, confirmed sepsis, retinopathy of prematurity), individually or as a composite outcome with or without peri/neonatal mortality, and long-term morbidity (such as cerebral palsy, epilepsy, visual impairment, hearing impairment, intellectual impairment, developmental delay). We assessed maternal mortality and maternal morbidity (adverse effects such as infections, surgical complications, cancer).

Other outcomes were any preterm birth <35, <33, <32, <28 gestational weeks, spontaneous preterm birth <37, <35, <34, <33, <32, <28 gestational weeks, gestational length, low birth weight (<2,500 g), and very low birth weight (<1,500 g).

Peri/neonatal and maternal mortality and long-term child outcome were considered to be the most critical outcomes.

### Eligibility criteria

Randomized controlled trials (RCTs) and systematic reviews of RCTs were eligible for inclusion. In addition, in a separate search addressing the intervention of progesterone and the outcome of cancer in the woman, cohort studies with any number of participants were included to support the discussion of long-term risk.

### Exclusion criteria

Exclusion criteria were as follows: Studies not fulfilling PICO, trials not published in English, Swedish, Norwegian, or Danish, abstracts, posters, conference papers, or duplicates.

### Search strategy

Two authors (AL, ACE) performed systematic searches in PubMed, Embase, Medline, and the Cochrane Library (January 25, 2021, with an update on February 1, 2022). In addition, the websites of the Swedish Agency for Health Technology Assessment and Assessment of Social Service (SBU) and The Norwegian Institute of Public Health (Folkehelseinstituttet) were searched, and reference lists of relevant articles were scrutinized for additional references. Search strategies and eligibility criteria are presented in Supplementary material, Appendix 1a.

A separate search was done (November 30, 2021), addressing the intervention progesterone and the long-term risk of cancer in the woman. Search strategies and eligibility criteria are presented in Supplementary material, Appendix 1b.

### Study selection

Two authors (AL, ACE), independently, assessed the obtained abstracts and made the first selection of full-text articles for inclusion. The selected articles were sent to all authors, and at least two other authors independently read the articles. In a consensus meeting, the authors decided which articles to include. Any disagreements were resolved in consensus.

**Figure 1:**
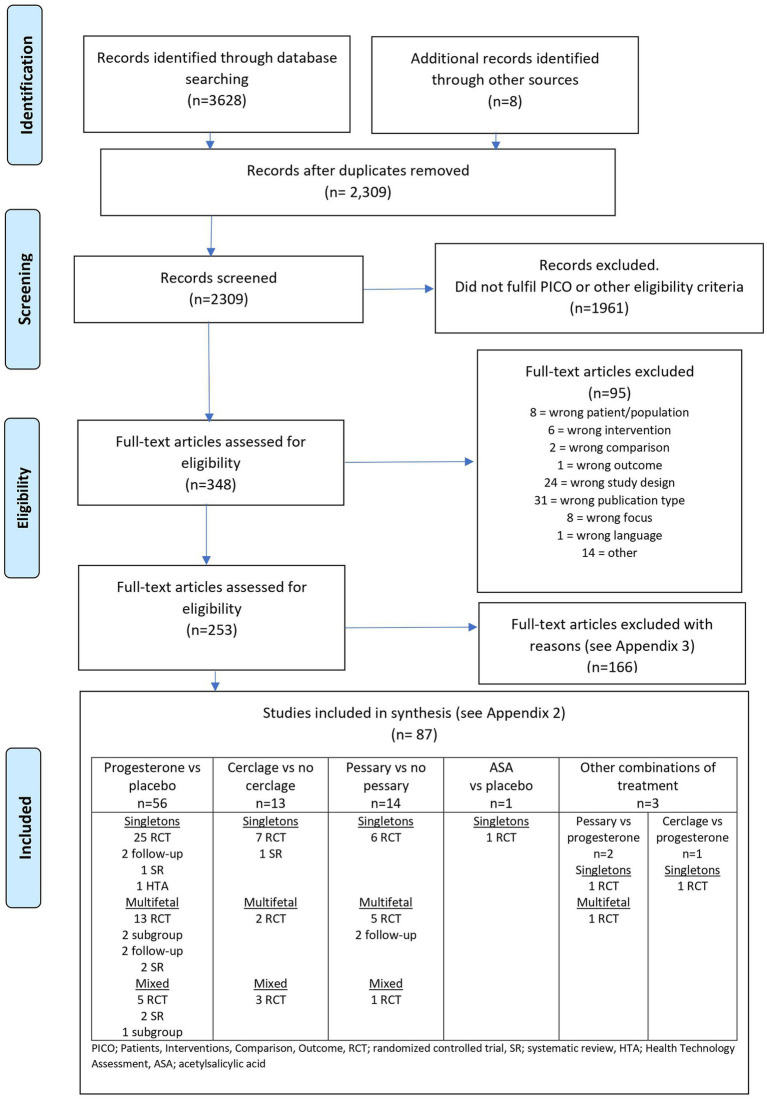
Flow diagram presenting the selection process after the literature search (all interventions).

A graphic presentation of the selection process is presented in Figure 1.

### Quality assessment

At least two authors critically appraised the included trials independently regarding directness (external validity), risk of bias (internal validity), and precision using a checklist for the assessment of RCTs from the Swedish Agency of Health Technology Assessment and the Assessment of Social Services (SBU) and modified by HTA-centrum (28).

A detailed risk of bias assessment of each trial and its primary outcome was conducted using the Cochrane risk-of-bias tool (29). Studies classified as having high risk of bias included those that, in an overall assessment, had major problems with risk of bias (−). In the detailed risk of bias assessment, random sequence generation and allocation concealment had to be evaluated as ‘no or minor problems’ (+) to be classified as low risk of bias. If one of these domains was evaluated as ‘some problems’ (?), at least three of the remaining domains (detection, performance, attrition bias, selective reporting, and other/conflict of interest) had to be classified as ‘no or minor problems’ (+). If both random sequence generation and allocation concealment was evaluated as ‘some problems’ (?), at least four of the remaining domains had to be classified as ‘no or minor problems’ (+). The risk of bias assessment of original trials was applied to all outcomes in subsequent publications.

### Data synthesis and analysis

Data were extracted independently by at least two authors per outcome. Individual data from systematic reviews were only included if not available in the original RCTs. When possible, data were pooled in meta-analyses using a random effects model in RevMan 5.4 and presented as forest plots with an associated risk-of-bias graph. Point estimates were presented as risk ratio (RR) with 95% confidence interval (CI) and as pooled weighted risk differences (RD) with 95% CI. In the case of trials with zero events in one arm, continuity correction by adding 0.5 in each cell was used in the meta-analysis. For three outcomes in there are discrepancies in significances between the presented RR and RD. This difference can occur when there is a borderline significance and is caused by (1) the difference in mathematical approximations when calculating CIs for RR and RD, respectively, and (2) the continuity corrections when calculating RR for trials with zero event-arms which is not done for RD. The latter occurred for two out of the three outcomes. Continuous data that were presented with median and interquartile range (IQR) were transformed to mean and standard deviation (SD), assuming a normal distribution. The heterogeneity of treatment effect was assessed with *I^2^* statistics (30). We assessed publication bias with a funnel plot if more than 10 trials were included in the meta-analysis. Stratified analyses were conducted based on the risk of bias. The two strata are referred to as trials with low and high risk of bias. Conclusions are based solely on trials with low risk of bias. Available data for each outcome of interest are analyzed on an intention-to-treat basis in trials with low risk of bias. Stata (version 17) was used to construct graphs with several pooled estimates.

**Table 2:** Summary of findings for main outcomes (based on trials with low risk of bias): Progesterone (any administration route and dosage unless otherwise stated) vs placebo in women with a singleton pregnancy and any risk factor(s) for preterm birth.

Outcomes	Number of RCTs (patients)	Absolute effect* n/N (%) intervention vs. control	Relative effect risk ratio (95% CI)	Risk difference** (95% CI)	Certainty of evidence GRADE^1^
**Preterm birth (PTB)**					
Any PTB <37 weeks	14 (6303)	940/3511 (26.8) vs. 844/2792 (30.2)	**0.82 (0.71 to 0.95)**	**−6.6 (−10.8 to −2.3)**	⊕⊕⊕⊕
Spontaneous PTB <37 weeks	6 (3698)	389/2198 (17.7) vs. 261/1500 (17.4)	0.87 (0.67 to 1.13)	−2.4 (−7.3 to 2.4)	⊕⊕⊕○ ^2^
Any PTB <35 weeks	5 (3872)	333/2290 (14.5) vs. 298/1582 (18.8)	**0.80 (0.68 to 0.93)**	**−4.1 (−7.8 to −0.5)**	⊕⊕⊕○ ^3^
Spontaneous PTB <35 weeks (17-OHPC)	1 (1687)	93/1113 (8.4) vs. 51/574 (8.9)	0.94 (0.68 to 1.30)	−0.5 (−3.4 to 2.3)	⊕⊕⊕○ ^4^
Any PTB <34 weeks	16 (7581)	487/4152 (11.7) vs. 522/3429 (15.2)	**0.78 (0.68 to 0.89)**	**−3.5 (−5.8 to −1.2)**	⊕⊕⊕⊕
Spontaneous PTB <34 weeks	2 (330)	27/166 (16.3) vs. 47/164 (28.7)	**0.57 (0.38 to 0.86)**	−9.4 (−22.3 to 3.4)***	⊕⊕○○ ^5^
Any PTB <33 weeks (vaginal prog)	5 (974)	70/498 (14.1) vs. 107/476 (22.5)	**0.63 (0.48 to 0.83)**	**−8.4 (−13.0 to −3.8)**	⊕⊕⊕○ ^6^
Any PTB <32 weeks	6 (3645)	153/2173 (7.0) vs. 149/1472 (10.1)	0.71 (0.50 to 1.01)	−4.6 (−9.2 to −0.1)***	⊕⊕○○ ^7^
Spontaneous PTB <32 weeks	2 (1770)	39/1157 (3.4) vs. 25/613 (4.1)	0.84 (0.51 to 1.39)	−0.6 (−2.5 to 1.2)	⊕⊕○○ ^8^
Any PTB <28 weeks	6 (2793)	40/1403 (2.9) vs. 63/1390 (4.5)	**0.64 (0.44 to 0.95)**	−1.4 (−3.1 to 0.2)***	⊕⊕⊕○ ^4^
**Neonatal mortality and morbidity**					
Neonatal mortality <28 days	12 (7169)	53/3953 (1.3) vs. 78/3216 (2.4)	**0.60 (0.39 to 0.92)**	−0.7 (−1.7 to 0.4)***	⊕⊕⊕○ ^4^
Composite adverse	5 (4742)	296/2650 (11.2) vs. 300/2092 (14.3)	0.83 (0.66 to 1.06)	−1.9 (−4.3 to 0.5)	⊕⊕○○ ^9^
Neonatal outcome					
Respiratory distress syndrome	9 (4636)	182/2671 (6.8) vs. 197/1965 (10.0)	**0.70 (0.57 to 0.87)**	**−3.2 (−5.9 to −0.5)**	⊕⊕⊕○^10^
Bronchopulmonary dysplasia	7 (5233)	44/2976 (1.5) vs. 42/2257 (1.9)	0.89 (0.58 to 1.37)	0.1 (−0.4 to 0.7)	⊕○○○ ^11^
Intraventricular	10 (6310)	39/3521 (1.1) vs. 51/2789 (1.8)	0.67 (0.45 to 1.02)	−0.3 (−1.0 to 0.4)	⊕⊕○○ ^11^
Hemorrhage					
Necrotizing enterocolitis	11 (6406)	37/3568 (1.0) vs. 46/2838 (1.6)	0.80 (0.51 to 1.23)	−0.3 (−0.7 to 0.2)	⊕⊕○○ ^12^
Neonatal sepsis	9 (5563)	71/3119 (2.3) vs. 76/2444 (3.1)	0.70 (0.39 to 1.24)	−0.5 (−1.4 to 0.4)	⊕⊕○○ ^13^
Retinopathy of prematurity	5 (3812)	25/2255 (1.1) vs. 24/1557 (1.5)	0.71 (0.34 to 1.45)	−0.4 (−1.2 to 0.3)	⊕⊕○○^14^
Admittance to neonatal intensive care unit	10 (5772)	419/2912 (14.4) vs. 418/2360 (17.7)	0.77 (0.60 to 1.00)	−6.1 (−11.0 to −1.3)	⊕⊕○○ ^15^
**Maternal morbidity**					
Hypertensive disorder in pregnancy	5 (4665)	114/2616 (4.4) vs. 91/2049 (4.0)	0.97 (0.74 to 1.27)	0.1 (−1.1 to 1.2)	⊕⊕⊕○ ^4^
Gestational diabetes mellitus	4 (3519)	80/2112 (3.8) vs. 67/1407 (4.8)	0.83 (0.60 to 1.15)	−0.8 (−2.1 to 0.5)	⊕⊕○○ ^16^
Intrahepatic cholestasis	2 (1369)	4/689 (0.6) vs. 6/680 (0.9)	0.66 (0.19 to 2.33)	−0.3 (−1.2 to 0.7)	⊕⊕○○ ^8^
Chorioamnionitis	6 (4021)	50/2370 (2.1) vs. 33/1651 (2.0)	1.16 (0.75 to 1.80)	0.4 (−0.1 to 0.8)	⊕⊕○○ ^12^
Preterm prelabor rupture of membranes	6 (3648)	197/1836 (10.7) vs. 206/1812 (11.4)	0.93 (0.78 to 1.11)	−0.5 (−1.9 to 0.8)	⊕⊕⊕⊕

Numbers in bold if difference is statistically significant. *Absolute effects for event rates in intervention group vs. control group, presented as the sum of all events/the total numbers of participants, across the RCTs with low risk of bias. **Pooled risk difference (95% CI), presented as the weighted difference in event rates per 100 participants. *** For discrepancies in significances between the presented RR and RD see methods for explanation.

^1Certainty of evidence. High certainty: ⊕⊕⊕⊕, We are very confident that the true effect lies close to that of the estimate of the effect; Moderate certainty: ⊕⊕⊕○, We are moderately confident in the effect estimate: The true effect is likely to be close to the estimate of the effect, but there is a possibility that it is substantially different; Low certainty: ⊕⊕○○, Confidence in the effect estimate is limited: The true effect may be substantially different from the estimate of the effect; Very low certainty: ⊕○○○, We have very little confidence in the effect: The true effect is likely to be substantially different from the estimate of the effect.^

Reasons for downgrading: ^2^ One level based on inconsistency (I^2^ 68%) and uncertain precision. ^3^ One level based on serious indirectness (ethnicity discrepancies). ^4^ One level based on serious imprecision. ^5^ Two levels based on serious indirectness (cervical length with different cut-offs, 10% twins included) and serious imprecision. ^6^ One level due to study limitations (3 of 5 trials include subgroups of original trials). ^7^ Two levels based on some inconsistency (I^2^ 51%), uncertainty in directness (high PTB rate in controls) and serious imprecision (crossing unity). ^8^ Two levels based on very serious imprecision. ^9^ Two levels based on some variation in the definition of the composite outcome, some inconsistency (I^2^ 49%) and serious imprecision (crossing unity). ^10^ One level based on various definitions of the outcome. ^11^ Two levels based on some study limitations, uncertain directness and serious imprecision. ^12^ Two levels based on various definitions of the outcome and serious imprecision. ^13^ Two levels based on various definitions of the outcome, some inconsistency (I^2^ 44%) and serious imprecision. ^14^ Two levels based on some study limitations, inconsistency (I^2^ 27%), indirectness and serious imprecision. ^15^ Two levels based on serious inconsistency (I^2^ 72%), and indirectness (high PTB rate). ^16^ Two levels based on serious imprecision and no definition of gestational diabetes mellitus in three trials.

Singleton and multifetal pregnancies are presented separately. Trials with mixed populations without separate reporting were included in meta-analyses of singleton pregnancies if twins constituted ≤2% of the study population. Trials with >2 and < 10% multifetal pregnancies were also included in meta-analyses of singleton pregnancies, followed by a sensitivity analysis with the exclusion of the mixed population trial. Trials with ≥10% multifetal pregnancies were not included in any meta-analyses of singleton pregnancies.

### Subgroup analyses

Pre-specified subgroup analyses were conducted according to risk factors (short cervix or history of preterm birth) in women with singleton pregnancies among trials with low risk of bias. Subgroup analyses according to the administration route of progesterone (vaginal, oral, or im 17-alpha-hydroxyprogesterone caproate [17-OHPC]) were conducted as exploratory analyses comprising trials with low risk of bias. Subgroup analyses of women with multifetal pregnancies were exploratory according to additional risk factors.

### Assessment of certainty of evidence

The certainty of evidence was defined according to the Grading of Recommendations Assessment, Development, and Evaluation (GRADE) system (31, 32). The GRADE rating results in an assessment of the certainty of evidence in four grades (i) high: we are very confident that the true effect lies close to that of the estimate of the effect, (ii) moderate: we are moderately confident in the effect estimate, and the true effect is likely to be close to the estimate of the effect, but there is a possibility that it is substantially different. (iii) low: confidence in the effect estimate is limited, and the true effect may be substantially different from the estimate of the effect, and (iv) very low: we have very little confidence in the effect estimate, and the true effect is likely to differ substantially from the estimate of effect. The evidence may be downgraded from high certainty by one level for serious (or two or three levels for very serious) limitations, depending on the risk of bias, inconsistency, indirectness of evidence, imprecision of effect estimates, or publication bias.

The domain directness (applicability of evidence) was evaluated generally, i.e., considering proportion of eligible women who were randomized. Directness was also evaluated specifically, i.e., considering applicability to a North European health care setting, including inclusion/exclusion criteria and ethnical background affecting risk for preterm birth. At least three researchers in a joint evaluation determined the certainty of evidence for each outcome.

## Results

### Search results

The literature search in January 2021 and February 2022 identified 2,309 articles after removing duplicates (Supplementary material, Appendix 1a). Two authors excluded 1961 articles after reading the abstracts and another 95 articles after reading the articles in full text. At least two authors assessed the remaining 253 articles, and 87 were included (33–118) in the final assessment (Figure 1). Characteristics of the included trials are presented in Supplementary material, Appendix 2. Excluded trials are listed with reasons for exclusion in Supplementary material, Appendix 3.

A separate search in November 2021, addressing the intervention of progesterone and the outcome of cancer in the woman, identified 369 references (Supplementary material, Appendix 1b). Two authors excluded 365 articles after reading the abstract and the remaining four articles after full text reading. No further references were found.

### Singleton pregnancies

#### Included studies

##### Progesterone

Thirty RCTs reporting on singleton pregnancies were included, and two long-term follow-up reports of RCTs (51, 90) in total 32 publications (Supplementary material, Appendix 2). Seventeen trials were classified as having low risk of bias, and 13 as having high risk of bias (Table 1). Three trials were not placebo-controlled, and as high risk of bias trials, not included in analyses on which conclusions were based. Five trials included both singleton and twin pregnancies, two of these presented results separately for singletons and twins, while three trials did not [percent twins 1.5% (49), 2.7% (71) and 9.6% (58)]. In total, 9,363/9,558 women/newborns were included in these trials.

**Table 1:** Risk of bias assessment (Low *n* = 50/High *n* = 21) of included original randomized controlled trials.

Singleton pregnancies	Risk of bias	Multifetal pregnancies	Risk of bias	Mixed singleton and multifetal pregnancies	Risk of bias
**Articles reporting progesterone vs. placebo**					
Aflatoonian, 2013	High	Awwad, 2015	Low	Aboulghar, 2012	High
Ali, 2020	High	Briery, 2009	High	Cetingoz, 2011	Low
Ashoush, 2017	Low	Brizot, 2015	Low	Crowther, 2017	Low
Azargoon, 2016	High	Caritis, 2009	Low	Fonseca, 2007	Low
Blackwell, 2020	Low	Combs, 2010	Low	Johnson, 1975	High
Da Fonseca, 2003	Low	Combs, 2011	Low		
Glover, 2011	Low	Lim, 2011	Low		
Grobman, 2012	Low	Norman, 2009	Low		
Hassan, 2011	Low	Rehal, 2021	Low		
Hauth, 1983	High	Rode, 2011	Low		
Hayashi, 2021	Low	Rouse, 2007	Low		
Ibrahim, 2010	High	Serra, 2013	Low		
Jabeen, 2012	High	Wood, 2012	Low		
Jafarpour, 2020	High				
Majhi, 2009	Low				
Meis, 2003	Low				
Norman, 2016	Low				
O’Brien, 2007	Low				
Price, 2021	Low				
Rai, 2009	Low				
Saghafi, 2011	High				
Shadab, 2018	High				
Shahgheibi, 2016	High				
Van Os, 2015	Low				
Yemini, 1985	High				
**Articles reporting other interventions vs. progesterone**					
Cruz-Melguizo, 2018	Low	Dang, 2019	Low		
Keeler, 2009a	Low				
**Articles reporting cerclage vs. placebo**					
Althuisius, 2001	High	Dor, 1982	High	Berghella, 2004	High
Ezechi, 2004	High	Roman, 2020	Low	Macnaughton, 1993	Low
Lazar, 1984	High			Rust, 2000	High
Otsuki, 2016	Low				
Owen, 2009	Low				
Rush, 1984	High				
To, 2004	Low				
**Articles reporting pessary vs. placebo**					
Dugoff, 2018	Low	Berghella, 2017a	Low	Pacagnella, 2022	Low
Goya, 2012	Low	Goya, 2016	Low		
Hui, 2013	Low	Liem, 2013a	Low		
Karbasian, 2016	Low	Nicolaides, 2016a	Low		
Nicolaides, 2016b	Low	Norman, 2021	Low		
Saccone, 2017c	Low				
**Articles reporting acetylsalicylic acid (ASA) vs. placebo**					
Landman, 2022	Low				

One secondary publication (63) reported on gestational diabetes mellitus in subgroups from two original trials. In addition, two systematic reviews contributed to the meta-analyses with individual participant data (56, 100), one systematic review (111), and one HTA-report (87) contributed with long-term follow-up data.

##### Cerclage

Ten RCTs reporting on singleton pregnancies were included (Supplementary material, Appendix 2). Four trials were classified as having low risk of bias, and six as having high risk of bias (Table 1). Three trials included both singleton and twin pregnancies, two of these presented results separately for singletons and twins (42, 79) while one study did not (105). In the trial by Macnaughton et al. (79), there were 2% twins, in Berghella et al. (42) 7% twins, and in Rust et al. (105) 11% twins. One trial (57) did not state if only singleton pregnancies were included; however, Ezechi et al. (57) reported individual patient data for singletons for a Cochrane meta-analysis (35).

In addition, one systematic review contributed to the meta-analyses with study data on singleton pregnancies (35). In total, 2,882/2923 women/newborns were included in the analyses.

##### Pessary

Seven RCTs reporting on singleton pregnancies were included (Supplementary material, Appendix 2). All trials were considered to have low risk of bias (Table 1). No systematic review contributed to the meta-analyses. One trial included both singleton (92.4%) and twin (7.6%) pregnancies (94). For this trial, the outcomes of any preterm birth <37, 34, 32, or 28 gestational weeks were reported separately for singletons and twins. For all other outcomes, singleton and twin pregnancies were mixed but reported as singleton outcomes. In total, 2,873/2,951 women/babies were included in the analyses.

##### ASA

One RCT with low risk of bias comprising 387 women/babies was included in the analyses (75) (Supplementary material, Appendix 2).

#### Setting

##### Progesterone

The RCTs were carried out in the USA (7), in the Netherlands (1), and in the Middle East (12), and the rest were conducted in Pakistan, India, Brazil, Japan, and Zambia. Five trials were multinational.

##### Cerclage

The RCTs were carried out in the USA (4), United Kingdom (2), France (2), the Netherlands (3), South Africa (2), Brazil, Slovenia, Greece, Chile, Nigeria, Hungary, Norway, Italy, Japan, Israel, Belgium, Zimbabwe, Iceland, Ireland, and Canada. Two trials were multinational.

##### Pessary

The RCTs were carried out in the USA (1), China (1), Iran (1), Brazil (1), Italy (1), and Spain (1). One trial was multinational.

##### ASA

The trial was conducted in 34 centers in the Netherlands.

#### Population

##### Progesterone

All trials included asymptomatic women with an increased risk of preterm birth, mainly a history of preterm birth (any or spontaneous preterm birth before 37 or 34 gestational weeks), short cervical length (defined as a sonographic cervical length ≤ 30 mm, 25 to <30 mm, ≤25 mm, 10 to ≤20 mm, ≤15 mm), or both. In addition, two trials included women with pregnancies after assisted reproductive technology (ART) as a risk factor. Finally, one trial included women with HIV as a risk factor.

##### Cerclage

The risk of preterm birth was assessed based on previous obstetric history (57, 79, 104) and short cervical length (<25 mm) detected with serial ultrasound examinations (93). Lazar et al. (76) used a mixed scoring system based on obstetric history and physical examination. Althuisius et al. (37) assessed the risk of preterm birth based on previous obstetric history in 2/3 of the study population and short cervical length detected with serial ultrasound examinations of the cervix in 1/3.

Otsuki et al. (92) and To et al. (113) included a largely unselected obstetric population with the need for cerclage assessed with screening for short cervical length in the second trimester with transvaginal ultrasound examination (short cervical length defined as <25 and ≤ 15 mm, respectively, so-called one-off ultrasound). In addition, two trials included a mixed population, with the indication for cerclage based either on short cervical length detected with serial ultrasound examinations of the cervix in women at high risk of preterm birth or a one-off ultrasound examination in women at low risk (42, 105).

##### Pessary

All trials included asymptomatic women with the specific risk factor for preterm birth and short cervical length. The cut-off for inclusion was cervical length ≤ 25 mm in all trials except for one that used ≤30 mm (94).

##### ASA

The trial included women with a history of previous spontaneous preterm birth of a singleton between 22 and 37 gestational weeks.

#### Intervention

##### Progesterone

The interventions included different routes of administration: vaginal progesterone (capsule, gel, or pessary) (13 trials), im injection of progesterone (17-OHPC) (14 trials), or oral progesterone (three trials). Doses of vaginal progesterone varied between 90 and 400 mg per day. Doses of oral progesterone varied between 400 and 600 mg per day. Doses of 17-OHPC were similar across trials (250 mg 17-OHPC im per week), except in one small trial [Hauth et al. (65) which used 1,000 mg/week]. All but three trials used placebo as a control (two used standard care, and one used im injection of vitamin B as a control).

The included trials differed in inclusion criteria regarding gestational age at the onset of intervention (ranging from early to late second trimester) and treatment duration (treatment was stopped between 34 and 37 gestational weeks).

Trials with low risk of bias reported adequate compliance or adherence to treatment (≥80% of prescribed medication) for >90% of participants in nine trials (43, 49, 58, 59, 62, 64, 83, 91, 95). Norman et al. (88), reported adequate compliance for 66% of women in the progesterone group and 71% in the placebo group, and Van Os et al. (115) for 43 and 50%, respectively. Six trials with low risk of bias did not report compliance (38, 52, 66, 80, 96).

##### Cerclage

All trials compared transvaginal cervical cerclage versus no cerclage. Blinding was not feasible due to the nature of the intervention. The intervention included either McDonald cerclage (seven trials) or Shirodkar cerclage (113). One trial used both types of cerclages (92). In one trial, the type of cerclage was not prespecified, but McDonald cerclage or similar was used in most cases (79). Two trials required women in both the cerclage and no cerclage groups to undertake bed rest (37, 42). Modified bed rest in both groups was recommended in two trials (93, 105). The other trials did not routinely recommend any restricted physical activity or did not state whether any recommendations were given. Three trials incorporated a rescue cerclage in the protocol for women randomized to the control group based on physical examination (93) or ultrasound detected changes of the cervix (37, 105). No trial included adjuvant progesterone treatment. The mean gestational age at study entry varied between 14.6 (79) and 24.6 gestational weeks (92). Althuisius et al. (37) included women before 27 gestational weeks and Lazar et al. (76) before 28 gestational weeks. The cerclage suture was removed in six included trials between 36 and 37 gestational weeks without pregnancy complications. Four trials did not report gestational age for removal of the cerclage suture. In the cerclage group, between zero and 13% did not have a cerclage; in the control group, between one and 12%.

##### Pessary

Five trials compared pessary with no pessary. In one of the trials, a vaginal examination was performed in the control group to simulate pessary insertion (67). Blinding was otherwise not considered feasible due to the nature of the intervention. Two trials compared pessary and vaginal progesterone with vaginal progesterone (72, 94). The Arabin pessary was used in five trials, the Biotech cup in one trial (55) and the Ingamed pessary in one trial (94). Women were randomized between 18 and 24 gestational weeks, and the pessary was inserted shortly after randomization. In all trials, the pessary was removed at 36 or 37 gestational weeks. The pessary was removed earlier in case of bleeding, contractions, PPROM, or other complications. In two trials, it was not stated whether the pessary was removed at PPROM (61, 106). If the cervical length was ≤20 mm (55, 106) or ≤ 15 mm (85), vaginal progesterone was recommended as adjuvant therapy.

##### ASA

The intervention was a daily oral intake of 80 mg ASA or a matched placebo, starting between 8 and 16 gestational weeks. Good medication adherence was defined as tablet intake ≥80%. Medication adherence was calculated by dividing the number of used tablets by the expected number of doses per participant. Good adherence was reported by 63.3% of participants. Other interventions for preventing preterm birth, such as progesterone, cerclage, or pessary, could be used alongside the studied intervention if deemed appropriate by the physician.

#### Directness, study limitations, and precision

Quality assessment (directness, study limitations, and precision) of the included trials is presented in the outcome tables in Supplementary material in Appendix 4.1 (progesterone, Supplementary Tables 4.1.1–4.1.26), Appendix 4.2 (cerclage, Supplementary Tables 4.2.1–4.2.22), Appendix 4.3 (pessary, Supplementary Tables 4.3.1–4.3.24), and Appendix 4.4 (ASA, Supplementary Tables 4.4.1–18). The risk of bias in the individual trials is presented in Table 2 (as low or high risk of bias). The detailed risk of bias assessment is presented graphically in color within the forest plots in Supplementary material in Appendix 5.1 (progesterone, Supplementary Figures 1–35), Appendix 5.2 (cerclage, Supplementary Figures 1–23), and Appendix 5.3 (pessary, Supplementary Figures 1–24).

Some of the included trials had some problems with directness, which was affected by ethnicity (i.e., many studies included a high proportion of black women). The study limitation in all trials on cerclage and pessary was the lack of blinding since blinding participants and personnel was considered impossible. The trials were generally underpowered for outcomes such as neonatal mortality and neonatal and maternal morbidity since these were not primary outcomes in most trials.

Publication bias was not detected in any of the funnel plots, and thus not utilized as a reason for downgrading the certainty of evidence.

##### Progesterone

In some studies, directness was affected by a high preterm birth rate in the control group.

More than half of the included trials had no severe study limitations. Three trials were stopped early (62, 83, 115) which affected precision.

##### Cerclage

Some trials were old, conducted, and published before 2000 [low risk of bias: Macnaughton et al. (79); high risk of bias: Lazar et al. (76); Rush et al. (104)], which affected directness. More than half of the included trials had serious study limitations. One trial included miscarriages in numerator and denominator, which affected precision (79).

##### Pessary

Directness was affected by an unusually high incidence of preterm birth in the control group in one study (61), 7.6% of twins in one trial (94), and a screening process that was not clearly described (106). All included trials were categorized as having low risk of bias, despite the lack of blinding.

Two trials were stopped early, affecting precision. The reasons were (1) a competing trial and (2) a lower enrolment than expected (55, 85).

##### ASA

The trial had minor problems with directness since the number of eligible women was not presented. There was no serious study limitation, although there were some baseline differences. A greater proportion of participants in the intervention group had previously undergone cervical or uterine surgery, had a higher rate of previous second-trimester fetal loss and had a positive family history of preterm birth compared with the placebo group.

The precision of the trial was affected by a lower rate of preterm birth than assumed in the sample size calculation. Typically, the certainty of evidence was downgraded two levels due to serious imprecision.

#### Effect of intervention in singleton pregnancies

Complete outcome tables are presented in Supplementary material in Appendix 4.1 (progesterone, Supplementary Tables 4.1.1–4.1.26), 4.2 (cerclage, Supplementary Tables 4.2.1–4.2.22), 4.3 (pessary, Supplementary Tables 4.2.1–4.2.22), and 4.4 (ASA, Supplementary Tables 4.4.1–18). Meta-analyses are presented in Supplementary material in Appendix 5.1 (progesterone, Supplementary Figures 1–35), 5.2 (cerclage, Supplementary Figures 1–23), and 5.3 (pessary, Supplementary Figures 1–24).

##### Progesterone

**Figure 2:**
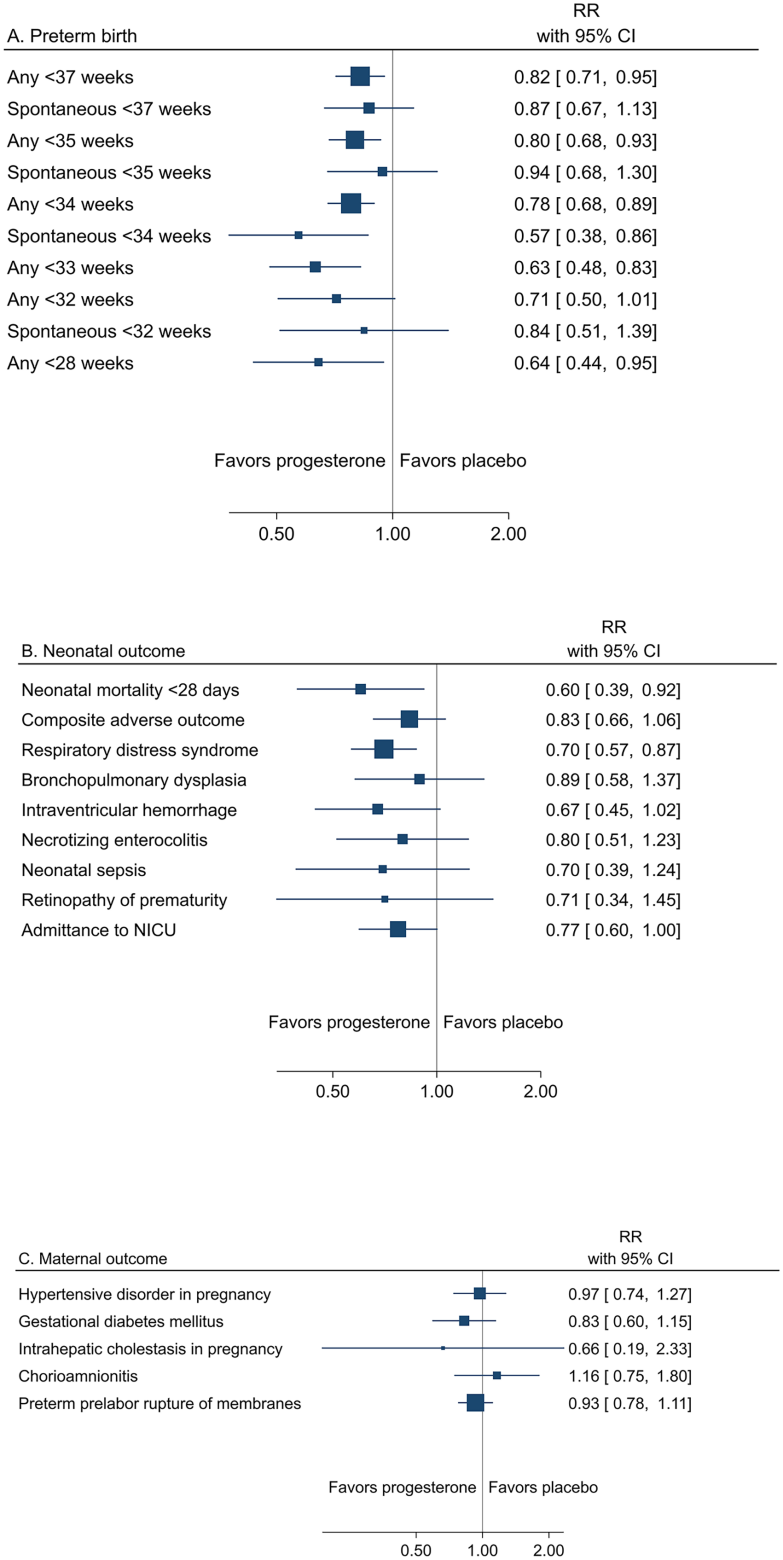
Summary graph of pooled estimates from meta-analyses comparing progesterone and placebo in women with a singleton pregnancy and any type of risk factor for preterm birth, from trials with low risk of bias regarding **(A)** preterm birth, **(B)** neonatal, and **(C)** maternal outcomes.

A summary result per outcome and the associated certainty of the evidence for the main outcomes are presented in Table 2. The pooled estimates from meta-analyses of trials reporting any or spontaneous preterm birth (<37, <35, <34, <33, <32, and < 28 gestational weeks), neonatal, and maternal outcomes from low risk of bias trials are summarized in Figures 2A–C.

###### Preterm birth across gestational weeks

Low risk of bias trials showed an overall effect of progesterone to reduce the risk of preterm birth (Table 2 and Figure 2A). A reduction of any preterm birth was demonstrated for <37 gestational weeks (26.7% vs. 30.3%, RR 0.82; 95% CI 0.71–0.96) and <34 gestational weeks (11.8% vs. 15.4%, RR 0.78; 95% CI 0.67–0.89) for any administration route (high certainty of evidence). A reduction of any preterm birth <35 and < 28 gestational weeks for any administration route and < 33 weeks for vaginal progesterone to women with short cervical length was demonstrated (moderate certainty of evidence).

Spontaneous preterm birth was less frequently reported and thus yielded imprecise estimates for a reduction (low to moderate certainty of evidence) (Table 2).

Prespecified subgroup analysis of trials with women with previous spontaneous preterm birth as a risk factor demonstrated an effect of progesterone on any preterm birth <37 gestational weeks (30.4% vs. 36.6%, RR 0.78; 95% CI 0.65 to 0.94) and < 34 gestational weeks (11.9% vs. 15.7%, RR 0.78; 95% CI 0.62 to 0.98) (Supplementary material, Appendix 5.1, Supplementary Figures 30, 31 and Supplementary Table 2). Exploring the effect of different administration routes, women with short cervical length given vaginal progesterone experienced a reduced risk of preterm birth <34 gestational weeks (Supplementary Figure 35).

###### Neonatal mortality and morbidity

The reduced risk of preterm birth was reflected in neonatal outcomes, in particular as reduced mortality within 28 days (1.3% vs. 2.4%, RR 0.60; 95% CI 0.39 to 0.92) (moderate certainty of evidence) (Table 2 and Figure 2B). All neonatal morbidity outcomes were assessed with risk ratios <1.0 (low certainty of evidence), but only respiratory distress syndrome expressed a significant reduction (6.8% vs. 10.0%; RR 0.70; 95% CI 0.57 to 0.87) (moderate certainty of evidence).

###### Maternal mortality and morbidity

One trial from Zambia in a population of women with HIV reported one maternal death in the placebo group (95). Maternal morbidity outcomes were not significantly affected by progesterone (low to high certainty of evidence) (Table 2 and Figure 2C).

No study has been reported on maternal cancer.

###### Long-term child outcome

Three trials (1,715 children) examined long-term child outcomes in singletons (51) (follow up of Van Os et al. (115) [Triple P]), Norman et al. (88) [OPPTIMUM], Northen et al. (90) [follow up of Meis et al. (83)] (Supplementary Table 4.1.20 in Supplementary material, Appendix 4.1).

A meta-analysis was not feasible due to heterogeneous outcomes. All three trials were included in a systematic review by (111). Follow-up rate was between 71 and 80%. A meta-analysis of two reports (Cuijpers et al. (51) *n* = 57 children [unpublished data), Norman et al. (87) [OPPTIMUM], *n* = 833 children) showed no difference in neurodevelopment assessed by the Bayley-III Cognitive Composite score at 2 years of age between children exposed to progesterone versus placebo (Standardised Mean Difference −0.04, 95% CI −0.26 to 0.19) (111). Northen et al. (90) used the Ages and Stages Questionnaire (ASQ) at 4 to 5 years of age and found no difference between the groups. General health, anthropometry, and behavior were similar between the groups.

##### Cerclage

**Figure 3:**
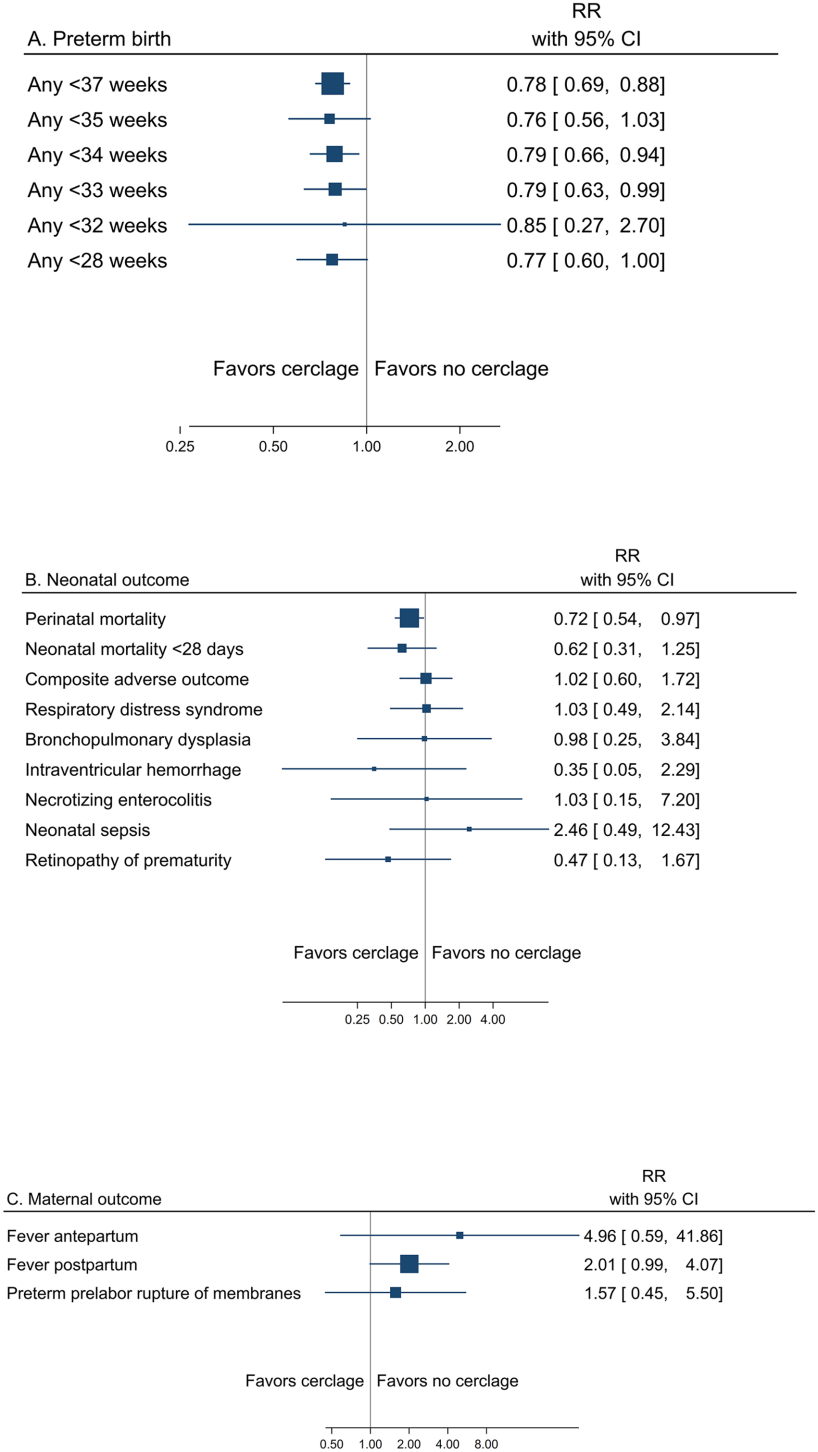
Summary graph of pooled estimates from meta-analyses comparing cerclage versus no cerclage in women with a singleton pregnancy and any type of risk factor for preterm birth, from trials with low risk of bias regarding **(A)** preterm birth, **(B)** neonatal, and **(C)** maternal outcomes.

A summary result per outcome and the associated certainty of the evidence for the main outcomes are presented in Table 3. The pooled estimates from meta-analyses of trials reporting any or spontaneous preterm birth (<37, <35, <34, <33, <32, and < 28 gestational weeks), neonatal, and maternal outcomes from low risk of bias trials are summarized in Figures 3A–C). Prespecified subgroup analysis of trials with women with short cervical length as a risk factor demonstrated an effect of cerclage on preterm birth <37 and < 34 gestational weeks (Supplementary material, Appendix 5.2, Supplementary Figures 22, 23).

**Table 3:** Summary of findings for main outcomes (based on trials with low risk of bias): Cerclage vs no cerclage in women with a singleton pregnancy and any risk factor(s) for preterm birth.

Outcomes	Number of RCTs (patients)	Absolute effect* n/N (%) intervention vs. control	Relative effect risk ratio (95% CI)	Risk difference** (95% CI)	Certainty of evidence GRADE
**Preterm birth (PTB)**					
Any PTB <37 weeks	4 (1919)	286/978 (29.2) vs. 354/941 (37.6)	**0.78 (0.69 to 0.88)**	**−9.9 (−17.2 to −2.7)**	⊕⊕⊕○ ^1^
Any PTB <35 weeks	1 (301)	47/148 (31.8) vs. 64/153 (41.8)	0.76 (0.56 to 1.03)	−10.1 (−20.9 to 0.8)	⊕⊕○○ ^2^
Any PTB <34 weeks	4 (1919)	169/978 (17.3) vs. 210/941 (22.3)	**0.79 (0.66 to 0.94)**	**−4.3 (−7.7 to −0.8)**	⊕⊕⊕○ ^1^
Any PTB <33 weeks	2 (1517)	110/762 (14.4) vs. 138/755 (18.3)	**0.79 (0.63 to 0.99)**	**−3.8 (−7.5 to −0.2)**	⊕⊕○○ ^3^
Any PTB <32 weeks	1 (101)	7/68 (10.3) vs. 4/33 (12.1)	0.85 (0.27 to 2.70)	−1.8 (−15.1 to 11.5)	⊕○○○ ^4^
Any PTB <28 weeks	4 (1915)	89/974 (9.1) vs. 115/941 (12.2)	0.77 (0.60 to 1.00)	−1.8 (−4.3 to 0.6)	⊕⊕⊕○ ^1^
**Peri/neonatal mortality and neonatal morbidity**					
Perinatal mortality	3 (1818)	73/910 (8.0) vs. 101/908 (11.1)	**0.72 (0.54 to 0.97)**	**−2.9 (−5.6 to −0.2)**	⊕⊕○○ ^5^
Neonatal mortality <28 days	3 (1674)	13/854 (1.5) vs. 20/820 (2.4)	0.62 (0.31 to 1.25)	−0.9 (−2.2 to 0.4)	⊕⊕○○ ^6^
Composite adverse neonatal outcome	2 (554)	25/275 (9.1) vs. 25/279 (9.0)	1.02 (0.60 to 1.72)	0.5 (−4.1 to 5.1)	⊕⊕○○ ^7^
Respiratory distress syndrome	1 (300)	13/148 (8.8) vs. 13/152 (8.6)	1.03 (0.49 to 2.14)	0.2 (−6.1 to 6.6)	⊕○○○ ^8^
Bronchopulmonary dysplasia	1 (244)	4/123 (3.3) vs. 4/121 (3.3)	0.98 (0.25 to 3.84)	−0.1 (−4.5 to 4.4)	⊕○○○ ^8^
Intraventricular hemorrhage	2 (544)	1/271 (0.4) vs. 4/273 (1.5)	0.35 (0.05 to 2.29)	−1.1 (−2.9 to 0.6)	⊕○○○ ^8^
Necrotizing enterocolitis	1 (300)	2/148 (1.4) vs. 2/152 (1.3)	1.03 (0.15 to 7.20)	0.04 (−2.6 to 2.6)	⊕○○○ ^8^
Neonatal sepsis	1 (244)	5/123 (4.1) vs. 2/121 (1.7)	2.46 (0.49 to 12.43)	2.4 (−1.8 to 6.6)	⊕○○○ ^8^
Retinopathy of prematurity	2 (544)	3/271 (1.1) vs. 8/273 (2.9)	0.47 (0.13 to 1.67)	−2.0 (−4.3 to 0.4)	⊕○○○ ^8^
**Maternal morbidity**					
Fever antepartum (To, 2004)	1 (253)	5/127 (3.9) vs. 1/126 (0.8)	4.96 (0.59 to 41.86)	NA***	⊕○○○ ^9^
Fever postpartum (Macnaughton, 1993)	1 (798)	23/407 (5.7) vs11/391 (2.8)	2.01 (0.99 to 4.07)	NA***	⊕○○○ ^9^
Preterm prelabor rupture of membranes	2 (1517)	26/762 (3.4) vs. 19/755 (2.5)	1.57 (0.45 to 5.50)	1.1 (−4.6 to 6.9)	⊕⊕○○ ^10^

Numbers in bold if difference is statistically significant. *Absolute effects for event rates in intervention group vs control group, presented as the sum of all events /the total numbers of participants, across the RCTs with low risk of bias. **Pooled risk difference (95% CI), presented as the weighted difference in event rates per 100 participants. ****No pooled estimate was calculated due to the clinical heterogeneity.

Reasons for downgrading: ^1^ One level based on some study limitations (open trials), and some indirectness (one old trial and ethnicity discrepancies). ^2^ Two levels based on some study limitations (open trial), and some indirectness (ethnicity discrepancies), and serious imprecision (few events, crossing unity). ^3^ Two levels based on some study limitations (open trials), some indirectness (one old trial and ethnicity discrepancies), and non-robust result after sensitivity analysis. ^4^ Three levels based on some study limitations (open trial), some indirectness (ethnicity discrepancies) and serious imprecision (very few events). ^5^ Two levels based on some study limitations (open trials), and some indirectness (including miscarriages in Macnaughton, 1993, but no major change after exclusion of that trial) and some imprecision. ^6^ Two levels based on some study limitations (open trials), and some indirectness (including miscarriages and 2% twins in Macnaughton, 1993), and serious imprecision (few events and crossing unity). ^7^ Two levels based on some study limitations (open trials), and some indirectness (ethnicity discrepancies), and serious imprecision (few events, crossing unity). ^8^ Three levels based on some study limitations (open trials), and some indirectness (ethnicity discrepancies), and very serious imprecision (few events, crossing unity). ^9^ Three levels based on some study limitations (open trials, reporting bias), and some indirectness (one old trial and ethnicity discrepancies, including miscarriages in Macnaughton, 1993), and very serious imprecision (few events). ^10^ Two levels based on serious study limitations (open trials and reporting bias regarding the reported number of PPROM), and some indirectness (one old trial and ethnicity discrepancies, including miscarriages and 2% twins in Macnaughton, 1993), and serious imprecision (few events and wide CI).

Low risk of bias trials showed an overall effect of cerclage to reduce the risk of any preterm birth, assessed from <37 down to <28 gestational weeks, reaching significance for <37 gestational weeks (29.2% vs. 37.6%, RR 0.78; 95% CI 0.69 to 0.88) and < 34 gestational weeks (17.3% vs. 22.3%, RR 0.79; 95% CI 0.66 to 0.94) (moderate certainty of evidence) (Table 3 and Figure 3A). The certainty of evidence was low for a reduced risk of preterm birth <35 and < 33 gestational weeks and very low for <32 gestational weeks. Spontaneous preterm birth was not reported.

In pre-specified subgroup analyses of women with short cervical length as a risk factor, cerclage significantly reduced the risk of any preterm birth <37 gestational weeks (36.4% vs. 52.6, RR 0.72; 0.61 to 0.86) and < 34 gestational weeks (22.4% vs. 31.1, RR 0.77; 95% CI 0.60 to 0.99) (Supplementary material, Appendix 5.2, Supplementary Figures 22–23).

The risk of perinatal mortality was significantly reduced with cerclage (8.0% vs. 11.1%, RR 0.72; 95% CI 0.54 to 0.97) (low certainty of evidence) (Table 3 and Figure 3B). Composite adverse neonatal outcome was not significantly affected by cerclage (low certainty of evidence).

Maternal morbidity outcomes were not significantly affected by cerclage (very low to low certainty of evidence) (Table 3 and Figure 3C).

No trial reported on long-term child outcome.

##### Pessary

**Figure 4:**
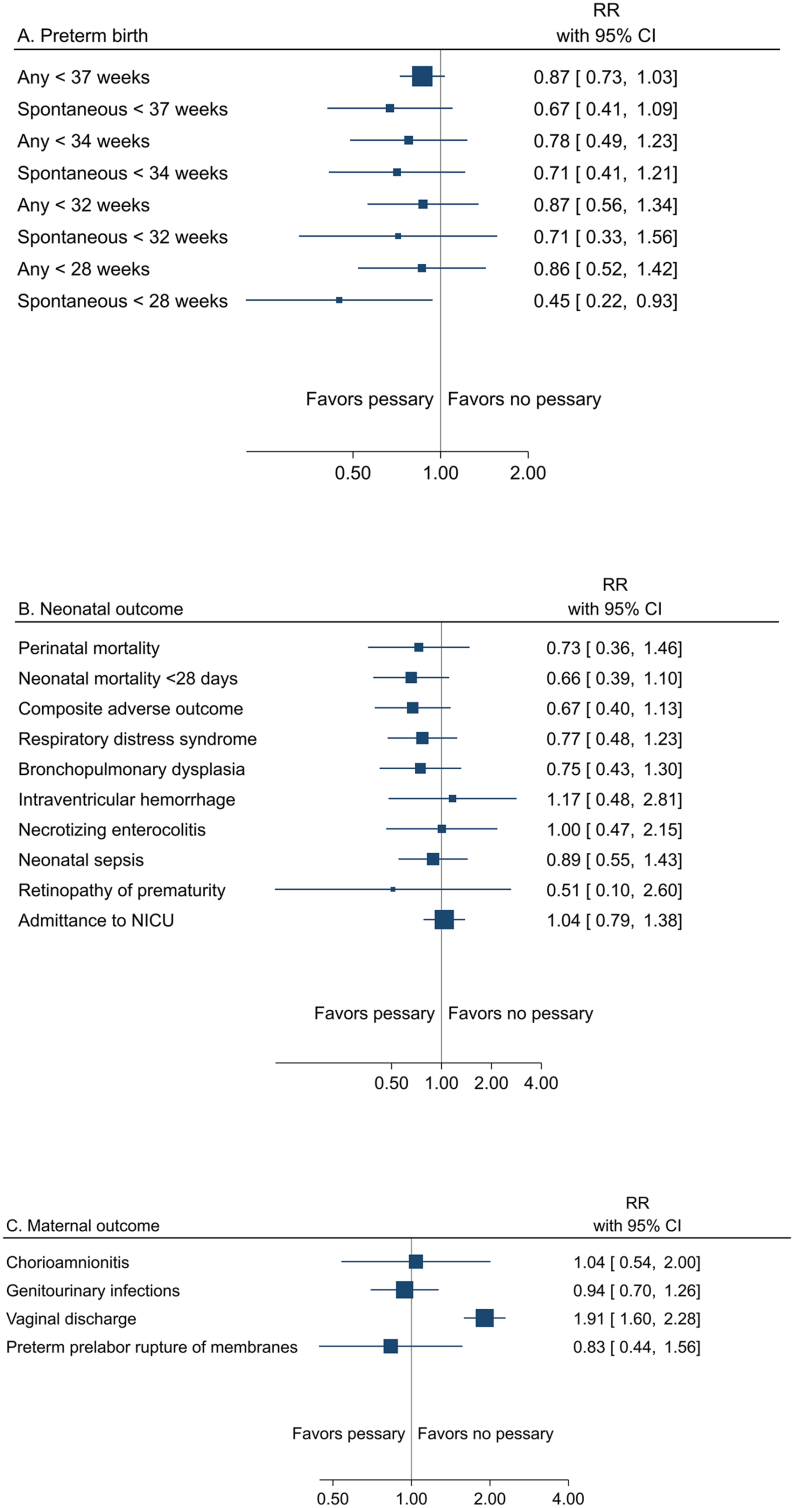
Summary graph of pooled estimates from meta-analyses comparing pessary versus pessary in women with a singleton pregnancy and short cervical length, from trials with low risk of bias regarding **(A)** preterm birth, **(B)** neonatal, and **(C)** maternal outcomes.

A summary result per outcome and the associated certainty of the evidence for the main outcomes are presented in Table 4. The pooled estimates from meta-analyses of trials reporting any or spontaneous preterm birth (<37, <35, <34, <33, <32, and < 28 gestational weeks), neonatal, and maternal outcomes from low risk of bias trials are summarized in Figures 4A–C).

**Table 4:** Summary of findings for main outcomes (based on trials with low risk of bias): Pessary vs. no pessary in women with a singleton pregnancy and short cervical length.

Outcomes	Number of RCTs (patients)	Absolute effect* n/N (%) intervention vs. control	Relative effect risk ratio (95% CI)	Risk difference** (95% CI)	Certainty of evidence GRADE
**Preterm birth (PTB)**					
Any PTB <37 weeks	5 (1531)	187/765 (24.4) vs. 217/766 (28.3)	0.87 (0.73 to 1.03)	−3.7 (−8.5 to 1.0)	⊕⊕⊕○ ^1^
Spontaneous PTB <37 weeks	4 (1694)	168/861 (19.5) vs. 265/833 (31.8)	0.67 (0.41 to 1.09)	−12.6 (−30.6 to 5.4)	⊕⊕○○ ^2^
Any PTB <34 weeks	7 (2843)	156/1420 (11.0) vs. 211/1423 (14.8)	0.78 (0.49 to 1.23)	−3.5 (−9.7 to 2.8)	⊕⊕○○ ^3^
Spontaneous PTB <34 weeks	6 (2726)	131/1374 (9.5) vs. 183/1352 (13.5)	0.71 (0.41 to 1.21)	−4.3 (−10.8 to 2.3)	⊕⊕○○ ^2^
Any PTB <32 weeks	4 (2239)	84/1119 (7.5) vs. 92/1120 (8.2)	0.87 (0.56 to 1.34)	−1.0 (−4.3 to 2.3)	⊕⊕○○ ^4^
Spontaneous PTB <32 weeks	1 (300)	10/150 (6.7) vs. 14/150 (9.3)	0.71 (0.33 to 1.56)	−2.7 (−8.8 to 3.5)	⊕⊕○○ ^5^
Any PTB <28 weeks	5 (2319)	54/1159 (4.7) vs. 59/1160 (5.1)	0.86 (0.52 to 1.42)	−0.6 (−3.1 to 1.9)	⊕⊕○○ ^4^
Spontaneous PTB <28 weeks	4 (1694)	25/861 (2.9) vs. 55/833 (6.6)	**0.45 (0.22 to 0.93)**	**−3.6 (−5.3 to −1.8)**	⊕⊕○○ ^5^
**Peri/neonatal mortality and neonatal morbidity**					
Perinatal mortality	4 (2353)	34/1195 (2.8) vs. 48/1158 (4.1)	0.73 (0.36 to 1.46)	−1.1 (−3.5 to 1.3)	⊕⊕○○ ^4^
Neonatal mortality <28 days	7 (2931)	24/1488 (1.6) vs. 35/1443 (2.4)	0.66 (0.39 to 1.10)	−0.5 (−1.4 to 0.5)	⊕⊕○○ ^5^
Composite adverse neonatal outcome	5 (2668)	167/1353 (12.3) vs. 214/1315 (16.3)	0.67 (0.40 to 1.13)	−5.9 (−13.2 to 1.4)	⊕⊕○○ ^3^
Respiratory distress syndrome	6 (2761)	153/1402 (10.9) vs. 176/1359 (13.0)	0.77 (0.48 to 1.23)	−2.7 (−7.7 to 2.2)	⊕⊕○○ ^4^
Bronchopulmonary dysplasia	3 (1365)	21/707 (3.0) vs. 27/658 (4.1)	0.75 (0.43 to 1.30)	−0.8 (−2.4 to 0.9)	⊕⊕○○ ^5^
Intraventricular hemorrhage	5 (1812)	17/903 (1.9) vs. 14/909 (1.5)	1.17 (0.48 to 2.81)	−0.0 (−1.6 to 1.5)	⊕⊕○○ ^4^
Necrotizing enterocolitis	5 (2651)	14/1347 (1.0 vs. 14/1304 (1.1)	1.00 (0.47 to 2.15)	−0.1 (−0.8 to 0.6)	⊕⊕○○ ^6^
Neonatal sepsis	6 (2759)	58/1400 (4.1) vs. 62/1359 (4.6)	0.89 (0.55 to 1.43)	−0.7 (−2.9 to 1.5)	⊕⊕○○ ^4^
Retinopathy of prematurity	4 (1704)	8/850 (0.9) vs. 16/854 (1.9)	0.51 (0.10 to 2.60)	−1.6 (−4.5 to 1.4)	⊕⊕○○ ^4^
Admittance to neonatal intensive care unit	5 (2428)	185/1230 (15.0) vs. 170/1198 (14.2)	1.04 (0.78 to 1.38)	0.4 (−3.4 to 4.2)	⊕⊕○○ ^4^
**Maternal morbidity**					
Chorioamnionitis	4 (942)	18/471 (3.8) vs. 17/471 (3.6)	1.04 (0.54 to 2.00)	0.1 (−2.0 to 2.3)	⊕⊕○○ ^6^
Genitourinary infection	3 (1165)	73/588 (12.4) vs. 77/577 (13.3)	0.94 (0.70 to 1.26)	−1.1 (−4.8 to 2.6)	⊕⊕○○ ^5^
Vaginal discharge	3 (798)	364/400 (91.0) vs. 184/398 (46.2)	**1.91 (1.60 to 2.28)**	**41.5 (26.3 to 56.8)**	⊕⊕⊕○ ^7^
Preterm prelabor rupture of membranes	5 (1838)	56/927 (6.0) vs. 61/911 (6.7)	0.83 (0.44 to 1.56)	−1.4 (−5.1 to 2.3)	⊕⊕○○ ^4^

Numbers in bold if difference is statistically significant. * Absolute effects for event rates in intervention group vs. control group, presented as the sum of all events /the total numbers of participants, across the RCTs with low risk of bias. ** Pooled risk difference (95% CI), presented as the weighted difference in event rates per 100 participants.

Reasons for downgrading: ^1^ One level based on lack of blinding, uncertain directness regarding populations, uncertain precision. ^2^ Two levels based on lack of blinding, uncertain directness regarding populations, uncertain precision, serious inconsistency. ^3^ Two levels based on serious inconsistency and imprecision. ^4^ Two levels based on lack of blinding, some inconsistency, uncertain directness regarding populations, serious imprecision. ^5^ Two levels based on lack of blinding, uncertain directness regarding populations, serious imprecision. ^6^ Two levels based on very serious imprecision. ^7^ One level based on lack of blinding and subjective outcome.

All women had short cervical length as a risk factor for preterm birth. Low risk of bias trials demonstrated no effect of pessary on the risk of any preterm birth <37 weeks (24.2% vs. 28.3%, RR 0.87; 95% CI 0.73 to 1.03) (moderate certainty of evidence), <34 gestational weeks (11.0% vs. 14.8%; RR 0.78; 95% CI 0.49 to 1.23) (low certainty of evidence), or for other assessed gestational weeks, apart from spontaneous preterm birth <28 weeks, where a reduced risk with pessary was shown (2.9% vs. 6.6%, RR 0.45; 95% CI 0.22 to 0.93) (low certainty of evidence) (Table 4; Figure 4A).

Peri/neonatal mortality and neonatal morbidity was not affected by pessary (perinatal mortality 2.8% vs. 4.1%, RR 0.73; 95% CI 0.36 to 1.46) (low certainty of evidence for all neonatal outcomes) (Table 4 and Figure 4B).

Maternal morbidity was not affected by pessary (low certainty of evidence), except for an increase in vaginal discharge (moderate certainty of evidence) (Table 4 and Figure 4C).

No trial has been reported on long term child outcomes.

##### ASA

Results per outcome and the associated certainty of the evidence for the main outcomes are presented in Table 5. No difference between groups was demonstrated below any gestational week, reported from <37 to <28 weeks (low certainty of evidence), (any preterm birth <37 gestational weeks: 21.2% vs. 25.4%, RR 0.83; 95% CI 0.58 to 1.20, any preterm birth <34 gestational weeks 9.3% vs. 8.8%, RR 1.05; 95% CI 0.56 to 1.98).

**Table 5:** Summary of findings for main outcomes (based on trials with low risk of bias): Acetylsalicylic acid (ASA) vs placebo in women with a singleton pregnancy and the risk factor previous spontaneous preterm birth.

Outcomes	Number of RCTs (patients)	Absolute effect*n/N (%) intervention vs. control	Relative effect risk ratio (95% CI)	Risk difference** (95% CI)	Certainty of evidence GRADE
**Preterm birth (PTB)**					
Any PTB <37 weeks	1 (387)	41/194 (21.2) vs. 49/193 (25.4)	0.83 (0.58 to 1.20)	−4.24 (−12.7 to 4.2)	⊕⊕○○ ^1^
Spontaneous PTB <37 weeks	1 (387)	39/194 (20.1) vs. 46/193 (23.8)	0.84 (0.58 to 1.23)	−3.7 (−12.0 to 4.5)	⊕⊕○○ ^1^
Any PTB <34 weeks	1 (387)	18/194 (9.3) vs. 17/193 (8.8)	1.05 (0.56 to 1.98)	0.5 (−5.2 to 6.2)	⊕⊕○○ ^1^
Spontaneous PTB <34 weeks	1 (387)	18/194 (9.3) vs. 16/193 (8.3)	1.12 (0.59 to 2.13)	1.0 (−4.6 to 6.6)	⊕⊕○○ ^1^
Any PTB <28 weeks	1 (387)	7/194 (3.6) vs. 5/193 (2.6)	1.39 (0.45 to 4.31)	1.0 (−2.4 to 4.5)	⊕⊕○○ ^1^
Spontaneous PTB <28 weeks	1 (387)	7/194 (3.6) vs. 5/193 (2.6)	1.39 (0.45 to 4.31)	1.0 (−2.4 to 4.5)	⊕⊕○○ ^1^
**Perinatal mortality and neonatal morbidity**					
Perinatal mortality	1 (387)	6/194 (3.1) vs. 2/193 (1.0)	2.99 (0.61 to 14.60)	2.1 (−0.8 to 4.9)	⊕○○○ ^2^
Composite adverse neonatal outcome	1 (387)	9/194 (4.6) vs. 5/193 (2.6)	1.79 (0.61 to 5.25)	2.0 (−1.7 to 5.8)	⊕○○○ ^2^
Bronchopulmonary dysplasia	1 (387)	1/194 (0.5) vs. 3/193 (1.6)	0.33 (0.04 to 3.16)	−1.0 (−3.0 to 1.0)	⊕○○○ ^3^
Intraventricular hemorrhage	1 (387)	1/194 (0.5) vs. 0/193 (0)	0.22 (0.01 to 5.05)	NA	⊕○○○ ^3^
Necrotizing enterocolitis	1 (387)	1/914 (0.5) vs. 0/193 (0)	0.22 (0.01 to 5.05)	NA	⊕○○○ ^3^
Neonatal sepsis	1 (387)	4/194 (2.1) vs. 2/193 (1.0)	1.99 (0.37 to 10.74)	1.0 (−1.4 to 3.5)	⊕○○○ ^3^
Retinopathy of prematurity	1 (387)	1/194 (0.5) vs. 2/193 (1.0)	0.50 (0.05 to 5.44)	0.5 (−2.3 to 1.2)	⊕○○○ ^3^
Admittance to neonatal intensive care unit	1 (387)	13/194 (6.7) vs. 11/193 (5.7)	1.18 (0.54 to 2.56)	1.0 (−3.8 to 5.8)	⊕⊕○○ ^1^
**Maternal morbidity**					
Gestational hypertension	1 (387)	4/194 (2.1) vs. 5/193 (2.6)	0.80 (0.22 to 2.92)	−0.5 (−3.5 to 2.5)	⊕○○○ ^3^
Gestational diabetes mellitus	1 (387)	15/194 (7.7) vs. 15/193 (7.8)	1.00 (0.50 to 1.98)	−0.0 (−5.4 to 5.3)	⊕⊕○○ ^1^
Any genital infection	1 (387)	6/194 (3.1) vs. 15/193 (7.8)	0.40 (0.16 to 1.00)	−4.7 (−9.2 to −0.2)	⊕○○○ ^4^
Vaginal bleeding	1 (222)	5/106 (4.7) vs. 7/116 (6.0)	0.78 (0.26 to 2.39)	−1.3 (−7.2 to 4.6)	⊕○○○ ^5^
Other bleeding		17/106 (16.0) vs. 12/115 (10.4)	1.54 (0.77 to 3.01)	5.7 (−3.2 to 14.6)	
Preterm prelabor rupture of membranes	1 (387)	9/194 (4.6) vs. 18/193 (9.3)	0.50 (0.23 to 1.08)	−4.7 (−9.8 to 0.4)	⊕⊕○○ ^1^

*Absolute effects for event rates in intervention group vs. control group, presented as the sum of all events /the total numbers of participants, across the RCTs with low risk of bias. ** Pooled risk difference (95% CI), presented as the weighted difference in event rates per 100 participants.

Reasons for downgrading: ^1^Two levels based on very serious imprecision. ^2^ Three levels based on very serious imprecision and uncertainty about directness (the outcome included fetal deaths (≥16 weeks). ^3^ Three levels based on extremely serious imprecision. ^4^ Three levels based on very serious imprecision and study limitation (cointervention with cerclage in the ASA group). ^5^ Three levels based on very serious imprecision and study limitation (self-reported/subjective outcome).

Any assessment of neonatal mortality and morbidity was uncertain (very low certainty of evidence), except for no difference in admittance to the neonatal intensive care unit (NICU) (low certainty of evidence).

The maternal morbidity and mortality assessments were uncertain (very low certainty of evidence), except for no differences in gestational diabetes mellitus and PPROM (low certainty of evidence).

Long-term child outcome was not reported.

### Comparison of other interventions vs. progesterone in singleton pregnancies

Two trials with low risk of bias, including singleton pregnancies, were identified (Table 2). One trial, including 79 women with different risk factors for preterm birth and short cervical length (<25 mm), compared McDonald cerclage versus im 17-OHPC (250 mg weekly) (73). The other trial, including 243 women with a singleton pregnancy and short cervical length (<25 mm), compared pessary versus vaginal progesterone (200 mg daily) (50). The trials were performed in the USA and Spain, respectively. Outcome tables are presented in Appendix 4.1.1–4.1.26 after the progesterone vs. placebo trials.

#### Effect of cerclage vs. 17-OHPC in singleton pregnancies

Women with different risk factors for preterm birth and a cervical length ≤ 25 mm had a similar risk of spontaneous preterm birth <35 weeks (primary outcome) when treated with cerclage or 17-OHPC, RR 1.14 (95% CI 0.67 to 1.93) (73). The certainty of evidence was downgraded three levels due to non-blinding, discrepancies in ethnicity, and very serious imprecision (very low certainty of evidence for no difference in spontaneous preterm birth <35 weeks). There were no differences in other preterm birth rates, neonatal mortality, or morbidity.

#### Effect of pessary vs. vaginal progesterone in singleton pregnancies

The Cruz-Melguizo et al. (50) trial did not show non-inferiority (exceeded margin of 4%) for pessary versus vaginal progesterone for the primary outcome of spontaneous preterm birth <34 weeks. The event rate was 14.0% in both groups, and the RD was −0.11 percentage points (95% CI −8.85 to 8.62%). The certainty of evidence was downgraded by two levels due to non-blinding and serious imprecision (low certainty of evidence for no difference in spontaneous preterm birth <34 weeks). No significant differences were found for secondary neonatal or maternal outcomes.

### Multifetal pregnancies

#### Included studies

##### Progesterone

Eighteen RCTs, three subgroup analyses of RCTs (63, 74, 82) and two long-term follow-up reports of RCTs (81, 116) were included, in total 23 publications (Supplementary material, Appendix 2). Fifteen RCTs were classified as having low risk of bias, and three as having high risk of bias (Table 2). Three trials (33, 71) included both singleton and twin pregnancies and presented results separately for singletons and twins. Nine RCTs included only twin pregnancies (39, 44, 45, 48, 86, 97, 98, 103, 108) as did the two follow-up reports (81, 116). Two trials included only triplet pregnancies (46, 47). One trial (117) included twin and triplet pregnancies, and one trial included twin, triplet, and quadruplet pregnancies (78).

In addition, four systematic reviews (Romero et al. (101) with an update Romero et al. (102), EPPPIC (56), Simons et al. (111)) contributed individual participant data for some outcomes, including data for twins from two trials with mixed singletons and twins [Fonseca et al. (58) low risk of bias, Crowther et al. (49) low risk of bias]. In total, 5,370/10,827 women/newborns were included in the meta-analyses.

##### Cerclage

Two RCTs with only twin pregnancies were included (Supplementary material, Appendix 2). One was classified as having low risk of bias, and one as having high risk of bias (Table 2). In addition, two RCTs, including singleton and twin pregnancies, contributed data on twin pregnancies (42, 79). Above, in the singleton section, the setting, population, and intervention are presented for these two trials. No trial included triplet or higher order multiple births. In total, 107/214 women/newborns were included in the meta-analyses.

##### Pessary

Seven publications of five RCTs were included, with two long-term follow-up reports (112, 114) (Supplementary material, Appendix 2). All trials were considered to have a low risk of bias (Table 2). No systematic reviews contributed to the meta-analysis. One trial included both twins (98%) and triplets (2%) (77). Another trial, including both singleton and twin pregnancies (7.6%), contributed with data on twin pregnancies (94). In total, 2,913/6042 number of women/newborns were included in the meta-analyses.

#### Setting

##### Progesterone

Six trials were single center studies carried out in Egypt, Lebanon, the USA, Brazil, and Turkey (2). Eight were national multicenter studies carried out in the USA (four trials), the Netherlands, the United Kingdom, Spain, and Canada, and four were multinational studies (49, 58, 97, 98) carried out in the United Kingdom, Chile, Brazil, Greece, Spain, Bulgaria, Italy, Belgium, France, Austria, Denmark. Australia, New Zeeland, and Canada.

##### Cerclage

The Dor trial (54) was a single-center study conducted in Israel. The Roman trial (99) was a multicenter study conducted at eight centers in the USA.

##### Pessary

The included trials were conducted in the USA, Spain, the Netherlands, and the United Kingdom, including one British study with cohorts from 12 countries.

#### Population

##### Progesterone

Three trials (33, 48, 108) included only dichorionic twin pregnancies. One of the triplet trials included only trichorionic triplet pregnancies (47), and the other included both dichorionic and trichorionic triplet pregnancies (46). Six twin studies (39, 45, 86, 97, 98, 103) included both monochorionic and dichorionic twin pregnancies (15–23% monochorionic pregnancies). Three trials excluded all women with prior preterm birth or spontaneous preterm birth (47, 48, 78). One trial with both singleton and twin pregnancies (58) exclusively included those with short cervical length (defined as a cervical length ≤ 15 mm). Three trials (45, 74, 78) presented results for subgroups of women with short cervix (<25 mm, ≤25 mm, and ≤ 30 mm, respectively). Two trials presented results for subgroups of women with a previous preterm birth (74, 82). One trial included only ART pregnancies (33), two trials excluded ART pregnancies (44, 45), and in 10 trials, ART pregnancies comprised between 25 and 95% of the study population. Five trials had no information on the use of ART.

##### Cerclage

The Dor trial (54) included asymptomatic women at 13 gestational weeks with twin pregnancies after ovulation induction. The Roman trial (99) had a high-risk group of women between 16 and 23 gestational weeks with diamniotic twin pregnancies, asymptomatic cervical dilation of 1 to 5 cm, and visible membranes identified by transvaginal ultrasound examination or physical examination.

##### Pessary

All trials included asymptomatic women with a multifetal pregnancy with or without additional risk factors. Three articles included only women with short cervical lengths ≤25 mm (60), ≤30 mm (41), and ≤ 35 mm (89). One trial had a subgroup of women with cervical length ≤ 38 mm (77).

#### Intervention

##### Progesterone

The interventions included different routes of administration: vaginal progesterone (capsule, gel, or pessary) (nine trials) and im injection of 17-OHPC (seven trials). Doses of vaginal progesterone varied between 90 and 600 mg per day. Doses of 17-OHPC were similar across trials (250 mg 17-OHPC im per week). All trials used placebo as a control and were double-blinded. The intervention started in the first trimester between 11 and 14 gestational weeks in one trial (97) and in the second trimester between 16 and 24 gestational weeks in the other trials. Treatment was stopped between 34 and 36 gestational weeks.

Trials with low risk of bias reported adequate compliance or adherence to treatment (≥80% of the prescribed medication) for >90% of patients participating in 10 trials. Three trials with low risk of bias reported compliance between 77 and 85% (97, 98, 108). Norman et al. (86), (low risk of bias) reported adequate compliance for 54% of women in the progesterone group and 60% in the placebo group. Compliance was not reported in one trial [Cetingoz et al. low risk of bias].

##### Cerclage

The trials compared McDonald cerclage versus no cerclage (54, 99). Blinding was not feasible due to the nature of the intervention. In the Dor et al. trial (54), women were followed at a high-risk pregnancy clinic. In the Roman et al. trial (99), women in both arms were observed at the hospital until they were stable for discharge. After discharge, there were no study-specific recommendations for pregnancy care. In the Roman et al. trial (99), 4/18 (22%) in the cerclage group did not receive any cerclage, and none (0/16) in the control group received any cerclage. The cerclage was removed at 36 gestational weeks in one trial (99) and 37 gestational weeks in the other trial (54) or earlier in case of labor, PPROM, or other pregnancy complications requiring delivery.

No trial included adjuvant progesterone treatment in both groups as a routine. Roman et al. (99) reported use of vaginal progesterone in 52.9% in the cerclage group and 76.9% in the no cerclage group.

##### Pessary

All seven publications (five trials) compared pessary with no pessary. Blinding was not feasible due to the nature of the intervention. The Arabin pessary was used in five trials and the Biotech cup in one trial (41). Women were randomized between the 16 + 0 and 27 + 6 gestational weeks, and the pessary was inserted shortly after randomization. In all trials, the pessary was removed at 36 or 37 gestational weeks. In the case of bleeding, contractions, PPROM, or other complications, the pessary was removed earlier. In Goya et al. (60) women were controlled once a month with ultrasound and a clinical questionnaire to confirm the correct placement of the pessary and cervical length. The pessary was not removed at PPROM, but the women were followed up at the hospital, and the pessary was removed in case of chorioamnionitis or onset of labor.

#### Directness, study limitations, and precision

Quality assessment (directness, study limitations, and precision) of the included trials is presented in the outcome tables in Supplementary material, Appendix 4.1 (progesterone, Supplementary Tables 4.1.1–4.1.26), Appendix 4.2 (cerclage, Supplementary Tables 4.2.1–4.2.22), and in Appendix 4.3 (pessary, Supplementary Tables 4.3.1–4.3.24). The risk of bias in the individual trials is presented in Table 2 (as low or high risk of bias). The detailed risk of bias assessment is presented graphically in color within the forest plots in Supplementary material, Appendix 6.1 (progesterone, Supplementary Figures 1–33, Appendix 6.2 (cerclage, Supplementary Figures 1–21), and in Appendix 6.3 (pessary, Supplementary Figures 1–26). Although not feasible, the cerclage and pessary trials were limited by lack of blinding. The progesterone and pessary trials were generally underpowered for outcomes such as neonatal mortality, neonatal and maternal morbidity, and the cerclage trials for all outcomes.

Publication bias was not detected in any of the funnel plots, and thus not utilized as a reason for downgrading the certainty of evidence.

##### Progesterone

Some of the trials had problems with directness, which was affected by ethnicity and the use of ART (i.e., many studies included a high proportion of black women and/or a high proportion of ART pregnancies). Three of the 18 trials were conducted in European countries with an ethnicity similar to the Swedish population (78, 98, 108). Most trials had no serious study limitations.

##### Cerclage

One old trial included only twin pregnancies after infertility treatment (54).

##### Pessary

The five trials and the two reports showed no major directness problem. However, it is also noted that the two reports on long-term outcomes had a significant loss to follow-up, which may have affected the results. The follow-up rate was 45% (112) and 83% (114). However, all original trials were considered to have low risk of bias.

### Effect of intervention in multifetal pregnancies

Complete outcome tables are presented in Supplementary material in Appendix 4.1 (progesterone, Supplementary Tables 4.1.1–4.1.26), 4.2 (cerclage, Supplementary Tables 4.2.1–4.2.22) and 4.3 (pessary, Supplementary Tables 4.3.1–4.3.24). Meta-analyses are presented in Supplementary material in Appendix 6.1 (progesterone, Supplementary Figures 1–33), 6.2 (cerclage, Supplementary Figures 1–21), and 6.3 (pessary, Supplementary Figures 1–26).

#### Progesterone

##### Preterm birth across gestational weeks

**Figure 5:**
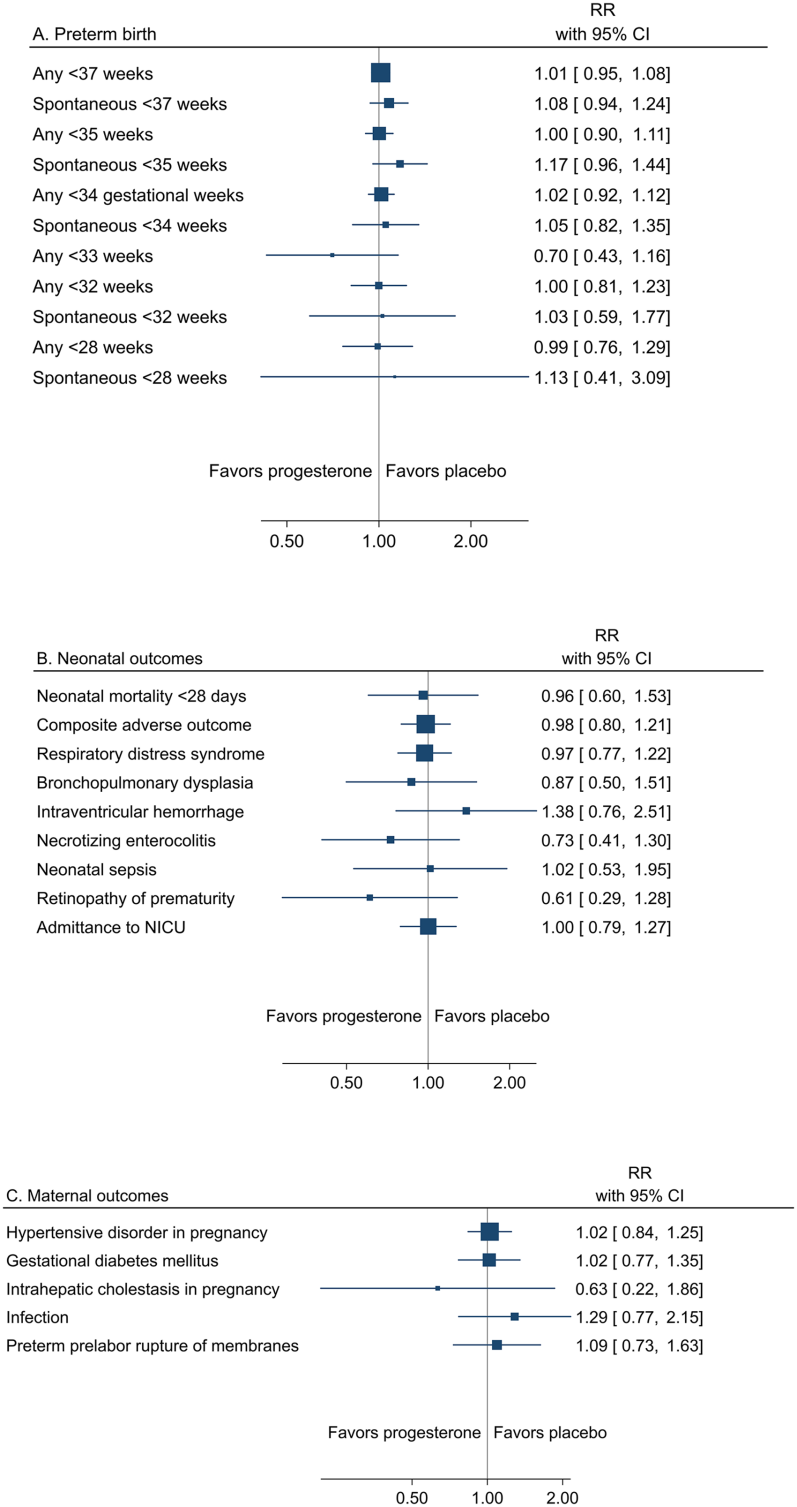
Summary graph of pooled estimates from meta-analyses comparing progesterone and placebo in women with a multifetal pregnancy with or without additional risk factor(s) for preterm birth, from trials with low risk of bias regarding **(A)** preterm birth, **(B)** neonatal, and **(C)** maternal outcomes.

A summary result per outcome and the associated certainty of the evidence for the main outcomes are presented in Table 6. The pooled estimates from meta-analyses of trials reporting any or spontaneous preterm birth (<37, <35, <34, <33, <32, and < 28 gestational weeks) from low risk of bias trials are summarized in Figure 5A. Low risk of bias trials demonstrated no effect of progesterone (any administration route) on the risk of any preterm birth <37 gestational weeks (58.5% vs. 56.6%, RR 1.03; 95% CI 0.97–1.08; moderate certainty of evidence), and <34 gestational weeks (22.8% vs. 21.5%, RR 1.02; 95% CI 0.93–1.13; high certainty of evidence), neither on <35, <32, <28 gestational weeks (high certainty of evidence), nor on the risk of spontaneous preterm birth (low to moderate certainty of evidence) (Table 6).

**Table 6:** Summary of findings for main outcomes (based on trials with low risk of bias) Progesterone (any administration route and dosage unless otherwise stated) vs placebo in women with a multifetal pregnancy with or without additional risk factor(s).

Outcomes	Number of RCTs (patients)	Absolute effect* n/N (%) intervention vs. control	Relative effect risk ratio (95% CI)	Risk difference** (95% CI)	Certainty of evidence GRADE
**Preterm birth (PTB)**					
Any PTB <37 weeks	10 (4517)	1396/2395 (58.3) vs. 1214/2122 (57.2)	1.01 (0.95 to 1.08)	−0.7 (−3.4 to 4.8)	⊕⊕⊕○ ^1^
Spontaneous PTB <37 weeks (vaginal progesterone)	3 (1189)	270/649 (41.6) vs. 200/540 (37.0)	1.08 (0.94 to 1.24)	3.4 (−2.2 to 9.0)	⊕⊕⊕○ ^2^
Any PTB <35 weeks	4 (954)	250/494 (50.6) vs. 212/460 (46.1)	1.00 (0.90 to 1.11)	1.2 (−4.6 to 7.0)	⊕⊕⊕⊕
Spontaneous PTB <35 weeks (17-OHPC)	2 (788)	135/395 (34.2) vs. 113/393 (28.8)	1.17 (0.96 to 1.44)	5.1 (−1.3 to 11.5)	⊕⊕⊕○ ^3^
Any PTB <34 weeks	15 (5257)	625/2788 (22.4) vs. 533/2469 (21.6)	1.02 (0.92 to 1.12)	0.6 (−1.6 to 2.7)	⊕⊕⊕⊕
Spontaneous PTB <34 weeks	4 (2372)	149/1224 (12.2) vs. 134/1148 (11.7)	1.05 (0.82 to 1.35)	0.7 (−1.6 to 3.6)	⊕⊕⊕⊕
Any PTB <33 weeks (vaginal progesterone)	6 (95)	20/52 (38.5) vs. 24/43 (55.8)	0.70 (0.43 to 1.16)	29.4 (−58.9 to 0.1)	⊕⊕○○ ^4^
Any PTB <32 weeks	10 (4581)	293/2441 (12.0) vs. 252/2140 (11.8)	1.00 (0.81 to 1.23)	0.3 (−2.1 to 2.8)	⊕⊕⊕⊕
Spontaneous PTB <32 weeks (vaginal progesterone)	1 (1100)	25/554 (4.5) vs. 24/546 (4.4)	1.03 (0.59 to 1.77)	0.1 (−2.3 to 2.6)	⊕⊕○○ ^4^
Any PTB <28 weeks	10 (4578)	118/2441 (4.8) vs. 105/2137 (4.9)	0.99 (0.76 to 1.29)	0.2 (−0.9 to 1.3)	⊕⊕⊕⊕
Spontaneous PTB <28 weeks (vaginal progesterone)	1 (1126)	8/567 (1.4) vs. 7/559 (1.3)	1.13 (0.41 to 3.09)	0.2 (−1.2 to 1.5)	⊕⊕○○ ^4^
**Neonatal mortality and morbidity**					
Neonatal mortality <28 days	10 (6869)	58/3764 (1.5) vs. 52/3105 (1.7)	0.96 (0.60 to 1.53)	0.0 (−0.6 to 0.7)	⊕⊕⊕○ ^5^
Composite adverse neonatal outcome	7 (4230)	471/2445 (19.3) vs. 331/1785 (18.5)	0.98 (0.80 to 1.21)	−0.5 (−4.5 to 3.5)	⊕⊕○○ ^6^
Respiratory distress syndrome	8 (5142)	417/2789 (15.0) vs. 326/2353 (13.9)	0.97 (0.77 to 1.22)	−0.5 (4.0 to 2.9)	⊕⊕⊕○ ^7^
Bronchopulmonary dysplasia	6 (3175)	64/1825 (3.5) vs. 50/1350 (3.7)	0.87 (0.50 to 1.51)	−0.2 (−2.7 to 2.3)	⊕⊕○○ ^8^
Intraventricular hemorrhage	7 (4559)	30/2396 (1.3) vs. 18/2163 (0.8)	1.38 (0.76 to 2.51)	0.4 (−0.1 to 0.9)	⊕⊕○○ ^9^
Necrotizing enterocolitis	8 (5137)	25/2786 (0.9) vs. 25/2351 (1.1)	0.73 (0.41 to 1.30)	−0.1 (−0.5 to 0.2)	⊕⊕○○ ^9^
Neonatal sepsis	7 (4968)	98/2700 (3.6) vs. 79/2268 (3.5)	1.02 (0.53 to 1.95)	0.0 (−2.0 to 2.0)	⊕⊕○○ ^10^
Retinopathy of prematurity	6 (3565)	20/1995 (1.0) vs. 23/1570 (1.5)	0.61 (0.29 to 1.28)	−0.2 (−1.3 to 0.8)	⊕⊕○○ ^11^
Admittance to neonatal intensive care unit	5 (4968)	755/2568 (29.4) vs. 745/2400 (31.0)	1.00 (0.79 to 1.27)	−0.4 (−6.6 to 5.9)	⊕⊕⊕○ ^12^
**Maternal morbidity**					
Hypertensive disorder in pregnancy	10 (4502)	264/2352 (11.2) vs. 221/2150 (10.3)	1.02 (0.84 to 1.25)	0.6 (−1.1 to 2.3)	⊕⊕⊕⊕
Gestational diabetes mellitus	8 (3268)	108/1782 (6.1) vs. 82/1486 (5.5)	1.02 (0.77 to 1.35)	0.1 (−1.4 to 1.5)	⊕⊕⊕○ ^13^
Intrahepatic cholestasis (vaginal prog)	4 (2535)	18/1310 (1.4) vs. 28/1225 (2.3)	0.63 (0.22 to 1.86)	−0.4 (−3.1 to 2.3)	⊕⊕○○^13^
Infection	5 (1773)	35/945 (3.7) vs. 23/828 (2.8)	1.29 (0.77 to 2.15)	0.9 (−0.5 to 2.3)	⊕⊕○○^13^
Preterm prelabor rupture of membranes	4 (1279)	51/732 (7.0) vs. 40/547 (7.3)	1.09 (0.73 to 1.63)	0.4 (−2.2 to 3.0)	⊕⊕⊕○^5^

*Absolute effects for event rates in intervention group vs. control group, presented as the sum of all events /the total numbers of participants, across the RCTs with low risk of bias. **Pooled risk difference (95% CI), presented as the weighted difference in event rates per 100 participants.

Reasons for downgrading: ^1^ One level based on some inconsistency (I^2^ 38%) and some indirectness (high proportion IVF pregnancies). ^2^ One level based on serious indirectness (34–95% IVF pregnancies). ^3^ One level based on uncertain directness (unclear selection process) and uncertain precision. ^4^ Two levels based on very serious imprecision. ^5^ One level based on serious imprecision. ^6^ Two levels based on some variation in the definition of the composite outcome, and serious inconsistency (I^2^ 61%). ^7^ One level based on various definitions of the outcome and serious inconsistency (I^2^ 64%). ^8^ Two levels based on some study limitations and inconsistency (I^2^ 48%) and serious imprecision. ^9^ Two levels based on some study limitations and serious imprecision. ^10^ Two levels based on various definitions of the outcome, serious inconsistency (I^2^ 75%) and uncertain precision. ^11^ Two levels based on some study limitations, inconsistency (I^2^ 29%), and serious imprecision. ^12^ One level based on serious inconsistency (I^2^ 84%). ^13^ One level based on some imprecision and no definition of GDM in most trials. ^14^ Two levels based on various definitions of the outcome and serious imprecision.

Exploratory subgroup analyses performed for the administration route (vaginal progesterone or im 17-OHPC) did not demonstrate any obviously more efficacious route (Supplementary material, Appendix 6.1, Supplementary Table S2, Supplementary Figures 29–30). In addition, exploratory subgroup analyses according to a specific risk factor (history of preterm birth or short cervical length) did not demonstrate any benefit from progesterone for any of the groups (Supplementary material, Appendix 6.1, Supplementary Table S2, Supplementary Figures 31–33).

##### Neonatal mortality and morbidity

Pooled estimates from meta-analyses of low risk of bias trials reporting mortality and morbidity in neonates from multifetal pregnancies are summarized in Figure 5B.

Neonatal mortality within 28 days (1.5% vs. 1.7%, RR 0.96; 95% CI 0.60 to 1.53), respiratory distress syndrome, and admittance to NICU was not affected by progesterone (moderate certainty of evidence) (Table 6), and neither were the other neonatal morbidity outcomes (low certainty of evidence).

##### Maternal mortality and morbidity

Pooled estimates from meta-analyses of low risk of bias trials reporting maternal morbidity are summarized in Figure 5C. Maternal morbidity outcomes were not significantly affected by progesterone (low to high certainty of evidence) (Table 6).

##### Long-term child outcomes

Long-term child outcome is presented in Supplementary material, Appendix 4.1, Supplementary Table 4.1.20. Three trials (3,030 children) examined long-term child outcomes in twins (Rode et al. (98) PREDICT-study, follow up at 6- and 18-months of age, Vedel et al. (116) follow up to 8 years of age [follow up of Rode et al. (98) PREDICT-study], McNamara et al. (81) follow up at three to 6 years of age [follow up of Norman et al. (86) STOPPIT-study]. Rode et al. (98) and Vedel et al. (116) measured neurodevelopment at different ages with Ages and Stages Questionnaire (ASQ), and McNamara et al. (81) used Child Developmental Inventory (CDI) score. Follow-up rate was between 44 and 80%. The groups had similar cognitive development, general health, anthropometry, and behavior. No meta-analysis was performed because of the heterogeneity of the studies.

#### Cerclage

**Figure 6:**
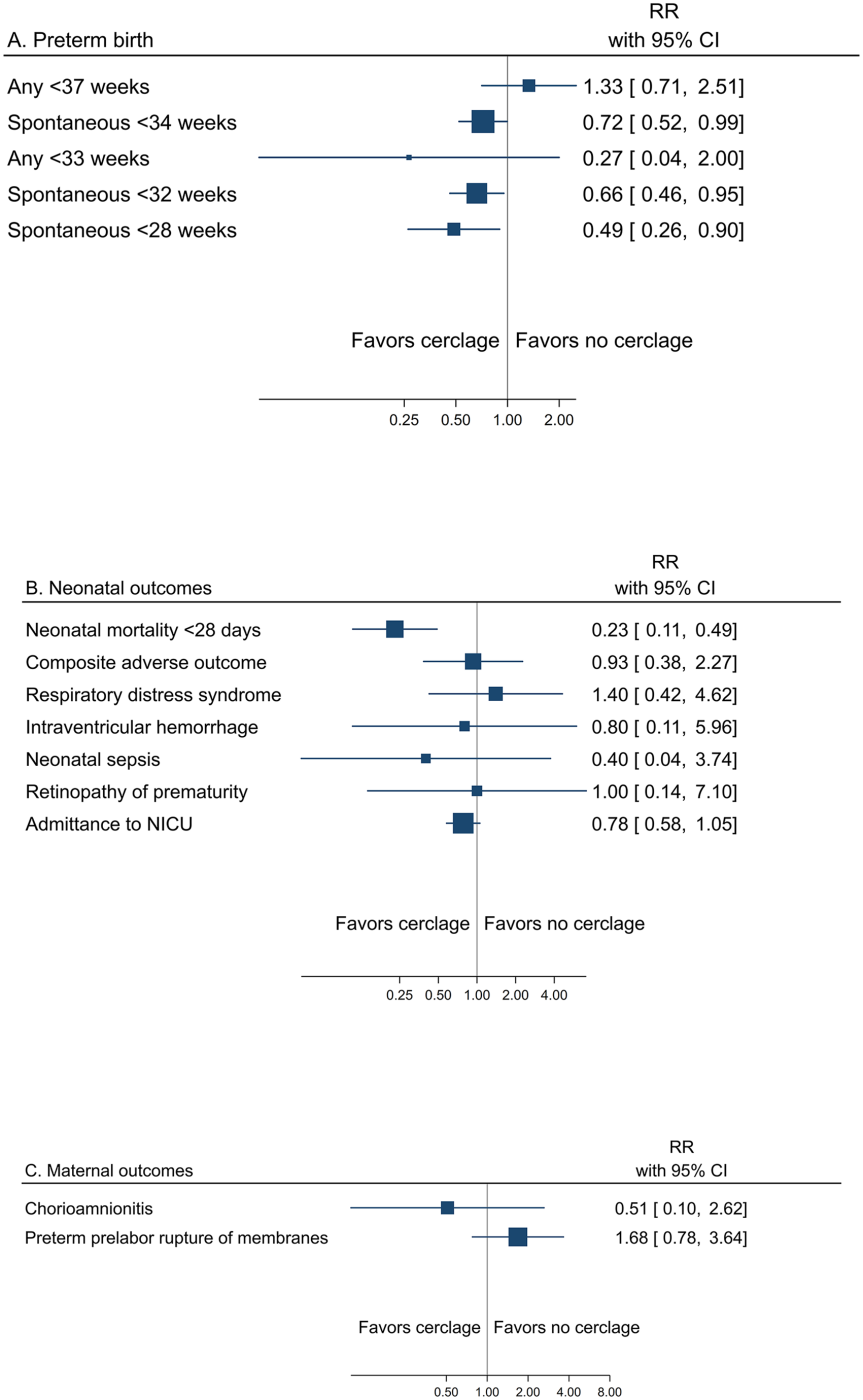
Summary graph of pooled estimates from meta-analyses comparing cerclage versus no cerclage in women with a multifetal pregnancy with or without additional risk factor(s) for preterm birth from trials with low risk of bias regarding **(A)** preterm birth, **(B)** neonatal, and **(C)** maternal outcomes.

A summary result per outcome and the associated certainty of the evidence for the main outcomes are presented in Table 7. The pooled estimates from meta-analyses of trials reporting any or spontaneous preterm birth (<37, <35, <34, <33, <32, and < 28 gestational weeks), neonatal, and maternal outcomes from low risk of bias trials are summarized in Figure 6. Any effect on preterm birth below any gestational week was uncertain (very low certainty of evidence) (Table 7 and Figure 6A).

**Table 7:** Summary of findings for main outcomes (based on trials with low risk of bias): Cerclage vs no cerclage in women with a multifetal (twin) pregnancy with or without additional risk factor(s).

Outcomes	Number of RCTs (patients)	Absolute effect* n/N (%) intervention vs. control	Relative effect risk ratio (95% CI)	Risk difference** (95% CI)	Certainty of evidence GRADE
**Preterm birth (PTB)**					
Any PTB <37 weeks	1 (28)	8/12 (66.7) vs. 8/16 (50.0)	1.33 (0.71 to 2.51)	16.7 (−19.6 to 52.9)	⊕○○○ ^1^
Spontaneous PTB <34 weeks	1 (30)	12/17 (70.6) vs. 13/13 (100)	**0.72 (0.52 to 0.99)**	**−29.4 (−52.8 to −6.0)**	⊕○○○ ^2^
Any PTB <33 weeks	1 (28)	1/12 (8.3) vs. 5/16 (31.3)	0.27 (0.04 to 1.99)	−22.9 (−50.5 to 4.7)	⊕○○○ ^1^
Spontaneous PTB <32 weeks	1 (30)	11/17 (64.7) vs. 13/13 (100)	**0.66 (0.46 to 0.95)**	**−35.3 (−59.2 to −11.1)**	⊕○○○ ^2^
Spontaneous PTB <28 weeks	1 (30)	7/17 (41.2) vs. 11/13 (84.6)	**0.49 (0.26 to 0.90)**	**−43.4 (−74.0 to −12.9)**	⊕○○○ ^2^
**Peri/neonatal mortality and neonatal morbidity**					
Perinatal mortality (Macnaughton, 1993)	1 (56)	2/24 (8.3) vs. 2/32 (6.3)	1.33 (0.20 to 8.80)	2.1 (−11.8 to 16.0)	⊕○○○ ^1^
Perinatal mortality (Roman, 2020)	1 (60)	6/34 (17.6) vs. 20/26 (76.9)	**0.23 (0.11 to 0.49)**	**−59.3 (−79.9 to −38.6)**	⊕⊕○○ ^3^
Neonatal mortality <28 days	1 (60)	6/34 (17.6) vs. 20/26 (76.9)	**0.23 (0.11 to 0.49)**	**−59.3 (−79.9 to −38.6)**	⊕⊕○○ ^3^
Composite adverse neonatal outcome	1 (36)	14/30 (46.7) vs. 3/6 (50.0)	0.93 (0.38 to 2.27)	−3.3 (−47.1 to 40.5)	⊕○○○ ^2^
Respiratory distress syndrome	1 (36)	14/30 (46.7) vs. 2/6 (33.3)	1.4 (0.42 to 4.62)	13.3 (−28.4 to 55.1)	⊕○○○ ^2^
Intraventricular hemorrhage	1 (36)	4/30 (13.3) vs. 1/6 (16.7)	0.8 (0.11 to 5.96)	−3.3 (−35.5 to 28.9)	⊕○○○ ^2^
Necrotizing enterocolitis	1 (36)	0 vs. 0	–	–	⊕○○○ ^2^
Neonatal sepsis	1 (36)	2/30 (6.7) vs. 1/6 (16.7)	0.4 (0.04 to 3.74)	10.1 (−41.1 to 21.1)	⊕○○○ ^2^
Retinopathy of prematurity	1 (36)	5/30 (16.7) vs. 1/6 (16.7)	1 (0.14 to 7.10)	0 (−32.7 to 32.7)	⊕○○○ ^2^
Admittance to neonatal intensive care unit	1 (36)	22/30 (73.3) vs. 6/6 (100)	0.78 (0.58 to 1.05)	27.7 (−51.4 to −2.0)	⊕⊕○○ ^3^
**Maternal morbidity**					
Chorioamnionitis	1 (30)	2/17 (11.8) vs. 3/13 (23.1)	0.51 (0.10 to 2.62)	−11.3 (−38.9 to 16.2)	⊕○○○ ^2^
Preterm prelabor rupture of membranes	1 (30)	11/17 (64.7) vs. 5/13 (38.5)	1.68 (0.78 to 3.64)	26.2 (−8.6 to 61.1)	⊕○○○ ^2^

*Absolute effects for event rates in intervention group vs. control group, presented as the sum of all events /the total numbers of participants, across the RCTs with low risk of bias. ** Pooled risk difference (95% CI), presented as the weighted difference in event rates per 100 participants.

Reasons for downgrading: ^1^ Three levels based on some study limitations (open trial), and some indirectness (old trial, ethnicity discrepancies), and very serious imprecision (few events, crossing unity). ^2^ Three levels based on some study limitations (open trial), and some indirectness (high risk population with dilated cervix and bulging membranes, Roman 2020), and very serious imprecision (few events). ^3^ Two levels based on some study limitations (open trial), some indirectness (high risk population with dilated cervix and bulging membranes, Roman 2020) and serious imprecision (few events).

Peri/neonatal mortality may be reduced by cerclage in a high-risk group of women with a twin pregnancy, dilated cervix, and visible membranes (17.6% vs. 76.9%, RR 0.23; 95% CI 0.11 to 0.49) (low certainty of evidence). However, neonatal morbidity outcomes were not significantly affected by cerclage (very low to low certainty of evidence) (Table 7 and Figure 6B).

Maternal morbidity outcomes were not significantly affected by cerclage (very low certainty of evidence) (Table 7 and Figure 6C).

No trial has been reported on long-term child outcomes.

#### Pessary

**Figure 7:**
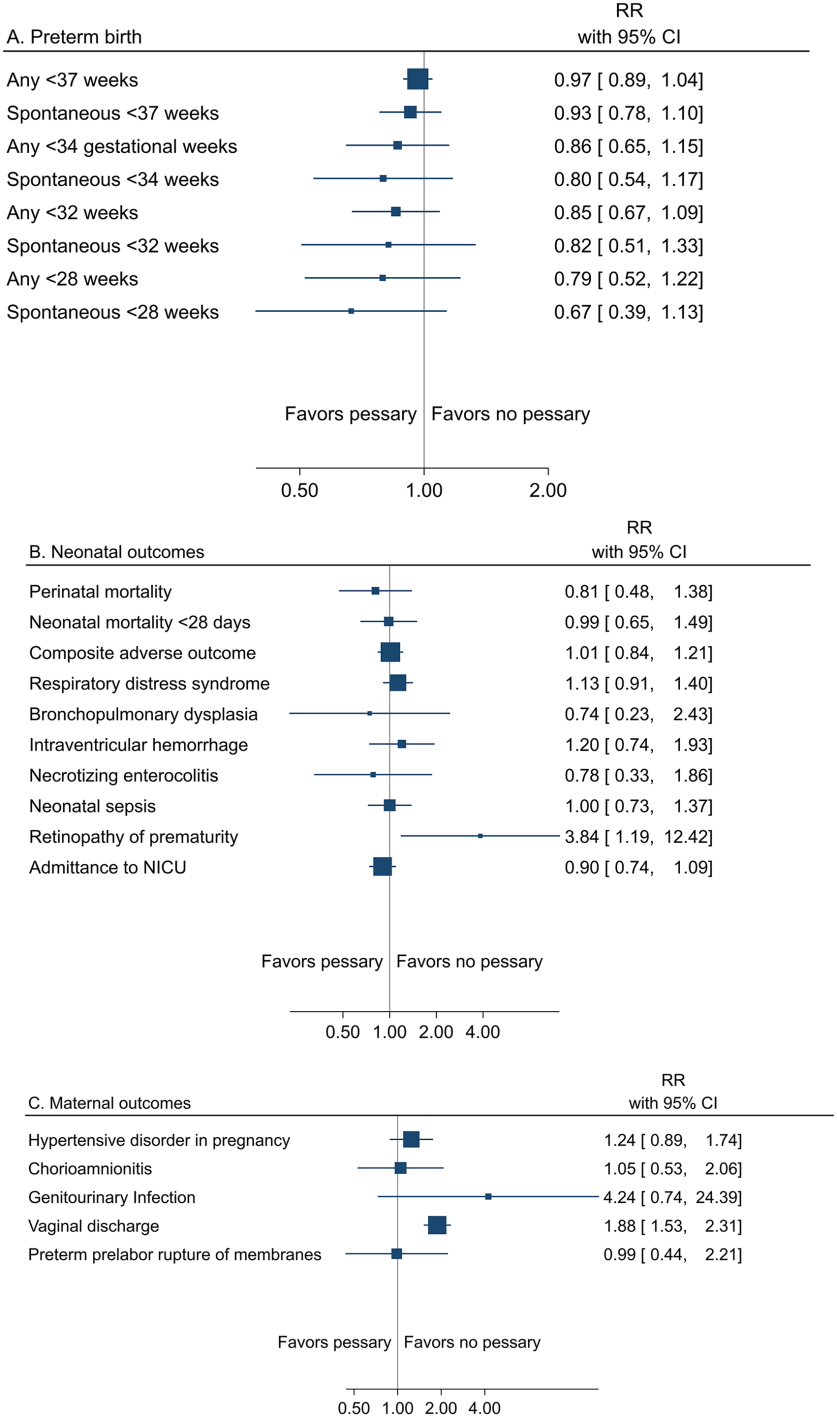
Summary graph of pooled estimates from meta-analyses comparing pessary versus no pessary in women with a multifetal pregnancy with or without additional risk factor(s) for preterm birth from trials with low risk of bias regarding **(A)** preterm birth, **(B)** neonatal, and **(C)** maternal outcomes.

A summary result per outcome and the associated certainty of evidence for the main outcomes are presented in Table 8. The pooled estimates from meta-analyses of trials reporting any or spontaneous preterm birth (<37, <35, <34, <33, <32, and < 28 gestational weeks), neonatal, and maternal outcomes from low risk of bias trials are summarized in Figure 7.

**Table 8:** Summary of findings for main outcomes (based on trials with low risk of bias): Pessary vs no pessary in women with a multifetal pregnancy with or without additional risk factor(s) for preterm birth.

Outcomes	Number of RCTs (patients)	Absolute effect* n/N (%) intervention vs. control	Relative effect risk ratio (95% CI)	Risk difference** (95% CI)	Certainty of evidence GRADE
**Preterm birth (PTB)**					
Any PTB <37 weeks	4 (1428)	433/717 (60.4) vs. 438/711 (61.6)	0.97 (0.89 to 1.04)	−2 (−7.0 to 2.9)	⊕⊕⊕○ ^1^
Spontaneous PTB <37 weeks	3 (683)	115/341 (33.7) vs. 126/342 (36.8)	0.93 (0.78 to 1.10)	−3.5 (−10.0 to 3.1)	⊕⊕⊕○ ^1^
Any PTB <34 weeks	5 (1931)	195/972 (20.1) vs. 205/959 (21.4)	0.86 (0.65 to 1.15)	−4.7 (−12.8 to 3.5)	⊕⊕○○ ^2^
Spontaneous PTB <34 weeks	4 (1860)	135/929 (14.5) vs. 155/931 (16.6)	0.8 (0.54 to 1.17)	−5.2 (−13.5 to 3.2)	⊕⊕○○ ^3^
Any PTB <32 weeks	4 (2559)	133/1282 (10.4) vs. 152/1277 (11.9)	0.85 (0.67 to 1.09)	−1.6 (−4.9 to 1.6)	⊕⊕⊕○ ^1^
Spontaneous PTB <32 weeks	1 (503)	26/250 (10.4) vs. 32/253 (12.6)	0.82 (0.51 to 1.33)	−2.3 (−7.8 to 3.3)	⊕⊕○○ ^4^
Any PTB <28 weeks	5 (2605)	58/1305 (4.4) vs. 71/1300 (5.5)	0.79 (0.52 to 1.22)	−1.3 (−4.3 to 1.7)	⊕⊕○○ ^2^
Spontaneous PTB <28 weeks	3 (683)	21/341 (6.2) vs. 32/342 (9.4)	0.67 (0.39 to 1.13)	−3.1 (−6.9 to 0.8)	⊕⊕○○ ^4^
**Peri/neonatal mortality and neonatal morbidity**					
Perinatal mortality	2 (2183)	24/1088 (2.2) vs. 30/1095 (2.7)	0.81 (0.48 to 1.38)	−0.7 (−1.8 to 0.5)	⊕⊕○○ ^4^
Neonatal mortality <28 days	4 (4346)	44/2169 (2.0) vs. 45/2177 (2.1)	0.99 (0.65 to 1.49)	0.1 (−0.6 to 0.8)	⊕⊕○○ ^4^
Composite adverse neonatal outcome	5 (5291)	169/1357 (12.5) vs. 176/1375 (12.8)	1.01 (0.84 to 1.21)	−0.3 (−2.4 to 1.9)	⊕⊕⊕○ ^1^
Respiratory distress syndrome	4 (4285)	164/2140 (7.7) vs. 145/2145 (6.8)	1.13 (0.91 to 1.40)	0.8 (−0.6 to 2.3)	⊕⊕⊕○ ^1^
Bronchopulmonary dysplasia	3 (2732)	12/1357 (0.9) vs. 17/1375 (1.2)	0.74 (0.23 to 2.43)	−0.2 (−1.5 to 1.0)	⊕⊕○○ ^5^
Intraventricular hemorrhage	5 (5291)	37/2640 (1.4) vs. 33/2651 (1.2)	1.2 (0.74 to 1.93)	0.2 (−0.5 to 0.8)	⊕⊕○○ ^6^
Necrotizing enterocolitis	5 (5291)	19/2640 (0.7) vs. 25/2651 (0.9)	0.78 (0.33 to 1.86)	−0.3 (−1.2 to 0.5)	⊕⊕○○ ^2^
Neonatal sepsis	5 (5291)	103/2640 (3.9) vs. 103/2651 (3.9)	1 (0.73 to 1.37)	0.1 (−0.9 to 1.2)	⊕⊕⊕○ ^1^
Retinopathy of prematurity	3 (2651)	13/1329 (1.0) vs. 3/1322 (0.2)	**3.84 (1.19 to 12.42)**	**0.7 (0.1 to 1.3)**	⊕○○○ ^7^
Admittance to neonatal intensive care unit	2 (2640)	174/1311 (13.3) vs. 196/1329 (14.7)	0.9 (0.74 to 1.09)	−1.5 (−4.1 to 1.2)	⊕⊕⊕○ ^8^
**Maternal morbidity**					
Hypertensive disorder in pregnancy	1 (808)	65/401 (16.2) vs. 53/407 (13.0)	1.24 (0.89 to 1.74)	3.2 (−1.7 to 8.1)	⊕⊕○○ ^4^
Chorioamnionitis	3 (988)	18/493 (3.7) vs. 16/495 (3.2)	1.05 (0.53 to 2.06)	0.6 (−3.1 to 4.2)	⊕⊕○○ ^9^
Genitourinary infection	2 (854)	7/425 (1.6) vs. 1/419 (0.2)	4.24 (0.74 to 24.39)	1.4 (−2.5 to 5.4)	⊕○○○^10^
Vaginal discharge	2 (180)	87/91 (95.6) vs. 45/89 (50.6)	**1.88 (1.53 to 2.31)**	**45.5 (34.6 to 56.5)**	⊕⊕⊕○ ^11^
Preterm prelabor rupture of membranes	4 (1491)	52/742 (7.0) vs. 50/749 (6.7)	0.99 (0.44 to 2.21)	−0.6 (−5.3 to 4.2)	⊕⊕○○ ^4^

Numbers in bold if difference is statistically significant. *Absolute effects for event rates in intervention group vs. control group, presented as the sum of all events /the total numbers of participants, across the RCTs with low risk of bias. **Pooled risk difference (95% CI), presented as the weighted difference in event rates per 100 participants.

Reasons for downgrading: ^1^ One level based on lack of blinding and uncertain precision. ^2^ Two levels based on lack of blinding, some inconsistency, serious imprecision. ^3^ Two levels based on lack of blinding, some inconsistency, uncertain precision. ^4^ Two levels based on lack of blinding, serious imprecision. ^5^ Two levels based on serious inconsistency and imprecision. ^6^ Two levels based on lack of blinding, different outcome definitions, serious imprecision. ^7^ Three levels based on lack of blinding, lack of classification of outcome, very serious imprecision. ^8^ One level based on lack of blinding, possible different routines regarding admittance to NICU, uncertain precision. ^9^ Two levels based on very serious imprecision. ^10^ Three levels based on lack of blinding, poorly defined outcome, very serious imprecision. ^11^ One level based on lack of blinding and subjective outcome.

Low risk of bias trials demonstrated no effect of pessary on the risk of preterm birth, assessed from <37 down to <28 gestational weeks (low to moderate certainty of evidence) for all gestational weeks (Table 8 and Figure 7A) (any preterm birth <37 gestational weeks 60.4% vs. 61.6%, RR 0.97; 95% CI 0.89 to 1.04, and any preterm birth <34 gestational weeks 20.1% vs. 21.4%, RR 0.86; 95% CI 0.65 to 1.15).

Subgroup analyses according to risk factors, of which only short cervical length was applicable, did not affect the results for any preterm birth <37 or < 34 gestational weeks (Appendix 6.3, Supplementary Figures 25–26).

No effect on peri/neonatal mortality and the composite adverse neonatal outcome was demonstrated with pessary (low to moderate certainty of evidence) (Table 8 and Figure 7B) (neonatal mortality 2.0% vs. 2.1%, RR 0.99; 95% CI 0.65 to 1.49, low certainty of evidence).

One trial (77) reported one maternal death in the intervention group. The treatment was a cerclage instead of a pessary, and death occurred later due to chorioamnionitis. Maternal morbidity was not affected by pessary (very low to low certainty of evidence), except for an increased risk of vaginal discharge (moderate certainty of evidence) (Table 8 and Figure 7C).

##### Long-term child outcomes in multifetal pregnancies

Two articles (714 children) examined long-term child outcomes in twins and triplets (112, 114) (Supplementary material, Appendix 4.3, Supplementary Table 4.3.18).

Both articles are a follow up of the ProTWIN trial (77). The follow-up rate was 45% (112) and 83% (114). A meta-analysis was not feasible due to different outcomes. Van’t Hooft et al. (114) showed no difference in neurodevelopment assessed by the Bayley-III Cognitive Composite score at 3 years, nor were there any differences in cognitive, language, or motor development. Simons et al. (112) concluded no improvement in the development, behavioral, or physical outcomes of surviving children after four years.

### Comparison of other interventions vs. progesterone in multifetal pregnancies

One trial with low risk of bias (Table 2) included 300 women with a twin pregnancy and short cervical length (less than 38 mm, 10th percentile), and compared pessary with vaginal progesterone (400 mg daily) (53). The trial was performed in Vietnam. Outcome tables are presented in Supplementary material in Appendix 4.1.1–4.1.26, after the progesterone vs. placebo trials.

#### Effect of pessary vs. vaginal progesterone in twin pregnancies

The Dang trial showed no difference in the primary outcome of preterm birth <34 weeks, comparing pessary with vaginal progesterone, RR 0.73 (95% CI 0.46 to 1.18) (53). The certainty of evidence was downgraded by two levels due to non-blinding, subpopulation of mainly IVF pregnancies and very serious imprecision (low certainty of evidence for no difference in preterm birth <34 weeks). The pessary group had lower rates of adverse neonatal outcomes.

## Discussion

### Main findings

In singleton asymptomatic pregnant women at high risk of preterm birth, progesterone and probably also cerclage have a protective effect against preterm birth. Neither pessary nor ASA showed any convincing protective effect.

In the subgroup analyses, vaginal progesterone appears to be the best prevention for preterm birth <34 gestational weeks in asymptomatic women with a singleton pregnancy and the risk factor short cervix. Another subgroup analysis displayed that weekly 17-OHPC injections did not decrease the risk of preterm birth. Trials on oral progesterone were small but showed a protective effect.

There were few trials on cerclage to prevent preterm birth in singleton pregnant women. The largest trial was old, including women with heterogeneous risk factors and no cervical length screening. Only two trials used universal cervical screening to identify women at high risk of preterm birth. Thus, the target population for treatment may differ between progesterone and cerclage. There is evident ambiguity regarding indications for cerclage. Cerclage is a more complex intervention compared with progesterone. It is an invasive procedure and considerably more costly than progesterone. In addition, by the nature of the intervention, it is not possible to blind participants and practitioners. Due to these disadvantages, cerclage has been less studied in recent years compared with progesterone. When comparing the trials, trials with progesterone are also more commonly funded by pharmaceutical companies. There is a gap in knowledge to which subgroup of women at risk of preterm birth that would profit from cerclage instead of progesterone. Suggested target population for trials on cerclage vs. progesterone may include women with clinically diagnosed cervical insufficiency, i.e., several late miscarriages or preterm births with painless cervical dilation. These women are today subject for cerclage but without convincing evidence in the literature. We did not find any trials comparing vaginal progesterone with cerclage. No trial reported clear harm with neither progesterone nor cerclage, an important result for women and caregivers.

Multifetal pregnancies carry a much higher risk of preterm birth than singleton pregnancies, with an approximately 50% incidence of preterm birth in twins. In multifetal pregnancies, the impact of preventive interventions of progesterone, cerclage, or pessary is minimal, if any. In most trials on multifetal pregnancies, the only risk factor was the multifetal pregnancy. In an extreme high-risk population, cerclage may reduce the risk of perinatal mortality. There is no evidence to support the use of progesterone, cerclage, or pessary in unselected multifetal pregnancies to prevent preterm birth or to improve neonatal outcomes.

### Results in context

In the literature search process, 39 recent systematic reviews relevant to our PICO and published between 2017 and 2022 were identified. Our results are generally consistent with previous systematic reviews. Two recent systematic reviews (119, 120) showed that vaginal progesterone did not prevent recurrent preterm delivery in mainly unselected asymptomatic women, which is in alignment with our findings. However, another recent systematic review with a network meta-analysis including progesterone, cerclage, and pessary in singletons, concluded that vaginal progesterone should be the choice in women with a previous preterm delivery or a short cervical length (121). A similar systematic review with a network meta-analysis on twins found no significant effect on the rate of preterm birth or neonatal morbidity for any of the interventions (122). Conflicting results and conclusions, compared with ours, regarding vaginal progesterone in women with a twin pregnancy and short cervix were reached by Romero et al. (102) solely based on different effect models in the meta-analyses. We believe our results are more robust using a random effect model compared to the fixed effect models used by Romero et al. (102).

### Clinical implications

This systematic review summarizes the most recent evidence on the effectiveness and safety of commonly used interventions for prevention of preterm birth. There is convincing evidence that progesterone but also cerclage are effective interventions in singleton pregnancies. However, except for short cervical length in asymptomatic women, the inclusion criteria in many trials are mixed. It is therefore unclear which women who will benefit the most from treatment. Preterm birth is also a heterogenous condition which requires individualized management. We encourage healthcare professionals to create new or update national clinical practice guidelines on these preventive strategies being aware of these limitations. Reliable clinical practice guidelines should be based on a systematic review, provide ratings of the certainty of evidence and the strength of recommendations, as well as consider patient values and economic aspects (123, 124). This review may provide a scientific basis for clinical practice guidelines.

All interventions for preterm birth prevention imply identifying women at high risk of preterm birth. Traditionally this is based on an accurate history of maternal and pregnancy-related risk factors. Unfortunately, available risk-scoring systems based on history have a low detection rate and a high false-positive rate (125, 126). An alternative strategy may be screening with transvaginal ultrasound to identify women with short cervical length in the second trimester. A recent Swedish blinded prospective observational multicentre study comprising more than 11,000 women with a singleton pregnancy showed that the diagnostic performance of transvaginal ultrasound screening in the second trimester was as best moderate (13). Assuming a 30% relative reduction of spontaneous preterm birth with progesterone or cerclage in any of the screened groups, the potential effect would be more significant by universal screening for short cervix compared with risk factor-based screening. An economic analysis concluded that any screening strategy followed by vaginal progesterone to women at risk was cost-effective (14). However, before implementation, the benefits and harms of universal cervical length screening need further evaluation.

### Research implications

In preterm birth prevention, multiple areas are subject to debate, mainly due to lack of clinical evidence.

Although vaginal progesterone has been studied extensively, there are still areas for further research. The effect of vaginal progesterone against preterm birth is best documented in women with a short cervix (≤25 mm) detected on ultrasound screening. The best cut-off for short cervical length has been debated, and a recent study has shown it to be 29 mm at 18–20 gestational weeks and 27 mm at 21–23 gestational weeks (13). However, there is uncertainty regarding the effect of vaginal progesterone in asymptomatic women with an ultrasound-screened cervical length between 25 and 30 mm. The EPPPIC trial concluded that progesterone also works when the cervical length is between 25 and 30 mm, but the number of included women was small (56). There is also some unclarity whether women with a previous preterm birth and normal cervical length should be offered progesterone (56, 127, 128). The same applies to whether progesterone works for other risk factors of preterm birth, e.g., cervical conization (129) or for newly identified risk factors as a history of a second stage caesarean delivery (130, 131). Other aspects that need to be clarified are if vaginal progesterone and weekly 17-OHPC injections are equally effective to prevent recurrent preterm birth (132, 133), the dosage and treatment interval of vaginal progesterone, if oral progesterone is effective, and whether progesterone should be offered to subgroups of women carrying a multifetal pregnancy with concomitant risk factors for preterm birth such as a short cervix or previous preterm birth. The long-term health consequences for the offspring need further studies, e.g., a recent cohort study found a possible link between 17-OHPC exposure in early pregnancy and cancer in the offspring (134). Further studies are needed to clarify all these issues.

Regarding cerclage, included trials were heterogenous regarding risk factors for preterm birth in the population. Therefore, additional trials are needed to clarify the proper indications for cerclage and its relation to preterm birth prevention by investigating specific subgroups of women at risk to target the population that would benefit the most from the intervention. This is a challenging area of research since it has been a common practice in many clinics for several decades (21). A recent study comparing vaginal and abdominal cerclage for previously failed cerclage indicates an advantage for the latter (135), but further trials are needed to verify these findings.

Regarding pessary, the results from different research groups are conflicting (61, 85). Thus, further research is needed to identify which women, if any, might benefit from pessary as an intervention for preterm birth prevention (23).

A large trial performed in low- and middle-income countries, including nulliparous women with singleton pregnancies and no other specified risk factors, found that low-dose aspirin initiated in the first trimester reduced the risk of any preterm birth <37 weeks and perinatal mortality (26). In addition, a recent observational register study among women with a previous preterm birth suggested that low-dose aspirin was associated with a reduced risk for recurrent spontaneous preterm birth (136). However, further trials are needed to clarify if low-dose aspirin enhances preterm birth prevention in other populations outside the area of preeclampsia prevention.

Further studies are also needed regarding different combinations of prevention strategies for preterm birth prevention. Some authors have suggested that using a placebo as a comparator in future trials is challenging and have indicated that the new golden standard for intervention to prevent preterm delivery should be vaginal progesterone (121). This might be right for screened short cervical length ≤ 25 mm for singleton pregnancies.

Finally, researchers should be encouraged to harmonize reporting of outcome data and use the recommended core outcome set that has been recommended for evaluations of interventions to prevent preterm birth (137). This will facilitate future individual patient data analyses and allow adequately powered subgroup analyses. Importantly, long-term neurodevelopment, a critical knowledge gap, is now included as an essential clinical outcome.

### Strengths and limitations

In this comprehensive report counting 23,886 women and 32,893 offspring, all 71 trials fulfilling inclusion criteria were used in the assessment. For reliability, conclusions were only based on 50 original trials with low risk of bias and their secondary publications. We consider our review to be broadly applicable, despite having considered differences in ethnicity, which only marginally affected downgrading certainty of evidence. The vast majority were trials on progesterone versus placebo (*n* = 29). There are also a few limitations. Most trials used any preterm birth as the primary outcome. Spontaneous preterm birth is a more clinically relevant outcome than any preterm birth because it may be possible to identify women at risk (either with a history of a prior spontaneous preterm birth or a short cervical length) with subsequent treatment and prevention. Still, few trials reported on spontaneous preterm birth. Many of our meta-analyses included several trials with sparse data, yielding low precision with wide CIs and uncertainty of results. Meta-analyses on long-term children follow-up data were not feasible due to the heterogeneity of data. Some early trials were not registered or registered after trial completion, and some did not report the predefined primary outcome. These trials may be biased by selective outcome reporting (so-called p-hacking) and distortion of results (138). Another problem is the increasing concern about falsified or fabricated RCTs which may threaten the robustness of a systematic review (139). We identified and excluded one retracted article (140). We also identified several trials registered many years ago, but still unpublished, thus ongoing research and publication bias cannot be excluded. Finally, we did not compare the different interventions with each other, apart from including trials with direct comparisons.

## Conclusion

Progesterone and probably also cerclage have a protective effect against preterm birth and perinatal/neonatal mortality in singleton asymptomatic pregnant women at high risk of preterm birth. There is no documented effect on improvement of long-term child outcomes. Further trials of ASA are needed.

## Data availability statement

The original contributions presented in the study are included in the article/Supplementary material, further inquiries can be directed to the corresponding author.

## Author contributions

U-BW, LB, AS, BJ, and PK: conception and design. AL and A-CE: literature search and screening of abstracts. U-BW, LB, AS, BJ, PK, AM, EL, CH, and PS: reading full text articles, deciding on inclusion of articles, and collection and assembly of data. U-BW, LB, AS, BJ, PK, AM, EL, CH, PS, and MP: data analysis and interpretation. U-BW, LB, AS, BJ, PK, AM, EL, CH, PS, MP, and MS: manuscript writing and final approval of manuscript. All authors contributed to the article and approved the submitted version.

## Funding

PK the Swedish state under the agreement between the Swedish government and the county councils, the ALF-agreement (ALFGBG-977519), Forskning och utbilding (FoU) Södra Älvsborg. LB the Swedish state under the agreement between the Swedish government and the county councils, the ALF-agreement (ALFGBG-942685). The Wallenberg Center for Molecular and Translational Medicine (WCMTM). BJ the Swedish state under the agreement between the Swedish government and the county councils, the ALF-agreement (ALFGBG-965353). The funding sources had no role in study design, in the collection, analysis and interpretation of data, in the writing of the report, and in the decision to submit the article for publication.

## Conflict of interest

LB board member and responsible for the biobank in the IMPACT study where PlGF reagents have been donated by Roche, Perkin Elmer, and Thermo Fischer. Course leader for the course in Preeclampsia in Sweden with sponsorship by Thermo Fischer and Roche. Obtained reimbursement for lecture by iLab Medical and reimbursement as expert opinion from Homburg and Partner. Board member in intervention trials where the trial drug is donated by Merck. Associate member in FIGO for the working group of maternal longterm health. Chair of the Swedish Preeclampsia Working Group. Board member in CoLab. Author of the chapter about preeclampsia in “Obstetrik” and about pulmonary hypertension in “Internetmedicin.” Research grants from the Swedish Research Council, STINT, Märta Lundqvist stiftelse, Swedish Society of Medicine, Gothenburg Society of Medicine, SSMF, Jane and Dan Olssons Stiftelse, Swedish Brain fund, Jeanssons stiftelse and Wallenberg Center for Molecular and Translational Medicine. BJ Research grants from Swedish Research Council, Norwegian Research Council, March of Dimes, Burroughs Wellcome Fund and the US National Institute of Health. During the last years performed clinical diagnostic trials on NIPT with Ariosa (completed), Natera (ongoing), Vanadis (completed), and Hologic (ongoing) with expenditures reimbursed per patient. Also performed clinical probiotic studies with the probiotic product provided by FukoPharma (ongoing, no funding) and BioGaia (ongoing; also provided a research grant for the specific study). Previously (2018–2020) a collaborator in IMPACT study where Roche, Perkin Elmer and Thermo Fisher provided reagents to PLGF analyses. No lectures, presentations, travel or personal reimbursement has been financed by any company. Division Director of Maternal and Neonatal Health of the International Federation of Gynecology and Obstetrics (FIGO), Board member of the European Association of Perinatal Medicine and chair the EAPM special interest group of preterm delivery. Steering group member of Genomic Medicine Sweden and is in charge of the Genomic Medicine Sweden complex diseases group. Swedish representative in Nordic Society of Precision Medicine. U-BW, BJ, and PK declare being co-investigators in the OPPTIMUM trial, an included trial in this review.

The remaining authors declare that the research was conducted in the absence of any commercial or financial relationships that could be construed as a potential conflict of interest.

## Publisher’s note

All claims expressed in this article are solely those of the authors and do not necessarily represent those of their affiliated organizations, or those of the publisher, the editors and the reviewers. Any product that may be evaluated in this article, or claim that may be made by its manufacturer, is not guaranteed or endorsed by the publisher.

## Supplementary material

The Supplementary material for this article can be found online at: https://www.frontiersin.org/articles/10.3389/fmed.2023.1111315/full#supplementary-material


