# Corrigendum: Progesterone, cerclage, pessary, or acetylsalicylic acid for prevention of preterm birth in singleton and multifetal pregnancies - A systematic review and meta-analyses

**DOI:** 10.3389/fmed.2025.1561413

**Published:** 2025-02-14

**Authors:** Ulla-Britt Wennerholm, Lina Bergman, Pihla Kuusela, Elin Ljungström, Anna C. Möller, Cecilie Hongslo Vala, Ann-Catrin Ekelund, Ann Liljegren, Max Petzold, Petteri Sjögren, Mikael Svensson, Annika Strandell, Bo Jacobsson

**Affiliations:** ^1^Region Västra Götaland, Sahlgrenska University Hospital, Department of Obstetrics and Gynecology, Gothenburg, Sweden; ^2^Department of Obstetrics and Gynecology, Institute of Clinical Sciences, Sahlgrenska Academy, University of Gothenburg, Gothenburg, Sweden; ^3^Department of Obstetrics and Gynecology, Stellenbosch University, Cape Town, South Africa; ^4^Department of Women's and Children's Health, Uppsala University, Uppsala, Sweden; ^5^Region Västra Götaland, Södra Älvsborg Hospital, Department of Obstetrics and Gynecology, Borås, Sweden; ^6^Region Västra Götaland, HTA-centrum, Gothenburg, Sweden; ^7^Region Västra Götaland, Skaraborg Hospital, Medical Library, Skövde, Sweden; ^8^Region Västra Götaland, Sahlgrenska University Hospital, Medical Library, Gothenburg, Sweden; ^9^School of Public Health and Community Medicine, Institute of Medicine, University of Gothenburg, Gothenburg, Sweden; ^10^Department of Pharmaceutical Outcomes & Policy, College of Pharmacy, University of Florida, Gainesville, FL, United States; ^11^Department of Genetics and Bioinformatics, Division of Health Data and Digitalization, Institute of Public Health, Oslo, Norway

**Keywords:** preterm birth, perinatal morbidity and mortality, progesterone, cerclage, pessary, acetylsalicylic acid, systematic review

In the published article, there was an error. The results of the meta-analyses have marginally changed after exclusion of an article [Cetingoz et al., 2011] (1) that has been retracted and was included in several meta-analyses. We observed a retraction note for this article that was published in Archives of Gynecology and Obstetrics: Cetingoz et al. (2024) (2). The scientific conclusions of our systematic review will not be changed.

A correction has been made to **Abstract**, *Results*, paragraph 2. This sentence previously stated:

“Singleton pregnancies: Progesterone compared with placebo, reduced the risk of preterm birth <37 weeks 26.8% vs. 30.2% (Risk Ratio [RR] 0.82 [95% Confidence Interval [CI] 0.71 to 0.95]) (high certainty of evidence, 14 trials) thereby reducing neonatal mortality and respiratory distress syndrome.”

The corrected sentence appears below:

“Singleton pregnancies: Progesterone compared with placebo, reduced the risk of preterm birth <37 weeks 26.7% vs. 30.3% [risk ratio (RR) 0.82 (95% confidence interval [CI] 0.71–0.96)] (high certainty of evidence, 13 trials) thereby reducing neonatal mortality and respiratory distress syndrome.”

A correction has been made to **Results***, Effect of intervention in singleton pregnancies, Progesterone, Preterm birth across gestational weeks*, paragraph 1.

This sentence previously stated:

“Low risk of bias trials showed an overall effect of progesterone to reduce the risk of preterm birth (Table 2 and Figure 2A). A reduction of any preterm birth was demonstrated for <37 gestational weeks (26.8% vs. 30.2%, RR 0.82; 95% CI 0.71 to 0.95) and <34 gestational weeks (11.7% vs. 15.2%, RR 0.78; 95% CI 0.68 to 0.89) for any administration route (high certainty of evidence).”

The corrected sentence appears below:

“Low risk of bias trials showed an overall effect of progesterone to reduce the risk of preterm birth (Table 2 and Figure 2A). A reduction of any preterm birth was demonstrated for <37 gestational weeks (26.7% vs. 30.3%, RR 0.82; 95% CI 0.71–0.96) and <34 gestational weeks (11.8% vs. 15.4%, RR 0.78; 95% CI 0.67–0.89) for any administration route (high certainty of evidence).”

A correction has been made to **Results**, *Effect of intervention in multifetal pregnancies, Progesterone, Preterm birth across gestational weeks*, paragraph 1. This sentence previously stated:

“Low risk of bias trials demonstrated no effect of progesterone (any administration route) on the risk of any preterm birth <37 gestational weeks (58.3% vs. 57.2%, RR 1.01; 95% CI 0.95 to 1.08) (moderate certainty of evidence), and <34 gestational weeks (22.4% vs. 21.6%, RR 1.02; 95% CI 0.92 to 1.12) (high certainty of evidence), neither on <35, <32, <28 gestational weeks (high certainty of evidence), nor on the risk of spontaneous preterm birth (low to moderate certainty of evidence).”

The corrected sentence appears below:

“Low risk of bias trials demonstrated no effect of progesterone (any administration route) on the risk of any preterm birth <37 gestational weeks (58.5% vs. 56.6%, RR 1.03; 95% CI 0.97–1.08; moderate certainty of evidence), and <34 gestational weeks (22.8% vs. 21.5%, RR 1.02; 95% CI 0.93–1.13; high certainty of evidence), neither on <35, <32, <28 gestational weeks (high certainty of evidence), nor on the risk of spontaneous preterm birth (low to moderate certainty of evidence).”

A correction has been made to the Supplementary material, the supplementary figures have been corrected. The corrected files are listed below:

- Appendix 5.1: SFigure 1, SFigure 5, SFigure 7, and SFigures 28-33.
- Appendix 6.1: SFigure 1, SFigure 5, and SFigure 7.

A correction has been made to the References. A note has been added next to reference 47 that it was not included when updating the Results.

The authors apologize for these errors and state that this does not change the scientific conclusions of the article in any way. The original article has been updated.
