# Supplementary material for: Progesterone, cerclage, pessary, or acetylsalicylic acid for prevention of preterm birth in singleton and multifetal pregnancies – A systematic review and meta-analyses
Source: Front Med (Lausanne). 2023 Feb 28;10:1111315. doi: 10.3389/fmed.2023.1111315 (PMC10015499; doi:10.3389/fmed.2023.1111315)
Supplement: Supplementary file 1 [file Data_Sheet_1.zip › Data Sheet 1_corrected/Appendix 1a and 1b Literature search.docx]

**Progesterone, cerclage, pessary, or acetylsalicylic acid for prevention of preterm birth in singleton and multifetal pregnancies**

**Table of contents**

[**Appendix 1a:** Search strategies for the main study question 2](#_Toc119577308)

**[Appendix 1b:](#_Toc119577309)** [Search strategies for the study question on progesterone and cancer in the woman](#_Toc119577309)

[9](#_Toc119577309)

# **Appendix 1a**

## **Search strategies for the main study question**

**Database:** PubMed

**Date:** 25 Jan 2021

**No. of results:** 47

**Search updated:** 1 Feb 2022, 12 results

| **Search** | **Query** | **Results** |
| --- | --- | --- |
| **#24** | **Search: #16 and #19 Filters: Danish, English, Norwegian, Swedish Sort by: First Author** | **47** |
| #20 | Search: #16 and #19 Sort by: First Author | 47 |
| #19 | Search: #17 or #18 Sort by: First Author | 3,641,306 |
| #18 | Search: review*[tiab] OR quantitative review*[tiab] OR quantitative overview*[tiab] OR quantitative synthes*[tiab] OR pooled analy*[tiab] OR Cochrane[tiab] OR Medline[tiab] OR Pubmed[tiab] OR Medlars[tiab] OR handsearch*[tiab] OR hand search*[tiab] OR meta-regression*[tiab] OR metaregression*[tiab] OR data synthes*[tiab] OR data extraction[tiab] OR data abstraction*[tiab] OR mantel haenszel[tiab] OR peto[tiab] OR der-simonian[tiab] OR dersimonian[tiab] OR fixed effect*[tiab] OR "Cochrane Database Syst Rev" OR "health technology assessment winchester, england"[Journal] OR "Evid Rep Technol Assess (Full Rep)"[Journal] OR "Evid Rep Technol Assess (Summ)"[Journal] OR "Int J Technol Assess Health Care"[Journal] OR "GMS Health Technol Assess"[Journal] OR "Health Technol Assess (Rockv)"[Journal] OR "Health Technol Assess Rep"[Journal] Sort by: First Author | 2,301,833 |
| #17 | Search: Randomized controlled trial[pt] OR Randomized controlled trials as topic[mh] OR Controlled Clinical Trial[Mesh] OR Controlled Clinical Trials as Topic[Mesh] OR Random allocation [mh] OR Double-blind method[mh] OR Single-blind method[mh] OR Control Groups[Mesh] OR Random*[tw] OR "Placebos"[Mesh] OR Placebo[tiab] OR ((singl*[tw] OR doubl*[tw] OR trebl*[tw] OR tripl*[tw]) AND (Mask*[tw] OR blind*[tw] OR dumm*[tw])) Sort by: First Author | 1,552,603 |
| #16 | Search: #14 and #15 Sort by: First Author | 112 |
| #15 | Search: pubmednotmedline[sb] Sort by: First Author | 3,599,407 |
| #14 | Search: #3 and #6 and #13 Sort by: First Author | 1,824 |
| #13 | Search: #7 or #8 or #9 or #10 or #11 or #12 Sort by: First Author | 116,681 |
| #12 | Search: Aspirin*[tw] or Acetylsalicylic*[tw] Sort by: First Author | 72,574 |
| #11 | Search: Shirodkar*[tiab] or McDonald*[tiab] or MacDonald*[tiab] Sort by: First Author | 3,072 |
| #10 | Search: ((cervi*[tiab]) AND (stitch*[tiab] or sutur*[tiab] or ligat*[tiab])) Sort by: First Author | 3,024 |
| #9 | Search: Progestin*[tw] or Progestagen*[tw] or Progestogen*[tw] or gestagen*[tw] or 20 alpha dihydroprogesteron*[tw] or algeston*[tw] or allylestrenol*[tw] or desogestrel*[tw] or dydrogesteron*[tw] or flurogestone acetate*[tw] or gestrinon*[tw] or progestative*[tw] or medroxyprogesteron* acetate*[tw] Sort by: First Author | 30,237 |
| #8 | Search: Progestron*[tw] or hydroxyprogesteron*[tw] or 17-alphahydroxyprogesteron*[tw] or 17-hydroxyprogesteron*[tw ] or 17-OHPC[tw] or 17OHPC[tw] or 17Pc[tw] or Progestin*[tw] or Progestagen*[tw] or Progestogen*[tw] Sort by: First Author | 33,262 |
| #7 | Search: Pessar*[tiab] or Arabin[tiab] Sort by: First Author | 1,825 |
| #6 | Search: #4 or #5 Sort by: First Author | [208,707](https://pubmed.ncbi.nlm.nih.gov/?term=%234+or+%235&sort=fauth&ac=no) |
| #5 | Search: Prematur*[tiab] or pre-matur*[tiab] Sort by: First Author | [153,177](https://pubmed.ncbi.nlm.nih.gov/?term=Prematur%2A%5Btiab%5D+or+pre-matur%2A%5Btiab%5D&sort=fauth&ac=no) |
| #4 | Search: ((preterm*[tiab] or pre-term*[tiab]) AND (birth*[tiab] or deliver*[tiab] or rupture*[tiab] or interruption*[tiab] or labo?r[tiab] or labo?rs[tiab] or labo?ring[tiab] or infant*[tiab] or baby[tiab] or babies[tiab] or child*[tiab] or neonate*[tiab])) Sort by: First Author | [74,945](https://pubmed.ncbi.nlm.nih.gov/?term=%28%28preterm%2A%5Btiab%5D+or+pre-term%2A%5Btiab%5D%29+AND+%28birth%2A%5Btiab%5D+or+deliver%2A%5Btiab%5D+or+rupture%2A%5Btiab%5D+or+interruption%2A%5Btiab%5D+or+labo%3Fr%5Btiab%5D+or+labo%3Frs%5Btiab%5D+or+labo%3Fring%5Btiab%5D+or+infant%2A%5Btiab%5D+or+baby%5Btiab%5D+or+babies%5Btiab%5D+or+child%2A%5Btiab%5D+or+neonate%2A%5Btiab%5D%29%29&sort=fauth&ac=no) |
| #3 | Search: #1 or #2 Sort by: First Author | [2,467,999](https://pubmed.ncbi.nlm.nih.gov/?term=%231+or+%232&sort=fauth&ac=no) |
| #2 | Search: ((Asymptomatic*[tiab] or risk*[tiab] or Twin*[tiab] or Triplet*[tiab] or Quadruplet*[tiab] or Quintuplet*[tiab] or multiple*[tiab] or spontaneous*[tiab]) AND (wom?n*[tiab] or patient*[tiab])) Sort by: First Author | [1,727,007](https://pubmed.ncbi.nlm.nih.gov/?term=%28%28Asymptomatic%2A%5Btiab%5D+or+risk%2A%5Btiab%5D+or+Twin%2A%5Btiab%5D+or+Triplet%2A%5Btiab%5D+or+Quadruplet%2A%5Btiab%5D+or+Quintuplet%2A%5Btiab%5D+or+multiple%2A%5Btiab%5D+or+spontaneous%2A%5Btiab%5D%29+AND+%28wom%3Fn%2A%5Btiab%5D+or+patient%2A%5Btiab%5D%29%29&sort=fauth&ac=no) |
| #1 | Search: Pregnan*[tiab] or Gestation*[tiab] or Trimester*[tiab] or Postpartum*[tiab] or Maternal*[tiab] or singleton*[tiab] Sort by: First Author | [807,579](https://pubmed.ncbi.nlm.nih.gov/?term=Pregnan%2A%5Btiab%5D+or+Gestation%2A%5Btiab%5D+or+Trimester%2A%5Btiab%5D+or+Postpartum%2A%5Btiab%5D+or+Maternal%2A%5Btiab%5D+or+singleton%2A%5Btiab%5D&sort=fauth&ac=no) |

**_________________________________________________________________________________**

**Database: Medline (OVID)** 1946 to January 22, 2021

**Date:** 25 Jan 2021

**No. of results:** 885

**Search updated:** 1 Feb 2022, 97 results

| **#** | **Searches** | **Results** |
| --- | --- | --- |
| 1 | exp Pregnancy/ or exp Pregnancy Trimesters/ or Pregnant Women/ or Postpartum Period/ or exp Multiple Birth Offspring/ | 928506 |
| 2 | (Pregnan* or Gestation* or Trimester* or Postpartum* or Maternal* or singleton*).ab,kf,ti. | 808169 |
| 3 | ((Asymptomatic* or risk* or Twin* or Triplet* or Quadruplet* or Quintuplet* or multiple* or spontaneous*) adj6 (wom?n* or patient*)).ab,kf,ti. | 651689 |
| 4 | 1 or 2 or 3 | 1809082 |
| 5 | exp Obstetric Labor, Premature/ | 27217 |
| 6 | exp Infant, Premature/ | 57056 |
| 7 | ((preterm* or pre-term*) adj6 (birth* or deliver* or rupture* or interruption* or labo?r or labo?rs or labo?ring or infant* or baby or babies or child* or neonate*)).ab,kf,ti. | 73975 |
| 8 | (Prematur* or pre-matur*).ab,kf,ti. | 153931 |
| 9 | 5 or 6 or 7 or 8 | 227593 |
| 10 | Pessaries/ | 1495 |
| 11 | (Pessar* or Arabin).ab,kf,ti. | 1837 |
| 12 | exp Progesterone/ | 70544 |
| 13 | (Progestron* or hydroxyprogesteron* or 17-alphahydroxyprogesteron* or 17-hydroxyprogesteron* or 17-OHPC or 17OHPC or 17Pc or Progestin* or Progestagen* or Progestogen*).mp. | 33319 |
| 14 | exp Progestins/ or Desogestrel/ | 68862 |
| 15 | (Progestin* or Progestagen* or Progestogen* or gestagen* or 20 alpha dihydroprogesteron* or algeston* or allylestrenol* or desogestrel* or dydrogesteron* or flurogestone acetate* or gestrinon* or progestative* or medroxyprogesteron* acetate*).mp. | 34665 |
| 16 | Cerclage, Cervical/ | 900 |
| 17 | (cervi* adj10 (stitch* or sutur* or ligat*)).ab,kf,ti. | 1097 |
| 18 | (Shirodkar* or McDonald* or MacDonald*).ab,kf,ti. | 3069 |
| 19 | exp Aspirin/ | 45504 |
| 20 | (Aspirin* or Acetylsalicylic*).mp. | 72719 |
| 21 | 10 or 11 or 12 or 13 or 14 or 15 or 16 or 17 or 18 or 19 or 20 | 173991 |
| 22 | (Randomized Controlled Trial or Controlled Clinical Trial or Pragmatic Clinical Trial or Equivalence Trial or Clinical Trial, Phase III).pt. | 614165 |
| 23 | exp Randomized Controlled Trial/ or exp Randomized Controlled Trials as Topic/ or Controlled Clinical Trial/ or exp Controlled Clinical Trials as Topic/ or Random Allocation/ or Double-Blind Method/ or Single-Blind Method/ or Placebos/ or Control Groups/ | 864509 |
| 24 | (random* or sham or placebo*).ti,ab,hw,kf. | 1564177 |
| 25 | ((singl* or doubl*) adj (blind* or dumm* or mask*)).ti,ab,hw,kf. | 243463 |
| 26 | ((tripl* or trebl*) adj (blind* or dumm* or mask*)).ti,ab,hw,kf. | 1125 |
| 27 | (control* adj3 (study or studies or trial* or group*)).ti,ab,kf. | 1032733 |
| 28 | (Nonrandom* or non random* or non-random* or quasi-random* or quasirandom*).ti,ab,hw,kf. | 46307 |
| 29 | allocated.ti,ab,hw. | 69497 |
| 30 | ((open label or open-label) adj5 (study or studies or trial*)).ti,ab,hw,kf. | 36706 |
| 31 | ((equivalence or superiority or non-inferiority or noninferiority) adj3 (study or studies or trial*)).ti,ab,hw,kf. | 9160 |
| 32 | (pragmatic study or pragmatic studies).ti,ab,hw,kf. | 441 |
| 33 | ((pragmatic or practical) adj3 trial*).ti,ab,hw,kf. | 5730 |
| 34 | ((quasiexperimental or quasi-experimental) adj3 (study or studies or trial*)).ti,ab,hw,kf. | 8661 |
| 35 | (phase adj3 (III or "3") adj3 (study or studies or trial*)).ti,hw,kf. | 29647 |
| 36 | or/22-35 | 2246945 |
| 37 | Meta-Analysis.pt. | 125413 |
| 38 | Meta-analysis/ or Systematic review/ or exp Meta-analysis as topic/ or Systematic Reviews as Topic/ or exp Technology assessment, biomedical/ | 239513 |
| 39 | ((systematic* adj3 (review* or overview*)) or (methodologic* adj3 (review* or overview*))).ab,kf,ti. | 215751 |
| 40 | ((quantitative adj3 (review* or overview* or synthes*)) or (research adj3 (integrati* or overview*))).ab,kf,ti. | 11828 |
| 41 | ((integrative adj3 (review* or overview*)) or (collaborative adj3 (review* or overview*)) or (pool* adj3 analy*)).ab,kf,ti. | 29332 |
| 42 | (data synthes* or data extraction* or data abstraction*).ab,kf,ti. | 29754 |
| 43 | (handsearch* or hand search*).ab,kf,ti. | 9682 |
| 44 | (mantel haenszel or peto or der simonian or dersimonian or fixed effect* or latin square*).ab,kf,ti. | 28201 |
| 45 | (met analy* or metanaly* or technology assessment* or HTA or HTAs or technology overview* or technology appraisal*).ab,kf,ti. | 9959 |
| 46 | (meta regression* or metaregression*).ab,kf,ti. | 10004 |
| 47 | (meta-analy* or metaanaly* or systematic review* or biomedical technology assessment* or bio-medical technology assessment*).mp,hw. | 338259 |
| 48 | (medline or cochrane or pubmed or medlars or embase or cinahl).ab,hw,ti. | 244677 |
| 49 | (cochrane or (health adj2 technology assessment) or evidence report).jw. | 20032 |
| 50 | (comparative adj3 (efficacy or effectiveness)).ab,kf,ti. | 14298 |
| 51 | (outcomes research or relative effectiveness).ab,kf,ti. | 9835 |
| 52 | ((indirect or indirect treatment or mixed-treatment) adj comparison*).ab,kf,ti. | 2295 |
| 53 | or/37-52 | 511050 |
| 54 | 36 or 53 | 2567627 |
| 55 | 4 and 9 and 21 and 54 | 1037 |
| 56 | (animals not (animals and humans)).sh. | 4746268 |
| 57 | 55 not 56 | 963 |
| 58 | (comment or editorial or letter).pt. | 1928598 |
| 59 | 57 not 58 | 929 |
| 60 | limit 59 to (danish or english or norwegian or swedish) | 885 |
| 61 | limit 60 to yr="1960 -Current" | 885 |

**________________________________________________________________________________**

**Database: Embase (OVID)** 1974 to 2021 January 21

**Date:** 25 Jan 2021

**No. of results:** 1,668

**Search updated:** 1 Feb 2022, 190 results

| **#** | **Searches** | **Results** |
| --- | --- | --- |
| 1 | exp pregnancy/ or exp high risk pregnancy/ or pregnant woman/ or puerperium/ or exp multiple birth offspring/ | 772481 |
| 2 | (Pregnan* or Gestation* or Trimester* or Postpartum* or Maternal* or singleton*).ab,kw,ti. | 986629 |
| 3 | ((Asymptomatic* or risk* or Twin* or Triplet* or Quadruplet* or Quintuplet* or multiple* or spontaneous*) adj6 (wom?n* or patient*)).ab,kw,ti. | 1040625 |
| 4 | 1 or 2 or 3 | 2180162 |
| 5 | exp premature labor/ | 47074 |
| 6 | exp prematurity/ | 106098 |
| 7 | ((preterm* or pre-term*) adj6 (birth* or deliver* or rupture* or interruption* or labo?r or labo?rs or labo?ring or infant* or baby or babies or child* or neonate*)).ab,kw,ti. | 103937 |
| 8 | (Prematur* or pre-matur*).ab,kw,ti. | 203169 |
| 9 | 5 or 6 or 7 or 8 | 309477 |
| 10 | exp vagina pessary/ | 2843 |
| 11 | (Pessar* or Arabin).ab,kw,ti. | 2761 |
| 12 | progesterone/ or hydroxyprogesterone/ or hydroxyprogesterone caproate/ | 95237 |
| 13 | (Progestron* or hydroxyprogesteron* or 17-alphahydroxyprogesteron* or 17-hydroxyprogesteron* or 17-OHPC or 17OHPC or 17Pc or Progestin* or Progestagen* or Progestogen*).mp. | 33096 |
| 14 | exp gestagen/ or 20-alpha-dihydroprogesterone/ or desogestrel/ or medroxyprogesterone acetate/ | 167659 |
| 15 | (Progestin* or Progestagen* or Progestogen* or gestagen* or 20 alpha dihydroprogesteron* or algeston* or allylestrenol* or desogestrel* or dydrogesteron* or flurogestone acetate* or gestrinon* or progestative* or medroxyprogesteron* acetate*).mp. | 54458 |
| 16 | exp cerclage/ | 2158 |
| 17 | Cerclage*.ab,kw,ti. | 4696 |
| 18 | (cervi* adj10 (stitch* or sutur* or ligat*)).ab,ti,kw. | 1562 |
| 19 | (Shirodkar* or McDonald* or MacDonald*).ab,ti,kw. | 5525 |
| 20 | exp acetylsalicylic acid/ | 214928 |
| 21 | (Aspirin* or Acetylsalicylic*).mp. | 227712 |
| 22 | 10 or 11 or 12 or 13 or 14 or 15 or 16 or 17 or 18 or 19 or 20 or 21 | 412362 |
| 23 | exp randomized controlled trial/ or "randomized controlled trial (topic)"/ or controlled clinical trial/ or "controlled clinical trial (topic)"/ or exp randomization/ or double blind procedure/ or single blind procedure/ or placebo/ or control group/ | 1423893 |
| 24 | (random* or sham or placebo*).ti,ab,hw,kw. | 2145687 |
| 25 | ((singl* or doubl*) adj (blind* or dumm* or mask*)).ti,ab,hw,kw. | 316802 |
| 26 | ((tripl* or trebl*) adj (blind* or dumm* or mask*)).ti,ab,hw,kw. | 1484 |
| 27 | (control* adj3 (study or studies or trial* or group*)).ti,ab,kw. | 1438186 |
| 28 | (Nonrandom* or non random* or non-random* or quasi-random* or quasirandom*).ti,ab,hw,kw. | 58043 |
| 29 | allocated.ti,ab,hw. | 89619 |
| 30 | ((open label or open-label) adj5 (study or studies or trial*)).ti,ab,hw,kw. | 67366 |
| 31 | ((equivalence or superiority or non-inferiority or noninferiority) adj3 (study or studies or trial*)).ti,ab,hw,kw. | 13374 |
| 32 | (pragmatic study or pragmatic studies).ti,ab,hw,kw. | 648 |
| 33 | ((pragmatic or practical) adj3 trial*).ti,ab,hw,kw. | 6017 |
| 34 | ((quasiexperimental or quasi-experimental) adj3 (study or studies or trial*)).ti,ab,hw,kw. | 13674 |
| 35 | (phase adj3 (III or "3") adj3 (study or studies or trial*)).ti,hw,kw. | 96974 |
| 36 | or/23-35 | 3193950 |
| 37 | exp meta analysis/ or "systematic review"/ or "meta analysis (topic)"/ or "systematic review (topic)"/ or biomedical technology assessment/ | 444709 |
| 38 | ((systematic* adj3 (review* or overview*)) or (methodologic* adj3 (review* or overview*))).ab,kw,ti. | 267467 |
| 39 | ((quantitative adj3 (review* or overview* or synthes*)) or (research adj3 (integrati* or overview*))).ab,kw,ti. | 14006 |
| 40 | ((integrative adj3 (review* or overview*)) or (collaborative adj3 (review* or overview*)) or (pool* adj3 analy*)).ab,kw,ti. | 41581 |
| 41 | (data synthes* or data extraction* or data abstraction*).ab,kw,ti. | 36563 |
| 42 | (handsearch* or hand search*).ab,kw,ti. | 11720 |
| 43 | (mantel haenszel or peto or der simonian or dersimonian or fixed effect* or latin square*).ab,kw,ti. | 37006 |
| 44 | (met analy* or metanaly* or technology assessment* or HTA or HTAs or technology overview* or technology appraisal*).ab,kw,ti. | 16117 |
| 45 | (meta regression* or metaregression*).ab,kw,ti. | 12396 |
| 46 | (meta-analy* or metaanaly* or systematic review* or biomedical technology assessment* or bio-medical technology assessment*).mp,hw. | 535571 |
| 47 | (medline or cochrane or pubmed or medlars or embase or cinahl).ab,hw,ti. | 320867 |
| 48 | (cochrane or (health adj2 technology assessment) or evidence report).jx. | 27490 |
| 49 | (comparative adj3 (efficacy or effectiveness)).ab,kw,ti. | 20811 |
| 50 | (outcomes research or relative effectiveness).ab,kw,ti. | 14094 |
| 51 | ((indirect or indirect treatment or mixed-treatment) adj comparison*).ab,kw,ti. | 4321 |
| 52 | or/37-51 | 741825 |
| 53 | 36 or 52 | 3642344 |
| 54 | 4 and 9 and 22 and 53 | 2534 |
| 55 | limit 54 to (article or article in press or conference paper or "review") | 1795 |
| 56 | limit 55 to (danish or english or norwegian or swedish) | 1694 |
| 57 | (animal not (animal and human)).sh. | 1100804 |
| **58** | **56 not 57** | **1668** |

**________________________________________________________________________________**

**Database:** The Cochrane Library
**Date:** 25 Jan 2021
**No of results:** 664

*Cochrane reviews: 31
Cochrane protocols: 1
Trials: 630
Clinical answers: 2*

**Search updated:** 1 Feb 2022, 65 results

*Cochrane reviews: 2
Trials: 63*

| **ID** | **Search** | **Hits** |
| --- | --- | --- |
| #1 | MeSH descriptor: [Pregnancy] explode all trees | 22051 |
| #2 | MeSH descriptor: [Pregnancy Trimesters] explode all trees | 1735 |
| #3 | MeSH descriptor: [Pregnant Women] this term only | 274 |
| #4 | MeSH descriptor: [Postpartum Period] this term only | 1188 |
| #5 | MeSH descriptor: [Multiple Birth Offspring] explode all trees | 191 |
| #6 | (Pregnan* or Gestation* or Trimester* or Postpartum* or Maternal* or singleton*):ti,ab,kw (Word variations have been searched) | 84049 |
| #7 | ((Asymptomatic* or risk* or Twin* or Triplet* or Quadruplet* or Quintuplet* or multiple* or spontaneous*) NEAR/6 (wom?n* or patient*)):ti,ab,kw (Word variations have been searched) | 85626 |
| #8 | {OR #1-#7} | 162396 |
| #9 | MeSH descriptor: [Obstetric Labor, Premature] explode all trees | 2175 |
| #10 | MeSH descriptor: [Infant, Premature] explode all trees | 3785 |
| #11 | ((preterm* or pre-term*) NEAR/6 (birth* or deliver* or rupture* or interruption* or labo?r or labo?rs or labo?ring or infant* or baby or babies or child* or neonate*).):ti,ab,kw (Word variations have been searched) | 13242 |
| #12 | (Prematur* or pre-matur*):ti,ab,kw (Word variations have been searched) | 23704 |
| #13 | {OR #9-#12} | 27970 |
| #14 | MeSH descriptor: [Pessaries] this term only | 183 |
| #15 | (Pessar* or Arabin):ti,ab,kw (Word variations have been searched) | 739 |
| #16 | MeSH descriptor: [Progesterone] explode all trees | 3162 |
| #17 | (Progestron* or hydroxyprogesteron* or "17 alphahydroxyprogesteron*" or "17 hydroxyprogesteron*" or "17 OHPC" or 17OHPC or 17Pc or Progestin* or Progestagen* or Progestogen*):ti,ab,kw (Word variations have been searched) | 3456 |
| #18 | MeSH descriptor: [Progestins] explode all trees | 537 |
| #19 | MeSH descriptor: [Desogestrel] this term only | 452 |
| #20 | (Progestin* or Progestagen* or Progestogen* or gestagen* or "20 alpha dihydroprogesteron*" or algeston* or allylestrenol* or desogestrel* or dydrogesteron* or "flurogestone acetate*" or gestrinon* or progestative* or "medroxyprogesteron* acetate*"):ti,ab,kw (Word variations have been searched) | 5465 |
| #21 | MeSH descriptor: [Cerclage, Cervical] this term only | 56 |
| #22 | ((cervi*) NEAR/10 (stitch* or sutur* or ligat*)):ti,ab,kw (Word variations have been searched) | 125 |
| #23 | (Shirodkar* or McDonald* or MacDonald*):ti,ab,kw (Word variations have been searched) | 681 |
| #24 | MeSH descriptor: [Aspirin] explode all trees | 5935 |
| #25 | (Aspirin* or Acetylsalicylic*):ti,ab,kw (Word variations have been searched) | 16547 |
| #26 | {OR #14-#25} | 25414 |
| #27 | #8 AND #13 AND #26 | 913 |
| #28 | (CINAHL):an (Word variations have been searched) | 18849 |
| #29 | #27 NOT #28 | 908 |
| #30 | (clinicaltrials or trialsearch):so | 352906 |
| #31 | #29 NOT #30 | 664 |

**________________________________________________________________________________**

The web-sites of **SBU** and **Folkehelseinstituttet** were visited

25 Jan 2021

Nothing relevant to the question at issue was found

**Reference lists**

A comprehensive review of reference lists brought 8 new records

# **Appendix 1b**

**Search strategies for the study question on progesterone and cancer in the woman**

**Database:** PubMed

**Date:** 30 Nov 2021
**No. of results: 7**

| **Search** | **Query** | **Results** |
| --- | --- | --- |
| #13 | Search: **#11 AND #12** Sort by: **First Author** | [7](https://pubmed.ncbi.nlm.nih.gov/?term=%2311+AND+%2312&sort=fauth&sort_order=asc&size=200&ac=no) |
| #12 | Search: **(pubmednotmedline[sb] OR inprocess[sb] OR publisher[sb])** Sort by: **First Author** | [4,659,948](https://pubmed.ncbi.nlm.nih.gov/?term=%28pubmednotmedline%5Bsb%5D+OR+inprocess%5Bsb%5D+OR+publisher%5Bsb%5D%29&sort=fauth&sort_order=asc&size=200&ac=no) |
| #11 | Search: **#3 AND #6 AND #9 AND #10** Sort by: **First Author** | [35](https://pubmed.ncbi.nlm.nih.gov/?term=%233+AND+%236+AND+%239+AND+%2310&sort=fauth&sort_order=asc&size=200&ac=no) |
| #10 | Search: **cancer*[tiab] or tumo?r*[tiab] or malignan*[tiab] or neoplas*[tiab] or carcino*[tiab] or oncolog*[tiab] or oncogen*[tiab]** Sort by: **First Author** | [3,100,087](https://pubmed.ncbi.nlm.nih.gov/?term=cancer%2A%5Btiab%5D+or+tumo%3Fr%2A%5Btiab%5D+or+malignan%2A%5Btiab%5D+or+neoplas%2A%5Btiab%5D+or+carcino%2A%5Btiab%5D+or+oncolog%2A%5Btiab%5D+or+oncogen%2A%5Btiab%5D&sort=fauth&sort_order=asc&size=200&ac=no) |
| #9 | Search: **#7 OR #8** Sort by: **First Author** | [37,190](https://pubmed.ncbi.nlm.nih.gov/?term=%237+OR+%238&sort=fauth&sort_order=asc&size=200&ac=no) |
| #8 | Search: **Progestin*[tw] or Progestagen*[tw] or Progestogen*[tw] or gestagen*[tw] or 20 alpha dihydroprogesteron*[tw] or algeston*[tw] or allylestrenol*[tw] or desogestrel*[tw] or dydrogesteron*[tw] or flurogestone acetate*[tw] or gestrinon*[tw] or progestative*[tw] or medroxyprogesteron* acetate*[tw]** Sort by: **First Author** | [30,789](https://pubmed.ncbi.nlm.nih.gov/?term=Progestin%2A%5Btw%5D+or+Progestagen%2A%5Btw%5D+or+Progestogen%2A%5Btw%5D+or+gestagen%2A%5Btw%5D+or+20+alpha+dihydroprogesteron%2A%5Btw%5D+or+algeston%2A%5Btw%5D+or+allylestrenol%2A%5Btw%5D+or+desogestrel%2A%5Btw%5D+or+dydrogesteron%2A%5Btw%5D+or+flurogestone+acetate%2A%5Btw%5D+or+gestrinon%2A%5Btw%5D+or+progestative%2A%5Btw%5D+or+medroxyprogesteron%2A+acetate%2A%5Btw%5D&sort=fauth&sort_order=asc&size=200&ac=no) |
| #7 | Search: **Progestron*[tw] or hydroxyprogesteron*[tw] or 17-alphahydroxyprogesteron*[tw] or 17-hydroxyprogesteron*[tw ] or 17-OHPC[tw] or 17OHPC[tw] or 17Pc[tw] or Progestin*[tw] or Progestagen*[tw] or Progestogen*[tw]** Sort by: **First Author** | [33,833](https://pubmed.ncbi.nlm.nih.gov/?term=Progestron%2A%5Btw%5D+or+hydroxyprogesteron%2A%5Btw%5D+or+17-alphahydroxyprogesteron%2A%5Btw%5D+or+17-hydroxyprogesteron%2A%5Btw+%5D+or+17-OHPC%5Btw%5D+or+17OHPC%5Btw%5D+or+17Pc%5Btw%5D+or+Progestin%2A%5Btw%5D+or+Progestagen%2A%5Btw%5D+or+Progestogen%2A%5Btw%5D&sort=fauth&sort_order=asc&size=200&ac=no) |
| #6 | Search: **#4 OR #5** Sort by: **First Author** | [220,117](https://pubmed.ncbi.nlm.nih.gov/?term=%234+OR+%235&sort=fauth&sort_order=asc&size=200&ac=no) |
| #5 | Search: **Prematur*[tiab] or pre-matur*[tiab]** Sort by: **First Author** | [160,450](https://pubmed.ncbi.nlm.nih.gov/?term=Prematur%2A%5Btiab%5D+or+pre-matur%2A%5Btiab%5D&sort=fauth&sort_order=asc&size=200&ac=no) |
| #4 | Search: **((preterm*[tiab] or pre-term*[tiab]) AND (birth*[tiab] or deliver*[tiab] or rupture*[tiab] or interruption*[tiab] or labo?r[tiab] or labo?rs[tiab] or labo?ring[tiab] or infant*[tiab] or baby[tiab] or babies[tiab] or child*[tiab] or neonate*[tiab]))** Sort by: **First Author** | [80,585](https://pubmed.ncbi.nlm.nih.gov/?term=%28%28preterm%2A%5Btiab%5D+or+pre-term%2A%5Btiab%5D%29+AND+%28birth%2A%5Btiab%5D+or+deliver%2A%5Btiab%5D+or+rupture%2A%5Btiab%5D+or+interruption%2A%5Btiab%5D+or+labo%3Fr%5Btiab%5D+or+labo%3Frs%5Btiab%5D+or+labo%3Fring%5Btiab%5D+or+infant%2A%5Btiab%5D+or+baby%5Btiab%5D+or+babies%5Btiab%5D+or+child%2A%5Btiab%5D+or+neonate%2A%5Btiab%5D%29%29&sort=fauth&sort_order=asc&size=200&ac=no) |
| #3 | Search: **#1 OR #2** Sort by: **First Author** | [2,636,683](https://pubmed.ncbi.nlm.nih.gov/?term=%231+OR+%232&sort=fauth&sort_order=asc&size=200&ac=no) |
| #2 | Search: **((Asymptomatic*[tiab] or risk*[tiab] or Twin*[tiab] or Triplet*[tiab] or Quadruplet*[tiab] or Quintuplet*[tiab] or multiple*[tiab] or spontaneous*[tiab]) AND (wom?n*[tiab] or patient*[tiab]))** Sort by: **First Author** | [1,862,176](https://pubmed.ncbi.nlm.nih.gov/?term=%28%28Asymptomatic%2A%5Btiab%5D+or+risk%2A%5Btiab%5D+or+Twin%2A%5Btiab%5D+or+Triplet%2A%5Btiab%5D+or+Quadruplet%2A%5Btiab%5D+or+Quintuplet%2A%5Btiab%5D+or+multiple%2A%5Btiab%5D+or+spontaneous%2A%5Btiab%5D%29+AND+%28wom%3Fn%2A%5Btiab%5D+or+patient%2A%5Btiab%5D%29%29+&sort=fauth&size=200) |
| #1 | Search: **Pregnan*[tiab] or Gestation*[tiab] or Trimester*[tiab] or Postpartum*[tiab] or Maternal*[tiab] or singleton*[tiab]** Sort by: **First Author** | [845,363](https://pubmed.ncbi.nlm.nih.gov/?term=Pregnan%2A%5Btiab%5D+or+Gestation%2A%5Btiab%5D+or+Trimester%2A%5Btiab%5D+or+Postpartum%2A%5Btiab%5D+or+Maternal%2A%5Btiab%5D+or+singleton%2A%5Btiab%5D+&sort=fauth&sort_order=asc&size=200) |

**Database:** Ovid MEDLINE(R) ALL

**Date:** 30 Nov 2021
**No. of results:** 100

| **#** | **Searches** | **Results** |
| --- | --- | --- |
| 1 | exp Pregnancy/ or exp Pregnancy Trimesters/ or Pregnant Women/ or Postpartum Period/ or exp Multiple Birth Offspring/ | 968497 |
| 2 | (Pregnan* or Gestation* or Trimester* or Postpartum* or Maternal* or singleton*).ab,kf,ti. | 842596 |
| 3 | ((Asymptomatic* or risk* or Twin* or Triplet* or Quadruplet* or Quintuplet* or multiple* or spontaneous*) adj6 (wom?n* or patient*)).ab,kf,ti. | 694817 |
| 4 | 1 or 2 or 3 | 1892283 |
| 5 | exp Obstetric Labor, Premature/ | 29699 |
| 6 | exp Infant, Premature/ | 60390 |
| 7 | ((preterm* or pre-term*) adj6 (birth* or deliver* or rupture* or interruption* or labo?r or labo?rs or labo?ring or infant* or baby or babies or child* or neonate*)).ab,kf,ti. | 78930 |
| 8 | (Prematur* or pre-matur*).ab,kf,ti. | 160595 |
| 9 | 5 or 6 or 7 or 8 | 238395 |
| 10 | exp Progesterone/ | 71649 |
| 11 | (Progestron* or hydroxyprogesteron* or 17-alphahydroxyprogesteron* or 17-hydroxyprogesteron* or 17-OHPC or 17OHPC or 17Pc or Progestin* or Progestagen* or Progestogen*).mp. | 33870 |
| 12 | exp Progestins/ or Desogestrel/ | 70056 |
| 13 | (Progestin* or Progestagen* or Progestogen* or gestagen* or 20 alpha dihydroprogesteron* or algeston* or allylestrenol* or desogestrel* or dydrogesteron* or flurogestone acetate* or gestrinon* or progestative* or medroxyprogesteron* acetate*).mp. | 35257 |
| 14 | 10 or 11 or 12 or 13 | 96144 |
| 15 | exp Neoplasms/ | 3579896 |
| 16 | (cancer* or tumo?r* or malignan* or neoplas* or carcino* or oncolog* or oncogen*).ab,kf,ti. | 3766887 |
| 17 | 15 or 16 | 4746186 |
| 18 | 4 and 9 and 14 and 17 | 115 |
| 19 | (animals not (animals and humans)).sh. | 4889136 |
| 20 | 18 not 19 | 108 |
| 21 | (comment or editorial or letter).pt. | 2016291 |
| 22 | 20 not 21 | 107 |
| 23 | limit 22 to (yr="1960 -Current" and (danish or english or norwegian or swedish)) | 100 |

**Database: Embase (OVID)**1974 to 2021 November 29

**Date: 30** Nov 2021
**No. of results:** 301

| **#** | **Searches** | **Results** |
| --- | --- | --- |
| 1 | exp pregnancy/ or exp high risk pregnancy/ or pregnant woman/ or puerperium/ or exp multiple birth offspring/ | 802167 |
| 2 | (Pregnan* or Gestation* or Trimester* or Postpartum* or Maternal* or singleton*).ab,kf,ti. | 1033178 |
| 3 | ((Asymptomatic* or risk* or Twin* or Triplet* or Quadruplet* or Quintuplet* or multiple* or spontaneous*) adj6 (wom?n* or patient*)).ab,kf,ti. | 1116274 |
| 4 | 1 or 2 or 3 | 2301027 |
| 5 | exp premature labor/ | 49984 |
| 6 | exp prematurity/ | 112093 |
| 7 | ((preterm* or pre-term*) adj6 (birth* or deliver* or rupture* or interruption* or labo?r or labo?rs or labo?ring or infant* or baby or babies or child* or neonate*)).ab,kf,ti. | 110735 |
| 8 | (Prematur* or pre-matur*).ab,kf,ti. | 212680 |
| 9 | 5 or 6 or 7 or 8 | 325869 |
| 10 | progesterone/ or hydroxyprogesterone/ or hydroxyprogesterone caproate/ | 97987 |
| 11 | (Progestron* or hydroxyprogesteron* or 17-alphahydroxyprogesteron* or 17-hydroxyprogesteron* or 17-OHPC or 17OHPC or 17Pc or Progestin* or Progestagen* or Progestogen*).mp. | 33836 |
| 12 | exp gestagen/ or 20-alpha-dihydroprogesterone/ or desogestrel/ or medroxyprogesterone acetate/ | 172010 |
| 13 | (Progestin* or Progestagen* or Progestogen* or gestagen* or 20 alpha dihydroprogesteron* or algeston* or allylestrenol* or desogestrel* or dydrogesteron* or flurogestone acetate* or gestrinon* or progestative* or medroxyprogesteron* acetate*).mp. | 55647 |
| 14 | 10 or 11 or 12 or 13 | 177682 |
| 15 | exp neoplasm/ | 4857623 |
| 16 | (cancer* or tumo?r* or malignan* or neoplas* or carcino* or oncolog* or oncogen*).ab,kf,ti. | 4949646 |
| 17 | cancer risk/ | 185312 |
| 18 | 15 or 16 or 17 | 6081329 |
| 19 | 4 and 9 and 14 and 18 | 435 |
| 20 | (animal not (animal and human)).sh. | 1125235 |
| 21 | 19 not 20 | 435 |
| 22 | limit 21 to (article or article in press or conference paper or note or "review") | 327 |
| **23** | **limit 22 to ((danish or english or norwegian or swedish) and yr="1960 -Current")** | **301** |

___________________________________________________________________________

**Database:** The Cochrane Library
**Date:** 30 Nov 2021
**No of results:** 18

*Cochrane reviews: 3
Trials: 15*

| **ID** | **Search** | **Hits** |
| --- | --- | --- |
| #1 | MeSH descriptor: [Pregnancy] explode all trees | 23560 |
| #2 | MeSH descriptor: [Pregnancy Trimesters] explode all trees | 1813 |
| #3 | MeSH descriptor: [Pregnant Women] this term only | 395 |
| #4 | MeSH descriptor: [Postpartum Period] this term only | 1297 |
| #5 | MeSH descriptor: [Multiple Birth Offspring] explode all trees | 200 |
| #6 | (Pregnan* or Gestation* or Trimester* or Postpartum* or Maternal* or singleton*):ti,ab,kw (Word variations have been searched) | 90285 |
| #7 | ((Asymptomatic* or risk* or Twin* or Triplet* or Quadruplet* or Quintuplet* or multiple* or spontaneous*) NEAR/6 (wom?n* or patient*)):ti,ab,kw (Word variations have been searched) | 92752 |
| #8 | {OR #1-#7} | 175112 |
| #9 | MeSH descriptor: [Obstetric Labor, Premature] explode all trees | 2323 |
| #10 | MeSH descriptor: [Infant, Premature] explode all trees | 4047 |
| #11 | ((preterm* or pre-term*) NEAR/6 (birth* or deliver* or rupture* or interruption* or labo?r or labo?rs or labo?ring or infant* or baby or babies or child* or neonate*).):ti,ab,kw (Word variations have been searched) | 14130 |
| #12 | (Prematur* or pre-matur*):ti,ab,kw (Word variations have been searched) | 25286 |
| #13 | {OR #9-#12} | 29768 |
| #14 | MeSH descriptor: [Progesterone] explode all trees | 3269 |
| #15 | (Progestron* or hydroxyprogesteron* or "17 alphahydroxyprogesteron*" or "17 hydroxyprogesteron*" or "17 OHPC" or 17OHPC or 17Pc or Progestin* or Progestagen* or Progestogen*):ti,ab,kw (Word variations have been searched) | 3639 |
| #16 | MeSH descriptor: [Progestins] explode all trees | 570 |
| #17 | MeSH descriptor: [Desogestrel] this term only | 477 |
| #18 | (Progestin* or Progestagen* or Progestogen* or gestagen* or "20 alpha dihydroprogesteron*" or algeston* or allylestrenol* or desogestrel* or dydrogesteron* or "flurogestone acetate*" or gestrinon* or progestative* or "medroxyprogesteron* acetate*"):ti,ab,kw (Word variations have been searched) | 5720 |
| #19 | {OR #14-#18} | 7760 |
| #20 | MeSH descriptor: [Neoplasms] explode all trees | 84994 |
| #21 | (cancer* or tumo?r* or malignan* or neoplas* or carcino* or oncolog* or oncogen*):ti,ab,kw (Word variations have been searched) | 239209 |
| #22 | #20 or #21 | 248390 |
| #23 | #8 AND #13 AND #19 AND #22 | 26 |
| #24 | (CINAHL):an (Word variations have been searched) | 20362 |
| #25 | #23 NOT #24 | 26 |
| #26 | (clinicaltrials or trialsearch):so | 382098 |
| **#27** | **#25 NOT #26** | **18** |

___________________________________________________________________________

The web-sites of **SBU** and **Folkehelseinstituttet** were visited

30 Nov 2021

Nothing relevant to the question at issue was found

**Reference lists**

A comprehensive review of reference lists brought 0 new records
