# Supplementary material for: Progesterone, cerclage, pessary, or acetylsalicylic acid for prevention of preterm birth in singleton and multifetal pregnancies – A systematic review and meta-analyses
Source: Front Med (Lausanne). 2023 Feb 28;10:1111315. doi: 10.3389/fmed.2023.1111315 (PMC10015499; doi:10.3389/fmed.2023.1111315)
Supplement: Supplementary file 1 [file Data_Sheet_1.zip › Data Sheet 1_corrected/Appendix 2 Included publications with characteristics.docx]

**Progesterone, cerclage, pessary, or acetylsalicylic acid for prevention of preterm birth in singleton and multifetal pregnancies**

**Table of contents**

[**Progesterone** 2](#_Toc119581390)

[**Cerclage** 20](#_Toc119581391)

[**Pessary** 27](#_Toc119581392)

[**Acetylsalicylic acid** 34](#_Toc119581393)

[**Other combinations of treatment** 34](#_Toc119581394)

| Progesterone | | | | | | | | | | | | |
| --- | --- | --- | --- | --- | --- | --- | --- | --- | --- | --- | --- | --- |
| Aboulghar  2012  Egypt  (single center) | ISRCTN  69810120  Funding NR | August 2008 to March 2010 | Singletons 215/306  (70.3%)  Twins (DC)  91/306  (29.7%) | ART | NR | GA between 18-24 w | I: 400 mg vag prog/d (Prontogest)  C: Placebo  Randomized 1:1  Until 36+6 | None | 410 invited,  313 women Randomized  I: 161  C: 152 | I: 30.2 (4.6)  C: 31.4 (4) | I: 161  (112 singletons, 49 sets of twins)  C: 145  (103 singletons, 42 set of twins) | PTB <37 w (PO)  PTB <34 w (PO)  LBW, VLBW  PNM, NNM  NICU admission |
| Aflatoonian  2013  Iran  (single center) | IRCT  201210161132  Funding NR | October 2010 to October 2011 | Singleton | ART | NR | GA between 16 w | I: 250 mg  17-OHPC im/w (Femolife)  C: Placebo  Randomized 1:1  Until 36+6 | None | 106 invited,  99 women Randomized  I: 52  C: 47 | I: 30.3  (4.5)  C: 29.1 (4.9) | I: 52  C: 47 | PTB, sPTB <37 w  PTB <34 w  LBW  PNM  NICU admission  Maternal  morbidity |
| Ali 2020  Egypt  (single center) | NCT  02846909  Funding NR | April 2017 to March 2019 | Singleton | Indication for cerclage:  previous  second trimester loss,  sPTD (<34 w) or short cervix  (<25 mm) | I: 23.8  (5.3)  C: 23.9  (5.6) | GA between 12-14 w | I: 400 mg prog vag (pessary)/d  (Prontogest)  C: Placebo  Randomized 1:1  Until 36+6 | Cerclage | 250 invited,  242 women Randomized  I: 121  C: 121 | I: 28.5  (4.7)  C: 29.1 (4.4) | I: 121  C: 121  Lost to follow up  I: 6  C: 9 | PTB <37 w  PTB <34 w  GA at delivery  PNM  NICU admission |
| Ashoush 2017  Egypt  (single center) | NCT  2571296  Funding NR | June 2015 to December 2016 | Singleton | Previous sPTB <37 weeks | I: 25.7  (8.3  C: 23.9  (9.7) | GA between 14-18 w | I: 100 mg prog oral/d  (Utrocare)  C: Placebo  Randomized 1:1  Until 36+6 | TVS CL- screening and cerclage for short cervix | 326 invited,  212 women Randomized  I: 106  C: 106 | I: 29.2  (4.5)  C: 29.5 (3.5) | I: 96  C: 91 | PTB <37 w  GA at delivery  LBW  PNM, NNM  Neonatal and  maternal  morbidity  NICU admission |
| Awwad 2015  Libanon  (single center) | PROGES-  TWIN  NCT  00141908  Funding NR | September 2006 to December 2011 | Twins | ART 75%  MC 17% | NR | GA between 16-20 w | I: 250mg  17-OHPC im/w (Proloton Depot)  C: Placebo (castor oil)  Until 36+6 | None | 344 invited,  293 women Randomized 2:1  I: 197  C: 96 | I: 30.5  (5.6)  C: 30.7 (5.0) | I: 194  C: 94 | PTB <37 w (PO)  PTB <32 w  PTB <28 w  GA at delivery  LBW, VLBW  PNM, NNM  Neonatal and maternal morbidity  NICU admission |
| Azargoon 2016  Iran  (single center) | IRCT  201012273386N2.  Funding  NR | November 2010 to April 2012 | Singleton | Previous PTB <37 w, uterine anomaly, | NR | GA between 16-22 w | I: 400 mg prog vag supp/d  (Aboryhan)  C: Placebo  Randomized 1:1  Until 36+6 | Cerclage if uterine anomaly, previous PTB or TVS CL < 28mm | 106 invited,  103 women Randomized  I: 51  C: 52 | I: 25.4  (4.8)  C: 24.6 (4.9) | I: 50  C: 50 | PTB <37 w (PO)  PTB <34 w  GA at delivery  LBW, VLBW  NNM  Neonatal  morbidity  NICU admission |
| Blackwell  2020  USA  (9 countries, 93 centers. USA (41), outside USA (52) | PRO-LONG  NCT  01004029  Funding reported | November 2009 to October 2018 | Singleton | Previous singleton sPTB | NR (TVS was not prescribed in study protocol) but  TVS CL <25 mm before intervention started:  I: 10/833 (1.2%)  C: 8/420 (1.9%) | GA between 16+0-20+6 w based on ultrasound at 14+0-20+3 w | I: 17-OHPC 250 mg im/w (Makena),  C: Placebo  Between 16+0-36+6  Randomized  2:1 | None | 1877 assessed,  1740 eligible,  1708  I: 1130  C: 578 | I: 30+0  (5.2)  C: 29+9 (5.2) | Women  I: 1130  C: 578  Children I: 1093  C: 559 | PTB, sPTB <37 w  PTB <35 w (PO)  sPTB <35 w  PTB, sPTB <32 w  PNM, NNM  Neonatal and  maternal  morbidity  NICU admission  Infant follow up  at 23-25 months,  ongoing. |
| Briery  2009  USA  (single center) | NCT  00811057  Funding NR | NR | Twins | 1/3 previous PTB | NR | GA between 20-30 w | I: 250 mg  17-OHPC im/w  C: Placebo  Until 34+6 | None | I: 16  C: 14 | I: 23.3  (5.8)  C: 25.4 (5.0) | I: 16  C: 14 | PTB <37w  PTB <35w (PO)  PTB <34w  GA at delivery  NNM  Neonatal and maternal morbidity  NICU admission |
| Brizot 2015  Brazil  (single center) | NCT  01031017  Funding NR | June 2007 to October 2013 | Twins  (DA) | MC  I: 25%  C: 19% | I: 37.8  (9.7)  C: 38.2 (8.0) | GA between 18-21 w | I: 200 mg natural prog vag/d  C: Placebo  Randomized 1:1  Until 34+6 | None | 390 women Randomized  I: 195  C: 195 | I: 28.1  (6.0)  C: 28.4 (6.2) | I: 189  C: 191 | PTB <37 w  PTB, sPTB <34 w  PTB <32 w  PTB <28 w  GA at delivery  LBW, VLBW  PNM, NNM  Neonatal and  maternal  morbidity  NICU admission |
| Caritis 2009  USA  (14 centers) | STTARS  NCT  00099164  Funding reported | April 2004 to September 2006 | Triplets | DC 30%  ART 70% | NR | GA between 16-20 w | I: 250 mg  17-OHPC im/w  C: Placebo  Randomized 1:1  Until 35w | None | 241 invited,  134 women Randomized  I: 71  C: 63 | I: 30  (20-35)  C: 32  (28-35)  median (IQR) | Women  I: 71  C: 63  Children  I: 212  C: 183 | PTB or fetal loss <35 w (PO)  PTB or fetal loss  <32 w  PTB or fetal loss  <28 w  sPTB <35 w  GA at delivery  LBW, VLBW  PNM, NNM  Neonatal and  maternal  morbidity |
| Cetingoz 2011  Turkey  (single center) | Registra-tion and funding NR | December 2004 to February 2007 | Singleton  I: 51.3%  C: 60%  Twins  I: 48.7%  C: 40.0% | Twin pregnancy, ≥1 previous sPTB, uterine anomaly | I: 34.6  (6.8)  C: 34.3 (6.1) | GA 24 w | I: 100 mg prog vag/d  C: Placebo  Randomized 1:1  Until 36+6 | None | 170 invited,  160 women Randomized  I: 84  C: 76 | NR | I: 80  C: 70 | PTB <37 w (PO?)  PTB <34 w  GA at delivery  NNM  NICU admission  Maternal  morbidity |
| Combs  2010  USA  (14 centers) | NCT  00163020  Funding reported | November 2004 to August 2008 | Triplets  (TCTA) | TCTA  ART  I: 90%  C: 84% | NR | GA between 18-22 w | I: 250mg  17-OHPC im/w  C: Placebo  Randomized 2:1  Until 34+0 | None | 248 assessed  81 women Randomized  I: 56  C: 25 | I: 33.4  (5.0)  C: 33.6 (5.4) | I: 56  C: 25 | PTB <35 w  PTB <32 w  PTB <28 w  GA at delivery  PNM, NNM  Composite  adverse neonatal  outcome (PO)  Maternal morbidity  NICU admission |
| Combs 2011  USA  (14 centers) | NCT  00163020  Funding reported | November 2004 to August 2009 | Twins (DCDA) | 20% fetal reduction  ART  I: 66%  C:58%  Prior PTB:  I: 12%  C: 13% | NR | GA between 16-24 w | I: 250mg 17-OHPC (in 1 mL castor oil) im/w  C: Placebo (1 mL castor oil)  Until 34+0 | None | 1450 assessed  240 women Randomized 2:1  I: 160  C: 80 | I: 34.0  (5.8)  C: 34.5 (6.6) | I: 160  C: 78 | PTB <37 w  PTB, sPTB <34 w  PTB <32 w  PTB <28 w  GA at delivery  LBW, VLBW  PNM, NNM  Composite  adverse neonatal  outcome (PO)  Maternal morbidity |
| Crowther  2017  Australia  (39 centers, Australia  (33),  New Zealand (4), Canada (2)) | PRO-GRESS  ISRCTN  20269066  Funding reported | February 2006 to  September 2012 | Singleton (98.5%)  Twins (1.5%) | Previous sPTB | NR | GA between 20+0- 23+6 w  I: 20.6 w  (19.3-22.1)  C: 20.4 w  (19.3-22.0) median (IQR) | I: 100 mg prog vag/d  (Cyclogest)  C: Placebo  Until 34 w, max 98 days | None | 1919 eligible,  787 Randomized  I: 398:  390 singletons, 8 twin pregnancies  C: 389:  385 singletons, 4 twin pregnancies | I: 30.0  (5.5)  C: 30.3 (5.6) | Women  I: 398  C: 393  Children  I: 406  C: 393 | PTB <37 w  PNM, NNM  Neonatal  morbidity (PO)  Maternal  morbidity  NICU admission |
| Cuijpers 2021  The Netherlands  Long term follow-up of Van Os 2015 (TRIPLE P study) | Registra-tion NR  Funding reported | November 2009 to August 2012 | Singleton | Short TVS CL ≤30 mm  at 18-22 w | NR | NR | I: 200 mg micronized prog vag/d  C: Placebo | None | Randomized  I: 41  C: 39 | I: 31  (26–34)  C: 31 (  29–34)  median (IQR) | Follow up  at 2 years  I: 29  C: 30 | Long-term child  outcome:  Bayley III  cognitive  and motor score  ASQ  CBCL,  Death or  abnormal  development |
| Da Fonseca  2003  Brazil  (single center) | Registra-tion and funding NR | February 1996 to March 2001 | Singleton | Previous sPTB, prophylactic  cerclage, uterine  anomaly | NR | I: 26.5 w  C: 25.2 w | I: 100 mg  vag prog/d  C: Placebo  24-34 w | Antibiotics if positive cervico-vaginal culture.  Home monitoring of contractions | 157 Randomized  I: 81  C: 76 | I: 26.8  C: 27.6 | I: 72  C: 70 | PTB <37 w  PTB <34 w  GA at delivery |
| Fonseca  2007  UK  (multicenter  UK (5), Chile, Brazil,  Greece) | NCT  00422526  Funding reported | September 2003 to May 2006 | Singleton  (90%)  and  twins (DA) (10%) | TVS CL ≤15 mm | I: 11.0  (9-14)  C: 12.0  (9-14)  median (IQR) | Screening at GA 20-25 w (median 22 w)  Randomized at  I: 165 days (159-168)  C: 164 days (160-169)  median (IQR) | I: 200 mg  vag prog/d  (Utrogestan)  C: Placebo  Between 24+0 to 33+6 w | None | 24 620 assessed  413 TVS CL  ≤15 mm,  250 Randomized  I: 125  (11 twins, 8 DC, 3 MC)  C: 125  (13 twins, 9 DC, 4 MC) | I: 29  (24-34)  C: 29  (24-34)  median (IQR) | Women  I: 125  C: 125  Children  I: 136  C:138 | PTB <34 w  sPTB <34 w (PO)  LBW, VLBW  PNM, NNM  Neonatal morbidity  NICU admission |
| Glover  2011  USA  (single center) | NCT  01180296  Funding reported | November 2006 to January 2009 | Singleton | Previous singleton sPTB | NR | GA <20 w | I: 400 mg  oral micronized progesterone/d  C: Placebo | Cerclage if TVS CL <5 mm (none) | I: 20  C: 16 | I: 29.3 (4.7) C: 27.2 (4.9) | I: 19  C: 14 | sPTB <37 w (PO)  GA at delivery  NICU admission |
| Grobman  2012  USA  (14 centers) | NCT  00439374  Funding reported | April 2007 to  May 2011 | Singleton | NulliparousTVS CL <30mm | I: 23.9  (5.6)  C: 23.8 (5.7) | GA between 16+0-22+3 w  I: 21.4 w  (1.2)  C: 21.3 w (1.3) | I: 250 mg  17-OHPC im/w  C: placebo im weekly  (castor oil)  Until 36+6 w | None | 15 435 assessed  657 Randomized  I: 327  C: 330 | I: 22.8  (5.3)  C: 21.6 (4.4) | I: 327  C: 330 | PTB <37 w (PO)  sPTB <37 w  PTB <35 w  PTB <34 w  PTB <32 w  PTB <28 w  GA at delivery  LBW, VLBW  PNM, NNM  Neonatal  morbidity  NICU admission |
| Gyamfi 2009  Secondary analysis of Meis 2003 (singleton) and Rouse 2007 (twins),  USA | Meis 2003: Registra-tion NR  Rouse 2007: SSTARS  NCT  00099164  Funding reported | Meis 2003:  April 1998  to February 2002  Rouse 2007:  April 2004 to February  2006 | Singleton  Meis 2003)  and twins  (Rouse, 2007) | Singleton:  Women with previous sPTB  Twins with or without additional risk factors | NA | GA between 16+0-20+6 w | I: 250 mg  17-OHPC im/w  C: placebo im weekly  (castor oil)  Until 36+6 w (singletons) or 34+6 w (twins) | None | Singleton (Meis 2003):  I: 310  C: 153  Twins (Rouse 2009):  I: 325  C: 330 | Singleton  I: 25.9  (5.6)  C: 26.4 (5.4)  Twins:  I: 29.7  (7.0)  C: 29.6 (6.8) | Singleton  I: 293  C: 148  Twins:  I: 323  C: 330 | GDM |
| Hassan  2011  USA  (10 countries,  44 centers) | PREG-NANT  NCT  00615550  Funding reported | March 2008 to November 2010 | Singleton | TVS CL 10-20 mm (all),  Previous  PTB 16%  (16%) between 20-35 w | I: 17 (2.5)  C: 17 (2.8) | GA between 20+0-23+6 w | I: 90 mg  vag prog gel/d  (Prochive 8%/ Crinone 8%)  C: Placebo  Until 36+6 w | None | 32091 assessed  733 eligible with TVS CL 10-20 mm,  465 Randomized  I: 236  C: 229 | I: 26.5  (5.8)  C: 26.2 (5.1) | I: 235  C: 223 | PTB <37 w  PTB <35 w  PTB <33 w (PO)  PTB <28 w  LBW, VLBW  PNM, NNM  Neonatal  morbidity |
| Hauth  1983  Texas, USA (single center) | Registra-tion and funding NR | July 1977 to March 1981 | Singleton | Women in active duty – military population | NR | GA between 16-20 w | I: 17-OHPC  C: Placebo (castor oil) | None | Women  I: 80  C:88  Children  I: 80  C: 88 | NR | Women  I: 80  C:88  Children  I: 80  C: 88 | PTB <37 w  LBW  PNM  NNM  Maternal  morbidity |
| Hayashi  2021  Japan  (12 centers) | TROPI-CAL  UMIN000013518  Funding NR | April 2014 to March 2018 | Singleton | TVS CL  25 - < 30 mm  Previous PTB  I: 11.9%  C: 16.7% | I: 27.8  (25-29.9)  C: 28  (25-29.8)  median (range) | GA between  I: 22 w  (17.1-24.1)  C: 21.9 w  (16.4 -23.9)  median(range) | I: 200 mg  vag prog/d  C: Placebo  Between 16 and 34 w | If TVS CL < 20 mm before 34 w, treatment was stopped, “other treatment”  was provided | 132 enrolled  and Randomized  I: 65  C: 67 | I: 33  (21-43)  C: 34  (22-41)  median (range) | I: 59  C: 60 | PTB <37 w  PTB <34 w  PTB <28 w  GA at delivery  NNM |
| Ibrahim  2010  Egypt  (single center) | Registra-tion and funding NR | August 2006 to November 2008 | Singleton | Previous PTB | NR | NR | I: 250 mg  17-OHPC  im/w  C: Placebo (saline) | NR | I: 25  C: 25 | I: 25.3  (4.2)  C: 25.6 (3.9) | I: 25  C: 25 | PTB <37 w  GA at delivery  LBW  NNM  NICU admission |
| Jabeen, 2012  Pakistan  (single center) | Registra-tion and funding NR | January 2011 to December 2011 | Singleton | Previous sPTB | NR | GA between 16-20 w | I: 250 mg  17-OHPC im/w  C: Placebo (inert oil) | NR | 60 women  I: 30  C: 30 | I: 29  C: 28 | I: 30  C: 30 | PTB <37 w (PO)  PTB <35 w  PTB <32 w  LBW  PNM  NICU admission |
| Jafarpour  2020  Iran  (single center) | IRCT 20190309042978N2  Funding NR | March 2015 to March 2015 | Singleton | Previous PTB. | NR | NR | I: 250 mg  17 OHPC im/w  C: Routine prenatal care  Between 16-37 w | NR | 100 enrolled  I: 50  C: 50 | I: 24.2 (2.6)  C: 25 (2.38) | I: 50  C: 50 | PTB < 37 w  GA at delivery  LBW |

| Johnson 1975  USA  (single center) | Registra-tion and funding NR | NR | Singleton and twins  Twin n=1 | Two spontaneous abortions immediately before this pregnancy or, one PTB and one spontaneous abortion immediately before this pregnancy or, two PTB at any point. | NR | GA determined by last menstruation  < 24 w | I: 250 mg  17-OHCP im/w  C: Placebo injection once a week | Cerclage if suspicion of cervical incompetence, also, 100 mg prog injected every six hours for 24-36 hours after procedure  I: 4  C: 3  If PTL; iv isoxsuprine | 50 Randomized  43/50 analysed,  37 included in final analysis  I: 18  C: 25 | NR | Per protocol analysis (43/50)  I: 21  C: 22 | LBW  PNM |
| --- | --- | --- | --- | --- | --- | --- | --- | --- | --- | --- | --- | --- |
| Klein  2011  Denmark and Austria  Secondary analysis of Rode 2011 (PREDICT study) | EudraCT 2006-000503-41 and NCT  00329914  Funding reported | June 2006 to October 2008 | Twins | TVS CL  ≤30 mm (≤10^th^ percentile)  Previous sPTB <34 w or late miscarriage >12 w | NR | GA between 21.9 w  (20.6-22.9)  median (IQR) | I: 200 mg prog vag (pessary)/d (Utrogestan), micronized progesterone  C: Placebo | NR | TVS CL ≤30 mm  I: 17  C: 30  Previous sPTB or late miscarriage:  I: 10  C: 18 | TVS CL  ≤30 mm: I: 30.8 (4.7)  C: 31.8(4.3)  Previous PTB or late miscarriage:  I: 33.6 (4.1)  C: 33.3(5.2) | TVS CL ≤30 mm  I: 17  C: 30  Previous sPTB or late mis-carriage:  I: 10  C: 18 | PTB<34 w (PO) |
| Lim  2011  the Netherlands  (55 centers) | ISRCTN 40512715  Funding reported | August 2006 to July 2009 | Multifetal pregnancy (all chorio-nicity, twins, triplets, quads) | Triplets/+  I: 9 (3%)  C: 9 (3%)  (incl.one quadruplet)  MC  I: 57 (17%)  C: 57 (17%)  Fertility treatment I: 140 (42%)  C: 120 (36%) | TVS CL measured in 542 women (81%): 2.4% had CL <25mm  (I: n=9  C: n=4) and,  11.3% had CL<35mm  (I: n=37  C: n=24) | GA between  I: 16.7±1.5 w  C: 16.8±1.6 w | I: 250 mg  17-OHPC im/w  C: Placebo | None | I: 336  C: 335 | I: 32.7  (4.4)  C: 32.8 (4.7) | Women  I: 336  C: 332  Children  I: 681  C: 674 | PTB < 37w  PTB < 32w  PTB < 28w  GA at delivery  LBW, VLBW  NNM  Composite  adverse neonatal  outcome (PO)  NICU admission  Maternal  morbidity |
| Majhi  2009  India  (single center) | Registra-tion and funding NR | December 2004 to February 2006 | Singleton | Previous sPTB | NR | GA between  I: 20.8±2.1 w  C: 20.5±2.4 w | I: 100mg vag prog /d  (Utrogestan),  C: No placebo | If infection was diagnosed, antibiotics was given. | I: 50  C: 50 | I: 26.56 (3.5)  C: 26.42 (3.2) | I: 50  C: 50 | PTB <37 w (PO)  PTB < 34 w (PO)  Neonatal morbidity  NICU admission |
| McNamara 2015  UK  Long term follow-up of  Norman 2009  (STOPPIT  study) | ISRCTN  35782581  Funding reported | After STOPPIT 2008, linkage March 2013, questionnaires mailed 2011 and 2012 | Twins | MC twins I: 46/247  C: 45/247  No MA twins | NR | GA 24+0 | I: 90 mg vag prog/d  C: Placebo | NR | I: 247/494  C: 247494 | NR | Children  I: 386  C: 395 | Long-term child outcome:  Effect of in utero progesterone exposure assessed with Health Utilities Index  CDI score |
| Megli, 2020  USA Secondary analysis with IPD MA of Rouse 2007  (MFMU SSTARS trial) and Combs 2011 (Obstetrix trial) | Registra-tion NR  Funding reported from AMAG Pharma-ceutical | Rouse (2007) April 2004 to February  2006 Combs  (2011)  November 2004 to August 2009 | 2 RCTs with twins | Twin pregnancy (DCDA) with history of PTB | I: 35.5  (1.8)  C: 36.8 (1.6) | GA between  I: 19.3 w (0.4)  C: 18.6 w (0.3) | I: 250 mg/w im 17-OHPC  C: Placebo | NR | Women  I: 34  C: 32  Children  I: 68  C: 64 | I: 31.1  (1.1)  C: 33.2 (1.0) | Women  I: 34  C: 32  Children  I: 68  C: 64 | PTB < 34 |
| Meis  2003  USA  (19 centers) | Registra-tion NR  Funding reported | April 1998  to February 2002 | Singleton | Previous sPTB | NR | GA between  15-20+6 w  I: 18.4 w (1.4)  C: 18.4 w (1.4)  (SD) | I: 250 mg  17-OHPC im/w  C: Placebo  (castor oil)  Until 36 w | None | 2980 assessed  1039 eligible  463 Randomized  2:1  I: 310  C: 153 | I: 26.0  (5.6)  C: 26.5 (5.4) | I: 306  C: 153 | PTB <37 w (PO)  sPTB <37 w  PTB <35 w  PTB <32 w  LBW, VLBW  PNM, NNM  Neonatal and  maternal  morbidity |
| Norman  2009  UK  (9 hospitals) | STOPPIT  ISRCTN  35782581  Funding reported | December 2004 to  April 2008 | Twins | Twins (MC) I: 46/247  C: 45/247  No MA twins | NR | GA 24+0 w | I: 90 mg  vag prog/d  (Crinone)  C: Placebo | NR | 1483  assessed  500 Randomized  I: 247  C: 247 | I: 33 (5)  C: 33 (6) | Women  I: 247  C: 247  Children  I: 494  C: 494 | PTB or IUFD <34 w (one or both twins) (PO)  GA at delivery  PNM, NNM  NICU admission  Maternal morbidity |
| Norman  2016  UK  (66 hospitals, UK (65),  Sweden (1)) | OPPTI-MUM  ISRCTN  14568373  Funding reported | February 2009 to April 2013 | Singleton | FFN pos group: Any of previous PTB, second trimester loss, cervical surgery  FFN neg group: previous sPTB <34 w or  short TVS CL ≤25 mm | I:28.2  (10.6)  C:28.8  (11.1) | GA between 22-24 w | I: 200 mg vag prog/d  C: Placebo  Until 34 w | None | 15132 assessed  1228  Randomized  I: 618  C: 610 | I: 31.5  (5.6)  C: 31.4 (5.8) | Mater-nal  I: 600  C: 597  Neona-tal  I: 589  C: 587  Child-hood  I: 430  C: 439 | IUFD or PTB <34 w (PO)  PNM, NNM  Neonatal morbidity (PO)  NICU admission  Long-term child outcome:  Bayley III cognitive score (PO)  Maternal  morbidity |
| Northen  2007  USA  Follow-up of Meis 2003 | Funding reported from NICHD. | November 2004 to November 2005 | Singleton | Previous PTB | NR | NR | I: 250 mg  17-OHPC im/w  C: Placebo | None | 463 women in original study,  I: 310  C: 153  348 children were potentially eligible for follow-up  I: 194  C: 84 | I: 26.4  (5.8)  C: 26.1 (5.5) | Children I: 194  C: 84 | Long-term child outcomes:  ASQ score  Preschool Activities Inventory |

| O’Brien  2007  USA  (5 countries, 53 centers) | Registra-tion NR  Funding reported | April 2004  to  January 2007 | Singleton | Previous sPTB,  I: 23.6%  C: 25.5% | I: 37 (7)  C: 37 (7) | GA between 18+0-22+6 w  I: 19.9 w (2.1)  C 20.1 w (3.3) mean (SD) | I: 90 mg  vag prog/d (Crinone 8%)  C: Placebo  Until 37 v | None | 711 consented  669 enrolled  659 Randomized  I: 332  C: 327 | I: 27.1  (5.8)  C: 27.3 (5.6) | I: 309  C: 302 | PTB < 37 w  PTB ≤35 w  PTB ≤32 w (PO)  PTB ≤28 w  GA at delivery  PNM, NNM  Neonatal and maternal morbidity  NICU admission |
| --- | --- | --- | --- | --- | --- | --- | --- | --- | --- | --- | --- | --- |
| Price  2021  USA  (2 centers in Zambia) | IPOP  NCT  03297216  Funding; Bill and Melinda Gates Founda-tion and US NIH | February 2018 to January 2020 | Singleton | HIV positive  receiving or intending to start anti-retroviral therapy  Previous sPTB excluded. | I: 41  (35-46)  C: 40  (36-46)  median IQR) | GA between  I: 19.3 w  (16.9-21.6)  C: 18.7 w  (16.7-21.3)  median (IQR) | I: 250 mg  17-OHPC im/w  C: Placebo  Until 37 w | NR | 1042 assessed  800 enrolled and Randomized  I: 399  C: 401 | I: 29  (25-33)  C: 30  (25-34)  median (IQR) | I: 399  C: 401 | PTB, sPTB <37 w  PTB <34 w  PTB <28 w  LBW, VLBW  PNM, NNM,  NICU admission  Maternal  mortality  morbidity |
| Rai  2009  India  (single center) | Registra-tion and funding NR | January 2005 to December 2006 | Singleton | Previous sPTB | NR | GA between  I: 20.69 w  (2.83)  C: 20.73 w  (1.78)  mean (SD) | I: 200 micronized oral prog/d  C: Placebo | Tocolysis given  I: 20.3%  C: 27% | I: 75  C: 75 | I: 26.07 (3.24)  C: 25.72 (3.42) | I: 74  C: 74 | Delivery ≥37 w (PO)  PTB 34-<37 w  PTB 32-<34 w  PTB 29-<32 w  PTB <28 w  GA at delivery  NNM  NICU admission |
| Rehal  2021  (6 countries, 22 hospitals) | EudraCT 2015-005180-16 and ISRCTN 66445401  Funding reported. | May 2017 to April 2019 | Twins | MC I: 23%  C: 23%  ART I: 34%  C: 35% | I: 34.4 (31.0-38.0)  C: 34.0 (30.0-37.6)  median (IQR) | GA between  I: 13.2 w  (12.7-13.6) I  C: 12.2 w  (12.7-13.7)  median (IQR) | I: 600mg  vag prog/d  C: Placebo  11-14 w until 34 w | NR | I: 582  C: 587 | I: 34.1 (30.3-37.7)  C: 34.0 (30.0-37.6)  median (IQR) | Women  I: 582  C: 587  Children  I: 1125  C: 1113 | sPTB 24 w-<34 w (PO)  sPTB between 24 and <28 w, <37 w  PTB between 24 and <28 w, <32, <34, <37 w  PTB between randomization and <28, <32, <34, <37w  VLBW  PNM  Maternal morbidity |
| Rode  2011  Denmark and Austria  (17 centers,  Denmark (13)  Austria (4)) | PREDICT  EudraCT 2006-000503-41 and NCT  00329914  Funding reported. | June 2006 to October 2008 | Twins | MC I: 43/334 (12.9%)  C: 57/343 (16.6%) | NR | GA between  I: 146.0 days (139–157)  C: 146.5 days  (139–158)  median (IQR) | I: 200 mg prog vag/d (pessary) (Utrogestan)  C: Placebo pessary | NR | I: 334  C: 343 | I: 32.0  (4.5)  C: 31.9 (4.4) | I: 334  C: 341 | PTB <37 w  PTB <34 w (PO)  sPTB <34 w  PTB <32 w  PTB <28 w  GA at delivery  LBW, VLBW  PNM, NNM  Neonatal and maternal morbidity  NICU admission  Long-term child outcome: ASQ |

| Rouse  2007  USA  (14 centers) | SSTARS  NCT  00099164  Funding reported. | April 2004  to February  2006 | Twins  (DCDA 82%,  MCDA 18%) | Twins (MC)  I: 18%  C: 17.1% | NR | GA between 16+0-20+6 w  Randomized at  I: 19.2 w (1.5)  C: 19.2 w (1.4)  mean (SD) | I: 250 mg  17-OHPC im/w  C: Placebo  Until 34 w | Cerclage  I: 1.9%  C: 1.2% | 1526 eligible  699  consented  661  random.  I: 327  C: 334 | I: 29.7 (7.0)  C: 29.6 (6.8) | I: 325  C: 330 | PTB or IUFD <37 w  PTB or IUFD <35 w (PO)  PTB or IUFD <32 w  PTB or IUFD <28 w  sPTB or IUFD <35 w  GA at delivery  LBW, VLBW  PNM  Maternal morbidity |
| --- | --- | --- | --- | --- | --- | --- | --- | --- | --- | --- | --- | --- |
| Saghafi  2011  Iran  (single center) | Registration and funding NR | 2007 to 2008 | NR  handled as singleton | Previous PTB | NR | Randomized at GA 16 w | I: 250 mg  17-OHPC im/w  Between 16-37 w  C: No placebo | NR | I: 50  C: 50 | I: 29.98 (5.36)  C: 29.32 (5.69) | I: 50  C: 50 | PTB <37 w  PTB <34 w  GA at delivery |
| Serra  2013  Spain  (5 university clinics) | EudraCT  2004-004136-31, NCT  00480402  and EF489-2004/1  Funding reported | December 2005 to January 2008 | Twins (DCDA) | ART  I1: 92/96 (95.8%)  I2: 94/97 (96.9%)  C:96/97 (99.0%) | I 1: 42.1 (7.7)  I:2: 42.4 (7.9)  C: 43.5 (7.6)  TVS CL  <25 mm  I1: 2.1%  I2: 2.1%  C: 1.0% | Randomized at GA 20 w | I 1: vag I1: 200 mg vaginal progesterone (pessary) /d  I2: 400 mg vaginal progesterone (pessary)/d  C: Placebo  Treatment from 20 to 34 weeks | None | I1: 98  I2: 98  C: 98 | I1: 33.5 (4.6)  I2: 33.5  (4.0)  C: 33.3 (5.2) | Women  I1:96  I2: 97  C: 96  Children  I1: 194  I2:191  C:190 | PTB <37 w (PO?)  sPTB <37 w  PTB <34 w  PTB <32 w  PTB <28 w  GA at delivery  LBW, VLBW  PNM, NNM  Neonatal and maternal morbidity  NICU admission |
| Shadab  2018  Pakistan  (single center) | Registra-tion and funding NR | NR | Singleton | Previous sPTB | NR | GA between 16-20 w | I: 250 mg  17-OHPC im/w  C: Placebo (Vitamin B)  Until 37 w | None | I: 66  C: 66 | I: 26.75 (3.76)  C: 29.99 (1.43) | I: 66  C: 66 | PTB <37 w  GA at delivery |
| Shahgheibi  2016  Iran  (single center) | IRCT  2014101019222N2  Funding reported | 2013 to 2014 | Singleton | Previous sPTB or  uterine anomaly | NR | GA between 24-34 w | I: 250 mg  17-OHPC im/w  C: Placebo | None | I: 50  C: 50 | I: 25.4 (2.58)  C: 27.4 (2.21) | I: 50  C: 50 | PTB <37 w |
| Van Os  2015  the Netherlands  (7 university hospitals, 23 general hospitals) | TRIPLE P  Registra-tion NR  Funding reported | November 2009 to August 2012 | Singleton | TVS CL  <30 mm,  no previous PTB | I: 26  (23-29)  C: 27  (25-28)  median (IQR) | GA between  I: 21.7 w  (20.7-22.6)  C: 21.6 w  (20.9-22.7)  median (IQR) | I: 200 mg micronized prog/d  C: Placebo | Standard care | I: 41  C: 39 | I: 31 (5)  C: 30 (5) | I: 41  C: 39 | PTB, sPTB <37 w  PTB, sPTB <34 w  PTB, sPTB <32 w  LBW, VLBW  PNM, NNM  Neonatal and  maternal  morbidity  NICU admission |
| Vedel  2016  Denmark  Follow-up of Rode 2011 (PREDICT study) | EudraCT 2006-000503-41 and NCT  00329914  Funding reported | June 2006 to October 2008.  questionnaire February 2012 to July 2013.  Register data April 2014 to November 2014. | Twins (DA) | ART  I: 50.0%  C: 47.2% | NR | GA between  I: 144 days  (138-158)  C: 145 days  (137-158)  median (IQR) | I: 200 mg micronised vag prog (pessary)  C: Placebo | None | I: 114  C: 106  women  (ASQ) | I: 32.2  (4.1)  C: 32.2 (4.2)  From PREDICT  I: 248  C: 250  median (IQR) | Women:  I: 114  C: 106  Children  I: 492  C: 497  (register-data)  I: 225  C: 212  (ASQ) | Long-term child outcome: ASQ  Medical history up to 8 years. |
| Wood  2012  Canada  (2 centers) | NCT 00343265  Funding reported | June 2006 to October 2010 | Twins and triplets | ART  I: 55%  C: 60%  Selective reduction: 5% vs 0%  Triplets:  I: 2 (5%)  C: 1 (2%) | NR | GA between 16+0-20+6 w  I: 19+2 w  (1-3)  C: 19+6 w  (1-3)  median (IQR) | I: 90 mg, 8% /d vag prog gel  C: Placebo  Until 35+6 w | None | Women  I: 42  C: 42  Children  I: 86  C: 85 | I: 34  (19-43)  C: 34  (22-44  median (range) | Women  I: 42  C: 42  Children  I: 86  C: 85 | PTB, sPTB <37 w  PTB <35 w  GA at delivery  PNM, NNM  Neonatal morbidity |
| Yemini  1985  Israel | Registra-tion and funding NR | NR | Singleton | Previous ≥2 PTB or ≥2 spontaneousmiscarriages | NR | GA between  I: 12.2 w (3.3)  C: 12.2 w (3.) | I: 250 mg  17-OHPC im/w  C: Placebo  Until 37 w | All had cerclage | I: 39  C: 40 | I: 27.8  (4.6)  C: 28.3 (5.2) | I: 39  C: 40 | PBT <37 w  Neonatal morbidity |
| Systematic reviews with individual patient data meta-analysis and unique study data | | | | | | | | | | | | |
| EPPPIC group  2021 | EPPPIC study  PROS-PERO, CRD  42017068299  Funding; PCORI award  PPA-1608-35702 | 1946 April 2017, updated  July 2019 | 31 RCTs with  singleton  or multifetal pregnancies | Asympto-matic women at increased risk of PTB | TVS CL ≤25 mm  Singletons  Vag. prog.  26.6%  17-OHPC  14.3%  Multifetal  pregnancy  Vag. prog.  3.5%  17-OHPC  10.0% | GA between 14-25 w | I: vag prog (14 RCTs),  17-OHPC im (13 RCTs)  oral prog (2 RCTs),  C: Placebo,  standard care,  or other forms of prog  (2 RCTs compared vag prog vs 17-OHPC im)  Treatment until  34-37 w | NR | 11644 women,  16185 children | Singletons  Vag prog  29.2  (5.9)  17-OHPC 27.6  (6.1)  Multifetal  pregnancies  Vag. prog. 31.3  (5.4)  17-OHPC 31.2  (5.9) | Singletons  Vag prog  I: 1904  C: 1865  17-OHPC  I: 1889  C: 1164  Oral prog  I: 93  C: 88  Multifetal pregnancies Vag prog I: 1073  C: 973  17-OHPC  I: 1240 | PTB <34 w  (PO, only  outcome with  unique study  data) |

| Norman  2018  UK  HTA-report | OPPTI-  MUM  ISRCTN  14568373 | January 2013 to June 2016 | One RCT with singleton pregnancies | FFN pos group: Any of previous PTB, second trimester loss, cervical surgery  FFN neg group: previous sPTB <34 w or  short TVS CL ≤25 mm | I: 28.2 (10.6)  C: 28.8 (11.1) | GA between 22-24 w | I: 200 mg  vag prog/d  C: Placebo  Until 34 w | Cerclage recorded; other interventions not prohibited but not recorded | 15132  assessed  1228  Randomized  I: 618  C: 610 | I: 31.5  (5.6)  C: 31.4 (5.8) | Maternal  I: 600  C: 597  Neonatal  I: 589  C: 587  Child-hood  I: 430  C: 439  Children with Bailey score at 2 years  I: 410  C: 423 | Long-term child outcome:  Bayley-III cognitive  composite score  (PO, only  outcome  with unique study  data) |
| --- | --- | --- | --- | --- | --- | --- | --- | --- | --- | --- | --- | --- |
| Romero  2017 | PROS-PERO  CRD  42016039682  Funding reported | From inception to  December  2016 | 6 RCTs with twin pregnancies | Asympto-matic women with short 2^nd^ trimester TVS CL  ≤25 mm and a twin pregnancy | All TVS CL  ≤25 mm | GA between  I: 21.7 (20.6-23.1) w  C: 22.1 (21.1-23.3) w  median (IQR) | I: 90-200 mg vag prog/d  C: Placebo or standard care  Treatment until 34-36 w | NR | 303 women, 606 children | I: 27  (25-30)  C: 28  (25-31)  median (IQR) | Women  I: 159  C: 144  Children  I: 318  C: 288 | PTB <33 w  (only outcome  with unique study  data) |
| Romero  2018 | PROS-PERO  CRD  42017057155  Funding reported | From inception to September 2017 | 5 RCTs  with singleton  pregnancies | Asympto-matic women with singleton  pregnancies  and short 2^nd^ trimester TVS CL  ≤25 mm | All TVS CL  ≤25 mm | GA between  I: 22.6 (21.4–23.6) w  C: 22.6 (21.4–23.4) w  median (IQR) | I: 90-200mg vag prog/d  C: Placebo  Treatment until 34-36 w | NR | 974 women | I: 28.0 (23.6-33.0)  C: 27.5  (23.5-32.8)  median (IQR) | I: 498  C: 476 | PTB <33 w  (only outcome  with unique study  data) |

| Romero 2022  Update IPD MA of Romero 2017 | Funding reported | From inception to  November 2021 | 6 RCTs with twin pregnancies | Asympto-matic women with short 2^nd^  trimester TVS CL  ≤25 mm and a twin pregnancy | All TVS CL ≤25 mm | NR | I: 100-600 mg vag prog/d  C: Placebo | NR | 95 women  190 children | NR | I: 52  C: 43 | PTB <33 w  (only outcome  with unique  study data) |
| --- | --- | --- | --- | --- | --- | --- | --- | --- | --- | --- | --- | --- |
| Simons  2021 | PROSPERO CRD  42019142422  Funding NR | From inception to May 2020 | 7 RCTs  (based on 5 RCTs) with singleton (3) and multifetal pregnancies (4) | Children born to women who received progesterone treatment for any indication during pregnancy | NR | NR | I: Prog treatment  C: Placebo or another intervention | NR | 4222 children aged 6 m to 8 years | NR | Children  2580  Singleton:  1206  Multiples:  1374 | Long-term child  outcome:  Composite  Bailey  score |
| Cerclage | | | | | | | | | | | | |
| Althuisius  2001  The Netherlands | CIPRACT  Registra-tion NR  Funding reported | July 1995 to July 2000 | Singleton | Previous PTB <34w, PPROM <32w or, cold knife conization and TVS CL <25 mm at <27 w or, uterine anomaly. | I: 19.9  (2.9)  C: 19.6 (4.3)  Before 27 w. | GA between  I: 20.9 w (3.0)  C: 20.4 w (3.3)  median (IQR) | I: Cerclage (McDonald) and bed rest  C: No cerclage, bed rest | NR? | 36 women Randomized  I: 19  C: 16 | I: 30.5 (4.6)  C: 34.5(4.9) | <27 w  I: 19  C: 16 | PTB <34 w (PO)  PNM (PO)  Neonatal morbidity (PO) |

| Berghella 2004  USA  (2 centers) | Registra-tion and funding NR | February 1998 to June 2003 | Singleton and twins (4/61, 7%) | ≥1 of high-risk factors for preterm birth (≥1 preterm birth <35  w, ≥2 curettages, diethyl-stilbestrol exposure, cone biopsy, Mullerian anomaly,  or twin pregnancy)  and/or TVS CL < 25 mm or significant funnelling | I: 15.7  (9.2)  C: 16.7 (8.0) | GA between 14-23 w | I: Cerclage  (McDonald) and bed rest  C: No cerclage, bed rest  Randomized 1:1 | Bed rest | 451 invited,  61 women Randomized  (6 lost to follow-up)  I: 31  C: 30 | I: 27.8  (6.4)  C: 29.9 (6.9) | I: 14/31  C: 14/30  <35 w | PTB <35 w (PO),  PTB <34 w  PTB <32 w  PTB <28 w  GA at delivery  NNM  Neonatal and  maternal  morbidity  NICU admission |
| --- | --- | --- | --- | --- | --- | --- | --- | --- | --- | --- | --- | --- |
| Dor  1982  Israel  (single center) | Registra-tion and funding NR | 1975 to 1979 | Twins | Twin pregnancy | NR | GA 13 w | I: Cerclage  (McDonald)  C: No cerclage | None | 45/90 | I: 28.1  C: 30.4 | Women  I: 22  C: 23  Children  I: 44  C: 46 | PTB <37 w  PTB <33 w  PTB <28 w  NNM  Maternal  morbidity |
| Ezechi, 2004 Nigeria | Registra-tion and funding NR | June 2000 to June 2002 | NR  handled as singleton | Previous PTB | NR | GA 14 w | I: Cerclage  (McDonald)  C: No cerclage | None | I: 38  C: 43 | I: 24.6  (4.4)  C: 24.4 (4.5) | I: 38  C: 43 | PTB, not  defined in text but  seems to be <37w  GA at delivery  LBW  PNM  NICU admission |

| Lazar  1984  France  (four centers) | Registra-tion and funding NR | NR | Singleton | Composite score of a combination of:  Previous PTB 29-36 w, history of previous miscarriage, prior threatening PTL treated by hospitaliza-tion, uterine malfor-mation, previous forced cervical dilatation, low lying placenta with bleeding, CL <2 cm, cervix open for inner os | NR | NR | I: Cerclage  (McDonald)  C: No cerclage | Hospitali-zation after cerclage 1-2 days | I: 268  C: 238 | I: 26.6  C: 26.4 | I: 268  C: 238 | PTB <37 w  PTB <35 w  PTB <32 w  PNM |
| --- | --- | --- | --- | --- | --- | --- | --- | --- | --- | --- | --- | --- |

| Macnaught-on  1993  UK, France, Hungary, Norway, Italy, Belgium, Zimbabwe, South Africa, Iceland, Ireland, the Netherlands, Canada | MRC/  RCOG  Registra-tion NR  Funding reported | 1981 to 1988 | Singleton (98%) and twins (2%) | Included if physician was uncertain if to place a cerclage or not for at risk patients with risk factors: previous PTB, previous 2^nd^ trimester miscarriage, previous early abortion, cervical amputation, cone biopsy, twin pregnancy, uterine anomaly | NR | GA between  I: 15.6 w  (5.1)  C: 14.9 w  (5.1)  mean (SD) | I: Cerclage  (type not prespecified)  C: No cerclage | Bedrest, hospital admission, tocolysis | 1318 enrolled  1292 Randomized  I:647  C:645 | I: 27.7  (5.1)  C: 27.2 (5.0) | Women  I: 647  C: 645  Children  I: 659  C: 661 | PTB <37 w  PTB <33 w (PO)  LBW, VLBW  PNM, NNM |
| --- | --- | --- | --- | --- | --- | --- | --- | --- | --- | --- | --- | --- |
| Otsuki  2016  Japan  (60 centers) | UMIN000001870  Funding reported | 2004 to 2009 | Singleton | General population screening; TVS CL  <25 mm  History of PTB:  11-15%, History of previous abortion:  20% | I1:18.3  (5.0)  I2: 16.9  (4.5)  C: 16.4  (5.9) | GA between  I1: 24.6 w  (2.8)  I2: 24.6 w  (2.9)  C: 24.0 w  (3.2)  mean (SD) | I1: Cerclage (Shirodkar)  I2: Cerclage  (McDonald)  C: Bedrest  Cerclage removed at 37 w | All were screened for infection or inflammation of the LGT, exclusion if LGTI was diagnosed | I1: 35  I2: 36  C: 35 | I1: 33.4  (4.5)  I2: 33.2  (4.9)  C: 33.9  (3.6) | I1: 34  I2: 34  C: 30 | PTB < 37 w  PTB < 34 w  PTB < 32 w  PTB < 28 w  NNM |

| Owen  2009  USA  (15 centers) | Registra-tion NR  Funding reported | January 2003 to November 2007 | Singleton | History of sPTB + TVS CL <25 mm | At randomi-  zation  I: 19.5  (5.3)  C: 18.6 (6.3) | GA at  randomization  I: 19.5 w (2.0)  C:19.4 w (1.9)  mean (SD) | I: Cerclage  (McDonald)  C: standard care  Cerclage removed at 37 w | Antibiotics if positive culture | 8770 assessed  1044 with prior SPTB  screened with TVS CL:  318  TVS CL <25 mm  302 Randomized  I: 149  C: 153 | I: 26.6  (5.1)  C: 26.4 (5.5) | I: 148  C: 153 | PTB <37 w  PTB <35 w (PO)  PNM  Neonatal morbidity |
| --- | --- | --- | --- | --- | --- | --- | --- | --- | --- | --- | --- | --- |
| Roman  2020  (8 centers, Italy, USA, Spain, Poland, Denmark, Switzerland | NCT 02490384  External funding NR | July 2015 to July 2019 | Twins (DA) | Asymptomatic women with twin pregnancy, with dilated cervix 1-5 cm by pelvic examination and/or speculum examination and/or TVS | I: 10/14 had CL<25mm  C: 10/10 had CL<25mm | GA between  I: 20.7 w (1.7)  C: 19.4 w (1.5)  mean (SD) | I: Cerclage  McDonald)  C: No cerclage | I: Indo-methasin and antibiotics | I: 17  C: 13 | I: 31.6 (4.4)  C: 28.2 (5.1) | I: 17  C: 13 | sPTB <34 w (PO)  sPTB <32 w  sPTB <28 w  VLBW  PNM, NNM  Neonatal and maternal morbidity  NICU admission |
| Rush  1984  South Africa  (single center) | Registra-tion and funding NR | January 1979 to April 1982 | Singleton | History of previous late miscarriage or PTB out of ≥1  between 14-36 w, total ≥2 pregnancies ending <37 w | NR | GA between  I: 124.1 days  (12.9)  C: 126.2 days  (14.0) | I: Cerclage  McDonald)  C: No cerclage | None | I: 96  C: 98 | I: 26.16 (4.40) C: 25.79 (4.09) | I: 96  C: 98 | PTB <37 w  LBW  Neonatal and maternal morbidity |
| Rust  2000  USA  (single center) | Registra-tion and funding NR | May 1998 to June 1999 | Singleton (88,5%) and twins (11,5%) | (1) width of dilatation of the internal os, (2) depth of membrane prolapses into the endo- cervical canal, (3) distal CL, and (4) total CL | I: 39 (12)  C: 36 (12) | GA between 16-24 w | I: Cerclage  (McDonald)  C: Modified bedrest | I: Bedrest 48-72 h, clindamycin and indo-methasin | I: 31  C: 30 | NR | I: 31  C: 30 | PTB <37 w  PTB <34 w  PTB <28 w  GA at delivery  NNM  Neonatal and maternal morbidity |
| To  2004  UK  (6 countries; UK, Brazil, South Africa, Slovenia, Greece, Chile; 12 hospitals) | Registra- tion and funding NR | January 1998 to May 2002 | Singleton | TVS CL ≤15 mm.  Previous cervical surgery: I: 6%  C: 7% | I: 9.6  (2-15)  C: 9.3  (2-15)  mean (range) | GA between 22-24 w  I: 23.5 w  (22.3-25.9)  C: 23.6 w  (22.3-25.3)  mean (range) | I: Cerclage (Shirodkar)  C: Standard care  Cerclage removed  at 37 w | Steroids at 26-28 w for fetal lung maturation  for all | 47123 assessed  470 eligible  253  Randomized  I: 127  C: 126 | I: 29.8 (14.7-43.0)  C: 29.3 (13.9-41.2)  mean (range) | I: 127  C: 125 | PTB <33 w (PO)  GA at delivery  PNM, NNM  Neonatal and maternal morbidity |
| Systematic reviews with individual patient data meta-analysis and unique study data | | | | | | | | | | | | |
| Alfirevic 2017 | Cochrane review  Registra- tion and funding NR | From inception to June 2016 | 15 RCTs of singleton pregnancies | History of PTB or short TVS CL | NR | NR | I: Cerclage  C: No cerclage, other intervention | Bedrest in some studies | 3490 women | NR | Women  3490 | PTB <37 w  PTB <34  PTB <28 w  PNM (PO)  Neonatal and maternal morbidity |
| Pessary | | | | | | | | | | | | |
| Berghella  2017a  USA  (3 centers) | PoPPT  NCT 02056639  Funding NR | April 2014 to  June 2016 | Twins | TVS CL <30 mm  DCDA 78% MCDA22%  Previous PTB 0% vs 13% | I: 16.7 (10.7-27.8)  C: 22.9 (15.9-25.6)  median (IQR) | GA between 18+0-27+6 w  I: 21.0 w  (20.1-24.3)  C: 21.2 w  (21.1-24.3) median (IQR) | I: Pessary  (Bioteque cup™)  C: No pessary  Pessary removed at 36 w | Progesterone  I: 1  C: 2 | 421 assessed  85  TVS CL ≤30 mm  46 Randomized  I: 23  C: 23 | I: 27.0 (23.4-33.0)  C: 32.9 (26.2-36.8)  median (IQR) | Women  I: 23  C: 23  Children  I: 46  C: 46 | PTB, sPTB <37 w  PTB, sPTB <34 w  PTB, sPTB <28 w  GA at delivery  NNM  Neonatal and maternal  morbidity |
| Dugoff  2018  USA  (5 centers) | PoPPS  NCT  02056652  Funding reported | March 2014 to July 2016 | Singleton | TVS CL ≤25 mm | I: 17.6 (10.9-22.0)  C: 19.0 (11.2-22.9)  median (IQR) | GA between 18+0-23+6 w  I: 20.9 w  (20.1-21.9)  C: 20.7 w  (20.1-22.1)  median (IQR) | I: Pessary (Biotech™ cup)  C: No pessary  Pessary removed at 37 w | Vag prog if TVS CL  ≤20 mm  I: 84%  C: 91%  Cerclage  I: 2 (3.3%)  C: 3 (5.2%) | 17383 assessed  391 met  inclusion criteria,  122 agreed to randomi-zation  I: 61  C: 61 | I: 27.7 (23.0-32.3)  C: 29.5 (23.0-34.8)  median (IQR) | I: 60  C: 58 | PTB <37 w (PO)  sPTB <37 w  PTB, sPTB <34 w  PTB, sPTB <28 w  GA at delivery  NNM  Neonatal and  maternal  morbidity |
| Goya  2012  Spain  (5 centers) | PECEP  NCT  00706264  Funding reported | June 2007 to June 2010 | Singleton | TVS CL ≤25 mm  Previous PTB: 11% in both groups | I: 19.0 (4.6)  C: 19.0 (4.9) | GA between  I: 22.2 w  (0.9)  C: 22.4 w  (0.9) | I: Pessary (Arabin)  C: Standard care  Pessary removed at 37 w | None | 18235 eligible,  6360 declined,  11875 assessed,  726 TVS CL ≤25 mm  385 Randomized  I:192  C:193 | I: 30.3  (5.1)  C: 29.6 (5.4) | I: 190  C: 190 | sPTB <37 w  PTB <34 w  sPTB < 34 w (PO)  sPTB < 28 w  GA at delivery  LBW, VLBW  NNM  Neonatal and maternal  morbidity |
| Goya  2016  Spain  (5 centers) | PECEP-twins  NCT  01242410  Funding NR | January 2011 to July 2014 | Twins | TVS CL ≤25 mm.  Previous PTB:  I: 16.7% C: 17.6%  Twins (MC)  I: 19.1%  C: 17.6% | I: 19.2  (3.5)  C: 19.6 (3.6) | GA between 18-22 w  I: 22.1 (0.8)  C: 22.5 (0.7) | I: Pessary (Arabin)  C: Standard care  Pessary removed at 37 w | None | 2931 eligible,  2287 assessed,  154  TVS CL ≤25 mm,  137 Randomized  I: 68  C: 66 | I: 35.4  (3.6)  C: 35.9 (5.6) | Women  I: 68  C: 65  Children  I: 136  C: 130 | sPTB <37 w  PTB <34 w  sPTB < 34 w (PO)  sPTB <28 w  GA at delivery  LBW, VLBW  NNM  Neonatal and maternal  morbidity |
| Hui  2013  China  (single center) | ISRCTN  18185477  Funding reported | October 2008 to February 2011 | Singleton | TVS CL ≤25 mm | I: 19.6  (0.5)  C: 20.5 (0.4) | GA between 20-24 w | I: Pessary (Arabin)  C: Digital examination at entry to simulate pessary insertion.  Pessary removed at 37 w | Dexa-methasone if TVS CL <10 mm | 4438 assessed, 203 TVS CL <25 mm, 17 not eligible, 78 declined, 108 consented, I: 53  C: 55 | I: 31.6  (4.7)  C: 31.8 (5.3) | I: 53  C: 55 | PTB <37 w  PTB < 34 w (PO)  sPTB <34 w  PTB <28 w  GA at delivery  NNM  Neonatal and maternal  morbidity  NICU admission |
| Karbasian, 2016  Iran  (single center) | Registra- tion NR  Funding reported | August 2014 to December 2015 | Singleton | TVS CL <25 mm | I: 22 (1.8)  C: 22 (1.6) | GA between 18-22 w | I: Pessary (Arabin) +400 mg/d vag prog  C: 400 mg/d vag prog | Proges-terone | I: 71  C: 73 | I: 28.8 (5.3) C: 27.9 (5.4) | I: 71  C: 73 | PTB <37 w (PO)  PTB <34 w  PTB <32 w  GA at delivery  LBW  PNM, NNM  NICU admission  Maternal  morbidity |

| Liem  2013a  the Netherlands  (40 hospitals) | ProTWIN  NTR 1858  Funding reported | September 2009 to  March 2012 | Twins (98%)  Triplets (2%) | Triplets:  I: 2% (n=9)  C: 2% (n=9)  Twins (MC): I: 22%  C: 24%  Previous PTB  I: 7%  C: 6% | CL measured  at GA 16-22 w  I: 43.6  (8.1)  C: 44.2 (8.5) | GA between  16-20 w  I: 18.4 (1.7)  C: 18.6 (2.3) | I: Pessary (Arabin)  C: Standard care  Pessary removed at 36 w | Cerclage  I: 5  C: 0 | 1242 assessed  813  Randomized  I: 403  C: 410 | I: 33.1  (4.6)  C: 32.7 (4.5) | Women  I: 401  C: 407  Children  I: 811  C: 823 | PTB <37 w  PTB <32 w  PTB <28 w  GA at delivery  LBW, VLBW  NNM  Composite adverse neonatal outcome (PO)  Neonatal morbidity (PO)  NICU admission (PO)  Maternal mortality and morbidity |
| --- | --- | --- | --- | --- | --- | --- | --- | --- | --- | --- | --- | --- |
| Nicolaides  2016a  UK  (12 countries,  23 centers) | ISRCTN  01096902  Funding reported | August 2008 to May 2011 | Twins | Twins (MC): I: 18.8%  C: 18.8%  Previous PTB: I: 8.8%  C: 14.3% | I: 32.0 (27.0-36.0)  C: 32.0 (27.0-37.0)  median (IQR) | GA between 20+0-24+6 w  I: 22+6 w  (21.4-23.9)  C: 22.7 w  (21.4-23.9)  median (IQR) | I: Pessary (Arabin)  C: Standard care  Pessary removed at 37 w | Steroids if TVS CL <10 mm after 26 w  Vag prog  I:0  C:2 | 2107  eligible  1180 Randomized  I:590  C: 590 | I: 33.1 (29.5-36.7)  C: 33.2 (29.1-36.6)  median (IQR) | Women  I: 588  C: 589  Children  I: 1176  C: 1178 | sPTB <34 w (PO)  PTB <34 w  PTB <32 w  PTB <28 w  GA at delivery  LBW, VLBW  PNM, NNM  Neonatal morbidity |
| Nicolaides  2016b  UK  (9 countries,  16 centers) | ISRCTN  01096902  Funding reported | September  2008 to  January 2013 | Singleton | Short TVS CL ≤25 mm Previous PTB  I: 15.1%  C: 18% | I: 20 (14-22)  C: 20  (15-22)  median (IQR) | GA between  20+0-24+6 w  I: 23.4 w (22.6-24.3)  C: 23.6 w (22.7-24.4) median (IQR) | I: Pessary (Arabin)  C: Standard care  Pessary removed at 37 w | Vag prog if TVS CL  ≤15 mm  I: 204  (43.9%)  C: 219 (46.9%)  Antibiotics:  I: 156  C: 111  Cerclage  I: 2  C: 5 | 1829 eligible  935 Randomized  I: 466  C: 469 | I: 30.1 (26.0-34.2)  C: 29.5 (25.4-34.1)  median (IQR) | I: 460  C: 464 | sPTB <34 w (PO)  PTB <34 w  PTB <32 w  PTB <28 w  GA at delivery  LBW, VLBW  PNM, NNM  Neonatal morbidity  NICU admission |

| Norman  2021 | STOPPIT-2  NCT  02235181  Funding reported | April 2015 to February 2019 | Twins | TVS CL <35 mm  MCDA 20%  DCDA 80% | I: 28.8  (5.8)  C: 29.5 (5.1) | GA between 18+0–21+6 w | I: Pessary (Arabin)  C: Standard care  Pessary removed at 37 w | None | 503 Randomized  I: 250  C: 253 | I: 32.4 (17.51)  C: 32.7 (17.50) | Women  I: 246  C:245  Children  I: 492  C: 490 | sPTB < 34 w (PO)  PTB, sPTB <37 w  PTB, sPTB <34 w  PTB, sPTB <32 w  PTB, sPTB <28 w  GA at delivery  PNM  Composite adverse neonatal outcome (PO)  Neonatal morbidity  NICU admission  Maternal  morbidity |
| --- | --- | --- | --- | --- | --- | --- | --- | --- | --- | --- | --- | --- |
| Pacagnella  2022  Brazil | ReBec  U1111-1164-2636  Funding reported | July 2015 to March 2019 | Singleton (92.4%)  Twins (7.6%) | TVS CL <30 mm  Twin pregnancy (all DA) | I: 25  (20.7-27.0)  C: 25.0 (21.1-27.0)  median (IQR) | Within 72h after randomization  GA at randomization  I: 21.2 w  (20.0-22.3)  C: 21.1 w  (20.0-22.1)  median (IQR) | I: Pessary (Ingamed) and vag prog  C: vag prog  Pessary removed at 36 w | None | 936 Randomized  I: 475  C: 461 | I: 26.5  (7.0)  C: 26.3 (6.6) | Women  I: 463  C:436  Children I: 509  C: 468 | PTB <37 w  sPTB <37 w (PO)  PTB, sPTB <34 w (PO)  PTB, sPTB <32 w (PO)  PTB <28 w  sPTB <28 w (PO)  GA at delivery  PNM, NNM  Neonatal morbidity (PO)  NICU admission (PO)  Maternal morbidity |

| Saccone  2017c  Italy  (single center) | NCT  02716909  Funding NR | March 2016 to May 2017 | Singleton | TVS-CL ≤25 mm  Prior cervical surgery:  I: 7 (4.7%)  C: 5 (3.3%) | I: 11.5  (5.7)  C: 12.5 (5.9) | GA between 18+0-23+6 w | I: Pessary (Arabin)  C: Standard care  Pessary removed at 37 w | If TVS CL  ≤ 20mm, vag prog 200 mg/d in both groups:  I: 133  (88.7%)  C: 125 (83.3%) | 503 women eligible  300 women Randomized  I: 150  C: 150 | I: 28.5  (6.2)  C: 28.9 (6.5) | I: 150  C: 150 | PTB, sPTB <37 w  PTB <34 w  sPTB <34 w (PO)  PTB, sPTB <32 w  PTB, sPTB <28 w  GA at delivery  LBW, VLBW  PNM, NNM  Neonatal and maternal  morbidity  NICU admission |
| --- | --- | --- | --- | --- | --- | --- | --- | --- | --- | --- | --- | --- |
| Simons  2019  the Netherlands  Planned secondary analysis of Liem 2013 (ProTWIN trial) | Funding reported | Liem:  September 2009 to  March 2012 | Twins  Triplets | Twins (MC) 24.4%  Triplets  2.7% | CL measured  at 16-22 w  I: 43.6  (8.1)  C: 44.2 (8.5) | GA between 16 -20 w | I: Pessary (Arabin)  C: No pessary | None | I: 140  C: 118  Eligible for follow up:  Unselected: I: 392/781  C: 395/788  Approached  I: 311/621  C: 268/531 | I: 32  (29-36)  N=140  C: 33  (30-37)  N=118  median (IQR) | Women  I: 140  C: 118  Children  I: 281  C: 233 | Long-term child outcome: 4-year follow  ASQ  SDQ  Physical problem  Abnormal child outcome |
| Van’t Hooft  2018  the Netherlands  Planned secondary analysis of Liem 2013  (ProTWIN trial) | ProTWIN  Kids  Funding reported | Liem:  September 2009 to  March 2012 | Twins  Triplets | Twins (MC) 27%  Triplets  1.1% | CL<38 mm | GA between 16 -20 w | I: Pessary (Arabin)  C: Standard care |  | I: 58  C: 31 | I: 32  (29-36)  C: 30  (28-34)  median (IQR) | Surving children  241  Response rate 83% n=200 children  I: 120  C: 80 | Long term child outcome: 3-year follow-up |

| Acetylsalicylic acid | | | | | | | | | | | | |
| --- | --- | --- | --- | --- | --- | --- | --- | --- | --- | --- | --- | --- |
| Landman  2022  The Netherlands  (34 centers) | NL5553  Funding reported | May 2016 to June 2019 | Singleton | History of sPTB | NR | GA between 8-16 w | I: ASA 80 mg/d oral  C: Placebo  Until 36+0 w or delivery | Proges-terone, cerclage, pessary at physicians  discretion | 406 women Randomized  (19 excluded)  I: 194  C: 193 | I: 32.8  (3.9)  C: 32.3 (3.6) | I: 194  C: 193 | PTB <37 w (PO)  sPTB <37 w  PTB, sPTB <34 w  PTB, sPTB <28 w  GA at delivery  PNM  Neonatal morbidity  NICU admission  Maternal mortality and  morbidity |
| Other combinations of treatment | | | | | | | | | | | | |
| Cruz-Melguizo 2018  Spain  (27 centers) | NCT  01643980  Funding reported | August 2012 to April 2016 | Singleton | Short CL <25 mm  (women with cervical surgery and ≥3 previous PTBs were excluded) | I: 20.8  (4.2)  C: 20.9 (4.1) | GA between 20-23 w | I: Cervical pessary  C: 200 mg prog vag/d  Randomized 1:1  Until 36+6 | None | 254 women Randomized  (11 excluded)  I: 125  C: 118 | I: 32.5  (5.3)  C: 33.1 (5.5) | I: 125  C: 118 | sPTB <37w  sPTB < 34 w (PO)  sPTB <28 w  GA at delivery  LBW, VLBW  PNM  Neonatal and maternal morbidity  NICU admission |

| Dang  2019  Vietnam  (single center) | NCT  02623881  Funding reported | March 2016 to June 2017 | Twins | Short TVS-CL <38 mm | I: 30.9  (4.5)  C: 31.7 (4.1) | GA between  I: 17.5 w (1.5)  C: 18.0 w (1.8) | I: Pessary (Arabin)  C: 400 mg/day  vag prog/d (Cyklogest)  Interventions until 36+0 w | None | 1113 assessed  444  TVS CL< 38 mm  300 Randomized  I: 150  C: 150 | I: 31.7  (5.2)  C: 32.1 (4.9) | Women  I: 148  C: 149  Children  I: 296  C: 298 | PTB <34 w (PO)  PTB < 37 w  PTB < 28 w  GA at delivery  LBW, VLBW  PNM, NNM  Neonatal  morbidity  NICU admission  Maternal  mortality and  morbidity |
| --- | --- | --- | --- | --- | --- | --- | --- | --- | --- | --- | --- | --- |
| Keeler 2009a  USA  (single center) | Registra-tion NR  Funding reported | November 2003 to December 2006 | Singleton | TVS CL ≤25 mm in women with risk factors for PTB (history of sPTB, 2^nd^  trimester pregnancy loss, previous cervical surgery or, uterine anomaly) | I: 16.8  (5.1)  C: 14.5 (6.6) | GA between 16-24 w | I: Cerclage  (MacDonald)  C: 250 mg  17-OHPC im/w | Indomethacin, antibiotics before randomization | I: 42  C: 37 | I: 27.6 (6.58)  C: 29.6 (7.15) | I: 42  C: 37 | PTB, sPTB <37 w  PTB <35 w  sPTB <35 w (PO)  PTB, sPTB <32 w  PTB, sPTB <28 w  GA at delivery  PNM  Neonatal and  maternal  morbidity |

17-OHPC; 17-α-hydroxyprogesterone caproate, AMAG; Advanced Magnetics Inc , ART; assisted reproductive technology, ASA; acetylsalicylic acid, ASQ Age and Stages Questionnaire, BPD; bronchopulmary dysplasia, C; control, CBCL; Child Behavior Checklist, CDI; child development inventory score categorization, CHI; Community Health Index, CIPRACT; Cervical incompetence prevention randomized cerclage trial, CL; cervical length, CRD; Center for Reviews and Dissemination, DCHIA; diamniotic, DC; dichorionic, DCDA; dichorionic diamniotic, EPPIC; Evaluating Progestogens for Preventing Preterm birth International Collaborative, EudraCT; European Union Drug Regulating Authorities Clinical Trials Database, FFN; fetal fibronectin, GA; gestational age, GDM; gestational diabetes mellitus, HIV; human immunodeficiency virus, I; intervention, im; intramuscular, IPD MA; Individual Participant Data Meta-Analysis, IPOP; The Improving Pregnancy Outcomes with Progesterone, IRCT; Iranian Clinical Trial registry, ISRCTN; International Standard Randomized Controlled Trial Number, IUFD; intrauterine foster death, IQR; interquartile range, IV; intra venous, LBW; low birth weight (<2500g), LGT; lower genital tract, LGTI; lower tract genital infection, MC; monochorionic, MCDA; monochorionic diamniotic, MFMU; Maternal-Fetal Medicine Units, MRC/RCOG; Medical Research Council/Royal College of Obstetrics and Gynaecology, n; number, neg; negative, NICHD; National Institute of Child Health and Human Development, NICU; neonatal intensive care unit, NCT; National Clinical Trial, NNM; neonatal mortality, NR; not reported, NTR; the Netherlands trial registration, OPPTIMUM; dOes Progesterone Prophylaxis To prevent preterm labour IMprove oUtcoMe, PCORI; Patient-Centered Outcomes Research Institute, PECEP; Pesario Cervical para Evitar Prematuridad, PECEP-twins; Pesario Cervical para Evitar Prematuridad-twins, PNM; perinatal mortality, PO; primary outcome, PoPPS; Prevention of preterm birth with pessary in singletons, PoPPT; Prevention of Preterm birth with Pessary in Twins, pos; positive, PPA; Public and Professional Abstract, PREGNANT; Vaginal Progesterone Bioadhesive Gel (Prochieve)® Extending Gestation A New Therapy, PTB; preterm birth, PTD; preterm delivery, PTL; preterm labor, PPROM; preterm prelabor rupture of membranes, Prog; progesterone, PROLONG; Progestin's Role in Optimizing Neonatal Gestation, RCT; Randomized controlled trial, ReBec; Brazilian Clinical Trial Registry, SD; standard deviation, SDQ; Strength and Difficulties Questionnaire, sPTB spontaneous preterm birth, SR; systematic review, STOPPIT; STudy Of Progesterone for the Prevention of Preterm Birth In Twins, TCTA; trichorionic triamniotic, TRIPLE P; Preventing preterm birth with progesterone: costs and effects of screening low risk women with a singleton pregnancy for short cervical length, the Triple P study, TROPICAL; TRial Of Progesterone vaginal tab In prevention of preterm delivery evaluated by CervicAl Length, TVS; transvaginal scan, UK; United Kingdom, UMIN; University Hospital Medical Information Network, USA; United States of America, US NIH; the United States National Institutes of Health, Vag; vaginal, VLBW; very low birth weight <1500g.
