# Supplementary material for: Progesterone, cerclage, pessary, or acetylsalicylic acid for prevention of preterm birth in singleton and multifetal pregnancies – A systematic review and meta-analyses
Source: Front Med (Lausanne). 2023 Feb 28;10:1111315. doi: 10.3389/fmed.2023.1111315 (PMC10015499; doi:10.3389/fmed.2023.1111315)
Supplement: Supplementary file 1 [file Data_Sheet_1.zip › Data Sheet 1_corrected/Appendix 3 Excluded studies with reasons.docx]

**Progesterone, cerclage, pessary, or acetylsalicylic acid for prevention of preterm birth in singleton and multifetal pregnancies**

**Appendix 3**

**Table of contents**

[Excluded studies 2](#_Toc119582288)

[Table 1 Reason for exclusion 17](#_Toc119582289)

# Excluded studies

Abdel Wahab AS, Abdelmonaem MI, Mahmoud WM, Mansour AE. A randomized controlled trial of two-doses of vaginal progesterone 400 vs. 200 mg for prevention of preterm labor in twin gestations. Journal of Perinatal Medicine. 2021;14:14. doi: <https://dx.doi.org/10.1515/jpm-2021-0131>.

Abdel-Aleem H, Shaaban OM, Abdel-Aleem MA. Cervical pessary for preventing preterm birth. Cochrane Database of Systematic Reviews. 2013(5):CD007873. doi: <https://dx.doi.org/10.1002/14651858.CD007873.pub3>.

Abdi N, Rozrokh A, Alavi A, Zare S, Vafaei H, Asadi N, et al. The effect of aspirin on preeclampsia, intrauterine growth restriction and preterm delivery among healthy pregnancies with a history of preeclampsia. Journal of the Chinese Medical Association: JCMA. 2020;83(9):852-7. doi: <https://dx.doi.org/10.1097/JCMA.0000000000000400>.

Abramovici A, Jauk V, Wetta L, Cantu J, Edwards R, Biggio J, et al. Low-dose aspirin, smoking status, and the risk of spontaneous preterm birth. American Journal of Perinatology. 2015;32(5):445-50. doi: <https://dx.doi.org/10.1055/s-0034-1390352>.

Agra IKR, Carvalho MHB, Hernandez WR, Francisco RPV, Zugaib M, Brizot ML. The effect of prenatal vaginal progesterone on cervical length in nonselected twin pregnancies. Journal of Maternal-Fetal & Neonatal Medicine. 2019;32(8):1245-9. doi: <https://dx.doi.org/10.1080/14767058.2017.1403577>.

Ahn KH, Bae NY, Hong SC, Lee JS, Lee EH, Jee HJ, et al. The safety of progestogen in the prevention of preterm birth: meta-analysis of neonatal mortality. Journal of Perinatal Medicine. 2017;45(1):11-20. doi: <https://dx.doi.org/10.1515/jpm-2015-0317>.

Allshouse AA, Jessel RH, Heyborne KD. The impact of low-dose aspirin on preterm birth: secondary analysis of a randomized controlled trial. Journal of Perinatology. 2016;36(6):427-31. doi: <https://dx.doi.org/10.1038/jp.2016.3>.

Almutairi AR, Aljohani HI, Al-Fadel NS. 17-Alpha-Hydroxyprogesterone vs. Placebo for Preventing of Recurrent Preterm Birth: A Systematic Review and Meta-Analysis of Randomized Trials. Front Med (Lausanne). 2021;8:764855. doi: <https://doi.org/10.3389/fmed.2021.764855>.

Althuisius S, Dekker G, Hummel P, Bekedam D, Kuik D, van Geijn H. Cervical Incompetence Prevention Randomized Cerclage Trial (CIPRACT): effect of therapeutic cerclage with bed rest vs. bed rest only on cervical length. Ultrasound in Obstetrics & Gynecology. 2002;20(2):163-7. doi: <https://doi.org/10.1046/j.1469-0705.2002.00770.x>.

Althuisius SM, Dekker GA, Van Geijn HP, Bekedam DJ, Hummel P. Cervical Incompetence Prevention Randomized Cerclage Trial (CIPRACT): Study design and preliminary results. American Journal of Obstetrics and Gynecology. 2000;183(4):823-9. doi: <http://dx.doi.org/10.1067/mob.2000.108874>.

Andrikopoulou M, Purisch SE, Handal-Orefice R, Gyamfi-Bannerman C. Low-dose aspirin is associated with reduced spontaneous preterm birth in nulliparous women. American Journal of Obstetrics & Gynecology. 2018;219(4):399.e1-.e6. doi: <https://dx.doi.org/10.1016/j.ajog.2018.06.011>.

Atallah AN. ECPPA: Randomized trial of low dose aspirin for the prevention of maternal and fetal complications in high risk pregnant women. British Journal of Obstetrics and Gynaecology. 1996;103(1):39-47. doi: <https://dx.doi.org/10.1111/j.1471-0528.1996.tb09513.x>.

Ayala DE, Ucieda R, Hermida RC. Chronotherapy with low-dose aspirin for prevention of complications in pregnancy. Chronobiology International. 2013;30(1-2):260-79. doi: <https://dx.doi.org/10.3109/07420528.2012.717455>.

Bachmann LM, Coomarasamy A, Honest H, Khan KS. Elective cervical cerclage for prevention of preterm birth: a systematic review. Acta Obstetricia et Gynecologica Scandinavica. 2003;82(5):398-404. doi: <https://dx.doi.org/10.1080/j.1600-0412.2003.00081.x>.

Bakhti A, Vaiman D. Prevention of gravidic endothelial hypertension by aspirin treatment administered from the 8th week of gestation. Hypertension research - clinical & experimental. 2011;34(10):1116‐20. doi: <https://doi.org/10.1038/hr.2011.111>.

Baradwan S, Abdulghani SH, Abuzaid M, Khadawardi K, Alshahrani MS, Al-Matary A, et al. 17-alpha hydroxyprogesterone caproate for the prevention of recurrent preterm birth among singleton pregnant women with a prior history of preterm birth: a systematic review and meta-analysis of six randomized controlled trials. Obstet Gynecol Sci. 2021;64(6):484-95. doi: <https://dx.doi.org/10.5468/ogs.21264>.

Belej-Rak T, Okun N, Windrim R, Ross S, Hannah ME. Effectiveness of cervical cerclage for a sonographically shortened cervix: a systematic review and meta-analysis. American Journal of Obstetrics & Gynecology. 2003;189(6):1679-87. doi: <https://doi.org/10.1016/S0002-9378(03)00871-8>.

Berghella V, Ciardulli A, Rust OA, To M, Otsuki K, Althuisius S, et al. Cerclage for sonographic short cervix in singleton gestations without prior spontaneous preterm birth: systematic review and meta-analysis of randomized controlled trials using individual patient-level data. Ultrasound in Obstetrics & Gynecology. 2017b;50(5):569-77. doi: <https://dx.doi.org/10.1002/uog.17457>.

Berghella V, Figueroa D, Szychowski JM, Owen J, Hankins GD, Iams JD, et al. 17-alpha-hydroxyprogesterone caproate for the prevention of preterm birth in women with prior preterm birth and a short cervical length. American Journal of Obstetrics & Gynecology. 2010a;202(4):351.e-6. doi: <https://dx.doi.org/10.1016/j.ajog.2010.02.019>.

Berghella V, Keeler SM, To MS, Althuisius SM, Rust OA. Effectiveness of cerclage according to severity of cervical length shortening: a meta-analysis. Ultrasound in Obstetrics & Gynecology. 2010b;35(4):468-73. doi: <https://dx.doi.org/10.1002/uog.7547>.

Berghella V, Rafael TJ, Szychowski JM, Rust OA, Owen J. Cerclage for short cervix on ultrasonography in women with singleton gestations and previous preterm birth: a meta-analysis. Obstetrics & Gynecology. 2011;117(3):663-71. doi: <https://dx.doi.org/10.1097/AOG.0b013e31820ca847>.

Beroyz G, Casale R, Farreiros A, Palermo M, Margulies M, Voto L, et al. CLASP: A randomized trial of low-dose aspirin for the prevention and treatment of pre-eclampsia among 9364 pregnant women. Lancet. 1994;343(8898):619-29. doi: <http://dx.doi.org/10.1016/S0140-6736%2894%2992633-6>.

Boelig RC, Della Corte L, Ashoush S, McKenna D, Saccone G, Rajaram S, et al. Oral progesterone for the prevention of recurrent preterm birth: systematic review and metaanalysis. American Journal of Obstetrics & Gynecology MFM. 2019;1(1):50-62. doi: <https://dx.doi.org/10.1016/j.ajogmf.2019.03.001>.

Buchbinder A, Sibai BM, Caritis S, Macpherson C, Hauth J, Lindheimer MD, et al. Adverse perinatal outcomes are significantly higher in severe gestational hypertension than in mild preeclampsia. American Journal of Obstetrics & Gynecology. 2002;186(1):66-71. doi: <https://dx.doi.org/10.1067/mob.2002.120080>.

Bujold E, Roberge S, Lacasse Y, Bureau M, Audibert F, Marcoux S, et al. Prevention of preeclampsia and intrauterine growth restriction with aspirin started in early pregnancy: a meta-analysis. Obstetrics & Gynecology. 2010;116(2 Pt 1):402-14. doi: <https://dx.doi.org/10.1097/AOG.0b013e3181e9322a>.

Caritis S, Sibai B, Hauth J, Lindheimer MD, Klebanoff M, Thom E, et al. Low-dose aspirin to prevent preeclampsia in women at high risk. New England Journal of Medicine. 1998;338(11):701-5. doi: <http://dx.doi.org/10.1056/NEJM199803123381101>.

Caritis SN, Simhan HN, Zhao Y, Rouse DJ, Peaceman AM, Sciscione A, et al. Relationship between 17-hydroxyprogesterone caproate concentrations and gestational age at delivery in twin gestation. American Journal of Obstetrics & Gynecology. 2012;207(5):396.e1-8. doi: <https://dx.doi.org/10.1016/j.ajog.2012.08.001>.

Caritis SN, Venkataramanan R, Thom E, Harper M, Klebanoff MA, Sorokin Y, et al. Relationship between 17-alpha hydroxyprogesterone caproate concentration and spontaneous preterm birth. American Journal of Obstetrics & Gynecology. 2014;210(2):128.e1-6. doi: <https://dx.doi.org/10.1016/j.ajog.2013.10.008>.

Chaemsaithong P, Cuenca-Gomez D, Plana MN, Gil MM, Poon LC. Does low-dose aspirin initiated before 11 weeks' gestation reduce the rate of preeclampsia? American Journal of Obstetrics & Gynecology. 2020;222(5):437-50. doi: <https://dx.doi.org/10.1016/j.ajog.2019.08.047>.

Chaman-Ara K, Bahrami MA, Bahrami E, Bahrami S, Bahrami MN, Moosazadeh M, et al. Efficacy of progesterone therapy in the prevention of preterm labor in women with mixed risk-factors: A systematic review and meta-analysis of randomized clinical trials. Erciyes Tip Dergisi. 2016;38(2):48-52. doi: <http://dx.doi.org/10.5152/etd.2016.0034>.

Combs CA, Schuit E, Caritis SN, Lim AC, Garite TJ, Maurel K, et al. 17-Hydroxyprogesterone caproate in triplet pregnancy: an individual patient data meta-analysis. BJOG: An International Journal of Obstetrics & Gynaecology. 2016;123(5):682-90. doi: <https://dx.doi.org/10.1111/1471-0528.13779>.

Conde-Agudelo A, Romero R, Da Fonseca E, O'Brien JM, Cetingoz E, Creasy GW, et al. Vaginal progesterone is as effective as cervical cerclage to prevent preterm birth in women with a singleton gestation, previous spontaneous preterm birth, and a short cervix: updated indirect comparison meta-analysis. American Journal of Obstetrics & Gynecology. 2018;219(1):10-25. doi: <https://dx.doi.org/10.1016/j.ajog.2018.03.028>.

Conde-Agudelo A, Romero R, Nicolaides KH. Cervical pessary to prevent preterm birth in asymptomatic high-risk women: a systematic review and meta-analysis. American Journal of Obstetrics & Gynecology. 2020;223(1):42-65.e42. doi: <https://dx.doi.org/10.1016/j.ajog.2019.12.266>.

Connealy BD, Carreno CA, Kase BA, Hart LA, Blackwell SC, Sibai BM. A history of prior preeclampsia as a risk factor for preterm birth. American Journal of Perinatology. 2014;31(6):483-8. doi: <http://dx.doi.org/10.1055/s-0033-1353439>.

Coomarasamy A, Honest H, Papaioannou S, Gee H, Khan KS. Aspirin for prevention of preeclampsia in women with historical risk factors: a systematic review. Obstetrics & Gynecology. 2003;101(6):1319-32. doi: <https://doi.org/10.1016/S0029-7844(03)00169-8>.

Coomarasamy A, Thangaratinam S, Gee H, Khan KS. Progesterone for the prevention of preterm birth: a critical evaluation of evidence. European Journal of Obstetrics, Gynecology, & Reproductive Biology. 2006;129(2):111-8. doi: <https://doi.org/10.1016/j.ejogrb.2006.05.013>.

Corrado F, Dugo C, Cannata ML, Di Bartolo M, Scilipoti A, Carlo Stella N. A randomized trial of progesterone prophylaxis after midtrimester amniocentesis. European Journal of Obstetrics, Gynecology, & Reproductive Biology. 2002;100(2):196-8. doi: <https://dx.doi.org/10.1016/s0301-2115(01)00418-3>.

Correa TD, Amorim EG, Tomazelli JAG, Correa MDJ. Use of the Pessary in the Prevention of Preterm Delivery. Revista Brasileira de Ginecologia e Obstetricia. 2019;41(1):53-8. doi: <https://dx.doi.org/10.1055/s-0038-1676511>.

Cui Y, Zhu B, Zheng F. Low-dose aspirin at ≤16 weeks of gestation for preventing preeclampsia and its maternal and neonatal adverse outcomes: A systematic review and meta-analysis. Exp Ther Med. 2018;15(5):4361-9. doi: <https://dx.doi.org/10.3892/etm.2018.5972>.

D'Antonio F, Berghella V, Di Mascio D, Saccone G, Sileo F, Flacco ME, et al. Role of progesterone, cerclage and pessary in preventing preterm birth in twin pregnancies: A systematic review and network meta-analysis. European Journal of Obstetrics, Gynecology, & Reproductive Biology. 2021;261:166-77. doi: <https://dx.doi.org/10.1016/j.ejogrb.2021.04.023>.

de Jong PG, Kaandorp S, Di Nisio M, Goddijn M, Middeldorp S. Aspirin and/or heparin for women with unexplained recurrent miscarriage with or without inherited thrombophilia. Cochrane Database of Systematic Reviews. 2014(7):CD004734. doi: <https://dx.doi.org/10.1002/14651858.CD004734.pub4>.

DeFranco EA, O'Brien JM, Adair CD, Lewis DF, Hall DR, Fusey S, et al. Vaginal progesterone is associated with a decrease in risk for early preterm birth and improved neonatal outcome in women with a short cervix: a secondary analysis from a randomized, double-blind, placebo-controlled trial. Ultrasound in obstetrics & gynecology. 2007;30(5):697‐705. doi: <https://dx.doi.org/10.1002/uog.5159>.

Dodd JM, Crowther CA, Cincotta R, Flenady V, Robinson JS. Progesterone supplementation for preventing preterm birth: a systematic review and meta-analysis. Acta Obstetricia et Gynecologica Scandinavica. 2005;84(6):526-33. doi: <https://doi.org/10.1080/j.0001-6349.2005.00835.x>.

Dodd JM, Flenady V, Cincotta R, Crowther CA. Prenatal administration of progesterone for preventing preterm birth. Cochrane Database of Systematic Reviews. 2006(1):CD004947. doi: <https://doi.org/10.1002/14651858.CD004947.pub3>.

Dodd JM, Flenady VJ, Cincotta R, Crowther CA. Progesterone for the prevention of preterm birth: a systematic review. Obstetrics & Gynecology. 2008;112(1):127-34. doi: <https://dx.doi.org/10.1097/AOG.0b013e31817d0262>.

Dodd JM, Grivell RM, Cm OB, Dowswell T, Deussen AR. Prenatal administration of progestogens for preventing spontaneous preterm birth in women with a multiple pregnancy. Cochrane Database of Systematic Reviews. 2019;11(11):20. doi: <https://dx.doi.org/10.1002/14651858.CD012024.pub3>.

Dodd JM, Jones L, Flenady V, Cincotta R, Crowther CA. Prenatal administration of progesterone for preventing preterm birth in women considered to be at risk of preterm birth. Cochrane Database of Systematic Reviews. 2013(7):CD004947. doi: <https://dx.doi.org/10.1002/14651858.CD004947.pub3>.

Drakeley AJ, Roberts D, Alfirevic Z. Cervical cerclage for prevention of preterm delivery: meta-analysis of randomized trials. Obstetrics & Gynecology. 2003a;102(3):621-7. doi: <https://doi.org/10.1016/S0029-7844(03)00673-2>.

Drakeley AJ, Roberts D, Alfirevic Z. Cervical stitch (cerclage) for preventing pregnancy loss in women. Cochrane Database of Systematic Reviews. 2003b(1):CD003253. doi: <https://doi.org/10.1002/14651858.CD003253>.

Duley L, Meher S, Hunter KE, Seidler AL, Askie LM. Antiplatelet agents for preventing pre-eclampsia and its complications. Cochrane Database of Systematic Reviews. 2019;10(10):30. doi: <https://dx.doi.org/10.1002/14651858.CD004659.pub3>.

Durnwald CP, Momirova V, Rouse DJ, Caritis SN, Peaceman AM, Sciscione A, et al. Second trimester cervical length and risk of preterm birth in women with twin gestations treated with 17-alpha hydroxyprogesterone caproate. Journal of Maternal-Fetal & Neonatal Medicine. 2010;23(12):1360-4. doi: <https://dx.doi.org/10.3109/14767051003702786>.

Ebrashy A, Ibrahim M, Marzook A, Yousef D. Usefulness of aspirin therapy in high-risk pregnant women with abnormal uterine artery Doppler ultrasound at 14-16 weeks pregnancy: randomized controlled clinical trial. Croatian Medical Journal. 2005;46(5):826-31. doi:

Eddama O, Petrou S, Regier D, Norrie J, MacLennan G, Mackenzie F, et al. Study of progesterone for the prevention of preterm birth in twins (STOPPIT): findings from a trial-based cost-effectiveness analysis. International Journal of Technology Assessment in Health Care. 2010;26(2):141-8. doi: <https://dx.doi.org/10.1017/S0266462310000036>.

Eke AC, Sheffield J, Graham EM. Adjuvant 17-hydroxyprogesterone caproate in women with history-indicated cerclage: A systematic review and meta-analysis. Acta Obstetricia et Gynecologica Scandinavica. 2019a;98(2):139-53. doi: <https://dx.doi.org/10.1111/aogs.13488>.

Eke AC, Sheffield J, Graham EM. 17alpha-Hydroxyprogesterone Caproate and the Risk of Glucose Intolerance in Pregnancy: A Systematic Review and Meta-analysis. Obstetrics & Gynecology. 2019b;133(3):468-75. doi: <https://dx.doi.org/10.1097/AOG.0000000000003115>.

El-Refaie W, Abdelhafez MS, Badawy A. Vaginal progesterone for prevention of preterm labor in asymptomatic twin pregnancies with sonographic short cervix: a randomized clinical trial of efficacy and safety. Archives of Gynecology & Obstetrics. 2016;293(1):61-7. doi: <https://dx.doi.org/10.1007/s00404-015-3767-1>.

Farinelli CK, Wing DA, Szychowski JM, Owen J, Hankins G, Iams JD, et al. Association between body mass index and pregnancy outcome in a randomized trial of cerclage for short cervix. Ultrasound in Obstetrics & Gynecology. 2012;40(6):669-73. doi: <https://dx.doi.org/10.1002/UOG.11170>.

Fernandez-Macias R, Martinez-Portilla RJ, Cerrillos L, Figueras F, Palacio M. A systematic review and meta-analysis of randomized controlled trials comparing 17-alpha-hydroxyprogesterone caproate versus placebo for the prevention of recurrent preterm birth. International Journal of Gynaecology & Obstetrics. 2019;147(2):156-64. doi: <https://dx.doi.org/10.1002/ijgo.12940>.

Fratto VM, Ananth CV, Gyamfi-Bannerman C. Late preterm neonatal morbidity in hypertensive versus normotensive women. Hypertension in Pregnancy. 2016;35(2):242-9. doi: <https://dx.doi.org/10.3109/10641955.2016.1139720>.

Ghazanfarpour M, Sathyapalan T, Banach M, Jamialahmadi T, Sahebkar A. Prophylactic aspirin for preventing pre-eclampsia and its complications: An overview of meta-analyses. Drug Discovery Today. 2020;25(8):1487-501. doi: <https://dx.doi.org/10.1016/j.drudis.2020.05.011>.

Goldstein P, Berrier J, Rosen S, Sacks HS, Chalmers TC. A meta-analysis of randomized control trials of progestational agents in pregnancy. British Journal of Obstetrics & Gynaecology. 1989;96(3):265-74. doi: <https://doi.org/10.1111/j.1471-0528.1989.tb02385.x>.

Grabovac M, Lewis-Mikhael AM, McDonald SD. Interventions to Try to Prevent Preterm Birth in Women With a History of Conization: A Systematic Review and Meta-analyses. Journal of Obstetrics & Gynaecology Canada: JOGC. 2019;41(1):76-88.e77. doi: <https://dx.doi.org/10.1016/j.jogc.2018.04.026>.

Groeneveld E, Lambers MJ, Lambalk CB, Broeze KA, Haapsamo M, de Sutter P, et al. Preconceptional low-dose aspirin for the prevention of hypertensive pregnancy complications and preterm delivery after IVF: a meta-analysis with individual patient data. Human Reproduction. 2013;28(6):1480-8. doi: <https://dx.doi.org/10.1093/humrep/det022>.

Gu W, Lin J, Hou YY, Lin N, Song MF, Zeng WJ, et al. Effects of low-dose aspirin on the prevention of preeclampsia and pregnancy outcomes: A randomized controlled trial from Shanghai, China. European Journal of Obstetrics, Gynecology, & Reproductive Biology. 2020;248:156-63. doi: <https://dx.doi.org/10.1016/j.ejogrb.2020.03.038>.

Haas DM, Hathaway TJ, Ramsey PS. Progestogen for preventing miscarriage in women with recurrent miscarriage of unclear etiology. Cochrane Database of Systematic Reviews. 2019;11(11):20. doi: <https://dx.doi.org/10.1002/14651858.CD003511.pub5>.

Hajizadeh N, Saharkhiz N, Hosseini S, Arabzadeh B. Comparison of cerclage and pessary in prevention of preterm birth in twin pregnancies. Med J Islam Repub Iran. 2020;34:74. doi: <https://dx.doi.org/10.34171/mjiri.34.74>.

Hamid R, Robson M, Pearce JM. Low dose aspirin in women with raised maternal serum alpha-fetoprotein and abnormal Doppler waveform patterns from the uteroplacental circulation. British Journal of Obstetrics & Gynaecology. 1994;101(6):481-4. doi: <https://dx.doi.org/10.1111/j.1471-0528.1994.tb13145.x>.

Hartikainen-Sorri AL, Kauppila A, Tuimala R. Inefficacy of 17 alpha-hydroxyprogesterone caproate in the prevention of prematurity in twin pregnancy. Obstetrics & Gynecology. 1980;56(6):692-5. doi:

Henderson JT, Whitlock EP, O'Conner E, Senger CA, Thompson JH, Rowland MG. Low-Dose Aspirin for the Prevention of Morbidity and Mortality From Preeclampsia: A Systematic Evidence Review for the U.S. Preventive Services Task Force. Agency for Healthcare Research and Quality. 2014a:04. doi: <https://doi.org/10.7326/M13-2844>.

Henderson JT, Whitlock EP, O'Connor E, Senger CA, Thompson JH, Rowland MG. Low-dose aspirin for prevention of morbidity and mortality from preeclampsia: a systematic evidence review for the U.S. Preventive Services Task Force. Annals of Internal Medicine. 2014b;160(10):695-703. doi: <https://dx.doi.org/10.7326/M13-2844>.

Heyborne KD, Allshouse AA. Smoking, 17 Alpha-Hydroxyprogesterone Caproate, and Preterm Birth. American Journal of Perinatology. 2016;33(12):1191-7. doi: <https://dx.doi.org/10.1055/s-0036-1586119>.

Heyborne KD, Allshouse AA, Carey JC. Does 17-alpha hydroxyprogesterone caproate prevent recurrent preterm birth in obese women? American Journal of Obstetrics & Gynecology. 2015;213(6):844.e1-6. doi: <https://dx.doi.org/10.1016/j.ajog.2015.08.014>.

Hezelgrave NL, Watson HA, Ridout A, Diab F, Seed PT, Chin-Smith E, et al. Rationale and design of SuPPoRT: a multi-centre randomized controlled trial to compare three treatments: cervical cerclage, cervical pessary and vaginal progesterone, for the prevention of preterm birth in women who develop a short cervix. BMC Pregnancy & Childbirth. 2016;16(1):358. doi: <https://dx.doi.org/10.1186/s12884-016-1148-9>.

Hoffman MK, Goudar SS, Kodkany BS, Metgud M, Somannavar M, Okitawutshu J, et al. Low-dose aspirin for the prevention of preterm delivery in nulliparous women with a singleton pregnancy (ASPIRIN): a randomized, double-blind, placebo-controlled trial. Lancet. 2020;395(10220):285-93. doi: <https://dx.doi.org/10.1016/S0140-6736(19)32973-3>.

Huai J, Lin L, Juan J, Chen J, Li B, Zhu Y, et al. Preventive effect of aspirin on preeclampsia in high-risk pregnant women with stage 1 hypertension. Journal of Clinical Hypertension. 2021;05:05. doi: <https://dx.doi.org/10.1111/jch.14149>.

Jarde A, Lewis-Mikhael AM, Dodd JM, Barrett J, Saito S, Beyene J, et al. The More, the Better? Combining Interventions to Prevent Preterm Birth in Women at Risk: a Systematic Review and Meta-Analysis. Journal of Obstetrics & Gynaecology Canada: JOGC. 2017a;39(12):1192-202. doi: <https://dx.doi.org/10.1016/j.jogc.2017.07.007>.

Jarde A, Lutsiv O, Beyene J, McDonald SD. Vaginal progesterone, oral progesterone, 17-OHPC, cerclage, and pessary for preventing preterm birth in at-risk singleton pregnancies: an updated systematic review and network meta-analysis. BJOG: An International Journal of Obstetrics & Gynaecology. 2019;126(5):556-67. doi: <https://dx.doi.org/10.1111/1471-0528.15566>.

Jarde A, Lutsiv O, Park CK, Barrett J, Beyene J, Saito S, et al. Preterm birth prevention in twin pregnancies with progesterone, pessary, or cerclage: a systematic review and meta-analysis. BJOG: An International Journal of Obstetrics & Gynaecology. 2017b;124(8):1163-73. doi: <https://dx.doi.org/10.1111/1471-0528.14513>.

Jarde A, Lutsiv O, Park CK, Beyene J, Dodd JM, Barrett J, et al. Effectiveness of progesterone, cerclage and pessary for preventing preterm birth in singleton pregnancies: a systematic review and network meta-analysis. BJOG: An International Journal of Obstetrics & Gynaecology. 2017c;124(8):1176-89. doi: <https://dx.doi.org/10.1111/1471-0528.14624>.

Jessani S, Saleem S, Hoffman MK, Goudar SS, Derman RJ, Moore JL, et al. Association of haemoglobin levels in the first trimester and at 26-30 weeks with fetal and neonatal outcomes: a secondary analysis of the Global Network for Women's and Children's Health's ASPIRIN Trial. BJOG: An International Journal of Obstetrics & Gynaecology. 2021;128(9):1487-96. doi: <https://dx.doi.org/10.1111/1471-0528.16676>.

Jin XH, Li D, Huang LL. Cervical Pessary for Prevention of Preterm Birth: A Meta-Analysis. Scientific Reports. 2017;7:42560. doi: <https://dx.doi.org/10.1038/srep42560>.

Jin Z, Chen L, Qiao D, Tiwari A, Jaunky CD, Sun B, et al. Cervical pessary for preventing preterm birth: a meta-analysis. Journal of Maternal-Fetal & Neonatal Medicine. 2019;32(7):1148-54. doi: <https://dx.doi.org/10.1080/14767058.2017.1401998>.

Jorgensen AL, Alfirevic Z, Tudur Smith C, Williamson PR, cerclage IPDMaG. Cervical stitch (cerclage) for preventing pregnancy loss: individual patient data meta-analysis. BJOG: An International Journal of Obstetrics & Gynaecology. 2007;114(12):1460-76. doi: 10.1111/j.1471-0528.2007.01515.x.

Keeler SM, Roman AS, Coletta JM, Kiefer DG, Feuerman M, Rust OA. Fetal fibronectin testing in patients with short cervix in the midtrimester: can it identify optimal candidates for ultrasound-indicated cerclage? American Journal of Obstetrics & Gynecology. 2009b;200(2):158.e1-6. doi: <https://dx.doi.org/10.1016/j.ajog.2008.08.050>.

Khazardoost S, Mousavi S, Borna S, Hantoushzadeh S, Alavi A, Khezerlou N. Effect of aspirin in prevention of adverse pregnancy outcome in women with elevated alpha-fetoprotein. Journal of Maternal-Fetal & Neonatal Medicine. 2014;27(6):561-5. doi: <https://dx.doi.org/10.3109/14767058.2013.822483>.

Kozer E, Costei AM, Boskovic R, Nulman I, Nikfar S, Koren G. Effects of aspirin consumption during pregnancy on pregnancy outcomes: meta-analysis. Birth Defects Research Part B, Developmental and Reproductive Toxicology. 2003;68(1):70-84. doi: 10.1002/bdrb.10002.

Kumar A, Begum N, Prasad S, Aggarwal S, Sharma S. Oral dydrogesterone treatment during early pregnancy to prevent recurrent pregnancy loss and its role in modulation of cytokine production: A double-blind, randomized, parallel, placebo-controlled trial. Fertility and Sterility. 2014;102(5):1357-63. doi: <http://dx.doi.org/10.1016/j.fertnstert.2014.07.1251>.

Kumar N, Das V, Agarwal A, Pandey A, Agrawal S, Singh A. Pilot Interventional Study Comparing Fetomaternal Outcomes of 150 mg Versus 75 mg Aspirin Starting Between 11 and 14 Weeks of Pregnancy in Patients with High Risk of Preeclampsia: A Randomized Control Trial. J Obstet Gynaecol India. 2020;70(1):23-9. doi: 10.1007/s13224-019-01277-5.

Lambers MJ, Groeneveld E, Hoozemans DA, Schats R, Homburg R, Lambalk CB, et al. Lower incidence of hypertensive complications during pregnancy in patients treated with low-dose aspirin during in vitro fertilization and early pregnancy. Human Reproduction. 2009;24(10):2447-50. doi: <http://dx.doi.org/10.1093/humrep/dep245>.

Le KD, Nguyen LK, Nguyen LTM, Mol BWJ, Dang VQ. Cervical pessary vs vaginal progesterone for prevention of preterm birth in women with twin pregnancy and short cervix: economic analysis following randomized controlled trial. Ultrasound in Obstetrics & Gynecology. 2020;55(3):339-47. doi: <https://dx.doi.org/10.1002/uog.20848>.

Leslie GI, Gallery ED, Arnold JD, Ross MR, Gyory AZ. Neonatal outcome in a randomized, controlled trial of low-dose aspirin in high-risk pregnancies. Journal of Paediatrics & Child Health. 1995;31(6):549-52. doi: 10.1111/j.1440-1754.1995.tb00882.x.

Li C, Shen J, Hua K. Cerclage for women with twin pregnancies: a systematic review and metaanalysis. American Journal of Obstetrics & Gynecology. 2019;220(6):543-57.e1. doi: <https://dx.doi.org/10.1016/j.ajog.2018.11.1105>.

Liem SM, Schuit E, van Pampus MG, van Melick M, Monfrance M, Langenveld J, et al. Cervical pessaries to prevent preterm birth in women with a multiple pregnancy: a per-protocol analysis of a randomized clinical trial. Acta Obstetricia et Gynecologica Scandinavica. 2016;95(4):444-51. doi: <https://dx.doi.org/10.1111/aogs.12849>.

Liem SM, van Baaren GJ, Delemarre FM, Evers IM, Kleiverda G, van Loon AJ, et al. Economic analysis of use of pessary to prevent preterm birth in women with multiple pregnancy (ProTWIN trial). Ultrasound in Obstetrics & Gynecology. 2014;44(3):338-45. doi: <https://dx.doi.org/10.1002/uog.13432>.

Liem SM, van Pampus MG, Mol BW, Bekedam DJ. Cervical pessaries for the prevention of preterm birth: a systematic review. Obstet Gynecol Int. 2013b;2013:576723. doi: 10.1155/2013/576723.

Likis FE, Andrews JC, Woodworth AL, Velez Edwards DR, Jerome RN, Fonnesbeck CJ, et al. Agency for Healthcare Research and Quality. 09. doi: . Progestogens for Prevention of Preterm Birth [Internet]. Rockville (MD): Agency for Healthcare Research and Quality (US). 2012a; 2012 Sep. Report No.: 12-EHC105-EF. PMID: 23101048.

Likis FE, Edwards DR, Andrews JC, Woodworth AL, Jerome RN, Fonnesbeck CJ, et al. Progestogens for preterm birth prevention: a systematic review and meta-analysis. Obstetrics & Gynecology. 2012b;120(4):897-907. doi: 10.1097/AOG.0b013e3182699a15.

Liu J, Song G, Meng T, Zhao G. Vaginal progesterone combined with cervical pessary in preventing preterm birth: a meta-analysis. Journal of Maternal-Fetal & Neonatal Medicine. 2019:1-7. doi: <https://dx.doi.org/10.1080/14767058.2019.1677596>.

Liu XR, Luo X, Xiao XQ, Qi HB. Cervical cerclage for preventing preterm birth in twin pregnancies. A systematic review and meta-analysis. Saudi Medical Journal. 2013;34(6):632-8. doi:

Mackenzie R, Walker M, Armson A, Hannah ME. Progesterone for the prevention of preterm birth among women at increased risk: a systematic review and meta-analysis of randomized controlled trials. American Journal of Obstetrics & Gynecology. 2006;194(5):1234-42. doi: 10.1016/j.ajog.2005.06.049

Man R, Hodgetts Morton V, Devani P, Morris RK. Aspirin for preventing adverse outcomes in low risk nulliparous women with singleton pregnancies: A systematic review and meta-analysis. European Journal of Obstetrics, Gynecology, & Reproductive Biology. 2021;262:105-12. doi: <https://dx.doi.org/10.1016/j.ejogrb.2021.05.017>.

Mancuso MS, Szychowski JM, Owen J, Hankins G, Iams JD, Sheffield JS, et al. Cervical funneling: effect on gestational length and ultrasound-indicated cerclage in high-risk women. American Journal of Obstetrics & Gynecology. 2010;203(3):259.e1-5. doi: <https://dx.doi.org/10.1016/j.ajog.2010.07.002>.

Manuck TA, Lai Y, Meis PJ, Dombrowski MP, Sibai B, Spong CY, et al. Progesterone receptor polymorphisms and clinical response to 17-alpha-hydroxyprogesterone caproate. American Journal of Obstetrics & Gynecology. 2011;205(2):135.e1-9. doi: <https://dx.doi.org/10.1016/j.ajog.2011.03.048>.

Marat A, Ukybassova T, Gabdilashimova Z. The short cervix management as a prevention method of preterm birth. Journal of Clinical and Diagnostic Research. 2019;13(2):QC05-QC9. doi: <http://dx.doi.org/10.7860/JCDR/2019/36398.12597>.

McNaughton MC, Chalmers IG, Chamberlain GVP, Dubowitz V, Dunn PM, Grand AM, et al. Interim report of the Medical Research Council/Royal College of Obstetricians and Gynaecologists multicentre randomized trial of cervical cerclage. British Journal of Obstetrics and Gynaecology. 1988;95(5):437-45. doi: 10.1111/j.1471-0528.1988.tb12794.x.

Meher S, Duley L. Progesterone for preventing pre-eclampsia and its complications. Cochrane Database of Systematic Reviews. 2006(4):CD006175. doi: 10.1002/14651858.CD006175.

Meher S, Duley L, Hunter K, Askie L. Antiplatelet therapy before or after 16 weeks' gestation for preventing preeclampsia: an individual participant data meta-analysis. American Journal of Obstetrics & Gynecology. 2017;216(2):121-8.e2. doi: <https://dx.doi.org/10.1016/j.ajog.2016.10.016>.

Meis PJ, Klebanoff M, Dombrowski MP, Sibai BM, Leindecker S, Moawad AH, et al. Does progesterone treatment influence risk factors for recurrent preterm delivery? Obstetrics & Gynecology. 2005;106(3):557-61. doi: 10.1097/01.AOG.0000174582.79364.a7.

Mendoza M, Goya M, Gascon A, Pratcorona L, Merced C, Rodo C, et al. Modification of cervical length after cervical pessary insertion: correlation weeks of gestation. Journal of Maternal-Fetal & Neonatal Medicine. 2017;30(13):1596-601. doi: <https://dx.doi.org/10.1080/14767058.2016.1216538>.

Mourad M, Too G, Gyamfi-Bannerman C, Zork N. Hypertensive disorders of pregnancy in twin gestations complicated by gestational diabetes*. Journal of Maternal Fetal and Neonatal Medicine. 2019. doi: <http://dx.doi.org/10.1080/14767058.2019.1614160>.

Mourad M, Too G, Gyamfi-Bannerman C, Zork N. Hypertensive disorders of pregnancy in twin gestations complicated by gestational diabetes*. Journal of Maternal-Fetal and Neonatal Medicine. 2021;34(5):720-4. doi: <https://dx.doi.org/10.1080/14767058.2019.1614160>.

Moussa HN, Wu ZH, Han Y, Pacheco LD, Blackwell SC, Sibai BM, et al. Customized versus Population Fetal Growth Norms and Adverse Outcomes Associated with Small for Gestational Age Infants in a High-Risk Cohort. American Journal of Perinatology. 2015;32(7):621-6. doi: <https://dx.doi.org/10.1055/s-0034-1390343>.

Odibo AO, Elkousy M, Ural SH, Macones GA. Prevention of preterm birth by cervical cerclage compared with expectant management: a systematic review. Obstetrical & Gynecological Survey. 2003;58(2):130-6. doi: 10.1097/01.OGX.0000047740.21512.FC.

Odibo AO, Goetzinger KR, Odibo L, Tuuli MG. Early prediction and aspirin for prevention of pre-eclampsia (EPAPP) study: a randomized controlled trial. Ultrasound in Obstetrics & Gynecology. 2015;46(4):414-8. doi: <https://dx.doi.org/10.1002/uog.14889>.

Perez-Lopez FR, Chedraui P, Perez-Roncero GR, Martinez-Dominguez SJ, Health O, Systematic Analyses P. Effectiveness of the cervical pessary for the prevention of preterm birth in singleton pregnancies with a short cervix: a meta-analysis of randomized trials. Archives of Gynecology & Obstetrics. 2019;299(5):1215-31. doi: <https://dx.doi.org/10.1007/s00404-019-05096-x>.

Phung J, Williams KP, McAullife L, Martin WN, Flint C, Andrew B, et al. Vaginal progesterone for prevention of preterm birth in asymptomatic high-risk women with a normal cervical length: a systematic review and meta-analysis. Journal of Maternal Fetal and Neonatal Medicine. 2021. doi: <http://dx.doi.org/10.1080/14767058.2021.1943657>.

Pizzi LT, Seligman NS, Baxter JK, Jutkowitz E, Berghella V. Cost and cost effectiveness of vaginal progesterone gel in reducing preterm birth: an economic analysis of the PREGNANT trial. Pharmacoeconomics. 2014;32(5):467-78. doi: <https://dx.doi.org/10.1007/s40273-014-0133-2>.

Poon LC, Wright D, Rolnik DL, Syngelaki A, Delgado JL, Tsokaki T, et al. Aspirin for Evidence-Based Preeclampsia Prevention trial: effect of aspirin in prevention of preterm preeclampsia in subgroups of women according to their characteristics and medical and obstetrical history. American Journal of Obstetrics & Gynecology. 2017;217(5):585.e1-.e. doi: <https://dx.doi.org/10.1016/j.ajog.2017.07.038>.

Prior M, Hibberd R, Asemota N, Thornton JG. Inadvertent P-hacking among trials and systematic reviews of the effect of progestogens in pregnancy? A systematic review and meta-analysis. BJOG: An International Journal of Obstetrics & Gynaecology. 2017;124(7):1008-15. doi: <https://dx.doi.org/10.1111/1471-0528.14506>.

Rafael TJ, Berghella V, Alfirevic Z. Cervical stitch (cerclage) for preventing preterm birth in multiple pregnancy. Cochrane Database of Systematic Reviews. 2014(9):CD009166. doi: <https://dx.doi.org/10.1002/14651858.CD009166.pub2>.

Roberge S, Sibai B, McCaw-Binns A, Bujold E. Low-Dose Aspirin in Early Gestation for Prevention of Preeclampsia and Small-for-Gestational-Age Neonates: Meta-analysis of Large Randomized Trials. American Journal of Perinatology. 2016;33(8):781-5. doi: <http://dx.doi.org/10.1055/s-0036-1572495>.

Roberge S, Villa P, Nicolaides K, Giguere Y, Vainio M, Bakthi A, et al. Early administration of low-dose aspirin for the prevention of preterm and term preeclampsia: a systematic review and meta-analysis. Fetal Diagnosis & Therapy. 2012;31(3):141-6. doi: <https://dx.doi.org/10.1159/000336662>.

Rode L, Klein K, Larsen H, Holmskov A, Andreasen KR, Uldbjerg N, et al. Cytokines and the risk of preterm delivery in twin pregnancies. Obstetrics and gynecology. 2012;120(1):60‐8. doi: 10.1097/AOG.0b013e31825bc3cd.

Rode L, Langhoff-Roos J, Andersson C, Dinesen J, Hammerum MS, Mohapeloa H, et al. Systematic review of progesterone for the prevention of preterm birth in singleton pregnancies. Acta Obstetricia et Gynecologica Scandinavica. 2009;88(11):1180-9. doi: <https://dx.doi.org/10.3109/00016340903280982>.

Romero R, Nicolaides K, Conde-Agudelo A, Tabor A, O'Brien JM, Cetingoz E, et al. Vaginal progesterone in women with an asymptomatic sonographic short cervix in the midtrimester decreases preterm delivery and neonatal morbidity: a systematic review and metaanalysis of individual patient data. American Journal of Obstetrics & Gynecology. 2012;206(2):124.e1-19. doi: <https://dx.doi.org/10.1016/j.ajog.2011.12.003>.

Romero R, Nicolaides KH, Conde-Agudelo A, O'Brien JM, Cetingoz E, Da Fonseca E, et al. Vaginal progesterone decreases preterm birth <= 34 weeks of gestation in women with a singleton pregnancy and a short cervix: an updated meta-analysis including data from the OPPTIMUM study. Ultrasound in Obstetrics & Gynecology. 2016;48(3):308-17. doi: <https://dx.doi.org/10.1002/uog.15953>.

Rossi AC, Mullin PM. Prevention of pre-eclampsia with low-dose aspirin or vitamins C and e in women at high or low risk: A systematic review with meta-analysis. European Journal of Obstetrics and Gynecology and Reproductive Biology. 2011;158(1):9-16. doi: <http://dx.doi.org/10.1016/j.ejogrb.2011.04.010>.

Rust OA, Atlas RO, Meyn J, Wells M, Kimmel S. Does cerclage location influence perinatal outcome? American Journal of Obstetrics & Gynecology. 2003;189(6):1688-91. doi: 10.1016/s0002-9378(03)00779-8.

Rust OA, Atlas RO, Reed J, van Gaalen J, Balducci J. Revisiting the short cervix detected by transvaginal ultrasound in the second trimester: why cerclage therapy may not help. American Journal of Obstetrics & Gynecology. 2001;185(5):1098-105. doi: 10.1067/mob.2001.118163.

Saccone G, Ciardulli A, Xodo S, Dugoff L, Ludmir J, D'Antonio F, et al. Cervical pessary for preventing preterm birth in twin pregnancies with short cervical length: a systematic review and meta-analysis. Journal of Maternal-Fetal & Neonatal Medicine. 2017a;30(24):2918-25. doi: <https://dx.doi.org/10.1080/14767058.2016.1268595>.

Saccone G, Ciardulli A, Xodo S, Dugoff L, Ludmir J, Pagani G, et al. Cervical Pessary for Preventing Preterm Birth in Singleton Pregnancies With Short Cervical Length: A Systematic Review and Meta-analysis. Journal of Ultrasound in Medicine. 2017b;36(8):1535-43. doi: <https://dx.doi.org/10.7863/ultra.16.08054>.

Saccone G, Rust O, Althuisius S, Roman A, Berghella V. Cerclage for short cervix in twin pregnancies: systematic review and meta-analysis of randomized trials using individual patient-level data. Acta Obstetricia et Gynecologica Scandinavica. 2015;94(4):352-8. doi: <https://dx.doi.org/10.1111/aogs.12600>.

Sanchez-Ramos L, Kaunitz AM, Delke I. Progestational agents to prevent preterm birth: a meta-analysis of randomized controlled trials. Obstetrics & Gynecology. 2005;105(2):273-9. doi: 10.1097/01.AOG.0000150559.59531.b2.

Schisterman EF, Silver RM, Lesher LL, Faraggi D, Wactawski-Wende J, Townsend JM, et al. Preconception low-dose aspirin and pregnancy outcomes: Results from the EAGeR randomized trial. The Lancet. 2014;384(9937):29-36. doi: <http://dx.doi.org/10.1016/S0140-6736%2814%2960157-4>.

Schuit E, Stock S, Rode L, Rouse DJ, Lim AC, Norman JE, et al. Effectiveness of progestogens to improve perinatal outcome in twin pregnancies: an individual participant data meta-analysis. BJOG: An International Journal of Obstetrics & Gynaecology. 2015;122(1):27-37. doi: <https://dx.doi.org/10.1111/1471-0528.13032>.

Senat MV, Porcher R, Winer N, Vayssiere C, Deruelle P, Capelle M, et al. Prevention of preterm delivery by 17 alpha-hydroxyprogesterone caproate in asymptomatic twin pregnancies with a short cervix: a randomized controlled trial. American Journal of Obstetrics & Gynecology. 2013;208(3):194.e1-8. doi: <https://dx.doi.org/10.1016/j.ajog.2013.01.032>.

Shabaan OM, Hassanin IM, Makhlouf AM, Salem MN, Hussein M, Mohamed M, et al. Vaginal progesterone for prevention of preterm delivery in women with twin pregnancy: a randomized controlled trial. Facts Views Vis Obgyn. 2018;10(2):93-8. doi:

Sharma N, Srinivasan S, Srinivasan KJ, Nadhamuni K. Role of Aspirin in High Pulsatility Index of Uterine Artery: A Consort Study. J Obstet Gynaecol India. 2018;68(5):382-8. doi: 10.1007/s13224-017-1058-4.

Short VL, Hoffman M, Metgud M, Kavi A, Goudar SS, Okitawutshu J, et al. Safety of daily low-dose aspirin use during pregnancy in low-income and middle-income countries. AJOG Glob Rep. 2021;1(1). doi: 10.1016/j.xagr.2021.100003.

Sibai BM, Hauth J, Caritis S, Lindheimer MD, MacPherson C, Klebanoff M, et al. Hypertensive disorders in twin versus singleton gestations. National Institute of Child Health and Human Development Network of Maternal-Fetal Medicine Units. American Journal of Obstetrics & Gynecology. 2000;182(4):938-42. doi: 10.1016/s0002-9378(00)70350-4.

Silver RM, Ahrens K, Wong LF, Perkins NJ, Galai N, Lesher LL, et al. Low-dose aspirin and preterm birth: a randomized controlled trial. Obstet Gynecol. 2015;125(4):876-84. doi: 10.1097/aog.0000000000000736.

Simons NE, Leeuw M, van't Hooft J, Limpens J, Roseboom TJ, Oudijk MA, et al. The long-term effect of prenatal progesterone treatment on child development, behaviour and health: a systematic review. BJOG: An International Journal of Obstetrics and Gynaecology. 2020. doi: <http://dx.doi.org/10.1111/1471-0528.16582>.

Sotiriadis A, Papatheodorou S, Makrydimas G. Perinatal outcome in women treated with progesterone for the prevention of preterm birth: a meta-analysis. Ultrasound in Obstetrics & Gynecology. 2012;40(3):257-66. doi: <https://dx.doi.org/10.1002/uog.11178>.

Spong CY, Meis PJ, Thom EA, Sibai B, Dombrowski MP, Moawad AH, et al. Progesterone for prevention of recurrent preterm birth: Impact of gestational age at previous delivery. American Journal of Obstetrics and Gynecology. 2005;193(3 SUPPL.):1127-31. doi: <http://dx.doi.org/10.1016/j.ajog.2005.05.077>.

Szychowski JM, Berghella V, Owen J, Hankins G, Iams JD, Sheffield JS, et al. Cerclage for the prevention of preterm birth in high risk women receiving intramuscular 17-alpha-hydroxyprogesterone caproate. Journal of Maternal-Fetal & Neonatal Medicine. 2012;25(12):2686-9. doi: <https://dx.doi.org/10.3109/14767058.2012.717128>.

Szychowski JM, Owen J, Hankins G, Iams JD, Sheffield JS, Perez-Delboy A, et al. Can the optimal cervical length for placing ultrasound-indicated cerclage be identified? Ultrasound in Obstetrics & Gynecology. 2016;48(1):43-7. doi: <https://dx.doi.org/10.1002/uog.15674>.

Talari H, Mesdaghinia E, Abedzadeh Kalahroudi M. Aspirin and preeclampsia prevention in patients with abnormal uterine artery blood flow. Iran Red Crescent Med J. 2014;16(8):e17175. doi: 10.5812/ircmj.17175.

Tewari S, Kaushish R, Sharma S, Gulati N. Role of low dose aspirin in prevention of pregnancy induced hypertension. Journal of the Indian Medical Association. 1997;95(2):43-4, 7. doi:

Thangatorai R, Lim FC, Nalliah S. Cervical pessary in the prevention of preterm births in multiple pregnancies with a short cervix: PRISMA compliant systematic review and meta-analysis. Journal of Maternal-Fetal & Neonatal Medicine. 2018;31(12):1638-45. doi: <https://dx.doi.org/10.1080/14767058.2017.1319930>.

Tolcher MC, Sangi-Haghpeykar H, Mendez-Figueroa H, Aagaard KM. Low-dose aspirin for preeclampsia prevention: efficacy by ethnicity and race. American Journal of Obstetrics & Gynecology MFM. 2020;2(4):100184. doi: <https://dx.doi.org/10.1016/j.ajogmf.2020.100184>.

Trivedi NA. A meta-analysis of low-dose aspirin for prevention of preeclampsia. Journal of Postgraduate Medicine. 2011;57(2):91-5. doi: <http://dx.doi.org/10.4103/0022-3859.81858>.

Turner JM, Robertson NT, Hartel G, Kumar S. Impact of low-dose aspirin on adverse perinatal outcome: meta-analysis and meta-regression. Ultrasound in Obstetrics & Gynecology. 2020;55(2):157-69. doi: <https://dx.doi.org/10.1002/uog.20859>.

van Limburg Stirum EVJ, Zegveld SJ, Simons NE, de Boer MA, Pajkrt E, Mol BWJ, et al. Consequences of cervical pessary for subsequent pregnancies: follow-up of randomized clinical trial (ProTWIN). Ultrasound in Obstetrics & Gynecology. 2021;26:26. doi: <https://dx.doi.org/10.1002/uog.24821>.

van Os MA, Kleinrouweler CE, Schuit E, van der Ven AJ, Pajkrt E, de Groot CJ, et al. Influence of cut-off value on prevalence of short cervical length. Ultrasound in Obstetrics & Gynecology. 2017;49(3):330-6. doi: <https://dx.doi.org/10.1002/uog.15967>.

van Vliet EO, Askie LA, Mol BW, Oudijk MA. Antiplatelet Agents and the Prevention of Spontaneous Preterm Birth: A Systematic Review and Meta-analysis. Obstetrics & Gynecology. 2017;129(2):327-36. doi: <https://dx.doi.org/10.1097/AOG.0000000000001848>.

Velez Edwards DR, Likis FE, Andrews JC, Woodworth AL, Jerome RN, Fonnesbeck CJ, et al. Progestogens for preterm birth prevention: a systematic review and meta-analysis by drug route. Archives of Gynecology & Obstetrics. 2013;287(6):1059-66. doi: <https://dx.doi.org/10.1007/s00404-013-2789-9>.

Villa PM, Kajantie E, Raikkonen K, Pesonen AK, Hamalainen E, Vainio M, et al. Aspirin in the prevention of pre-eclampsia in high-risk women: A randomized placebo-controlled PREDO Trial and a meta-analysis of randomized trials. BJOG: An International Journal of Obstetrics and Gynaecology. 2013;120(1):64-74. doi: <http://dx.doi.org/10.1111/j.1471-0528.2012.03493.x>.

Winer N, Bretelle F, Senat MV, Bohec C, Deruelle P, Perrotin F, et al. 17 alpha-hydroxyprogesterone caproate does not prolong pregnancy or reduce the rate of preterm birth in women at high risk for preterm delivery and a short cervix: a randomized controlled trial. American Journal of Obstetrics & Gynecology. 2015;212(4):485.e1-.e10. doi: <https://dx.doi.org/10.1016/j.ajog.2014.10.1097>.

Wing DA, Szychowski J, Owen J, Hankins G, Iams JD, Sheffield JS, et al. Gestational age at previous preterm birth does not affect cerclage efficacy. American Journal of Obstetrics & Gynecology. 2010;203(4):377.e1-4. doi: <https://dx.doi.org/10.1016/j.ajog.2010.05.018>.

Wright D, Nicolaides KH. Aspirin delays the development of preeclampsia. American Journal of Obstetrics & Gynecology. 2019;220(6):580.e1-.e6. doi: <https://dx.doi.org/10.1016/j.ajog.2019.02.034>.

Wright D, Poon LC, Rolnik DL, Syngelaki A, Delgado JL, Vojtassakova D, et al. Aspirin for Evidence-Based Preeclampsia Prevention trial: influence of compliance on beneficial effect of aspirin in prevention of preterm preeclampsia. American Journal of Obstetrics & Gynecology. 2017;217(6):685.e1-.e. doi: <https://dx.doi.org/10.1016/j.ajog.2017.08.110>.

Xiong YQ, Tan J, Liu YM, Qi YN, He Q, Li L, et al. Cervical pessary for preventing preterm birth in singletons and twin pregnancies: an update systematic review and meta-analysis. Journal of Maternal-Fetal & Neonatal Medicine. 2020:1-10. doi: <https://dx.doi.org/10.1080/14767058.2020.1712705>.

Xu TT, Zhou F, Deng CY, Huang GQ, Li JK, Wang XD. Low-Dose Aspirin for Preventing Preeclampsia and Its Complications: A Meta-Analysis. Journal of Clinical Hypertension. 2015;17(7):567-73. doi: <https://dx.doi.org/10.1111/jch.12541>.

Yan Y, Chen Z, Yang Y, Zheng X, Zou M, Cheng G, et al. Efficacy of progesterone on threatened miscarriage: an updated meta-analysis of randomized trials. Archives of Gynecology and Obstetrics. 2020. doi: <http://dx.doi.org/10.1007/s00404-020-05808-8>.

Yu CK, Papageorghiou AT, Parra M, Palma Dias R, Nicolaides KH, Fetal Medicine Foundation Second Trimester Screening G. Randomized controlled trial using low-dose aspirin in the prevention of pre-eclampsia in women with abnormal uterine artery Doppler at 23 weeks' gestation. Ultrasound in Obstetrics & Gynecology. 2003;22(3):233-9. doi: 10.1002/uog.218.

Zheng L, Dong J, Dai Y, Zhang Y, Shi L, Wei M, et al. Cervical pessaries for the prevention of preterm birth: a systematic review and meta-analysis. Journal of Maternal-Fetal & Neonatal Medicine. 2019;32(10):1654-63. doi: <https://dx.doi.org/10.1080/14767058.2017.1414795>.

Table 1 Reason for exclusion **for randomized controlled trials and systematic reviews**

| **Author year** | **Randomized controlled trials: Reason for exclusion** |
| --- | --- |
| Abdel 2021 | Wrong intervention: Comparing different dosages, no placebo. |
| Abdi 2020 | Wrong population: Women with a previous history of preeclampsia (PE). |
| Abramovici 2015 | Wrong population: Women with low or high risk of PE. |
| Agra 2019 | Wrong outcome: Women with cervical shortening. |
| Allshouse 2016 | Wrong population: Women with high risk of PE. |
| Althuisius 2000 | Preliminary results. Double publication, Althuisius 2001. |
| Althuisius 2002 | Wrong outcome: Cervical length. |
| Andrikopoulou 2018 | Wrong population: Nulliparous women without any specific risk factors. Secondary analysis of Sibai 1993. |
| Atallah 1996 | Wrong population: Women with high risk of PE. |
| Ayala 2013 | Wrong population: Women with high risk of PE. |
| Bakhtii 2011 | Wrong population: Nulliparous women without any specific risk factors. |
| Berghella 2010a | Wrong study design: No RCT. |
| Beroyz 1994 | Wrong population: Women with high risk of PE or intra uterine growth restriction (IUGR). |
| Buchbinder 2002 | Wrong population: Women with a previous history of PE. |
| Caritis 1998 | Wrong population: Women with high risk of PE. |
| Caritis 2012 | Wrong intervention: Concentration of 17-hydroxyprogesteron caproate (17-OHPC) in blood. |
| Caritis 2014 | Wrong intervention: Concentration of 17-OHPC in blood. |
| Connealy 2014 | Wrong population: Women with a previous history of PE. |
| Corrado 2002 | Wrong population: Women who underwent midtrimester amniocentesis. |
| De Franco 2007 | Double publication, O´Brien 2007. |
| Durnwald 2010 | Secondary analysis of Rouse, 2007. |
| Ebrashy 2005 | Wrong population: Women with high risk of PE or IUGR. |
| Eddama 2010 | Wrong study design: Cost effectiveness article. |
| El Refaie 2016 | Retracted article. |
| Farinelli 2012 | Wrong intervention: Association between body mass index and pregnancy outcome. |
| Fratto 2016 | Wrong population: Women with low or high risk of PE. |
| Gu 2020 | Wrong population: Women with risk of PE. |
| Hajizadeh 2020 | Wrong design: Pseudorandomization. Wrong intervention: Unclear gestational age at start of intervention. |
| Hamid 1994 | Wrong population: Women with raised alpha-fetoprotein in blood and abnormal doppler waveform pattern. |
| Hartikainen-Sorri 1980 | Wrong intervention: 17-OHPC in third trimester. |
| Heyborne 2016 | Secondary analysis of PTB and smoking, Meis 2003. |
| Heyborne 2015 | Secondary analysis of PTB and obese women, Meis 2003. |
| Hezelgrave 2016 | Wrong study design: Study protocol. |
| Hoffman 2020 | Wrong population: Nulliparous women without any specific risk factor |
| Huai 2021 | Wrong population: Women with high risk of PE. |
| Jessani 2021 | Wrong outcome: Hemoglobin level. Secondary analysis of Hoffman 2020. |
| Keeler 2009b | Double publication to Rust 2001, Roman 2003, Rust 2007. |
| Khazardoost 2014 | Wrong population: Women with raised alfa-fetoprotein in blood. |
| Kumar 2014 | Wrong intervention: Oral progesterone in first trimester. |
| Kumar 2020 | Wrong population: Women with high risk of PE. |
| Lambers 2009 | Wrong outcome: Hypertensive disorders in pregnancy. |
| Le 2020 | Wrong study design: Cost effectiveness article. |
| Leslie 1995 | Wrong population: Women with high risk of PE. |
| Liem 2014 | Wrong study design: Cost effectiveness article. |
| Liem 2016 | Double publication, Liem 2013, per protocol analysis. |
| Mancuso 2010 | Wrong population: Women with different type of cervical funneling. |
| Manuck 2011 | Wrong intervention: Receptor polymorphisms and response to 17-alpha-hydroxyprogesterone caproate. |
| Marat 2019 | Wrong study design: No RCT. |
| Macnaughton 1988 | Wrong study design: Interim report. |
| Meis 2005 | Wrong outcome: Secondary analysis on different risk factors, Meis 2003. |
| Mendoza 2017 | Wrong outcome: Cervical length. Secondary analysis, Goya 2012. |
| Mourad 2019 | Double publication with Mourad 2021 (same article first published on-line) |
| Mourad 2021 | Secondary publication analyzing the effect of GDM on hypertensive disorder of pregnancy |
| Moussa 2015 | Wrong population: Women with high risk of PE. |
| Odibo 2015 | Wrong population: Women with high risk of PE. |
| Pizzi 2014 | Wrong study design: Cost effectiveness article. |
| Poon 2017 | Wrong population: Women with high risk of PE. |
| Rode 2012 | Wrong outcome: Secondary analysis of Rode 2011, inflammatory markers and PTB. |
| Rust 2001 | Wrong outcome: Secondary analysis of Rust 2000, PTB and different risk factors. |
| Rust 2003 | Wrong outcome: Gestational age (GA) in relationship with location of cerclage. |
| Schisterman 2014 | Wrong outcome: Preconception initiated ASA and live birth. |
| Senat 2013 | Wrong intervention: Intervention (progesterone) starts too late in pregnancy. |
| Shabaan 2018 | Wrong intervention: Intervention (progesterone) starts too late in pregnancy (third trimester). |
| Sharma 2018 | Wrong population: Women with high risk of PE. |
| Short 2021 | Wrong outcome: Safety of ASA. Secondary analysis of Hoffman 2020. |
| Sibai 2000 | Wrong intervention: Comparing singleton and twin pregnancies. Secondary analysis of Sibai 1993 (ASA) |
| Silver 2015 | Wrong population: Secondary analysis of Schisterman 2014 (EAGER trial). |
| Spong 2005 | Wrong outcome: GA compared to GA of last delivery. Secondary analysis of Meis 2003. |
| Szychowski 2012 | Secondary analysis of Owen 2009. Additional effect of cerclage in women randomized to 17-OHPC |
| Szychowski 2016 | Wrong outcome: Cervical length. Secondary analysis of Owen 2009. |
| Talari 2014 | Wrong population: Women with high risk of PE. Wrong outcome: Incidence of PE. |
| Tewari 1997 | Wrong population: Women with high risk of PE. |
| Tolcher 2020 | Wrong population: Women with high risk of PE. |
| Van Limburg 2021 | Wrong outcome: Pessary effect on subsequent pregnancy. Secondary analysis of Liem 2013. |
| van Os 2017 | Wrong outcome: Cervical length cut off values. Secondary analysis of van der Veen 2015. |
| Winer 2015 | Wrong intervention: Intervention (progesterone) starts too late in pregnancy. |
| Wing 2010 | Wrong study design: Secondary analysis, Owen 2009. Observational study on risk factors for PTB (gestational age at prior PTB). |
| Wright 2019 | Wrong population: Women with high risk vs low risk of PE. |
| Wright 2017 | Wrong population: Women with high risk of PE. |
| Yu 2003 | Wrong population: Women with abnormal uterine artery Doppler at 23 weeks |
| **Author, year** | **Systematic reviews and reason for exclusion** |
| Abdel-Aleem 2013 | SR without unique study data |
| Ahn 2017 | SR without unique study data |
| Almutairi 2021 | SR without unique study data |
| Bachmann 2003 | SR without unique study data |
| Baradwan 2021 | SR without unique study data |
| Belej Rak 2003 | SR without unique study data |
| Berghella 2010b | SR without unique study data |
| Berghella 2011 | SR without unique study data |
| Berghella 2017b | SR without unique study data |
| Boelig 2019 | SR with wrong intervention (vaginal progesterone vs im progesterone) |
| Bujold 2010 | SR without unique study data |
| Chaemsaithong 2020 | SR with wrong population and outcome (PE) |
| Chaman Ara 2016 | SR without unique study data |
| Combs 2016 | SR without unique study data |
| Conde Agudelo 2018 | SR without unique study data |
| Conde Agudelo 2020 | SR without unique study data |
| Coomarasamy 2003 | SR with wrong outcome (PE) |
| Coomarasamy 2006 | SR without unique study data |
| Correa 2019 | SR without unique study data |
| Cui 2018 | SR with wrong population (PE) |
| D´Antonio 2021 | SR without unique study data |
| De Jong 2014 | SR with wrong population and outcome (ASA and recurrent miscarriage) |
| Dodd 2006 | SR without unique study data |
| Dodd 2008 | SR without unique study data |
| Dodd 2019 | SR without unique study data |
| Dodd 2013 | SR without unique study data |
| Dodd 2005 | SR without unique study data |
| Drakeley 2003a | SR without unique study data |
| Drakeley 2003b | SR without unique study data |
| Duley 2019 | SR with wrong population (PE) |
| Eke 2019b | SR without unique study data |
| Eke 2019a | SR without unique study data |
| Fernandez-Macias 2019 | SR without unique study data |
| Ghazanfarpour 2020 | SR with wrong outcome (PE) |
| Goldstein 1989 | SR without unique study data |
| Grabovac 2019 | SR without unique study data |
| Groeneveld 2013 | SR with wrong outcome (PE) |
| Haas 2019 | SR with wrong outcome (miscarriage) |
| Henderson 2014a | SR with wrong outcome (PE) |
| Henderson 2014b | SR with wrong outcome (PE) |
| Jarde 2017a | SR without unique study data |
| Jarde 2017b | SR without unique study data |
| Jarde 2017c | SR without unique study data |
| Jarde 2019 | SR without unique study data |
| Jin 2017 | SR without unique study data |
| Jin 2019 | SR without unique study data |
| Jorgensen 2007 | SR without unique study data |
| Kozer 2003 | SR without unique study data |
| Li 2019 | SR without unique study data |
| Liem 2013b | SR without unique study data |
| Likis 2012a | SR without unique study data |
| Likis 2012b | SR without unique study data |
| Liu 2019 | SR without unique study data |
| Liu 2013 | SR without unique study data |
| Mackenzie 2006 | SR without unique study data |
| Man 2021 | SR with wrong population: ASA in nulliparous women without any specific risk factor |
| Meher 2017 | SR with wrong population (PE) |
| Meher 2006 | SR with wrong outcome (PE) |
| Odibo 2003 | SR without unique study data |
| Perez-Lopez 2019 | SR without unique study data |
| Phung 2021 | SR without unique study data |
| Prior 2017 | SR with wrong outcome (P-hacking) |
| Rafael 2014 | SR without unique study data |
| Roberge 2016 | SR with wrong outcome (PE) |
| Roberge 2012 | SR with wrong outcome (PE) |
| Rode 2009 | SR with wrong intervention and comparison (cytokines in twin pregnancies) |
| Romero 2012 | SR without unique study data |
| Romero 2016 | SR without unique study data |
| Rossi 2011 | SR with wrong outcome (PE) |
| Saccone 2015 | SR without unique study data |
| Saccone 2017a | SR without unique study data |
| Saccone 2017b | SR without unique study data |
| Sanchez-Ramos 2005 | SR without unique study data |
| Schuit 2015 | SR without unique study data |
| Simons 2020 | Same article as Simons 2021 |
| Sotiriadis 2012 | SR without unique study data |
| Thangatorai 2018 | SR without unique study data |
| Trivedi 2011 | SR with wrong outcome (PE) |
| Turner 2020 | SR with wrong population (ASA on different indications) |
| van Vliet 2017 | SR with wrong population (ASA for prevention of PE) |
| Velez Edwards 2013 | SR without unique study data |
| Villa 2013 | SR with wrong outcome (PE) |
| Xiong 2020 | SR without unique study data |
| Xu 2015 | SR with wrong outcome (PE) |
| Yan 2020 | SR with wrong outcome (miscarriage) |
| Zheng 2019 | SR without unique study data |

ASA; acetylsalicylic acid, GA; gestational age, IUGR; intrauterine growth retardation, PE; preeclampsia, PTB; preterm birth, RCT; randomized controlled trial, SR; systematic review
