# Supplementary material for: Progesterone, cerclage, pessary, or acetylsalicylic acid for prevention of preterm birth in singleton and multifetal pregnancies – A systematic review and meta-analyses
Source: Front Med (Lausanne). 2023 Feb 28;10:1111315. doi: 10.3389/fmed.2023.1111315 (PMC10015499; doi:10.3389/fmed.2023.1111315)
Supplement: Supplementary file 1 [file Data_Sheet_1.zip › Data Sheet 1_corrected/Appendix 4.1 Outcome table_Progesterone.docx]

**Progesterone, cerclage, pessary, or acetylsalicylic acid for prevention of preterm birth**

**in singleton and multifetal pregnancies**

**Appendix 4.1**  [**Outcome tables**](#_Toc104894177) **progesterone in singleton and multifetal pregnancies**

**Preterm birth**

STable 4.1.1.a Any preterm birth <37 weeks

STable 4.1.1.b Spontaneous preterm birth <37 weeks

STable 4.1.2.a Any preterm birth <35 weeks

STable 4.1.2.b Spontaneous preterm birth <35 weeks

STable 4.1.3.a Any preterm birth <34 weeks

STable 4.1.3.b Spontaneous preterm birth <34 weeks

STable 4.1.4.a Any spontaneous preterm birth <32 weeks

STable 4.1.5.a Spontaneous preterm birth <32 weeks

STable 4.1.6.a Any preterm birth <28 weeks

STable 4.1.6.b Spontaneous preterm birth <28 weeks

**Gestational age and birth weight**

STable 4.1.7 Gestational age at delivery

STable 4.1.8 Low birth weight

STable 4.1.9 Very low birth weight

**Perinatal mortality and neonatal morbidity**

STable 4.1.10 Perinatal mortality

STable 4.1.11 Neonatal mortality <28 days

STable 4.1.12 Composite adverse neonatal outcome

STable 4.1.13 Respiratory distress syndrome

STable 4.1.14 Bronchopulmonary dysplasia

STable 4.1.15 Intraventricular hemorrhage

STable 4.1.16 Necrotizing enterocolitis

STable 4.1.17 Neonatal sepsis

STable 4.1.18 Retinopathy of prematurity

STable 4.1.19 Admittance to neonatal intensive care unit

STable 4.1.20 Long term child outcome

**Maternal mortality and morbidity**

STable 4.1.21 Maternal mortality

STable 4.1.22 Hypertensive disorders in pregnancy

STable 4.1.23 Gestational diabetes mellitus

STable 4.1.24 Cholestasis

STable 4.1.25 Infectious including chorioamnionitis

STable 4.1.26 Preterm prelabor rupture of membranes

Prevention of preterm birth

STable 4.1.1.a. Intervention progesterone

Outcome variable: Any preterm birth before 37 gestational weeks

* + No or minor problems

? Some problems

- Major problems

| **Author, year Country**  **Trial acronym** | **Singletons/ Twins/ Triplets** | **Risk factor** | | **Number**  **of randomized**  **patients**  **n=** | | **Results** | | | | **Comments** | | **Directness *** | | **Study limitations *** | | **Precision *** | |  |
| --- | --- | --- | --- | --- | --- | --- | --- | --- | --- | --- | --- | --- | --- | --- | --- | --- | --- | --- |
|  |  |  |  |  |  | **Intervention** | | **Control** | |  |  |  |  |  |  |  |  |  |
|  |  | |  | |  | |  | |  | |  | |  | |  | |  | |
| Aflatoonian, 2013 Iran | Singletons | | ART pregnancies | | I: 52  C: 47 | | 17-OHPC 250 mg im/w 4/52 (7.7%)?  RR 2.48 (95% CI: 0.81-9.94) p=0.203 | | Placebo  9/47 (19.1%)? Table 1  8/47? (17.0%)? Table 2 | | PO not stated Incorrect numbers in the  article? | | ? | | - | | - | |
| Ali, 2020  Egypt | Singletons | | Indication for cerclage: previous  second trimester loss,  sPTD (<34 w) or short cervix (<25 mm) | | I: 121 C:121 | | 400 mg progesterone vag (pessary)/d  12/97 (12.4%) p=0.005 | | Placebo 12/75 (16.0%) | | Not PO Progesterone was used as an  adjuvant after cerclage NB PTB 28-34 weeks, with spontaneous abortion< 28  weeks = primary outcome was excluded | | ? | | ? | | ? | |
| Ashoush, 2017 Egypt | Singletons | | Previous sPTB (<37 w) | | I: 106  C: 106 | | 400 mg oral progesterone/d 43/96 (44.8%)  RR 0.7 (95% CI 0.54-0.92) p=0.01 | | Placebo 58/91 (63.7%) | | Not PO  NB critical comment to article by Katsanevakis, Mol and Thornton concerning recruitment and results  NB High rates of cerclage in both groups | | - | | ? | | - | |
| Azargoon, 2016 Iran | Singletons | | Previous PTB (<37 w),  uterine malformations | | I: 51  C: 52 | | 400 mg progesterone supp vag/d 18/50 (36%)  RR 0.53 (95% CI 0.35-0.80)  (from MA) | | Placebo 34/50 (68 %)  RR 1.89 (95% CI 1.25-2.86) p=0.001 | | PO  Results also for previous PTB and previous PTB and short TVS CL  In article RR for controls vs intervention, RR for  intervention vs controls from MA | | - | | ? | | - | |
| Blackwell, 2020 USA PROLONG | Singletons | | Previous singleton sPTB | | I: 1130  C: 578  Randomized 2:1 | | 17-OHPC 250 mg im/w 257/1112 (23.1%)  RR 1.06 (95% CI 0.88–1.28)  No p value | | Placebo 125/572 (21.9%) | | Not PO | | + | | ? | | ? | |
| Da Fonseca, 2003 Brazil | Singletons | | Previous sPTB, prophylactic cerclage, uterine malformations | | I: 81 C:76 | | 100 mg vaginal progesterone/d 10/72 (13.9%)  p=0.03 | | Placebo 20/70 (28.6%) | | PO not defined | | ? | | ? | | ? | |

Prevention of preterm birth

STable 4.1.1.a. cont. Intervention progesterone

Outcome variable: Any preterm birth before 37 gestational weeks

* + No or minor problems

? Some problems

- Major problems

| **Author, year Country**  **Trial acronym** | **Singletons/ Twins/ Triplets** | **Risk factor** | | **Number**  **of randomized**  **patients**  **n=** | | **Results** | | | | **Comments** | | **Directness *** | | **Study limitations *** | | **Precision *** | |  |
| --- | --- | --- | --- | --- | --- | --- | --- | --- | --- | --- | --- | --- | --- | --- | --- | --- | --- | --- |
|  |  |  |  |  |  | **Intervention** | | **Control** | |  |  |  |  |  |  |  |  |  |
|  |  | |  | |  | |  | |  | |  | |  | |  | |  | |
| Grobman, 2012 USA  SCAN | Singletons | | Nulliparous with short TVS CL  <30 mm | | I: 327  C: 330 | | 17-OHPC 250 mg im/w 82/327 (25.1%)  RR 1.03  (95% CI 0.79-1.35)  No p value | | Placebo (castor oil) 80/330 (24.2%) | | PO | | ? | | + | | + | |
| Hassan, 2011 USA + 9 other countries PREGNANT | Singletons | | Short TVS CL 10-20 mm (all)  Previous PTB 16% | | I: 236  C: 229 | | 90 mg vaginal progesterone gel 8%/d  71/235 (30.2%)  RR 0.89 (95% CI 0.68-1.16) p=0.376 | | Placebo 76/223 (34.1%) | | Not PO | | ? | | ? | | + | |
| Hauth, 1983 USA | Singletons | | Women from an active –duty military population | | I: 80  C: 88 | | 17-OHPC 1000mg im/w  5/80 (6.3%) NS  No p value, OR or RR | | Placebo (castor oil) 5/88 (5.7%) | | PO not defined | | - | | - | | - | |
| Hayashi, 2021 Japan TROPICAL | Singletons | | Short TVS CL 25-<30 mm  Previous PTB I: 11.9%  C: 16.7% | | I: 59  C: 60 | | 200 mg vaginal progesterone/d  2/59 (3.4%) p=0.029  No RR | | Placebo 9/60 (15%) | | Not PO | | ? | | ? | | - | |
| Ibrahim, 2010 Egypt | Singletons | | Previous PTB | | I: 25  C: 25 | | 17-OHPC 250 mg/w  8/25 (32%)  RR 0.079  (95% CI 0.021-0.302) | | Placebo (saline) 13/25 (52%) | | PO not defined | | - | | - | | - | |
| Jabeen, 2012 Pakistan | Singletons | | Previous sPTB | | I: 30  C: 30 | | 17-OHPC 250 mg/w  11/30 (36.7%)  p< 0.001 | | Placebo (inert oil) 25/30 (83.3%) | | PO | | ? | | - | | - | |
| Jafarpour, 2020 Iran | Singletons | | Previous PTB | | I: 50  C: 50 | | 17-OHPC 250 mg/w  21/50 (42%)  RR 1.2 (95% CI 0.85-1.88) p=0.23 | | Routine prenatal care 27/50 (54%) | | PO? (not defined) | | ? | | - | | - | |
| Majhi, 2009 India | Singletons | | Previous sPTB | | I: 50  C: 50 | | 100 mg vaginal progesterone/d 6/50 (12%)  RR 0.315  (95% CI 0.137-0.724)  p= 0.0027 | | No placebo 19/50 (38%) | | PO? | | ? | | ? | | ? | |

* + No or minor problems

? Some problems

- Major problems

Prevention of preterm birth

STable 4.1.1.a. cont. Intervention progesterone

Outcome variable: Any preterm birth before 37 gestational weeks

| **Author, year Country**  **Trial acronym** | **Singletons/ Twins/ Triplets** | **Risk factor** | | **Number**  **of**  **randomized**  **patients**  **n=** | | **Results** | | | | **Comments** | | **Directness *** | | **Study limitations *** | | **Precision *** | |  |
| --- | --- | --- | --- | --- | --- | --- | --- | --- | --- | --- | --- | --- | --- | --- | --- | --- | --- | --- |
|  |  |  |  |  |  | **Intervention** | | **Control** | |  |  |  |  |  |  |  |  |  |
|  |  | |  | |  | |  | |  | |  | |  | |  | |  | |
| Meis, 2003 USA | Singletons | | Previous sPTB | | I:310 C:153  Randomized2:1 | | 17-OHPC 250 mg im/w 111/306 (36.3%)  RR 0.66 (95% CI 0.54-0.81)  No p value | | Placebo (castor oil) 84/153 (54.9%) | | PO | | ? | | ? | | ? | |
| O’Brien, 2007  USA (+4 other countries) | Singletons | | Previous sPTB | | I: 332  C: 327 | | 90 mg vaginal progesterone gel 8%/d  129/309 (41.7%)  OR 1.08 (95% CI 0.76-1.52)  No p value | | Placebo 123/302 (40.7%) | | Not PO | | ? | | ? | | ? | |
| Price, 2021 Zambia | Singletons | | HIV | | I: 399 C:401 | | 17-OHPC 250 mg/w  31/399 (8%)  RR 0.9 (95% CI 0.6-1.4)  No p value | | Placebo 35/401 (9%) | | Not PO | | ? | | + | | ? | |
| Rai, 2009  India | Singletons | | Previous sPTB | | I: 75 C:75 | | 200 micronized oral progesterone/d 29/74 (39.2%)  No RR or OR p=0.002 | | Placebo 44/74 (59.5%) | | Not PO | | - | | ? | | ? | |
| Saghafi, 2011 Iran | Singletons | | Previous PTB | | I: 50  C: 50 | | 17-OHPC 250 mg im/w 16/50 (32%)  p<0.05 | | No placebo 30/50 (60%) | | PO not defined  Not stated but assumed to be only singletons | | - | | - | | - | |
| Shadab, 2018 Pakistan | Singletons | | Previous sPTB | | I: 66  C: 66 | | 17-OHPC 250 mg im/w 19/66 (28.8%)  No OR or RR p=0.00045 | | Vitamin B im as placebo 39/66 (59.1%) | | PO not defined | | - | | - | | - | |
| Shahgheibi, 2016 Iran | Singletons | | Previous sPTB, uterine mal- formations | | I: 50  C: 50 | | 17-OHPC 250 mg im/w 11/50 (22%)  OR 0.69 (95% CI 0.58-0.83)  No p value | | Placebo 29/50 (58%) | | PO not defined | | - | | - | | - | |
| Van Os, 2015 The Netherlands TRIPLE P | Singleton | | Short TVS CL  ≤30 mm,  no previous PTB | | I: 41  C: 39 | | 200 mg micronized progesterone vaginal/d  9/41 (22%)  RR1.25 (95% CI 0.52-3.03)  No p value | | Placebo 7/39 (18%) | | Not PO | | + | | ? | | - | |

Prevention of preterm birth

STable 4.1.1.a. cont. Intervention progesterone

Outcome variable: Any preterm birth before 37 gestational weeks

* + No or minor problems

? Some problems

- Major problems

| **Author, year Country**  **Trial acronym** | **Singletons/ Twins/ Triplets** | **Risk factor** | | **Number**  **of randomized patients**  **n=** | | **Results** | | | | **Comments** | | **Directness *** | | **Study limitations *** | | **Precision *** | |  |
| --- | --- | --- | --- | --- | --- | --- | --- | --- | --- | --- | --- | --- | --- | --- | --- | --- | --- | --- |
|  |  |  |  |  |  | **Intervention** | | **Control** | |  |  |  |  |  |  |  |  |  |
|  |  | |  | |  | |  | |  | |  | |  | |  | |  | |
| Yemini, 1985 Israel | Singletons | | Previous ≥2 PTB or  ≥2 spontaneous  miscarriages | | I: 39  C: 40 | | 17-OHPC 250 mg im/w 5/31(16.1%)  p<0.05  RR not presented | | Placebo 14/37 (37.8%) | | Not PO  All had cerclage Miscarriage occurred in 8 and 3  women | | - | | - | | - | |
| Awwad, 2015 Lebanon PROGESTWIN | Twins, unselected | | ART 75%  MC 17% | | I:197 C: 96  Randomized 2:1 | | 17-OHPC 250 mg im/w 119/194 (61.3%)  RR 1.0 (95% CI 0.6-1.6) p=0.95 | | Placebo (castor oil) 58/94 (61.7%) | | PO | | + | | + | | ? | |
| Briery, 2009 USA | Twins, unselected | | 1/3 previous PTB | | I: 16  C: 14 | | 17-OHPC 250 mg im/w 14/16 (87.5%)  p=0.565 | | Placebo 13/14 (92.9%) | | Not PO Numbers for PTB <37 w calculated from Table 2 | | ? | | ? | | - | |
| Brizot, 2015 Brazil | Twins DA | | MC I: 25%  C: 19%  Only naturally conceived,  no history of PTB | | I: 195  C: 195 | | 200 mg vaginal natural progesterone/d 127/189 (67.2%)  OR 1.32 (95% CI 0.85-2.06)  No p value  Subgroup analysis TVS CL ≤25 mm n=22  20/22 (90.9%) | | Placebo 116/191 (60.7%)  Subgroup analysis TVS CL ≤25 mm n=13  12/13 (92.3%) | | Not PO  Subgroup analysis TVS CL ≤25 mm | | ? | | ? | | - | |
| Combs, 2011 USA | Twins DCDA | | 20% fetal reduction ART  I: 66% C:58%  Prior PTB: I: 12%  C: 13% | | I: 160  C: 80  Randomized 2:1 | | 17-OHPC 250 mg (in 1 mL castor oil) im/w  113/160 (71%)  RR 1.2 (95% CI 1.0-1.5) p=0.08 | | Placebo (1 mL castor oil) 46/78 (59%) | | Not PO | | + | | ? | | ? | |
| Rehal, 2021  UK (+5 other European countries) | Twins | | MC I: 23%  C: 23% ART I: 34%  C: 35% | | I: 582  C: 587 | | 600 mg vaginal progesterone/d 330/582 (56.7%)*  OR 1.13 (95% CI 0.88-1.46)* 315/567 (55.6%)**  OR 1.16 (95% CI 0.90-1.51)**  No p values | | Placebo 322/587 (54.9%)*  296/561 (52.8%)** | | *any birth after randomization to 37 w  **any birth 24-37 w | | + | | + | | ? | |

Prevention of preterm birth

STable 4.1.1.a. cont. Intervention progesterone

Outcome variable: Any preterm birth before 37 gestational weeks

* + No or minor problems

? Some problems

- Major problems

| **Author, year Country**  **Trial acronym** | **Singletons/ Twins/ Triplets** | **Risk factor** | | **Number**  **of randomized**  **patients**  **n=** | | **Results** | | | | **Comments** | | **Directness *** | | ***Study limitations **** | | **Precision *** | |  |
| --- | --- | --- | --- | --- | --- | --- | --- | --- | --- | --- | --- | --- | --- | --- | --- | --- | --- | --- |
|  |  |  |  |  |  | **Intervention** | | **Control** | |  |  |  |  |  |  |  |  |  |
|  |  | |  | |  | |  | |  | |  | |  | |  | |  | |
| Rode, 2011 Denmark and Austria PREDICT | Twins (DA) | | MC  I: 43/334 (12.9%)  C: 57/343 (16.6%) | | I: 334  C: 343 | | 200 mg vaginal progesterone/d (pessary)  158/334 (47.3%)  OR 0.8 (95% CI 0.6-1.1)  No p values | | Placebo 179/341 (52.5%) | | Not PO | | *+* | | *+* | | + | |
| Rouse, 2007 USA SSTARS | Twins (DA) | | MC  I: 59/327 (18%)  C: 57/334 (17.1%) | | I: 327  C: 334 | | 17-OHPC 250 mg im/w 226/325 (69.5%)*  RR 1.0 (95% CI 0.9-1.1)* | | Placebo 232/330 (70.3%)* | | Not PO  *PTB or fetal death <37 weeks (fetal death includes miscarriage, termination of pregnancy, and stillbirth) | | *+* | | *?* | | + | |
| Serra, 2013 Spain | Twins (DCDA) | | MAR  I1: 92/96 (95.8%)  I2: 94/97 (96.9%)  C:96/97 (99.0%) | | I1: 98 I2:98 C: 98 | | I1: 200 mg vaginal progesterone (pessary) /d  48/97 (49.5%)  I2: 400 mg vaginal progesterone (pessary)/d  44/97 (45.4%)  I1 + I2: 92/194 (47.4%) I1, I2, C= NS  I1+I2 vs C= NS I1 vs I2=NS | | Placebo 47/96 (49.0%) | | PO? | | *?* | | *?* | | - | |
| Lim, 2011  The Netherlands AMPHIA | Multifetal pregnancies | | Triplets/+ I: 9 (3%)  C: 9 (3%)  (incl.one quadruplet) MC  I: 57 (17%)  C: 57 (17%)  Fertility treatment I: 140 (42%)  C: 120 (36%) | | I: 336  C: 335 | | 17-OHPC 250 mg im/w 186/336 (55%)  RR 1.11 (95% CI 0.97-1.28)  No p value Subgroup analyses TVS CL <35mm 22/37 (59%)  RR 0.85 (95% CI 0.58-1.26)  No p value TVS CL <25mm  6/9 (67%)  No statistics | | Placebo 165/335 (50%)  Subgroup analyses TVS CL <35mm n=24  17/24 (71%)  TVS CL <25mm 4/4 (100%) | | Not PO  Post hoc subgroup analysis TVS CL <35mm  Prespecified subgroup analysis  <25 mm | | *+* | | *+* | | ? | |
| Wood, 2012 Canada | Twins and triplets | | ART: I: 55%  C: 60%  Triplets: I: 2 (5%)  C: 1(2%) | | I: 42  C: 42 | | 90 mg progesterone vaginal gel 8%/d  25/42 (60%)  RR 0.93 (95% CI 0.66-1.30) p=0.823 | | Placebo 27/42 (64%) | | Not PO | | *+* | | *?* | | + | |

* + No or minor problems

? Some problems

- Major problems

Prevention of preterm birth

STable 4.1.1.a. cont. Intervention progesterone

Outcome variable: Any preterm birth before 37 gestational weeks

| **Author, year Country**  **Trial acronym** | **Singletons/ Twins/ Triplets** | **Risk factor** | | **Number**  **of randomized patients**  **n=** | | **Results** | | | | **Comments** | | **Directness *** | | **Study limitations *** | | **Precision *** | |  |
| --- | --- | --- | --- | --- | --- | --- | --- | --- | --- | --- | --- | --- | --- | --- | --- | --- | --- | --- |
|  |  |  |  |  |  | **Intervention** | | **Control** | |  |  |  |  |  |  |  |  |  |
|  |  | |  | |  | |  | |  | |  | |  | |  | |  | |
| Aboulghar 2012  Egypt | Mixed Singletons 215/306 (70.3%)  DC twins 91/306 (29.7%) | | ART  pregnancies | | RandomizedI:161 C:152  Analyzed I:161  (112 singletons,  49  sets of twins)  C:145  (103 singletons, 42 sets of twins) | | 400 mg vaginal natural progesterone/d  Singleton and twins 55/161 (34.2%)  OR 0.672 (95% CI 0.42-1.0)  Singletons 24/112 (21.4%)  OR 0.53 (95% CI 0.28-0.973)  p= 0.039  Twins 31/49 (63.3%)  OR 0.86 (95% CI 0.36–2) | | Placebo  Singleton and twins 63/145 (43.3%)  Singletons 35/103 (34.0%)  Twins 28/42 (66.7%) | | PO: any PTB (<37 w and <34 w for singletons and twins combined) | | ? | | ? | | - | |
| Cetingoz, 2011 Turkey | Mixed Singletons  I: 41/80 (51.3%)  C: 42/70 (60%)  Twins  I: 39/80 (48.7%)  C: 28/70 (40.0) | | Twin pregnancy, previous sPTD, uterine mal- formation | | I: 84  C: 76 | | 100 mg vaginal progesterone/d Singletons and twins  32/80 (40%)  Singletons 12/41 (29.3%)  RR 0.68 (95% CI 0.38-1.23)  (from MA) Twins  20/39 (51.3%)  RR 0.65 (95% CI 0.45-0.94)  (from MA) | | Placebo Singletons and twins  40/70 (57.2%)  OR 2 (95% CI 1.04–3.83) p=0.036  Singletons: 18/42 (42.9%)  Twins 22/28 (78.6%)  OR 3.48 (95% CI 1.16–10.46) | | PO?  Numbers for singletons calculated from table 3 (no statistics)  OR in article for controls vs intervention. RR for intervention vs controls from MA in App. 5.1.1 and 5.1.2 | | ? | | ? | | - | |

* + No or minor problems

? Some problems

- Major problems

Prevention of preterm birth

STable 4.1.1.a. cont. Intervention progesterone

Outcome variable: Any preterm birth before 37 gestational weeks

| **Author, year Country**  **Trial acronym** | **Singletons/ Twins/ Triplets** | **Risk factor** | | **Number**  **of randomized**  **patients**  **n=** | | **Results** | | | | **Comments** | | **Directness *** | | **Study limitations *** | | **Precision *** | |  |
| --- | --- | --- | --- | --- | --- | --- | --- | --- | --- | --- | --- | --- | --- | --- | --- | --- | --- | --- |
|  |  |  |  |  |  | **Intervention** | | **Control** | |  |  |  |  |  |  |  |  |  |
|  |  | |  | |  | |  | |  | |  | |  | |  | |  | |
| Crowther, 2017 Australia PROGRESS | Mixed Singletons n=775 (98.5%  Twins n=12 (1.5%) | | Previous sPTD  <37 w | | I: 398 (390  singletons and 8 twin pregnancies) 406 infants  C: 389  (385 singletons  and 4 twin pregnancies) 393 infants | | 100 mg vaginal progesterone pessary/d  148/406 (36.5%)  Adj. RR 0.97  (95% CI 0.81–1.17) p=0.765  Adjusted for GA at randomization, GA of previous PTB, and reason for previous PTB  If no twin hade the outcome:  148/390 (37.9%) | | Placebo  146/393 (37.2%)  If no twin hade the outcome: 146/385 (37.9%) | | Not PO  NB neonatal outcome | | + | | + | | ? | |
| **Progesterone in comparison with other interventions** | | | | | | | | | | | | | | | | | | |
| Keeler, 2009a USA | Singletons | | Short TVS CL ≤25  mm in women with | | I: 42  C: 37 | | Cerclage (McDonald) 22/42 (52.4%) | | 17-OHPC 250 mg weekly 22/37 (59.4%) | | Not PO  No indicated PTB | | + | | ? | | - | |
|  |  | | risk factors for | |  | | RR 1.14 (95% CI 0.77-1.68) | |  | | i.e. any PTB= sPTB | |  | |  | |  | |
|  |  | | PTB (history of | |  | | No p value | |  | |  | |  | |  | |  | |
|  |  | | sPTB, second- | |  | | Post hoc analysis | | Post hoc analysis | |  | |  | |  | |  | |
|  |  | | trimester | |  | | TVS CL ≤15 mm | | TVS CL ≤15 mm | |  | |  | |  | |  | |
|  |  | | pregnancy loss, | |  | | 10/22 (45.5%) | | 13/15 (86.7%) | |  | |  | |  | |  | |
|  |  | | previous cervical | |  | | RR 0.52 (95% CI 0.32-0.86) | |  | |  | |  | |  | |  | |
|  |  | | surgery or uterine | |  | | No p-value | |  | |  | |  | |  | |  | |
|  |  | | anomaly) | |  | |  | |  | |  | |  | |  | |  | |
| Dang 2019 | Twins | | Short TVS CL  ≤38 mm | | I:150 C: 150 | | Arabin pessary 73/148 (49%) | | Vaginal progesterone 400 mg/d | | Not PO | | ? | | ? | | ? | |
| Vietnam |  | | (women with | |  | | RR 0.81 (95% CI 0.66-0.99) | | 91/149 (61%) | |  | |  | |  | |  | |
| (single center) |  | | history of cervical | |  | | p=0.05 | |  | |  | |  | |  | |  | |
|  |  | | surgery excluded) | |  | | Subgroup analysis TVS CL | | Subgroup analysis TVS CL | |  | |  | |  | |  | |
|  |  | |  | |  | | ≤28 mm | | ≤28 mm | |  | |  | |  | |  | |
|  |  | |  | |  | | n=47 | | n=35 | |  | |  | |  | |  | |
|  |  | |  | |  | | 23/47 (49%) | | 26/35 (74%) | |  | |  | |  | |  | |
|  |  | |  | |  | | RR 0.66 (95% CI 0.46-0.94) | |  | |  | |  | |  | |  | |
|  |  | |  | |  | | p=0.02 | |  | |  | |  | |  | |  | |

17-OHPC; 17-α-hydroxyprogesterone caproate, ART; assisted reproductive technology, C; control, CL; cervical length, DCDA; dichorionic diamniotic, DA; diamniotic, DC; dichorionic, GA; gestational age, HIV; human immunodeficiency virus I; intervention, im; intramuscular, IUFD; intrauterine fetal death, MA; meta-analysis, MAR; medically assisted reproduction; MC; monochorionic, MCDA; monochorionic diamniotic, NS; not significant, OR; odds ratio, PO; primary outcome, PTB; preterm birth, RR; risk ratio, sPTB: spontaneous preterm birth, TVS; transvaginal scan, w; week

Prevention of preterm birth

STable 4.1.1.b. Intervention progesterone

Outcome variable: Spontaneous preterm birth before 37 gestational weeks

* + No or minor problems

? Some problems

- Major problems

| **Author, year Country**  **Trial acronym** | **Singletons/ Twins/ Triplets** | **Risk factor** | **Number of**  **randomizedpatients**  **n=** | **Results** | | **Comments** | **Directness *** | **Study limitations *** | **Precision *** |
| --- | --- | --- | --- | --- | --- | --- | --- | --- | --- |
|  |  |  |  | **Intervention** | **Control** |  |  |  |  |
|  |  |  |  |  |  |  |  |  |  |
| Aflatoonian, 2013 Iran | Singletons | ART  pregnancies | I: 52  C: 47 | 17-OHPC 250 mg im/w 4/52 (7.7%)  No p value | Placebo 5/47 (10.6%) | Not PO | ? | - | - |
| Blackwell, 2020 USA PROLONG | Singletons | Previous singleton sPTB | I: 1130  C: 578 | 17-OHPC 250 mg im/w 209/1112 (18.8%)  RR 1.10 (95% CI 0.88-1.36)  No p value | Placebo 98/572 (17.1%) | Not PO | + | ? | ? |
| Glover, 2011 USA | Singletons | Previous singleton sPTB (<37 w) | I: 20 C:16 | 400 mg oral micronized progesterone/d 5/19 (26.3%)  RR 0.55 (95% CI 0.26-1.16)  p=0.15 | Placebo 8/14 (57.1%) | PO | + | ? | - |
| Grobman, 2012 USA  SCAN | Singletons | Nulliparous with short TVS CL  <30 mm | I: 327  C: 330 | 17-OHPC 250 mg im/w 54/327 (16.5%)  RR 0.99 (95% CI 0.70-1.40)  No p value | Placebo (castor oil) 55/330 (16.7%) | Not PO | ? | + | + |
| Meis, 2003 USA | Singletons | Previous sPTB | I:310 C:153  Randomized  2:1 | 17-OHPC 250 mg im/w 90/306 (29.4%)  RR 0.65 (95% CI 0.51-0.83)  No p value | Placebo (castor oil) 69/153 (45.1%) | Not PO | ? | ? | ? |
| Price, 2021 Zambia | Singletons | HIV | I: 399 C:401 | 17-OHPC 250 mg/w  25/393 (6%)  RR 1.0 (95% CI 0.6-1.6)  No p value | Placebo 26/392 (7%) | Not PO | ? | + | ? |
| Van Os, 2015 The Netherlands | Singletons | Short TVS CL  ≤30 mm, no previous PTB | I: 41  C: 39 | 200 mg micronized progesterone vaginal/d 6/41 (15%)  RR 1.17 (95% CI 0.39-3.52)  No p value | Placebo 5/39 (13%) | Not PO | + | ? | - |
| Rehal, 2021  UK (+5 other European countries) | Twins | MC: I: 23%  C: 23% ART: I: 34%  C: 35% | I: 582  C: 587 | 600 mg vaginal progesterone/d 161/413 (39.0%)  OR 1.36 (95% CI 1.00-1.83)  No p value | Placebo 137/402 (34.1%) | Not PO | + | + | ? |

* + No or minor problems

? Some problems

- Major problems

Prevention of preterm birth

STable 4.1.1.b. cont. Intervention progesterone

Outcome variable: Spontaneous preterm birth before 37 gestational weeks

| **Author, year Country**  **Trial acronym** | **Singletons/ Twins/ Triplets** | **Risk factor** | **Number of**  **randomizedpatients**  **n=** | **Results** | | **Comments** | **Directness *** | **Study limitations *** | **Precision *** |
| --- | --- | --- | --- | --- | --- | --- | --- | --- | --- |
|  |  |  |  | **Intervention** | **Control** |  |  |  |  |
|  |  |  |  |  |  |  |  |  |  |
| Serra, 2013 Spain | Twins (DCDA) | MAR I1: 92/96 (95.8%) I2: 94/97 (96.9%) C: 96/97 (99.0%) | I1: 98 I2:98 C: 98 | I1: 200 mg vaginal progesterone/d 48/97 (49.5%)  I2: 400 mg vaginal progesterone/d 44/97 (45.4%)  I1 + I2: 92/194 (47.4%)  I1 vs I2 vs C= NS I1+I2 vs C= NS I1 vs I2=NS  No other statistics presented | Placebo 47/96 (49.0%) | Not PO  Numbers calculated for sPTB, from numbers for indicated PTB in table 2 | ? | ? | - |
| Wood, 2012 Canada | Twins and triplets | ART I: 55%  C: 60%  Triplets I: 2 (5%)  C: 1 (2%) | I: 42  C: 42 | 90 mg progesterone vaginal gel 8%/d  17/42 (40%)  RR 1.04 (95% CI 0.61-1.76) p=0.893 | Placebo 16/42 (38%) | Not PO | + | ? | + |

Prevention of preterm birth

STable 4.1.1.b. cont. Intervention progesterone

Outcome variable: Spontaneous preterm birth before 37 gestational weeks

* + No or minor problems

? Some problems

- Major problems

| **Author, year Country**  **Trial acronym** | **Singletons/ Twins/ Triplets** | **Risk factor** | **Number of**  **randomizedpatients**  **n=** | **Results** | | **Comments** | **Directness *** | **Study limitations *** | **Precision *** |
| --- | --- | --- | --- | --- | --- | --- | --- | --- | --- |
|  |  |  |  | **Intervention** | **Control** |  |  |  |  |
|  | | | | | | | | | |
| **Progesterone in comparison with other intervention** | | | | | | | | | |
| Cruz-Melguizo, 2018 Spain  (27 centers) | Singletons | Short TVS CL ≤25 mm (women with cervical surgery and  ≥3 previous PTBs were excluded) | I: 125  C: 118 | Pessary 27/125 (22%)  RD (rate in progesterone group minus the rate in pessary group)  0.41% (-9.90 to 10.73) p=0.94 | 200 mg vaginal progesterone/d 25 /118 (21%) | Not PO | + | ? | ? |
| Keeler, 2009a USA | Singletons | Short TVS CL ≤25 in women with risk factors for PTB (history of sPTB, second trimester pregnancy loss, previous cervical surgery  or uterine anomaly) | I: 42  C: 37 | Cerclage (McDonald) 22/42 (52.4%)  RR 1.14 (95% CI 0.77-1.68)  No p value Post hoc analysis TVS CL ≤15 mm  10/22 (45.5%)  RR 0.52 (95% CI 0.32-0.86)  No p value | 17-OHPC 250 mg/w  22/37 (59.4%)  Post hoc analysis TVS CL ≤15 mm 13/15 (86.7%) | Not PO  There was no indicated PTB  i.e. any PTB= sPTB | + | ? | - |

17-OHPC;17-α-hydroxyprogesterone caproate, ART; assisted reproductive technology, C; control, CI; confidence interval, CL cervical length, d; day, DCDA; dichorionic diamniotic, HIV; human immunodeficiency virus, I; intervention, im; intramuscular, MAR; medically assisted reproduction, PO; primary outcome, PTB; preterm birth, RD; risk difference, RR; risk ratio, sPTB; spontaneous preterm birth, TVS; transvaginal scan. w; week

Prevention of preterm birth

STable 4.1.2.a. Intervention progesterone

Outcome variable: Any preterm birth before 35 gestational weeks

* + No or minor problems

? Some problems

- Major problems

| **Author, year Country**  **Trial acronym** | **Singletons/ Twins/ Triplets** | **Risk factor** | **Number of**  **randomizedpatients**  **n=** | **Results** | | **Comments Risk factor** | **Directness *** | **Study limitations *** | **Precision *** |
| --- | --- | --- | --- | --- | --- | --- | --- | --- | --- |
|  |  |  |  | **Intervention** | **Control** |  |  |  |  |
|  |  |  |  |  |  |  |  |  |  |
| Blackwell, 2020 USA PROLONG | Singletons | Previous singleton sPTB | I: 1130  C: 578  Randomized  2:1 | 17-OHPC 250 mg im/w 122/1113 (11.0%)  RR 0.95 (95% CI 0.71-1.26)  No p value | Placebo 66/574 (11.5%) | PO | + | ? | ? |
| Grobman, 2012 USA  SCAN | Singletons | Nulliparous with short TVS CL  <30 mm | I: 327  C: 330 | 17-OHPC 250 mg im/w 44/327 (13.5%)  RR 0.84 (95% CI 0.58-1.21)  No p value | Placebo (castor oil) 53/330 (16.1%) | Not PO | ? | + | + |
| Hassan, 2011 USA + 9 other  countries PREGNANT | Singletons | Short TVS CL 10-20 mm (all)  Previous PTB 16% | I: 236  C: 229 | 90 mg vaginal progesterone gel 8%/d 34/235 (14.5%)  RR 0.62 (95% CI 0.42-0.92) p=0.016 | Placebo  52/223 (23.3%) | Not PO | ? | ? | + |
| Jabeen, 2012 Pakistan | Singletons | Previous sPTB | I: 30  C: 30 | 17-OHPC 250 mg im/w 6/30 (20%)  No statistics | Placebo (inert oil) 9/30 (30%) | Not PO | ? | - | - |
| Meis, 2003 USA | Singletons | Previous sPTB | I:310 C:153  Randomized 2:1 | 17-OHPC 250 mg im/w 63/306 (20.6%)  RR 0.67 (95% CI 0.48-0.93)  No p value | Placebo (castor oil) 47/153 (30.7%) | Not PO | ? | ? | ? |
| O’Brien 2007  USA +4 other countries | Singletons | Previous sPTB | I: 332  C: 327 | 90 mg vaginal progesterone gel 8%/d 70/309 (22.7%)  OR 0.9 (95% CI 0.61-1.34)  No p value | Placebo  80/302 (26.5%) | Not PO | ? | ? | ? |
| Briery, 2009 USA | Twins, unselected | 1/3 previous PTB | I: 16  C: 14 | 17-OHPC 250 mg im/w 7 /16 (44%)  p=0.117  No RR or OR | Placebo 11/14 (79%) | PO | ? | ? | - |
| Rouse, 2007 USA SSTARS | Twins (DA) | MC  I: 59/327 (18%)  C: 57/334 (17.1%) | I: 327  C: 334 | 17-OHPC 250 mg im/w 135/325 (41.5%)*  RR 1.1 (95% CI 0.9–1.3) | Placebo 123/330 (37.3%)* | PO  *PTB or fetal death <35 w (fetal death includes miscarriage,  termination of pregnancy, and stillbirth) | + | ? | + |

Prevention of preterm birth

STable 4.1.2.a. cont. Intervention progesterone

Outcome variable: Any preterm birth before 35 gestational weeks

* + No or minor problems

? Some problems

- Major problems

| **Author, year Country**  **Trial acronym** | **Singletons/ Twins/ Triplets** | **Risk factor** | **Number of**  **randomizedpatients**  **n=** | **Results** | | **Comments Risk factor** | **Directness *** | **Study limitations *** | **Precision *** |
| --- | --- | --- | --- | --- | --- | --- | --- | --- | --- |
|  |  |  |  | **Intervention** | **Control** |  |  |  |  |
|  |  |  |  |  |  |  |  |  |  |
| Wood, 2012 Canada | Twins and triplets | ART: I: 55%  C: 60%  Triplets: I: 2 (5%)  C: 1(2%) | I: 42  C: 42 | 90 mg progesterone vaginal gel 8%/d 13/42 (31%)  RR 0.87 (95% CI 0.47-1.59) p=0.817 | Placebo 15/42 (36%) | Not PO | + | ? | + |
| Caritis, 2009 USA SSTARS | Triplets | 30% DC or  unknown chorionicity ART:  70% | I: 71  C: 63 | 17-OHPC 250 mg im/w 59/71 (83.1%)  RR 1.0 (95% CI 0.9–1.1)  No p value | Placebo 53/63 (84.1%) | PO | ? | ? | ? |
| Combs, 2010 USA | Triplets | Trichorionic triamniotic triplets MAR:  I: 90%  C: 84% | I: 56  C: 25 | 17-OHPC 250 mg im/w 43/56 (76%)  RR 0.9 (95% CI 0.7–1.1) p=0.56 | Placebo 21/25 (84%) | Not PO  Errata, corrected 21, in article it was 13 | + | ? | ? |
| **Progesterone in comparison with other interventions** | | | | | | | | | |
| Keeler, 2009a USA | Singletons | Short TVS CL ≤25 in women with risk factors for PTB (history of sPTB, second-  trimester pregnancy loss, previous cervical surgery  or uterine anomaly) | I: 42  C: 37 | Cerclage (McDonald) 16/42 (38.1%)  RR 1.14 (95% CI 0.67-1.93)  No p value Post hoc analysis TVS CL ≤15 mm  7/22 (31.8%)  RR 0.48 (95% CI 0.24-0.97)  No p value | 17-OHPC 250 mg weekly 16/37 (43.2%)  Post hoc analysis TVS CL ≤15 mm 10/15 (66.7%) | Not PO  There was no indicated PTB  i.e. any PTB = sPTB | + | ? | - |

17-OHPC; 17-α-hydroxyprogesterone caproate, ART; assisted reproductive technology, BMI; body mass index, C; control, CI; confidence interval, CL; cervical length, DA; diamniotic, DCDA; dichorionic diamniotic, DC; dichorionic, GA; gestational age, I; intervention, im; intramuscular, IUFD; intrauterine fetal death, MAR; medically assisted reproduction; MC; monochorionic, MCDA; monochorionic diamniotic, OR; odds ratio, PO; primary outcome, PTB; preterm birth, RR; risk ratio, sPTB: spontaneous preterm birth, TVS; transvaginal scan, w; week

Prevention of preterm birth

STable 4.1.2.b. Intervention progesterone

Outcome variable: Spontaneous preterm birth before 35 gestational weeks

* + No or minor problems

? Some problems

- Major problems

| **Author, year Country**  **Trial acronym** | **Singletons/ Twins/ Triplets** | **Risk factor** | **Number**  **of randomized**  **patients**  **n=** | **Results** | | **Comments** | **Directness *** | **Study limitations *** | **Precision *** |
| --- | --- | --- | --- | --- | --- | --- | --- | --- | --- |
|  |  |  |  | **Intervention** | **Control** |  |  |  |  |
|  |  |  |  |  |  |  |  |  |  |
| Blackwell, 2020 USA PROLONG | Singletons | Previous singleton sPTB | I: 1130  C: 578  Randomized  2:1 | 17-OHPC 250 mg im/w 93/1113 (8.4%)  RR 0.93 (95% CI 0.67-1.30)  No p value | Placebo 51/574 (8.9%) | Not PO | + | ? | ? |
| Rouse, 2007 USA SSTARS | Twins (DA) | MC  I: 59/327 (18%)  C: 57/334 (17.1%) | I: 327  C: 334 | 17-OHPC 250 mg im/w 101/324 (31.2)*  RR 1.2 (95% CI 0.9-1.5)  No p value | Placebo 86/330 (26.1)* | Not PO  *sPTB or fetal death <35 w (fetal death includes miscarriage, termination of pregnancy, and  stillbirth) | + | ? | + |
| Caritis, 2009 USA STTARS | Triplets | Around 70% ART, 30% DC or unknown chorionicity | I: 71  C: 63 | 17-OHPC 250 mg im/w 34/71 (48%)  RR 1.1 (95% CI 0.8-1.6)  No p value | Placebo 27 /63 (43%) | Not PO | ? | ? | ? |
| **Progesterone in comparison with other interventions** | | | | | | | | | |
| Keeler, 2009a USA | Singletons | Short TVS CL ≤25 in women with risk factors for PTB (history of sPTB, second-  trimester pregnancy loss, previous cervical surgery  or uterine anomaly) | I: 42  C: 37 | Cerclage (McDonald) 16/42 (38.1%)  RR 1.14 (95% CI 0.67-1.93)  No p value Post hoc analysis TVS CL ≤15 mm  7/22 (31.8%)  RR 0.48 (95% CI 0.24-0.97)  No p value | 17-OHPC 250 mg im/w 16/37 (43.2%)  Post hoc analysis TVS CL ≤15 mm 10/15 (66.7%) | PO  There was no indicated PTB  i.e. any PTB = sPTB | + | ? | - |

17-OHPC; 17-α-hydroxyprogesterone caproate, ART, assisted reproductive technology, C; control, CI, confidence interval, CL; cervical length, DC; dichorionic, I; intervention, im; intramuscular, MC; monochorionic, PO; primary outcome, PTB; preterm birth, RR; risk ratio, sPTB; spontaneous preterm birth, TVS transvaginal scan, w; week

Prevention of preterm birth

STable 4.1.3.a. Intervention progesterone

Outcome variable: Any preterm birth before 34 gestational weeks

* + No or minor problems

? Some problems

- Major problems

| **Author, year Country**  **Trial acronym** | **Singletons/ Twins/ Triplets** | **Risk factor** | **Number of**  **randomizedpatients**  **n=** | | **Results** | | **Comments Risk factor** | | **Directness *** | | **Study limitations *** | | **Precision *** |
| --- | --- | --- | --- | --- | --- | --- | --- | --- | --- | --- | --- | --- | --- |
|  |  |  |  |  | **Intervention** | **Control** |  |  |  |  |  |  |  |
|  | | | | | | | | | | | | | |
| **Randomized controlled trials** | | | | | | | | | | | | | |
| Aflatoonian, 2013 Iran | Singletons | ART pregnancies | I: 52  C: 47 | 17-OHPC 250 mg im/w 1/52 (1.9%)?  No statistics for <34 w | | Placebo 1/47 (2.1%)? | Incorrect numbers in the article? | ? | | - | | - | |
| Ali, 2020  Egypt | Singletons | Indication for cerclage: previous  second trimester loss,  sPTD (<34 w) or short cervix  (<25 mm) | I: 121 C:121 | 400 mg progesterone vag (pessary)/d 4/97 (4.2%)  p=0.005 | | Placebo 12/75 (16%) | Progesterone was used as an adjuvant after cerclage  NB: PTB 28-34 weeks, i.e. excludes those who stopped treatment before 28 w, those who were lost to follow-up, and  those who had an abortion before 28 w | ? | | ? | | ? | |
| Azargoon, 2016 Iran | Singletons | Previous PTB (<37 w),  uterine malformations | I: 51  C: 52 | 400 mg progesterone supp vag/d 9/50 (18%)  RR 0.43 (95% CI 0.22-0.84) p=0.04 | | Placebo 21/50 (42%)  RR 2.33 (95% CI 1.19–4.58) p=0.009 | Results also for previous PTB and, previous PTB and short TVS CL  RR calculated for I vs C | - | | ? | | - | |
| Da Fonseca, 2003 Brazil | Singletons | Previous sPTB, prophylactic cerclage, uterine malformations | I: 81 C:76 | 100 mg vaginal progesterone/d 2/72 (2.8%)  p=0.002 | | Placebo 13/70 (18.6%) | Not PO | ? | | ? | | ? | |
| Grobman, 2012 USA  SCAN | Singletons | Nulliparous with short TVS CL  <30 mm | I: 327  C: 330 | 17-OHPC 250 mg im/w 41/327 (12.5%)  RR 0.86 (95% CI 0.58-1.27) p=0.45  TVS CL <15 mm  RR 0.86 (95% CI 0.44-1.67)  TVS CL ≥15 mm  RR 0.91 (95% CI 0.58-1.42)  Interaction p value 0.82 | | Placebo (castor oil) 48/330 (14.5%) | Not PO  RR and p-values for all TVS CLs calculated | ? | | + | | + | |
| Hayashi, 2021 Japan TROPICAL | Singletons | Short TVS CL 25-<30 mm  Previous PTB I: 11.9%  C: 16.7% | I: 59  C: 60 | 200 mg vaginal progesterone/d  0/59 (0%) p=1.0  No RR | | Placebo 1/60 (1.7%) | Not PO | ? | | ? | | - | |

* + No or minor problems

? Some problems

- Major problems

Prevention of preterm birth

STable 4.1.3.a. cont. Intervention progesterone

Outcome variable: Any preterm birth before 34 gestational weeks

| **Author, year Country**  **Trial acronym** | **Singletons/ Twins/ Triplets** | **Risk factor** | **Number of**  **randomizedpatients**  **n=** | **Results** | | | **Comments Risk factor** | | **Directness *** | | **Study limitations *** | | **Precision *** |
| --- | --- | --- | --- | --- | --- | --- | --- | --- | --- | --- | --- | --- | --- |
|  |  |  |  | **Intervention** | | **Control** |  |  |  |  |  |  |  |
|  |  |  |  | |  |  |  |  | |  | |  | |
| Majhi, 2009 India | Singletons | Previous sPTB | I: 50  C: 50 | | 100 mg vaginal progesterone/d 2/50 (4%)  RR 0.666  (95% CI 0.116–3.82)  p= 0.64 | No placebo 3/50 (6%) | PO≤34 w  (PO also <37 w) | ? | | ? | | ? | |
| Norman, 2016 UK  (UK 65 hospitals,  Sweden 1 hospital) OPPTIMUM | Singletons | FFN pos group: Any of previous PTB, second trimester loss, cervical surgery FFN neg group: previous sPTB  <34 w or short TVS CL  ≤25 mm | I: 618  C: 610 | | 200 mg progesterone/day  96/600 (16%)  aOR 0.86 (95% CI 0.61-1.22) p=0.67  CI for OR and p value adjusted for multiple primary outcomes | Placebo 108/597 (18%) | PO = <34 w or IUFD | + | | + | | + | |
| Price, 2021 Zambia | Singletons | HIV | I: 399 C:401 | | 17-OHPC 250 mg/w  14/399 (4%)  RR 0.9 (95% CI 0.4-1.8)  No p value | Placebo 16/401 (4%) | Not PO | ? | | + | | ? | |
| Rai, 2009  India | Singletons | Previous sPTB | I: 75 C:75 | | 200 micronized oral progesterone/d 22/74 (29.7%)  No RR, OR or p value | Placebo  37/74 (50%) |  | - | | ? | | ? | |
| Saghafi, 2011 Iran | Singletons | Previous PTB | I: 50  C: 50 | | 17-OHPC 250 mg im/w 8/50 (16%)  No RR, OR or p value | No placebo 18/50 (36%) | Not stated but assumed to be only singletons | - | | - | | - | |
| Van Os, 2015 The Netherlands TRIPLE P | Singleton | Short TVS CL  ≤30 mm,  no previous PTB | I: 41  C: 39 | | 200 mg vaginal micronized progesterone/d  5/41 (12%)  RR 0.81 (95% CI 0.27–2.44)  No p value | Placebo 6/39 (15%) | Not PO | + | | ? | | - | |
| Briery, 2009 USA | Twins, unselected | 1/3 previous PTB | I: 16  C: 14 | | 17-OHPC 250 mg im/w 5/16 (31.3%)  p=0.217  No RR or OR | Placebo 8/14 (57.1%) | Not PO | ? | | ? | | - | |

* + No or minor problems

? Some problems

- Major problems

Prevention of preterm birth

STable 4.1.3.a. cont. Intervention progesterone

Outcome variable: Any preterm birth before 34 gestational weeks

| **Author, year Country**  **Trial acronym** | **Singletons/ Twins/ Triplets** | **Risk factor** | **Number of**  **randomizedpatients**  **n=** | **Results** | | | **Comments Risk factor** | | **Directness *** | | **Study limitations *** | | **Precision *** |
| --- | --- | --- | --- | --- | --- | --- | --- | --- | --- | --- | --- | --- | --- |
|  |  |  |  | **Intervention** | | **Control** |  |  |  |  |  |  |  |
|  |  |  |  | |  |  |  |  | |  | |  | |
| Brizot, 2015 Brazil | Twins DA | MC 25% (I) and 19% (C) Only naturally conceived,  no history of PTB | I: 195  C: 195 | | 200 mg vaginal natural progesterone/d 44/189 (23.3%)  OR 1.45 (95% CI 0.85-2.49)  No p value  Subgroup analysis TVS CL ≤25mm n=22  16/22 (71.7%)  OR 0.80(95% CI 0.1-4.89)  No p value | Placebo 33/191 (17.3%)  Subgroup analysis TVS CL ≤25mm n=13  10/13 (76.9 %) | Not PO | ? | | ? | | - | |
| Combs, 2011 USA | Twins DCDA | 20% fetal reduction ART  I: 66% C:58%  Prior PTB: I: 12%  C: 13% | I: 160  C: 80  Randomized  2:1 | | 17-OHPC 250 mg (in 1 mL castor oil) im/w  31/160 (19%)  RR 1.4 (95% CI 0.7–2.6) p=0.32 | Placebo (1 mL castor oil) 11/78 (14%) | Not PO | + | | ? | | ? | |
| Klein, 2011 Subgroup analysis of Rode (PREDICT) 2011  Austria & Denmark | Twins | Short cervix TVS CL  ≤30 mm  ≤10^th^ percentile) or  Previous PTB  <34 w or late miscarriage >12 w | TVS CL  ≤30 mm: I:17 C:30  Previous PTB <34 w  or late miscarriage: I:10  C:18 | | 200 mg vaginal progesterone/d  TVS CL ≤30 mm  5/17 (29.4%)  OR 0.63 (95% CI 0.18-2.23) p=0.47  Previous PTB 3/10 (30.0%)  OR 1.50 (95% CI 0.26-8.64) p=0.65 | Placebo  TVS CL ≤30 mm  12/30 (40%)  Previous PTB 4/18 (22.2%) | PO  Subgroup analysis Assessment of directness and study limitations refer to the original study and not to the subgroups | + | | + | | - | |

* + No or minor problems

? Some problems

- Major problems

Prevention of preterm birth

STable 4.1.3.a. cont. Intervention progesterone

Outcome variable: Any preterm birth before 34 gestational weeks

| **Author, year Country**  **Trial acronym** | **Singletons/ Twins/ Triplets** | **Risk factor** | **Number of**  **randomizedpatients**  **n=** | **Results** | | | **Comments Risk factor** | | **Directness *** | | **Study limitations *** | | **Precision *** |
| --- | --- | --- | --- | --- | --- | --- | --- | --- | --- | --- | --- | --- | --- |
|  |  |  |  | **Intervention** | | **Control** |  |  |  |  |  |  |  |
|  |  |  |  | |  |  |  |  | |  | |  | |
| Megli, 2020 USA  IPD of Rouse, 2007 (MFMU  study) and Combs, 2011(Obstetrix  Trial) trials | Twins DA | Previous PTB | I:34 I:32 | | 17-OHPC 250 mg im/w 10/34 (29.4 %)  p=0.09 | 16/32 (50%) | Not PO  IPD with data from Rouse 2007 and Combs 2011  History of PTB not defined Assessment of directness, study limitations, and precision refer to the original studies and not to the  subgroups | + | | ? | | - | |
| Norman, 2009 UK  (9 hospitals) STOPPIT | Twins | MC twins I: 46/247 C: 45/247  No MA twins | I: 247  C: 247 | | 90 mg vaginal progesterone /day (Crinone)  61/247 (24.7%)  OR 1.36 (95% CI 0.89-2.09) p=0.16 | Placebo 48/247 (19.4%) | PO = <34 w or IUFD (one or both twins)  NB: Numbers slightly different in EPPPIC (only PTB and not IUFD?)  I: 59/245 C: 45/245  RR: 1.28 (95% CI 0.91-1.80) | + | | + | | ? | |
| Rehal, 2021  UK (+5 other European countries) | Twins | MC I: 23%  C: 23% ART  I: 34%  C: 35% | I: 582  C: 587 | | 600 mg vaginal progesterone/d 97/582 (16.7%)*  OR 1.10 (95% CI 0.80-1.51)  No p value 82/567 (14.5%)**  OR 1.28 (95% CI 0.90-1.82)  No p value | Placebo 93/587 (15.8%)*  67/561 (11.9%)** | Not PO  *any birth after randomization to 34 w  **any birth 24-34 w | + | | + | | ? | |
| Rode, 2011 PREDICT  Austria & Denmark | Twins | DA twins MC I:12.9% C: 16.6% MAR  I:46.7% C:47.5% | I: 334  C: 343 | | 200 mg progesterone pessary (Utrogestan)/d  51/334 (15.3%)  OR 0.8 (95% CI 0.5-1.2)  No p value | Placebo pessary 63/341 (18.5%) | PO | + | | + | | + | |

* + No or minor problems

? Some problems

- Major problems

Prevention of preterm birth

STable 4.1.3.a. cont. Intervention progesterone

Outcome variable: Any preterm birth before 34 gestational weeks

| **Author, year Country**  **Trial acronym** | **Singletons/ Twins/ Triplets** | **Risk factor** | **Number of**  **randomizedpatients**  **n=** | | **Results** | | **Comments Risk factor** | | **Directness *** | | **Study limitations *** | | **Precision *** |
| --- | --- | --- | --- | --- | --- | --- | --- | --- | --- | --- | --- | --- | --- |
|  |  |  |  |  | **Intervention** | **Control** |  |  |  |  |  |  |  |
|  |  |  |  |  | |  |  |  | |  | |  | |
| Serra, 2013 Spain | Twins (DCDA) | MAR I1: 92/96 (95.8%) I2: 94/97 (96.9%)  C:96/97 (99.0%) | I1: 98 I2:98 C: 98 | I1: 200 mg vaginal progesterone (pessary) /d  13/97 (13.4%)  I2: 400 mg vaginal progesterone (pessary)/d  10/97 (10.3%)  I1 + I2: 23/194 (11.9%) I1, I2, C= NS  I1+I2 vs C= NS I1 vs I2=NS | | Placebo 13/96 (13.5%) | Not PO | ? | | ? | | - | |
| Aboulghar 2012  Egypt | Mixed Singletons 215/306 (70.3%)  DC twins 91/306 (29.7%) | ART  pregnancies | RandomizedI:161 C:152  Ana-  lyzed I:161 (112  singletons, 49  sets of twins) C:145 (103  singletons, 42 sets of  twins) | 400 mg vaginal natural progesterone/d Singletons and twins  11/161 (8.2%)  OR 0.686 (95% CI 0.3-1.56)  Singletons 7/112 (3.3%)  OR 0.68 (95% CI 0.15-3.11)  No p value Twins  8/49 (16.3%)  OR 0.62 (95% CI 0.2–1.76)  No p value | | Placebo  Singletons and twins 14/145 (9.7%)  Singletons 4/103 (3.9%)  Twins  4 /42 (23.8%) | PO: any PTB (<37 w and <34 w for singletons and twins combined) | ? | | ? | | - | |
| Cetingoz, 2011 Turkey | Mixed Singletons I: 41/80 (51.3%)  C: 42/70 (60%)  Twins I: 39/80 (48.7%)  C: 28/70 (40.0) | Twin pregnancy, previous sPTD, uterine mal- formation | I: 84  C: 76 | 100 mg vaginal progesterone/d Singletons and twins  7/80 (8.8%)  Singletons 3/41 (7.3%)  RR 1.02 (0.22-4.79) p=0.98  Twins 4/39 (10.3%) | | Placebo Singletons and twins  17//70 (24.3%)  OR 3.35 (95% CI 1.3–8.63) p=0.010  Singletons: 3/42 (7.1%)  Twins 7/28 (25%)  OR 2.9 (95% CI (0.76–11.2) P=0.200 | Numbers for singletons calculated from table 3 (no statistics).  RR calculated Not PO | ? | | ? | | - | |

* + No or minor problems

? Some problems

- Major problems

Prevention of preterm birth

STable 4.1.3.a. cont. Intervention progesterone

Outcome variable: Any preterm birth before 34 gestational weeks

| **Author, year Country**  **Trial acronym** | **Singletons/ Twins/ Triplets** | **Risk factor** | **Number of**  **randomizedpatients**  **n=** | **Results** | | | **Comments Risk factor** | | **Directness *** | | **Study limitations *** | | **Precision *** |
| --- | --- | --- | --- | --- | --- | --- | --- | --- | --- | --- | --- | --- | --- |
|  |  |  |  | **Intervention** | | **Control** |  |  |  |  |  |  |  |
|  |  |  |  | |  |  |  |  | |  | |  | |
| Fonseca, 2007 Brazil (multicenter inter- national, UK [5 centers], Chile, Brazil,  Greece) | Mixed Singletons (226,  90%)  Twins (24, 10%, all DA) | Short TVS CL  ≤15 mm | I: 125  C: 125 | | 200 mg vaginal progesterone /day (Utrogestan)  26/125 (20.8%)  aRR 0.60 (0.35-0.94) p=0.02  RR adjusted for maternal age, BMI, smoking status, race, history of preterm  birth, and cervical length at the time of randomization. | Placebo 45/125 (36%) | Not PO  See Stewart, 2021 (EPPPIC) for data on singletons and twins | ? | | ? | | + | |
| **Systematic reviews with individual patient meta-analysis**  (Only articles with results not shown in original articles are included here)  Assessment of Directness, Study limitations and Precision refer to the original articles and not to the subgroups presented below | | | | | | | | | | | | | |
| **EPPPIC, 2021** | **Singletons** | | | | | | |  | |  | |  | |
| Blackwell, 2020 USA PROLONG | Singletons | Previous singleton sPTB | I: 1130  C: 578  Randomized  2:1 | | 17-OHPC 250 mg im/w 89/1112 (8.0%)  RR 1.04 (95% CI 0.74-1.47) | Placebo 44/572 (7.7%) | Not PO | + | | ? | | ? | |
| Crowther, 2017 Australia PROGRESS | Mixed Singletons n=775 (98.5%)  Twins n=12 (1.5%) | Previous sPTD  <37 w | Singletons I: 390  C: 385 | | 100 mg vaginal progesterone pessary/d Singletons  64/390 (16.4%)  RR 1.02 (95% CI 0.74-1.40) | Placebo  Singletons 62/385 (16.1%) | Not PO | + | | + | | ? | |
| Fonseca, 2007 Brazil (multicenter inter- national, UK [5  centers], Chile, Brazil, Greece) | Mixed Singletons (226,  90%)  Twins (24, 10%, all DA) | Short TVS CL  ≤15 mm | Singletons I: 114  C: 112 | | 200 mg vaginal progesterone /day (Utrogestan) Singletons  22/114 (19.3%)  RR 0.57 (95% CI 0.36-0.90) | Placebo  Singletons 38/112 (33.9%) | Not PO | ? | | ? | | + | |
| Glover, 2011 USA | Singletons | Previous singleton sPTB (<37 w) | I: 20 C:16 | | 400 mg oral micronized progesterone/d 3/19 (15.8%)  RR 0. 74 (95% CI 0.17-3.12) | Placebo 3/14 (21.4%) | Not PO | + | | ? | | - | |
| Hassan, 2011 USA + 9 other countries PREGNANT | Singletons | Short TVS CL 10-20 mm (all) Previous PTB 16% | I: 236  C: 229 | | 90 mg vaginal progesterone gel 8%/d 26/235 (11.1%)  RR. 0.57 (95% CI 0.37-0.90) | Placebo  43/223 (19.3%) | Not PO | ? | | ? | | + | |

* + No or minor problems

? Some problems

- Major problems

Prevention of preterm birth

STable 4.1.3.a. cont. Intervention progesterone

Outcome variable: Any preterm birth before 34 gestational weeks

| **Author, year Country**  **Trial acronym** | **Singletons/ Twins/ Triplets** | **Risk factor** | **Number of**  **randomizedpatients**  **n=** | **Results** | | | **Comments Risk factor** | | **Directness *** | | **Study limitations *** | | **Precision *** |
| --- | --- | --- | --- | --- | --- | --- | --- | --- | --- | --- | --- | --- | --- |
|  |  |  |  | **Intervention** | | **Control** |  |  |  |  |  |  |  |
|  |  |  |  | |  |  |  |  | |  | |  | |
| Meis, 2003 USA | Singletons | Previous sPTB | I:310 C:153  Randomized  2:1 | | 17-OHPC 250 mg im/w 49/306 (16.0%)  RR 0.63 (95% CI 0.43-0.91) | Placebo (castor oil) 39/153 (25.5%) | Not PO | ? | | ? | | ? | |
| O’Brien, 2007  USA (+4 other countries) | Singletons | Previous sPTB | I: 332  C: 327 | | 90 mg vaginal progesterone gel 8% /d 49/313 (15.7%)  RR 0.83 (95% CI 0.59-1.17) | Placebo 58/307 (18.9%) | Not PO | ? | | ? | | ? | |
| **EPPPIC, 2021** | **Multifetal pregnancies** | | | | | | | | | | | | |
| Awwad, 2015 Libanon PROGESTWIN | Twins, unselected | ART 75%  MC 17% | I:197 C: 96  Randomized  2:1 | | 17-OHPC 250 mg im/w 44/194 (22.7%)  RR 0.82 (95% CI 0.54–1.24) | Placebo (castor oil) 26/94 (27.7%) | Not PO | + | | + | | ? | |
| Fonseca, 2007 Brazil (multicenter inter- national, UK [5  centers], Chile, Brazil, Greece) | Mixed Singletons (226,  90%)  Twins (24, 10%, all DA) | Short TVS CL  ≤15 mm | Twins I: 11  C: 13 | | 200 mg vaginal progesterone /day (Utrogestan)  Twins 4/11 (36.4%)  RR 0.68 (95% CI 0.27-1.71) | Placebo  Twins 7/13 (53.8%) | Not PO | ? | | ? | | + | |
| Combs, 2010 USA | Triplets | Trichorionic triamniotic triplets MAR  I: 90%  C: 84% | I: 56  C: 25 | | 17-OHPC 250 mg im/w 33/56 (58.9%)  RR 0.82 (95% CI 0.59–1.14) p=0.56 | Placebo 18/25 (72.0%) | Not PO | + | | ? | | ? | |
| Caritis, 2009 USA SSTARS | Triplets | 30% DC or  unknown  chorionicity ART: 70% | I: 71  C: 63 | | 17-OHPC 250 mg im/w 48/71 (67.6%)  RR 1.06 (95% CI 0.83-1.36) | Placebo 40/63 (63.5%) | Not PO | ? | | ? | | ? | |
| Crowther, 2017 Australia PROGRESS | Mixed Singletons n=775 (98.5%)  Twins n=12 (1.5%) | Previous sPTB  <37 w | Twins I: 8  C: 4 | | 100 mg vaginal progesterone pessary/d Twins  4/8 (50%)  RR 1.00 (95% CI 0.30-3.32) | Placebo  Twins 2/4 (50%) | Not PO | + | | + | | ? | |

Prevention of preterm birth

STable 4.1.3.a. cont. Intervention progesterone

Outcome variable: Any preterm birth before 34 gestational weeks

* + No or minor problems

? Some problems

- Major problems

| **Author, year Country**  **Trial acronym** | **Singletons/ Twins/ Triplets** | **Risk factor** | **Number of**  **randomizedpatients**  **n=** | **Results** | | | **Comments Risk factor** | | **Directness *** | | **Study limitations *** | | **Precision *** |
| --- | --- | --- | --- | --- | --- | --- | --- | --- | --- | --- | --- | --- | --- |
|  |  |  |  | **Intervention** | | **Control** |  |  |  |  |  |  |  |
|  |  |  |  | |  |  |  |  | |  | |  | |
| Lim, 2011  The Netherlands AMPHIA | Twins and triplets+ | Triplets/+ I: 9 (3%)  C: 9 (3%)  (incl.one quadruplet) MC  I: 57 (17%)  C: 57 (17%)  Fertility treatment I: 140 (42%)  C: 120 (36%) | I: 336  C: 335 | | 17-OHPC 250 mg im/w 80/336 (23.8%)  RR 1.08 (95% CI 0.81-1.42) | Placebo 73/330 (22.1%) | Not PO | + | | + | | ? | |
| Rouse, 2007 USA SSTARS | Twins (DA) | MC  I: 59/327 (18%)  C: 57/334 (17.1%) | I: 327  C: 334 | | 17-OHPC 250 mg im/w 93/325 (28.6%)*  RR 1.05 (95% CI 0.82-1.34) | Placebo 90/330 (27.3%)* | PO  *in original article PTB or fetal death <35 w (fetal death includes miscarriage, termination of pregnancy, and stillbirth) Unclear whether EPPPIC  included only PTB and not IUFD. | + | | ? | | + | |
| Wood, 2012 Canada | Twins and triplets | ART I: 55%  C: 60%  Triplets I: 2 (5%)  C: 1(2%) | I: 42  C: 42 | | 90 mg progesterone vaginal gel 8%/d 8/42 (19.0%)  RR 0.89 (95% CI 0.38-2.08) | Placebo 9/42 (21.4%) | Not PO | + | | ? | | + | |

Prevention of preterm birth

STable 4.1.3.a. cont. Intervention progesterone

Outcome variable: Any preterm birth before 34 gestational weeks

* + No or minor problems

? Some problems

- Major problems

| **Author, year Country**  **Trial acronym** | **Singletons/ Twins/ Triplets** | **Risk factor** | **Number of**  **randomizedpatients**  **n=** | | **Results** | | **Comments Risk factor** | | **Directness *** | | **Study limitations *** | | **Precision *** |
| --- | --- | --- | --- | --- | --- | --- | --- | --- | --- | --- | --- | --- | --- |
|  |  |  |  |  | **Intervention** | **Control** |  |  |  |  |  |  |  |
|  | | | | | | | | | | | | | |
| **Progesterone in comparison with other interventions** | | | | | | | | | | | | | |
| Dang | Twins | Short TVS-CL | I:150 | Arabin pessary | | Vaginal progesterone 400 | PO | ? | | ? | | ? | |
| 2019 |  | ≤38 mm | C: 150 |  | | mg/d |  |  | |  | |  | |
| Vietnam |  | (women with |  | 24/148 (16%) | |  |  |  | |  | |  | |
| (single center) |  | history of |  | RR 0.73 (95% CI 0.46-1.18) | | 33/149 (22%) |  |  | |  | |  | |
|  |  | cervical surgery |  | p=0.24 | |  |  |  | |  | |  | |
|  |  | excluded) |  | Subgroup analysis TVS CL ≤28 mm | | Subgroup analysis TVS CL |  |  | |  | |  | |
|  |  |  |  | n=47 | | ≤28 mm |  |  | |  | |  | |
|  |  |  |  | 10/47 (21%) | |  |  |  | |  | |  | |
|  |  |  |  | RR 0.47 (95% CI 0.24-0.90) | | n=35 |  |  | |  | |  | |
|  |  |  |  | p=0.03 | | 16/35 (46%) |  |  | |  | |  | |

17-OHPC;17-α-hydroxyprogesterone caproate, aOR; adjusted odds ratio, aRR; adjusted relative risk, ART; assisted reproductive technology, BMI; body mass index, C; control, CL; cervical length, d; day, I; intervention, DA, diamniotic, DCDA; dichorionic diamniotic, FFN; fetal fibronectin, im; intramuscular, IUFD; intrauterine fetal death, MA; monoamniotic, MAR; medically assisted reproduction, MC; monochorionic, NNT; number needed to treat, PO; primary outcome, sPTB; spontaneous preterm birth, RR; risk ratio, TVS; transvaginal scan, w; week

* + No or minor problems

? Some problems

- Major problems

Prevention of preterm birth

STable 4.1.3.b. Intervention progesterone

Outcome variable: Spontaneous preterm birth before 34 gestational weeks

| **Author, year Country**  **Trial acronym** | **Singletons/ Twins/ Triplets** | **Risk factor** | **Number of**  **randomizedpatients**  **n=** | | **Results** | | **Comments Risk factor** | **Directness *** | **Study limitations *** | **Precision *** |  |
| --- | --- | --- | --- | --- | --- | --- | --- | --- | --- | --- | --- |
|  |  |  |  |  | **Intervention** | **Control** |  |  |  |  |  |
|  |  |  | |  |  |  |  |  |  |  | |
| Van Os, 2015 The Netherlands TRIPLE P | Singletons | Short TVS CL  ≤30 mm,  no previous PTB | | I: 41  C: 39 | 200 mg vaginal micronized progesterone/d 3/41 (7%)  RR 0.73 (95% CI 0.17-3.057)  No p value | Placebo 4/39 (10%) | Not PO | + | ? | - | |
| Brizot, 2015 Brazil | Twins DCDA | MC 25% (I) and 19% (C)  Only naturally conceived, no history of PTB | | I: 195  C: 195 | 200 mg vaginal natural progesterone/d 35/189 (18.5%)  OR 1.32 (95% CI 0.24-2.37)  No p value | Placebo  28/191 (14.6%) | Not PO | ? | ? | - | |
| Combs, 2011 USA | Twins DCDA | 20% fetal reduction ART  I: 66%, C:58%  Prior PTB:  I: 12%, C: 13% | | I: 160  C: 80  Randomized  2:1 | 17-OHPC 250 mg (in 1 mL castor oil) im/w  16/160 (10%)  RR 0.9 (95% CI 0.4-1.9) p=0.72 | Placebo (1 mL castor oil) 9/78 (11.5%) | Not PO | + | ? | ? | |
| Rehal, 2021  UK (+5 other European countries) | Twins | MC I: 23%  C: 23% ART  I: 34%  C: 35% | | I: 582  C: 587 | 600 mg vaginal progesterone/d 56/541 (10.4%)*  OR 1.35 (95% CI 0.88-2.05)  No p value | Placebo 44/538 (8.2%)* | PO sPTB 24-34 w | + | + | ? | |
| Rode, 2011 PREDICT  Austria & Denmark | Twins | DA twins MC I:12.9% C: 16.6% MAR  I:46.7% C:47.5% | | I: 334  C: 343 | 200 mg progesterone pessary (Utrogestan)/d  42/334 (12.6%)  OR 0.8 (95% CI 0.5-1.2)  No p value | Placebo pessary 53/341 (15.5%) | Not PO | + | + | + | |
| Fonseca, 2007 Brazil (multicenter inter-  national, UK [5 centers], Chile, Brazil,  Greece) | Mixed Singletons (226, 90%)  Twins (24,  10%, all DA) | Short TVS CL ≤15 mm | | I: 125  C: 125 | 200 mg vaginal progesterone /day (Utrogestan)  24/125 (19.2%)  aRR 0.56 (0.32-0.91), p=0.02  RR adjusted for maternal age, BMI, smoking status, race, history of preterm birth, and cervical length at the time of randomization. | Placebo 43/125 (34.4%) | PO | ? | ? | + | |

Prevention of preterm birth

STable 4.1.3.b. cont. Intervention progesterone

Outcome variable: Spontaneous preterm birth before 34 gestational weeks

* + No or minor problems

? Some problems

- Major problems

| **Author, year Country**  **Trial acronym** | **Singletons/ Twins/ Triplets** | **Risk factor** | **Number of**  **randomizedpatients**  **n=** | | **Results** | | **Comments Risk factor** | **Directness *** | **Study limitations *** | **Precision *** |  |
| --- | --- | --- | --- | --- | --- | --- | --- | --- | --- | --- | --- |
|  |  |  |  |  | **Intervention** | **Control** |  |  |  |  |  |
|  | | | | | | | | | | | |
| **Other interventions in comparison with progesterone** | | | | | | | | | | | |
| Cruz-Melguizo, 2018 | Singletons | Short TVS CL ≤25 mm (women with cervical | | I: 125  C: 118 | Pessary 18/125 (14%) | 200 mg vaginal progesterone/d 17 /118 (14%) | PO | + | ? | ? | |
| Spain |  | surgery and ≥3 previous | |  | RD (rate in progesterone group minus the |  |  |  |  |  | |
| (27 centers) |  | PTBs were excluded) | |  | rate in pessary group) |  |  |  |  |  | |
|  |  |  | |  | -0.01% (95% CI -8.84 to 8.83) |  |  |  |  |  | |
|  |  |  | |  | p=0.99 |  |  |  |  |  | |

17-OHPC;17-α-hydroxyprogesterone caproate, ART; assisted reproductive technology, BMI; body mass index, C; control, CL; cervical length, d; day, DCDA; dichorionic diamniotic, DA; diamniotic, DC; dichorionic, GA; gestational age, I; intervention, im; intramuscular, IUFD; intrauterine fetal death, MAR; medically assisted reproduction; MC; monochorionic, MCDA; monochorionic diamniotic, OR; odds ratio, PTB; preterm birth, RD; risk difference, RR; risk ratio, sPTB: spontaneous preterm birth, TVS; transvaginal scan, w; week

* + No or minor problems

? Some problems

- Major problems

Prevention of preterm birth

STable 4.1.4.a. Intervention progesterone

Outcome variable: Any preterm birth before 33 gestational weeks

| **Author, year Country**  **Trial acronym** | **Singletons/Twins**  **/ Triplets** | **Risk factor** | **Number**  **of randomized**  **patients**  **n=** | **Results** | | **Comments** | **Directness *** | **Study limitations *** | **Precision *** |
| --- | --- | --- | --- | --- | --- | --- | --- | --- | --- |
|  |  |  |  | **Intervention** | **Control** |  |  |  |  |
|  | | | | | | | | | |
| **Randomized controlled trials** | | | | | | | | | |
| Hassan, 2011 USA + 9 other countries PREGNANT | Singletons | Short TVS CL 10-20 mm (all) Previous PTB 16% | I: 236  C: 229 | 90 mg vaginal progesterone gel 8%/d 21/235 (8.9%)  RR 0.55 (95% CI 0.33-0.92) p=0.020 | Placebo 36/223 (16.1%) | PO | ? | ? | + |
| **Systematic reviews with individual patient meta-analysis**  (only articles with results not shown in original articles are included here)  Assessment of Directness, Study limitations and Precision refer to the original articles and not to the subgroups presented below | | | | | | | | | |
| **Romero, 2018** | **Singletons** | | | | | |  | | |
| Cetingoz, 2011 Turkey | Mixed Singletons  I: 41/80 (51.3%)  C: 42/70 (60%)  Twins  I: 39/80 (48.7%)  C: 28/70 (40.0) | Twin pregnancy, previous sPTD, uterine malformation | Singletons with TVS CL  ≤25 mm  I: 4  C: 4 | 100 mg vaginal progesterone/d Singletons  0/4 (0%)  RR 0.33 (95% CI 0.02-6.37) | Placebo Singletons 1/4 (25%) | Not PO  9.6% of all randomized  singletons (8/83) | ? | ? | - |
| Fonseca, 2007 Brazil (multicenter inter- national, UK [5  centers], Chile, Brazil, Greece) | Mixed Singletons (226,  90%)  Twins (24, 10%, all DA) | Short TVS CL  ≤15 mm | Singletons I: 114  C: 112 | 200 mg vaginal progesterone /day (Utrogestan) Singletons  19/114 (16.7%)  RR 0.60 (95% CI 0.36-1.00) | Placebo  Singletons 31/112 (27.7%) | Not PO 100% of all randomized singletons | ? | ? | + |
| Norman, 2016 UK  (UK 65 hospitals,  Sweden 1 hospital) OPPTIMUM | Singletons | FFN pos group: Any of previous PTB, second trimester loss, cervical surgery FFN neg group: previous sPTB  <34 w or short  TVS CL ≤25 mm | Singletons with TVS CL  ≤25 mm I:133 C:118 | 200 mg progesterone/day  29/133 (21.8%)  RR 0.74 (95% CI 0.48-1.12) | Placebo 35/118 (29.7%) | Not PO 20.4% of all randomizedsingletons (251/1228) | + | + | + |

* + No or minor problems

? Some problems

- Major problems

Prevention of preterm birth

STable 4.1.4.a. cont. Intervention progesterone

Outcome variable: Any preterm birth before 33 gestational weeks

| **Author, year Country**  **Trial acronym** | **Singletons/Twins**  **/Triplets** | **Risk factor** | **Number**  **of randomized**  **patients**  **n=** | **Results** | | **Comments** | **Directness *** | **Study limitations *** | **Precision *** |
| --- | --- | --- | --- | --- | --- | --- | --- | --- | --- |
|  |  |  |  | **Intervention** | **Control** |  |  |  |  |
|  |  |  |  |  |  |  |  |  |  |
| O’Brien 2007  USA (+4 other countries) | Singletons | Previous sPTB | Singletons with TVS CL  ≤25 mm  I: 12  C: 19 | 90 mg vaginal progesterone gel 8%/d 1/12 (8.3%)  RR 0.40 (95% CI 0.05-3.13) | Placebo 4/19 (21.1%) | Not PO 4.7% of all randomized  singletons (31/659) | ? | ? | ? |
| **Romero, 2017** | **Twins** | | | | | |  | | |
| Brizot, 2015 Brazil | Twins DA | MC I: 25%C: 19%  Only naturally conceived,  no history of PTB | Twins with TVS CL  ≤25 mm  I: 15  C: 6 | 200 mg vaginal natural progesterone/d  9/15 (60%)  RR 0.90 (95% CI 0.45-1.81) | Placebo 4/6 (66.7%) | Not PO 5.4% of all  randomized twins (21/390) | ? | ? | - |
| Cetingoz, 2011 Turkey | Mixed Singletons  I: 41/80 (51.3%)  C: 42/70 (60%)  Twins  I: 39/80 (48.7%)  C: 28/70 (40.0) | Twin pregnancy, previous sPTD, uterine malformation | Twins with TVS CL  ≤25 mm  I:5  C:2 | 100 mg vaginal progesterone/d 1/5 (20%)  RR 0.51 (95% CI 0.17-1.50) | Placebo 1/2 (50%) | Not PO 10.4% of all  randomized twins (7/67) | ? | ? | - |
| Fonseca, 2007 Brazil (Multicenter inter- national, UK [5  centers], Chile, Brazil, Greece) | Mixed Singletons (226,  90%)  Twins (24, 10%, all DA) | Short TVS CL  ≤15 mm | Twins with TVS CL  ≤15 mm I: 11  C: 13 | 200 mg vaginal progesterone /d (Utrogestan)  3/11 (27.3%)  RR 0.51 (95% CI 0.17-1.50) | Placebo 7/13 (53.8%) | Not PO 100% of all  randomized twins (24/24) | ? | ? | + |
| Rode, 2011  Denmark and Austria PREDICT | Twins (DA) | MC  I: 43/334 (12.9%)  C: 57/343 (16.6%) | Twins with TVS  CL ≤25  I: 7  C: 14 | 200 mg vaginal progesterone/d (pessary)  3/7 (42.9%)  RR 1.20 (95% CI 0.40-3.63) | Placebo 5/14 (35.7%) | Not PO  3.1% of all randomized twins (21/677) | + | + | + |

* + No or minor problems

? Some problems

- Major problems

Prevention of preterm birth

STable 4.1.4.a. cont. Intervention progesterone

Outcome variable: Any preterm birth before 33 gestational weeks

| **Author, year Country**  **Trial acronym** | **Singletons/Twins**  **/ Triplets** | **Risk factor** | **Number**  **of randomized patients**  **n=** | **Results** | | **Comments** | **Directness *** | **Study limitations *** | **Precision *** |
| --- | --- | --- | --- | --- | --- | --- | --- | --- | --- |
|  |  |  |  | **Intervention** | **Control** |  |  |  |  |
|  |  |  |  |  |  |  |  |  |  |
| Serra, 2013 Spain | Twins (DCDA) | MAR I1: 92/96 (95.8%) I2: 94/97 (96.9%)  C: 96/97 (99.0%) | Twins with TVS  CL ≤25  I:5  C:1 | I1: 200 mg vaginal progesterone (pessary) /d  or  I2: 400 mg vaginal progesterone (pessary)/d  3/5 (60%)  0.78 (95% CI 0.27-2.22)  I1 vs I2=NS | Placebo  1/1 (100%) | Not PO  2.1% of all randomized twins (6/290) | ? | ? | - |
| **Romero, 2022**  Updated IPD MA of Romero 2017 | **Twins** | | | | | |  |  |  |
| Rehal, 2021  UK (+5 other European countries) | Twins | MC I: 23%  C: 23% ART I: 34%  C: 35% | Twins with TVS  CL ≤25  I: 9  C: 7 | 600 mg vaginal progesterone/d 1/9 (11.1%)  OR 0.13 (95% CI 0.02-0.84)  No p value | Placebo 6/7 (85.7%) | Not PO | + | + | ? |

C; control, CL; cervical length, d; day. DA; diamniotic, DCDA; diamniotic dichorionic, FFN; fetal fibronectin, im; intramuscular, I; intervention, MAR; medically assisted reproductive medicine, MC; monochorionic, NS; not significant, PO; primary outcome, PTB; preterm birth, RR; risk ratio, sPTB, spontaneous preterm birth, TVS; transvaginal scan, w; week

* + No or minor problems

? Some problems

- Major problems

Prevention of preterm birth

STable 4.1.5.a. Intervention progesterone

Outcome variable: Any preterm birth before 32 gestational weeks

| **Author, year Country**  **Trial acronym** | **Singletons/ Twins/ Triplets** | **Risk factor** | **Number of**  **randomizedpatients**  **n=** | **Results** | | **Comments** | **Directness *** | **Study limitations *** | **Precision *** |
| --- | --- | --- | --- | --- | --- | --- | --- | --- | --- |
|  |  |  |  | **Intervention** | **Control** |  |  |  |  |
|  |  |  |  |  |  |  |  |  |  |
| Blackwell, 2020 USA PROLONG | Singletons | Previous singleton sPTB | I: 1130  C: 578  Randomized 2:1 | 17-OHPC 250 mg im/w 54/1116 (4.8%)  RR 0.92 (95% CI 0.60-1.42)  No p value | Placebo 30/574 (5.2%) | Not PO  NB denominator more than in outcome <37 w | + | ? | ? |
| Grobman, 2012 USA  SCAN | Singletons | Nulliparous with short TVS CL  <30 mm | I: 327  C: 330 | 17-OHPC 250 mg im/w 28/327 (8.6%)  RR 0.88 (95% CI 0.54-1.43)  No p value | Placebo (castor oil) 32/330 (9.7%) | Not PO | ? | + | + |
| Jabeen, 2012 Pakistan | Singletons | Previous sPTB | I: 30  C: 30 | 17-OHPC 250 mg/w  4/30 (13.33%)  No statistics | Placebo (inert oil) 7/30 (23.33%) | Not PO | ? | - | - |
| Meis, 2003 USA | Singletons | Previous sPTB | I:310 C:153  Randomized 2:1 | 17-OHPC 250 mg im/w 35/306 (11.4%)  RR 0.58 (95% CI 0.37-0.91)  No p value | Placebo (castor oil) 30/153 (19.6%) | Not PO | ? | ? | ? |
| O’Brien 2007  USA (+4 other countries) | Singletons | Previous sPTB | I: 332  C: 327 | 90 mg vaginal progesterone gel 8%/d 31/309 (10.0%)  OR 0.9 (95% CI 0.52-1.56)  No p value | Placebo  34/302 (11.3%) | PO ≤32 w | ? | ? | ? |
| Rai, 2009  India | Singletons | Previous sPTB | I: 75 C:75 | 200 micronized oral progesterone/d  2/74 (2.7%)  No RR, OR or p value | Placebo  18/74 (24.3%) | Not PO | - | ? | ? |
| Van Os, 2015 The Netherlands TRIPLE P | Singleton | Short TVS CL  ≤30 mm,  no previous PTB | I: 41  C: 39 | 200 mg vaginal micronized progesterone/d 3/41 (7%)  RR 0.58 (95% CI 0.14-2.30)  No p value | Placebo 5/39 (13%) | Not PO | + | ? | - |
| Awwad, 2015 Libanon PROGESTWIN | Twins, unselected | ART 75%  MC 17% | I:197 C: 96  Randomized 2:1 | 17-OHPC 250 mg im/w 18/194 (9.3%)  RR 0.5 (95% CI 0.3-1.1) p=0.09 | Placebo (castor oil) 15/94 (16.0%) | Not PO | + | + | ? |
| Brizot, 2015 Brazil | Twins DA | MC 25% (I) and 19% (C) Only naturally conceived,  no history of PTB | I: 195  C: 195 | 200 mg vaginal natural progesterone/d 22/189 (11.6%)  OR 1.34 (95% CI 0.65-2.80)  No p value | Placebo 17/191 (8.9%) | Not PO | ? | ? | - |

* + No or minor problems

? Some problems

- Major problems

Prevention of preterm birth

STable 4.1.5.a. cont. Intervention progesterone

Outcome variable: Any preterm birth before 32 gestational weeks

| **Author, year Country**  **Trial acronym** | **Singletons/ Twins/ Triplets** | **Risk factor** | **Number of**  **randomizedpatients**  **n=** | **Results** | | **Comments** | **Directness *** | **Study limitations *** | **Precision *** |
| --- | --- | --- | --- | --- | --- | --- | --- | --- | --- |
|  |  |  |  | **Intervention** | **Control** |  |  |  |  |
|  |  |  |  |  |  |  |  |  |  |
| Combs, 2011 USA | Twins DCDA | 20% fetal reduction ART  I: 66% C:58%  Prior PTB: I: 12%  C: 13% | I: 160  C: 80  Randomized 2:1 | 17-OHPC 250 mg (in 1 mL castor oil) im/w  15/160 (9%)  RR 1.8 (95% CI 0.6–5.3) p=0.32 | Placebo (1 mL castor oil) 4/78 (5%) | Not PO | + | ? | ? |
| Rehal, 2021  UK (+5 other European countries) | Twins | MC I: 23%  C: 23% ART I: 34%  C: 35% | I: 582  C: 587 | 600 mg vaginal progesterone/d 53/582 (9.1%)*  OR 0.81 (95% CI 0.55-1.20) No p value 38/567 (6.7%)**  OR 0.97 (95% CI 0.61-1.55)  No p value | Placebo 65/587 (11.1%)*  39/561 (7.0%)** | Not PO  *any birth after randomization to 32 w  **any birth 24-32 w | + | + | ? |
| Rode, 2011 PREDICT  Austria & Denmark | Twins | DA twins MC I:12.9% C: 16.6% MAR  I:46.7% C:47.5% | I: 334  C: 343 | 200 mg progesterone pessary (Utrogestan)/d 24/334 (7.2%)  OR 0.8 (95% CI 0.4-1.3)  No p value | Placebo pessary 31/341 (9.1%) | Not PO | + | + | + |
| Rouse, 2007 USA SSTARS | Twins (DA) | MC  I: 59/327 (18%)  C: 57/334 (17.1%) | I: 327  C: 334 | 17-OHPC 250 mg im/w 55/325 (16.9%)*  RR 1.2 (95% CI 0.8–1.7) | Placebo 48/330 (14.5%)* | *PTB or fetal death <32 weeks  (fetal death includes miscarriage, termination of pregnancy, and stillbirth) | + | ? | + |
| Serra, 2013 Spain | Twins (DCDA) | MAR  I1: 92/96 (95.8%)  I2: 94/97 (96.9%)  C:96/97 (99.0%) | I1: 98 I2:98 C: 98 | I1: 200 mg vaginal progesterone (pessary)  /d 3/97 (3.1%)  I2: 400 mg vaginal progesterone (pessary)/d  7/97 (7.2%)  I1 + I2: 10/194 (5.2%)  I1 vs I2 vs C= NS  I1+I2 vs C= NS I1 vs I2=NS | Placebo 6/96 (6.3%) | Not PO | ? | ? | - |

* + No or minor problems

? Some problems

- Major problems

Prevention of preterm birth

STable 4.1.5.a. cont. Intervention progesterone

Outcome variable: Any preterm birth before 32 gestational weeks

| **Author, year Country**  **Trial acronym** | **Singletons/ Twins/ Triplets** | **Risk factor** | **Number of**  **randomizedpatients**  **n=** | **Results** | | **Comments** | **Directness *** | **Study limitations *** | **Precision *** |
| --- | --- | --- | --- | --- | --- | --- | --- | --- | --- |
|  |  |  |  | **Intervention** | **Control** |  |  |  |  |
|  |  |  |  |  |  |  |  |  |  |
| Caritis, 2009 USA | Triplets | 30% DC or  unknown chorionicity ART:  70% | I: 71  C: 63 | 17-OHPC 250 mg im/w 29/71 (41%)  RR 1.4 (95% CI 0.8–2.2)  No p value | Placebo 19/63 (30%) | PTB <32 w or fetal loss Not PO | ? | ? | ? |
| Combs, 2010 USA | Triplets | Trichorionic triamniotic triplets MAR  I: 90%  C: 84% | I: 56  C: 25 | 17-OHPC 250 mg im/w 19/56 (34%)  RR 0.7 (95% CI 0.4–1.1) p=0.15 | Placebo 13/25 (52%) | Not PO | + | ? | ? |
| Lim, 2011 | Multifetal | Triplets/+ | I: 336 | 17-OHPC 250 mg im/w | Placebo |  | + | + | ? |
| The Netherlands | pregnancies | I: 9 (3%) | C: 335 | 48/336 (14%) | 34/335 (10%) |  |  |  |  |
| AMPHIA |  | C: 9 (3%) |  | RR 1.37 (95% CI 0.91–2.05) |  |  |  |  |  |
|  |  | (incl.one |  | No p value |  |  |  |  |  |
|  |  | quadruplet) |  |  |  |  |  |  |  |
|  |  | MC |  |  |  |  |  |  |  |
|  |  | I: 57 (17%) |  |  |  |  |  |  |  |
|  |  | C: 57 (17%) |  |  |  |  |  |  |  |
|  |  | Fertility treatment |  |  |  |  |  |  |  |
|  |  | I: 140 (42%) |  |  |  |  |  |  |  |
|  |  | C: 120 (36%) |  |  |  |  |  |  |  |

* + No or minor problems

? Some problems

- Major problems

Prevention of preterm birth

STable 4.1.5.a. cont. Intervention progesterone

Outcome variable: Any preterm birth before 32 gestational weeks

| **Author, year Country**  **Trial acronym** | **Singletons/ Twins/ Triplets** | **Risk factor** | **Number of**  **randomizedpatients**  **n=** | **Results** | | **Comments** | **Directness *** | **Study limitations *** | **Precision *** |
| --- | --- | --- | --- | --- | --- | --- | --- | --- | --- |
|  |  |  |  | **Intervention** | **Control** |  |  |  |  |
|  | | | | | | | | | |
| **Other interventions in comparison with progesterone** | | | | | | | | | |
| Keeler, 2009a USA | Singletons | Short TVS-CL ≤25 in women with risk factors for PTB (history of sPTB, second-  trimester pregnancy loss, previous cervical surgery  or uterine anomaly) | I: 42  C: 37 | Cerclage (McDonald) 15/42 (35.7%)  RR 0.98 (95% CI 0.54-1.79)  No p-value Post hoc analysis TVS CL ≤15 mm  7/22 (31.8%)  RR 0.60 (95% CI 0.27-1.29)  No p-value | 17-OHPC 250 mg weekly 13/37 (35.1%)  Post hoc analysis TVS CL ≤15 mm 8/15 (53.3%) | Not PO  There was no indicated PTB  i.e. any PTB=sPTB | + | ? | - |

17-OHPC;17-α-hydroxyprogesterone caproate, ART; assisted reproductive technology, C; control, CL; cervical length, d; day, DA; diamniotic. DCDA; dichorionic diamniotic, , DC; dichorionic, GA; gestational age, I; intervention, im; intramuscular, IUFD; intrauterine fetal death, MAR; medically assisted reproduction; MC; monochorionic, MCDA; monochorionic diamniotic, OR; odds ratio, PTB; preterm birth, RR; risk ratio, sPTB: spontaneous preterm birth, TVS; transvaginal scan, w: week

Prevention of preterm birth

STable 4.1.5.b. Intervention progesterone

Outcome variable: Spontaneous preterm birth before 32 weeks gestational weeks

* + No or minor problems

? Some problems

- Major problems

| **Author, year Country**  **Trial acronym** | **Singletons/ Twins/ Triplets** | **Risk factor** | **Number of**  **randomized**  **patients**  **n=** | **Results** | | **Comments** | **Directness *** | **Study limitations *** | **Precision *** |
| --- | --- | --- | --- | --- | --- | --- | --- | --- | --- |
|  |  |  |  | **Intervention** | **Control** |  |  |  |  |
|  |  |  |  |  |  |  |  |  |  |
| Blackwell, 2020 USA PROLONG | Singletons | Previous singleton sPTB | I: 1130  C: 578  Randomized  2:1 | 17-OHPC 250 mg im/w 38/1116 (3.4%)  RR 0.88 (95% CI 0.52-1.48)  No p value | Placebo 22/574 (3.8%) | Not PO  NB Different numbers in denominator for different  gestational week. | + | ? | ? |
| Van Os, 2015 The Netherlands TRIPLE P | Singletons | Short TVS CL  ≤30 mm,  no previous PTB | I: 41  C: 39 | 200 mg vaginal micronized progesterone/d  1/41 (2.4%)  RR 0.33 (95% CI 0.035-2.99)  No p value | Placebo 3/39 (7.7%) | Not PO | + | ? | - |
| Rehal, 2021  UK (+5 other European countries) | Twins | MC I: 23%  C: 23% ART I: 34%  C: 35% | I: 582  C: 587 | 600 mg vaginal progesterone/d 25/554 (4.5%)*  OR 1.05 (95% CI 0.59-1.86)  No p value | Placebo 24/546 (4.4%)* | Not PO  *any birth 24-32 w | + | + | ? |
| **Other interventions in comparison with progesterone** | | | | | | | | | |
| Keeler, 2009a USA | Singletons | Short TVS CL  ≤25 mm in women with risk factors for PTB (history of sPTB, second- trimester pregnancy loss, previous cervical surgery  or uterine anomaly) | I: 42  C: 37 | Cerclage (McDonald) 15/42 (35.7%)  RR 0.98 (95% CI 0.54-1.79)  No p-value Post hoc analysis TVS CL ≤15 mm  7/22 (31.8%)  RR 0.60 (95% CI 0.27-1.29)  No p-value | 17-OHPC 250 mg/w  13/37 (35.1%)  Post hoc analysis TVS CL ≤15 mm 8/15 (53.3%) | Not PO  There was no indicated PTB  i.e. any PTB=sPTB | + | ? | - |

17-OHPC; 17-α-hydroxyprogesterone caproate, ART; assisted reproductive technology, d; day, C; control, CI; confidence interval, CL; cervical length, I; intervention, im; intramuscular, MC; monochorionic, NB; nota bene, sPTB; spontaneous preterm birth, OR; odds ratio, RR; risk ratio, TVS; transvaginal scan, w; week

Prevention of preterm birth

STable 4.1.6.a. Intervention progesterone

Outcome variable: Any preterm birth before 28 gestational weeks

* + No or minor problems

? Some problems

- Major problems

| **Author, year Country**  **Trial acronym** | **Singletons/ Twins/ Triplets** | **Risk factor** | **No. of randomizedpatients**  **n=** | | **Results** | | **Comments** | **Directness *** | **Study limitations *** | **Precision *** | |
| --- | --- | --- | --- | --- | --- | --- | --- | --- | --- | --- | --- |
|  |  |  |  |  | **Intervention** | **Control** |  |  |  |  |  |
|  |  |  | |  |  |  |  |  |  |  |  |
| Grobman, 2012 USA  SCAN | Singletons | Nulliparous with short TVS CL  <30 mm | | I: 327  C: 330 | 17-OHPC 250 mg im/w 15/327 (4.6%)  RR 0.69 (95% CI 0.36-1.30)  No p value | Placebo (castor oil) 22/330 (6.7%) | Not PO | ? | + | + |  |
| Hassan, 2011 USA + 9 other  countries PREGNANT | Singletons | Short TVS CL 10-20 mm (all)  Previous PTB 16% | | I: 236  C: 229 | 90 mg vaginal progesterone gel 8%/d 12/235 (5.1%)  RR 0.50 (95% CI 0.25-0.97)  p=0.036 | Placebo 23/223 (10.3%) | Not PO | ? | ? | + |  |
| Hayashi, 2021 Japan TROPICAL | Singletons | Short TVS CL 25-<30 mm  Previous PTB I: 11.9%  C: 16.7% | | I: 59  C: 60 | 200 mg vaginal progesterone/d  0/59 (0%) p=1.0  No RR | Placebo 1/60 (1.7%) | Not PO | ? | ? | - |  |
| O’Brien 2007  USA (+4 other countries) | Singletons | Previous sPTB | | I: 332  C: 327 | 90 mg vaginal progesterone gel 8%/d 10/309 (3.2%)*  OR 1.07 (95% CI 0.38-2.96)  No p value | Placebo 9/302 (3.0%)* | Not PO  * ≤28 w | ? | ? | ? |  |
| Price, 2021 Zambia | Singletons | HIV | | I: 399 C:401 | 17-OHPC 250 mg/w  3/399 (1%)  No statistics | Placebo 5/401 (1%) | Not PO | ? | + | ? |  |
| Rai, 2009  India | Singletons | Previous sPTB | | I: 75 C:75 | 200 micronized oral progesterone/d  0/74 (0%) p=0.25 | Placebo  3/74 (4.1%) | Not PO | - | ? | ? |  |
| Awwad, 2015 Libanon PROGESTWIN | Twins, unselected | ART 75%  MC 17% | | I:197 C: 96  Randomized2:1 | 17-OHPC 250 mg im/w 8/194 (4.1%)  RR 0.5 (95% CI 0.2-1.3) p=0.13 | Placebo (castor oil) 8/94 (8.5%) | Not PO | + | + | ? |  |
| Brizot, 2015 Brazil | Twins DA | MC 25% (I) and 19% (C)  Only naturally conceived, no history of PTB | | I: 195  C: 195 | 200 mg vaginal natural progesterone/d 10/189 (5.3%)  OR 1.72 (95% CI 0.55-5.88)  No p value | Placebo 6/191 (3.1%) | Not PO | ? | ? | - |  |

Prevention of preterm birth

STable 4.1.6.a. cont. Intervention progesterone

Outcome variable: Any preterm birth before 28 gestational weeks

* + No or minor problems

? Some problems

- Major problems

| **Author, year Country**  **Trial acronym** | **Singletons/ Twins/ Triplets** | | **Risk factor** | **No. of randomizedpatients**  **n=** | | **Results** | | **Comments** | **Directness *** | **Study limitations *** | **Precision *** | |
| --- | --- | --- | --- | --- | --- | --- | --- | --- | --- | --- | --- | --- |
|  |  |  |  |  |  | **Intervention** | **Control** |  |  |  |  |  |
|  | |  |  | |  |  |  |  |  |  |  |  |
| Combs, 2011 USA | | Twins DCDA | 20% fetal reduction ART  I: 66%  C: 58%  Prior PTB: I: 12%  C: 13% | | I: 160  C: 80  Randomized  2:1 | 17-OHPC 250 mg (in 1 mL castor oil) im/w  3/160 (2%)  RR 1.5 (95% CI 0.2–14.0) p=1.0 | Placebo (1 mL castor oil) 1/78 (1%) | Not PO | + | ? | ? |  |
| Rehal, 2021  UK (+5 other European countries) | | Twins | MC I: 23%  C: 23% ART  I: 34%  C: 35% | | I: 582  C: 587 | 600 mg vaginal progesterone/d 23/582 (4.0%)*  OR 0.65 (95% CI 0.38-1.13)  No p value 8/567 (1.4%)**  OR 0.89 (95% CI 0.34-2.33)  No p value | Placebo 35/587 (6.0%)*  9/561 (1.6%)** | Not PO  *any birth after randomization to 28 w  **any birth 24-28 w | + | + | ? |  |
| Rode, 2011 PREDICT  Austria & Denmark | | Twins | DA twins MC I:12.9% C:16.% MAR  I:46.% C:47.5% | | I: 334  C: 343 | 200 mg progesterone pessary (Utrogestan)/d  9/334 (2.7%)  OR 1.3 (95% CI 0.5-3.6))  No p value | Placebo pessary 7/341 (2.1%) | Not PO | + | + | + |  |
| Rouse, 2007 USA SSTARS | | Twins (DA) | MC  I: 59/327 (18%)  C: 57/334 (17.1%) | | I: 327  C: 334 | 17-OHPC 250 mg im/w 26/325 (8.0%)*  RR 1.3 (95% CI 0.8-2.3)  No p value | Placebo 20/330 (6.1%)* | Not PO  *PTB or fetal death <28 w (fetal death includes miscarriage, termination of  pregnancy, and stillbirth) | + | ? | + |  |

Prevention of preterm birth

STable 4.1.6.a. cont. Intervention progesterone

Outcome variable: Any preterm birth before 28 gestational weeks

* + No or minor problems

? Some problems

- Major problems

| **Author, year Country**  **Trial acronym** | **Singletons/ Twins/ Triplets** | | **Risk factor** | **Number of randomizedpatients**  **n=** | | **Results** | | **Comments** | **Directness *** | **Study limitations *** | **Precision *** | |
| --- | --- | --- | --- | --- | --- | --- | --- | --- | --- | --- | --- | --- |
|  |  |  |  |  |  | **Intervention** | **Control** |  |  |  |  |  |
|  | |  |  | |  |  |  |  |  |  |  |  |
| Serra, 2013 Spain | | Twins (DCDA) | MAR  I1: 92/96 (95.8%)  I2: 94/97 (96.9%)  C:96/97 (99.0%) | | I1: 98 I2:98 C: 98 | I1: 200 mg vaginal progesterone (pessary)/d  1/97 (1.0%)  I2: 400 mg vaginal progesterone (pessary)/d  3/97 (3.1%)  I1 + I2: 4/194 (2.1%)  I1 vs I2 vs C= NS I1+I2 vs C= NS I1 vs I2=NS | Placebo 1/96 (1.0%) | Not PO | ? | ? | - |  |
| Caritis, 2009 USA | | Triplets | 30% DC or unknown chorionicity  ART: 70% | | I: 71  C: 63 | 17-OHPC 250 mg im/w 7/71 (10%)*  RR 0.9 (95% CI 0.3-2.4)  No p value | Placebo 7/63 (11%)* | Not PO  *PTB <28 w or fetal loss | ? | ? | ? |  |
| Combs, 2010 | | Triplets | Trichorionic triamniotic triplets MAR  I: 90%  C: 84% | | I: 56  C: 25 | 17-OHPC 250 mg im/w 9/56 (16%)  RR 2.0 (95% CI 0.5-8.6) p=0.49 | Placebo 2/25 (8%) | Not PO | + | ? | ? |  |
| Lim, 2011  The Netherlands AMPHIA | | Multifetal pregnancies | Triplets/+ I: 9 (3%)  C: 9 (3%)  (incl.one quadruplet) MC  I: 57 (17%)  C: 57 (17%)  Fertility treatment I: 140 (42%)  C: 120 (36%) | | I: 336  women C: 335  women | 17-OHPC 250 mg im/w 19/336 (6%)  RR 1.04 (95% CI 0.56-1.94)  No p value | Placebo 18/332 (5%) | Not PO | + | + | ? |  |

Prevention of preterm birth

STable 4.1.6.a. cont. Intervention progesterone

Outcome variable: Any preterm birth before 28 gestational weeks

* + No or minor problems

? Some problems

- Major problems

| **Author, year Country**  **Trial acronym** | **Singletons/ Twins/ Triplets** | | **Risk factor** | **No. of randomizedpatients**  **n=** | **Results** | | **Comments** | **Directness *** | **Study limitations *** | **Precision *** | |
| --- | --- | --- | --- | --- | --- | --- | --- | --- | --- | --- | --- |
|  |  |  |  |  | **Intervention** | **Control** |  |  |  |  |  |
|  | | | | | | | | | | |  |
| **Other interventions in comparison with progesterone** | | | | | | | | | | |  |
| Keeler, 2009a USA | | Singletons | Short TVS CL ≤25 in women with risk factors for PTB (history of sPTB, second  trimester pregnancy loss, previous cervical surgery or uterine anomaly) | I: 42  C: 37 | Cerclage (McDonald) 10/42 (23.8%)  RR 0.79 (0.34-1.88)  No p-value Post hoc analysis TVS CL ≤15 mm  5/22 (22.7%)  RR 0.68 (0.24-1.95)  No p-value | 17-OHPC 250 mg weekly 7/37 (18.9%)  Post hoc analysis TVS CL ≤15 mm 5/15 (33.3%) | Not PO  There was no indicated PTB  i.e. any PTB=sPTB | + | ? | - |  |
| Dang 2019  Vietnam (single center) | | Twins | Short TVS CL ≤38  mm  (women with history of cervical surgery excluded) | I: 150  C: 150 | Arabin pessary  9/148 (6%)  RR 1.29 (95% CI 0.50-3.38) p=0.62  Subgroup analysis TVS CL  ≤28 mm n=47 4/47 (9%)  RR 0.74 (95% CI 0.2-2.77) p=0.72 | Vaginal progesterone 400 mg/d  7/149 (5%)  Subgroup analysis TVS CL  ≤28 mm  n=35 4/35 (11%) | Not PO | ? | ? | ? |  |

17-OHPC; 17-α-hydroxyprogesterone caproate, ART; assisted reproductive technology, C; control, CL; cervical length, DCDA; dichorionic diamniotic, d: day, DA; diamniotic, DC; dichorionic, GA; gestational age, I; intervention, im; intramuscular, IUFD; intrauterine fetal death, MAR; medically assisted reproduction; MC; monochorionic, MCDA; monochorionic diamniotic, OR; odds ratio, PTB; preterm birth, RR; risk ratio, sPTB: spontaneous preterm birth, TVS; transvaginal scan. w; week

* + No or minor problems

? Some problems

- Major problems

Prevention of preterm birth
STable 4.1.6.b. Intervention progesterone

Outcome variable: Spontaneous preterm birth before 28 gestational weeks

| **Author, year Country**  **Trial acronym** | **Singletons/ Twins/ Triplets** | **Risk factor** | | **Number of**  **randomizedpatients**  **n=** | **Results** | | | **Comments** | **Directness *** | **Study limitations *** | **Precision *** | |
| --- | --- | --- | --- | --- | --- | --- | --- | --- | --- | --- | --- | --- |
|  |  |  |  |  | **Intervention** | | **Control** |  |  |  |  |  |
|  |  | |  |  | |  |  |  |  |  |  |  |
| Rehal, 2021  UK (+5 other European countries) | Twins | | MC I: 23%  C: 23% ART  I: 34%  C: 35% | I: 582  C: 587 | | 600 mg vaginal progesterone/d 8/567 (1.4%)*  OR 1.15 (95% CI 0.41-3.21)  No p value | Placebo 7/559 (1.3%)* | Not PO  *any birth 24-28 w | + | + | ? |  |
| **Other interventions in comparison with progesterone** | | | | | | | | | | | |  |
| Cruz-Melguizo, 2018  Spain  (27 centers) | Singletons | | Short TVS CL ≤25 mm (women with cervical surgery and ≥3 previous PTBs were excluded) | I: 125  C: 118 | | Pessary 10/125 (8%)  RD (rate in progesterone group minus the rate in pessary group)  0.37%  (95% CI -6.38 to 7.12) p=0.91 | 200 mg vaginal progesterone/d 9 /118 (8%) | Not PO | + | ? | ? |  |
| Keeler, 2009a USA | Singletons | | Short TVS CL ≤25 mm in women with risk factors for PTB (history of sPTB, second-  trimester pregnancy loss, previous cervical surgery  or uterine anomaly) | I: 42  C: 37 | | Cerclage (McDonald) 10/42 (23.8%)  RR 0.79 (95% CI 0.34-1.88)  No p value Post hoc analysis TVS CL ≤15 mm  5/22 (22.7%)  RR 0.68 (95% CI 0.24-1.95)  No p value | 17-OHPC 250 mg/w  7/37 (18.9%)  Post hoc analysis TVS CL ≤15 mm 5/15 (33.3%) | Not PO  There was no indicated PTB  i.e. any PTB=sPTB | + | ? | - |  |

17-OHPC; 17-α-hydroxyprogesterone caproate, ART; assisted reproductive technology, C; control, CI; confidence interval, CL; cervical length, d; day, im; intramuscular, I; intervention, MC; monochorionic, OR; odds ratio, PTB; preterm birth, RD; risk difference, RR; risk ratio, sPTB; spontaneous preterm birth, TVS; transvaginal scan

* + No or minor problems

? Some problems

- Major problems

Prevention of preterm birth
STable 4.1.7. Intervention progesterone

Outcome variable: Gestational age

| **Author, year Country**  **Trial acronym** | **Singletons/ Twins/ Triplets** | **Risk factor** | **Number**  **of randomized**  **patients**  **n=** | **Results** | | **Comments** | **Directness *** | **Study limitations *** | **Precision *** |
| --- | --- | --- | --- | --- | --- | --- | --- | --- | --- |
|  |  |  |  | **Intervention** | **Control** |  |  |  |  |
|  |  |  |  |  |  |  |  |  |  |
| Ali, 2020  Egypt | Singletons | Indication for cerclage: previous  second trimester loss, sPTB (<34 w) or short cervix  (<25 mm) | I: 121 C:121 | 400 mg vaginal progesterone (pessary)/d  Mean ± SD: 37.88 ± 0.81 w n=121  p<0.001 | Placebo  Mean ± SD: 36.98 ± 1.20 w n=121 | Not PO Progesterone was used as an adjuvant after cerclage  NB PTB 28-34 weeks, with spontaneous abortion< 28 weeks = primary outcome was  excluded | ? | ? | ? |
| Ashoush, 2017 Egypt | Singletons | Previous sPTB (<37 w) | I: 106  C: 106 | 400 mg oral progesterone/d Mean ± SD: 35.4±2.7 w n=96  p=0.01 | Placebo  Mean ± SD: 33.9±2.9 w n=91 | Not PO  NB critical comment to article by Katsanevakis, Mol and Thornton concerning recruitment and results  NB High rates of cerclage in both groups | - | ? | - |
| Azargoon, 2016 Iran | Singletons | Previous PTB (<37 w),  uterine malformations | I: 51  C: 52 | 400 mg vaginal progesterone supp /d Mean ± SD: 36.5±3.8 w  n=50 p=0.001 | Placebo  Mean ± SD: 33.6±4.5 w n=50 | Not PO | - | ? | - |
| Da Fonseca, 2003 Brazil | Singletons | Previous sPTB, prophylactic  cerclage, uterine malformations | I: 81 C:76 | 100 mg vaginal progesterone/d Mean ± SD: 36 ±3.3 w  n=72 p=0.029 | Placebo  Mean ± SD: 37±2.8 w n=70 | Not PO | ? | ? | ? |
| Glover, 2011 USA | Singletons | Previous singleton sPTB (<37 w) | I: 20 C:16 | 400 mg oral micronized progesterone/d  Mean ± SD: 37.0±2.7 w n=19  p=0.3 | Placebo  Mean ± SD: 35.9±3.8 w n=14 | Not PO  In abstract: 35.9±2.6 | + | ? | - |
| Grobman, 2012 USA  SCAN | Singletons | Nulliparous with short TVS CL  <30 mm | I: 327  C: 330 | 17-OHPC 250 mg im/w Mean ± SD: 37.6±3.9 n=327  p=0.93 | Placebo (castor oil) Mean ± SD: 37.4±4.3 n=330 | Not PO | ? | + | + |

* + No or minor problems

? Some problems

- Major problems

Prevention of preterm birth

STable 4.1.7. cont. Intervention progesterone Outcome variable: Gestational age at delivery

| **Author, year Country**  **Trial acronym** | **Singletons/ Twins/ Triplets** | **Risk factor** | **Number**  **of randomized patients**  **n=** | **Results** | | **Comments** | **Directness *** | **Study limitations *** | **Precision *** |
| --- | --- | --- | --- | --- | --- | --- | --- | --- | --- |
|  |  |  |  | **Intervention** | **Control** |  |  |  |  |
|  |  |  |  |  |  |  |  |  |  |
| Hayashi, 2021 Japan TROPICAL | Singletons | Short TVS CL 25-<30 mm  Previous PTB I: 11.9%  C: 16.7% | I: 59  C: 60 | 200 mg vaginal progesterone/d Median (range):  38.9 (36.4-41) w n=59  p=0.694 | Placebo Median (range): 39.1 (23.7-41.6) w  n=60 | Not PO | ? | ? | _ |
| Ibrahim, 2010 Egypt | Singletons | Previous PTB | I: 25  C: 25 | 17-OHPC 250 mg/w Mean ± SD: 37.47 ± 1.56 w  n=25 p<0.001 | Placebo (saline)  Mean ± SD: 34.71 ± 2.49 n=25 | Not PO | - | - | - |
| Jafarpour, 2020 Iran | Singletons | Previous PTB | I: 50  C: 50 | 17-OHPC 250 mg/w  Mean ± SD: 35.90 (1.21) n=50  p=0.26 | Routine prenatal care Mean ± SD: 35.3 (2.06) n=50 | Not PO | ? | ? | - |
| O’Brien 2007  USA (+4 other countries) | Singletons | Previous sPTB | I: 332  C: 327 | 90 mg vaginal progesterone gel 8%/d Mean ± SD: 36.6 ±3.8 w  n=309 NS | Placebo  Mean ± SD: 36.6±4.2 w n=302 | Not PO | ? | ? | ? |
| Rai, 2009  India | Singletons | Previous sPTB | I: 75 C:75 | 200 micronized oral progesterone/d  Mean ± SD: 36.1±2.66 w n=74  p<0.001 | Placebo  Mean ± SD: 34.0±3.25 w n=74 | Not PO | - | ? | ? |
| Saghafi, 2011 Iran | Singletons | Previous PTB | I: 50  C: 50 | 17-OHPC 250 mg im/w Mean: 36.94 w  n=50 p=0.011 | No placebo Mean: 35.10 w n=50 | Not PO  Not stated but assumed to be only singletons  No SD or range | - | - | - |
| Shadab, 2018 Pakistan | Singletons | Previous sPTB | I: 66  C: 66 | 17-OHPC 250 mg im/w Mean ± SD: 36.35±1.36 w n=66  No p value | Vitamin B im as placebo Mean ± SD: 34.51±3.33 w n=66 | Not PO | - | - | - |
| Awwad, 2015 Libanon PROGESTWIN | Twins, unselected | ART 75%  MC 17% | I:197 C: 96  Randomized  2:1 | 17-OHPC 250 mg im/w Mean ± SD: 35.1±3.1 w n=194  p=0.21 | Placebo (castor oil) Mean ± SD: 34.6±3.8 w n=94 | Not PO | + | + | ? |

* + No or minor problems

? Some problems

- Major problems

Prevention of preterm birth

STable 4.1.7. cont. Intervention progesterone Outcome variable: Gestational age at delivery

| **Author, year Country**  **Trial acronym** | **Singletons/ Twins/ Triplets** | **Risk factor** | **Number**  **of randomized patients**  **n=** | **Results** | | **Comments** | **Directness *** | **Study limitations *** | **Precision *** |
| --- | --- | --- | --- | --- | --- | --- | --- | --- | --- |
|  |  |  |  | **Intervention** | **Control** |  |  |  |  |
|  |  |  |  |  |  |  |  |  |  |
| Briery, 2009 USA | Twins, unselected | 1/3 previous PTB | I: 16  C: 14 | 17-OHPC 250 mg im/w Mean ± SD: 33.9±4.1 n=16  p=0.19 | Placebo  Mean ± SD: 33.1±2.9 n=14 | Not PO | ? | ? | - |
| Brizot, 2015 Brazil | Twins DA | MC 25% (I) and 19% (C)  Only naturally conceived, no history of PTB | I: 195  C: 195 | 200 mg vaginal natural progesterone/d  Mean ± SD: 35.08±3.19 w n=189  No statistics | Placebo  Mean ± SD: 35.55±2.85 w n=191 | Not PO | ? | ? | - |
| Combs, 2011 USA | Twins DCDA | 20% fetal reduction ART  I: 66% C:58%  Prior PTB: I: 12%  C: 13% | I: 160  C: 80  Randomized 2:1 | 17-OHPC 250 mg (in 1 mL castor oil) im/w  Mean ± SD: 35.3±2.5 w n=160  p=0.10 | Placebo (1 mL castor oil)  Mean ± SD: 35.9±2.3 w n=78 | Not PO | + | ? | ? |
| Norman, 2009 UK  (9 hospitals) STOPPIT | Twins | MC twins I: 46/247 C: 45/247  No MA twins | I: 247  C: 247 | 90 mg vaginal progesterone /day (Crinone)  Mean (SD): 35.4 (3.5) w n=247  mean difference –0.3 (–0.9 to 0.3) w p=0.31 | Placebo  Mean (SD): 35.7 (3) w n=247 | Not PO | + | + | ? |
| Rode, 2011 Denmark and Austria PREDICT | Twins (DA) | MC  I: 43/334 (12.9%)  C: 57/343 (16.6%) | I: 334  C: 343 | 200 mg vaginal progesterone/d (pessary)  Mean ± SD: 252±19.6 d n=334  p=0.43 | Placebo  Mean ± SD: 251±19.1 d n=341 | Not PO | + | + | + |
| Rouse, 2007 USA SSTARS | Twins (DA) | MC  I: 59/327 (18%)  C: 57/334 (17.1%) | I: 327  C: 334 | 17-OHPC 250 mg im/w Mean ± SD: 34.6±3.9 w n=325  No p value | Placebo  Mean ± SD: 34.9±3.6 w n=330 | Not PO | + | ? | + |

Prevention of preterm birth

STable 4.1.7. cont. Intervention progesterone Outcome variable: Gestational age at delivery

* + No or minor problems

? Some problems

- Major problems

| **Author, year Country**  **Trial acronym** | **Singletons/ Twins/ Triplets** | **Risk factor** | **Number**  **of randomized**  **patients**  **n=** | **Results** | | **Comments** | **Directness *** | **Study limitations *** | **Precision *** |
| --- | --- | --- | --- | --- | --- | --- | --- | --- | --- |
|  |  |  |  | **Intervention** | **Control** |  |  |  |  |
|  |  |  |  |  |  |  |  |  |  |
| Serra, 2013 Spain | Twins (DCDA) | MAR  I1: 92/96 (95.8%)  I2: 94/97 (96.9%)  C: 96/97 (99.0%) | I1: 98 I2:98 C: 98 | I1: 200 mg vaginal progesterone (pessary) /d  Mean ± SD: 36 ± 2.2 w n=97  I2: 400 mg vaginal progesterone (pessary)/d  Mean ± SD: 36 ± 2.8 w n=97  I1 + I2: Mean ± SD: 36 ± 0.3 w n=194  No p value | Placebo  Mean ± SD: 36 ± 2.6 w n=96 | Not PO | ? | ? | - |
| Lim, 2011  The Netherlands AMPHIA | Multifetal pregnancies | Triplets/+ I: 9 (3%)  C: 9 (3%)  (incl.one quadruplet) MC  I: 57 (17%)  C: 57 (17%)  Fertility treatment I: 140 (42%)  C: 120 (36%) | I: 336  C: 335 | 17-OHPC 250 mg im/w Mean ± SD: 35.4±3.6 w n=336  p=0.32 | Placebo  Mean ± SD: 35.7±3.8 w n=332 | Not PO | + | + | ? |
| Wood, 2012 Canada | Twins and triplets | ART I: 55%  C: 60%  Triplets: I: 2 (5%)  C: 1(2%) | I: 42  C: 42 | 90 mg vaginal progesterone gel 8%/d Median: 36+3 (IQR, 2+6) w  n=42  Difference 1 day (95% CI 4 to -1 days)  p=0.585 | Placebo  Median: 36+2 (IQR, 3+0) w n=42 |  | + | ? | + |
| Cetingoz, 2011 Turkey | Mixed Singletons I: 41/80 (51.3%)  C: 42/70 (60%)  Twins I: 39/80 (48.7%)  C: 28/70 (40.0) | Twin pregnancy, previous sPTD, uterine malformation | I: 84  C: 76 | 100 mg vaginal progesterone/d Singletons and twins  Mean ± SD: 36w 6d ± 2w 3d n=80  p<0.05 | Placebo Singletons and twins  Mean ± SD: 35w 6d ± 3w 2d n=70 | Not PO | ? | ? | - |

| **Author, year Country**  **Trial acronym** | **Singletons/ Twins/ Triplets** | **Risk factor** | **Number**  **of randomized**  **patients**  **n=** | **Results** | | **Comments** | **Directness *** | **Study limitations *** | **Precision *** |
| --- | --- | --- | --- | --- | --- | --- | --- | --- | --- |
|  |  |  |  | **Intervention** | **Control** |  |  |  |  |
|  |  |  |  |  |  |  |  |  |  |
| eCaritis, 2009 USA SSTARS | Triplets | 30% DC or unknown chorionicity ART: 70% | I: 71  C: 63 | 17-OHPC 250 mg im/w Median: 32.4 (IQR, 30.0, 34.4) w n=71  p=0.527 | Placebo Median: 33.0  (IQR, 31.6, 34.3) w n=63 | Not PO | + | ? | ? |
| Combs, 2010 USA | Triplets | Trichorionic triamniotic triplets MAR  I: 90%  C: 84% | I: 56  C: 25 | 17-OHPC 250 mg im/w Mean ± SD: 31.9±4.1 w n=56  p=0.36 | Placebo  Mean ± SD: 31.8±2.9 w n=25 | Not PO | + | ? | ? |
| **Other interventions in comparison with progesterone** | | | | | | | | | |
| Cruz-Melguizo, 2018 | Singletons | Short TVS CL ≤25 mm (women with cervical | I: 125  C: 118 | Pessary  37.3 w (no SD) | 200 mg vaginal progesterone/d | Not PO | + | ? | ? |
| Spain |  | surgery and ≥3 previous |  | n=125 | 37.5 w (no SD) |  |  |  |  |
| (27 centers) |  | PTBs were excluded) |  | p=0.71 | n=118 |  |  |  |  |
| Keeler, 2009a USA | Singletons | Short TVS CL ≤25 in  women with risk factors | I: 42  C: 37 | Cerclage (McDonald) Mean ± SD: 32.9±6.4 w | 17-OHPC 250 mg/w Mean ± SD: 33.0±5.9) w | Not PO | + | ? | - |
|  |  | for PTB (history of |  | n=42 | n=37 |  |  |  |  |
|  |  | sPTB, second- |  | p=0.96 |  |  |  |  |  |
|  |  | trimester pregnancy loss, |  |  |  |  |  |  |  |
|  |  | previous cervical surgery |  |  |  |  |  |  |  |
|  |  | or uterine anomaly) |  |  |  |  |  |  |  |

* + No or minor problems

? Some problems

- Major problems

Prevention of preterm birth

STable 4.1.7. cont. Intervention progesterone Outcome variable: Gestational age at delivery

| **Author, year Country**  **Trial acronym** | **Singletons/ Twins/ Triplets** | **Risk factor** | **Number**  **of randomized**  **patients**  **n=** | **Results** | | **Comments** | **Directness *** | **Study limitations *** | **Precision *** |
| --- | --- | --- | --- | --- | --- | --- | --- | --- | --- |
|  |  |  |  | **Intervention** | **Control** |  |  |  |  |
|  |  |  |  |  |  |  |  |  |  |
| Dang 2019  Vietnam (single center) | Twins | Short TVS CL ≤38 mm (women with history of cervical surgery excluded) | I:150 C: 150 | Arabin pessary  Median (range) 37 (35.5-37.5) w p=0.50  Subgroup analysis TVS CL ≤28 mm Median (range) 37 (35-37.5) w n=47  p=0.09 | 400 mg vaginal progesterone/d Median (range) 36.5 (34.3-37.5) w  Subgroup analysis TVS CL ≤28 mm Median (range) 34.5 (32.8-36.8) w n=35 | Not PO | ? | ? | ? |

17-OHPC;17-α-hydroxyprogesterone caproate, ART; assisted reproductive technology, C; control, CL; cervical length, d; days, DCDA; dichorionic diamniotic, DA; diamniotic, DC; dichorionic, GA; gestational age, I; intervention, im; intramuscular, IQR; interquartile range, IUFD; intrauterine fetal death, MAR; medically assisted reproduction; MC; monochorionic, MCDA; monochorionic diamniotic, OR; odds ratio, PTB; preterm birth, RR; risk ratio, sPTB: spontaneous preterm birth, TVS; transvaginal scan, w; weeks

* + No or minor problems

? Some problems

- Major problems

Prevention of preterm birth

STable 4.1.8. Intervention progesterone

Outcome variable: Low birth weight (<2500 g)

| **Author, year Country**  **Trial acronym** | **Singletons/ Twins/ Triplets** | **Risk factor** | **Number**  **of randomized**  **patients**  **n=** | **Results** | | **Comments Risk factor** | **Directness *** | **Study limitations *** | **Precision *** |
| --- | --- | --- | --- | --- | --- | --- | --- | --- | --- |
|  |  |  |  | **Intervention** | **Control** |  |  |  |  |
|  | | | | | | | | | |
| **Singletons** | | | | | | | | | |
| Aflatoonian, 2013 Iran | Singletons | ART  pregnancies | I: 52  C: 47 | 17-OHPC 250 mg im/w 2/52 (3.8%)  p=0.41 | Placebo 4/47(8.5%) | Not PO  LBW was not defined | ? | - | - |
| Ashoush, 2017 Egypt | Singletons | Previous sPTB (<37 w) | I: 106  C: 106 | 400 mg oral progesterone/d 29/96 (33.7%)  p=0.003 | Placebo 48/91(52.8%) | Not PO  NB critical comment to article by Katsanevakis, Mol and Thornton concerning recruitment and results  NB High rates of cerclage in both groups | - | ? | - |
| Azargoon, 2016 Iran | Singletons | Previous PTB (<37 w),  uterine malformations | I: 51  C: 52 | 400 mg vaginal progesterone supp/d 20/50 (40 %)  RR 1.65 (95% CI 1.11-2.45) p=0.009 | Placebo 33/50 (66 %) | Not PO | - | ? | - |
| Grobman, 2012 USA  SCAN | Singletons | Nulliparous with short TVS CL  <30 mm | I: 327  C: 330 | 17-OHPC 250 mg im/w 72/323 (22.3%)  RR 0.97 (95% CI 0.73-1.30)  No p value | Placebo (castor oil) 75/328 (22.9%) | Not PO | ? | + | + |
| Hassan, 2011 USA + 9 other countries  PREGNANT | Singletons | Short TVS CL 10-20 mm (all) Previous PTB  16% | I: 236  C: 229 | 90 mg vaginal progesterone gel 8%/d 60/234 (25.6%)  RR 0.83 (95% CI 0.62-1.11) p=0.213 | Placebo 68/220 (30.9%) | Not PO | ? | ? | + |
| Hauth, 1983 USA | Singletons | Women from an active –duty  military population | I: 80  C: 88 | 17-OHPC 1000 mg im/w  6/80 (7.5%) NS | Placebo (castor oil) 8/88 (9.1%) | Not PO (calculated from Table II) | - | - | - |
| Ibrahim, 2010 Egypt | Singletons | Previous PTB | I: 25  C: 25 | 17-OHPC 250 mg/w  5 /25(25%) p=0.04 | Placebo (saline) 10/25 (40%) | Not PO | - | - | - |
| Jabeen, 2012 Pakistan | Singletons | Previous sPTB | I: 30  C: 30 | 17-OHPC 250 mg/w  5/30 (16.7%) p=0.739 | Placebo (inert oil) 6/30 (20%) | Not PO | ? | - | - |

* + No or minor problems

? Some problems

- Major problems

Prevention of preterm birth

STable 4.1.8. cont. Intervention progesterone

Outcome variable: Low birth weight (<2500 g)

| **Author, year Country**  **Trial acronym** | **Singletons/ Twins/ Triplets** | **Risk factor** | **Number**  **of randomized**  **patients**  **n=** | **Results** | | **Comments Risk factor** | **Directness *** | **Study limitations *** | **Precision *** |
| --- | --- | --- | --- | --- | --- | --- | --- | --- | --- |
|  |  |  |  | **Intervention** | **Control** |  |  |  |  |
|  |  |  |  |  |  |  |  |  |  |
| afarpour, 2020 Iran | Singletons | Previous PTB | I: 50  C: 50 | 17-OHPC 250 mg/w  14/50 (28%)  RR 1.56 (95% CI 1.6-2.29) p=0.023  NB in text: 95% CI 1.06-2.29 | Routine prenatal care 25/50 (50%) | Not PO | ? | - | - |
| Meis, 2003 USA | Singletons | Previous sPTB | I:310 C:153  Randomized 2:1 | 17-OHPC 250 mg im/w 82/301 (27.2%)  RR 0.66 (95% CI 0.51-0.87)  No p value | Placebo (castor oil) 62/151 (41.1%) | Not PO | ? | ? | ? |
| Price, 2021 Zambia | Singletons | HIV | I: 399 C:401 | 17-OHPC 250 mg/w  41/395 (10%)  RR 0.9 (95% CI 0.6-1.3)  No p value | Placebo 46/395 (12%) | Not PO | ? | + | ? |
| Van Os, 2015 The Netherlands TRIPLE P | Singletons | Short TVS CL  ≤30 mm, no previous PTB | I: 41  C: 39 | 200 mg micronized progesterone vaginal/d  9/41 (22%)  RR 1.07 (95% CI 0.46-2.48)  No p value | Placebo 8/39 (21%) | Not PO | + | ? | - |
| **Multifetal pregnancies** | | | | | | | | | |
| Awwad, 2015 Libanon PROGESTWIN | Twins, unselected | ART 75%  MC 17% | Randomized 2:1  I: 197 C:96  Analyzed I:194 women/388  infants  C: 94 women/188 infants | 17-OHPC 250 mg im/w 241/383 (62.9%)  RR 0.7 (95% CI 0.5-1.1) p=0.11 | Placebo (castor oil) 127/182 (69.8%) | Not PO | + | + | ? |
| Brizot, 2015 Brazil | Twins DA | MC 25% (I)  and 19% (C) Only naturally conceived,  no history of PTB | I: 195/390 C: 195/390 | 200 mg vaginal natural progesterone/d 234/354 (66.1%)  OR 1.18 (95% CI 0.81-1.71)  No p value | Placebo 235/375 (62.7%) | Not PO | ? | ? | - |

* + No or minor problems

? Some problems

- Major problems

Prevention of preterm birth

STable 4.1.8. cont. Intervention progesterone

Outcome variable: Low birth weight (<2500 g)

| **Author, year Country**  **Trial acronym** | **Singletons/ Twins/ Triplets** | **Risk factor** | **Number**  **of randomized patients**  **n=** | **Results** | | **Comments Risk factor** | **Directness *** | **Study limitations *** | **Precision *** |
| --- | --- | --- | --- | --- | --- | --- | --- | --- | --- |
|  |  |  |  | **Intervention** | **Control** |  |  |  |  |
|  |  |  |  |  |  |  |  |  |  |
| Combs, 2011 USA | Twins DCDA | 20% fetal reduction ART  I: 66% C:58%  Prior PTB: I: 12%  C: 13% | I: 160/320 C: 80/160  Randomized 2:1 | 17-OHPC 250 mg (in 1 mL castor oil) im/w  195/320 (61%)  OR 1.9 (95% CI 1.2-3.1) p=0.009 | Placebo (1 mL castor oil) 70/156 (45%) | Not PO | + | ? | ? |
| Rode, 2011 Denmark and Austria PREDICT | Twins (DA) | MC  I: 43/334 (12.9%)  C: 57/343 (16.6%) | I: 334/668 C: 343/686 | 200 mg vaginal progesterone/d (pessary)  306/659 (46.4%)  OR 0.8 (95% CI 0.6-1.0)  No p value | Placebo 357/677 (52.9%) | Not PO | + | + | + |
| Rouse, 2007 USA SSTARS | Twins (DA) | MC  I: 59/327 (18%)  C: 57/334 (17.1%) | I: 327/654 C: 334/668 | 17-OHPC 250 mg im/w 377/632 (60.0%)  RR 0.9 (95% CI 0.8-1.0)  No p value | Placebo 415/648 (64.0%) | Not PO | + | ? | + |
| Serra, 2013 Spain | Twins (DCDA) | MAR I1: 92/96 (95.8%) I2: 94/97 (96.9%) C: 96/97 (99.0%) | I1: 98/196 I2: 98/196 C: 98/196 | I1: 200 mg vaginal progesterone (pessary) /d  I2: 400 mg vaginal progesterone (pessary)/d  I1: 104/194 (53.6%)  I2: 113/191 (59.2%)  I1+I2: 217/385 (56.4%)  Comparison between groups NS | Placebo 117/190 (61.6%) | Not PO | ? | ? | - |
| Caritis, 2009 USA | Triplets | 30% DC or  unknown chorionicity ART:  70% | I: 71/213 C: 63/183 | 17-OHPC 250 mg im/w 191/212 /91%)  RR 0.9 (95% CI 0.9-1.0)  No p value | Placebo  175/183 (96%) | Not PO | ? | ? | ? |
| Lim, 2011  The Netherlands AMPHIA | Multifetal pregnancies | Triplets/+ I: 9 (3%)  C: 9 (3%)  (incl.one | I: 336 women  /681 infants (654 twins, 27 triplets)  C: 335 women/ | 17-OHPC 250 mg im/w 363/681 (53%)  RR 1.0. (95% CI 0.89–1.13)  No p value | Placebo 355/674 (53%) | Not PO | + | + | ? |

* + No or minor problems

? Some problems

- Major problems

Prevention of preterm birth

STable 4.1.8. cont. Intervention progesterone

Outcome variable: Low birth weight (<2500 g)

| **Author, year Country**  **Trial acronym** | **Singletons/ Twins/ Triplets** | **Risk factor** | **Number**  **of randomized patients**  **n=** | **Results** | | **Comments Risk factor** | **Directness *** | **Study limitations *** | **Precision *** |
| --- | --- | --- | --- | --- | --- | --- | --- | --- | --- |
|  |  |  |  | **Intervention** | **Control** |  |  |  |  |
|  |  |  |  |  |  |  |  |  |  |
|  |  | quadruplet) MC  I: 57 (17%)  C: 57 (17%)  Fertility treatment  I: 140 (42%)  C: 120 (36%) | 680 infants (652 twins,  24 triplets, 4 quads) |  |  |  |  |  |  |
| **Mixed singletons and twins** | | | | | | | | | |
| Aboulghar 2012  Egypt | Mixed Singletons 215/306 (70.3%)  DC twins 91/306  (29.7%) | ART  pregnancies | RandomizedI:161 C:152  Analyzed I:161  (112 singletons, 49 sets of twins [98 twins])  C:145  (103 singletons, 42 sets of twins [84 twins]) | 400 mg vaginal natural progesterone/d  Singletons 5/112 (4.5%)  Twin pregnancies: 29/49 (59.2%)  OR 1.63 (95% CI 0.6-1.9) | Placebo  Singletons 11/103 (10.7%)  Twin pregnancies 31/42 (73.8%) | Not PO  NB denominator for twins is pregnancy level not fetal level | ? | ? | - |
| Fonseca, 2007 Brazil (multicenter inter- national, UK [5 centers], Chile, Brazil, Greece) | Mixed Singletons (226, 90%)  Twins (24,  10%, all DA) | Short TVS CL  ≤15 mm | I: 125 women (114  singletons, 11 twin pregnancies)/136 infants C: 125 women (112  singletons, 13 twin pregnancies)/138 infants | 200 mg vaginal progesterone /d (Utrogestan)  56/136 (41.2%)  RR 0.96 (95% CI 0.69-1.26) p=0.81  aRR 0.97 (95% CI 0.68-1.29) p=0.85 | Placebo 59/138 (42.8%) | Not PO  No separate neonatal outcome for twins | ? | ? | + |

* + No or minor problems

? Some problems

- Major problems

Prevention of preterm birth

STable 4.1.8. cont. Intervention progesterone

Outcome variable: Low birth weight (<2500 g)

| **Author, year Country**  **Trial acronym** | **Singletons/ Twins/ Triplets** | **Risk factor** | **Number**  **of randomized patients**  **n=** | **Results** | | **Comments Risk factor** | **Directness *** | **Study limitations *** | **Precision *** |
| --- | --- | --- | --- | --- | --- | --- | --- | --- | --- |
|  |  |  |  | **Intervention** | **Control** |  |  |  |  |
|  |  |  |  |  |  |  |  |  |  |
| Johnson, 1975, single center, USA | Singletons and twins  Only one twin pregnancy | Two spontaneous abortions immediately before this pregnancy or one preterm birth and one spontaneous abortion immediately before this pregnancy or two preterm births at any  point. | 50 randomized 43/50 Analyzed,  37 included in final analysis  I:18 C:19 | 17-OHPC 250 mg im/w 4/18 (22.2%)  No statistics | Placebo 11/19 (57.9%) | Not stated if PO Calculated from Figure 2 | - | - | - |

* + No or minor problems

? Some problems

- Major problems

Prevention of preterm birth

STable 4.1.8. cont. Intervention progesterone

Outcome variable: Low birth weight (<2500 g)

| **Author, year Country**  **Trial acronym** | **Singletons/ Twins/ Triplets** | **Risk factor** | **Number**  **of randomized patients**  **n=** | **Results** | | **Comments Risk factor** | **Directness *** | **Study limitations *** | **Precision *** |
| --- | --- | --- | --- | --- | --- | --- | --- | --- | --- |
|  |  |  |  | **Intervention** | **Control** |  |  |  |  |
|  | | | | | | | | | |
| **Other interventions in comparison with progesterone** | | | | | | | | | |
| Cruz-Melguizo, 2018 | Singletons | Short TVSCL  ≤25 mm | I: 125  C: 118 | Pessary 32/125 (26%) | 200 mg vaginal progesterone/d | Not PO | + | ? | ? |
| Spain |  | (women with |  | No RR | 25 /118 (21%) |  |  |  |  |
| (27 centers) |  | cervical |  | p=0.38 |  |  |  |  |  |
|  |  | surgery and ≥3 |  |  |  |  |  |  |  |
|  |  | previous PTBs |  |  |  |  |  |  |  |
|  |  | were excluded) |  |  |  |  |  |  |  |
| Dang 2019 | Twins | Short TVS CL  ≤38 mm | I:150 C: 150 | Arabin pessary 143/296 (48%) | Vaginal progesterone 400 mg/d | Not PO | ? | ? | ? |
| Vietnam |  | (women with |  | RR 0.80 (95% CI 0.44-0.84) | 181/298 (61%) |  |  |  |  |
| (single center) |  | history of |  | p<0.001 |  |  |  |  |  |
|  |  | cervical |  | Subgroup analysis |  |  |  |  |  |
|  |  | surgery |  | TVS CL ≤28 mm | Subgroup analysis |  |  |  |  |
|  |  | excluded) |  | n=94 | TVS CL ≤28 mm |  |  |  |  |
|  |  |  |  | 50/94 (53%) | n=70 |  |  |  |  |
|  |  |  |  | RR 0.73 (95% CI 0.22-0.82) | 51/70 (73%) |  |  |  |  |
|  |  |  |  | p=0.02 |  |  |  |  |  |

17-OHPC;17-α-hydroxyprogesterone caproate, aRR; adjusted risk ratio, ART; assisted reproductive technology, C; control, CL; cervical length, DCDA; dichorionic diamniotic, DA; diamniotic, DC; dichorionic, im; intramuscular, I; intervention, IUFD; intrauterine fetal death, MAR; medically assisted reproduction, MC; monochorionic, MCDA; monochorionic diamniotic, NNM; neonatal mortality, NS: not significant, OR; odds ratio, PO: primary outcome, PTB; preterm birth, RR; risk ratio, sPTB: spontaneous preterm birth, TVS; transvaginal scan, w; weeks

Prevention of preterm birth

STable 4.1.9. Intervention progesterone

Outcome variable: Very low birth weight (<1500 g)

* + No or minor problems

? Some problems

- Major problems

| **Author, year Country**  **Trial acronym** | **Singletons/ Twins/ Triplets** | **Risk factor** | **Number**  **of randomized patients**  Prevention of preterm birth  STable 4.1.9. Intervention progesterone  Outcome variable: Very low birth weight (<1500 g)  * + No or minor problems  ? Some problems  - Major problems  **n=** | **Results** | | **Comments Risk factor** | **Directness *** | **Study limitations *** | **Precision *** |
| --- | --- | --- | --- | --- | --- | --- | --- | --- | --- |
|  |  |  |  | **Intervention** | **Control** |  |  |  |  |
|  | | | | | | | | | |
| **Singletons** | | | | | | | | | |
| Azargoon, 2016 Iran | Singletons | Previous PTB (<37 w),  uterine malformations | I: 51  C: 52 | 400 mg vaginal progesterone supp /d 5/50 (10 %)  RR 3.6 (95% CI 1.45-8.94) p=0.002 | Placebo 18/50 (36 %) | Not PO | - | ? | - |
| Grobman, 2012 USA  SCAN | Singletons | Nulliparous with short TVS CL  <30 mm | I: 327  C: 330 | 17-OHPC 250 mg im/w 23/323 (7.1%)  RR 0.81 (95% CI 0.48-1.36) | Placebo (castor oil) 29/328 (8.8%) | Not PO | ? | + | + |
| Hassan, 2011 USA + 9 other  countries PREGNANT | Singletons | Short TVS CL 10-20 mm (all)  Previous PTB 16% | I: 236  C: 229 | 90 mg vaginal progesterone gel 8%/d 15/234 (6.4%)  RR 0.47 (95% CI 0.26-0.85) p=0.010 | Placebo  30/220 (13.6%) | Not PO | ? | ? | + |
| Meis, 2003 USA | Singletons | Previous sPTB | I:310 C:153  Randomized 2:1 | 17-OHPC 250 mg im/w 26/301 (8.6%)  RR 0.62 (95% CI 0.36-1.07)  No p value | Placebo (castor oil) 21/151 (13.9%) | Not PO | ? | ? | ? |
| Price, 2021 Zambia | Singletons | HIV | I: 399 C:401 | 17-OHPC 250 mg/w  7/395 (2%)  RR 1.0 (95% CI 0.4-2.8)  No p value | Placebo 7/395 (2%) | Not PO | ? | + | ? |
| Van Os, 2015 The Netherlands TRIPLE P | Singletons | Short TVS CL  ≤30 mm,  no previous PTB | I: 41  C: 39 | 200 mg micronized progesterone vaginal/d 2/41 (5%)  RR 0.46 (95% CI 0.081-2.62)  No p value | Placebo 4/39 (11%) | Not PO | + | ? | - |
| **Twins** | | | | | | | | | |
| Awwad, 2015 Libanon PROGESTWIN | Twins, unselected | ART 75%  MC 17% | Randomized 2.1  I: 197 C:96  Analyzed I:194 women/388  infants  C: 94 women/188 infants | 17-OHPC 250 mg im/w 29/383 (7.6%)  RR 0.5 (95% CI 0.3-0.9) p=0.01 | Placebo (castor oil) 26/182 (14.3%) | Not PO PNM:  IUFD after 24 w and neonatal deaths <28 d | + | + | ? |

* + No or minor problems

? Some problems

- Major problems

Prevention of preterm birth

STable 4.1.9. cont. Intervention progesterone

Outcome variable: Very low birth weight (<1500 g)

| **Author, year Country**  **Trial acronym** | **Singletons/ Twins/ Triplets** | **Risk factor** | **Number**  **of randomized patients**  **n=** | **Results** | | **Comments Risk factor** | **Directness *** | **Study limitations *** | **Precision *** |
| --- | --- | --- | --- | --- | --- | --- | --- | --- | --- |
|  |  |  |  | **Intervention** | **Control** |  |  |  |  |
|  |  |  |  |  |  |  |  |  |  |
| Brizot, 2015 Brazil | Twins DA | MC 25% (I) and 19%  (C) Only naturally conceived,  no history of PTB | I: 195/390 C: 195/390 | 200 mg vaginal natural progesterone/d 34/354 (9.6%)  OR 0.98 (95% CI 0.53-1.81)  No p value | Placebo 39/375 (10.4%) | Not PO | ? | ? | - |
| Combs, 2011 USA | Twins DCDA | 20% fetal reduction ART  I: 66% C:58%  Prior PTB: I: 12%  C: 13% | I: 160/320 C: 80/160  Randomized 2:1 | 17-OHPC 250 mg (in 1 mL castor oil) im/w 28/320 (9%)  OR 1.8 (95% CI 0.6-5.1) p=0.29 | Placebo (1 mL castor oil) 8/156 (5%) | Not PO | + | ? | ? |
| Rehal, 2021  UK (+5 other European countries) | Twins | MC I: 23%  C: 23% ART I: 34%  C: 35% | I: 582/1164 C: 587/1174 | 600 mg vaginal progesterone/d Pregnancy level 51/569 (9.0%)  OR 1.03 (95% CI 0.68-1.56)  No p value Fetal level 75/1125 (6.7%)  OR 0.93 (95% CI 0.65-1.32)  No p value | Placebo  Pregnancy level 50/565 (8.8%)  Fetal level 76/1113 (6.8%) | Not PO | + | + | ? |
| Rode, 2011 Denmark and  Austria PREDICT | Twins (DA) | MC  I: 43/334 (12.9%)  C: 57/343 (16.6%) | I: 334/668 C: 343/686 | 200 mg vaginal progesterone/d (pessary) 36/659 (5.5%)  OR 0.8 (95% CI 0.4-1.4)  No p value | Placebo 48/677 (7.1%) | Not PO | + | + | + |
| Rouse, 2007 USA SSTARS | Twins (DA) | MC  I: 59/327 (18%)  C: 57/334 (17.1%) | I: 327/654 C: 334/668 | 17-OHPC 250 mg im/w 81/632 (12.9%)  RR 2.0 (95% CI 1.0-3.9)  No p value | Placebo 64/648 (9.9%) | Not PO | + | ? | + |
| Serra, 2013 Spain | Twins (DCDA) | MAR  I1: 92/96 (95.8%)  I2: 94/97 (96.9%)  C: 96/97 (99.0%) | I1: 98/196 I2: 98/196 C: 98/196 | I1: 200 mg vaginal progesterone (pessary) /d I2: 400 mg vaginal progesterone (pessary)/d I1: 9/194 (4.6%)  I2: 13/191 (6.8%)  I1+I2: 22/385 (5.7%)  Comparison between groups NS | Placebo 13/191 (6.8%) | Not PO | ? | ? | - |

* + No or minor problems

? Some problems

- Major problems

Prevention of preterm birth

STable 4.1.9. cont. Intervention progesterone

Outcome variable: Very low birth weight (<1500 g)

| **Author, year Country**  **Trial acronym** | **Singletons/ Twins/ Triplets** | **Risk factor** | **Number**  **of randomized patients**  **n=** | **Results** | | **Comments Risk factor** | **Directness *** | **Study limitations *** | **Precision *** |
| --- | --- | --- | --- | --- | --- | --- | --- | --- | --- |
|  |  |  |  | **Intervention** | **Control** |  |  |  |  |
|  |  |  |  |  |  |  |  |  |  |
| Caritis, 2009 USA | Triplets | 30% DC or unknown chorionicity  ART: 70% | I: 71/213 C: 63/183 | 17-OHPC 250 mg im/w  91/212 (43%)  RR 1.7 (95% CI 1.1-2.7)  No p value | Placebo 46/183 (25%) | Not PO | ? | ? | ? |
| **Mixed singletons and twins** | | | | | | | | | |
| Aboulghar 2012  Egypt | Mixed Singletons 215/306 (70.3%)  DC twins 91/306 (29.7%) | ART  pregnancies | RandomizedI:161 C:152  Analyzed I:161  (112 singletons, 49 sets of twins [98 twins])  C:145  (103 singletons, 42 sets of twins [84 twins]) | 400 mg vaginal natural progesterone/d Singletons  2/112 (1.8%)  OR 1.8 (95% CI 0.16–20.76)  Twins:  2/49 (4.3%)  OR 0.25 (95% CI 0.049-1.34) | Placebo  Singletons 1/103 (1,0%)  Twins 6/42 (14.3%) | Not PO  NB Denominator for twins are pregnancy level not fetal level | ? | ? | - |
| Fonseca, 2007 Brazil (multicenter international, UK [5 centers], Chile, Brazil,  Greece) | Mixed Singletons (226, 90%)  Twins (24,  10%, all DA) | Short TVS CL ≤15  mm | I: 125 women (114  singletons, 11 twin pregnancies)/136 infants  C: 125 women (112  singletons, 13 twin pregnancies)/138 infants | 200 mg vaginal progesterone /d (Utrogestan) 18/136 (13.2%)  RR 0.68 (95% CI 0.36-1.21) p=0.20  aRR 0.74 (95% CI 0.36-1.37) p=0.35 | Placebo 27/138 (19.6%) | Not PO  No separate neonatal outcome for twins | ? | ? | + |
| Lim, 2011  The Netherlands AMPHIA | Multifetal pregnancies | Triplets/+ I: 9 (3%)  C: 9 (3%)  (incl.one quadruplet) MC  I: 57 (17%)  C: 57 (17%)  Fertility treatment I: 140 (42%)  C: 120 (36%) | I: 336 women  /681 infants (654  twins, 27 triplets)  C: 335 women/  680 infants (652 twins,  24 triplets, 4 quads) | 17-OHPC 250 mg im/w 90/681 (13%)  RR 1.25 (95% CI 0.83-1.90)  No p value | Placebo 64/674 (9%) | Not PO | + | + | ? |

Prevention of preterm birth

STable 4.1.9. cont. Intervention progesterone

Outcome variable: Very low birth weight (<1500 g)

* + No or minor problems

? Some problems

- Major problems

| **Author, year Country**  **Trial acronym** | **Singletons/ Twins/ Triplets** | **Risk factor** | **Number**  **of randomized patients**  **n=** | **Results** | | **Comments Risk factor** | **Directness *** | **Study limitations *** | **Precision *** |
| --- | --- | --- | --- | --- | --- | --- | --- | --- | --- |
|  |  |  |  | **Intervention** | **Control** |  |  |  |  |
|  | | | | | | | | | |
| **Other interventions in comparison with progesterone** | | | | | | | | | |
| Cruz-Melguizo, 2018 | Singletons | Short TVS CL ≤25 mm | I: 125  C: 118 | Pessary 10/125 (8%) | 200 mg vaginal progesterone/d | Not PO | + | ? | ? |
| Spain |  | (women with |  | No RR | 10 /118 (8%) |  |  |  |  |
| (27 centers) |  | cervical surgery and |  | p=0.92 |  |  |  |  |  |
|  |  | ≥3 previous PTBs |  |  |  |  |  |  |  |
|  |  | were excluded) |  |  |  |  |  |  |  |
| Dang 2019 | Twins | Short TVS CL ≤38  mm | I:150 C: 150 | Arabin pessary 29/296 (10%) | 400 mg vaginal progesterone/d | Not PO | ? | ? | ? |
| Vietnam |  | (women with history |  | RR 1.17 (0.68-2.08) | 25/298 (8%) |  |  |  |  |
| (single center) |  | of cervical surgery |  | p=0.57 |  |  |  |  |  |
|  |  | excluded) |  | Subgroup analysis | Subgroup analysis |  |  |  |  |
|  |  |  |  | TVS CL ≤28 mm | TVS CL ≤28 mm |  |  |  |  |
|  |  |  |  | n=94 | n=70 |  |  |  |  |
|  |  |  |  | 12/94 (13%) | 12/70 (17%) |  |  |  |  |
|  |  |  |  | RR 0.74 (95% CI 0.30-1.68) |  |  |  |  |  |
|  |  |  |  | p=0.51 |  |  |  |  |  |

17-OHPC;17-α-hydroxyprogesterone caproate, aRR; adjusted risk ratio, ART; assisted reproductive technology, C; control, CL; cervical length, d; days, DCDA; dichorionic diamniotic, DA; diamniotic, DC; dichorionic, im; intramuscular, I; intervention, IUFD; intrauterine fetal death, MAR; medically assisted reproduction, MC; monochorionic, MCDA; monochorionic diamniotic, NNM; neonatal mortality, NS; not significant, OR; odds ratio, PO; primary outcome, PTB; preterm birth, RR; risk ratio, sPTB; spontaneous preterm birth, TVS; transvaginal scan, w; weeks

* + No or minor problems

? Some problems

- Major problems

Prevention of preterm birth

STable 4.1.10. Intervention progesterone Outcome variable: Perinatal mortality

| **Author, year Country**  **Trial acronym** | **Singletons/ Twins/ Triplets** | **Risk factor** | **Number**  **of randomized patients**  **n=** | | **Results** | | | **Comments** | **Directness *** | **Study limitations *** | **Precision *** | |
| --- | --- | --- | --- | --- | --- | --- | --- | --- | --- | --- | --- | --- |
|  |  |  |  |  | **Intervention** | **Control** | |  |  |  |  |  |
|  |  |  |  |  | |  |  | |  |  |  |  |
| Aflatoonian, 2013  Iran | Singletons | ART pregnancies | I: 52  C: 47 | 17-OHPC 250 mg im/w IUFD 0/52 | | Placebo IUFD 1/47 (2.1%) | Not PO  IUFD not defined PNM not reported | | ? | - | - |  |
| Ali, 2020  Egypt | Singletons | Indication for cerclage: previous  second trimester loss, sPTD (<34 w) or short cervix  TVS CL <25 mm | I: 121 C:121 | 400 mg vaginal progesterone (pessary)/d  Abortion before 28 w 21/121 (17.4%) p=0.016 | | Placebo  Abortion before 28 w 37/121 (30.6%) | PO abortion before 28 w Progesterone was used as an adjuvant after cerclage PNM not reported | | ? | ? | ? |  |
| Ashoush, 2017 Egypt | Singletons | Previous sPTB (<37 w) | I: 106  C: 106 | 400 mg oral progesterone/d Mid trimester abortion 7/103 (6.8%)  p=0.46 | | Placebo  Mid trimester abortion 11/102 (10.8%) | Not PO  NB critical comment to article by Katsanevakis, Mol and Thornton concerning recruitment and results  NB High rates of cerclage in both groups  PNM not reported | | - | ? | - |  |
| Blackwell, 2020 USA PROLONG | Singletons | Previous singleton sPTB | I: 1130  C: 578  Randomized 2:1 | 17-OHPC 250 mg im/w IUFD  12/1124 (1.1%)  RR 2.07 (95% CI 0.59-7.29) PNM  15/1128 (1.3%)  No statistics | | Placebo IUFD  3/571 (0.5%)  PNM 4/578 (0.7%) | Not PO IUFD from 20+0 w  Denominator is women pregnant from 20+0 w  PNM defined as IUFD or NNM  <28 d (calculated from Table 4) | | + | ? | ? |  |
| Grobman, 2012 USA  SCAN | Singletons | Nulliparous with short TVS CL  <30 mm | I: 327  C: 330 | 17-OHPC 250 mg im/w Fetal loss/abortion <20 w 1/327 (0.3%)  No statistics IUFD 4/327 (1.2%)  RR 4.04 (95% CI 0.45-35.92)  PNM 10/327 (3.1%)  No statistics | | Placebo (castor oil) Fetal loss/abortion <20 w 0/330  IUFD 1/330 (0.3%)  PNM 9/330 (2.7%) | Not PO  PNM defined as IUFD or NNM (calculated from Table 3) NNM not defined | | ? | + | + |  |

* + No or minor problems

? Some problems

- Major problems

Prevention of preterm birth

STable 4.1.10. cont. Intervention progesterone Outcome variable: Perinatal mortality

| **Author, year Country**  **Trial acronym** | **Singletons/ Twins/ Triplets** | **Risk factor** | **Number**  **of randomized patients**  **n=** | **Results** | | | **Comments** | **Directness *** | **Study limitations *** | **Precision *** |
| --- | --- | --- | --- | --- | --- | --- | --- | --- | --- | --- |
|  |  |  |  | **Intervention** | **Control** | |  |  |  |  |
|  |  |  |  |  |  |  | |  |  |  |
| Hassan, 2011 USA + 9 other countries PREGNANT | Singletons | Short TVS CL 10-20 mm (all)  Previous PTB 16% | I: 236  C: 229 | 90 mg vaginal progesterone gel 8%/d  IUFD 5/235 (2.1%)  RR 0.79 (95% CI 0.25–2.57) p=0.700  PNM 8/235 (3.4%)  RR 0.69 (95% CI 0.28-1.68) p=0.413 | Placebo IUFD 6/223 (2.7%)  PNM 11/223 (4.9%) | Not PO  PNM: IUFD and NNM  IUFD and NNM not defined | | ? | ? | + |
| Hauth, 1983 USA | Singletons | Women from an active –duty military population | I: 80  C: 88 | 17-OHPC 1000 mg im/w IUFD 2/80  PNM 3/88 (3.8%)  No statistics | Placebo (castor oil) IUFD 0/88  PNM 3/88 (3.4%) | Not PO  IUFD or NNM not defined | | - | - | - |
| Jabeen, 2012 Pakistan | Singletons | Previous sPTB | I: 30  C: 30 | 17-OHPC 250 mg/w  PNM 2/30 (6.7%) p=0.228 | Placebo (inert oil) PNM 5/30 (16.7%) | Not PO PNM not defined | | ? | - | - |
| Meis, 2003 USA | Singletons | Previous sPTB | I:310 C:153  Randomized 2:1 | 17-OHPC 250 mg im/w IUFD 6/306 (2.0%)  RR 1.50 (95% CI 0.31–7.34)  NNM 8/306 (2.6%)  RR 0.44 (95% CI 0.17–1.13)  no p-values PNM 14/306 (4.6%)  No statistics | Placebo (castor oil) IUFD 2/153 (1.3%)  NNM 9/153 (5.9%)  PNM 11/153 (7.2%) | Not PO  IUFD or NNM not defined PNM calculated from IUFD+NNM in Table 3 | | ? | ? | ? |
| Norman, 2016 UK  (UK 65  hospitals, Sweden 1 hospital) OPPTIMUM | Singletons | FFN pos group: Any of previous PTB, second trimester loss, cervical surgery  FFN neg group: previous sPTB <34 w or  short TVS CL ≤25  mm | I: 618  C: 610 | 200 mg vaginal progesterone/d IUFD 8/600 (1%)  RR 1.14 (95% CI 0.41-3.17) p=0.8  NNM 1/600 (<1%)  RR 0.17 (95% CI 0.06-0.49) p=0.0009  PNM 9/600 (1.5%)  No statistics | Placebo IUFD 7/597 (1%)  NNM 6/597 (1%)  PNM 13/600 (2.2%) | Not PO  PNM or NNM not defined PNM calculated from IUFD+NNM | | + | + | + |

Prevention of preterm birth

STable 4.1.10. cont. Intervention progesterone Outcome variable: Perinatal mortality

* + No or minor problems

? Some problems

- Major problems

| **Author, year Country**  **Trial acronym** | **Singletons/ Twins/ Triplets** | **Risk factor** | **Number**  **of randomized patients**  **n=** | **Results** | | | **Comments** | **Directness *** | **Study limitations *** | **Precision *** |
| --- | --- | --- | --- | --- | --- | --- | --- | --- | --- | --- |
|  |  |  |  | **Intervention** | **Control** | |  |  |  |  |
|  |  |  |  |  |  |  | |  |  |  |
| O’Brien 2007  USA (+4 other countries) | Singletons | Previous sPTB | I: 332  C: 327 | 90 mg vaginal progesterone gel 8%/d  IUFD <20 w 0/309 IUFD >20 w 5/309 (1.6%)  OR 1.22 (95% CI 0.33- 4.61)  No p value PNM 11/309 (3.6%)  No statistics | Placebo  IUFD <20 w 0/309 IUFD >20 w 4/302 (1.3%)  PNM 11/302 (3.6%) | Not PO  PNM: IUFD >20 and NNM <28  d  Calculated from Table 2 | | ? | ? | ? |
| Van Os, 2015 The Netherlands TRIPLE P | Singletons | Short TVS CL  ≤30 mm,  no previous PTB | I: 41  C: 39 | 200 mg micronized progesterone vaginal/d  PNM 1/41 (2%)  RR 0.46 (95% CI 0.031–6.8)  No p value | Placebo PNM 2/39 (5%) | Not PO  PNM: IUFD (no IUFD occurred) and  NNM before discharge | | + | ? | - |
| Awwad, 2015 Libanon PROGESTWIN | Twins, unselected | ART 75%  MC 17% | RandomizedI: 197 C:96  Analyzed I:194 women/388  infants  C: 94 women/188  infants Randomized 2:1 | 17-OHPC 250 mg im/w PNM 17/388 (4.4%)  OR 0.53 (95% CI 0.21-1.33) p=0.18 | Placebo (castor oil) PNM 15/188 (8.0%) | Not PO  PNM: IUFD after 24 w and neonatal deaths <28 d | | + | + | ? |
| Brizot, 2015 Brazil | Twins DA | MC 25% (I) and 19%  (C) Only naturally conceived,  no history of PTB | I: 195/390 C: 195/390 | 200 mg vaginal natural progesterone/d  IUFD 8/378 (2.1%)  OR 1.63 (95% CI 0.42-6.23)  PNM 17/378 (4.5%)  OR 1.33 (95% CI 0.53-3.34)  no p values | Placebo IUFD 5/382 (1.3%)  PNM 13/382 (3.4%) | Not PO  PNM: IUFD and NNM  IUFD not defined NNM death before discharge | | ? | ? | - |
| Combs, 2011 USA | Twins DCDA | 20% fetal reduction ART  I: 66% C:58%  Prior PTB: I: 12%  C: 13% | I: 160/320 C: 80/160  Randomized 2:1 | 17-OHPC 250 mg (in 1 mL castor oil) im/w  IUFD/ miscarriage 0/320 PNM 0/320  p=0.03 | Placebo (1 mL castor oil)  IUFD/miscarriage 0/156 PNM 3/156 (1.9%) | Not PO  PNM: IUFD/miscarriage and NNM  NNM not defined | | + | ? | ? |

* + No or minor problems

? Some problems

- Major problems

Prevention of preterm birth

STable 4.1.10. cont. Intervention progesterone Outcome variable: Perinatal mortality

| **Author, year Country**  **Trial acronym** | **Singletons/ Twins/ Triplets** | **Risk factor** | **Number**  **of randomized patients**  **n=** | **Results** | | | **Comments** | **Directness *** | **Study limitations *** | **Precision *** |
| --- | --- | --- | --- | --- | --- | --- | --- | --- | --- | --- |
|  |  |  |  | **Intervention** | **Control** | |  |  |  |  |
|  |  |  |  |  |  |  | |  |  |  |
| Norman, 2009 UK  (9 hospitals) STOPPIT | Twins | MC twins I: 46/247 C: 45/247  No MA twins | I: 247/494 C: 247/494 | 90 mg vaginal progesterone /day (Crinone)  IUFD 6/494 (1.2%) p=0.52  NNM 8/494 (1.6%) p=0.59  PNM: 14/494 (2.8%)  No statistics | Placebo IUFD 4/494 (0.8%)  NNM 6/494 (1.2%)  PNM: 10/494 (2.0%) | Not PO  PNM (IUFD+ NNM) calculated from 7 Table  IUFD and NNM not defined | | + | + | ? |
| Rehal, 2021  UK (+5 other European countries) | Twins | MC I: 23%  C: 23% ART I: 34%  C: 35% | I: 582/1164 C: 587/1174 | 600 mg vaginal progesterone/d Pregnancy level  PNM 12/582 (2.1%)  OR 1.41 (95% CI 0.58-3.39)  Fetal level PNM 15/1164 (1.3%)  OR 1.57 (95% CI 0.70-3.53) | Placebo Pregnancy level  PNM 9/587 (1.5%)  Fetal level PNM 10/1174 (0.9%) | Not PO  PNM: IUFD and NNM  IUFD and NNM not defined | | + | + | ? |
| Rode, 2011 Denmark and Austria PREDICT | Twins (DA) | MC  I: 43/334 (12.9%)  C: 57/343 (16.6%) | I: 334/668 C: 343/686 | 200 mg vaginal progesterone/d (pessary)  Pregnancy level IUFD 2/334* (0.6%)  OR 1.0 (95% CI 0.1–7.3)  Infant death during delivery One infant  1/334 (0.3%)  OR 1.0 (95% CI 0.1–16.4)  Both infants 0/334 Fetal level (calculated) IUFD: 2/668 (0.3%)  Infant death during delivery: 1/664 (0.2%)  PNM: 10/664 (1.5%)  No statistics | Placebo  Pregnancy level IUFD 2/341* (0.6%)  Infant death during delivery One infant  1/334 (0.3%)  Both infants 1/334 (0.3%) Fetal level (calculated) IUFD: 2/682 (0.3%)  Infant death during delivery: 3/678 (0.4%)  PNM: 7/678 (1.0%) | Not PO IUFD not defined  *only one fetus died PNM: IUFD and death during  delivery and NNM <28 d PNM calculated from Table 3 | | + | + | + |

* + No or minor problems

? Some problems

- Major problems

Prevention of preterm birth

STable 4.1.10. cont. Intervention progesterone Outcome variable: Perinatal mortality

| **Author, year Country**  **Trial acronym** | **Singletons/ Twins/ Triplets** | **Risk factor** | **Number**  **of randomized patients**  **n=** | | **Results** | | **Comments** | **Directness *** | **Study limitations *** | **Precision *** | |
| --- | --- | --- | --- | --- | --- | --- | --- | --- | --- | --- | --- |
|  |  |  |  |  | **Intervention** | **Control** |  |  |  |  |  |
|  |  |  |  |  | |  |  |  |  |  |  |
| Rouse, 2007 USA SSTARS | Twins (DA) | MC  I: 59/327 (18%)  C: 57/334 (17.1%) | I: 327/654 C: 334/668 | 17-OHPC 250 mg im/w Pregnancy level  IUFD 12/325 (3.7)* RR 1.4 (95% CI 0.6-3.2)  PNM 22/325**  RR 1.5 (95% CI 0.8- 2.8)  Fetal level IUFD 18/650 (2.8%)  No statistics PNM 34/650 (5.2%)  RR 1.5 (95% CI 0.8-2.8)  No p value | | Placebo Pregnancy level  IUFD 9/330 (2.7%)* PNM 15/330**  Fetal level IUFD 12/660 (1.8%)  PNM 22/660 (3.3%) | Not PO  *At least one fetus died  **At least one fetus or neonate died  IUFD includes miscarriage, termination of pregnancy, and stillbirth.  PNM: IUFD or NNM  NNM not defined Denominator for fetal level from flowchart and numbers from text | + | ? | + |  |
| Serra, 2013 Spain | Twins (DCDA) | MAR  I1: 92/96 (95.8%)  I2: 94/97 (96.9%)  C: 96/97 (99.0%) | I1: 98/196 I2: 98/196 C: 98/196 | I1: 200 mg vaginal progesterone (pessary) /d  I2: 400 mg vaginal progesterone (pessary)/d  IUFD of co twin I1: 0/97  I2: 3/97 (3.1%)  I1 + I2: 3/194 (1.5%)  Comparison between groups: NS  PNM I1: 0/194  I2: 8/191 (4.2%)  I1+I2: 8/385 (2.1%)  No statistics | | Placebo  IUFD of co twin 2/96 (2.1%)  PNM 5/190 (2.6%) | Not PO  IUFD occurred after 24 w NNM <28 d  PNM calculated | ? | ? | - |  |
| Caritis, 2009 USA | Triplets | 30% DC or unknown chorionicity  ART: 70% | I: 71/213 C: 63/189 | 17-OHPC 250 mg im/w IUFD 1/213 (0.5%)  No statistics PNM 6/213 (2.8%)  No statistics | | Placebo IUFD 6/189 (3.2%)  PNM 8/189 (4.2%) | Not PO  PNM: IUFD and NNM  NNM not defined IUFD included miscarriage,  termination, or IUFD occurring  anytime after randomization PNM calculated | ? | ? | ? |  |

* + No or minor problems

? Some problems

- Major problems

Prevention of preterm birth

STable 4.1.10. cont. Intervention progesterone Outcome variable: Perinatal mortality

| **Author, year Country**  **Trial acronym** | **Singletons/ Twins/ Triplets** | **Risk factor** | **Number**  **of randomized patients**  **n=** | **Results** | | | | **Comments** | **Directness *** | **Study limitations *** | **Precision *** | |
| --- | --- | --- | --- | --- | --- | --- | --- | --- | --- | --- | --- | --- |
|  |  |  |  | **Intervention** | | **Control** | |  |  |  |  |  |
|  |  |  |  | |  |  |  | |  |  |  |  |
| Combs, 2010 | Triplets | Trichorionic triamniotic triplets MAR  I: 90%  C: 84% | I: 56/168 fetuses C: 25 women/75 fetuses | | 17-OHPC 250 mg im/w IUFD 13/168 (8%) p=0.01  PNM 19/168 (11%)  OR 4.7 (95% CI 1.0–22.0) p=0.05 | Placebo IUFD 0/75  PNM 2/75 (3%) | Not PO  PNM: IUFD, miscarriage and NMM  NNM not defined | | + | ? | ? |  |
| Wood, 2012 Canada | Twins and triplets | ART I: 55%  C: 60%  Triplets: I: 2 (5%)  C: 1(2%) | I: 42 women/86 infants  C: 42 women/85 infants | | 90 mg vaginal progesterone gel 8%/d  PNM 2/84* (2%)  RR 1.98 (0.18-21.39) p>0.999  *2 unknown | Placebo PNM 1/85 (1%) | Not PO  PNM: IUFD and NNM  IUFD occurred after 20 w No NNM occurred | | + | ? | + |  |
| Aboulghar 2012  Egypt | Mixed Singletons 215/306 (70.3%)  DC twins 91/306  (29.7%) | ART  pregnancies | Randomized  I:161  C:152  Analyzed I:161  (112 singletons, 49 sets of twins [98 twins])  C:145  (103 singletons, 42 sets of twins [84  twins]) | | 400 mg vaginal natural progesterone/d  Singletons PNM 1/112 (0.9%)  Twins: PNM 4/98 (4.1%)  No statistics | Placebo  Singletons PNM 5/103 (4.9%)  Twins PNM 5/84 (6.0%) | Not PO  PNM: IUFD + NNM  Only one IUFD reported (in singletons, none in twins) NNM < 28 d  PNM calculated from Tables  2 and 3 | | ? | ? | - |  |
| Crowther, 2017 Australia PROGRESS | Mixed Singletons n=775 (98.5%  Twins n=12 (1.5%) | Previous sPTD  <37 w | I: 398 (390  singletons and 8 twin pregnancies) 406 infants  C: 389  (385 singletons and 4 twin pregnancies) 393 infants | | 100 mg vaginal progesterone pessary/d  IUFD 4/406 (1.0%) p=0.749  PNM 5/406 (1.2%)  Unadjusted RR 0.69 (95% CI  0.22–2.16) p=0.526 | Placebo IUFD 5/393 (1.3%)  PNM 7/393 (1.8%) | Not PO PNM: IUFD + NNM  IUFD after trial entry and prior to birth  NNM before discharge | | + | + | ? |  |

* + No or minor problems

? Some problems

- Major problems

Prevention of preterm birth

STable 4.1.10. cont. Intervention progesterone Outcome variable: Perinatal mortality

| **Author, year Country**  **Trial acronym** | **Singletons/ Twins/ Triplets** | **Risk factor** | **Number**  **of randomized patients**  **n=** | **Results** | | | | **Comments** | **Directness *** | **Study limitations *** | **Precision *** | |
| --- | --- | --- | --- | --- | --- | --- | --- | --- | --- | --- | --- | --- |
|  |  |  |  | **Intervention** | | **Control** | |  |  |  |  |  |
|  |  |  |  | |  |  |  | |  |  |  |  |
| Fonseca, 2007 Brazil (multicenter inter-  national, UK [5 centers], Chile, Brazil,  Greece) | Mixed Singletons (226, 90%)  Twins (24,  10%, all DA) | Short TVS CL  ≤15 mm | I: 125 women (114  singletons, 11 twin pregnancies)/136 infants  C: 125 women (112  singletons, 13 twin pregnancies)/138 infants | | 200 mg vaginal progesterone /d (Utrogestan)  IUFD 1/136 (0.7%) p=0.98  PNM 3/136 (2.2%)  No statistics | Placebo IUFD 1/138 (0.7%)  PNM 8/138 (5.8%) | Not PO  PNM: IUFD and NND  PNM calculated from Table 2 IUFD and NND not defined | | ? | ? | + |  |
| Johnson, 1975, single center, USA | Singletons and twins Only one twin pregnancy | Two spontaneous abortions or one preterm birth and one spontaneous abortion immediately before this pregnancy or two preterm birth at any  point. | 50 randomized 43/50 analyzed,  37 included in final analysis  I:18 C:19 | | 17-OHPC 250 mg im/w Fetal level  PNM 0/18 p<0.05 | Placebo Fetal level  PNM 7*/26 (26.9%)  *2 deaths in a twin pregnancy 5 IUFD and 2 NND | Not stated if PO PNM not defined  NB the high PNM rate.  *PNM from Table 1 in article, but number of pregnancies differs from number of pregnancies included in final analysis. | | - | - | - |  |
| **Other interventions in comparison with progesterone** | | | | | | | | | | | |  |
| Cruz-Melguizo, 2018  Spain  (27 centers) | Singletons | Short TVS CL ≤25  mm  (women with cervical  surgery and ≥3  previous PTBs were excluded) | I: 125  C: 118 | | Pessary 6/125 (5%) No RR p=0.35 | 200 mg vaginal progesterone/d 3/118 (3%) | Not PO  PNM: fetal and neonatal death | | + | ? | ? |  |
| Keeler, 2009a USA | Singletons | Short TVS CL ≤25 in women with risk factors for PTB (history of sPTB, second-  trimester pregnancy loss, previous cervical surgery  or uterine anomaly) | I: 42  C: 37 | | Cerclage (McDonald) 5/42 (11.9%)  No statistics | 17-OHPC 250 mg/w  4/37 (10.8%) | Not PO  PNM: any stillbirth or neonatal death during the study period | | + | ? | - |  |

* + No or minor problems

? Some problems

- Major problems

Prevention of preterm birth

STable 4.1.10. cont. Intervention progesterone Outcome variable: Perinatal mortality

| **Author, year Country**  **Trial acronym** | **Singletons/ Twins/ Triplets** | **Risk factor** | **Number**  **of randomized patients**  **n=** | | **Results** | | **Comments** | **Directness *** | **Study limitations *** | **Precision *** | |
| --- | --- | --- | --- | --- | --- | --- | --- | --- | --- | --- | --- |
|  |  |  |  |  | **Intervention** | **Control** |  |  |  |  |  |
|  |  |  |  |  | |  |  |  |  |  |  |
| Dang 2019  Vietnam (single center) | Twins | Short TVS CL  ≤38 mm (women with history of cervical surgery  excluded) | I:150 C: 150 | Arabin pessary Stillbirth 14/296 (5%)  RR 1.08 (95% CI 0.50-2.36) p=0.85  NNM 7/296 (2%)  RR 1.76 (95% CI 0.52-6.15 p=0.38  Subgroup analysis TVS CL ≤28 mm n=94  Stillbirth 4/94 (4%)  RR 0.43 (95% CI 0.11-1.42) p=0.21  NNM 3/94 (3%)  No statistics | | 400 mg vaginal progesterone/d Stillbirth 13/298 (4%)  NNM 4/ 298 (1%)  Subgroup analysis TVS CL ≤28 mm n=70  Stillbirth 7/70 (10%)  NNM 0/94 (0%) | Not PO  PNM: Stillbirths (>28 weeks) and neonatal mortality <28 days | ? | ? | ? |  |

17-OHPC; 17-α-hydroxyprogesterone caproate, ART; assisted reproductive technology, C; control, CL; cervical length, d. days, DCDA; dichorionic diamniotic, DA; diamniotic, DC; dichorionic, GA; gestational age, im; intramuscular, I; intervention, IUFD; intrauterine fetal death, MAR; medically assisted reproduction; MC; monochorionic, MCDA; monochorionic diamniotic, IUFD; intrauterine fetal death, NB nota bene; NNM; neonatal mortality, NR; not reported, OR; odds ratio, PNM; perinatal mortality, PTB; preterm birth, RR; risk ratio, sPTB: spontaneous preterm birth, TVS; transvaginal scan, w; weeks

* + No or minor problems

? Some problems

- Major problems

Prevention of preterm birth

STable 4.1.11. Intervention progesterone

Outcome variable: Neonatal mortality before 28 days

| **Author, year Country**  **Trial acronym** | **Singletons/ Twins/ Triplets** | **Risk factor** | **Number**  **of randomized patients**  **n=** | **Results** | | **Comments** | **Directness *** | **Study limitations *** | **Precision *** |
| --- | --- | --- | --- | --- | --- | --- | --- | --- | --- |
|  |  |  |  | **Intervention** | **Control** |  |  |  |  |
|  |  |  |  |  |  |  |  |  |  |
| Ashoush, 2017 Egypt | Singletons | Previous sPTB (<37 w) | I: 106  C: 106 | 400 mg oral progesterone/d 7/96 (7.3%)  p<0.001 | Placebo 23/91 (25.3%) | Not PO NNM not defined  NB critical comment to article by Katsanevakis, Mol and Thornton concerning recruitment and results  NB High rates of cerclage in both groups | - | ? | - |
| Azargoon, 2016 Iran | Singletons | Previous PTB (<37 w),  uterine malformations | I: 51  C: 52 | 400 mg vaginal progesterone supp/d  2/50 (4%)  RR 4.0 (95% CI 0.89-17.91) p=0.056 | Placebo 21/50 (42 %) | Not PO NNM not defined | - | ? | - |
| Blackwell, 2020 USA PROLONG | Singletons | Previous singleton sPTB | I: 1130  C: 578  Randomized 2:1 | 17-OHPC 250 mg im/w 3/1112 (0.3%)  RR 1.48 (95% CI 0.14-15.24)  No p value | Placebo 1/568 (0.2%) | Not PO NNM <28 d  Patients with missing data assumed not to have the outcome | + | ? | ? |
| Grobman, 2012 USA SCAN | Singletons | Nulliparous with short TVS CL  <30 mm | I: 327  C: 330 | 17-OHPC 250 mg im/w 6/327 (1.8%)  RR 0.76 (95% CI 0.27-2.16)  No p value | Placebo (castor oil) 8/330 (2.4%) | Not PO  NNM not defined | ? | + | + |
| Hassan, 2011 USA + 9 other countries PREGNANT | Singletons | Short TVS CL 10-20 mm (all)  Previous PTB 16% | I: 236  C: 229 | 90 mg vaginal progesterone gel 8%/d  3/235 (1.3%)  RR 0.57 (95% CI 0.14-2.35) p=0.431 | Placebo 5/223 (2.2%) | Not PO  NNM not defined | ? | ? | + |
| Hauth, 1983 USA | Singletons | Women from an active duty  military population | I: 80  C: 88 | 17-OHPC 1000 mg im/w  2/80 (2.5%)  No statistics | Placebo (castor oil) 0/88 | Not PO  NNM not defined | - | - | - |
| Hayashi, 2021 Japan TROPICAL | Singletons | Short TVS CL 25-<30 mm  Previous PTB I: 11.9%  C: 16.7% | I: 59  C: 60 | 200 mg vaginal progesterone/d  0/59 (0%) p=1.0 | Placebo 1/60 (1.7%) | Not PO Infant death | ? | ? | - |
| Ibrahim, 2010 Egypt | Singletons | Previous PTB | I: 25  C: 25 | 17-OHPC 250 mg/w  1 /25 (4%) p<0.05 | Placebo (saline) 4/25 (16%) | Not PO  NNM not defined | - | - | - |

* + No or minor problems

? Some problems

- Major problems

Prevention of preterm birth

STable 4.1.11. cont. Intervention progesterone

Outcome variable: Neonatal mortality before 28 days

| **Author, year Country**  **Trial acronym** | **Singletons/ Twins/ Triplets** | **Risk factor** | **Number**  **of randomized patients**  **n=** | **Results** | | **Comments** | **Directness *** | **Study limitations *** | **Precision *** |
| --- | --- | --- | --- | --- | --- | --- | --- | --- | --- |
|  |  |  |  | **Intervention** | **Control** |  |  |  |  |
|  |  |  |  |  |  |  |  |  |  |
| Meis, 2003 USA | Singletons | Previous sPTB | I:310 C:153  Randomized 2:1 | 17-OHPC 250 mg im/w 8/306 (2.6%)  RR 0.44 (95% CI 0.17–1.13)  no p-value | Placebo (castor oil) 9/153 (5.9%) | Not PO NNM not defined | ? | ? | ? |
| Norman, 2016 UK  (UK 65  hospitals, Sweden 1 hospital) OPPTIMUM | Singletons | FFN pos group: Any of previous PTB, second trimester loss, cervical surgery  FFN neg group: previous sPTB <34 w or  short TVS CL ≤25 mm | I: 618  C: 610 | 200 mg vaginal progesterone/d 1/600 (<1%)  RR 0.17 (95% CI 0.06-0.49) p=0.0009 | Placebo 6/597 (1%) | Not PO NNM not defined | + | + | + |
| O’Brien 2007  USA (+4 other countries) | Singletons | Previous sPTB | I: 332  C: 327 | 90 mg vaginal progesterone gel 8%/d  6/309 (1.9%)  OR 0.87 (95% CI 0.29-2.60)  No p value | Placebo 7/302 (2.3%) | Not PO NNM <28 d | ? | ? | ? |
| Price, 2021 Zambia | Singletons | HIV | I: 399 C:401 | 17-OHPC 250 mg/w  14/388 (4%)  RR 2.0 (95% CI 0.8- 4.9)  No p value | Placebo 7/386 (2%) | Not PO  21 stillborns excluded and 5 infants  with undocumented vital status at 28 days | ? | + | ? |
| Rai, 2009  India | Singletons | Previous sPTB | I: 75 C:75 | 200 micronized oral progesterone/d  3/74 (4.1%)  No RR or OR p=0.190 | Placebo 7/74 (9.5%) | Not PO NNM not defined | - | ? | ? |
| Van Os, 2015 The Netherlands TRIPLE P | Singleton | Short TVS CL  ≤30 mm,  no previous PTB | I: 41  C: 39 | 200 mg micronized progesterone vaginal/d  1/41 (2%)  RR 0.46 (95% CI 0.031–6.8)  No p value | Placebo 2/39 (5%) | Not PO  NNM before discharge | + | ? | - |

Prevention of preterm birth

STable 4.1.11. cont. Intervention progesterone

Outcome variable: Neonatal mortality before 28 days

* + No or minor problems

? Some problems

- Major problems

| **Author, year Country**  **Trial acronym** | **Singletons/ Twins/ Triplets** | **Risk factor** | **Number**  **of randomized patients**  **n=** | **Results** | | **Comments** | **Directness *** | **Study limitations *** | **Precision *** |
| --- | --- | --- | --- | --- | --- | --- | --- | --- | --- |
|  |  |  |  | **Intervention** | **Control** |  |  |  |  |
|  |  |  |  |  |  |  |  |  |  |
| Awwad, 2015 Libanon PROGESTWIN | Twins, unselected | ART 75%  MC 17% | RandomizedI: 197 C:96  Analyzed I:194 women/388  infants C: 94  women/188 infants Randomized 2:1 | 17-OHPC 250 mg im/w 5/388 (1.3%)  RR 0.48 (95% CI 0.10–2.32) p=0.36 | Placebo (castor oil) 5/188 (2.7%) | Not PO NNM <28 d | + | + | ? |
| Briery, 2009 USA | Twins, unselected | 1/3 previous PTB | I: 16/32 C: 14/28 | 17-OHPC 250 mg im/w 2/32 (6%) p=0.359 | Placebo 0/28 | Not PO NNM not defined | ? | ? | - |
| Brizot, 2015 Brazil | Twins DA | MC 25% (I) and 19%  (C) Only naturally conceived,  no history of PTB | I: 195/378 C: 195/382 | 200 mg vaginal natural progesterone/d 9/378 (2.4%)  OR 1.14 (0.34-3.81)  No p value | Placebo 8/382 (2.1%) | Not PO  NNM before discharge | ? | ? | - |
| Combs, 2011 USA | Twins DCDA | 20% fetal reduction ART  I: 66% C:58%  Prior PTB: I: 12%  C: 13% | I: 160/320 C: 80/160  Randomized 2:1 | 17-OHPC 250 mg (in 1 mL castor oil) im/w  0/320 p=0.03 | Placebo (1 mL castor oil) 3/156 (2%) | Not PO NNM not defined | + | ? | ? |
| Norman, 2009 UK  (9 hospitals) STOPPIT | Twins | MC twins I: 46/247 C: 45/247  No MA twins | I: 247/494 C: 247494 | 90 mg vaginal progesterone /day (Crinone)  8/494 (1.6%) p=0.59 | Placebo 6/494 (1.2%) | Not PO NNM not defined | + | + | ? |
| Rode, 2011 Denmark and Austria PREDICT | Twins (DA) | MC  I: 43/334 (12.9%)  C: 57/343 (16.6%) | I: 334/668 C: 343/686 | 200 mg vaginal progesterone/d (pessary)  7/664 (1.0%)  No statistics | Placebo 2/678 (0.3%) | Not PO NNM <28 d | + | + | + |

* + No or minor problems

? Some problems

- Major problems

Prevention of preterm birth

STable 4.1.11. cont. Intervention progesterone

Outcome variable: Neonatal mortality before 28 days

| **Author, year Country**  **Trial acronym** | **Singletons/ Twins/ Triplets** | **Risk factor** | **Number**  **of randomized patients**  **n=** | **Results** | | **Comments** | **Directness *** | **Study limitations *** | **Precision *** |
| --- | --- | --- | --- | --- | --- | --- | --- | --- | --- |
|  |  |  |  | **Intervention** | **Control** |  |  |  |  |
|  |  |  |  |  |  |  |  |  |  |
| Serra, 2013 Spain | Twins (DCDA) | MAR  I1: 92/96 (95.8%)  I2: 94/97 (96.9%)  C: 96/97 (99.0%) | I1: 98/196 I2: 98/196 C: 98/196 | I1: 200 mg vaginal progesterone (pessary) /d  I2: 400 mg vaginal progesterone (pessary)/d  I1: 0/194  I2: 5/191 (2.6%)  I1+I2: 5/385 (1.3%)  Comparison between groups: NS | Placebo  3/190 (1.6%) | Not PO NNM <28 d | ? | ? | - |
| Caritis, 2009 USA | Triplets | 30% DC or unknown chorionicity  ART: 70% | I: 71/213 C: 63/183 | 17-OHPC 250 mg im/w 5/213 (2.3%)  RR 2.2 (95% CI 0.4–12.4) | Placebo 2/183 (1.1%) | Not PO NNM not defined | ? | ? | ? |
| Combs, 2010 | Triplets | Trichorionic triamniotic triplets MAR  I: 90%  C: 84% | I: 56 women/155 liveborn neonates C: 25 women/75 liveborn neonates | 17-OHPC 250 mg im/w 6/155 (4%)  OR 1.5 (95% CI 0.3-8.1) p=0.66 | Placebo 2/75 (3%) | Not PO NNM not defined | + | ? | ? |
| Wood, 2012 Canada | Twins and triplets | ART I: 55%  C: 60%  Triplets: I: 2 (5%)  C: 1(2%) | I: 42 women/86 infants  C: 42 women/85 infants | 90 mg vaginal progesterone gel 8%/d  0/86 | Placebo 0/85 | Not PO NNM not defined | + | ? | + |
| Lim, 2011  The Netherlands AMPHIA | Multifetal pregnancies | Triplets/+ I: 9 (3%)  C: 9 (3%)  (incl.one quadruplet) MC  I: 57 (17%)  C: 57 (17%)  Fertility treatment I: 140 (42%)  C: 120 (36%) | I: 336 women  /681 infants (654  twins, 27 triplets)  C: 335 women/  680 infants (652 twins,  24 triplets, 4 quads) | 17-OHPC 250 mg im/w 13/681 (2%)  RR 0.60 (95% CI 0.25–1.43)  No p value | Placebo 21/674 (3%) | Not PO  NNM before discharge | + | + | ? |

* + No or minor problems

? Some problems

- Major problems

Prevention of preterm birth

STable 4.1.11. cont. Intervention progesterone

Outcome variable: Neonatal mortality before 28 days

| **Author, year Country**  **Trial acronym** | **Singletons/ Twins/ Triplets** | **Risk factor** | **Number**  **of randomized patients**  **n=** | **Results** | | **Comments** | **Directness *** | **Study limitations *** | **Precision *** |
| --- | --- | --- | --- | --- | --- | --- | --- | --- | --- |
|  |  |  |  | **Intervention** | **Control** |  |  |  |  |
| Aboulghar 2012  Egypt | Mixed Singletons 215/306 (70.3%)  DC twins 91/306 (29.7%) | ART  pregnancies | RandomizedI:161 C:152  Analyzed I:161  (112 singletons,  49  sets of twins [98 twins])  C:145  (103 singletons,  42 sets of twins [84 twins]) | 400 mg vaginal natural progesterone/d  Singletons 1/112 (0.9%)  No statistics Twins Pregnancy level  2/49 (4.1%)  OR 0.31 (95% CI 0.05-1.71  Fetal level 4/98 (4.1%) | Placebo  Singletons 4/103 (3.9%)  Twins Pregnancy level 5/42 (11.9%)  Fetal level 5/84 (6.0%) | Not PO NNM <28 d  Fetal level calculated | ? | ? | - |
| Cetingoz, 2011 Turkey | Mixed Singletons I: 41/80 (51.3%) C: 42/70 (60%)  Twins I: 39/80 (48.7%) C: 28/70  (40.0%) | Twin pregnancy, previous sPTD, uterine malformation | I: 84 women  C: 76 women | 100 mg vaginal progesterone/d Singletons and twins  3/80* (3.8%)  OR 1.15 (95% CI 0.2-5.9)  (C vs I) p=0.867 | Placebo Singletons and twins  3/70* (4.3%) | Not PO  *Number of births with at least one NNM  NNM not defined | ? | ? | - |
| Crowther, 2017 Australia PROGRESS | Mixed Singletons n=775 (98.5%  Twins n=12 (1.5%) | Previous sPTD  <37 w | I: 398 women  (390 singletons,  8 twin pregnancies)/ 406 infants  C: 389 women  (385 singletons,  4 twin pregnancies)/ 393 infants | 100 mg vaginal progesterone pessary/d  1/406 (0.3%) p=0.619 | Placebo 2/393 (0.5%) | Not PO  NNM before discharge | + | + | ? |

* + No or minor problems

? Some problems

- Major problems

Prevention of preterm birth

STable 4.1.11. cont. Intervention progesterone

Outcome variable: Neonatal mortality before 28 days

| **Author, year Country**  **Trial acronym** | **Singletons/ Twins/ Triplets** | **Risk factor** | **Number**  **of randomized patients**  **n=** | **Results** | | **Comments** | **Directness *** | **Study limitations *** | **Precision *** |
| --- | --- | --- | --- | --- | --- | --- | --- | --- | --- |
|  |  |  |  | **Intervention** | **Control** |  |  |  |  |
|  |  |  |  |  |  |  |  |  |  |
| Fonseca, 2007 Brazil (multicenter inter-  national, UK [5 centers], Chile, Brazil,  Greece) | Mixed Singletons (226, 90%)  Twins (24,  10%, all DA) | Short TVS CL  ≤15 mm | I: 125 women  (114 singletons,  11 twin pregnancies)/136 infants  C: 125 women  (112 singletons,  13 twin pregnancies)/138 infants | 200 mg vaginal progesterone /day (Utrogestan)  2/136 (1.5%)  RR 0.29 (95% CI 0.06-1.42) p=0.13  Adjusted RR 0.34 (95% CI 0.06-  1.81) p=0.22  RR adjusted for maternal age, BMI, smoking status, race, history of preterm birth, and cervical  length at the time of randomization. | Placebo 7/138 (5.1%) | Not PO NNM not defined | ? | ? | + |
| **Other interventions in comparison with progesterone** | | | | | | | | | |
| Dang 2019  Vietnam (single center) | Twins | Short TVS CL  ≤38 mm (women with history of  cervical surgery excluded) | I:150 C: 150 | Arabin pessary  7/296 (2%)  RR 1.76 (95% CI 0.52-6.15 p=0.38  Subgroup analysis TVS CL ≤28 mm n=94  3/94 (3%)  No statistics | Vaginal progesterone 400 mg/d  4/149 (1%)  Subgroup analysis TVS CL ≤28 mm n=70  0/70 (0%) | Not PO | ? | ? | ? |

17-OHPC;17-α-hydroxyprogesterone caproate, ART; assisted reproductive technology, BMI; body mass index, C; control, CL; cervical length, DC; dichorionic, DCDA; dichorionic diamniotic, GA; gestational age, im; intramuscular, I; intervention, IUFD; intrauterine fetal death, MAR; medically assisted reproduction; MC; monochorionic, MCDA; monochorionic diamniotic, NNM; neonatal mortality, OR; odds ratio, PTB; preterm birth, RR; risk ratio, sPTB: spontaneous preterm birth, TVS; transvaginal scan, w; weeks

|  |  |  |  |  |  |  |  |  |  |
| --- | --- | --- | --- | --- | --- | --- | --- | --- | --- |
| Blackwell, 2020 USA PROLONG | Singletons | Previous singleton sPTB | I: 1130  C: 578  Randomized 2:1 | 17-OHPC 250 mg im/w 61/1093 (5.6%)  RR 1.12 (95% CI 0.72-1.72) p=0.62 | Placebo 28/559 (5.0%) | Not PO  Patients with missing data assumed not to have the outcome  Composite neonatal outcome: any of NNM, IVH 3-4, RDS, BPD, NEC, or  proven sepsis | + | ? | ? |
| Grobman, 2012 USA  SCAN | Singletons | Nulliparous with short TVS CL  <30 mm | I: 327  C: 330 | 17-OHPC 250 mg im/w 23/327 (7.0%)  RR 0.77 (95% CI 0.46-1.30)  No p value | Placebo (castor oil) 30/330 (9.1%) | Not PO  Composite neonatal outcome: RDS, BPD, sepsis, NEC, IVH 3-4, PVL,  ROP, IUFD or NNM | ? | + | + |
| Hassan, 2011 USA + 9 other countries PREGNANT | Singletons | Short TVS CL 10-20 mm (all)  Previous PTB 16% | I: 236  C: 229 | 90 mg vaginal progesterone gel 8%/d  18/235 (7.7%)  RR 0.57 (95% CI 0.33-0.99) p=0.043 | Placebo 30/223 (13.5%) | Not PO  Composite neonatal outcome: RDS, BPD, IVH 3-4, PVL, proven sepsis, NEC and PNM (IUFD or NNM) | ? | ? | + |
| Norman, 2016 UK  (UK 65  hospitals, Sweden 1 hospital) OPPTIMUM | Singletons | FFN pos group: Any of previous PTB, second trimester loss, cervical surgery  FFN neg group: previous sPTB <34 w or  short TVS CL ≤25 mm | I: 618  C: 610 | 200 mg vaginal progesterone/d 39/589 (7%)  OR 0.62 (95% CI 0.41-1.07)* p=0.02?  aOR 0.62 (95% CI (0.38-1.03) p=0.072  aOR adjusted for multiple primary outcomes using Bonferroni-Holm | Placebo 60/587 (10%) | PO (one of three) Composite neonatal outcome: Neonatal morbidity (BPD and/or brain injury on  ultrasound scan) or NNM  *corrected (in article 95% CI 0.41- 0.94), no errata of p value  Subgroup analyses for TVS CL and history of PTB, but without events | + | + | + |
| Awwad, 2015 Libanon PROGESTWIN | Twins, unselected | ART 75%  MC 17% | Randomized 2:1  I: 197 C:96  Analyzed I:194 women/388  fetuses C: 94  women/188  fetuses | 17-OHPC 250 mg im/w 74/388 (19.1%)  OR 0.53 (95% CI 0.31-0.90) p=0.02 | Placebo (castor oil) 58/188 (30.9%) | Not PO  Composite neonatal outcome: RDS, pneumonia, proven sepsis, IVH (grade III-IV), NEC, PVL, ROP, PDA,  seizures, and/or BPD | + | + | ? |

* + No or minor problems

? Some problems

- Major problems

Prevention of preterm birth

STable 4.1.12. Intervention progesterone

Outcome variable: Composite adverse neonatal outcome

| **Author, year Country**  **Trial acronym** | **Singletons/ Twins/ Triplets** | **Risk factor** | **Number**  **of randomized patients**  **n=** | **Results** | | **Comments/definitions** | **Directness *** | **Study limitations *** | **Precision *** |
| --- | --- | --- | --- | --- | --- | --- | --- | --- | --- |
|  |  |  |  | **Intervention** | **Control** |  |  |  |  |

|  |  |  |  |  |  |  |  |  |  |
| --- | --- | --- | --- | --- | --- | --- | --- | --- | --- |
| Brizot, 2015 Brazil | Twins DA | MC 25% (I) and 19%  (C) Only naturally conceived,  no history of PTB |  | 200 mg vaginal natural progesterone/d 45/291 (15.5%)  OR 1.01 (95% CI 0.58-1.75)  No p value  * + No or minor problems  ? Some problems  - Major problems | Placebo 51/320 (15.9%) | * + No or minor problems  ? Some problems  - Major problems | ? | ? | - |
| Combs, 2011 USA | Twins DCDA | 20% fetal reduction ART  I: 66% C:58%  Prior PTB: I: 12%  C: 13% |  | 17-OHPC 250 mg (in 1 mL castor oil) im/w  46/320 (14%)  OR 1.2 (95% CI 0.6-2.5) p=0.62 | Placebo (1 mL castor oil)  19/155 (12%) | * + No or minor problems  ? Some problems  - Major problems | + | ? | ? |
| Serra, 2013 Spain | Twins (DCDA) | MAR  I1: 92/96 (95.8%)  I2: 94/97 (96.9%)  C: 96/97 (99.0%) |  | I1: 200 mg vaginal progesterone (pessary) /d  I2: 400 mg vaginal progesterone (pessary)/d  Short-term morbidity I1: 24/194 (12.4%)  I2: 31/191 (16.2%)  I1+I2: 55/385 (14.3%)  Long-term morbidity I1: 2/194 (1.0%)  I2: 0 /191  I1+I2: 2/385 (0.5%)  Comparison between groups: NS | Placebo  Short-term morbidity 27/190 (14.2%)  Long-term morbidity 2/190 (1.1%) | * + No or minor problems  ? Some problems  - Major problems | ? | ? | - |
| Caritis, 2009 USA | Triplets | 30% DC or unknown chorionicity  ART: 70% |  | 17-OHPC 250 mg im/w 78/212 (37%)  RR 1.1 (95% CI 0.7-1.7)  No p value | Placebo 65/183 (34%) | * + No or minor problems  ? Some problems  - Major problems | ? | ? | ? |

* + No or minor problems

? Some problems

- Major problems

Prevention of preterm birth

STable 4.1.12. cont. Intervention progesterone

Outcome variable: Composite adverse neonatal outcome

| **Author, year Country**  **Trial acronym** | **Singletons/ Twins/ Triplets** | **Risk factor** | **Number**  **of randomized patients**  **n=** | **Results** | | **Comments/definitions** | **Directness *** | **Study limitations *** | **Precision *** |
| --- | --- | --- | --- | --- | --- | --- | --- | --- | --- |
|  |  |  |  | **Intervention** | **Control** |  |  |  |  |

| Combs, 2010 USA | Triplets | Trichorionic triamniotic triplets MAR  I: 90%  C: 84% | I: 56 women/168  (155 liveborn neonates)  C: 25 women/75  (75 liveborn neonates) |  | Placebo 31/75 (41%) | PO  Composite neonatal morbidity: PNM (IUFD, NNM, miscarriage), RDS, BPD (use of oxygen therapy at 28 days of life), proven sepsis, pneumonia, IVH 3-  4, PVL, NEC, ROP, or asphyxia | + | ? | ? |
| --- | --- | --- | --- | --- | --- | --- | --- | --- | --- |
| Lim, 2011  The Netherlands AMPHIA | Multifetal pregnancies | Triplets/+ I: 9 (3%)  C: 9 (3%)  (incl.one quadruplet) MC  I: 57 (17%)  C: 57 (17%)  Fertility treatment I: 140 (42%)  C: 120 (36%) | I: 336 women  /681 infants (327 sets of twins, 9 sets of triplets) C: 335 women/  680 infants (326 sets of twins, 8 sets of triplets, one quadruplet) |  | Placebo 80/674 (12%) | PO  Composite  severe RDS, BPD, IVH grade II B or worse, NEC, proven sepsis, and NNM before discharge from the hospital | + | + | ? |
| Crowther, 2017 Australia PROGRESS | Mixed Singletons n=775 (98.5%  Twins n=12 (1.5%) | Previous sPTD  <37 w | I: 398/406  infants (390 singletons  and 8 twin pregnancies) C: 389/393  infants (385 singletons  and 4 twin  pregnancies) |  | Placebo  152/393 (38.7%) | Not PO  Composite neonatal outcome: PTB <37w, PNM, RDS, BPD, Apgar score <4, SGA (< 3^rd^ centile), IVH, PVL, PDA, NEC, proven sepsis, and/or ROP | + | + | ? |
| Fonseca, 2007 Brazil (multicenter inter-  national, UK [5 centers], Chile, Brazil,  Greece) | Mixed Singletons (226, 90%)  Twins (24  sets, 10%, all DA) | Short TVS CL  ≤15 mm | I: 125  women/136 infants (114  singletons, 22 twins)  C: 125  women/138 infants (112  singletons, 26 twins) |  | Placebo 19/138 (13.8%) | Not PO Composite neonatal outcome: RDS, IVH, NEC, and/or ROP | ? | ? | + |

* + No or minor problems

? Some problems

- Major problems

Prevention of preterm birth

STable 4.1.12. cont. Intervention progesterone

Outcome variable: Composite adverse neonatal outcome

| **Author, year Country**  **Trial acronym** | **Singletons/ Twins/ Triplets** | **Risk factor** | **Number**  **of randomized patients**  **n=** | **Results** | | **Comments/definitions** | **Directness *** | **Study limitations *** | **Precision *** |
| --- | --- | --- | --- | --- | --- | --- | --- | --- | --- |
|  |  |  |  | **Intervention** | **Control** |  |  |  |  |

|  | | | | | | | | | |
| --- | --- | --- | --- | --- | --- | --- | --- | --- | --- |
| **Other interventions in comparison with progesterone** | | | | | | | | | |
| Cruz-Melguizo, 2018  Spain  (27 centers) | Singletons | Short TVS CL ≤25 mm  (women with cervical surgery and ≥3 previous PTBs were  excluded) | I: 125  C: 118 | Pessary 24/125 (19%) No RR p=0.65 | 200 mg vaginal progesterone/d 20/118 (17%) | Not PO  Composite adverse outcome included mechanical ventilation, IVH, RDS, ROP, NEC, NICU admissions | + | ? | ? |
| Keeler, 2009a USA | Singletons | Short TVS  CL ≤25 in women with risk factors for PTB (History of sPTB, second-  trimester pregnancy loss, previous cervical surgery  or uterine anomaly) | I: 42  C: 37 | Cerclage (McDonald) Mild 1/42 (2.3%)  Severe 9/42 (21.4%) p=0.34 for any morbidity | 17-OHPC 250 mg  weekly  Mild 5/37 (13.5%)  Severe 7/37 (18.9%) | Not PO  Mild morbidity defined as NICU admission without severe morbidity. Severe morbidity defined as life threatening morbidity including RDS requiring mechanic ventilation >24 h, IVH, sepsis, or NEC | + | ? | - |
| Dang 2019  Vietnam (single center) | Twins | Short TVS  CL ≤38  mm  (women with history of cervical surgery excluded) | I:150 C: 150 | Arabin pessary  55/296 (19%)  RR 0.70 (95% CI 0.43-0.93) p=0.02  Subgroup analysis TVS CL ≤28 mm n=94  18/94 (19%)  RR 0.38 (95% CI 0.12-0.47) p<0.001 | 400 mg vaginal progesterone /d 79/298 (27%)  Subgroup analysis TVS CL ≤28 mm n=70  35/70 (50%) | Not PO Post hoc analysis  Not defined, but PNM, RDS, BPD, IVH, NEC, sepsis, Apgar <7 at 5 min, NICU admissions were reported | ? | ? | ? |

17-OHPC;17-α-hydroxyprogesterone caproate, aRR; adjusted risk ratio, aOR; adjusted odds ratio, ART; assisted reproductive technology, BMI; body mass index, BPD; bronchopulmonary dysplasia, C; control, CL; cervical length, DCDA; dichorionic diamniotic, DA; diamniotic, DC; dichorionic, GA; gestational age, im; intramuscular, h; hours, I; intervention, IUFD; intrauterine fetal death, IVH; intraventricular hemorrhage, MAR; medically assisted reproduction; MC; monochorionic, MCDA; monochorionic diamniotic, NEC; necrotizing enterocolitis, NNM; neonatal mortality, OR; odds ratio, PDA; patent ductus arteriosus, PTB; preterm birth, PVL; periventricular leucomalaci, RDS; respiratory distress syndrome, RR; risk ratio, ROP; retinopathy of prematurity, sPTB: spontaneous preterm birth, TVS; transvaginal scan, w; weeks

| **Author, year Country**  **Trial acronym** | **Singletons/ Twins/ Triplets** | **Risk factor** | **Number**  **of randomized patients**  **n=** | **Results** | | **Comments/definitions** | **Directness *** | **Study limitations *** | **Precision *** |
| --- | --- | --- | --- | --- | --- | --- | --- | --- | --- |
|  |  |  |  | **Intervention** | **Control** |  |  |  |  |

* + No or minor problems

? Some problems

- Major problems

Prevention of preterm birth

STable 4.1.12. cont. Intervention progesterone

Outcome variable: Composite adverse neonatal outcome

| Ashoush, 2017 Egypt | Singletons |  | I: 106  C: 106 | 400 mg oral progesterone/d 21/96 (21.8%)  p=0.004 No RR | Placebo 39/91 (42.8%) | Not PO  NB critical comment to article by Katsanevakis, Mol and Thornton concerning recruitment and results  NB High rates of cerclage in both groups | - | ? | - |
| --- | --- | --- | --- | --- | --- | --- | --- | --- | --- |
| Azargoon, 2016 Iran | Singletons | Previous PTB (<37 w),  uterine malformations | I: 51  C: 52 | 400 mg vaginal progesterone supp  /d 10/50 (20%)  RR 2.1 (95% CI 1.10-3.99) p=0.017  No RR | Placebo21/50 (42%) | Not PO | - | ? | - |
| Blackwell, 2020 USA PROLONG | Singletons | Previous singleton sPTB | Randomized2:1  I: 1130  C: 578 | 17-OHPC 250 mg im/w 54/1093 (4.9%)  RR 1.06 (95% CI 0.67-1.68)  No p value | Placebo 26/559 (4.7%) | Not PO  Patients with missing data assumed not to have the outcome | + | ? | ? |
| Grobman, 2012 USA  SCAN | Singletons | Nulliparous with short TVS CL  <30 mm | I: 327  C: 330 | 17-OHPC 250 mg im/w 13/320 (4.1%)  RR 0.82 (95% CI 0.40-1.68)  No p value | Placebo (castor oil) 16/323 (5.0%) | Not PO | ? | + | + |
| Hassan, 2011 USA + 9 other countries PREGNANT | Singletons | Short TVS CL 10-20 mm (all)  Previous PTB 16% | I: 236  C: 229 | 90 mg vaginal progesterone gel 8%/d  7/235 (3.0%)  RR 0.39 (95% CI 0.17-0.92) p=0.026 | Placebo 17/223 (7.6%) | Not PO ITT analysis set | ? | ? | + |
| Meis, 2003 USA | Singletons | Previous sPTB | Randomized 2:1  I:310 C:153 | 17-OHPC 250 mg im/w 29/305 (9.5%)  RR 0.63 (95% CI 0.38-1.05)  No p value | Placebo (castor oil) 23/152 (15.1%) | Not PO | ? | ? | ? |
| O’Brien,  2007  USA (+4 other countries) | Singletons | Previous sPTB | I: 332  C: 327 | 90 mg vaginal progesterone gel 8%/d  34/309 (11.0%)  OR 0.91 (95% CI 0.56-1.50)  No p value | Placebo 36/302 (11.9%) | Not PO | ? | ? | ? |
| Van Os, 2015 The Netherlands TRIPLE P | Singletons | Short TVS CL  ≤30 mm,  no previous PTB | I: 41  C: 39 | 200 mg micronized progesterone vaginal/d  2/41 (5%)  RR 0.92 (95% CI 0.14-6.21)  No p value | Placebo 2/39 (6%) | Not PO | + | ? | - |

| **Author, year Country**  **Trial acronym** | **Singletons/ Twins/ Triplets** | **Risk factor** | **Number**  **of randomized patients**  **n=** | **Results** | | **Comments** | **Directness *** | **Study limitations *** | **Precision *** |
| --- | --- | --- | --- | --- | --- | --- | --- | --- | --- |
|  |  |  |  | **Intervention** | **Control** |  |  |  |  |

| Author, year Country  Trial acronym | Singletons/ Twins/ Triplets | Risk factor | Number  of randomized patients  n= | Results | | Comments/definitions | Directness * | Study limitations * | Precision * |
| --- | --- | --- | --- | --- | --- | --- | --- | --- | --- |
|  |  |  |  | Intervention | Control |  |  |  |  |

* + No or minor problems

? Some problems

- Major problems

Prevention of preterm birth

STable 4.1.13. Intervention progesterone

Outcome variable: Respiratory distress syndrome (RDS)

| Yemini, 1985 Israel | Singletons | Previous ≥2 PTB or  ≥2 spontaneous  miscarriages | I: 39  C: 40 | 17-OHPC 250 mg im/w 1/5 (20%)  No statistics | Placebo 4/14 (28.6%) | Not PO  All had cerclage Denominator = infants born < 36 w | - | | - | - |
| --- | --- | --- | --- | --- | --- | --- | --- | --- | --- | --- |
| Awwad, 2015 Libanon PROGESTWIN | Twins, unselected | ART 75%  MC 17% | Randomized 2:1  I: 197 C:96  Analyzed I:194 women/388  fetuses C: 94  women/188 fetuses | 17-OHPC 250 mg im/w 55/381 (14.4%)  OR 0.55 (95% CI 0.31-0.98) p=0.04 | Placebo (castor oil) 44/188 (23.4%) | Not PO | + | | + | ? |
| Briery, 2009 USA | Twins, unselected | 1/3 previous PTB | I: 16/32 C: 14/28 | 17-OHPC 250 mg im/w 10/32 (31%)  p=0.838 No RR | Placebo 9/28 (32%) | Not PO | | ? | ? | - |
| Brizot, 2015 Brazil | Twins DA | MC 25% (I) and 19% (C) Only naturally conceived, no history of PTB | I: 195/378 C: 195/382 | 200 mg vaginal natural progesterone/d 39/296 (13.2%)  OR 0.95 (95% CI 0.54-1.69)  No p value | Placebo 44/321 (13.7%) | Not PO | | ? | ? | - |
| Rode, 2011 Denmark and Austria PREDICT | Twins DA | MC  I: 43/334 (12.9%)  C: 57/343 (16.6%) | I: 334/668 C: 343/686 | 200 mg vaginal progesterone/d (pessary)  73/659 (11.1%)  OR 1.1 (95% CI 0.7-1.7)  No p values | Placebo 69/674 (10.2%) | Not PO | | + | + | + |
| Combs, 2011 USA | Twins DCDA | 20% fetal reduction ART  I: 66% C:58%  Prior PTB: I: 12%  C: 13% | Randomized 2:1 I: 160/320  C: 80/160 | 17-OHPC 250 mg (in 1 mL castor oil) im/w  44/319 (14%)  OR 1.2 (95% CI 0.6-2.6) p=0.64 | Placebo (1 mL castor oil) 18/153 (12%) | Not PO | | + | ? | ? |
| Caritis, 2009 USA | Triplets | 30% DC or  unknown chorionicity ART:  70% | I: 71 women/212 infants  C: 63  women/183 infants | 17-OHPC 250 mg im/w 65/212 (31%)  RR 1.1 (95% CI 0.7-1.8) | Placebo 50/183 (27%) | Not PO | | ? | ? | ? |

Prevention of preterm birth

STable 4.1.13. cont. Intervention progesterone

Outcome variable: Respiratory distress syndrome (RDS)

* + No or minor problems

? Some problems

- Major problems

| **Author, year Country**  **Trial acronym** | **Singletons/ Twins/ Triplets** | **Risk factor** | **Number**  **of randomized patients**  **n=** | **Results** | | **Comments** | **Directness *** | **Study limitations *** | **Precision *** |
| --- | --- | --- | --- | --- | --- | --- | --- | --- | --- |
|  |  |  |  | **Intervention** | **Control** |  |  |  |  |

|  |  |  |  |  |  |  |  |  |  |
| --- | --- | --- | --- | --- | --- | --- | --- | --- | --- |
| Combs, 2010 USA | Triplets | Trichorionic triamniotic triplets MAR  I: 90%  C: 84% | I: 56/155 | 17-OHPC 250 mg im/w 44/155 (28%)  OR 0.68 (95% CI 0.3-1.6) p=0.38 | Placebo 28/75 (37%) | Not PO | + | ? | ? |
| Lim, 2011  The Netherlands AMPHIA | Multifetal pregnancies | Triplets/+ I: 9 (3%)  C: 9 (3%)  (incl.one quadruplet) MC  I: 57 (17%)  C: 57 (17%)  Fertility treatment I: 140 (42%)  C: 120 (36%) | I: 336 women  /681 infants (327 sets of twins, 9 sets of triplets) C: 335 women/  680 infants (326 sets of twins, 8 sets of triplets, one quadruplet) | 17-OHPC 250 mg im/w 82/681 (12%)  RR 1.55 (95% CI 1.01-2.37)  No p value | Placebo 51/674 (8%) | Not PO | + | + | ? |
| Wood, 2012 Canada | Twins and triplets | ART I: 55%  C: 60%  Triplets: I: 2 (5%)  C: 1(2%) | I: 42/86 (40 sets of twins, 2 sets of triplets)  C: 42/85 (40 sets of twins, one set  of triplets) | 90 mg progesterone vaginal gel 8%/d  15/86 (17%)  Difference 0.68 (95% CI 0.38-  1.22) p=0.264 | Placebo 22/85 (26%) | Not PO | + | ? | + |
| Crowther, 2017 Australia PROGRESS | Mixed Singletons n=775 (98.5%  Twins n=12 (1.5%) | Previous sPTD  <37 w | I: 398 (390  singletons and 8 twin pregnancies) 406 infants  C: 389  (385 singletons  and 4 twin pregnancies) 393 infants | 100 mg vaginal progesterone pessary/d  42/402 (10.5%)  RR 0.99 (95% CI 0.65-1.51) p=0.958  aRR 0.98 (95% CI 0.64-1.49) p=0.912 | Placebo 41/388 (10.6%) | PO | + | + | ? |


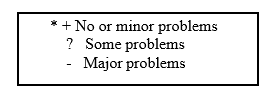


| **Author, year Country**  **Trial acronym** | **Singletons/ Twins/ Triplets** | **Risk factor** | **Number**  **of randomized patients**  **n=** | **Results** | | **Comments** | **Directness *** | **Study limitations** | **Precision *** |
| --- | --- | --- | --- | --- | --- | --- | --- | --- | --- |
|  |  |  |  | **Intervention** | **Control** |  |  |  |  |

Prevention of preterm birth

STable 4.1.13. cont. Intervention progesterone

Outcome variable: Respiratory distress syndrome (RDS)

| Fonseca, 2007 Brazil (multicenter inter- national, UK [5 centers], Chile, Brazil,  Greece) | Mixed Singletons (226,  90%)  Twins (24, 10%, all DA) | Short TVS CL ≤15 mm | I: 125  women/136 infants (114  singletons, 22 twins)  C: 125  women/138 infants (112  singletons, 26 twins) | 200 mg vaginal progesterone /day (Utrogestan)  11/136 (8.1%)  RR 0.59 (95% CI 0.26-1.25) p=0.17  aRR 0.57 (95% CI 0.23-1.31) p=0.19  aRR adjusted for maternal age, BMI, smoking status, race, history of PTB, and cervical length at the  time of randomization. | Placebo 19/138 (13.8%) | Not PO | ? | ? | + |
| --- | --- | --- | --- | --- | --- | --- | --- | --- | --- |
| **Other interventions in comparison with progesterone** | | | | | | | | | |
| Cruz-Melguizo, 2018  Spain  (27 centers) | Singletons | Short TVS CL ≤25 mm (women with cervical surgery and ≥3 previous  PTBs were excluded) | I: 125  C: 118 | Pessary 7/125 (6%) No RR p=0.81 | 200 mg vaginal progesterone/d 6/118 (5%) | Not PO | + | ? | ? |
| Dang 2019 | Twins | Short TVS  CL ≤38 mm | I:150 C: 150 | Arabin pessary | 400 mg vaginal progesterone /d | Not PO | ? | ? | ? |
| Vietnam |  | (women with |  | 32/296 (11%) | 51/298 (17%) |  |  |  |  |
| (single center) |  | history of cervical |  | RR 0.63 (95 % 0.37-0.94) |  |  |  |  |  |
|  |  | surgery excluded) |  | p=0.03 |  |  |  |  |  |
|  |  |  |  | Subgroup analysis | Subgroup analysis |  |  |  |  |
|  |  |  |  | TVS CL ≤28 mm | TVS CL ≤28 mm |  |  |  |  |
|  |  |  |  | n=94 | n=70 |  |  |  |  |
|  |  |  |  | 12/94 (13%) | 21/70 (30%) |  |  |  |  |
|  |  |  |  | RR 0.43 (95% CI 0.15-0.75) |  |  |  |  |  |
|  |  |  |  | p=0.01 |  |  |  |  |  |


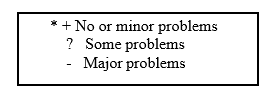
17-OHPC;17-α-hydroxyprogesterone caproate, aRR; adjusted risk ratio, ART; assisted reproductive technology, BMI; body mass index, C; control, CL; cervical length, DCDA; dichorionic diamniotic, DA; diamniotic, DC; dichorionic, GA; gestational age, im; intramuscular, I; intervention, IUFD; intrauterine fetal death, MAR; medically assisted reproduction; MC; monochorionic, MCDA; monochorionic diamniotic, NB; nota bene, NNM; neonatal mortality, OR; odds ratio, PTB; preterm birth, RR; risk ratio, sPTB: spontaneous preterm birth, TVS; transvaginal scan, w; weeks

Prevention of preterm birth

STable 4.1.13. cont. Intervention progesterone

Outcome variable: Respiratory distress syndrome (RDS)

| **Author, year Country**  **Trial acronym** | **Singletons/ Twins/ Triplets** | **Risk factor** | **Number**  **of randomized patients**  **n=** | **Results** | | **Comments** | **Directness *** | **Study limitations** | **Precision *** |
| --- | --- | --- | --- | --- | --- | --- | --- | --- | --- |
|  |  |  |  | **Intervention** | **Control** |  |  |  |  |

* + No or minor problems

? Some problems

- Major problems

Prevention of preterm birth

STable 4.1.14. Intervention progesterone

Outcome variable: Bronchopulmonary dysplasia (BPD)

| **Author, year Country**  **Trial acronym** | **Singletons/ Twins/ Triplets** | **Risk factor** | **Number**  **of randomized patients**  **n=** | **Results** | | **Comments** | **Directness *** | **Study limitations *** | **Precision *** |
| --- | --- | --- | --- | --- | --- | --- | --- | --- | --- |
|  |  |  |  | **Intervention** | **Control** |  |  |  |  |
|  |  |  |  |  |  |  |  |  |  |
| Blackwell, 2020 USA PROLONG | Singletons | Previous singleton sPTB | Randomized 2:1  I: 1130  C: 578 | 17-OHPC 250 mg im/w 6/1093 (0.5%)  RR 3.02 (95% CI 0.38-24.1)  No p value | Placebo 1/559 (0.2%) | Not PO Patients with missing data assumed not to  have the outcome | + | ? | ? |
| Grobman, 2012 USA  SCAN | Singletons | Nulliparous with short TVS CL  <30 mm | I: 327  C: 330 | 17-OHPC 250 mg im/w 3/320 (0.9%)  RR 0.60 (95% CI 0.15-2.51)  No p value | Placebo (castor oil) 5/322 (1.6%) | Not PO | ? | + | + |
| Hassan, 2011 USA + 9 other countries PREGNANT | Singletons | Short TVS CL 10-20 mm (all)  Previous PTB 16% | I: 236  C: 229 | 90 mg vaginal progesterone gel 8%/d 4/235 (1.7%)  RR 0.76 (95% CI 0.21-2.79) p=0.678 | Placebo 5/223 (2.2%) | Not PO | ? | ? | + |
| Meis, 2003 USA | Singletons | Previous sPTB | Randomized 2:1  I:310  C:153 | 17-OHPC 250 mg im/w 4/305 (1.3%)  RR 0.40 (95% CI 0.11-1.46)  No p value | Placebo (castor oil) 5/152 (3.3%) | Not PO | ? | ? | ? |
| Norman, 2016 UK  (UK 65  hospitals, Sweden 1  hospital) OPPTIMUM | Singletons | FFN pos group: Any of previous PTB, second trimester loss, cervical surgery  FFN neg group: previous sPTB <34 w or short TVS CL ≤25 mm | I: 618  C: 610 | 200 mg vaginal progesterone/d 17/580 (3%)  OR 0.94 (95% CI 0.49-1.78) p=0.84 | Placebo 18/574 (3%) | Not PO | + | + | + |
| Van Os, 2015 The Netherlands TRIPLE P | Singletons | Short TVS CL  ≤30 mm,  no previous PTB | I: 41  C: 39 | 200 mg micronized progesterone vaginal/d  0/41  NA  No p value | Placebo 1/39 (3%) | Not PO | + | ? | - |

* + No or minor problems

? Some problems

- Major problems

Prevention of preterm birth

STable 4.1.14. cont. Intervention progesterone

Outcome variable: Bronchopulmonary dysplasia (BPD)

| **Author, year Country**  **Trial acronym** | **Singletons/ Twins/ Triplets** | **Risk factor** | **Number**  **of randomized patients**  **n=** | **Results** | | **Comments** | **Directness *** | **Study limitations *** | **Precision *** |
| --- | --- | --- | --- | --- | --- | --- | --- | --- | --- |
|  |  |  |  | **Intervention** | **Control** |  |  |  |  |
|  |  |  |  |  |  |  |  |  |  |
| Awwad, 2015 Libanon PROGESTWIN | Twins, unselected | ART 75%  MC 17% | Randomized 2:1  I: 197 C:96  Analyzed I:194 women/388  fetuses  C: 94 women/188 fetuses | 17-OHPC 250 mg im/w 6/385 (1.6%)  OR 0.31 (95% CI 0.08-1.23) p=0.10 | Placebo (castor oil) 9/188 (4.8%) | Not PO | + | + | ? |
| Combs, 2011 USA | Twins DCDA | 20% fetal reduction ART  I: 66% C:58%  Prior PTB: I: 12%  C: 13% | Randomized 2:1 I: 160/320  C: 80/160 | 17-OHPC 250 mg (in 1 mL castor oil) im/w  9/308 (3%) OR NA p=0.03 | Placebo (1 mL castor oil) 0/150 | Not PO  In article defined as oxygen use at 28 d of life | + | ? | ? |
| Caritis, 2009 USA | Triplets | 30% DC or unknown chorionicity  ART: 70% | I: 71 women/212 infants  C: 63 women/183 infants | 17-OHPC 250 mg im/w 15/212 (7%)  RR 0.8 (95% CI 0.3-2.0)  No p value | Placebo 17/183 (9%) | Not PO | ? | ? | ? |
| Combs, 2010 USA | Triplets | Trichorionic triamniotic triplets MAR  I: 90%  C: 84% | I: 56/155 liveborn neonates  C: 25 women/75 liveborn neonates | 17-OHPC 250 mg im/w 11/153 (7%)  OR 0.63 (95% CI 0.2-2.5) p=0.51 | Placebo 7/70 (10%) | Not PO  In article defined as oxygen use at 28 d of life | + | ? | ? |
| Lim, 2011  The Netherlands AMPHIA | Multifetal pregnancies | Triplets/+ I: 9 (3%)  C: 9 (3%)  (incl.one quadruplet) MC  I: 57 (17%)  C: 57 (17%)  Fertility treatment I: 140 (42%)  C: 120 (36%) | I: 336 women /681 infants (327 sets of twins, 9 sets of triplets) C: 335 women/  680 infants (326 sets of twins, 8 sets of triplets, one quadruplet) | 17-OHPC 250 mg im/w 16/681 (2%)  RR 1.68 (95% CI 0.64–4.39) | Placebo 9/674 (1%) | Not PO | + | + | ? |

Prevention of preterm birth

STable 4.1.14. cont. Intervention progesterone

Outcome variable: Bronchopulmonary dysplasia (BPD)

* + No or minor problems

? Some problems

- Major problems

| **Author, year Country**  **Trial acronym** | **Singletons/ Twins/ Triplets** | **Risk factor** | **Number**  **of randomized patients**  **n=** | **Results** | | **Comments** | **Directness *** | **Study limitations *** | **Precision *** |
| --- | --- | --- | --- | --- | --- | --- | --- | --- | --- |
|  |  |  |  | **Intervention** | **Control** |  |  |  |  |
|  |  |  |  |  |  |  |  |  |  |
| Wood, 2012 Canada | Twins and triplets | ART I: 55%  C: 60%  Triplets: I: 2 (5%)  C: 1(2%) | I: 42/86 (40 sets of twins, 2 sets of triplets) C: 42/85 (40 sets of twins, one set of triplets) | 90 mg vaginal progesterone gel 8%/d 7/86 (8%)  Difference 0.85  (95% CI 0.32-2.25) p=0.792 | Placebo 8/85 (9%) | Not PO | + | ? | + |
| Crowther, 2017 Australia PROGRESS | Mixed Singletons n=775 (98.5%  Twins n=12 (1.5%) | Previous sPTD  <37 w | I: 398 (390 singletons  and 8 twin pregnancies) 406 infants  C: 389  (385 singletons and 4 twin pregnancies) 393 infants | 100 mg vaginal progesterone pessary/d  10/402 (2.5%)  RR 1.38 (95% CI 0.49–3.87) p=0.542 | Placebo 7/388 (1.8%) | Not PO Chronic lung disease  defined as BPD | + | + | ? |
| **Other interventions in comparison with progesterone** | | | | | | | | | |
| Dang 2019  Vietnam (single center) | Twins | Short TVS  CL ≤38 mm (women with history of cervical surgery excluded) | I:150 C: 150 | Arabin pessary  0/296 (0%)  No statistics Subgroup analysis TVS CL ≤28 mm n=94  0/94 (%) | 400 mg vaginal progesterone/d 0/298 (0%)  Subgroup analysis TVS CL ≤28 mm n=70  0/70 (0%) | Not PO | ? | ? | ? |

17-OHPC;17-α-hydroxyprogesterone caproate, ART; assisted reproductive technology, BPD; bronchopulmonary dysplasia, C; control, CL; cervical length, DCDA; dichorionic diamniotic, DA; diamniotic, DC; dichorionic, GA; gestational age, im; intramuscular, I; intervention, IUFD; intrauterine fetal death, MAR; medically assisted reproduction; MC; monochorionic, MCDA; monochorionic diamniotic, NA; not applicable, NEC; necrotizing enterocolitis, NNM; neonatal mortality, OR; odds ratio, PO; primary outcome PTB; preterm birth, RR; risk ratio, sPTB: spontaneous preterm birth, TVS; transvaginal scan, w; weeks

* + No or minor problems

? Some problems

- Major problems

Prevention of preterm birth

STable 4.1.15. Intervention progesterone

Outcome variable: Intraventricular hemorrhage (IVH)

| **Author, year Country**  **Trial acronym** | **Singletons/ Twins/ Triplets** | | **Risk factor** | **Number**  **of randomized patients**  **n=** | | **Results** | | | | **Comments** | | **Directness *** | | **Study limitations *** | | **Precision *** | |  |  |
| --- | --- | --- | --- | --- | --- | --- | --- | --- | --- | --- | --- | --- | --- | --- | --- | --- | --- | --- | --- |
|  |  |  |  |  |  | **Intervention** | | **Control** | |  |  |  |  |  |  |  |  |  |  |
|  | | |  |  | |  | |  | |  | |  | |  | |  | |  | |
| Ashoush, 2017 Egypt | | | Singletons | Previous sPTB (<37 w) | | I: 106  C: 106 | | 400 mg oral progesterone/d ICH (not defined) 8/96 (8.3%) p=0.55  No RR | | Placebo  ICH (not defined) 11/91 (12%) | | Not PO  NB critical comment to article by Katsanevakis, Mol and Thornton concerning recruitment and results  NB High rates of cerclage in both groups | | - | | ? | | - | |
| Azargoon, 2016 Iran | | | Singletons | Previous PTB (<37 w),  uterine malformations | | I: 51  C: 52 | | 400 mg vaginal progesteron supp/d IVH (grade not defined) 5/50 (10%)  RR 2.0 (95% CI 0.74-5.43) p=0.161 | | Placebo  IVH (grade not defined) 10/50 (20%) | | Not PO | | - | | ? | | - | |
| Blackwell, 2020 USA PROLONG | | | Singletons | Previous singleton sPTB | | I: 1130  C: 578  Randomized 2:1 | | 17-OHPC 250 mg im/w IVH 3-4 2/1093 (0.2%)  RR 0.99 (95% CI 0.09-10.52)  No p value | | Placebo  IVH 3-4 1/559 (0.2%) | | Not PO  Patients with missing data assumed not to have the outcome | | + | | ? | | ? | |
| Grobman, 2012 USA  SCAN | | | Singletons | Nulliparous with short TVS CL  <30 mm | | I: 327  C: 330 | | 17-OHPC 250 mg im/w IVH 3-4 2/320 (0.6%)  RR 2.01 (95% CI 0.18-22.08)  No p value | | Placebo (castor oil) IVH 3-4 1/322 (0.3%) | | Not PO | | ? | | + | | + | |
| Hassan, 2011 USA + 9 other countries PREGNANT | | | Singletons | Short TVS CL 10-20 mm (all)  Previous PTB 16% | | I: 236  C: 229 | | 90 mg vaginal progesterone gel 8%/d  IVH 3-4 0/235 (0.0)  0.32 (0.01-7.73)* p=0.305  No RR | | Placebo  IVH 3-4 1/223 (0.5%) | | Not PO  *Based on Logit estimator with continuity correction | | ? | | ? | | + | |
| Meis, 2003 USA | | | Singletons | Previous sPTB | | I:310 C:153  Randomized 2:1 | | 17-OHPC 250 mg im/w IVH 3-4 2/305 (0.7%) NA  Any IVH 4/305 (1.3%) RR 0.25 (95% CI 0.08-0.82)  No p value | | Placebo (castor oil) IVH 3-4 0/153  Any IVH 8/153 (5.2%) | | Not PO  CI corrected (from incorrect value in article) | | ? | | ? | | ? | |

* + No or minor problems

? Some problems

- Major problems

Prevention of preterm birth

STable 4.1.15. cont. Intervention progesterone

Outcome variable: Intraventricular hemorrhage (IVH)

| **Author, year Country**  **Trial acronym** | **Singletons/ Twins/ Triplets** | | **Risk factor** | **Number**  **of randomized patients**  **n=** | | **Results** | | | | **Comments** | | **Directness *** | | **Study limitations *** | | **Precision *** | |  |  |
| --- | --- | --- | --- | --- | --- | --- | --- | --- | --- | --- | --- | --- | --- | --- | --- | --- | --- | --- | --- |
|  |  |  |  |  |  | **Intervention** | | **Control** | |  |  |  |  |  |  |  |  |  |  |
|  | | |  |  | |  | |  | |  | |  | |  | |  | |  | |
| Norman, 2016 UK  (UK 65 hospitals,  Sweden 1 hospital) OPPTIMUM | | | Singletons | FFN pos group: Any of previous PTB, second trimester loss, cervical surgery  FFN neg group: previous sPTB <34 w or short TVS CL ≤25 mm | | I: 618  C: 610 | | 200 mg vaginal progesterone/d Brain injury on ultrasound scan* 18/584 (3%)  OR 0.50 (95% CI 0.31-0.84) p=0.008  IVH (grade not defined) 7/584 (1.2%)  No statistics | | Placebo  Brain injury on ultrasound scan* 34/574 (6%)  IVH (grade not defined) 13/574 (2.3%) | | Not PO  *any IVH, parenchymal cystic or hemorrhagic lesion, persistent ventriculomegaly | | + | | + | | + | |
| O’Brien 2007  USA (+4 other countries) | | | Singletons | Previous sPTB | | I: 332  C: 327 | | 90 mg vaginal progesterone gel 8%/d  IVH 1-4 6/309 (1.9%)  OR 1.18 (95% CI 0.36 to 3.90)  No p value  IVH 3-4 1/309 (0.3%)  No statistics | | Placebo  IVH 1-4 5/302 (1.6%)  IVH 3-4 1/302 (0.3%) | | Not PO | | ? | | ? | | ? | |
| Van Os, 2015 The Netherlands TRIPLE P | | | Singleton | Short TVS CL  ≤30 mm,  no previous PTB | | I: 41  C: 39 | | 200 mg micronized progesterone vaginal/d  IVH 2-4 0/41 NA | | Placebo  IVH 2-4 0/39 | | Not PO | | + | | ? | | - | |
| Briery, 2009 USA | | | Twins, unselected | 1/3 previous PTB | | I: 16/32 C: 14/28 | | 17-OHPC 250 mg im/w IVH (grade not defined) 3/32 (9%)  p=0.851 No RR | | Placebo  IVH (grade not defined) 4/28 (14%) | | Not PO | | ? | | ? | | - | |
| Brizot, 2015 Brazil | | | Twins DA | MC 25% (I) and 19% (C)  Only naturally conceived, no history of PTB | | I: 195/378 C: 195/382 | | 200 mg vaginal natural progesterone/d  IVH (grade not defined) 4/292 (1.4%)  OR 2.20 (95% CI 0.40-12.06)  No p value | | Placebo  IVH (grade not defined) 2/320 (0.6%) | | Not PO | | ? | | ? | | - | |

* + No or minor problems

? Some problems

- Major problems

Prevention of preterm birth

STable 4.1.15. cont. Intervention progesterone

Outcome variable: Intraventricular hemorrhage (IVH)

| **Author, year Country**  **Trial acronym** | **Singletons/ Twins/ Triplets** | | **Risk factor** | **Number**  **of randomized patients**  **n=** | | **Results** | | | | **Comments** | | **Directness *** | | **Study limitations *** | | **Precision *** | |  |  |
| --- | --- | --- | --- | --- | --- | --- | --- | --- | --- | --- | --- | --- | --- | --- | --- | --- | --- | --- | --- |
|  |  |  |  |  |  | **Intervention** | | **Control** | |  |  |  |  |  |  |  |  |  |  |
|  | | |  |  | |  | |  | |  | |  | |  | |  | |  | |
| Combs, 2011 USA | | | Twins DCDA | 20% fetal reduction ART  I: 66% C:58%  Prior PTB: I: 12%  C: 13% | | I: 160/320 C: 80/160  Randomized 2:1 | | 17-OHPC 250 mg  (in 1 mL castor oil) im/w IVH 3-4 3/316 (1%)  OR NA p=0.55 | | Placebo (1 mL castor oil) IVH 3-4 0/152 | | Not PO | | + | | ? | | ? | |
| Rode, 2011 Denmark and Austria PREDICT | | | Twins DA | MC  I: 43/334 (12.9%)  C: 57/343 (16.6%) | | I: 334/668 C: 343/686 | | 200 mg vaginal progesterone/d (pessary)  IVH (grade not defined) 10/659 (1.5%)  OR 1.7 (95% CI 0.5-5.6)  No p value | | Placebo  IVH (grade not defined) 6/674 (0.9%) | | Not PO | | + | | + | | + | |
| Caritis, 2009 USA | | | Triplets | 30% DC or unknown chorionicity  ART: 70% | | I: 71 women/212 infants  C: 63  women/183 infants | | 17-OHPC 250 mg im/w IVH 3-4 2/212 (0.9%)  RR 0.4 (95% CI 0.0-3.8)  No p value | | Placebo  IVH 3-4 4/183 (2%) | | Not PO | | ? | | ? | | ? | |
| Combs, 2010 USA | | | Triplets | Trichorionic triamniotic triplets MAR  I: 90%  C: 84% | | I: 56/155  liveborn neonates C: 25 women/75 liveborn neonates | | 17-OHPC 250 mg im/w IVH 3-4 4/150 (3%)  OR 0.7 (95% CI 0.1-3.4) p=0.63 | | Placebo  IVH 3-4 3/75 (4%) | | Not PO | | + | | ? | | ? | |
| Lim, 2011  The Netherlands AMPHIA | | | Multi-fetal pregnan-cies | Triplets/+ I: 9 (3%)  C: 9 (3%)  (incl.one quadruplet) MC  I: 57 (17%)  C: 57 (17%)  Fertility treatment I: 140 (42%)  C: 120 (36%) | | I: 336 women  /681 infants (327 sets of twins, 9 sets of triplets) C: 335 women/  680 infants (326 sets of twins, 8 sets of triplets, one quadruplet) | | 17-OHPC 250 mg im/w  IVH grade 2B or worse 4/681 (1%) RR 1.98 (95% CI 0.37-10.7)  No p value | | Placebo  IVH grade 2B or worse 2/674 (0%) | | Not PO | | + | | + | | ? | |

* + No or minor problems

? Some problems

- Major problems

Prevention of preterm birth

STable 4.1.15. cont. Intervention progesterone

Outcome variable: Intraventricular hemorrhage (IVH)

| **Author, year Country**  **Trial acronym** | **Singletons/ Twins/ Triplets** | | **Risk factor** | **Number**  **of randomized patients**  **n=** | | **Results** | | | | **Comments** | | **Directness *** | | **Study limitations *** | | **Precision *** | |  |  |
| --- | --- | --- | --- | --- | --- | --- | --- | --- | --- | --- | --- | --- | --- | --- | --- | --- | --- | --- | --- |
|  |  |  |  |  |  | **Intervention** | | **Control** | |  |  |  |  |  |  |  |  |  |  |
|  | | |  |  | |  | |  | |  | |  | |  | |  | |  | |
| Wood, 2012 Canada | | | Twins and triplets | ART I: 55%  C: 60%  Triplets: I: 2 (5%)  C: 1(2%) | | I: 42/86 (40 sets of twins, 2 sets of triplets)  C: 42/85 (40 sets of twins, one set of triplets) | | 90 mg progesterone vaginal gel 8%/d  IVH 3-4 3/86 (3%)  Difference 2.93 (95% CI 0.31-  27.58) p=0.621 | | Placebo  IVH 3-4 1/85 (1%) | | Not PO | | + | | ? | | + | |
| Crowther, 2017 Australia PROGRESS | | | Mixed Singletons n=775 (98.5%  Twins n=12 (1.5%) | Previous sPTD  <37 w | | I: 398 (390  singletons and 8 twin pregnancies) 406 infants  C: 389  (385 singletons  and 4 twin pregnancies)  393 infants | | 100 mg vaginal progesterone pessary/d  Any IVH 9/402 (2.2%) RR 0.97 (95% CI 0.39-2.41)  p=0.939  IVH 3-4 1/402 (0.3%) p=1.0  RR NA | | Placebo  Any IVH 9/388 (2.3%) IVH 3-4 1/388 (0.3%) | | Not PO | | + | | + | | ? | |
| Fonseca, 2007 Brazil (multicenter inter-  national, UK [5 centers], Chile, Brazil,  Greece) | | | Mixed Singletons (226, 90%)  Twins (24,  10%, all DA) | Short TVS  CL ≤15 mm | | I: 125  women/136 infants (114  singletons, 22 twins)  C: 125  women/138 infants (112  singletons, 26 twins) | | 200 mg vaginal progesterone/d (Utrogestan)  Any IVH 1/136 (0.7%) RR 0.51 (95% CI 0.05-5.30)  p=0.58  aRR 0.33 (95% CI 0.01-8.84) p=0.52  aRR adjusted for maternal age, BMI, smoking status, race, history of PTB, and cervical length at the  time of randomization. | | Placebo  Any IVH 2/138 (1.4%) | | Not PO  IVH was grade 2 in all infants | | ? | | ? | | + | |

* + No or minor problems

? Some problems

- Major problems

Prevention of preterm birth

STable 4.1.15. cont. Intervention progesterone

Outcome variable: Intraventricular hemorrhage (IVH)

| **Author, year Country**  **Trial acronym** | **Singletons/ Twins/ Triplets** | | | **Risk factor** | **Number**  **of randomized patients**  **n=** | | **Results** | | | | **Comments** | | **Directness *** | | **Study limitations *** | | **Precision *** | |  |  |
| --- | --- | --- | --- | --- | --- | --- | --- | --- | --- | --- | --- | --- | --- | --- | --- | --- | --- | --- | --- | --- |
|  |  |  |  |  |  |  | **Intervention** | | **Control** | |  |  |  |  |  |  |  |  |  |  |
|  | | | | | | | | | | | | | | | | | | | | |
| **Other interventions in comparison with progesterone** | | | | | | | | | | | | | | | | | | | | |
| Cruz-Melguizo, 2018 | | | Singletons | Short TVS CL ≤25 mm | | | I: 125  C: 118 | | Pessary 0/125 (0%) | | 200 mg vaginal progesterone/d 1/118 (0.9%) | | Not PO | | + | | ? | | ? | |
| Spain | | |  | (women with cervical | | |  | | No RR | |  | |  | |  | |  | |  | |
| (27 centers) | | |  | surgery and ≥3 previous | | |  | | p=0.31 | |  | |  | |  | |  | |  | |
|  | | |  | PTBs were excluded) | | |  | |  | |  | |  | |  | |  | |  | |
| Dang 2019 | | | Twins | Short TVS  CL ≤38 mm | | | I:150 C: 150 | | Arabin pessary | | 400 mg vaginal progesterone/d | | Not PO | | ? | | ? | | ? | |
| Vietnam | | |  | (women with history of | | |  | | 3/296 (1%) | | 2/298 (1%) | |  | |  | |  | |  | |
| (single center) | | |  | cervical surgery | | |  | | RR 1.51 (95% CI 0.25-9.14) | |  | |  | |  | |  | |  | |
|  | | |  | excluded) | | |  | | p=0.69 | |  | |  | |  | |  | |  | |
|  | | |  |  | | |  | | Subgroup analysis | | Subgroup analysis | |  | |  | |  | |  | |
|  | | |  |  | | |  | | TVS CL ≤28 mm | | TVS CL ≤28 mm | |  | |  | |  | |  | |
|  | | |  |  | | |  | | n=94 | | n=70 | |  | |  | |  | |  | |
|  | | |  |  | | |  | | 3/94 (3%) | | 1/70 (1%) | |  | |  | |  | |  | |
|  | | |  |  | | |  | | RR 2.23 (95% CI 0.23-22.34) | |  | |  | |  | |  | |  | |
|  | | |  |  | | |  | | p=0.64 | |  | |  | |  | |  | |  | |

17-OHPC;17-α-hydroxyprogesterone caproate, ART; assisted reproductive technology, BMI; body mass index, C; control, CI; confidence interval, CL; cervical length, DA; diamniotic, DCDA; dichorionic diamniotic, DC; dichorionic, im; intramuscular, I; intervention, ICH; intracranial hemorrhage, IUFD; intrauterine fetal death, MAR; medically assisted reproduction; MC; monochorionic, MCDA; monochorionic diamniotic, NA; not applicable, OR; odds ratio, PTB; preterm birth, RR; risk ratio, sPTB: spontaneous preterm birth, TVS; transvaginal scan, w; week

Prevention of preterm birth

STable 4.1.16. Intervention progesterone

Outcome variable: Necrotizing enterocolitis (NEC)

* + No or minor problems

? Some problems

- Major problems

| **Author, year Country**  **Trial acronym** | **Singletons/ Twins/ Triplets** | **Risk factor** | **Number**  **of randomized patients**  **n=** | **Results** | | **Comments** | **Directness *** | **Study limitations *** | **Precision *** |
| --- | --- | --- | --- | --- | --- | --- | --- | --- | --- |
|  |  |  |  | **Intervention** | **Control** |  |  |  |  |
|  |  |  |  |  |  |  |  |  |  |
| Ashoush, 2017 Egypt | Singletons | Previous sPTB (<37 w) | I: 106  C: 106 | 400 mg oral progesterone/d 5/96 (5.2%)  p=0.36 | Placebo 9/91 (9.8%) | Not PO  NB critical comment to article by Katsanevakis, Mol and Thornton concerning recruitment and results  NB High rates of cerclage in both groups | - | ? | - |
| Azargoon, 2016 Iran | Singletons | Previous PTB (<37 w),  uterine malformations | I: 51  C: 52 | 400 mg vaginal progesterone supp/d  4/50 (8%)  RR 2.25 (95% CI 0.74-6.83) p=0.137 | Placebo 9/50 (18%) | Not PO | - | ? | - |
| Blackwell, 2020 USA PROLONG | Singletons | Previous singleton sPTB | Randomized2:1  I: 1130  C: 578 | 17-OHPC 250 mg im/w 2/1093 (0.2%)  RR 0.5 (95% CI 0.07-3.40)  No p value | Placebo 2/559 (0.4%) | Not PO  Patients with missing data  assumed not to have the outcome | + | ? | ? |
| Grobman, 2012 USA  SCAN | Singletons | Nulliparous with short TVS CL  <30 mm | I: 327  C: 330 | 17-OHPC 250 mg im/w NEC grade II-III 2/320 (0.6%) RR 0.40 (95% CI 0.08-2.06)  No p value | Placebo (castor oil)  NEC grade II-III  5/322 (1.6%) | Not PO NEC II-III | ? | + | + |
| Hassan, 2011 USA + 9 other countries PREGNANT | Singletons | Short TVS CL 10-20 mm (all)  Previous PTB 16% | I: 236  C: 229 | 90 mg vaginal progesterone gel 8%/d  5/235 (2.1%)  RR 1.19 (95% CI 0.32-4.36) p=0.797 | Placebo 4/223 (1.8%) | Not PO ITT analysis set | ? | ? | + |
| Majhi, 2009 India | Singletons | Previous sPTB | I: 50  C: 50 | 100 mg vaginal progesterone/d 0/50  p=0.31 | No placebo 1/50 (2%) | Not PO | ? | ? | ? |
| Meis, 2003 USA | Singletons | Previous sPTB | Randomized  2:1  I:310  C:153 | 17-OHPC 250 mg im/w 0/305  No p-value | Placebo (castor oil) 4/152 (2.6%) | Not PO | ? | ? | ? |

Prevention of preterm birth

STable 4.1.16. cont. Intervention progesterone

Outcome variable: Necrotizing enterocolitis (NEC)

* + No or minor problems

? Some problems

- Major problems

| **Author, year Country**  **Trial acronym** | **Singletons/ Twins/ Triplets** | **Risk factor** | **Number**  **of randomized patients**  **n=** | **Results** | | **Comments** | **Directness *** | **Study limitations *** | **Precision *** |
| --- | --- | --- | --- | --- | --- | --- | --- | --- | --- |
|  |  |  |  | **Intervention** | **Control** |  |  |  |  |
|  |  |  |  |  |  |  |  |  |  |
| Norman, 2016 UK  (UK 65 hospitals,  Sweden 1 hospital) OPPTIMUM | Singletons | FFN pos group: Any of previous PTB, second trimester loss, cervical surgery FFN neg group: previous sPTB  <34 w or  TVS CL ≤25 mm | I: 618  C: 610 | 200 mg vaginal progesterone/d Suspected or confirmed NEC 18/581 (3%)  OR 1.37 (95% CI 0.76-2.45) p=0.29 | Placebo Suspected or confirmed NEC  13/574 (2%) | Not PO | + | + | + |
| O’Brien 2007  USA (+4 other countries) | Singletons | Previous sPTB | I: 332  C: 327 | 90 mg vaginal progesterone gel 8%/d  3/309 (1.0%)  OR 0.58 (95% CI 0.14-2.46)  No p value | Placebo 5/302 (1.7%) | Not PO | ? | ? | ? |
| Van Os, 2015 The Netherlands TRIPLE P | Singletons | Short TVS CL  ≤30 mm,  no previous PTB | I: 41  C: 39 | 200 mg micronized progesterone vaginal/d  NEC >stage 1 0/41 NA  No p value | Placebo NEC >stage 1 0/39 | Not PO | + | ? | - |
| Awwad, 2015 Libanon PROGESTWIN | Twins, unselected | ART 75%  MC 17% | Randomized 2:1  I: 197 C:96  Analyzed I:194 women/388  fetuses C: 94  women/188 fetuses | 17-OHPC 250 mg im/w 4/386 (1.0%)  OR 0.32 (95% CI 0.06–1.57) p=0.16 | Placebo (castor oil) 6/188 (3.2%) | Not PO | + | + | ? |
| Briery, 2009 USA | Twins, unselected | 1/3 previous PTB | I: 16/32 C: 14/28 | 17-OHPC 250 mg im/w  1/32 (3%) p=0.946 | Placebo 0/28 | Not PO | ? | ? | - |
| Brizot, 2015 Brazil | Twins DA | MC 25% (I) and 19% (C) Only  naturally conceived, no history of PTB | I: 195/378 C: 195/382 | 200 mg vaginal natural progesterone/d 1/293 (0.3%)  OR 0.54 (95% CI 0.05-6.02) | Placebo  2/320 (0.6%) | Not PO | ? | ? | - |

Prevention of preterm birth

STable 4.1.16. cont. Intervention progesterone

Outcome variable: Necrotizing enterocolitis (NEC)

* + No or minor problems

? Some problems

- Major problems

| **Author, year Country**  **Trial acronym** | **Singletons/ Twins/ Triplets** | **Risk factor** | **Number**  **of randomized patients**  **n=** | **Results** | | **Comments** | **Directness *** | **Study limitations *** | **Precision *** |
| --- | --- | --- | --- | --- | --- | --- | --- | --- | --- |
|  |  |  |  | **Intervention** | **Control** |  |  |  |  |
|  |  |  |  |  |  |  |  |  |  |
| Combs, 2011 USA | Twins DCDA | 20% fetal reduction ART  I: 66% C:58%  Prior PTB: I: 12%  C: 13% | Randomized 2:1 I: 160/320  C: 80/160 | 17-OHPC 250 mg (in 1 mL castor oil) im/w  0/315  OR NA | Placebo (1 mL castor oil) 0/152 | Not PO | + | ? | ? |
| Caritis, 2009 USA | Triplets | 30% DC or  unknown chorionicity ART:  70% | I: 71 women/212 infants  C: 63  women/183 infants | 17-OHPC 250 mg im/w 2/212 (0.9%)  RR 0.3 (95% CI 0.0-3.1) | Placebo 5/183 (3%) | Not PO | ? | ? | ? |
| Combs, 2010 USA | Triplets | Trichorionic triamniotic triplets MAR  I: 90%  C: 84% | I: 56/155  liveborn neonates C: 25 women/75 liveborn neonates | 17-OHPC 250 mg im/w NEC requiring surgery 8/154 (5%)  OR 1.4 (95% CI 0.2-7.6) p=0.73 | Placebo  NEC requiring surgery 3/75 (4%) | Not PO | + | ? | ? |
| Lim, 2011  The Netherlands AMPHIA | Multifetal pregnancies | Triplets/+ I: 9 (3%)  C: 9 (3%)  (incl.one quadruplet) MC  I: 57 (17%)  C: 57 (17%)  Fertility treatment I: 140 (42%)  C: 120 (36%) | I: 336 women  /681 infants (327 sets of twins, 9 sets of triplets) C: 335 women/  680 infants (326 sets of twins, 8 sets of triplets, one quadruplet) | 17-OHPC 250 mg im/w 8/681 (1%)  RR 1.59 (95% CI 0.50-5.06) | Placebo 5/674 (1%) | Not PO | + | + | ? |

* + No or minor problems

? Some problems

- Major problems

Prevention of preterm birth

STable 4.1.16. cont. Intervention progesterone

Outcome variable: Necrotizing enterocolitis (NEC)

| **Author, year Country**  **Trial acronym** | **Singletons/ Twins/ Triplets** | **Risk factor** | **Number**  **of randomized patients**  **n=** | **Results** | | **Comments** | **Directness *** | **Study limitations *** | **Precision *** |
| --- | --- | --- | --- | --- | --- | --- | --- | --- | --- |
|  |  |  |  | **Intervention** | **Control** |  |  |  |  |
|  |  |  |  |  |  |  |  |  |  |
| Rode, 2011 Denmark and Austria PREDICT | Twins DA | MC  I: 43/334 (12.9%)  C: 57/343 (16.6%) | I: 334/668 C: 343/686 | 200 mg vaginal progesterone/d (pessary)  1/659 (0.2%)  OR 0.5 (95% CI 0.0-5.6)  No p values | Placebo 2/674 (0.3%) | Not PO | + | + | + |
| Wood, 2012 Canada | Twins and triplets | ART I: 55%  C: 60%  Triplets: I: 2 (5%)  C: 1(2%) | I: 42/86 (40 sets of twins, 2 sets of triplets)  C: 42/85 (40 sets of twins, one set  of triplets) | 90 mg vaginal progesterone gel 8%/d  1/86 (1%)  Difference 0.49  (95% CI 0.05-5.28) p=0.618 | Placebo 2/85 (2%) | Not PO | + | ? | + |
| Crowther, 2017 Australia PROGRESS | Mixed Singletons n=775 (98.5%  Twins n=12 (1.5%) | Previous sPTD  <37 w | I: 398 (390  singletons and 8 twin pregnancies) 406 infants  C: 389  (385 singletons  and 4 twin pregnancies) 393 infants | 100 mg vaginal progesterone pessary/d  2/402 (0.5%) p=1.0  RR NA | Placebo 2/388 (0.5%) | Not PO | + | + | ? |
| Fonseca, 2007 Brazil (multicenter inter- national, UK [5 centers], Chile, Brazil,  Greece) | Mixed Singletons (226,  90%)  Twins (24, 10%, all DA) | Short TVS CL  ≤15 mm | I: 125  women/136 infants (114  singletons, 22 twins)  C: 125  women/138 infants (112  singletons, 26 twins) | 200 mg vaginal progesterone /day (Utrogestan)  0/136  Statistics NA | Placebo 1/138 (0.7%) | Not PO | ? | ? | + |

* + No or minor problems

? Some problems

- Major problems

Prevention of preterm birth

STable 4.1.16. cont. Intervention progesterone

Outcome variable: Necrotizing enterocolitis (NEC)

| **Author, year Country**  **Trial acronym** | **Singletons/ Twins/ Triplets** | **Risk factor** | **Number**  **of randomized patients**  **n=** | **Results** | | **Comments** | **Directness *** | **Study limitations *** | **Precision *** |
| --- | --- | --- | --- | --- | --- | --- | --- | --- | --- |
|  |  |  |  | **Intervention** | **Control** |  |  |  |  |
|  | | | | | | | | | |
| **Other interventions in comparison with progesterone** | | | | | | | | | |
| Cruz-Melguizo, 2018 | Singletons | Short TVS CL ≤25 mm | I: 125  C: 118 | Pessary 2/125 (1.7%) | 200 mg vaginal progesterone/d 0/118 (0%) | Not PO | + | ? | ? |
| Spain |  | (women with |  | No RR |  |  |  |  |  |
| (27 centers) |  | cervical surgery |  | p=0.16 |  |  |  |  |  |
|  |  | and ≥3 previous |  |  |  |  |  |  |  |
|  |  | PTBs were |  |  |  |  |  |  |  |
|  |  | excluded) |  |  |  |  |  |  |  |
| Dang 2019 | Twins | Short TVS  CL ≤38 mm | I:150 C: 150 | Arabin pessary 8/296 (3%) | 400 mg vaginal progesterone/d 18/298 (6%) | Not PO | ? | ? | ? |
| Vietnam |  | (women with |  | RR 0.45 (95% CI 0.18-1.01) |  |  |  |  |  |
| (single center) |  | history of cervical |  | p=0.07 |  |  |  |  |  |
|  |  | surgery excluded) |  | Subgroup analysis | Subgroup analysis |  |  |  |  |
|  |  |  |  | TVS CL ≤28 mm | TVS CL ≤28 mm |  |  |  |  |
|  |  |  |  | n=94 | n=70 |  |  |  |  |
|  |  |  |  | 4/94 (4%) | 7/70 (10%) |  |  |  |  |
|  |  |  |  | RR 0.43 (95% CI 0.11-1.42) |  |  |  |  |  |
|  |  |  |  | p=0.21 |  |  |  |  |  |

17-OHPC;17-α-hydroxyprogesterone caproate, ART; assisted reproductive technology, C; control, CL; cervical length, DCDA; dichorionic diamniotic, DA; diamniotic, DC; dichorionic, GA; gestational age, im; intramuscular, I; intervention, IUFD; intrauterine fetal death, MAR; medically assisted reproduction; MC; monochorionic, MCDA; monochorionic diamniotic, NEC; necrotizing enterocolitis, NNM; neonatal mortality, OR; odds ratio, PO; primary outcome, PTB; preterm birth, RR; risk ratio, sPTB: spontaneous preterm birth, TVS; transvaginal scan, w; week

| Blackwell, 2020 USA PROLONG | Singletons | Previous singleton sPTB | I: 1130  C: 578  Randomized 2:1 | 17-OHPC 250 mg im/w Proven sepsis 5/1093 (0.5%) RR 0.84 (95% CI 0.20-3.56)  No p value | Placebo  Proven sepsis 3/559 (0.5%) | Not PO  Patients with missing data assumed not to have the outcome | + | ? | ? |
| --- | --- | --- | --- | --- | --- | --- | --- | --- | --- |
| Grobman, 2012 USA  SCAN | Singletons | Nulliparous with short TVS CL  <30 mm | I: 327  C: 330 | 17-OHPC 250 mg im/w Early onset sepsis 3/320 (0.9%)  RR 0.27 (95% CI 0.08-0.97)  No p value | Placebo (castor oil)  Early onset sepsis 11/322 (3.4%) | Not PO | ? | + | + |
| Hassan, 2011 USA + 9 other countries PREGNANT | Singletons | Short TVS CL 10-20 mm (all)  Previous PTB 16% | I: 236  C: 229 | 90 mg vaginal progesterone gel 8%/d  Proven sepsis 7/235 (3.0%)  RR 1.11 (95% CI 0.38–3.24) p=0.853 | Placebo  Proven sepsis 6/223 (2.7%) | Not PO | ? | ? | + |
| Majhi, 2009 India | Singletons | Previous sPTB | I: 50  C: 50 | 100 mg vaginal progesterone/d 0/50  p=0.16 | No placebo 3/50 (6%) | Not PO | ? | ? | ? |
| Meis, 2003 USA | Singletons | Previous sPTB | Randomized2:1  I:310  C:153 | 17-OHPC 250 mg im/w Proven sepsis 9/305 (3.0%) RR 1.12 (95% CI 0.35-3.58)  No p value | Placebo (castor oil) Proven sepsis 4/152 (2.6%) | Not PO | ? | ? | ? |
| Norman, 2016 UK  (UK 65 hospitals,  Sweden 1 hospital) OPPTIMUM | Singletons | FFN pos group: Any of previous PTB, second trimester loss, cervical surgery FFN neg group: previous sPTB  <34 w or  TVS CL ≤25 mm | I: 618  C: 610 | 200 mg vaginal progesterone/d Neonatal infection (positive blood or CNS culture)  44/537 (8%)  OR 1.22 (95% CI 0.79-1.88) p=0.36 | Placebo  Neonatal infection (positive blood or CNS culture)  36/573 (6%) | Not PO | + | + | + |
| Van Os, 2015 The Netherlands TRIPLE P | Singletons | Short TVS CL  ≤30 mm,  no previous PTB | I: 41  C: 39 | 200 mg micronized progesterone vaginal/d  Proven sepsis 0/41 NA | Placebo  Proven sepsis 0/39 | Not PO | + | ? | - |
| Yemini, 1985 Israel | Singletons | Previous ≥2 PTB or  ≥2 spontaneous miscarriages | I: 39  C: 40 | 17-OHPC 250 mg im/w 1/5 (20%)  No statistics | Placebo 2/14 (14.3%) | Not PO  All had cerclage Denominator = infants born  < 36 w | - | - | - |

Cont.

| **Author, year Country**  **Trial acronym** | **Singletons/ Twins/ Triplets** | **Risk factor** | **Number**  **of randomized**  **patients**  **n=** | **Results** | | **Comments** | **Directness *** | **Study limitations *** | **Precision *** |
| --- | --- | --- | --- | --- | --- | --- | --- | --- | --- |
|  |  |  |  | **Intervention** | **Control** |  |  |  |  |

* + No or minor problems

? Some problems

- Major problems

Prevention of preterm birth

STable 4.1.17. Intervention progesterone

Outcome variable: Neonatal sepsis

| Awwad, 2015 Libanon PROGESTWIN | Twins, unselected | ART 75%  MC 17% | Randomized 2:1  I: 197 C:96  Analyzed I:194 women/ 388 fetuses  C: 94 women/  188 fetuses | 17-OHPC 250 mg im/w Proven sepsis 13/384 (3.4%) OR 0.24 (95% CI 0.10-0.57) p=0.00 | Placebo (castor oil) Proven sepsis 24/188 (12.8%) | Not PO | + | + | ? |
| --- | --- | --- | --- | --- | --- | --- | --- | --- | --- |
| Brizot, 2015 Brazil | Twins DA | MC 25% (I) and 19% (C) Only naturally conceived, no history of PTB | I: 195/378 C: 195/382 | 200 mg vaginal natural progesterone/d 15/291 (5.2%)  OR 2.16 (95% CI 0.87-5.33)  No p value | Placebo 8/320 (2.5%) | Not PO | ? | ? | - |
| Combs, 2011 USA | Twins DCDA | 20% fetal reduction ART  I: 66% C:58%  Prior PTB: I: 12%  C: 13% | Randomized 2:1 I: 160/320  C: 8/160 | 17-OHPC 250 mg (in 1 mL castor oil) im/w  Proven sepsis 3/319 (1%) OR 1.5 (95% CI 0.1-16.8) p=0.77 | Placebo (1 mL castor oil) Proven sepsis 1/154 (1%) | Not PO | + | ? | ? |
| Rode, 2011 Denmark and Austria PREDICT | Twins DA | MC  I: 43/334 (12.9%)  C: 57/343 (16.6%) | I: 334/668 C: 343/686 | 200 mg vaginal progesterone/d (pessary)  Septicemia 20/659 (3.0%)  OR 1.1 (95% CI 0.5-2.4)  No p values | Placebo Septicemia 18/674 (2.7%) | Not PO | + | + | + |
| Caritis, 2009 USA | Triplets | 30% DC or  unknown chorionicity ART:  70% | I: 71 women/212 infants  C: 63  women/183 infants | 17-OHPC 250 mg im/w Proven sepsis 20/212 (9%) RR 1.3 (95% CI 0.6–3.0)  No p value | Placebo  Proven sepsis 13/183 (7%) | Not PO | ? | ? | ? |
| Combs, 2010 USA | Triplets | Trichorionic triamniotic triplets MAR  I: 90%  C: 84% | I: 56 women /155 liveborn neonates  C:25 women/75 liveborn neonates | 17-OHPC 250 mg im/w Proven sepsis 4/154 (3%) OR 0.49 (95% CI 0.1-2.2) p=0.36 | Placebo  Proven sepsis 4/75 (5%) | Not PO | + | ? | ? |

Cont.

| **Author, year Country**  **Trial acronym** | **Singletons/ Twins/ Triplets** | **Risk factor** | **Number**  **of randomized**  **patients**  **n=** | **Results** | | **Comments** | **Directness *** | **Study limitations *** | **Precision *** |
| --- | --- | --- | --- | --- | --- | --- | --- | --- | --- |
|  |  |  |  | **Intervention** | **Control** |  |  |  |  |

* + No or minor problems

? Some problems

- Major problems

Prevention of preterm birth

STable 4.1.17. cont. Intervention progesterone

Outcome variable: Neonatal sepsis

| Lim, 2011  The Netherlands AMPHIA | Multifetal pregnancies | Triplets/+ I: 9 (3%)  C: 9 (3%)  (incl.one quadruplet) MC  I: 57 (17%)  C: 57 (17%)  Fertility treatment I: 140 (42%)  C: 120 (36%) | I: 336 women  /681 infants (327 sets of twins, 9 sets of triplets) C: 335 women/  680 infants (326 sets of twins, 8 sets of triplets, one quadruplet) | 17-OHPC 250 mg im/w Proven sepsis 23/681 (3%) RR 2.06 (95% CI 0.91-4.66) | Placebo  Proven sepsis 11/674 (2%) | Not PO | + | + | ? |
| --- | --- | --- | --- | --- | --- | --- | --- | --- | --- |
| Crowther, 2017 Australia PROGRESS | Mixed Singletons n=775 (98.5%  Twins n=12 (1.5%) | Previous sPTD  <37 w | I: 398 (390  singletons and 8 twin pregnancies)/ 406 infants  C: 389  (385 singletons  and 4 twin pregnancies)/  393 infants | 100 mg vaginal progesterone pessary/d  Proven early sepsis 0/402 (0%) p=0.24  RR NA | Placebo  Proven early sepsis 2/388 (0.5%) | Not PO | + | + | ? |
| Fonseca, 2007 Brazil (multicenter inter- national, UK [5 centers], Chile, Brazil,  Greece) | Mixed Singletons (226,  90%)  Twins (24, 10%, all DA) | Short TVS CL ≤15 mm | I: 125  women/136 infants (114  singletons, 22 twins)  C: 125  women/138 infants (112  singletons, 26 twins) | 200 mg vaginal progesterone /day (Utrogestan)  Treatment for proved or suspected sepsis 3/136 (2.2%)  RR 0.28 (95% CI 0.07-1.01) p=0.05  aRR 0.29 (95% CI 0.07-1.10) p=0.07  aRR adjusted for maternal age, BMI, smoking status, race, history of PTB, and TVS CL at the time of | Placebo  Treatment for proved or suspected sepsis 11/138 (8.0%) | Not PO | ? | ? | + |

Cont.

* + No or minor problems

? Some problems

- Major problems

Prevention of preterm birth

STable 4.1.17. cont. Intervention progesterone

Outcome variable: Neonatal sepsis

| **Author, year Country**  **Trial acronym** | **Singletons/ Twins/ Triplets** | **Risk factor** | **Number**  **of randomized**  **patients**  **n=** | **Results** | | **Comments** | **Directness *** | **Study limitations *** | **Precision *** |
| --- | --- | --- | --- | --- | --- | --- | --- | --- | --- |
|  |  |  |  | **Intervention** | **Control** |  |  |  |  |

|  | | | | | | | | | |
| --- | --- | --- | --- | --- | --- | --- | --- | --- | --- |
| **Other interventions in comparison with progesterone** | | | | | | | | | |
| Dang 2019 | Twins | Short TVS  CL ≤38 mm | I:150 C: 150 | Arabin pessary | 400 mg vaginal progesterone/d | Not PO | ? | ? | ? |
| Vietnam |  | (women with |  | 17/296 (6%) | 33/298 (11%) |  |  |  |  |
| (single center) |  | history of cervical |  | RR 0.52 (95% CI 0.27-0.90) |  |  |  |  |  |
|  |  | surgery excluded) |  | p=0.03 |  |  |  |  |  |
|  |  |  |  | Subgroup analysis | Subgroup analysis |  |  |  |  |
|  |  |  |  | TVS CL ≤28 mm | TVS CL ≤28 mm |  |  |  |  |
|  |  |  |  | n=94 | n=70 |  |  |  |  |
|  |  |  |  | 6/94 (6%) | 15/70 (21%) |  |  |  |  |
|  |  |  |  | RR 0.30 (95% CI 0.09-0.68) |  |  |  |  |  |
|  |  |  |  | p=0.01 |  |  |  |  |  |

17-OHPC;17-α-hydroxyprogesterone caproate, ART; assisted reproductive technology, BMI; body mass index, C; control, CL; cervical length, d; day, DA; diamniotic, DCDA; dichorionic diamniotic, DC; dichorionic, FFN; fetal fibronectin, im; intramuscular, I; intervention, IUFD; intrauterine fetal death, MAR; medically assisted reproduction; MC; monochorionic, MCDA; monochorionic diamniotic, NA; not applicable, NEC; necrotizing enterocolitis, OR; odds ratio, PO; primary outcome, PTB; preterm birth, RR; risk ratio, sPTB: spontaneous preterm birth, TVS; transvaginal scan, w; week

| **Author, year Country**  **Trial acronym** | **Singletons/ Twins/ Triplets** | **Risk factor** | **Number**  **of randomized**  **patients**  **n=** | **Results** | | **Comments** | **Directness *** | **Study limitations *** | **Precision *** |
| --- | --- | --- | --- | --- | --- | --- | --- | --- | --- |
|  |  |  |  | **Intervention** | **Control** |  |  |  |  |

* + No or minor problems

? Some problems

- Major problems

Prevention of preterm birth

STable 4.1.17. cont. Intervention progesterone

Outcome variable: Neonatal sepsis

* + No or minor problems

? Some problems

- Major problems

| **Author, year Country**  **Trial acronym** | **Singletons/ Twins/ Triplets** | **Risk factor** | **Number**  **of randomized patients**  **n=** | **Results** | | **Comments** | **Directness *** | **Study limitations *** | **Precision *** |
| --- | --- | --- | --- | --- | --- | --- | --- | --- | --- |
|  |  |  |  | **Intervention** | **Control** |  |  |  |  |
|  |  |  |  |  |  |  |  |  |  |
| Blackwell, 2020 USA PROLONG | Singletons | Previous singleton sPTB | Randomized 2:1  I: 1130  C: 578 | 17-OHPC 250 mg im/w 5/1093 (0.5%)  RR 0.37 (95% CI (0.12-1.16)  No p value | Placebo 7/559 (1.3%) | Not PO Patients with missing data assumed not to  have the outcome | + | ? | ? |
| Grobman, 2012 USA  SCAN | Singletons | Nulliparous with short TVS CL  <30 mm | I: 327  C: 330 | 17-OHPC 250 mg im/w ROP grade 3-4 1/320 (0.3%)  RR 0.34 (95% CI 0.04-3.21)  No p value | Placebo (castor oil) ROP grade 3-4 3/322 (0.9%) | Not PO ROP 3-4 | ? | + | + |
| Meis, 2003 USA | Singletons | Previous sPTB | Randomized 2:1 I:310 C:153 | 17-OHPC 250 mg im/w 5/305 (1.6%)  RR 0.50 (95% CI 0.15-1.70)  No p value | Placebo (castor oil) 5/152 (3.3%) | Not PO | ? | ? | ? |
| Awwad, 2015 Libanon PROGESTWIN | Twins, unselected | ART 75%  MC 17% | Randomized 2:1  I: 197 C:96  Analyzed I:194 women/388  fetuses  C: 94 women/188 fetuses | 17-OHPC 250 mg im/w 4/379 (1.1%)  OR 0.21 (95% CI 0.05-0.96) p=0.04 | Placebo (castor oil) 9/186 (4.6%) | Not PO | + | + | ? |
| Brizot, 2015 Brazil | Twins DA | MC 25% (I) and 19% (C) Only naturally conceived, no history of PTB | I: 195/378 C: 195/382 | 200 mg vaginal natural progesterone/d 6/292 (2.1%)  OR 1.27 (95% CI 0.32-5.00)  No p value | Placebo 6/320 (1.9%) | Not PO | ? | ? | - |
| Combs, 2011 USA | Twins DCDA | 20% fetal reduction ART  I: 66% C:58%  Prior PTB: I: 12%  C: 13% | Randomized 2:1 I: 160/320  C: 80/160 | 17-OHPC 250 mg (in 1 mL castor oil) im/w  2/308 (0.6%) RR/OR NA p=1.0 | Placebo (1 mL castor oil) 0/145 | Not PO | + | ? | ? |
| Rode, 2011 Denmark and Austria PREDICT | Twins DA | MC  I: 43/334 (12.9%)  C: 57/343 (16.6%) | I: 334/668 C: 343/686 | 200 mg vaginal progesterone/d (pessary)  4/659 (0.6%)  OR 1.0 (95% CI 0.2–4.8)  No p value | Placebo 4/674 (0.6%) | Not PO | + | + | + |

Prevention of preterm birth

STable 4.1.18. Intervention progesterone

Outcome variable: Retinopathy of prematurity

* + No or minor problems

? Some problems

- Major problems

| **Author, year Country**  **Trial acronym** | **Singletons/ Twins/ Triplets** | **Risk factor** | **Number**  **of randomized patients**  **n=** | **Results** | | **Comments** | **Directness *** | **Study limitations *** | **Precision *** |
| --- | --- | --- | --- | --- | --- | --- | --- | --- | --- |
|  |  |  |  | **Intervention** | **Control** |  |  |  |  |
|  |  |  |  |  |  |  |  |  |  |
| Caritis, 2009 USA | Triplets | 30% DC or  unknown  chorionicity ART: 70% | I: 71 women/212 infants C: 63 women/183 infants | 17-OHPC 250 mg im/w Severe ROP ≥grade 3 0/212 RR/OR NA | Placebo  Severe ROP ≥grade 3 0/183 | Not PO | ? | ? | ? |
| Combs, 2010 USA | Triplets | Trichorionic triamniotic triplets MAR  I: 90%  C: 84% | I: 56/155 liveborn neonates  C: 25 women/75 liveborn neonates | 17-OHPC 250 mg im/w 4/145 (3%)  OR 0.4 (95% CI 0.1-3.0) p=0.39 | Placebo 4/62 (6.5%) | Not PO | + | ? | ? |
| Crowther, 2017 Australia PROGRESS | Mixed Singletons n=775 (98.5%  Twins n=12 (1.5%) | Previous sPTD  <37 w | I: 398 (390 singletons and 8 twin pregnancies) 406 infants  C: 389  (385 singletons and 4 twin pregnancies) 393 infants | 100 mg vaginal progesterone pessary/d  12/401 (3.0%)  RR 1.28 (95% CI 0.51-3.26) p=0.600 | Placebo 9/386 (2.3%) | Not PO | + | + | ? |
| Fonseca, 2007 Brazil (multicenter inter- national, UK [5  centers], Chile, Brazil, Greece) | Mixed Singletons (226,  90%)  Twins (24, 10%, all DA) | Short TVS CL ≤15  mm | I: 125 women/136  infants (114 singletons,  22 twins)  C: 125 women/138  infants (112 singletons, 26 twins) | 200 mg vaginal progesterone /day (Utrogestan)  2/136 (1.5%)  Statistics NA | Placebo 0/138 | Not PO | ? | ? | + |
| **Other interventions in comparison with progesterone** | | | | | | | | | |
| Cruz-Melguizo, 2018  Spain  (27 centers) | Singletons | Short TVS CL ≤25 mm (women with cervical surgery and ≥3 previous  PTBs were excluded) | I: 125  C: 118 | Pessary 2/125 (1.7%) No RR p=0.58 | 200 mg vaginal progesterone/d 1/118 (0.9%) | Not PO | + | ? | ? |

17-OHPC;17-α-hydroxyprogesterone caproate, ART; assisted reproductive technology, C; control, CL; cervical length, DCDA; dichorionic diamniotic, DA; diamniotic, DC; dichorionic, GA; gestational age, I; intervention, IUFD; intrauterine fetal death, MAR; medically assisted reproduction; MC; monochorionic, MCDA; monochorionic diamniotic, NA; not applicable, NEC; necrotizing enterocolitis, NNM; neonatal mortality, OR; odds ratio, PTB; preterm birth, ROP; retinopathy of prematurity; RR; risk ratio, sPTB: spontaneous preterm birth, TVS; transvaginal scan

* + No or minor problems

? Some problems

- Major problems

Prevention of preterm birth

STable 4.1.18. cont. Intervention progesterone

Outcome variable: Retinopathy of prematurity

* + No or minor problems

? Some problems

- Major problems

Prevention of preterm birth

STable 4.1.19. Intervention progesterone

Outcome variable: Admittance to neonatal intensive care unit (NICU)

| **Author, year Country**  **Trial acronym** | **Singletons/ Twins/ Triplets** | **Risk factor** | **Number of**  **randomizedpatients**  **n=** | **Results** | | **Comments** | **Directness *** | **Study limitations *** | **Precision *** |
| --- | --- | --- | --- | --- | --- | --- | --- | --- | --- |
|  |  |  |  | **Intervention** | **Control** |  |  |  |  |
|  |  |  |  |  |  |  |  |  |  |
| Aflatoonian, 2013  Iran | Singletons | ART pregnancies | I: 52  C: 47 | 17-OHPC 250 mg im/w 5/52 (9.6%)*  No RR p=0.24 | Placebo 9/47 (19.1%)* | Not PO  *Figures from article text.  In Table IV: NICU  admission 47/52 (90.4%) and 38/47 (80.9 %) p=0.24 | ? | - | - |
| Ali, 2020  Egypt | Singletons | Indication for cerclage: previous  second trimester loss, sPTD (<34 w) or short cervix  (<25 mm) | I: 121 C:121 | McDonald cerclage +  400 mg vaginal progesterone (pessary)/d 8/100* (8.0%)  No RR p=0.044 | McDonald cerclage  +Placebo 15/84* (17.9%) | Not PO  *Denominator includes only those women who completed the study period; excludes those who stopped treatment before 28 wk, those who were lost to follow-up, and those who had an abortion before 28 w (21/121 and 37/121)  NB discrepancy with data in Table 2 8.5% and 20%, respectively | ? | ? | ? |
| Ashoush, 2017 Egypt | Singletons | Previous sPTB (<37 w) | I: 106  C: 106 | 400 mg oral progesterone/d 22/96 (22.9%)  p<0.001 No RR  Duration of NICU stay, mean (SD) 15.4±5.5 days  p=0.008 | Placebo 42/91 (46.2%)  Duration of NICU stay, mean (SD) 19.5±5.8 days | Not PO Neonatal outcomes not  defined  NB critical comment to article by Katsanevakis, Mol and Thornton concerning recruitment and results  NB High rates of cerclage in both groups | - | ? | - |
| Azargoon, 2016 Iran | Singletons | Previous PTB (<37 w),  uterine malformations | I: 51  C: 52 | 400 mg vaginal progesterone supp /d 13/50 (26 %)  RR 2.08 (95% CI 1.22-3.54) p=0.004  Duration of NICU stay, mean (SD) 11.6±5.8 days  p=0.53 | Placebo 27/50 (54 %)  Duration of NICU stay, mean (SD)  10.4±6.1 days | Not PO | - | ? | - |

Prevention of preterm birth

STable 4.1.19. cont. Intervention progesterone

Outcome variable: Admittance to neonatal intensive care unit (NICU)

* + No or minor problems

? Some problems

- Major problems

| **Author, year Country**  **Trial acronym** | **Singletons/ Twins/ Triplets** | **Risk factor** | **Number of**  **randomizedpatients**  **n=** | **Results** | | **Comments** | **Directness *** | **Study limitations *** | **Precision *** |
| --- | --- | --- | --- | --- | --- | --- | --- | --- | --- |
|  |  |  |  | **Intervention** | **Control** |  |  |  |  |
|  |  |  |  |  |  |  |  |  |  |
| Blackwell, 2020 USA PROLONG | Singletons | Previous singleton sPTB | Randomized2:1  I: 1130  C: 578 | 17-OHPC 250 mg im/w 137/1093 (12.5%)  RR 1.21 95% CI 0.90-1.62)  No p value Duration of NICU stay* Mean (SD) 18.6±20.4 d  n=137  No statistics | Placebo 58/559 (10.4%)  Duration of NICU stay* Mean (SD) 23.3±24.5 d n=58 | Not PO  Patients with missing data assumed not to have the outcome  * of those admitted to NICU | + | ? | ? |
| Glover, 2011 USA | Singletons | Previous singleton sPTB (<37 w) | I: 20 C:16 | 400 mg oral micronized progesterone/d Duration of NICU stay  Mean (SD) 6.5±10.5 d n=19  p=0.12 | Placebo  Duration of NICU stay Mean (SD) 7.5±9.0 d n=14 | Not PO | + | ? | - |
| Grobman, 2012 USA  SCAN | Singletons | Nulliparous with short TVS CL  <30 mm | I: 327  C: 330 | 17-OHPC 250 mg im/w 63/322 (19.6%)  RR 0.93 (95% CI 0.69–1.27) | Placebo (castor oil) 69/329 (21.0%) | Not PO  *Denominator unclear | ? | + | + |
|  |  |  |  | No p value |  |  |  |  |  |
|  |  |  |  | Duration of NICU stay | Duration of NICU stay |  |  |  |  |
|  |  |  |  | Median (IQR) | Median (IQR) |  |  |  |  |
|  |  |  |  | 17 (6.0-43.0) d | 15.5 (6.0-57.5) d |  |  |  |  |
|  |  |  |  | n=327?* | n=330?* |  |  |  |  |
|  |  |  |  | p=0.61 |  |  |  |  |  |
| Ibrahim, 2010 Egypt | Singletons | Previous PTB | I: 25  C: 25 | 17-OHPC 250 mg/w  3/25 (12%) | Placebo (saline) 9/25 (36%) | Not PO | - | - | - |
|  |  |  |  | p=0.03 |  |  |  |  |  |
|  |  |  |  | No RR |  |  |  |  |  |
| Jabeen, 2012 Pakistan | Singletons | Previous sPTB | I: 30  C: 30 | 17-OHPC 250 mg/w  7/30 (23.3%) | Placebo (inert oil) 9/30 (30%) | Not PO | ? | - | - |
|  |  |  |  | p=0.559 |  |  |  |  |  |
| Majhi, 2009 India | Singletons | Previous sPTB | I: 50  C: 50 | 100 mg vaginal progesterone/d 0/50 | No placebo 4/50 (8%) |  | ? | ? | ? |
|  |  |  |  | p=0.12 |  |  |  |  |  |

* + No or minor problems

? Some problems

- Major problems

Prevention of preterm birth

STable 4.1.19. cont. Intervention progesterone

Outcome variable: Admittance to neonatal intensive care unit (NICU)

| **Author, year Country**  **Trial acronym** | **Singletons/ Twins/ Triplets** | **Risk factor** | **Number of**  **randomizedpatients**  **n=** | **Results** | | **Comments** | **Directness *** | **Study limitations *** | **Precision *** |
| --- | --- | --- | --- | --- | --- | --- | --- | --- | --- |
|  |  |  |  | **Intervention** | **Control** |  |  |  |  |
|  |  |  |  |  |  |  |  |  |  |
| Norman, 2016 UK  (UK 65  hospitals, Sweden 1 hospital) OPPTIMUM | Singletons | FFN pos group: Any of previous PTB, second trimester loss, cervical surgery  FFN neg group: previous sPTB <34 w or  short TVS CL ≤25 mm | I: 618  C: 610 | 200 mg vaginal progesterone/d Intensive care  Mean (SD) 1.9 (8.1) d n=580  High dependency care Mean (SD) 2.1 (10.4) d n=580  Special care Mean (SD) 2.9 (8.3) d  n=581 | Placebo Intensive care  Mean (SD) 1.8 (7.3) d n=569  High dependency care Mean (SD) 2.2 (8.4) d n=569  Special care Mean (SD) 4.2 (10.6) d  n=570 | Not PO | + | + | + |
| O’Brien 2007  USA (+4 other countries) | Singletons | Previous sPTB | I: 332  C: 327 | 90 mg vaginal progesterone gel 8%/d 54/309 (17.5%)  OR 0.75 (95% CI 0.51-1.11)  No p value Duration of NICU stay Mean (SD)14.2 (16.6) d  n=54  Mean difference −6.2 (−15.2 to 2.8) d | Placebo 65/302 (21.5%)  Duration of NICU stay Mean (SD) 20.5 (30.7) d n=65 | Not PO | ? | ? | ? |
| Price, 2021 Zambia | Singletons | HIV | I: 399 C:401 | 17-OHPC 250 mg/w  29/389 (7%)  RR 1.2 (95% CI 0.7-2.0)  No p value | Placebo 24/390 (6%) | Not PO  21 stillborns excluded | ? | + | ? |
| Rai, 2009  India | Singletons | Previous sPTB | I: 75 C:75 | 200 micronized oral progesterone/d 10/74 (13.5%) p<0.001  No RR or OR | Placebo 38/74 (51.4%) | Not PO | - | ? | ? |
| Van Os, 2015 The Netherlands TRIPLE P | Singleton | Short TVS CL  ≤30 mm,  no previous PTB | I: 41  C: 39 | 200 mg micronized vaginal progesterone/d 3/41 (7%)  RR 0.53 (95% CI 0.12–2.25)  No p value Duration of NICU stay  Median (IQR) 3 (1.5–5.5) d  n=3  Median difference (95% CI  -5.0 (-27–0.15) | Placebo 5/39 (13%)  Duration of NICU stay Median (IQR) 8 (7–31) d n=5 | Not PO | + | ? | - |

* + No or minor problems

? Some problems

- Major problems

Prevention of preterm birth

STable 4.1.19. cont. Intervention progesterone

Outcome variable: Admittance to neonatal intensive care unit (NICU)

| **Author, year Country**  **Trial acronym** | **Singletons/ Twins/ Triplets** | **Risk factor** | **Number of**  **randomizedpatients**  **n=** | **Results** | | **Comments** | **Directness *** | **Study limitations *** | **Precision *** |
| --- | --- | --- | --- | --- | --- | --- | --- | --- | --- |
|  |  |  |  | **Intervention** | **Control** |  |  |  |  |
|  |  |  |  |  |  |  |  |  |  |
| Awwad, 2015 Libanon PROGESTWIN | Twins, unselected | ART 75%  MC 17% | Analyzed I:194  women/388 fetuses C: 94  women/188 fetuses  Randomized  2:1 | 17-OHPC 250 mg im/w Only hospital stay not NICU Mean (SD) 9 (17) days n=376  p=0.15 | Placebo (castor oil) Only hospital stay not NICU  Mean (SD) 13 (17) days n=178 | Not PO | + | + | ? |
| Briery, 2009 USA | Twins, unselected | 1/3 previous PTB | I: 16/32 C: 14/28 | 17-OHPC 250 mg im/w Mean (SD) NICU stay 18.4 (65.8) d  n=32 p=0.155 | Placebo  Mean (SD) NICU stay 17.3 (29.8) d  n=28 | Not PO | ? | ? | - |
| Brizot, 2015 Brazil | Twins DA | MC 25% (I) and 19%  (C) Only naturally conceived,  no history of PTB | I: 195/378 C: 195/382 | 200 mg vaginal natural progesterone/d 88/344 (25.6%)  OR 1.06 (95% CI 0.68-1.67)  No p value | Placebo  89/364 (24.5%) | Not PO | ? | ? | - |
| Norman, 2009 UK  (9 hospitals) STOPPIT | Twins | MC twins I: 46/247 C: 45/247  No MA twins | I: 247/494 C: 247/494 | 90 mg vaginal progesterone /day (Crinone) 167/494 (33.8%)  OR 1.08 (95% CI 0.76-1.54) p=0.65  Mean (SD) NICU stay 7.5 (19.9) d n=494 (all)  Mean difference 1.5 (–1.9 -5.0) p=0.38  Mean (SD) NICU stay 26.9 (33.5)  n=167 (those admitted NICU) Mean difference 3.3 (–5.3-11.9) p=0.45 | Placebo 158/494 (32.0%)  Mean (SD) NICU stay 8.7 (23.1) d  n=494 (all)  Mean (SD) NICU stay 23.6 (29.5) d  n=158 (those admitted  NICU) | Not PO | + | + | ? |
| Rode, 2011 Denmark and Austria  PREDICT | Twins DA | MC  I: 43/334 (12.9%)  C: 57/343 (16.6%) | I: 334/668 C: 343/686 | 200 mg vaginal progesterone/d (pessary) 307/664 (46.2%)  OR 0.8 (95% CI 0.6–1.1)  No p values | Placebo  354/678 (52.2%) | Not PO | + | + | + |

* + No or minor problems

? Some problems

- Major problems

Prevention of preterm birth

STable 4.1.19. cont. Intervention progesterone

Outcome variable: Admittance to neonatal intensive care unit (NICU)

| **Author, year Country**  **Trial acronym** | **Singletons/ Twins/ Triplets** | **Risk factor** | **Number of**  **randomizedpatients**  **n=** | **Results** | | **Comments** | **Directness *** | **Study limitations *** | **Precision *** |
| --- | --- | --- | --- | --- | --- | --- | --- | --- | --- |
|  |  |  |  | **Intervention** | **Control** |  |  |  |  |
|  |  |  |  |  |  |  |  |  |  |
| Serra, 2013 Spain | Twins DCDA | MAR  I1: 92/96 (95.8%)  I2: 94/97 (96.9%)  C: 96/97 (99.0%) | I1: 98/196 I2: 98/196 C: 98/196 | I1: 200 mg vaginal progesterone (pessary) /d I2: 400 mg vaginal progesterone (pessary)/d I1: 21/194 (10.8%)  I2: 19/191 (9.9%)  I1+I2: 40/385 (10.4%)  Comparison between groups: NS | Placebo  28/190 (14.7%) | Not PO | ? | ? | - |
| Combs, 2010 USA | Triplets | Trichorionic triamniotic triplets MAR  I: 90%  C: 84% | I: 56/155  liveborn neonates C: 25  women/75 liveborn neonates | 17-OHPC 250 mg im/w Mean (SD)  NICU 16.0 (23.2) d  Intermediate stay 9.3 (12.5) d No statistics | Placebo Mean (SD)  NICU 18.8 (30.1) d  Intermediate stay 17.3  (17.8) d | Not PO Denominator unclear if pregnancies or neonates | + | ? | ? |
| Lim, 2011  The Netherlands AMPHIA | Multifetal pregnancies | Triplets/+ I: 9 (3%)  C: 9 (3%)  (incl.one quadruplet) MC  I: 57 (17%)  C: 57 (17%)  Fertility treatment I: 140 (42%)  C: 120 (36%) | I: 336 /681  (327 sets of twins, 9 sets of triplets)  C: 335 women/  680 (326 sets of twins, 8 sets of triplets, one quadruplet) | 17-OHPC 250 mg im/w 153/681 (22%)  RR 1.29 (95% CI 0.97–1.72)  No p value | Placebo 116/674 (16%) | Not PO | + | + | ? |
| Aboulghar 2012  Egypt | Mixed Singletons 215/306 (70.3%)  DC twins 91/306 (29.7%) | ART  pregnancies | RandomizedI:161 C:152  Analyzed I:161 (112  singletons, 49 sets of twins)  C:145 (103  singletons, 42 sets of twins) | 400 mg vaginal natural progesterone/d Singletons  13/112 (11.6%)  OR 1.8 (95% CI 0.68-4.7)  No p value Twin pregnancies*  6/49 (12.2%)  OR 0.39 (95% CI 0.1-1.1)  No p value | Placebo  Singletons 7/103 (6.8%)  Twin pregnancies* 11/42 (26.2%) | Not PO  *Only reported on pregnancy level | ? | ? | - |

* + No or minor problems

? Some problems

- Major problems

Prevention of preterm birth

STable 4.1.19. cont. Intervention progesterone

Outcome variable: Admittance to neonatal intensive care unit (NICU)

| **Author, year Country**  **Trial acronym** | **Singletons/ Twins/ Triplets** | **Risk factor** | **Number of**  **randomizedpatients**  **n=** | **Results** | | **Comments** | **Directness *** | **Study limitations *** | **Precision *** |
| --- | --- | --- | --- | --- | --- | --- | --- | --- | --- |
|  |  |  |  | **Intervention** | **Control** |  |  |  |  |
|  |  |  |  |  |  |  |  |  |  |
| Cetingoz, 2011 Turkey | Mixed Singletons I: 41/80 (51.3%) C: 42/70 (60%)  Twins I: 39/80 (48.7%)  C: 28/70 (40.0) | Twin pregnancy, previous sPTD, uterine malformation | I: 84  C: 76  Analyzed  I: 80/119 C: 70/98 | 100 mg vaginal progesterone/d Singletons and twins  13/80* (16.3%)  C vs I OR: 3.04 (95% CI 1.41-6.54) p=0.004 | Placebo Singletons and twins  26/80* (37.1%) | Not PO  *Number of births with at least one neonate admitted to NICU | ? | ? | - |
| Crowther, 2017 Australia PROGRESS | Mixed Singletons n=775 (98.5%  Twins n=12 (1.5%) | Previous sPTD  <37 w | I: 398 (390  singletons and 8 twin pregnancies) 406 infants  C: 389  (385 singletons  and 4 twin  pregnancies) 393 infants | 100 mg vaginal progesterone pessary/d 68/402 (16.9%)  RR 0.92 (95% CI 0.68-1.27) p=0.624  aRR 0.92 (95% CI 0.67-1.25) p=0.591  aRR adjusted for GA at randomization, GA of previous PTB, and reason for PTB | Placebo 71/388 (18.3%) | Not PO | + | + | ? |
| Fonseca, 2007 Brazil (multicenter inter-  national, UK [5 centers], Chile, Brazil,  Greece) | Mixed Singletons (226, 90%)  Twins (24,  10%, all DA) | Short TVS CL ≤15 mm | I: 125  women/136 infants (114  singletons, 22 twins)  C: 125  women/138 infants (112  singletons, 26 twins) | 200 mg vaginal progesterone /day (Utrogestan)  33/136 (24.3%)  RR 0.80 (95% CI 0.49-1.21) p=0.30  aRR 0.80 (95% CI 0.47-1.24) p=0.34  aRR adjusted for maternal age, BMI, smoking status, race, history of PTB, and CL  at the time of randomization. | Placebo 42/138 (30.4%) | Not PO | ? | ? | + |

Prevention of preterm birth

STable 4.1.19. cont. Intervention progesterone

Outcome variable: Admittance to neonatal intensive care unit (NICU)

* + No or minor problems

? Some problems

- Major problems

| **Author, year Country**  **Trial acronym** | **Singletons/ Twins/ Triplets** | **Risk factor** | **Number of**  **randomizedpatients**  **n=** | **Results** | | **Comments** | **Directness *** | **Study limitations *** | **Precision *** |
| --- | --- | --- | --- | --- | --- | --- | --- | --- | --- |
|  |  |  |  | **Intervention** | **Control** |  |  |  |  |
|  | | | | | | | | | |
| **Other interventions in comparison with progesterone** | | | | | | | | | |
| Cruz-Melguizo, 2018 | Singletons | Short TVS CL ≤25 mm (women with cervical | I: 125  C: 118 | Pessary 14/125 (12%) | 200 mg vaginal progesterone/d | Not PO | + | ? | ? |
| Spain |  | surgery and ≥3 |  | No RR | 14/118 (12%) |  |  |  |  |
| (27 centers) |  | previous PTBs were |  | p=0.90 |  |  |  |  |  |
|  |  | excluded) |  |  |  |  |  |  |  |
| Dang 2019 | Twins | Short TVS CL ≤38 mm (women with history | I:150 C: 150 | Arabin pessary | 400 mg vaginal progesterone mg/d | Not PO | ? | ? | ? |
| Vietnam |  | of cervical surgery |  | 39/296 (13%) |  |  |  |  |  |
| (single center) |  | excluded) |  | RR 0.59 (95% CI 0.35-0.82) | 66/298 (22%) |  |  |  |  |
|  |  |  |  | p=0.01 |  |  |  |  |  |
|  |  |  |  | Subgroup analysis TVS CL |  |  |  |  |  |
|  |  |  |  | ≤28 mm | Subgroup analysis TVS CL |  |  |  |  |
|  |  |  |  | n=94 | ≤28 mm |  |  |  |  |
|  |  |  |  | 14/94 (15%) | n=70 |  |  |  |  |
|  |  |  |  | RR 0.37 (95% CI 0.12-0.55) | 28/70 (40%) |  |  |  |  |
|  |  |  |  | p<0.001 |  |  |  |  |  |

17-OHPC;17-α-hydroxyprogesterone caproate, aRR; adjusted risk ratio, ART; assisted reproductive technology, BMI; body mass index, C; control, CL; cervical length, DA; diamniotic, DC; dichorionic, DCDA; dichorionic, diamniotic GA; gestational age, I; intervention, IUFD; intrauterine fetal death, MAR; medically assisted reproduction; MC; monochorionic, MCDA; monochorionic diamniotic, NICU; neonatal intensive care unit, NNM; neonatal mortality, OR; odds ratio, PO; primary outcome, PTB; preterm birth, RR; risk ratio, SD; standard deviation, sPTB: spontaneous preterm birth, TVS; transvaginal sca

Prevention of preterm birth

STable 4.1.20. Intervention progesterone

Outcome variable: Long term child outcome

* + No or minor problems

? Some problems

- Major problems

| **Author, year Country**  **Trial acronym** | **Singletons/ Twins/ Triplets** | **Risk factor** | | **Number of**  **randomizedpatients**  **n=** | **Results** | | **Comments/definitions** | **Directness *** | **Study limitations *** | **Precision *** |
| --- | --- | --- | --- | --- | --- | --- | --- | --- | --- | --- |
|  |  |  |  |  | **Intervention** | **Control** |  |  |  |  |
|  |  | |  |  |  |  |  |  |  |  |
| Cuijpers, 2021 The Netherlands TRIPLE P follow up (Van Os 2015) | Singletons | | Short TVS CL  ≤30 mm  at 18-22 w | RandomizedI: 41  C: 39  Follow up at 2 years  I: 29  C: 30 | 200 mg micronized progesterone/d (Utrogestan pessary)  Bayley-III cognitive score Mean (SD) 101.6 (9.7) n=28  Mean difference  -3.4 (95% CI -9.3-2.6) p=0.29  Bayley-III motor score Mean (SD) 102.4 (10.9) n=28  Mean difference  -4.9 (95% CI -11.2-1.4)  p=0.13  Abnormal ASQ  5/27 (19%)  RR (95% CI 0.33 to 3.06)  p=1.00  Abnormal CBCL  1/27 (4%)  RR 0.22 (95% CI 0.02 to 2.12) p=0.35  Death or abnormal developmental outcome  Complete case analysis  6/41 (15%)  RR 0.78 (95% CI 0.24-2.58) p=0.69  With multiple imputed data  14.4/41 (35%)  RR 1.00 (95% CI 0.50-1.98) p=0.99 | Placebo pessary  Bayley-III cognitive score Mean (SD) 105.0 (12.5) n=29  Bayley-III motor score Mean (SD) 107.3 (12.6) n=29  Abnormal ASQ  5/27 (19%)  Abnormal CBCL  4/27 (15%)  Death or abnormal developmental outcome Complete case analysis 7/39 (18%)  With multiple imputed data  13.7/39 (35%) | Not PO  2 year corrected year infant follow up  No difference in physical (including genital and neurological examination) behavioral and health related outcomes  Median (IQR) age at follow up at 25 (23-27) months of age in both groups  Abnormal ASQ score: 1 SD below normative mean on two or more domains, or as score of 2 SD below normative mean on at least one domain  Abnormal CBCL: score in the clinical range (>  97th percentile)  Complete case analysis or with multiple imputed data includes all randomized infants | + | ? | - |

| **Author, year Country**  **Trial acronym** | **Singletons/ Twins/ Triplets** | **Risk factor** | | **Number of**  **randomizedpatients**  **n=** | **Results** | | **Comments/definitions** | **Directness *** | **Study limitations *** | **Precision *** |
| --- | --- | --- | --- | --- | --- | --- | --- | --- | --- | --- |
|  |  |  |  |  | **Intervention** | **Control** |  |  |  |  |
|  |  | |  |  |  |  |  |  |  |  |
| Norman, 2016 UK  (UK 65 hospitals,  Sweden 1 hospital) OPPTIMUM | Singletons | | FFN pos group: Any of previous PTB, second trimester loss, cervical surgery  FFN neg group: previous sPTB <34 w or short TVS CL ≤25 mm | RandomizedI: 618  C: 610 | 200 mg vaginal progesterone/d Cognitive composite score at 2 years*  Mean (SD) 97.3 (17.9) n=430  Median (IQR) weeks at assessment 110.4 (104.0-121.5)  Mean difference -0.48  (95% CI -2.77-1.81) p=0.68  Adj. mean difference -0.48  (95% CI -2.77-1.81) p=0.68 | Placebo  Cognitive composite score at 2 years*  Mean (SD) 97.7 (17.5) n=439  Median (IQR) weeks at assessment  111.6 (104.6-122.2) | PO (one of three)  *Bayley-III cognitive composite score at 22-26 months of chronological age.  Scores were imputed for deaths | + | + | + |
| Northen, 2007  USA  (follow-up of  Meis 2003) | Singletons | | Previous PTB | 463 women in original study, I: 310  C: 153  348 children were potentially eligible for follow-up  I: 194  C: 84 | 17-OHPC 250 mg im weekly ASQ  Scored below cut off on  at least one area at mean age 48 months  53/193 (27.5%) p=0.92  NS overall or in individual domains  Preschool Activities Inventory Mean score boys 66.5  p=0.3  Mean score girls 32  p=0.5  NS difference in growth or physical abnormalities, including genital malformations | Placebo ASQ  Scored below cut off on at least one area at mean age  48 months  23/82 (28%)  Preschool Activities Inventory  Mean score boys 67.3 Mean score girls 33 | Not PO | ? | ? | ? |

* + No or minor problems

? Some problems

- Major problems

Prevention of preterm birth

STable 4.1.20. cont. Intervention progesterone

Outcome variable: Long term child outcome

Prevention of preterm birth

STable 4.1.20. cont. Intervention progesterone

Outcome variable: Long term child outcome

| **Author, year Country**  **Trial acronym** | **Singletons/ Twins/ Triplets** | **Risk factor** | | **Number of**  **randomizedpatients**  **n=** | **Results** | | **Comments/definitions** | **Directness *** | **Study limitations *** | **Precision *** |
| --- | --- | --- | --- | --- | --- | --- | --- | --- | --- | --- |
|  |  |  |  |  | **Intervention** | **Control** |  |  |  |  |
|  |  | |  |  |  |  |  |  |  |  |
| Norman, 2018 UK  (UK 65 hospitals,  Sweden 1 hospital) OPPTIMUM  HTA-report | Singletons | | FFN pos group: Any of previous PTB, second trimester loss, cervical surgery  FFN neg group:  previous sPTB <34 w or short TVS CL ≤25 mm | RandomizedI: 618  C: 610 | 200 mg vaginal progesterone/d Cognitive composite score at 2 years*  Mean (SD) 99.7 (14.7)  n=410  Mean difference -0.04  (95% CI -0.26-0.19) | Placebo  Cognitive composite score at 2 years*  Mean (SD) 99.5 (14.7) n=423 | PO (one of three)  *Bayley-III cognitive composite score at 22-26 months of chronological age.  Denominator is children alive at 2 years | + | + | + |
| Simons, 2021  Systematic review | 7 RCTs  (based on 5 RCTs) with singletons (n=3) and multifetal pregnan-cies (n=4) | | Children born to women who received progesterone treatment for any indication during pregnancy | 4222 children  aged 6 m to 8 years | Singletons exposed to progesterone  Mean (SD) composite Bailey score at 2 years  99.7 (14.7) n=438  (2 RCTs)  Mean standardized difference  -0.04 (95% CI -0.26 to 0.19) | Singletons exposed to placebo  Mean (SD) composite Bailey score at 2 years  99.5 (14.7)  n=452 (2 RCTs) | MA based two RCTs, unpublished data from one RCT, Cuijpers et al., 2020 and published data from one RCT, Norman et al., 2018 | + | ? | + |
| McNamara, 2015 UK  STOPPIT follow  up (Norman 2009) | Twins | | MC twins I: 46/247 C: 45/247  No MA twins | I: 247/494 C: 247494 | 90 mg vaginal progesterone /day  (Crinone) Delayed development  CDI score (≥30% below age range) 42/140 (30%)  0.87 (95% CI 0.46–1.63)  p=0.66  Borderline/delayed development CDI score (≥25% below age range) 60/140 (43%)  OR 0.67 (95% CI 0.35–1.28) p=0.23 | Placebo  Delayed development  CDI score (≥30% below age  range)  65/184 (35%)  Borderline/delayed development  CDI score (≥25% below age  range)  104/184 (57%) | Not PO  No evidence of difference in general health status assessed with Health Utilities Index | + | + | ? |

* + No or minor problems

? Some problems

- Major problems

Cont.

| Prevention of preterm birth  STable 4.1.20. cont. Intervention progesterone  Outcome variable: Long term child outcome | | | | | | | | | |
| --- | --- | --- | --- | --- | --- | --- | --- | --- | --- |
| **Author, year Country**  **Trial acronym** | **Singletons/ Twins/ Triplets** | **Risk factor** | **Number of**  **randomizedpatients**  **n=** | **Results** | | **Comments/definitions** | **Directness *** | **Study limitations *** | **Precision *** |
|  |  |  |  | **Intervention** | **Control** |  |  |  |  |

| Rode, 2011 Denmark and Austria PREDIT | Twins DA | MC  I: 43/334 (12.9%)  C: 57/343 (16.6%) | I: 334/668 C: 343/686 | 200 mg micronized vaginal progesterone (Utrogestan pessary) Mean (SD) ASQ score at 6 m  215 (37.5)  n=517* (78.8%)  p=0.45  Mean (SD) ASQ score at 18 m  193 (42.6)  n=505* (76.8%) p=0.89  ASQ <115 3.8% | Placebo pessary  Mean (SD) ASQ score at 6 m  218 (36.7)  n=533* (79.7%)  Mean (SD) ASQ score at 18 m  194 (40.6)  n=486* (72.8%)  ASQ <115 3.7% | Not PO  *Numbers calculated, only % in  article | + | + | + |
| --- | --- | --- | --- | --- | --- | --- | --- | --- | --- |
| Vedel, 2016 Denmark PREDICT follow up, Rode 2011 | Twins DA | Fertility treatment I: 50.0%  C: 47.2% | RandomizedI: 114  C: 106  women returned ASQ for at least one child  I: 492 children  C: 497 children (register data) I: 225 children  C: 212 children (ASQ data) | 200 mg micronized vaginal progesterone (Utrogestan pessary) Total ASQ score  Mean (SD) 269 (28.2)  n=225  p=0.03  Gross motor skill 54.6 (7.6) p=0.03  NS difference for the other 4 domains  Total score <10^th^ percentile 14/225 (6.2%)  OR 0.47 (95% CI 0.21-1.06)  No difference for the 5 different domains <10^th^ centile  Age at completion, mean (SD)  56.9 (6.0) months p=0.76 | Placebo pessary  Total ASQ score  Mean (SD) 261.7 (31.4)  n=212  Gross motor skill 52.5 (9.6)  Total score <10^th^ percentile  26/212 (12.3%)  Age at completion, mean (SD)  57.1 (6.1) months | No difference in hospital admissions, length of hospital stay and no overall difference in the rate of different diagnoses. ASQ (5 domains) assessed at 4 or 5 years of age (max score total 300 points, each domain [gross motor, fine motor, communication, problem solving, personal /social skills] max 60 points) | + | ? | ? |

17-OHPC; 17-α-hydroxyprogesterone caproate, aRR; adjusted risk ratio, ASQ; Age and Stages Questionnaire, C; control, CBCL; child behavior checklist, CDI; child development inventory score categorization, CL; cervical length, DA; diamniotic, HTA; Health Technology Assessment, I; intervention, IQR; interquartile range, m; months, MC; monochorionic, NS; not significant, OR; odds ratio, PTB; preterm birth, RCT; randomized controlled trial, RR; risk ratio, ROP; retinopathy of prematurity, SD; standard deviation, sPTB, spontaneous preterm birth, TVS; transvaginal scan

Prevention of preterm birth

* + No or minor problems

? Some problems

- Major problems

STable 4.1.21. Intervention progesterone

Outcome variable: Maternal mortality

| **Author, year Country**  **Trial acronym** | **Singletons/ Twins/ Triplets** | **Risk factor** | **Number**  **of randomized patients**  **n=** | **Results** | | **Comments** | **Directness *** | **Study limitations *** | **Precision *** |
| --- | --- | --- | --- | --- | --- | --- | --- | --- | --- |
|  |  |  |  | **Intervention Progesterone** | **Control Placebo** |  |  |  |  |

| Price, 2021 Zambia | Singletons | HIV | I: 399  C: 401 | 17-OHPC 250 mg im/w 0/399 (0%)  No statistics | Placebo 1/401 (<1%) | Not PO | ? | + | ? |
| --- | --- | --- | --- | --- | --- | --- | --- | --- | --- |
| **Other interventions in comparison with progesterone** | | | | | | | | | |
| Dang 2019  Vietnam (single center) | Twins | Short TVS CL ≤38 mm (women with history of cervical surgery excluded) | I:150 C: 150 | Arabin pessary  0/148 (0%)  No statistics | 400 mg vaginal progesterone /d 0/149 (0%) | Not PO | ? | ? | ? |

17-OHPC;17-α-hydroxyprogesterone caproate, C; control, CL; cervical length, HIV; human immunodeficiency virus, im; intramuscular, I; intervention, PO; primary outcome, TVS; transvaginal scan, w; week

Prevention of preterm birth

STable 4.1.22. Intervention progesterone

Outcome variable: Maternal morbidity, hypertensive disorders in pregnancy, (gestational hypertension, preeclampsia, eclampsia)

* + No or minor problems

? Some problems

- Major problems

| **Author, year Country**  **Trial acronym** | **Singletons/ Twins/ Triplets** | **Risk factor** | **Number**  **of randomized**  **patients**  **n=** | **Results** | | **Comments** | **Directness *** | **Study limitations *** | **Precision *** |
| --- | --- | --- | --- | --- | --- | --- | --- | --- | --- |
|  |  |  |  | **Intervention Progesterone** | **Control Placebo** |  |  |  |  |
|  |  |  |  |  |  |  |  |  |  |
| Aflatoonian, 2013  Iran | Singletons | ART pregnancies | I: 52  C: 47 | 17-OHPC 250 mg im/w 8/52 (15.4%)  No statistics | Placebo 1/47 (2.1%) | Not PO  No definition of hypertension | ? | - | - |
| Ashoush, 2017 Egypt | Singletons | Previous sPTB (<37 w) | I: 106  C: 106 | 400 mg oral progesterone/d GH 3/96 (3.1%)  p=0.66 | Placebo  GH 2/91 (2.2%) | Not PO  OBS critical comment to article by Katsanevakis, Mol and Thornton concerning recruitment and results  NB: High cerclage rates in both groups | - | ? | - |
| Blackwell, 2020 USA PROLONG | Singletons | Previous singleton sPTB | I: 1130  C: 578  Randomized 2:1 | 17-OHPC 250 mg im/w PE 47/1130 (4.2%)  RR 0.86 (95% CI 0.51-1.46)  No p value | Placebo  PE 30/578 (5.2%) | Not PO | + | ? | ? |
| Hauth, 1983 USA | Singletons | Women from an active duty  military population | I: 80  C: 88 | 17-OHPC 1000mg im/w PIH 10/80 (12.5%)  NS  No p value, OR or RR | Placebo (castor oil) PIH 12/88 (13.6%) | Not PO | - | - | - |
| Norman, 2016 UK  (UK 65  hospitals, Sweden 1  hospital) OPPTIMUM | Singletons | FFN pos group: Any of previous PTB, second trimester loss, cervical surgery  FFN neg group: previous sPTB <34 w or  short TVS CL ≤25 mm | I: 618  C: 610 | 200 mg progesterone/day  GH 23/593 (4%)  PE 10/593 (2%)  Eclampsia 0/593 No statistics | Placebo  GH 24/590 (4%)  PE 11/590 (2%)  Eclampsia 1/590 (<1%) | Not PO | + | + | + |
| Price, 2021 Zambia | Singletons | HIV | I: 399 C:401 | 17-OHPC 250 mg/w  PIH 13/399 (3%)  RR 2.2 (95% CI 0.8-5.7)  PE 6/399 (2%)  RR 0.7 (95% CI 0.2-1.9)  Eclampsia 0/399 (0%) No RR. No p values | Placebo PIH 6/401 (2%)  PE 9/401 (2%)  Eclampsia 1/401 (< 1%) | Not PO | ? | + | ? |

Prevention of preterm birth

STable 4.1.22. cont. Intervention progesterone

Outcome variable: Maternal morbidity, hypertensive disorders in pregnancy, (gestational hypertension, preeclampsia, eclampsia)

* + No or minor problems

? Some problems

- Major problems

| **Author, year Country**  **Trial acronym** | **Singletons/ Twins/ Triplets** | **Risk factor** | **Number**  **of**  **randomized**  **patients**  **n=** | **Results** | | **Comments** | **Directness *** | **Study limitations *** | **Precision *** |
| --- | --- | --- | --- | --- | --- | --- | --- | --- | --- |
|  |  |  |  | **Intervention Progesterone** | **Control Placebo** |  |  |  |  |
|  |  |  |  |  |  |  |  |  |  |
| Awwad, 2015 Libanon PROGESTWIN | Twins, unselected | ART 75%  MC 17% | I:197 C: 96  Randomized 2:1 | 17-OHPC 250 mg im/w HDP 16/194 (8.2%)  OR 1.0 (95% CI 0.4–2.8) p=0.51 | Placebo (castor oil) 7/94 (7.4%) | Not PO | + | + | ? |
| Brizot, 2015 Brazil | Twins DA | MC 25% (I) and 19% (C)  Only naturally conceived, no history of PTB | I: 195  C: 195 | 200 mg vaginal natural progesterone/d  PE 24/188 (12.8%)  OR 0.85 (95% CI 0.45-1.60)  No p value | Placebo  PE 28/190 (14.7%) | Not PO | ? | ? | - |
| Combs, 2011 USA | Twins DCDA | 20% fetal reduction ART  I: 66% C:58%  Prior PTB: I: 12%  C: 13% | I: 160  C: 80  Randomized 2:1 | 17-OHPC 250 mg  (in 1 mL castor oil) im/w GH/PE 40/160 (25%)  OR 1.40 (0.72-2.73) p=0.41 | Placebo (1 mL castor oil) GH/PE 15/78 (19%) | Not PO | + | ? | ? |
| Rehal, 2021  UK (+5 other European countries) | Twins | MC I: 23%  C: 23% ART  I: 34%  C: 35% | I: 582  C: 587 | 600 mg vaginal progesterone/d PE 3/596  Eclampsia 0/596 No statistics | Placebo PE 0/598  Eclampsia 1/598 | Not PO  PE >5 d hospitalization Eclampsia >10 d hospitalization | + | + | ? |
| Rode, 2011 Denmark and Austria PREDICT | Twins (DA) | MC  I: 43/334 (12.9%)  C: 57/343 (16.6%) | I: 334  C: 343 | 200 mg vaginal progesterone/d (pessary)  PE 27/332 (8.1%)  OR 0.9 (95% CI 0.5-1.5)  No p values | Placebo  PE 30/341 (8.8%) | Not PO | + | + | + |
| Rouse, 2007 USA SSTARS | Twins (DA) | MC  I: 59/327 (18%)  C: 57/334 (17.1%) | I: 327  C: 334 | 17-OHPC 250 mg im/w HDP 66/325 (20.3%)  RR 1.2 (95% CI 0.9-1.7)  No p values | Placebo  HDP 55/330 (16.7%) | Not PO | + | ? | + |

.

Prevention of preterm birth

STable 4.1.22. cont. Intervention progesterone

Outcome variable: Maternal morbidity, hypertensive disorders in pregnancy, (gestational hypertension, preeclampsia, eclampsia)

* + No or minor problems

? Some problems

- Major problems

| **Author, year Country**  **Trial acronym** | **Singletons/ Twins/ Triplets** | **Risk factor** | **Number**  **of randomized patients**  **n=** | **Results** | | **Comments** | **Directness *** | **Study limitations *** | **Precision *** |
| --- | --- | --- | --- | --- | --- | --- | --- | --- | --- |
|  |  |  |  | **Intervention Progesterone** | **Control Placebo** |  |  |  |  |
|  |  |  |  |  |  |  |  |  |  |
| Serra, 2013 Spain | Twins (DCDA) | MAR  I1: 92/96 (95.8%)  I2: 94/97 (96.9%)  C: 96/97 (99.0%) | I1: 98 I2:98 C: 98 | I1: 200 mg vaginal progesterone (pessary) /d  HDP 5/97 (5.2%)  I2: 400 mg vaginal progesterone (pessary)/d  HDP 6/97 (6.2%) HDP I1 + I2: 11/194 (5.7%)  I1, I2, C= NS I1+I2 vs C= NS I1 vs I2=NS | Placebo HDP 3/96 (3.1%) | Not PO | ? | ? | - |
| Lim, 2011  The Netherlands AMPHIA | Multifetal pregnancies | Triplets/+ I: 9 (3%)  C: 9 (3%)  (incl.one quadruplet) MC  I: 57 (17%)  C: 57 (17%)  Fertility treatment I: 140 (42%)  C: 120 (36%) | I: 336 women  C: 335 women | 17-OHPC 250 mg im/w HDP 54/336 (16%)  RR 0.93 (95% CI 0.67-1.30)  No p value | Placebo  HDP 57/335 (17%) | Not PO | + | + | ? |
| Crowther, 2017 Australia PROGRESS | Mixed Singletons n=775 (98.5%  Twins n=12 (1.5%) | Previous sPTD  <37 w | I: 398 (390  singletons and 8 twin pregnancies) 406 infants  C: 389  (385 singletons and 4 twin pregnancies) 393 infants | 100 mg vaginal progesterone pessary/d  PE 12/398 (3.0%)  RR 1.47 (95% CI 0.61-3.55) p=0.396 | Placebo  PE 8/389 (2.1%) | Not PO | + | + | ? |
| Caritis, 2009 USA SSTARS | Triplets | 30% DC or unknown chorionicity  ART: 70% | I: 71  C: 63 | 17-OHPC 250 mg im/w GH/PE 15/71 (21%)  RR 0.7 (95% CI 0.4-1.3)  No p value | Placebo GH/PE 18/63 (29%) | Not PO | ? | ? | ? |

Prevention of preterm birth

STable 4.1.22. cont. Intervention progesterone

Outcome variable: Maternal morbidity, hypertensive disorders in pregnancy, (gestational hypertension, preeclampsia, eclampsia)

* + No or minor problems

? Some problems

- Major problems

| **Author, year Country**  **Trial acronym** | **Singletons/ Twins/ Triplets** | **Risk factor** | **Number**  **of randomized patients**  **n=** | **Results** | | **Comments** | **Directness *** | **Study limitations *** | **Precision *** |
| --- | --- | --- | --- | --- | --- | --- | --- | --- | --- |
|  |  |  |  | **Intervention Progesterone** | **Control Placebo** |  |  |  |  |
|  |  |  |  |  |  |  |  |  |  |
| Combs, 2010 | Triplets | Trichorionic triamniotic triplets MAR  I: 90%  C: 84% | I: 56  C: 25 | 17-OHPC 250 mg im/w GH/PE 8/56 (14%)  RR 0.43 (95% CI 0.12-1.62) p=0.21 | Placebo GH/PE 7/25 (28%) | Not PO | + | ? | ? |
| **Other interventions in comparison with progesterone** | | | | | | | | | |
| Dang 2019 | Twins | Short TVS CL ≤38  mm | I:150 C: 150 | Arabin pessary HDP | 400 mg vaginal progesterone/d | Not PO | ? | ? | ? |
| Vietnam |  | (women with history of |  | 16/148 (10.8%) | HDP |  |  |  |  |
| (single center) |  | cervical surgery excluded) |  | RR 0.81 (95% CI 0.43-1.49) | 20/149 (13.4%) |  |  |  |  |
|  |  |  |  | p=0.59 |  |  |  |  |  |
|  |  |  |  | Eclampsia/HELLP |  |  |  |  |  |
|  |  |  |  | 1/148 (0.7%) | Eclampsia/HELLP |  |  |  |  |
|  |  |  |  | RR 0.34 (95% CI 0.04-3.19) | 3/148 (2%) |  |  |  |  |
|  |  |  |  | p=0.62 |  |  |  |  |  |

17-OHPC;17-α-hydroxyprogesterone caproate, aRR; adjusted relative risk, ART; assisted reproductive technology, C; control, CL; cervical length, DA; diamniotic, DC; dichorionic, DCDA; dichorionic diamniotic, GA; gestational age, GH; gestational hypertension, HDP; hypertensive disorder of pregnancy, HELLP; hemolysis elevated liver enzymes low platelets, I; intervention, IUFD; intrauterine fetal death, MAR; medically assisted reproduction; MC; monochorionic, MCDA; monochorionic diamniotic, NA; not applicable, OR; odds ratio, PE; preeclampsia, PIH; pregnancy induced hypertension, PTB; preterm birth, RR; risk ratio, sPTB: spontaneous preterm birth, TVS; transvaginal scan

* + No or minor problems

? Some problems

- Major problems

Prevention of preterm birth

STable 4.1.23. Intervention progesterone

Outcome variable: Maternal morbidity, gestational diabetes mellitus (GDM)

| **Author, year Country**  **Trial acronym** | **Singletons/ Twins/ Triplets** | **Risk factor** | | **Number of**  **randomizedpatients**  **n=** | **Results** | | **Comments** | **Directness *** | **Study limitations *** | **Precision *** |
| --- | --- | --- | --- | --- | --- | --- | --- | --- | --- | --- |
|  |  |  |  |  | **Intervention Progesterone** | **Control Placebo** |  |  |  |  |
|  |  | |  |  |  |  |  |  |  |  |
| Aflatoonian, 2013 Iran | Singletons | | ART pregnancies | I: 52  C: 47 | 17-OHPC 250 mg im/w 11/52 (21.2%)  No statistics | Placebo 6/47 (12.8%) | Not PO | ? | - | - |
| Ashoush, 2017 Egypt | Singletons | | Previous sPTB (<37 w) | I: 106  C: 106 | 400 mg oral progesterone/d 1/96 (1.0%)  p=0.51 | Placebo 2/91 (2.2%) | Not PO  OBS critical comment to article by Katsanevakis, Mol and Thornton concerning recruitment and results  NB High rates of cerclage in both groups | - | ? | - |
| Blackwell, 2020 USA PROLONG | Singletons | | Previous singleton sPTB | I: 1130  C: 578  Randomized  2:1 | 17-OHPC 250 mg im/w 35/1130 (3.1%)  RR 0.91 (95% CI 0.54-1.54)  No p value | Placebo 21/578 (3.6%) | Not PO | + | ? | ? |
| Norman, 2016 UK  (UK 65 hospitals,  Sweden 1 hospital) OPPTIMUM | Singletons | | FFN pos group: Any of previous PTB, second trimester loss, cervical surgery  FFN neg group: previous sPTB <34 w or short TVS CL ≤25 mm | I: 618  C: 610 | 200 mg progesterone/day  27/593 (5%)  No statistics | Placebo 37/590 (6%) | Not PO | + | + | + |
| Awwad, 2015 Libanon PROGESTWIN | Twins, unselected | | ART 75%  MC 17% | I:197 C: 96  Randomized  2:1 | 17-OHPC 250 mg im/w 13/194 (6.7%)  OR 1.0 (95% CI 0.3-2.3) p=0.51 | Placebo (castor oil) 7/94 (7.4%) | Not PO | + | + | ? |
| Brizot, 2015 Brazil | Twins DA | | MC 25% (I) and 19% (C)  Only naturally conceived, no history of PTB | I: 195  C: 195 | 200 mg vaginal natural progesterone/d 12/188 (6.4%)  OR 0.79 (95% CI 0.33-1.88)  No p value | Placebo 15/190 (7.9%) | Not PO | ? | ? | - |
| Combs, 2011 USA | Twins DCDA | | 20% fetal reduction  ART, I: 66% C:58%  Prior PTB, I: 12% C: 13% | I: 160  C: 80  Randomized 2:1 | 17-OHPC 250 mg  (in 1 mL castor oil) im/w 21/160 (13%)  OR 1.30 (95% CI 0.55-3.09) p=0.67 | Placebo (1 mL castor oil)  8/78 (10%) | Not PO | + | ? | ? |

* + No or minor problems

? Some problems

- Major problems

Prevention of preterm birth

STable 4.1.23. cont. Intervention progesterone

Outcome variable: Maternal morbidity, gestational diabetes mellitus (GDM)

| **Author, year Country**  **Trial acronym** | **Singletons/ Twins/ Triplets** | **Risk factor** | **Number of**  **randomizedpatients**  **n=** | **Results** | | **Comments** | **Directness *** | **Study limitations *** | **Precision *** |
| --- | --- | --- | --- | --- | --- | --- | --- | --- | --- |
|  |  |  |  | **Intervention Progesterone** | **Control Placebo** |  |  |  |  |
|  |  |  |  |  |  |  |  |  |  |
| Rode, 2011 Denmark and Austria PREDICT | Twins (DA) | MC  I: 43/334 (12.9%)  C: 57/343 (16.6%) | I: 334  C: 343 | 200 mg vaginal progesterone/d (pessary)  16/332 (4.8%)  OR 1.4 (95% CI 0.6-3.0)  No p values | Placebo 12/341 (3.5%) | Not PO | + | + | + |
| Serra, 2013 Spain | Twins (DCDA) | MAR  I1: 92/96 (95.8%)  I2: 94/97 (96.9%)  C:96/97 (99.0%) | I1: 98 I2:98 C: 98 | I1: 200 mg vaginal progesterone (pessary) /d  2/97 (2.1%)  I2: 400 mg vaginal progesterone (pessary)/d  4/97 (4.1%)  I1 + I2: 6/194 (3.1%) I1, I2, C= NS I1+I2 vs C= NS  I1 vs I2=NS | Placebo 5/96 (5.2%) | Not PO | ? | ? | - |
| Lim, 2011  The Netherlands AMPHIA | Multi-fetal pregnan-  cies | Triplets/+ I: 9 (3%)  C: 9 (3%)  (incl.one quadruplet) MC  I: 57 (17%)  C: 57 (17%)  Fertility treatment I: 140 (42%)  C: 120 (36%) | I: 336 women  C: 335 women | 17-OHPC 250 mg im/w 7/336 (2%)  RR 0.99 (95% CI 0.35-2.78)  No p value | Placebo 7/332 (2%) | Not PO | + | + | ? |
| Combs, 2010 USA | Triplets | Trichorionic triamniotic triplets MAR  I: 90%  C: 84% | I: 56  C: 25 | 17-OHPC 250 mg im/w 9/55 (16%)  RR 1.43 (95% CI 0.31-9.01) p=0.77 | Placebo 3/25 (12%) | Not PO | + | ? | ? |

* + No or minor problems

? Some problems

- Major problems

Prevention of preterm birth

STable 4.1.23. cont. Intervention progesterone

Outcome variable: Maternal morbidity, gestational diabetes mellitus (GDM)

| **Author, year Country**  **Trial acronym** | **Singletons/ Twins/ Triplets** | **Risk factor** | **Number of**  **randomizedpatients**  **n=** | **Results** | | **Comments** | **Directness *** | **Study limitations *** | | | **Precision *** |
| --- | --- | --- | --- | --- | --- | --- | --- | --- | --- | --- | --- |
|  |  |  |  | **Intervention Progesterone** | **Control Placebo** |  |  |  |  |  |  |
|  |  |  |  |  |  |  |  | | | | |
| Gyamfi, 2009 Secondary analysis of Meis, 2003 (singletons)  and Rouse, 2007 (twins)  USA | Mixed singletons and  twins | Singletons:  Women with previous sPTB  Twins: Unselected DA twins | Singleton I: 293  C: 148  Twins: I: 323  C: 330 | 17-OHPC 250 mg im/w Singletons  17*/293 (5.8%)  RR 1.23 (95% CI 0.52-2.89) p=0.64  Twins 24*/323 (7.4%)  RR 0.98 (95% CI 0.57-1.68) p=0.94 | Placebo Singletons 7*/148 (4.7%)  Twins 25*/330 (7.6%) | Not PO  *Numbers calculated from percentages in article | Meis, 2003 | | | | |
|  |  |  |  |  |  |  | ? | | ? | ? | |
|  |  |  |  |  |  |  | Rouse, 2007 | | | | |
|  |  |  |  |  |  |  | + | | ? | + | |

17-OHPC;17-α-hydroxyprogesterone caproate, ART; assisted reproductive technology, C; control, CL; cervical length, DA; diamniotic, DC; dichorionic, DCDA; dichorionic diamniotic, FFN; fetal fibronectin, GA; gestational age, I; intervention, IUFD; intrauterine fetal death, MAR; medically assisted reproduction; MC; monochorionic, MCDA; monochorionic diamniotic, OR; odds ratio, PO; primary outcome, PTB; preterm birth, RR; risk ratio, sPTB: spontaneous preterm birth, TVS; transvaginal scan

§

* + No or minor problems

? Some problems

- Major problems

Prevention of preterm birth

STable 4.1.24. Intervention progesterone

Outcome variable: Maternal morbidity, intrahepatic cholestasis

| **Author, year Country**  **Trial acronym** | **Singletons/ Twins/ Triplets** | **Risk factor** | **Number of**  **randomizedpatients**  **n=** | **Results** | | **Comments** | **Directness *** | **Study limitations *** | **Precision *** |
| --- | --- | --- | --- | --- | --- | --- | --- | --- | --- |
|  |  |  |  | **Intervention Progesterone** | **Control Placebo** |  |  |  |  |
|  |  |  |  |  |  |  |  |  |  |
| Ashoush, 2017 Egypt | Singletons | Previous sPTB (<37 w) | I: 106  C: 106 | 400 mg oral progesterone/d Elevated liver enzymes 0/96 | Placebo Elevated liver enzymes  0/91 | Not PO  OBS critical comment to article by Katsanevakis, Mol and Thornton concerning recruitment and results  OBS High rates of cerclage in both groups | - | ? | - |
| Norman, 2016 UK  (UK 65 hospitals,  Sweden 1 hospital) OPPTIMUM | Singletons | FFN pos group: Any of previous PTB, second trimester loss, cervical surgery  FFN neg group: previous sPTB <34 w or  short TVS CL ≤25 mm | I: 618  C: 610 | 200 mg vaginal progesterone/d Cholestasis  4/593 (1%)  No statistics | Placebo Cholestasis 6/589 (1%) | Not PO | + | + | + |
| Brizot, 2015 Brazil | Twins DA | MC I: 25%  C: 19%  Only naturally conceived,  no history of PTB | I: 195  C: 195 | 200 mg vaginal natural progesterone/d Elevated liver enzymes 0/188  NA  No p value | Placebo  Elevated liver enzymes 2/190 (1.0%) | Not PO | ? | ? | - |
| Rehal, 2021  UK (+5 other European countries) | Twins | MC I: 23%  C: 23% ART  I: 34%  C: 35% | I: 582  C: 587 | 600 mg vaginal progesterone/d Cholestasis  0/596  Elevated liver enzymes 1/596  No statistics | Placebo Cholestasis 1/598  Elevated liver enzymes 1/598 | Not PO Cholestasis > 2 days  hospitalization | + | + | ? |
| Rode, 2011 Denmark and Austria PREDICT | Twins (DA) | MC  I: 43/334 (12.9%)  C: 57/343 (16.6%) | I: 334  C: 343 | 200 mg vaginal progesterone/d (pessary)  Elevated liver enzymes 11/332 (3.3%)  OR 0.4 (95% CI 0.2-0.9)  No p value | Placebo  Elevated liver enzymes 25/341 (7.3%) | Not PO | + | + | + |

Prevention of preterm birth

STable 4.1.24. cont. Intervention progesterone

Outcome variable: Maternal morbidity, intrahepatic cholestasis

* + No or minor problems

? Some problems

- Major problems

| **Author, year Country**  **Trial acronym** | **Singletons/ Twins/ Triplets** | **Risk factor** | **Number of**  **randomizedpatients**  **n=** | **Results** | | **Comments** | **Directness *** | **Study limitations *** | **Precision *** |
| --- | --- | --- | --- | --- | --- | --- | --- | --- | --- |
|  |  |  |  | **Intervention Progesterone** | **Control Placebo** |  |  |  |  |
|  |  |  |  |  |  |  |  |  |  |
| Serra, 2013 Spain | Twins (DCDA) | MAR  I1: 92/96 (95.8%)  I2: 94/97 (96.9%)  C: 96/97 (99.0%) | I1: 98 I2:98 C: 98 | I1: 200 mg vaginal progesterone (pessary) /d Cholestasis  1/97 (1.0%)  I2: 400 mg vaginal progesterone (pessary)/d Cholestasis  5/97 (5.2%)  I1 + I2: Cholestasis 6/194 (3.1%) I1, I2, C=NS  I1+I2 vs C=NS I1 vs I2=NS | Placebo  Cholestasis 0/96 | Not PO | ? | ? | - |

17-OHPC;17-α-hydroxyprogesterone caproate, ART; assisted reproductive technology, C; control, CL; cervical length, DA; diamniotic, DC; dichorionic, DCDA; dichorionic diamniotic, FFN: fetal fibronectine, GA; gestational age, I; intervention, IUFD; intrauterine fetal death, MAR; medically assisted reproduction; MC; monochorionic, MCDA; monochorionic diamniotic, NS; not significant, OR; odds ratio, PTB; preterm birth, RR; risk ratio, sPTB: spontaneous preterm birth, TVS; transvaginal scan

* + No or minor problems

? Some problems

- Major problems

Prevention of preterm birth

STable 4.1.25. Intervention progesterone

Outcome variable: Maternal morbidity, infections including chorioamnionits

| **Author, year Country**  **Trial acronym** | **Singletons/ Twins/ Triplets** | **Risk factor** | **Number of**  **randomizedpatients**  **n=** | **Results** | | | **Comments** | **Directness *** | **Study limitations *** | **Precision *** | |
| --- | --- | --- | --- | --- | --- | --- | --- | --- | --- | --- | --- |
|  |  |  |  | **Intervention Progesterone** | | **Control**  **Placebo** |  |  |  |  |  |
|  |  |  |  | |  |  |  |  |  |  |  |
| Ashoush, 2017 Egypt | Singletons | Previous sPTB (<37 w) | I: 106  C: 106 | | 400 mg oral progesterone/d CA 9/96 (9.3%)  p=0.55  PP sepsis 4/96 (4.1%) p=0.13 | Placebo  CA 12/91 (13.1%)  PP sepsis 10/91 (0.9%) | Not PO  OBS critical comment to article by Katsanevakis, Mol and Thornton concerning recruitment and results  NB: High rates of cerclage in both groups | - | ? | - |  |
| Blackwell, 2020 USA PROLONG | Singletons | Previous singleton sPTB | I: 1130  C: 578  Randomized2:1 | | 17-OHPC 250 mg im/w CA 9/1130 (0.8%)  RR 2.24 (95% CI 0.48-10.41)  No p value | Placebo  CA 2/578 (0.3%) | Not PO | + | ? | ? |  |
| Meis, 2003 USA | Singletons | Previous sPTB | I:310 C:153  Randomized2:1 | | 17-OHPC 250 mg im/w CA 11/306 (3.6%)  RR 1.09 (95% CI 0.39-3.09)  No p value | Placebo (castor oil) CA 5/153(3.3%) | Not PO | ? | ? | ? |  |
| Norman, 2016 UK  (UK 65 hospitals,  Sweden 1 hospital) OPPTIMUM | Singletons | FFN pos group: Any of previous PTB, second trimester loss, cervical surgery  FFN neg group: previous sPTB <34 w or  short TVS CL ≤25 mm | I: 618  C: 610 | | 200 mg progesterone/day CA  9/83 (11%)  No statistics | Placebo CA  10/84 (12%) | Not PO  Infectious morbidity: antibiotics during delivery, and histology CA based on placenta examination. | + | + | + |  |
| Price, 2021 Zambia | Singletons | HIV | I: 399 C:401 | | 17-OHPC 250 mg/w  CA 1/399 (<1%)  No statistics | Placebo CA 0/401 (0%) | Not PO | ? | + | ? |  |
| Van Os, 2015 The Netherlands TRIPLE P | Singletons | Short TVS CL  ≤30 mm,  no previous PTB | I: 41  C: 39 | | 200 mg micronized progesterone vaginal/d  1/41 (12%)  RR 1.1 (95% CI 0.083-15)  No p value | Placebo 1/39 (11%) | Not PO | + | ? | - |  |

Prevention of preterm birth

STable 4.1.25. cont. Intervention progesterone

Outcome variable: Maternal morbidity, infections including chorioamnionits

* + No or minor problems

? Some problems

- Major problems

| **Author, year Country**  **Trial acronym** | **Singletons/ Twins/ Triplets** | **Risk factor** | **Number of**  **randomizedpatients**  **n=** | **Results** | | | **Comments** | **Directness *** | **Study limitations *** | **Precision *** | |
| --- | --- | --- | --- | --- | --- | --- | --- | --- | --- | --- | --- |
|  |  |  |  | **Intervention Progesterone** | | **Control**  **Placebo** |  |  |  |  |  |
|  |  |  |  | |  |  |  |  |  |  |  |
| Combs, 2011 USA | Twins DCDA | 20% fetal reduction ART  I: 66% C:58%  Prior PTB: I: 12%  C: 13% | I: 160  C: 80  Randomized2:1 | | 17-OHPC 250 mg (in 1 mL castor oil) im/w  CA 3/158 (2%) p=0.55  Sepsis 0/155 NA  PP endometritis 0/155 NA | Placebo (1 mL castor oil) CA 0/78  Sepsis 0/78  PP endometritis 0/78 | Not PO | + | ? | ? |  |
| Norman, 2009 UK  (9 hospitals) STOPPIT | Twins | MC twins I: 46/247 C: 45/247  No MA twins | I: 247  C: 247 | | 90 mg vaginal progesterone /day (Crinone)  CA or intrauterine infection 0  No denominator | Placebo  CA or intrauterine infection 0  No denominator | Not PO | + | + | ? |  |
| Rouse, 2007 USA SSTARS | Twins (DA) | MC  I: 59/327 (18%)  C: 57/334 (17.1%) | I: 327  C: 334 | | 17-OHPC 250 mg im/w CA 6/324 (1.9%)  RR 1.0 (95% CI 0.3-3.1)  No p value | Placebo  CA 6/330 (1.8%) | Not PO | + | ? | + |  |
| Lim, 2011  The Netherlands AMPHIA | Multifetal pregnancies | Triplets/+ I: 9 (3%)  C: 9 (3%)  (incl.one quadruplet) MC  I: 57 (17%)  C: 57 (17%)  Fertility treatment I: 140 (42%)  C: 120 (36%) | I: 336  women C: 335  women | | 17-OHPC 250 mg im/w CA 20/336 (6%)  RR 1.30 (95% CI 0.68-2.49)  No p value | Placebo  CA 15/332(5%) | Not PO  CA as determined with placenta histology | + | + | ? |  |

Prevention of preterm birth

STable 4.1.25. cont. Intervention progesterone

Outcome variable: Maternal morbidity, infections including chorioamnionits

* + No or minor problems

? Some problems

- Major problems

| **Author, year Country**  **Trial acronym** | **Singletons/ Twins/ Triplets** | **Risk factor** | **Number of**  **randomizedpatients**  **n=** | **Results** | | | **Comments** | **Directness *** | **Study limitations *** | **Precision *** | |
| --- | --- | --- | --- | --- | --- | --- | --- | --- | --- | --- | --- |
|  |  |  |  | **Intervention Progesterone** | | **Control**  **Placebo** |  |  |  |  |  |
|  |  |  |  | |  |  |  |  |  |  |  |
| Crowther, 2017 Australia PROGRESS | Mixed Singletons n=775 (98.5%  Twins n=12 (1.5%) | Previous sPTD  <37 w | I: 398 (390  singletons and 8 twin pregnancies) 406 infants  C: 389  (385  singletons and 4 twin  pregnancies) 393 infants | | 100 mg vaginal progesterone pessary/d  CA 19/398 (4.8%)  RR 1.43 (95% CI 0.72-2.85) p=0.312  aRR 1.49 (95% CI 0.75–2.98)  adjusted p=0.253 Adjusted for GA at randomization,  GA of previous PTB, and reason for previous PTB | Placebo  CA 13/389 (3.3%) | Not PO  CA treated with antibiotics | + | + | ? |  |
| Caritis, 2009 USA  SSTARS | Triplets | 30% DC or unknown chorionicity  ART: 70% | I: 71  C: 63 | | 17-OHPC 250 mg im/w CA 1/71 (1%)  NA | Placebo CA 0/63 |  | ? | ? | ? |  |
| Combs, 2010 | Triplets | Trichorionic triamniotic triplets MAR  I: 90%  C: 84% | I: 56  C: 25 | | 17-OHPC 250 mg im/w CA 5/56 (9%)  RR 1.13 (95% CI 0.17-12.65) p>0.99  Sepsis 1/56 (2%) p>0.99  PP endometritis 2/56 (4%) p>0.99 | Placebo CA 2/25 (8%)  Sepsis 0/25  PP endometritis 0/25 | Not PO | + | ? | ? |  |

Prevention of preterm birth

STable 4.1.25. cont. Intervention progesterone

Outcome variable: Maternal morbidity, infections including chorioamnionits

* + No or minor problems

? Some problems

- Major problems

| **Author, year Country**  **Trial acronym** | **Singletons/ Twins/ Triplets** | **Risk factor** | **Number of**  **randomizedpatients**  **n=** | **Results** | | **Comments** | **Directness *** | **Study limitations *** | **Precision *** |
| --- | --- | --- | --- | --- | --- | --- | --- | --- | --- |
|  |  |  |  | **Intervention Progesterone** | **Control**  **Placebo** |  |  |  |  |

| **Other interventions in comparison with progesterone** | | | | | | | | | |
| --- | --- | --- | --- | --- | --- | --- | --- | --- | --- |
| Keeler, 2009a USA | Singletons | Short TVS CL ≤25 mm in women with risk factors for PTB (history of sPTB, second-  trimester pregnancy loss, previous cervical surgery  or uterine anomaly) | I: 42  C: 37 | Cerclage (McDonald) CA 12/42 (28.6%)  RR 0.76 (95% CI 0.35-1.65)  No p value | 17-OHPC 250 mg weekly CA 8/37 (21.6%) | Not PO | + | ? | - |
| Dang 2019 | Twins | Short TVS CL ≤38  mm | I:150 C: 150 | Arabin pessary | 400 mg vaginal progesterone  /d | Not PO | ? | ? | ? |
| Vietnam |  | (women with history of |  | ABU treated with antibiotics | ABU treated with antibiotics |  |  |  |  |
| (single center) |  | cervical surgery |  | 14/148 (9.5%) | 12/149 (8.1%) |  |  |  |  |
|  |  | excluded) |  | RR 1.17 (95% CI 0.56-2.45) |  |  |  |  |  |
|  |  |  |  | p=0.69 |  |  |  |  |  |
|  |  |  |  | CA 0/148 (0%) | CA 0/148 (0%) |  |  |  |  |
|  |  |  |  | No statistics |  |  |  |  |  |
|  |  |  |  | Genital tract infection | Genital tract infection |  |  |  |  |
|  |  |  |  | 5/148 (3.4) | 7 /149 (4.7%) |  |  |  |  |
|  |  |  |  | RR 0.72 (95% CI 0.23-2.21) |  |  |  |  |  |
|  |  |  |  | p=0.77 |  |  |  |  |  |

17-OHPC;17-α-hydroxyprogesterone caproate, ABU; asymptomatic bacteriuria, aRR, adjusted relative risk, ART; assisted reproductive technology, C; control, CA; chorioamnionitis, CL; cervical length, DA; diamniotic, DC; dichorionic, DCDA; dichorionic diamniotic, GA; gestational age, I; intervention, IUFD; intrauterine fetal death, MAR; medically assisted reproduction; MC; monochorionic, MCDA; monochorionic diamniotic, NB; nota bene, OR; odds ratio, PP; postpartum, PTB; preterm birth, RR; risk ratio, sPTB: spontaneous preterm birth, TVS; transvaginal scan

Prevention of preterm birth

STable 4.1.26 Intervention progesterone

Outcome variable: Maternal morbidity, preterm prelabor rupture of membranes (PPROM)

* + No or minor problems

? Some problems

- Major problems

| **Author, year Country**  **Trial acronym** | **Singletons/ Twins/ Triplets** | **Risk factor** | | **Number**  **of randomized**  **patients**  **n=** | | **Results** | | **Comments** | **Directness *** | **Study limitations *** | **Precision *** |
| --- | --- | --- | --- | --- | --- | --- | --- | --- | --- | --- | --- |
|  |  |  |  |  |  | **Intervention** | **Control** |  |  |  |  |
|  |  | |  | |  |  |  |  |  |  |  |
| Aflatoonian, 2013 Iran | Singletons | | ART pregnancies | | I: 52  C: 47 | 17-OHPC 250 mg im/w 4/52 (7.7%)  No statistics | Placebo 2/47 (4.3%) | Not PO | ? | - | - |
| Ashoush, 2017 Egypt | Singletons | | Previous sPTB (<37 w) | | I: 106  C: 106 | 400 mg oral progesterone/d 36/96 (37.5%)  p=0.27 | Placebo 40/91 (44.0%) | Not PO  NB critical comment to article by Katsanevakis, Mol and Thornton concerning recruitment and results  NB High rates of cerclage in both groups | - | ? | - |
| Norman, 2016 UK  (UK 65 hospitals,  Sweden 1 hospital) OPPTIMUM | Singletons | | FFN pos group: Any of previous PTB, second trimester loss, cervical surgery  FFN neg group: previous sPTB <34 w or  short TVS CL ≤25 mm | | I: 618  C: 610 | 200 mg progesterone/day  65/593 (11%)  No statistics | Placebo 72/590 (12%) | Not PO | + | + | + |
| O’Brien 2007  USA (+4 other countries) | Singletons | | Previous sPTB | | I: 332  C: 327 | 90 mg vaginal progesterone gel 8%/d  37/309 (12.0%)  0.95 (95%CI 0.58-1.53)  No p value | Placebo 38/302 (12.6%) | Not PO | ? | ? | ? |
| Price, 2021 Zambia | Singletons | | HIV | | I: 399 C:401 | 17-OHPC 250 mg/w  5/399 (1%)  RR 0.7 (95% CI 0.2-2.2)  No p value | Placebo 7/401 (2%) | Not PO | ? | + | ? |
| Van Os, 2015 The Netherlands TRIPLE P | Singleton | | Short TVS CL  ≤30 mm,  no previous PTB | | I: 41  C: 39 | 200 mg micronized progesterone vaginal/d  3/41 (7%)  RR 0.59 (95% CI 0.15–2.3)  No p value | Placebo 5/39 (13%) | Not PO | + | ? | - |

* + No or minor problems

? Some problems

- Major problems

Prevention of preterm birth

STable 4.1.26 cont. Intervention progesterone

Outcome variable: Maternal morbidity, preterm prelabor rupture of membranes (PPROM)

| **Author, year Country**  **Trial acronym** | **Singletons/ Twins/ Triplets** | **Risk factor** | | **Number**  **of randomized**  **patients**  **n=** | | **Results** | | **Comments** | **Directness *** | **Study limitations *** | **Precision *** |
| --- | --- | --- | --- | --- | --- | --- | --- | --- | --- | --- | --- |
|  |  |  |  |  |  | **Intervention** | **Control** |  |  |  |  |
|  |  | |  | |  |  |  |  |  |  |  |
| Awwad, 2015 Libanon PROGESTWIN | Twins, unselected | | ART 75%  MC 17% | | I:197 C: 96  Randomized 2:1 | 17-OHPC 250 mg im/w 6/131 (4.6%)  OR 1.3 (95% CI 0.2–6.6) p=0.55 | Placebo (castor oil) 2/56 (3.6%) | Not PO | + | + | ? |
| Briery, 2009 USA | Twins, unselected | | 1/3 previous PTB | | I: 16  C: 14 | 17-OHPC 250 mg im/w 1/16 (6%) p=0.525 | Placebo 1/14 (7%) | Not PO Numbers for PTB <37 w calculated from table 2 | ? | ? | - |
| Serra, 2013 Spain | Twins (DCDA) | | MAR  I1: 92/96 (95.8%)  I2: 94/97 (96.9%)  C: 96/97 (99.0%) | | I1: 98 I2:98 C: 98 | I1: 200 mg vaginal progesterone (pessary) /d  4/97 (4.1%)  I2: 400 mg vaginal progesterone (pessary)/d  1/97 (1.0%)  I1 + I2: 5/194 (2.6%)  I1 vs I2 vs C= NS  I1+I2 vs C= NS I1 vs I2=NS | Placebo 3/96 (3.1%) | Not PO | ? | ? | - |
| Lim, 2011  The Netherlands AMPHIA | Multifetal pregnancies | | Triplets/+ I: 9 (3%)  C: 9 (3%)  (incl.one quadruplet) MC  I: 57 (17%)  C: 57 (17%)  Fertility treatment I: 140 (42%)  C: 120 (36%) | | I: 336 women  C: 335 women | 17-OHPC 250 mg im/w 34/336 (10%)  RR 1.22 (95% CI 0.76-1.96)  No p value | Placebo 28/332(8%) | Not PO | + | + | ? |

* + No or minor problems

? Some problems

- Major problems

Prevention of preterm birth

STable 4.1.26 cont. Intervention progesterone

Outcome variable: Maternal morbidity, preterm prelabor rupture of membranes (PPROM)

| **Author, year Country**  **Trial acronym** | **Singletons/ Twins/ Triplets** | **Risk factor** | | **Number**  **of randomized**  **patients**  **n=** | | **Results** | | **Comments** | **Directness *** | **Study limitations *** | **Precision *** |
| --- | --- | --- | --- | --- | --- | --- | --- | --- | --- | --- | --- |
|  |  |  |  |  |  | **Intervention** | **Control** |  |  |  |  |
|  |  | |  | |  |  |  |  |  |  |  |
| Cetingoz, 2011 Turkey | Mixed Singletons I: 41/80 (51.3%) C: 42/70 (60%)  Twins I: 39/80 (48.7%)  C: 28/70 (40.0) | | Twin pregnancy, previous sPTD, uterine malformation | | I: 84  C: 76 | 100 mg vaginal progesterone/d Singletons and twins  3/80 (3.8%) p>0.05 | Placebo Singletons and twins  2/70 (2.9%) | Not PO  No data for singletons and twins. respectively | ? | ? | - |
| Crowther, 2017 Australia PROGRESS | Mixed Singletons n=775 (98.5%  Twins n=12 (1.5%) | | Previous sPTD  <37 w | | I: 398 (390  singletons and 8 twin pregnancies) 406 infants  C: 389  (385 singletons  and 4 twin pregnancies) 393 infants | 100 mg vaginal progesterone pessary/d  51/398 (12.8%)  RR 1.13 (95% CI 0.78-1.65) p=0.518  aRR 1.14 (95% CI 0.78-1.66)  adjusted p=0.500 Adjusted for GA at randomization,  GA of previous PTB, and reason for previous PTB | Placebo 44/389 (11.3%) | Not PO | + | + | ? |
| Caritis, 2009 USA SSTARS | Triplets | | 30% DC or unknown chorionicity  ART: 70% | | I: 71  C: 63 | 17-OHPC 250 mg im/w 6/71 (8%)  RR 0.8 (95% CI 0.3-2.1)  No p value | Placebo 7/63 (11%) | Not PO | ? | ? | ? |

Prevention of preterm birth

STable 4.1.26 cont. Intervention progesterone

Outcome variable: Maternal morbidity, preterm prelabor rupture of membranes (PPROM)

* + No or minor problems

? Some problems

- Major problems

| **Author, year Country**  **Trial acronym** | **Singletons/ Twins/ Triplets** | | **Risk factor** | **Number**  **of randomized patients**  **n=** | | **Results** | | **Comments** | **Directness *** | **Study limitations *** | **Precision *** |
| --- | --- | --- | --- | --- | --- | --- | --- | --- | --- | --- | --- |
|  |  |  |  |  |  | **Intervention** | **Control** |  |  |  |  |
|  | | | | | | | | | | | |
| **Other interventions in comparison with progesterone** | | | | | | | | | | | |
| Keeler, 2009a USA | Singletons | Short TVS CL ≤25 in women with risk | | | I: 42  C: 37 | Cerclage (McDonald) 13/42 (32.5%) | 17-OHPC 250 mg weekly 13/37 (37.1%) | Not PO | + | ? | - |
|  |  | factors for PTB | | |  | RR 1.14 (95% CI 0.61-2.12) |  |  |  |  |  |
|  |  | (history of sPTB, | | |  | No p-value |  |  |  |  |  |
|  |  | second- | | |  |  |  |  |  |  |  |
|  |  | trimester pregnancy | | |  |  |  |  |  |  |  |
|  |  | loss, previous cervical | | |  |  |  |  |  |  |  |
|  |  | surgery | | |  |  |  |  |  |  |  |
|  |  | or uterine anomaly) | | |  |  |  |  |  |  |  |
| Cruz-Melguizo, 2018 | Singletons | Short TVS CL ≤25 mm (women with cervical | | | I: 125  C: 118 | Pessary 12/125 (10%) | 200 mg vaginal progesterone/d 11/118 (9%) | Not PO | + | ? | ? |
| Spain |  | surgery and ≥3 | | |  | RD 0.28 (95% CI -7.08 to 7.64) |  |  |  |  |  |
| (27 centers) |  | previous PTBs were  excluded) | | |  | p=0.94 |  |  |  |  |  |

17-OHPC; 17-α-hydroxyprogesterone caproate, aRR; adjusted risk ratio, ART; assisted reproductive technology, C; control, CL; cervical length, DA; diamniotic, DC; dichorionic, DCDA; dichorionic diamniotic, GA; gestational age, HIV; human immunodeficiency virus I; intervention, IUFD; intrauterine fetal death, MAR; medically assisted reproduction; MC; monochorionic, MCDA; monochorionic diamniotic, NS; not significant, OR; odds ratio, PO; primary outcome, PTB; preterm birth, RR; risk ratio, RD; risk difference, sPTB: spontaneous preterm birth, TVS; transvaginal scan
