# Supplementary material for: Progesterone, cerclage, pessary, or acetylsalicylic acid for prevention of preterm birth in singleton and multifetal pregnancies – A systematic review and meta-analyses
Source: Front Med (Lausanne). 2023 Feb 28;10:1111315. doi: 10.3389/fmed.2023.1111315 (PMC10015499; doi:10.3389/fmed.2023.1111315)
Supplement: Supplementary file 1 [file Data_Sheet_1.zip › Data Sheet 1_corrected/Appendix 4.2 Outcome tables_Cerclage.docx]

**Progesterone, cerclage, pessary, or acetylsalicylic acid for prevention of preterm birth in**

**singleton and multifetal pregnancies**

Appendix 4.2 [Outcome tables](#_Toc104881914) cerclage in singleton and multifetal pregnancies

**Preterm birth**

STable 4.2.1.a Any preterm birth <37 weeks

STable 4.2.2.a Any preterm birth <35 weeks

STable 4.2.3.a Any preterm birth <34 weeks

STable 4.2.3.b Spontaneous preterm birth <34 weeks

STable 4.2.4.a Any preterm <33 weeks

STable 4.2.5.a Any preterm birth <32 weeks

STable 4.2.5.b Spontaneous preterm birth <32 weeks

STable 4.2.6.a Any preterm birth <28 weeks

STable 4.2.6.b Spontaneous preterm birth <28 weeks

**Gestational age and birth weight**

STable 4.2.7 Gestational age at delivery

STable 4.2.8 Low birth weight (<2500g)

STable 4.2.9 Very low birth weight (<1500g)

**Perinatal mortality and neonatal morbidity**

STable 4.2.10 Perinatal mortality

STable 4.2.11 Neonatal mortality <7 days

STable 4.2.12 Neonatal mortality <28 days

STable 4.2.13 Composite adverse neonatal outcome

STable 4.2.14 Respiratory distress syndrome

STable 4.2.15 Bronchopulmonary dysplasia

STable 4.2.16 Intraventricular hemorrhage or periventricular leukomalacia

STable 4.2.17 Necrotizing enterocolitis

STable 4.2.18 Neonatal sepsis

STable 4.2.19 Retinopathy of prematurity

STable 4.2.20 Admittance to neonatal intensive care unit

**Maternal mortality and morbidity**

STable 4.2.21 Infections

STable 4.2.22 Preterm prelabor rupture of membranes

| **Author, year Country**  **Trial acronym** | **Singletons/ Twins/ Triplets** | **Risk factor** | **Number of**  **randomized patients**  **n=** | **Results** | | **Comments Risk factor** | **Directness *** | **Study limitations *** | **Precision *** |
| --- | --- | --- | --- | --- | --- | --- | --- | --- | --- |
|  |  |  |  | **Intervention** | **Control** |  |  |  |  |

| Ezechi, 2004 Nigeria | Not stated | History of PTB: 1 (63-69%)  2 (21-32%)  3 (5-9%) | I: 38  C: 43 | McDonald cerclage 4/38 (10.5%)  OR 0.20  (95% CI 0.05-0.74) p=0.012 | No cerclage 16/43 (37.2%) | PO not defined Outcome stated as PTB, no  definition but seems to be <37 w as described in text.  Singletons/twins not stated but singletons correspond to neonatal outcomes. | - | ? | ? |
| --- | --- | --- | --- | --- | --- | --- | --- | --- | --- |
| Lazar, 1984 France | Singletons | Composite score of a combination of:  History of PTB  29-36 w, history of previous miscarriage, prior threatening PTL treated by hospitalization, uterine malformation, previous forced cervical dilatation, low lying  placenta with bleeding, CL <2 cm, cx open for inner os | I: 268  C: 238 | McDonald cerclage 18/268 (6.7%) p=0.35  RR not presented | No cerclage 13/238 (5.5%) | PO not defined | ? | ? | ? |
| Otsuki, 2016 Japan | Singletons | General population screening: TVS CL <25 mm  History of PTB 11-15%, history of previous abortion 20% | I1  (Shirodkar): 35  I2  (McDonald): 36  C: 35 | I1: Shirodkar cerclage 7/34 (20%)  p=0.50  RR not presented  I2: McDonald cerclage 11/34 (32.4%)  p=0.99  RR not presented | No cerclage, bedrest 10/33 (30.3%) | Not PO | ? | ? | ? |
| Owen, 2009 USA | Singletons | History of sPTB <34 w or PPROM  + short TVS CL <25 mm | I: 149  C: 153 | McDonald cerclage 66/148 (45%) p=0.01  RR not presented | No cerclage 91/153 (60%) | Not PO | ? | ? | ? |
| Rush, 1984 South Africa | Singletons | History of previous late miscarriage or PTB out of at least one spontaneous between 14-36 and 2,3, or 4 previous pregnancies  ending spontaneously before 37 w | I: 96  C: 98 | McDonald cerclage 33/96 (34.4%)  NS | No cerclage 31/98 (31.6%) | PO not defined | ? | ? | ? |

| **Author, year Country**  **Trial acronym** | **Singletons/ Twins/ Triplets** | **Risk factor** | **Number of**  **randomized patients**  **n=** | **Results** | | **Comments Risk factor** | **Directness *** | **Study limitations *** | **Precision *** |
| --- | --- | --- | --- | --- | --- | --- | --- | --- | --- |
|  |  |  |  | **Intervention** | **Control** |  |  |  |  |

| Macnaughton MRC/RCOG, 1993  UK, France, Hungary, Norway, Italy, Belgium, Zimbabwe, South Africa, Iceland, Ireland, the Netherlands, Canada | Singletons and twins (28/1292, 2%)  Twins: I: 12  C: 16 | Included if uncertainty if cerclage or not for risk patients: Previous PTB, previous second trimester miscarriage, previous early abortion, cervical amputation, cone biopsy, uterine anomaly, twin pregnancy | I: 647  C: 645 | Cerclage (not prespecified)  Singletons and twins: 169/647 (26%)*  OR 0.80, 95% CI 0.63-1.02 p=0.07  Sensitivity analysis: Excluding early miscarriage (<13 w) in numerator and denominator (n=12) 157/635 (24.7%)  No statistics in article  Singletons (calculated, and same numbers as in Alfirevic, Cochrane, 2017)  161/635 (25.4%)*  No statistics Twins  8/12 (67%)*, NS | No cerclage  Singletons and twins: 198/645 (31%)*  Sensitivity analysis: Excluding early miscarriage  (<13 w) in numerator and denominator (n=9) 189/636 (29.7%)  Singletons (calculated, and same numbers as in Alfirevic,  Cochrane, 2017) 190/629 (30.2%)*  Twins 8/16 (50%)* | PO, then PO changed to <33 w after study initiation  *All miscarriages included in numerator  and denominator  I: 43/647 and C: 50/645 | ? | ? | ? |
| --- | --- | --- | --- | --- | --- | --- | --- | --- | --- |
| Rust, 2000 USA | Singletons and twins (7/61, 11.5%  twins) | History of PTB, second trimester pregnancy loss, previous cervical surgery, uterine anomaly, multifetal pregnancy  and a  TVS CL <25 mm or dilated internal os | I: 31  C: 30 | McDonald cerclage and modified bedrest  18/31 (58.1%), p=0.4  RR not presented | Modified bedrest 3/30 (43.3%) | 5 twin and 2 triplets evenly distributed between the two groups, though not defined in the outcomes. Not included in meta-analysis due to multifetal pregnancies >10% | - | - | ? |
| Dor, 1982  Israel | Twins | Twin pregnancy after OI | I: 22/44 C: 23/46 | McDonald cerclage 10/22 (45.4%), NS  RR not presented | No cerclage 11/23 (47.8%) | PO not defined | ? | - | - |

| **Author, year Country**  **Trial acronym** | **Singletons/ Twins/ Triplets** | **Risk factor** | **Number of**  **randomized patients**  **n=** | **Results** | | **Comments Risk factor** | **Directness *** | **Study limitations *** | **Precision *** |
| --- | --- | --- | --- | --- | --- | --- | --- | --- | --- |
|  |  |  |  | **Intervention** | **Control** |  |  |  |  |

| **Systematic reviews**  (only articles with results not shown in original articles are included here) Assessment of Directness, Study limitations and Precision refer to the original articles | | | | | | | | | |
| --- | --- | --- | --- | --- | --- | --- | --- | --- | --- |
| **Alfirevic, 2017 Cochrane** | **Singletons** | | | | | | | | |
| Althuisius, 2001 The Netherlands CIPRACT | Singletons | History of PTB <34 w, PPROM  <32 w, cold knife conization, diethylstilbestrol exposure, or uterine anomaly  and TVS CL <25 mm | I: 20 C:16 | McDonald cerclage and bedrest  4/19 (21.1%)  No statistics | Bedrest 10/16 (62.5%) | Not PO | + | - | - |
| To, 2004 UK  (6 countries; UK, Brazil, South Africa, Slovenia, Greece, Chile; 12  hospitals) | Singletons | Short TVS CL ≤15 mm Previous cervical surgery: I: 6%  C: 7% | I: 127  C: 126 | Shirodkar cerclage 41/127 (32.3%) | No cerclage 63/126 (50%) | Not PO | ? | ? | ? |
| Berghella, 2004 USA  (2 centers) | Singletons and twins (4/61)  7% twins | ≥1 of high-risk factors for PTB (≥1 PTB <35 w,  ≥2 curettages, diethylstilbestrol exposure, cone biopsy, Mullerian anomaly,  or twin pregnancy) and or TVS CL  <25 mm or significant funneling | I: 31  C: 30 | McDonald cerclage with bedrest  All 17/31 (54.8%)  No statistics Singletons 14/28 (50.0%)  No statistics Twins  3/3 (100%)  No statistics | No cerclage, bedrest All 22/30 (73.3%)  Singletons 21/29 (72.4%)  Twins 1/1 (100%) | Not PO  PTB in twins calculated from original article, 3 women with twin pregnancies in the intervention group, all early PTB at 20, 21 and 22 weeks. One woman with twin pregnancy in the bedrest group, PTB at 34 w | + | ? | - |

C; control, CI; confidence interval, CL; cervical length, I; intervention, OI; ovulation induction, OR; odds ratio, PO; primary outcome, PPROM, preterm prelabor rupture of the membranes, PTB; preterm birth, RR; risk ratio, SO; secondary outcome, TVS; transvaginal scan, UK; United Kingdom, w; week

Prevention of preterm birth

* + No or minor problems

? Some problems

- Major problems

STable 4.2.2.a. Intervention cerclage

Outcome variable: Any preterm birth before 35 gestational weeks

| **Author, year Country**  **Trial acronym** | **Singletons/ Twins/ Triplets** | **Risk factor** | **Number**  **of randomized patients n =** | **Results** | | **Comments Risk factor** | **Directness *** | **Study limitations *** | **Precision *** |
| --- | --- | --- | --- | --- | --- | --- | --- | --- | --- |
|  |  |  |  | **Intervention** | **Control** |  |  |  |  |

| Lazar, 1984 France | Singletons | Composite score of a combination of: History of PTB  29-36 w, history of previous miscarriage, prior threatening PTL treated by hospitalization, uterine malformation, previous forced cervical dilatation, low lying placenta with bleeding, CL <2 cm,  cx open for inner os | I: 268 C:238 | McDonald cerclage 10/268 (3.7%)  No statistics | No cerclage 10/238 (4.2%) | PO not defined | ? | ? | ? |
| --- | --- | --- | --- | --- | --- | --- | --- | --- | --- |
| Owen, 2009 USA | Singletons | History of sPTB or PPROM and short TVS CL <25 mm | I: 149  C: 153 | McDonald cerclage 47/148 (32%)  OR 0.67, 95% CI  0.42-1.07 p=0.09 | No cerclage 64/153 (42%) | PO | ? | ? | ? |
| Berghella, 2004 USA  (2 centers) | Singletons and twins  (twins 4/61, 7%) | ≥1 of high-risk factors for preterm birth (≥1 preterm birth <35  w, ≥2 curettages, diethylstilbestrol exposure, cone biopsy, Mullerian anomaly,  or twin pregnancy) and/or TVS CL < 25 mm or  significant funneling | I: 31  C: 30 | McDonald cerclage + bedrest  All 14/31 (45%)  RR 0.94  (95% CI 0.34-2.58)  Singletons 11/28 (39.3%)  No statistics Twins  3/3 (100%)  No statistics | No cerclage, bedrest All 14/30 (47%)  Singletons 13/29 (44.8%)  Twins 1/1 (100%) | PO  3 women with twin pregnancies in the intervention group, all early PTB at 20, 21 and 22 weeks.  One woman with twin pregnancy in the bedrest group, PTB at 34 w | + | ? | - |

C; control, CI; confidence interval, CL; cervical length, I; intervention, OR; odds ratio, PO; primary outcome, PPROM; preterm prelabor rupture of the membranes, PTB; preterm birth, RR; risk ratio, TVS; transvaginal scan, w; weeks

| **Author, year Country**  **Trial acronym** | **Singletons/ Twins/ Triplets** | **Risk factor** | **Number**  **of randomized patients**  **n=** | **Results** | | **Comments Risk factor** | **Directness *** | **Study limitations *** | **Precision *** |
| --- | --- | --- | --- | --- | --- | --- | --- | --- | --- |
|  |  |  |  | **Intervention** | **Control** |  |  |  |  |

| Althuisius, 2001 The Netherlands | Singletons | Previous PTB <34 w, PPROM<32 w or uterine anomaly, or prior cold knife  conization and TVS CL <25 mm at <27 w | I: 19 C:16 | McDonald cerclage + bedrest  0/19 (0%) p=0.002 | No cerclage, bedrest 7/16 (43.8%) | One of 3 POs (together with neonatal  morbidity/mortality and neonatal survival) | + | - | - |
| --- | --- | --- | --- | --- | --- | --- | --- | --- | --- |
| Otsuki, 2016 Japan  60 tertiary centers | Singletons | TVS CL  <25 mm | I1  (Shirodkar): 35 I2  (McDonald): 36  C: 35 | Shirodkar cerclage 1/34 (2.9%) p=0.34  RR not presented McDonald cerclage 6/34 (17.6%), p=0.78  RR not presented | No cerclage (bedrest) 4/33 (12.1%) | Not PO | + | ? | - |
| Berghella, 2004 USA  (2 centers) | Singletons and twins (4/61, 7% twins) | ≥1 of high-risk factors for PTB (≥1 PTB <35 w, ≥2  curettages, diethylstilbestrol exposure, cone biopsy, Mullerian anomaly,  or twin pregnancy) and or TVS CL < 25 mm or significant funneling | I: 31  C: 30 | McDonald cerclage + bedrest  All 13/31 (42%)  RR 1.05 (95% CI 0.57-  1.92)  Singletons 10/28 (35.7%)  No statistics Twins  3/3 (100%)  No statistics | Bedrest  All 12/30 (40%)  Singletons 12/29 (41.4%)  Twins 0/1 (0%) | Not PO  3 women with twin pregnancies in the intervention group, all early PTB at 20, 21 and 22 weeks. One woman with twin pregnancy in the bedrest group, PTB at 34 w | + | ? | - |
| Rust, 2000 USA | Singletons and twins/triplets Singletons (n=54) and 5 sets of twins and 2 sets of triplets (11%  multifetal pregnancies) | History of PTB, second trimester pregnancy loss, previous cervical surgery, uterine anomaly, multiple gestation  and a  TVS CL <25 mm or dilated internal os | I:31 C:30 | McDonald cerclage + modified bedrest  12/31 (38.7%) p=0.6  RR not presented | No cerclage, modified bedrest  9/30 (30.0%) | No PO. 5 twin and 2 triplets evenly distributed between the two groups, though not defined in the outcomes. Not included in meta-analysis due to multifetal pregnancies >10%.  Rust 2001 not included due to wrong intervention (risk factor analysis). Rust 2001 included an expanded study population from Rust 2000,  I:55, C: 58 | - | - | ? |

| **Author, year Country**  **Trial acronym** | **Singletons/ Twins/ Triplets** | **Risk factor** | **Number**  **of randomized patients**  **n=** | **Results** | | **Comments Risk factor** | **Directness *** | **Study limitations *** | **Precision *** |
| --- | --- | --- | --- | --- | --- | --- | --- | --- | --- |
|  |  |  |  | **Intervention** | **Control** |  |  |  |  |

| **Systematic reviews**  (only articles with results not shown in original articles are included here) Assessment of Directness, Study limitations and Precision refer to the original articles | | | | | | | | | |
| --- | --- | --- | --- | --- | --- | --- | --- | --- | --- |
| **Alfirevic 2017** | **Singletons** | | | | | | | | |
| Ezechi, 2004 Nigeria | Not stated | Previous PTB; 1 (63-69%),  2 (21-32%),  3 (5-9%) | I:39 C:42 | McDonald cerclage 0/39 (0%)  No statistics | No cerclage 11/42 (26.2%) | PO not defined  NB in original article 38 resp 43 | - | ? | ? |
| Macnaughton MRC/RCOG, 1993  UK, France, Hungary, Norway, Italy, Belgium, Zimbabwe, South  Africa, Iceland, Ireland, the Netherlands, Canada | Singletons (subanalysis from original paper) | Included if uncertainty if cerclage or not for risk patients: Previous PTB, previous second trimester miscarriage, previous early abortion, cervical  amputation, cone biopsy, twin, uterine anomaly | I: 635  C: 629  (singletons) | Cerclage  (not prespecified) 92/635 (14.5%)*  No statistics | No cerclage 113/629 (18.0%)* | Not PO  *Includes all miscarriages in numerator and denominator According to Alfirevic, Cochrane 2017:  Miscarriages in singletons I: 37/635, C: 42/629 | ? | ? | ? |
| Owen, 2009 USA | Singletons | History of sPTB <34 w  or PPROM + TVS CL <25 mm | I: 149  C: 153 | McDonald cerclage 42/148 (28.4%)  No statistics | No cerclage 57/153 (37.3%) | Not PO | ? | ? | ? |
| Rush, 1984 South Africa | Singletons | History of previous late miscarriage or PTB out of at least one spontaneous between 14-36 w and 2,3, or 4 previous pregnancies ending  spontaneously before 37 w | I: 96  C: 98 | McDonald cerclage 14/96  No statistics | No cerclage 14/98 | Not PO | ? | ? | ? |
| To, 2004 UK  (6 countries; UK, Brazil, South Africa,  Slovenia, Greece, Chile; 12 hospitals) | Singletons | TVS CL ≤15 mm Previous cervical surgery: I: 6%  C: 7% | I: 127  C: 126 | Shirodcar cerclage 28/127 (22.0%)  No statistics | No cerclage 36/126 (28.6%) | Not PO | ? | ? | ? |

C; control, CI; confidence interval, CL; cervical length, w; gestational week, I; intervention, PPROM; preterm prelabor rupture of membranes, PTB; preterm birth, RR; risk ratio, PO; primary outcome, TVS; transvaginal scan, w; weeks

| **Author, year Country**  **Trial acronym** | **Singletons/ Twins/ Triplets** | **Risk factor** | **Number of**  **randomized patients**  **n=** | **Results** | | **Comments Risk factor** | **Directness *** | **Study limitations *** | **Precision *** |
| --- | --- | --- | --- | --- | --- | --- | --- | --- | --- |
|  |  |  |  | **Intervention** | **Control** |  |  |  |  |

| Roman, 2020  Multicenter (8 centers, Italy, US, Spain, Poland, Denmark, Switzerland. | Twins DA | Asymptomatic women with twin pregnancy, with dilated cervix 1-5 cm identified by pelvic examination and/or speculum examination and/or TVS | I: 17 C:13 | Physical examination indicated McDonald cerclage  12/17 (70.6%)  RR 0.71 (95% CI 0.52-0.96) p=0.05 | No cerclage 13/13 (100%) | PO | + | ? | - |
| --- | --- | --- | --- | --- | --- | --- | --- | --- | --- |

C; control, CI; confidence interval, DA; diamniotic, I; intervention, PO; primary outcome, RR; risk ratio, US; United States of America, TVS; transvaginal scan

| **Author, year Country**  **Trial acronym** | **Singletons/ Twins/ Triplets** | **Risk factor** | **Number of**  **randomized patients**  **n=** | **Results** | | **Comments Risk factor** | **Directness *** | **Study limitations *** | **Precision *** |
| --- | --- | --- | --- | --- | --- | --- | --- | --- | --- |
|  |  |  |  | **Intervention** | **Control** |  |  |  |  |

| To, 2004 UK  (6 countries; UK, Brazil, South Africa, Slovenia,  Greece, Chile; 12 hospitals) | Singletons | TVS CL ≤15 mm.  Previous cervical surgery:  I: 6%  C: 7% | I: 127  C: 126 | Shirodkar cerclage 28/127 (22%)  RR 0.84, 95% CI 0.54-1.31 p=0.44 | No cerclage 33/126 (26%) | PO | ? | ? | ? |
| --- | --- | --- | --- | --- | --- | --- | --- | --- | --- |
| Macnaughton MRC/RCOG, 1993  UK, France, Hungary, Norway, Italy, Belgium, Zimbabwe, South Africa, Iceland, Ireland, the Netherlands, Canada | Singletons and twins (2%)  (Twins I:12 C:16) | Included if uncertainty if cerclage or not for risk patients: Previous PTB, previous second trimester miscarriage, previous early abortion, cervical amputation, cone biopsy, uterine anomaly, twin pregnancy | I: 647  C: 645 | Cerclage (not prespecified) Singletons and twins 83/647 (13%)*  OR 0.72 (95% CI 0.53-0.97) p=0.03  Sensitivity analysis excluding early miscarriage (<13 w, n=12) in numerator and denominator  71/635 (11%)  OR 0.67 (95% CI 0.49-0.92) p=0.015  Singletons (calculated)  82/635 (12.9%)*  No statistics  Twins  1/12 (8.3%)*, NS | No cerclage Singletons and twins 110/645 (17%)*  Sensitivity analysis excluding early miscarriage (<13 w, n=9) in numerator and denominator  101/636 (16%)  Singletons (calculated)  105/629 (16.7%)*  Twins  5/16 (31.3%)* | PO (together with <37 w, changed to  < 33 w after study initiation) All miscarriages (I: 43/647 and C: 50/645) included in numerator and  denominator. In article also presented without early miscarriage (<13 w) for singletons and twins combined | ? | ? | ? |
| Dor, 1982  Israel | Twins | Twin pregnancies after OI | I: 22/44 C: 23/46 | Mc Donald cerclage 3/22 (13.6%)  No statistics | No cerclage 3/23 (13.1%) | Not PO From Figure 1 | ? | - | - |

C; control, CI; confidence interval, CL; cervical length, I; intervention, OI; ovulation induction, PO; primary outcome, PTB; preterm birth, RR; risk ratio, TVS; transvaginal scan, UK; United Kingdom, w; weeks.

| **Author, year Country**  **Trial acronym** | **Singletons/ Twins/ Triplets** | **Risk factor** | **Number**  **of randomized patients**  **n=** | **Results** | | **Comments Risk factor** | **Directness *** | **Study limitations *** | **Precision *** |
| --- | --- | --- | --- | --- | --- | --- | --- | --- | --- |
|  |  |  |  | **Intervention** | **Control** |  |  |  |  |

| Lazar 1984, France | Singletons | Composite score of a combination of: History of PTB 29-36 w, history of previous miscarriage, prior threatening PTL treated by hospitalization, uterine malformation, previous forced cervical dilatation, low lying placenta with bleeding, CL  <2 cm, cx open for inner os | I: 268 C:238 | McDonald cerclage 4/268 (1.5%)  No p value or RR presented | No cerclage 1/238 (0.4%) | Primary outcome not defined | ? | ? | ? |
| --- | --- | --- | --- | --- | --- | --- | --- | --- | --- |
| Otsuki 2016 Japan  60 tertiary centers | Singletons | TVS CL  <25 mm | I1  (Shirodkar): 35 I2  (McDonald): 36  C: 35 | Shirodkar cerclage: 1/34 (2.9%) p=0.79  RR not presented McDonald cerclage: 6/34 (17.6%) p=0.90  RR not presented | No cerclage, bedrest 4/33 (12.1%) | Not PO | ? | ? | ? |
| Berghella 2004 USA  (2 centers) | Singletons and twins (4/61, 7%  twins) | ≥1 of high-risk factors for PTB (≥1 PTB <35 w, ≥2 curettages, diethylstilbestrol exposure, cone biopsy, Mullerian anomaly, or twin pregnancy) and/or TVS CL <25 mm or significant funneling | I: 31  C: 30 | McDonald cerclage+ bedrest All 11/31 (35%)  RR 0.97 (95% CI 0.50-1.89)  Singletons 8/28 (28.6%) No statistics  Twins 3/3 (100%) No statistics | Bedrest  All 11/30 (37%)  Singletons 12/29 (41.4%)  Twins 0/1 (0%) | Not PO  3 women with twin pregnancies in the intervention group, all early PTB at 20, 21 and 22 weeks.  One woman with twin  pregnancy in the bedrest group, PTB at 34 w | ? | ? | ? |

C; control, CI; confidence interval, CL; cervical length, I; intervention, RR; risk ratio, PO; primary outcome, PTB; preterm birth, TVS; transvaginal scan, w, weeks

| **Author, year Country**  **Trial acronym** | **Singletons/ Twins/ Triplets** | **Risk factor** | **Number of**  **randomized patients**  **n=** | **Results** | | **Comments Risk factor** | **Directness *** | **Study limitations *** | **Precision *** |
| --- | --- | --- | --- | --- | --- | --- | --- | --- | --- |
|  |  |  |  | **Intervention** | **Control** |  |  |  |  |

| Roman, 2020  Multicenter (8 centers, Italy, US, Spain, Poland, Denmark, Switzerland) | Twins DA | Asymptomatic women with twin pregnancy, with dilated cervix 1-5 cm identified by pelvic examination and/or speculum examination and/or TVS | I: 17 C:13 | Physical examination indicated McDonald cerclage 11/17 (64.7%)  RR 0.65 (95% CI 0.46-0.92) p=0.02 | No cerclage 13/13 (100%) | Not PO | + | + | - |
| --- | --- | --- | --- | --- | --- | --- | --- | --- | --- |

C; control, CI; confidence interval, DA; diamniotic, I; intervention, RR; risk ratio, PO; primary outcome, US; United States of America, TVS; transvaginal scan

| **Author, year Country**  **Trial acronym** | **Singletons/ Twins/ Triplets** | **Risk factor** | **Number of**  **randomized patients**  **n=** | **Results** | | **Comments Risk factor** | **Directness *** | **Study limitations *** | **Precision *** |
| --- | --- | --- | --- | --- | --- | --- | --- | --- | --- |
|  |  |  |  | **Intervention** | **Control** |  |  |  |  |

| Otsuki, 2016 Japan  60 tertiary centers | Singletons | TVS CL  <25 mm | I1 (Shirodkar): 35  I2 (McDonald): 36  C: 35 | Shirodkar cerclage: 0/34 (0%)  NS  McDonald cerclage 0/34 (0%)  NS | No cerclage, bedrest 0/33 (0%) | Not PO | ? | ? | ? |
| --- | --- | --- | --- | --- | --- | --- | --- | --- | --- |
| Berghella, 2004 USA  (2 centers) | Singletons and  Twins (4/61, 7% twins) | ≥1 of high-risk factors for preterm birth (≥1 preterm birth <35w,  ≥2 curettages, diethylstilbestrol exposure, cone biopsy, Mullerian anomaly, or twin pregnancy), and/or TVS CL <25 mm or significant funneling | I: 31  C: 30 | McDonald cerclage + bedrest  9/31 (29%)  RR 1.45 (95% CI 0.59-  3.58)  Singletons 6/28 (21.4%)  No statistics Twins  3/3 (100%)  No statistics | Bedrest 6/30 (20%)  Singletons 6/29 (20.7%)  Twins 0/1 (0%) | Not PO  3 women with twin pregnancies in the intervention group, all early PTB at 20, 21 and 22 weeks.  One woman with twin pregnancy in the bedrest group, PTB at 34 w | ? | ? | ? |
| Rust, 2000 USA | Singletons and twins 11%  multifetal pregnancies | History of PTB, second trimester pregnancy loss, previous cervical surgery, uterine anomaly, multifetal pregnancy and a TVS CL <25 mm or dilated internal os | I: 31  C: 30 | McDonald cerclage and modified bedrest  7/31 (22.6%) p=0.5  RR not presented | Modified bedrest 4/30 (13.3%) | Not PO  5 twin and 2 triplets evenly distributed between the two groups, though not defined in the outcomes  Not included in meta- analysis due to multifetal pregnancies>10% | - | - | ? |
| Dor 1982, Israel | Twins | Twin pregnancies after OI | I: 22/44 C: 23/46 | Cerclage 1/22 (4.5%)  No statistics | No cerclage 2/23 (8.7%) | Not PO From Figure 1 | ? | - | - |

| **Author, year Country**  **Trial acronym** | **Singletons/ Twins/ Triplets** | **Risk factor** | **Number of**  **randomized patients**  **n=** | **Results** | | **Comments Risk factor** | **Directness *** | **Study limitations *** | **Precision *** |
| --- | --- | --- | --- | --- | --- | --- | --- | --- | --- |
|  |  |  |  | **Intervention** | **Control** |  |  |  |  |

| **Systematic reviews**  (only articles with results not shown in original articles are included here)  Assessment of Directness, Study limitations and Precision refer to the original articles and not to the subgroups presented below | | | | | | | | | |
| --- | --- | --- | --- | --- | --- | --- | --- | --- | --- |
| **Alfirevic 2017** | **Singletons** | | | | | | | | |
| Ezechi, 2004 Nigeria | Not stated | Previous PTB: 1 (63-69%), 2 (21-  32%), 3 (5-9%) | I:39 C:42 | McDonald cerclage 0/39 (0%)  No statistics | No cerclage 1/42 (2.4%) | PO not defined  NB in original article I:38 and C:43 | - | ? | ? |
| Macnaughton MRC/RCOG, 1993  UK, France, Hungary, Norway, Italy, Belgium, Zimbabwe, South Africa, Iceland, Ireland, the Netherlands, Canada | Singletons | Included if uncertainty if cerclage or not for risk patients: Previous PTB, previous second trimester miscarriage, previous early abortion, cervical amputation, cone biopsy, uterine anomaly, twin pregnancy | I: 635  C: 629  (singletons) | Cerclage  (not prespecified) 53/635 (8.3%)*  No statistics | No cerclage 65/629 (10.3%)* | No PO  *All miscarriages included in numerator and denominator  According to Alfirevic, Cochrane 2017:  Miscarriage in singletons I: 37/635, C: 42/629 | ? | ? | ? |
| Owen, 2009 USA | Singletons | History of sPTB <34 w or PPROM  + short TVS CL <25 mm | I: 149  C: 153 | McDonald cerclage 21/148 (14.2%)  No statistics | No cerclage 33/153 (21.6%) | Not PO | ? | ? | ? |
| Rush, 1984 South Africa | Singletons | History of previous late miscarriage or PTB out of at least one spontaneous between 14-36 w and 2,3, or 4 previous pregnancies  ending spontaneously before 37 w | I: 96  C: 98 | McDonald cerclage 7/96 (7.3%)  No statistics | No cerclage 7/98 (7.1%) | Not PO | ? | ? | ? |
| To, 2004  UK(6 countries; UK, Brazil, South Africa, Slovenia,  Greece, Chile; 12 hospitals) | Singletons | TVS CL ≤15 mm Previous cervical surgery: I: 6%  C: 7% | I: 127  C: 126 | Shirodcar cerclage 15/127 (11.8%)  No statistics | No cerclage 17/126 (13.5%) | Not PO | ? | ? | ? |

C; control, CL; cervical length, I; intervention, PPROM; preterm premature rupture of the membranes, PTB; preterm birth, RR; risk ratio, PO; primary outcome, sPTB; spontaneous PTB, TVS; transvaginal scan, w; weeks

| **Author, year Country**  **Trial acronym** | **Singletons/ Twins/ Triplets** | **Risk factor** | **Number**  **of randomized patients**  **n=** | **Results** | | **Comments Risk factor** | **Directness *** | **Study limitations *** | **Precision *** |
| --- | --- | --- | --- | --- | --- | --- | --- | --- | --- |
|  |  |  |  | **Intervention** | **Control** |  |  |  |  |

| Roman, 2020 Multicenter  (8 centers, Italy, USA, Spain, Poland, Denmark, Switzerland) | Twins DA | Asymptomatic women with twin pregnancy, with dilated cervix 1-5 cm identified by pelvic examination and/or speculum examination and/or TVS | I: 17 C:13 | Physical examination indicated McDonald cerclage 1/17 (41.2%)  RR 0.49  (95% CI 0.26-0.89) p=0.02 | No cerclage 11/13 (84.6%) | Not PO | + | + | - |
| --- | --- | --- | --- | --- | --- | --- | --- | --- | --- |

C; control, CI; confidence interval, DA; diamniotic, I; intervention, RR; risk ratio, PO; primary outcome, TVS; transvaginal scan

| **Author, year Country**  **Trial acronym** | **Singletons/ Twins/ Triplets** | **Risk factor** | **Number of**  **randomized patients**  **n=** | **Results** | | **Comments Risk factor** | **Directness *** | **Study limitations *** | **Precision *** |
| --- | --- | --- | --- | --- | --- | --- | --- | --- | --- |
|  |  |  |  | **Intervention Mean (SD)** | **Control**  **Mean (SD)** |  |  |  |  |

| Ezechi, 2004, Nigeria | Not stated | Previous PTB | I: 38  C: 43 | McDonald cerclage Mean (SD) 38.9 (2.9) w p=0.001 | No cerclage Mean (SD) 36.9 (3.5) w | Not PO Singletons/twins not stated. | - | ? | ? |
| --- | --- | --- | --- | --- | --- | --- | --- | --- | --- |
| To 2004 UK  (6 countries 12 hospitals) | Singletons | TVS  CL ≤15 mm  Previous cervical surgery: I: 6%  C: 7% | I: 127  C: 126 | Shirodkar cerclage Mean (SE) 36.4 (0.42) w  Difference in means 0.95 (95% CI -0.26 to  2.15) w p=0.12 | No cerclage Mean (SE) 35.4 (0.45)  w | Not PO | ? | ? | ? |
| Berghella 2004 USA  (2 centers) | Singletons and twins (4/61)  (7%) | ≥1 of high-risk factors for preterm birth (≥1 preterm birth <35 w, ≥2 curettages, diethylstilbestrol exposure, cone biopsy, Mullerian anomaly, or twin pregnancy) and/or TVS CL <25 mm or significant funneling | I: 31  C: 30 | McDonald cerclage and bedrest  Singletons and twins Mean (SD) 32.6 (6.9) w p=0.89 | Bedrest Singletons and twins  Mean (SD) 32.9 (6.7) w | Not PO  3 women with twin pregnancies in the intervention group, all early PTB at 20, 21 and 22 weeks. One woman with twin  pregnancy in the bedrest group, PTB at 34 w | + | ? | - |
| Rust 2000 USA | Singletons and twins/triplets (7/61)  (11%) | History of PTB, second trimester pregnancy loss, previous cervical surgery, uterine anomaly, multifetal pregnancy and a TVS CL <25 mm or dilated internal os | I: 31  C: 30 | McDonald cerclage Mean (SD) 33.5 (6.3) w p=0.4 | Bedrest  Mean (SD) 34.7 (4.7)  w | 5 twin and 2 triplets evenly distributed between the two groups, though not defined in the outcomes. | - | - | ? |

C; control, CL; cervical length, I; intervention, PTB; preterm birth, RR; risk ratio, SD; standard deviation, SE; standard error, PO; primary outcome, TVS; transvaginal scan, w; weeks

| **Author, year Country**  **Trial acronym** | **Singletons/ Twins/ Triplets** | **Risk factor** | **Number of**  **randomized patients**  **n=** | **Results** | | **Comments Risk factor** | **Directness *** | **Study limitations *** | **Precision *** |
| --- | --- | --- | --- | --- | --- | --- | --- | --- | --- |
|  |  |  |  | **Intervention** | **Control** |  |  |  |  |

| Ezechi, 2004 Nigeria | Not stated | Previous PTB; 1 (63-69%), 2  (21-32%), 3 (5-9%) | I: 38  C: 43 | McDonald cerclage 3/38 (7.9%)  OR 0.22 (95% CI  0.00-0.96) p=0.04 | No cerclage 12/43 (27.9%) | Not PO Singletons/twins not stated.  Singletons/twins not stated but singletons correspond to neonatal outcomes | - | ? | ? |
| --- | --- | --- | --- | --- | --- | --- | --- | --- | --- |
| Rush, 1984 South Africa | Singletons | History of previous late miscarriage or PTB out of at least one spontaneous between 14-36 w and  2,3, or 4 previous pregnancies ending spontaneously before 37 w | I: 96  C: 98 | McDonald cerclage 36/96 (38%)  No statistics | No cerclage 34/98 (35%) | Not PO | ? | ? | ? |
| Macnaughton MRC/RCOG, 1993  UK, France, Hungary, Norway, Italy, Belgium, Zimbabwe, South Africa, Iceland, Ireland, the Netherlands, Canada | Singletons and twins Twins 28/1292 (2%)  Twins: I: 12  C: 16 | Included if uncertainty if cerclage or not for risk patients: Previous PTB, previous second trimester miscarriage, previous early abortion, cervical amputation, cone biopsy, uterine anomaly, twin pregnancy | I: 647/659 C: 645/661 | Cerclage (not specified)  Singletons and twins 154/659 (23.4%)*  No statistics | No cerclage  Singletons and twins 174/661 (26.3%)* | Not PO  2% twins in intervention group, 2.5% twins in no cerclage group  This mix rendered the minus for directness  *Miscarriages included in  numerator and denominator (I: 43 and C: 50) | ? | ? | ? |

C; control, CI; confidence interval, I; intervention, OR; odds ratio, PO; primary outcome, PTB; preterm birth, RR; risk ratio, PO; primary outcome, UK; United Kingdom, w; weeks

| **Author, year Country**  **Trial acronym** | **Singletons/ Twins/ Triplets** | **Risk factor** | **Number of**  **randomized patients**  **n=** | **Results** | | **Comments Risk factor** | **Directness *** | **Study limitations *** | **Precision *** |
| --- | --- | --- | --- | --- | --- | --- | --- | --- | --- |
|  |  |  |  | **Intervention** | **Control** |  |  |  |  |

| Macnaughton MRC/RCOG, 1993  UK, France, Hungary, Norway, Italy, Belgium, Zimbabwe, South Africa, Iceland, Ireland, the Netherlands, Canada | Singletons and twins 28/1292 (2%)  Twins: I: 12  C: 16 | Included if uncertainty if cerclage or not for risk patients: Previous PTB, previous second trimester miscarriage, previous early abortion, cervical amputation, cone biopsy, uterine anomaly, twin pregnancy | I: 647/659 C: 645/661 | Cerclage  (not specified)  Singletons and twins 63/659 (10%)*  OR 0.70 (95% CI 0.50-0.99)  No p value | No cerclage  Singletons and twins 86/661 (13%)* | Not PO  2% twins in intervention group, 2.5% twins in no cerclage group  This mix rendered the minus for directness  *Miscarriages included in numerator and denominator (I: 43 and C: 50) | - | ? | ? |
| --- | --- | --- | --- | --- | --- | --- | --- | --- | --- |
| Roman, 2020  Multicenter (8 centers, Italy, US, Spain, Poland, Denmark, Switzerland. | Twins DA | Asymptomatic women with twins, dilated cervix 1-5 cm by pelvic examination and/or speculum examination and/or TVS | I: 17/34 C:13/26 | Physical examination indicated McDonald cerclage 21/34 (61.7%)  RR 0.67 (95% CI 0.50-0.89) p=0.007 | No cerclage 24/26 (92.3) | Not PO | + | ? | - |

C; control, CI; confidence interval, CL; cervical length, DA; diamniotic, I; intervention, OR; odds ratio, PO; primary outcome PTB; preterm birth, RR; risk ratio, TVS; transvaginal ultrasound, UK; United Kingdom

| **Author, year Country**  **Trial acronym** | **Singletons/ Twins/ Triplets** | **Risk factor** | **Number of randomize d patients n=** | **Results** | | **Comments Risk factor** | **Directness *** | **Study limitations** | **Precision *** |
| --- | --- | --- | --- | --- | --- | --- | --- | --- | --- |
|  |  |  |  | **Intervention** | **Control** |  |  |  |  |

| Althuisius, 2001 The Netherlands | Singleton | History of PTB <34 w, PPROM <32 w, cold knife conization, diethylstilbestrol exposure, or uterine anomaly  and TVS CL <25 mm < 27 w | I: 19 C:16 | McDonald cerclage, bedrest 0/19 (0%)  p=0.002 | No cerclage, bedrest 3/16 (18.8%) | One of 3 POs (together with neonatal  morbidity/mortality and PTB  <34 w)  Defined as neonatal survival in the article, translated as perinatal mortality | + | - | - |
| --- | --- | --- | --- | --- | --- | --- | --- | --- | --- |
| Ezechi, 2004 Nigeria | Not stated | Previous PTB | I: 38  C: 43 | McDonald cerclage 0/38 (0%)  OR 0.00, 95% CI 0.0-4.68 p=0.53 | No cerclage 2/43 (6.1%) | Not PO Singletons/twins not stated.  Singletons/twins not stated but singletons correspond to  neonatal outcomes | - | ? | ? |
| Lazar, 1984 France | Singletons | Composite score of a combination of: History of PTB 29-36 w, history of previous miscarriage, prior threatening PTL treated by hospitalization, uterine malformation, previous forced cervical dilatation, low lying placenta with bleeding, CL <2 cm, cx open for inner  os | I: 268 C:238 | McDonald cerclage 2/268 (0.7%)  No statistics | No cerclage 1/238 (0.4%) | Not PO  Perinatal mortality not defined | ? | ? | ? |
| Owen, 2009 USA | Singletons | History of sPTB + short TVS CL <25 mm | I: 149  C: 153 | McDonald cerclage 13/148 (8.8%) p=0.046 | No cerclage 25/153 (16%) | Not PO  One person in the cerclage group lost to follow up  Defined as stillbirth or neonatal death prior to discharge | ? | ? | ? |
| To, 2004 UK  (6 countries; UK, Brazil, South Africa, Slovenia, Greece,  Chile; 12 hospitals) | Singletons | TVS CL ≤15 mm Previous cervical surgery: I: 6%  C: 7% | I: 127  C: 126 | Shirodkar cerclage 7/127 (6%)  RR 0.69, 95% CI 0.27-1.77 p=0.44 | No cerclage 10/126 (8%) | Not PO  Perinatal death not defined in days | ? | ? | ? |

| **Author, year Country**  **Trial acronym** | **Singletons/ Twins/ Triplets** | **Risk factor** | **Number of randomize d patients n=** | **Results** | | **Comments Risk factor** | **Directness *** | **Study limitations** | **Precision *** |
| --- | --- | --- | --- | --- | --- | --- | --- | --- | --- |
|  |  |  |  | **Intervention** | **Control** |  |  |  |  |

| Macnaughton MRC/RCOG, 1993  UK, France, Hungary, Norway, Italy, Belgium, Zimbabwe, South Africa, Iceland, Ireland, the Netherlands, Canada | Singletons and twins (28 sets of  twins, 2.2% I: 12  C: 16 | Included if uncertainty if cerclage or not for risk patients: Previous PTB, previous second trimester miscarriage, previous early abortion, cervical amputation, cone biopsy, uterine anomaly, twin pregnancy | I: 647  (659 fetuses)  C: 645  (661 fetuses) | Cerclage (not prespecified) Singletons and twins 55/659 (1.8%)*  NS  Singletons (calculated) 53/635 (8.3%)*  No statistics Twins  2/24 (8.3%)*  Fetal level as denominator | No cerclage Singletons and twins  68/661 (2.7%)*  Singletons (calculated) 66/629 (10.4%)*  No statistics Twins  2/32 (6.3%)*  Fetal level as denominator | Not PO  “Perinatal mortality” is the sum of all miscarriages, stillbirth and neonatal mortality, (neonatal mortality not defined in days) same as presented as all perinatal losses for singletons in Alfirevic, Cochrane, 2017  *Numerator and denominator include all miscarriages. | ? | ? | ? |
| --- | --- | --- | --- | --- | --- | --- | --- | --- | --- |
| Roman, 2020  Multicenter (8 centers, Italy, USA, Spain, Poland, Denmark, Switzerland) | Twins DA | Asymptomatic women with twins, and dilated cervix 1-5 cm by pelvic examination and/or speculum examination  and/or TVS | I: 17 C:13 | Physical examination indicated McDonald cerclage 6/34 (17.6%)  RR 0.23, 95% CI 0.1-0.49 p<0.001  (fetal level denominator) | No cerclage  20/26 (76.9%)  (fetal level denominator) | Not PO  Perinatal death not defined in days postpartum  No case of fetal demise (i.e.  PNM= NNM) | + | + | - |

| **Author, year Country**  **Trial acronym** | **Singletons/ Twins/ Triplets** | **Risk factor** | **Number of randomize d patients n=** | **Results** | | **Comments Risk factor** | **Directness *** | **Study limitations** | **Precision *** |
| --- | --- | --- | --- | --- | --- | --- | --- | --- | --- |
|  |  |  |  | **Intervention** | **Control** |  |  |  |  |

| **Systematic reviews**  (only articles with results not shown in original articles are included here) Assessment of Directness, Study limitations and Precision refer to the original articles | | | | | | | | | |
| --- | --- | --- | --- | --- | --- | --- | --- | --- | --- |
| **Alfirevic, 2017 Cochrane** | **Singletons** | | | | | | | | |
| Rush, 1984 South Africa | Singletons | History of previous late miscarriage or PTB out of at least one spontaneous between 14-36 w and 2, 3, or 4 previous pregnancies ending  spontaneously before 37 w | I: 96  C: 98 | McDonald cerclage 9/96  No statistics | No cerclage 9/98 | Not PO | ? | ? | ? |
| Berghella, 2004 USA  (2 centers) | Singletons | ≥1 of high-risk factors for preterm birth (≥1 preterm birth <35  w, ≥2 curettages, diethylstilbestrol exposure, cone biopsy, Mullerian anomaly,  or twin pregnancy)  and/or TVS CL <25 mm or significant funneling | I: 28  C: 29 | McDonald cerclage and bedrest  4/28 (14.3%) | No cerclage, bedrest  4/29 (13.8%) | Not PO  Outcome defined as neonatal survival, no definition in days. No stillbirths.  Cochrane data not corresponding to original article where it should be 3/28 after removing twins. | + | ? | - |

C; control, CI; confidence interval, CL; cervical length, DA; diamniotic, I; intervention, NS; non significant, PO; primary outcome, RR; risk ratio, TVS; transvaginal scan, UK; United Kingdom

| **Author, year Country**  **Trial acronym** | **Singletons/ Twins/ Triplets** | **Risk factor** | **Number of**  **randomized patients**  **n=** | **Results** | | **Comments Risk factor** | **Directness *** | **Study limitations *** | **Precision *** |
| --- | --- | --- | --- | --- | --- | --- | --- | --- | --- |
|  |  |  |  | **Intervention** | **Control** |  |  |  |  |

| Dor, 1982  Israel | Twins | Twin pregnancies after OI | I: 22/44 C: 23/46 | 8/44 (18.2%)  No statistics | No cerclage 7/46 (15.2%) | Not PO  Outcome defined as death within first week of life | ? | - | - |
| --- | --- | --- | --- | --- | --- | --- | --- | --- | --- |

C; control, CI; confidence interval, I; intervention, OI; ovulation induction, PO; primary outcome, RR; risk ratio

| **Author, year Country**  **Trial acronym** | **Singletons/**  **Twins/**  **Triplets** | **Risk factor** | **Number**  **of randomized patients**  **n=** | **Results** | | **Comments Risk factor** | **Directness *** | **Study limitations *** | **Precision *** |
| --- | --- | --- | --- | --- | --- | --- | --- | --- | --- |
|  |  |  |  | **Intervention** | **Control** |  |  |  |  |

| Otsuki, 2016 Japan | Singletons | TVS CL  <25 mm | I1 (Shirodkar): 35  I2 (McDonald): 36  C: 35 | I1: Shirodkar cerclage 0/34 (0%), p=0.56  12: McDonald cerclage  1/34 (2.6%), p=0.99  RR not presented | No cerclage, bedrest 1/33 (5.7%) | Not PO | ? | ? | ? |
| --- | --- | --- | --- | --- | --- | --- | --- | --- | --- |
| To, 2004 UK  (6 countries; UK, Brazil, South Africa,  Slovenia, Greece, Chile; 12 hospitals) | Singletons | TVS C  ≤15 mm Previous cervical  surgery: I: 6%  C: 7% | I: 127  C: 126 | Shirodkar cerclage 4/127 (3%) | No cerclage 5/126 (4%) | Not PO  Neonatal mortality from table 2 PNM - stillbirths and text page 1851. No definition of perinatal mortality in days | ? | ? | ? |
| Berghella, 2004 USA  (2 centers) | Singletons and twins (4 sets of  twins, 7%) | ≥1 of high-risk factors for PTB (≥1 PTB <35  w, ≥2 curettages, diethylstilbestrol exposure, cone biopsy, Mullerian anomaly,  or twin pregnancy) and/or TVS CL < 25 mm or  significant funneling | I: 31 (34 fetuses)  C: 30  (31 fetuses) | McDonald cerclage and bedrest  All 9/34 (26%) p=0.22  Singletons 3/28 (10.7%)  Twins 6/6 (100%) | No cerclage, bedrest All 4/31 (13%)  Singletons 4/29 (13.8%)  Twins 0/2 (0%) | Not PO  3 sets of twins (n=6) in intervention group, 1 set of twins (n=2) in the control group  Outcome defined as neonatal survival, no definition in days | + | ? | - |
| Macnaughton MRC/RCOG, 1993  multicenter UK, France, Hungary, Norway, Italy, Belgium, Zimbabwe, South Africa, Iceland, Ireland, the Netherlands, Canada | Singletons and twins (28 sets of  twins, 2.2% I: 12  C: 16 | Included if uncertainty if cerclage or not for risk patients: Previous PTB, previous second trimester miscarriage, previous early abortion, cervical amputation, cone biopsy, uterine anomaly, twin pregnancy | I: 647  (659 fetuses)  C: 645  (661 fetuses) | Cerclage (not prespecified) Singletons and twins 8/659 (1.2%)*, NS  Fetal level as denominator | No cerclage Singletons and twins 14/661 (2.1%)*  Fetal level as denominator | No discrimination about if neonatal deaths included twins or not  Outcome defined as “liveborn died”, no definition in days  *Denominator includes all miscarriages, numerator “liveborn died” | ? | ? | ? |

| **Author, year Country**  **Trial acronym** | **Singletons/**  **Twins/**  **Triplets** | **Risk factor** | **Number**  **of randomized patients**  **n=** | **Results** | | **Comments Risk factor** | **Directness *** | **Study limitations *** | **Precision *** |
| --- | --- | --- | --- | --- | --- | --- | --- | --- | --- |
|  |  |  |  | **Intervention** | **Control** |  |  |  |  |

| Rust, 2000 USA | Singleton and twins: Singletons (n=54)  and 5 sets of twins and 2 sets of triplets, 11.5% multifetal pregnancies | History of PTB, second trimester pregnancy loss, previous cervical surgery, uterine anomaly, multifetal pregnancy  and a  TVS CL <25 mm or dilated internal os | I: 31  C: 30 | McDonald cerclage, modified bedrest 4/31 (12.9%), p=0.9  Pregnancy level as denominator | No cerclage, modified bedrest 3/30 (10%)  Pregnancy level as denominator | Not PO  5 sets of twins and 2 sets of triplets evenly distributed between the two groups, though not defined in the outcomes.  Neonatal death defined as death  <28 days after delivery Data reanalyzed without  multifetal pregnancies NS (data not shown)  Not included in meta-analysis due to multifetal pregnancies>10% | - | - | ? |
| --- | --- | --- | --- | --- | --- | --- | --- | --- | --- |
| Roman, 2020  multicenter (8 centers, Italy, USA, Spain, Poland, Denmark, Switzerland) | Twins DA | Asymptomatic women with twins and dilated cervix 1-5 cm by pelvic examination and/or speculum examination and/or TVS | I: 17  C: 13 | Physical examination indicated McDonald cerclage  Both twins died: 2/17 (11.7%)  RR 0.15, 95% CI 0.04-  0.58 p=0.005  1 twin died: 2/17 (11.7%)  RR N/A, p=0.49  Denominator pregnancy level  Fetal level as denominator (calculated): 6/34 (17.6%), No  statistics | No cerclage  Both twins died: 10/13 (76.9%)  1 twin died: 0/13 (0%)  Denominator pregnancy level  Fetal level as denominator (calculated): 20/26 (76.9%) | Not PO  Outcome defined as neonatal survival, no definition in days | + | + | - |

C; control, CI; confidence interval, CL; cervical length, DA; diamniotic, I; intervention, NS; non-significant, PO; primary outcome, RR; risk ratio, TVS; transvaginal scan, UK; United Kingdom, w; weeks

| **Author, year Country**  **Trial acronym** | **Singletons/ Twins/ Triplets** | **Risk factor** | **Number**  **of randomized patients n=** | **Results** | | **Comments Risk factor** | **Directness *** | **Study limitations *** | **Precision *** |
| --- | --- | --- | --- | --- | --- | --- | --- | --- | --- |
|  |  |  |  | **Intervention** | **Control** |  |  |  |  |

| Althuisius, 2001 The Netherlands | Singletons | Previous PTB <34 w, PPROM  <32 w or uterine anomaly, or prior cold knife conization and TVS CL <25 mm at <27 w | I: 19 C:16 | McDonald cerclage + bedrest  1/19 (5.3%)  RR 9.5, 95% CI 1.3-68.1 p=0.005 | Bedrest 8/16 (50%) | Not PO  Composite outcome: admission to NICU and/or neonatal death | + | - | - |
| --- | --- | --- | --- | --- | --- | --- | --- | --- | --- |
| Berghella, 2004 USA  (2 centers) | Singletons and twins (4/61, 7%) | ≥1 of high-risk factors for PTB (≥1 PTB <35  w, ≥2 curettages, diethylstilbestrol exposure, cone biopsy, Mullerian anomaly,  or twin pregnancy) and/or TVS CL  <25 mm or significant funneling | I: 31 (34 fetuses)  C: 30  (31 fetuses) | McDonald cerclage, bedrest  All 7/34 (21%) p=0.80  Singletons 7/28 (25.0%)  (3 singletons died) Twins  0/6  (all twins died) | Bedrest All 8/31 (13%)  Singletons 6/29 (20.7%)  (4 singletons died) Twins  2/2 (100%) | Not PO  3 women with twin pregnancies in the intervention group, all early PTB at 20, 21 and 22 weeks. One woman with twin pregnancy in the bedrest group, PTB at 34 w Defined as RDS, IVH III or IV, NEC or sepsis  Denominator all neonates including deaths | + | ? | - |
| Rust, 2000 USA | Singleton and twins:  Singletons (n=54) and 5 sets of twins and 2 sets of triplets, 11.5% multiple pregnancies | History of PTB, second trimester pregnancy loss, previous cervical surgery, uterine anomaly, multifetal pregnancy and a TVS CL <25 mm or dilated internal os | I:31 C:30 | McDonald cerclage, modified bedrest  3/31 (9.7%) p=0.6  Pregnancy level | Modified bedrest 1/30 (3.3%)  Pregnancy level | 5 twin and 2 triplets evenly distributed between the two groups, though not defined in the outcomes.  Not PO  Serious morbidity defined as mechanical ventilation, RDS, NEC, IVH, sepsis  Data reanalyzed without multifetal pregnancies NS (data not shown)  Not included in meta-analysis due to multifetal pregnancies>10% | - | - | ? |
| Roman, 2020  multicenter (8 centers, Italy, US, Spain, Poland, Denmark, Switzerland. | Twins DA | Asymptomatic women with twins, dilated cervix 1-5 cm by pelvic examination and/or speculum examination and/or TVS | I: 17  (34 fetuses)  C: 13  (26 fetuses) | Physical examination indicated McDonald cerclage  Child level 14/30 (46%)  RR 0.93, 95% CI 0.4-2.2 p=1.0 | No cerclage  Child level 3/6 (50%) | Not PO  Outcomes of neonates born alive. Composite neonatal outcome not defined but probably a summary of other morbidities listed, i.e. RDS,  IVH, NEC, sepsis, and ROP (laser therapy) | + | + | - |

| **Author, year Country**  **Trial acronym** | **Singletons/ Twins/ Triplets** | **Risk factor** | **Number**  **of randomized patients n=** | **Results** | | **Comments Risk factor** | **Directness *** | **Study limitations *** | **Precision *** |
| --- | --- | --- | --- | --- | --- | --- | --- | --- | --- |
|  |  |  |  | **Intervention** | **Control** |  |  |  |  |

| **Systematic reviews**  (only articles with results not shown in original articles are included here) Assessment of Directness, Study limitations and Precision refer to the original articles | | | | | | | | | |
| --- | --- | --- | --- | --- | --- | --- | --- | --- | --- |
| **Alfirevic, 2017 Cochrane** | **Singletons** | | | | | | | | |
| Owen, 2009 USA | Singletons | History of sPTB <34 w or PPROM + short TVS CL <25 mm | I: 149  C: 153 | McDonald cerclage 16/148 (10.8%)  No statistics | No cerclage 18/153 (11.8%) | Not PO  No definition i article or Cochrane | ? | ? | ? |
| To, 2004 UK  (6 countries; UK, Brazil, South Africa, Slovenia,  Greece, Chile; 12 hospitals) | Singletons | TVS CL ≤15 mm Previous cervical surgery: I: 6%  C: 7% | I: 127  C: 126 | Shirodcar cerclage 9/127 (7.1%)  No statistics | No cerclage 7/126 (5.6%) | Not PO  No composite adverse neonatal outcome in article, but positive fetal blood culture, BPD, IVH/PVH, and ROP are reported separately | ? | ? | ? |

BPD; bronchopulmonary dysplasia, Cx; cervix, CL; cervical length, C; control, DA diamniotic, I; intervention, IVH; intraventricular hemorrhage, NEC; necrotizing enterocolitis, NND; neonatal death, NICU; neonatal intensive care unit, NS; not significant, OR; odds ratio, PO; primary outcome, PPROM; preterm prelabor rupture of the membranes, PTB; preterm birth, RDS; respiratory distress syndrome, ROP; retinopathy of prematurity, RR; risk ratio, TVS; transvaginal scan, w; weeks

| **Author, year Country**  **Trial acronym** | **Singletons/ Twins/ Triplets** | **Risk factor** | **Number**  **of randomized patients**  **n=** | **Results** | | **Comments Risk factor** | **Directness *** | **Study limitations *** | **Precision *** |
| --- | --- | --- | --- | --- | --- | --- | --- | --- | --- |
|  |  |  |  | **Intervention** | **Control** |  |  |  |  |

| Roman, 2020 Multicenter  (8 centers, Italy, US, Spain, Poland, Denmark, Switzerland) | | Twins DA | | Asymptomatic women with twins, dilated cervix 1-5 cm by pelvic examination and or speculum examination and/or TVS | I: 17 C:13 | Physical examination indicated McDonald cerclage  Child level 14/30 (46.6%) RR 1.41, 95% CI 0.42-4.6 p=0.67 | No cerclage  Child level 2/6 (33.3%) | Not PO RDS defined as  intubation or CPAP Outcomes of neonates born alive. | + | + | - |
| --- | --- | --- | --- | --- | --- | --- | --- | --- | --- | --- | --- |
| **Systematic reviews**  (only articles with results not shown in original articles are included here)  Assessment of Directness, Study limitations and Precision refer to the original articles and not to the subgroups presented below | | | | | | | | | | | |
| **Alfirevic 2017** | **Singletons** | | | | | | | | | | |
| Althuisius, 2001 Netherlands | Singletons | | Previous PTB <34 w, PPROM<32 w or uterine anomaly, or prior cold knife conization and TVS CL  <25 mm at <27 w | | I: 19 C:16 | McDonald cerclage + bedrest  0/19  No statistics | No cerclage, bedrest 0/16 | Not PO | + | - | - |
| Berghella, 2004 USA  (2 centers) | Singletons | | ≥1 of high-risk factors for PTB (≥1 PTB <35w,  ≥2 curettages, diethylstilbestrol exposure, cone biopsy, Mullerian anomaly, or twin pregnancy)  and/or TVS CL <25 mm or significant funneling | | I: 28  C: 29 | McDonald cerclage + bedrest  Singletons 6/28 (21.4%)  No statistics | No cerclage, bedrest Singletons 6/29 (20.7%) | Not PO | + | ? | - |
| Owen, 2009 USA | Singletons | | History of sPTB <34 w or PPROM + TVS CL <25 mm | | I: 149  C: 153 | McDonald cerclage  13/148 (8.8%)  No statistics | No cerclage 13/152 (8.6%) | Not PO  Control group: 153 in article | ? | ? | ? |
| Rush, 1984 South Africa | Singletons | | History of previous late miscarriage or PTB out of at least one spontaneous between 14-36 w and 2, 3, or 4 previous pregnancies ending spontaneously before 37 w | | I: 96  C: 98 | McDonald cerclage 3/96 (3.1%)  No statistics | No cerclage 1/98 (1.0%) | Not PO | ? | ? | ? |

C; control, CL; cervical length, CPAP; continuous positive airway pressure, I; intervention, PO; primary outcome, PPROM; preterm prelabor rupture of the membranes, PTB; preterm birth, RDS; respiratory distress syndrome, RR; risk ratio, sPTB; spontaneous preterm birth, TVS; transcervical length, w; weeks

| **Author, year Country**  **Trial acronym** | **Singletons/ Twins/ Triplets** | **Risk factor** | **Number**  **of randomized patients n=** | **Results** | | **Comments Risk factor** | **Directness *** | **Study limitations *** | **Precision *** |
| --- | --- | --- | --- | --- | --- | --- | --- | --- | --- |
|  |  |  |  | **Intervention** | **Control** |  |  |  |  |

| To, 2004 UK  (6 countries; UK, Brazil, South Africa, Slovenia, Greece, Chile; 12 hospitals) | Singletons | TVS CL ≤15 mm.  Previous cervical surgery:  I: 6%  C: 7% | I: 127  C: 126 | Shirodkar cerclage 4/123 (3%)  RR 0.98 (95% CI 0.25-3.84) p=1.0 | No cerclage 4/121 (3%) | Not PO | ? | ? | ? |
| --- | --- | --- | --- | --- | --- | --- | --- | --- | --- |

BPD; bronchopulmonary dysplasia, C; control, CL; cervical length, I; intervention, PO; primary outcome, RR; risk ratio, TVS; transvaginal scan, UK; United Kingdom

| **Author, year Country**  **Trial acronym** | **Singletons/ Twins/ Triplets** | **Risk factor** | **Number**  **of randomized patients n=** | **Results** | | **Comments Risk factor** | **Directness *** | **Study limitations *** | **Precision *** |
| --- | --- | --- | --- | --- | --- | --- | --- | --- | --- |
|  |  |  |  | **Intervention** | **Control** |  |  |  |  |

| To, 2004 UK  (6 countries; UK, Brazil, South Africa, Slovenia,  Greece, Chile; 12 hospitals) | Singletons | TVS CL ≤15 mm.  Previous cervical surgery: I: 6%  C: 7% | I: 127  C: 126 | Shirodkar cerclage 1/123 (1%)  RR 0.49, 95% CI 0.05-5.35 p=0.62 | No cerclage 2/121 (2%) | Not PO Outcome of neonates born alive  IVH or PVH  grade 3 and 4 | ? | ? | ? |
| --- | --- | --- | --- | --- | --- | --- | --- | --- | --- |
| Roman, 2020  multicenter (8 centers, Italy, US, Spain, Poland,  Denmark, Switzerland) | Twins DA | Asymptomatic women with twins, dilated cervix 1-5 cm by pelvic examination and or speculum examination and/or TVS | I: 17 C:13 | Physical examination indicated McDonald cerclage Child level  4/30 (13.3%)  RR 0.80, 95% CI 0.1-5.9 p=1.0 | No cerclage  Child level 1/6 (16.6%) | Not PO Outcome of neonates born alive. IVH grade 3 and 4 | + | + | - |
| **Systematic reviews**  (only articles with results not shown in original articles are included here)  Assessment of Directness, Study limitations and Precision refer to the original articles and not to the subgroups presented below | | | | | | | | | |
| **Alfirevic 2017** | **Singletons** | | | | | | | | |
| Althuisius 2001 Netherlands | Singletons | Previous PTB <34 w, PPROM<32 w or uterine  anomaly, or prior cold knife conization together with TVS CL<25 mm at <27 w | I: 19 C:16 | McDonald cerclage + bedrest 0/19 (0%)  No statistics | No cerclage, bedrest  0/16 (0%) | Not PO | + | - | - |
| Owen, 2009 USA | Singletons | History of sPTB <34 w or PPROM + TVS CL <25 mm | I: 149  C: 153 | McDonald cerclage 0/148 (0%)  No statistics | No cerclage 2/152 (1.3%) | Not PO Control group:  153 in article | ? | ? | ? |
| Rush, 1984 South Africa | Singletons | History of previous late miscarriage or PTB out of at least one spontaneous between 14-36 w and 2, 3, or 4 previous pregnancies ending spontaneously before 37 w | I: 96  C: 98 | McDonald cerclage 1/96 (1.0%)  No statistics | No cerclage 1/98 (1.0%) | Not PO | ? | ? | ? |
| Berghella, 2004 USA  (2 centers) | Singletons | ≥1 of high-risk factors for PTB (≥1 PTB <35 w, ≥2 curettages, diethylstilbestrol exposure, cone  biopsy, Mullerian anomaly, or twin pregnancy) and/or TVS CL <25 mm or significant funneling | I: 28  C: 29 | McDonald cerclage + bedrest 2/28 (7.1%)  No statistics | No cerclage, bedrest 1/29 (3.4%) | Not PO | + | ? | - |

C; control, CI; confidence interval, CL; cervical length, I; intervention, IVH; intraventricular hemorrhage, PO; primary outcome, PTB; preterm birth, PVH; periventricular hemorrhage, TVS; transvaginal scan, RR; risk ratio, TVS; transvaginal scan, w; weeks

| **Author, year Country**  **Trial acronym** | **Singletons/ Twins/ Triplets** | **Risk factor** | **Number of**  **randomized patients**  **n=** | **Results** | | **Comments Risk factor** | **Directness *** | **Study limitations *** | **Precision *** |
| --- | --- | --- | --- | --- | --- | --- | --- | --- | --- |
|  |  |  |  | **Intervention** | **Control** |  |  |  |  |

| Roman, 2020  multicenter (8 centers, Italy, US, Spain,  Poland, Denmark, Switzerland. | | Twins DA | | Asymptomatic women with twins, dilated cervix 1-5 cm by pelvic examination and or speculum examination  and/or TVS | I: 17 C:13 | Physical examination indicated McDonald cerclage  Child level: 0/30 (0%), RR NA | No cerclage  Child level: 0/6 (0%) | Not PO  NEC grade 3 and 4 | + | + | - |
| --- | --- | --- | --- | --- | --- | --- | --- | --- | --- | --- | --- |
| **Systematic reviews**  (only articles with results not shown in original articles are included here) Assessment of Directness, Study limitations and Precision refer to the original articles | | | | | | | | | | | |
| **Alfirevic 2017** | **Singletons** | | | | | | | | | | |
| Althuisius, 2001 Netherlands | Singletons | | Previous PTB <34 w, PPROM<32 w or uterine anomaly, or prior cold knife conization  and TVS CL <25 mm at <27 w | | I: 19 C:16 | McDonald cerclage + bedrest  0/19  No statistics | No cerclage, bedrest  0/16 | Not PO | + | - | - |
| Berghella, 2004  USA (2 centers) | Singletons | | ≥1 of high-risk factors for PTB (≥1 PTB <35 w,  ≥2 curettages, diethylstilbestrol exposure, cone biopsy, Mullerian anomaly, or twin pregnancy) and/or TVS CL <25 mm or significant funneling | | I: 28  C: 29 | McDonald cerclage + bedrest  1/28 (3.6%)  No statistics | No cerclage, bedrest  0/29 (0%) | Not PO Denominator in Cochrane stated as 9, corrected to 29 | + | ? | - |
| Owen, 2009 USA | Singletons | | History of sPTB <34 w  or PPROM + TVS CL <25 mm | | I: 149  C: 153 | McDonald cerclage 2/148 (1.4%)  No statistics | No cerclage 2/152 (1.3%) | Not PO Control group: 153  in article | ? | ? | ? |

C; control, CL; cervical length, DA; diamniotic, I; intervention, NA; not applicable, NEC; necrotizing enterocolitis, PO; primary outcome, PPROM; preterm prelabor rupture of the membranes, PTB; preterm birth, RR; risk ratio, sPTB; spontaneous PTB, TVS; transvaginal scan, w; weeks

| **Author, year Country**  **Trial acronym** | **Singletons/ Twins/ Triplets** | **Risk factor** | **Number**  **of randomized patients**  **n=** | **Results** | | **Comments Risk factor** | **Directness *** | **Study limitations *** | **Precision *** |
| --- | --- | --- | --- | --- | --- | --- | --- | --- | --- |
|  |  |  |  | **Intervention** | **Control** |  |  |  |  |

| To, 2004 UK  (6 countries; UK, Brazil, South Africa, Slovenia, Greece, Chile; 12 hospitals) | Singletons | TVS CL ≤15 mm.  Previous cervical surgery: I: 6%  C: 7% | I: 127  C: 126 | Shirodkar cerclage 5/123 (4%)  RR 2.46  (95% CI 0.49-12.43) p=0.44 | No cerclage 2/121 (2%) | Not PO Positive fetal blood culture | ? | ? | ? |
| --- | --- | --- | --- | --- | --- | --- | --- | --- | --- |
| Roman, 2020  multicenter (8 centers, Italy, USA, Spain, Poland, Denmark, Switzerland) | Twins DA | Asymptomatic women with twins, dilated cervix 1-5 cm by pelvic examination and or speculum examination and/or TVS | I: 17 C:13 | Physical examination indicated McDonald cerclage  2/30 (6.6%)  RR 0.40 (95% CI 0.04-3.74),  p=0.43 (fetal level denominator) | No cerclage  1/6 (16.6%)  (fetal level denominator) | Not PO Outcomes of neonates born alive | + | + | - |

C; control, CI; confidence interval, CL; cervical length, DA, diamniotic, I; intervention, RR; risk ratio PO; primary outcome, TVS; transvaginal scan, UK; United Kingdom

| **Author, year Country**  **Trial acronym** | **Singletons/ Twins/ Triplets** | **Risk factor** | **Number of**  **randomized patients**  **n=** | **Results** | | **Comments Risk factor** | **Directness *** | **Study limitations *** | **Precision *** |
| --- | --- | --- | --- | --- | --- | --- | --- | --- | --- |
|  |  |  |  | **Intervention** | **Control** |  |  |  |  |

| To, 2004 UK  (6 countries; UK, Brazil, South Africa, Slovenia, Greece, Chile; 12 hospitals) | Singletons | TVS CL ≤15 mm.  Previous cervical surgery: I: 6%  C: 7% | I: 127  C: 126 | | Shirodkar cerclage 0/123 (0%)  RR 0.14 (95% CI 0.01-2.69), p=0.12 | | No cerclage 3/121 (2%) | No PO  Outcome of neonates born alive. | ? | ? | ? |
| --- | --- | --- | --- | --- | --- | --- | --- | --- | --- | --- | --- |
| Roman, 2020  multicenter (8 centers, Italy, US, Spain, Poland, Denmark,  Switzerland) | Twins DA | Asymptomatic women with twins, dilated cervix 1-5 cm by pelvic examination and or speculum examination  and/or TVS | I: 17 C:13 | | Physical examination indicated McDonald cerclage  Child level 5/30 (16.6%)  RR 1.0 (95% CI 0.14-7.1), p=1.0 | | No cerclage  Child level 1/6 (16.6%) | No PO  Outcome of neonates born alive.  ROP treated with laser therapy | + | + | - |
| **Systematic reviews**  (only articles with results not shown in original articles are included here) Assessment of Directness, Study limitations and Precision refer to the original articles | | | | | | | | | | | |
| **Alfirevic, 2017** | **Singletons** | | | | | | | | | | |
| Owen, 2009 USA | Singletons | History of sPTB <34 w or PPROM + TVS CL <25 mm | | I: 149  C: 153 | McDonald cerclage 3/148 (2.0%)  No statistics | No cerclage 5/152 (3.3%) | | Not PO  153 women in the original paper | ? | ? | ? |

C; control, CI; confidence interval, CL; cervical length, I; intervention, PPROM; preterm prelabor rupture of the membranes, PO; primary outcome, PTB; preterm birth, ROP; retinopathy of prematurity, RR; risk ratio; sPTB; spontaneous preterm birth, TVS; transvaginal scan, UK; United Kingdom, w; weeks

| **Author, year Country**  **Trial acronym** | **Singletons/ Twins/ Triplets** | **Risk factor** | **Number of**  **randomized patients**  **n=** | **Results** | | **Comments Risk factor** | **Directness *** | **Study limitations *** | **Precision *** |
| --- | --- | --- | --- | --- | --- | --- | --- | --- | --- |
|  |  |  |  | **Intervention** | **Control** |  |  |  |  |

| Ezechi, 2004, Nigeria | Not stated | Previous PTB | I: 38  C: 43 | McDonald cerclage 1/38 (2.6%)  OR 0.10 (95% CI 0.00-0.87) p=0.03 | No cerclage 9/43 (20.9%) | Not PO Singletons/twins not stated but singletons correspond  to neonatal outcomes | - | ? | ? |
| --- | --- | --- | --- | --- | --- | --- | --- | --- | --- |
| Berghella, 2004 USA  (2 centers) | Singletons and twins (4 sets of  twins, 7%) | Previous PTB <35w, cone biopsy, uterine  malformation,  and TVS CL <25 mm | I: 31  C: 30 | McDonald cerclage All 9/34 (26%) p=0.53  Mean (SD) NICU stay  60.4 (62.1) days p=0.55  NICU admissions Singletons 9/28 (32%)  Twins 0/6 | No cerclage, bedrest All 11/31 (35%)  Mean (SD) NICU stay  45.7 (44.6) days  NICU admissions Singletons 9/29 (31%)  Twins 2/2 (100%) | Not PO  3 sets of twins (n=6) in intervention group, 1 set of twins (n=2) in the control group  Admission to NICU Length of NICU stay in days  Denominator all neonates including deaths | + | ? | - |
| Roman, 2020  multicenter (8 centers, Italy, USA, Spain, Poland, Denmark, Switzerland) | Twins DA | Asymptomatic women with twins, dilated cervix 1-5 cm by pelvic examination and or speculum examination and/or TVS | I:17 C:13 | Physical examination indicated McDonald cerclage Child level  22/30 (73.3%)  RR 0.73 (95% CI 0.6-1.0) p=0.3  Mean (SD) NICU stay  68.2 (65.3) days Mean difference  29.8 days  (95% CI -14.6 to 74.2) | No cerclage  Child level 6/6 (100%)  Mean (SD) NICU stay  92.3 (53.4) days | Not PO Denominator surviving  neonates.  Admission to NICU Length of NICU stay in days | + | + | - |

C; control, CI; confidence interval, CL; cervical length, DA; diamniotic, I; intervention, NICU; neonatal intensive care unit, OR; odds ratio, PO; primary outcome, RR; risk ratio, SD; standard deviation, TVS; transvaginal scan, w; weeks

| **Author, year Country**  **Trial acronym** | **Singletons/ Twins/ Triplets** | **Risk factor** | **Number of**  **randomized patients**  **n=** | **Results** | | **Comments Risk factor** | **Directness *** | **Study limitations *** | **Precision *** |
| --- | --- | --- | --- | --- | --- | --- | --- | --- | --- |
|  |  |  |  | **Intervention** | **Control** |  |  |  |  |

| Rush, 1984 South Africa | Singletons | History of previous late miscarriage or PTB out of at least one spontaneous between 14-36 w and 2, 3, or 4 previous pregnancies ending spontaneously  before 37 w | I: 96  C: 98 | McDonald cerclage Maternal pyrexia 10/96 (10.4%) p=0.07 | No cerclage Maternal pyrexia 3/98 (3.1%) | Not PO  Fever ≥38° C on at least one occasion during puerperium  Cochrane 11/96 resp 4/98 | ? | ? | ? |
| --- | --- | --- | --- | --- | --- | --- | --- | --- | --- |
| To, 2004 UK  (6 countries; UK, Brazil, South Africa, Slovenia, Greece, Chile; 12 hospitals) | Singletons | TVS CL ≤15 mm Previous cervical surgery: I: 6%  C: 7% | I: 127  C: 126 | Shirodkar cerclage Maternal pyrexia 5/127 (4%)  RR 4.92 (95% CI 0.58-41.93) p=0.21  Symptomatic vaginal discharge 8/127 (6%)  RR 7.87 (95% CI 1.00-62.04) p=0.04 | No cerclage Maternal pyrexia 1/126 (1%)  Symptomatic vaginal discharge  1/126 (1%) | No PO  Maternal pyrexia defined as fever of 38°C or more during antenatal hospital stay | ? | ? | ? |
| Rust, 2000 USA | Singletons and twins Singletons (n=54), twins  and triplets (7/61, 11.5%) | History of PTB, second trimester pregnancy loss, previous cervical surgery, uterine anomaly, multifetal pregnancy and a TVS CL <25 mm or dilated internal os | I:31 C:30 | McDonald cerclage, modified bedrest  Chorioamnionitis 5/31 (16.1%) p=0.4 | No cerclage, modified bedrest  Chorioamnionitis 2/30 (6.7%) | 5 twin and 2 triplets evenly distributed between the two groups, though not defined in the outcomes  Not included in MA due to  >10% multifetal pregnancies | - | - | ? |
| Roman, 2020  Multicenter (8 centers, Italy, US, Spain, Poland, Denmark, Switzerland) | Twins DA | Asymptomatic women with twins, dilated cervix 1-5 cm by pelvic examination and or speculum examination and/or TVS | I: 17 C:13 | Physical examination indicated McDonald cerclage  Clinical chorioamnionitis 2/17 (11.8%)  RR 0.51 (95% CI 0.1-2.6) p=0.62 | No cerclage  Clinical chorioamnionitis 3/13 (23.1%) | Not PO | + | + | - |

| **Author, year Country**  **Trial acronym** | **Singletons/ Twins/ Triplets** | **Risk factor** | **Number of**  **randomized patients**  **n=** | **Results** | | **Comments Risk factor** | **Directness *** | **Study limitations *** | **Precision *** |
| --- | --- | --- | --- | --- | --- | --- | --- | --- | --- |
|  |  |  |  | **Intervention** | **Control** |  |  |  |  |

| **Systematic reviews**  (only articles with results not shown in original articles are included here) Assessment of Directness, Study limitations and Precision refer to the original articles | | | | | | | | | |
| --- | --- | --- | --- | --- | --- | --- | --- | --- | --- |
| **Alfirevic, 2017 Cochrane** | **Singletons** | | | | | | | | |
| Macnaughton MRC/RCOG, 1993  Multicenter: UK, France, Hungary, Norway, Italy, Belgium, Zimbabwe, South  Africa, Iceland, Ireland, the Netherlands, Canada | Singletons | Included if uncertainty if cerclage or not for risk patients: Previous PTB, previous second trimester miscarriage, previous early abortion, cervical amputation, cone biopsy, uterine anomaly, twin pregnancy | I: 635  C: 629 | Cerclage (not prespecified) Pyrexia  23/407 (5.7%)  No statistics Chorioamnionitis 1/635 (0.2%)  No statistics | No cerclage Pyrexia 11/391 (2.8%)  Chorioamnionitis 0/629 (0%) | Not PO  All miscarriages included in denominator unclear if included in numerator | ? | ? | ? |

C; control, CI; confidence interval, CL; cervical lengh, I; intervention, PO; primary outcome, PTB, preterm delivery, RR; risk ratio, TVS; transvaginal scan, UK; United Kingdom, w; weeks

| **Author, year Country**  **Trial acronym** | **Singletons**  **/Twins/ Triplets** | **Risk factor** | **Number of**  **randomized patients**  **n=** | **Results** | | **Comments Risk factor** | **Directness *** | **Study limitations *** | **Precision *** |
| --- | --- | --- | --- | --- | --- | --- | --- | --- | --- |
|  |  |  |  | **Intervention** | **Control** |  |  |  |  |

| Rush, 1984 South Africa | Singletons | History of previous late miscarriage or PTB out of at least one spontaneous between 14-36 w and 2, 3, or 4 previous pregnancies ending spontaneously before  37 w | I: 96  C: 98 | McDonald cerclage 18/96 (18.8%)  NS | No cerclage 12/98 (12.2%) | Not PO | ? | ? | ? |
| --- | --- | --- | --- | --- | --- | --- | --- | --- | --- |
| To, 2004 UK  (6 countries; UK, Brazil, South Africa, Slovenia, Greece, Chile; 12 hospitals) | Singletons | TVS CL ≤15 mm Previous cervical surgery: I: 6%  C: 7% | I: 127  C: 126 | Shirodkar cerclage 23/127 (18%)  RR 1.20 (95% CI 0.69-2.09)  p= 0.52 | No cerclage 19/126 (15%) | No PO | ? | ? | ? |
| Berghella, 2004 USA  (2 centers) | Singletons and twins (4 sets of  twins, 7%) | Previous PTB <35 w, cone biopsy, uterine malformation  and/or short TVS CL <25 mm | I: 31  C: 30 | McDonald cerclage + bedrest Singletons  8/28 (32.0%)  No statistics Twins (calculated) 3/3 (100%)  No statistics | No cerclage, bedrest Singletons  10/29 (34.5%)  Twins calculated 0/1 | Not PO  3 women with twin pregnancies in the intervention group, all early PTB at 20, 21 and 22 weeks. One woman with twin pregnancy in the bedrest group, PTB at 34 w  Information about singleton pregnancies from Alfirevic, Cochrane 2017 | + | ? | - |
| Dor, 1982  Israel | Twins | Twin pregnancy after OI | I: 22/44 C: 23/46 | McDonald cerclage 2/22 (9.1%)  NS  RR not presented | No cerclage 3/23 (13.0%) | PO not defined | ? | - | - |
| Roman, 2020  Multicenter (8 centers; Italy, USA, Spain, Poland, Denmark, Switzerland) | Twins DA | Asymptomatic women with twins, dilated cervix 1-5 cm by pelvic examination and/or speculum examination  or TVS | I: 17 C:13 | Physical examination indicated McDonald cerclage 11/17 (64.7%)  RR 1.68 (95% CI 0.83-3.86) p=0.26 | No cerclage 5/13 (38.5%) | Not PO PPROM defined<34 w | + | + | - |

| **Author, year Country**  **Trial acronym** | **Singletons**  **/Twins/ Triplets** | **Risk factor** | **Number of**  **randomized patients**  **n=** | **Results** | | **Comments Risk factor** | **Directness *** | **Study limitations *** | **Precision *** |
| --- | --- | --- | --- | --- | --- | --- | --- | --- | --- |
|  |  |  |  | **Intervention** | **Control** |  |  |  |  |

| **Systematic reviews**  (only articles with results not shown in original articles are included here) Assessment of Directness, Study limitations and Precision refer to the original articles | | | | | | | | | |
| --- | --- | --- | --- | --- | --- | --- | --- | --- | --- |
| **Alfirevic 2017** | **Singletons** | | | | | | | | |
| Althuisius, 2001 Netherlands | Singletons | Previous PTB <34 w, PPROM<32 w or uterine anomaly, or prior cold knife conization and TVS CL<25 mm at <27 w | I: 19 C:16 | McDonald cerclage + bedrest 0/19  No statistics | No cerclage, bedrest  8/16 (50.0%) | Not PO | + | - | - |
| Macnaughton MRC/RCOG, 1993  Multicenter: UK, France, Hungary, Norway, Italy, Belgium, Zimbabwe, South Africa, Iceland, Ireland, the Netherlands, Canada | Singletons | Included if uncertainty if cerclage or not for risk patients: Previous PTB, previous second trimester miscarriage, previous early abortion, cervical amputation, cone biopsy, uterine anomaly, twin pregnancy | I: 635  C: 629 | Cerclage (not prespecified) 3/635 (0.5%)  No statistics | No cerclage 0/629 (0%) | Not PO  NB low rate of PPROM Miscarriage included in denominator | ? | ? | ? |

CL; cervical length, C; control, I; intervention, OI; ovulation induction, NS; not significant, PPROM; preterm prelabor rupture of membranes, RR; risk ratio, PO; primary outcome, TVS; transvaginal scan, UK; United Kingdom, w; weeks
