# Supplementary material for: Progesterone, cerclage, pessary, or acetylsalicylic acid for prevention of preterm birth in singleton and multifetal pregnancies – A systematic review and meta-analyses
Source: Front Med (Lausanne). 2023 Feb 28;10:1111315. doi: 10.3389/fmed.2023.1111315 (PMC10015499; doi:10.3389/fmed.2023.1111315)
Supplement: Supplementary file 1 [file Data_Sheet_1.zip › Data Sheet 1_corrected/Appendix 4.3 Outcome tables_Pessary.docx]

## **Progesterone, cerclage, pessary, or acetylsalicylic acid for prevention of preterm birth in singleton and multifetal pregnancies**

**Appendix 4.3 Outcome tables pessary vs no pessary in singleton and multifetal pregnancies**

**Preterm birth**STable 4.3.1a Any preterm birth <37 weeks
STable 4.3.1b Spontaneous preterm birth <37 weeks
STable 4.3.2a Any preterm birth <34 weeks

STable 4.3.2b Spontaneous preterm birth <34 weeks

STable 4.3.3a Any preterm birth <32 weeks

STable 4.3.3b Spontaneous preterm birth <32 weeks

STable 4.3.4a Any preterm birth <28 weeks

STable 4.3.4b Spontaneous preterm birth <28 weeks

**Gestational age and birth weight**

STable 4.3.5 Gestational age at delivery

STable 4.3.6 Low birth weight

STable 4.3.7 Very low birth weigh

**Perinatal mortality and neonatal morbidity**

STable 4.3.5 Perinatal mortality

STable 4.3.6 Neonatal mortality <28d

STable 4.3.7 Composite adverse neonatal outcome

STable 4.3.8 Respiratory distress syndrome

STable 4.3.9 Bronchopulmonary dysplasia

STable 4.3.10 Intraventricular hemorrhage

STable 4.3.11 Necrotizing enterocolitis

STable 4.3.12 Neonatal sepsis

STable 4.3.13 Retinopathy of prematurity

STable 4.3.14 Admittance to neonatal intensive care unit

STable 4.3.15 Long-term child outcome

**Maternal mortality and morbidity**

STable 4.3.19 Maternal mortality

STable 4.3.20 Hypertensive disorders in pregnancy

STable 4.3.21 Chorioamnionitis

STable 4.3.22 Genitourinary infections

STable 4.3.23 Vaginal discharge

STable 4.3.24 Preterm prelabor rupture of membranes

Prevention of preterm birth

* + No or minor problems

? Some problems

- Major problems

STable 4.3.1.a. Intervention pessary

Outcome variable: Any preterm birth before 37 gestational weeks

| **Author, year Country**  **Trial acronym** | **Singletons/ Twins/ Triplets** | **Risk factor** | **Number of**  **randomized patients**  **n=** | **Results** | | **Comments** | **Directness *** | **Study limitations *** | **Precision *** |
| --- | --- | --- | --- | --- | --- | --- | --- | --- | --- |
|  |  |  |  | **Intervention** | **Control** |  |  |  |  |
|  |  |  |  |  |  |  |  |  |  |
| Dugoff 2018 USA PoPPS | Singletons | TVS CL <25 mm | 122  I: 61  C: 61 | Pessary (Biotech cup) 26/60 (43.3%)  RR 1.09 (95% CI 0.71-1.68) p=0.71 | No pessary 23/58 (39.7%) | PO  If CL <20 mm progesterone, I: 84% C: 91% | ? | ? | - |
| Hui 2013  China | Singletons | TVS CL <25 mm | 108  I: 53  C: 55 | Pessary (Arabin) 8/53 (15.1%)  RR 0.96 (95% CI 0.81-1.14) p=0.67 | Digital examination at entry to simulate pessary insertion 10/55 (18.2%) | Not PO | + | ? | - |
| Karbasian 2016  Iran | Singletons | TVS CL <25 mm | 146  I: 73  C: 73 | Pessary (Arabin)+vaginal progesterone  14/71 (19.7%) p=0.6 | Vaginal progesterone 12/73 (16.4%) | PO | + | ? | - |
| Pacagnella 2022  Brazil | Singletons 92.4%  Twins 7.6% | All DA CL <30 mm | 936  I: 475  C: 461 | Pessary (Ingamed)+vaginal progesterone Singletons  104/431 (24.1%)  RR 0.87 (95% CI 0.70-1.09)  p= 0.24  Multifetal 34/43 (79.1%)  RR 0.89 (95%CI 0.72-1.08)  p= 0.24 | Vaginal progesterone  Singletons 119/430 (27.7%)  Multifetal 25/28 (89.3%) | Not PO | ? | ? | ? |
| Saccone 2017c  Italy | Singletons | TVS CL ≤25 mm Prior cervical surgery I: 7 (4.7%)  C: 5 (3.3%) | 300  I: 150 C:150 | Pessary (Arabin) 35/150 (23.3%)  RR 0.66 (95% CI 0.46-0.95) p=0.03 | Standard care 53/150 (35.3%) | Not PO  If CL <20 mm progesterone, I: 133 (88.7%) C: 125  (83.3%) | ? | ? | ? |

Prevention of preterm birth

* + No or minor problems

? Some problems

- Major problems

STable 4.3.1.a. cont. Intervention pessary

Outcome variable: Any preterm birth before 37 gestational weeks

| **Author, year Country**  **Trial acronym** | **Singletons/ Twins/ Triplets** | **Risk factor** | **Number of**  **randomized patients**  **n=** | **Results** | | **Comments** | **Directness *** | **Study limitations *** | **Precision *** |
| --- | --- | --- | --- | --- | --- | --- | --- | --- | --- |
|  |  |  |  | **Intervention** | **Control** |  |  |  |  |
|  |  |  |  |  |  |  |  |  |  |
| Berghella 2017a USA PoPPT | Twins | TVS CL ≤30 mm DCDA 78%  MCDA 22%  Previous PTB 0 vs 13% | 46  I: 23  C: 23 | Pessary (Biotech cup) 19/23 (83%)  RR 1.00 (95% CI 0.76-2.30) p=1.00 | No pessary 19/23 (83%) | Not PO | + | ? | - |
| Liem 2013a  The Netherlands ProTWIN | Twins Triplets (2%) | Triplets I: 2% (n=9)  C: 2% (n=9)  MC twins I: 22%  C: 24%  Previous PTB 7 vs 6% | 813  I: 403  C: 410 | Pessary (Arabin) 222/401 (55%)  RR 0.94 (95% CI 0.87-1.07)  No p-value | Standard care 233/407 (57%) | Not PO  I: 5 cerclage  C: 0 cerclage | + | ? | ? |
| Norman 2021 United Kingdom,  Belgium STOPPIT-2 | Twins | MCDA 20%  DCDA 80% CL<35mm | 503  I: 250  C: 253 | Pessary (Arabin) 158/250 (63.2%)  OR 0.95 (95% CI 0.57-1.58) p=0.79 | Standard care 161/253 (63.6%) | Not PO | + | ? | ? |

C; control, CI; confidence interval, CL; cervical length, DA; diamniotic, DCDA; dichorionic diamniotic, I; intervention, MC; monochorionic, MCDA; monochorionic diamniotic, OR; odds ratio, PO; primary outcome, PTB; preterm birth, RR; risk ratio, TVS; transvaginal sonography

Preterm birth STable 4.3.1.b. Intervention pessary

* + No or minor problems

? Some problems

- Major problems

Outcome variable: Spontaneous preterm birth before 37 gestational weeks

| **Author, year Country**  **Trial acronym** | **Singletons/ Twins/ Triplets** | **Risk factor** | **Number of**  **randomized patients**  **n=** | **Results** | | **Comments** | **Directness *** | **Study limitations *** | **Precision *** |
| --- | --- | --- | --- | --- | --- | --- | --- | --- | --- |
|  |  |  |  | **Intervention** | **Control** |  |  |  |  |
|  |  |  |  |  |  |  |  |  |  |
| Dugoff 2018 USA PoPPS | Singletons | TVS CL <25 mm | 122  I: 61  C: 61 | Pessary (Biotech cup) 23/60 (38.3%)  RR 1.17 (95% CI 0.72-1.91) p=0.59 | No pessary 19/58 (32.8%) | Not PO  If CL <20 mm progesterone, I: 84% C: 91% | ? | ? | - |
| Goya 2012  Spain PECEP | Singletons | TVS CL <25 mm  11% in both groups had previous PTB | 385  I: 192  C: 193 | Pessary (Arabin) 41/190 (22%)  OR 0.19 (95% CI 0.12-0.30) p=<0.0001 | Standard care 113/190 (59%) | Not PO | ? | ? | + |
| Pacagnella 2022  Brazil | Singletons 92.4%  Twins 7.6% | All DA CL <30 mm | 936  I: 475  C: 461 | Pessary (Ingamed)+vaginal progesterone  74/461 (16.1%)  RR 0.85 (95% CI 0.64-1.13) | Vaginal progesterone  84/435 (19.3%) | PO | ? | ? | ? |
| Saccone 2017c  Italy | Singletons | TVS CL ≤25 mm Prior cervical surgery I: 7 (4.7%)  C: 5 (3.3%) | 300  I: 150  C: 150 | Pessary (Arabin) 30/150 (20.0%)  RR 0.61 (95% CI 0.41-0.91) p=0.02 | Standard care 49/150 (32.7%) | Not PO  If CL <20 mm progesterone, I: 133 (88.7%) C: 125  (83.3%) | ? | ? | ? |
| Berghella 2017a USA PoPPT | Twins | TVS CL ≤30 mm DCDA 78%  MCDA 22%  Previous PTB 0 vs 13% | 46  I: 23  C: 23 | Pessary (Biotech cup) 12/23 (52%)  RR 1.00 (95% CI 0.58-1.74) p=1.00 | No pessary 12/23 (52%) | Not PO | + | ? | - |
| Goya 2016  Spain  PECEP-Twins | Twins | TVS CL ≤25 mm Previous PTB  I: 16.7%  C: 17.6%  MC twins I: 19.1%  C: 17.6% | 137  I: 68  C: 66 | Pessary (Arabin) 47/68 (69.1%)  RR 0.95 (95% CI 0.76-1.18)  No p-value | Standard care 48/66 (72.7%) | Not PO | + | ? | + |
| Norman 2021 United Kingdom,  Belgium STOPPIT-2 | Twins | MCDA 20%  DCDA 80% CL<35mm | 503  I: 250  C: 253 | Pessary (Arabin) 56/250 (22.4%)  OR 0.81 (95% CI 0.47-1.41) p=0.32 | Standard care 66/253 (26.1%) | Not PO | + | ? | ? |

C; control, CI; confidence interval, CL; cervical length, DA; diamniotic, DCDA; dichorionic diamniotic, I; intervention, MC; monochorionic, MCDA; monochorionic diamniotic, OR; odds ratio, PO; primary outcome, PTB; preterm birth, RR; risk ratio, TVS; transvaginal sonography

Prevention of preterm birth

STable 4.3.2.a. Intervention pessary

Outcome variable: Any preterm birth before 34 gestational weeks

* + No or minor problems

? Some problems

- Major problems

| **Author, year Country**  **Trial acronym** | **Singletons/ Twins/ Triplets** | **Risk factor** | **Number of**  **randomized patients**  **n=** | **Results** | | **Comments** | **Directness *** | **Study limitations *** | **Precision *** |
| --- | --- | --- | --- | --- | --- | --- | --- | --- | --- |
|  |  |  |  | **Intervention** | **Control** |  |  |  |  |
|  |  |  |  |  |  |  |  |  |  |
| Dugoff 2018 USA PoPPS | Singletons | TVS CL <25 mm | 122  I: 61  C: 61 | Pessary (Biotech cup) 20/60 (33.3%)  RR 1.14 (95% CI 0.67-1.94) p=0.69 | No pessary 17/58 (29.3%) | Not PO  If CL <20 mm progesterone, I: 84% C: 91% | ? | ? | - |
| Goya 2012  Spain PECEP | Singletons | TVS CL <25 mm  11% in both groups had previous PTB | 385  I: 192  C: 193 | Pessary (Arabin) 14/190 (7%)  OR 0.21 (95% CI 0.10-0.40) p<0.0001 | Standard care 53/190 (28%) | Not PO | ? | ? | + |
| Hui 2013  China | Singletons | TVS CL <25 mm  Previous PTB 5.7 vs 10.9% | 108  I: 53  C: 55 | Pessary (Arabin) 5/53 (9.4%)  RR 1.04 (95% CI 0.94-1.12) p=0.46 | Digital examination at entry to simulate pessary insertion  3/55 (5.5%) | PO | + | ? | - |
| Karbasian 2016  Iran | Singletons | TVS CL <25 mm | 146  I: 73  C: 73 | Pessary (Arabin)+vaginal progesterone  10/71 (14.1%)  No p-value | Vaginal progesterone 7/73 (9.6%) | Not PO | + | ? | - |
| Nicolaides 2016b  9 countries | Singletons | TVS CL <25 mm  Previous PTB 15.1  vs 18% | 935  I: 466  C: 469 | Pessary (Arabin) 60/465 (12.9%)  OR 1.16 (95% CI 0.78-1.72) p=0.47 | Standard care 53/467 (11.3%) | Not PO  If CL <15 mm progesterone, I: 204 (43.9%) C: 219  (46.9%) | + | ? | ? |
| Pacagnella 2022  Brazil | Singletons 92.4%  Twins 7.6% | All DA CL <30 mm | 936  I: 475  C: 461 | Pessary (Ingamed)+vaginal progesterone  Singletons 33/431 (7.7%)  RR 0.63 (95% CI 0.42-0.96)  p= 0.03  Multifetal 14/43 (32.6%)  RR 0.76 (95%CI 0.41-1.39)  p= 0.43 | Vaginal progesterone  Singletons 52/430 (12.1%)  Multifetal 12/28 (42.9%) | Not PO | ? | ? | ? |

* + No or minor problems

? Some problems

- Major problems

Prevention of preterm birth

STable 4.3.2.a. cont. Intervention pessary

Outcome variable: Any preterm birth before 34 gestational weeks

| **Author, year Country**  **Trial acronym** | **Singletons/ Twins/ Triplets** | **Risk factor** | **Number of**  **randomized patients**  **n=** | **Results** | | **Comments** | **Directness *** | **Study limitations *** | **Precision *** |
| --- | --- | --- | --- | --- | --- | --- | --- | --- | --- |
|  |  |  |  | **Intervention** | **Control** |  |  |  |  |
|  |  |  |  |  |  |  |  |  |  |
| Saccone 2017c  Italy | Singletons | TVS CL ≤25 mm Prior cervical surgery I: 7 (4.7%)  C: 5 (3.3%) | 300  I: 150  C: 150 | Pessary (Arabin) 14/150 (9.3%)  RR 0.54 (95% CI 0.29-0.99) p=0.04 | Standard care 26/150 (17.3%) | Not PO  If CL <20mm progesterone, I: 133 (88.7%) C: 125  (83.3%) | ? | ? | ? |
| Berghella 2017a USA PoPPT | Twins | TVS CL ≤30 mm DCDA 78% MCDA22%  Previous PTB 0 vs 13% | 46  I: 23  C: 23 | Pessary (Biotech cup) 9/23 (39%)  RR 1.13 (95% CI 0.53-2.40) p=1.00 | No pessary 8/23 (35%) | Not PO | + | ? | - |
| Goya 2016  Spain  PECEP-Twins | Twins | TVS CL ≤25 mm Previous PTB  I: 16.7%  C: 17.6%  MC twins I: 19.1%  C: 17.6% | 137  I: 68  C: 66 | Pessary (Arabin) 12/68 (17.6%)  RR 0.43 (95% CI 0.24-0.78) p=0.002 | Standard care 27/66 (40.9%) | Not PO | + | ? | + |
| Nicolaides 2016a  12 countries | Twins | MC twins: I: 18.8%  C: 18.8%  Previous PTB 8.8 vs 14.3% | 1180  I: 590  C: 590 | Pessary (Arabin) 98/588 (16.7%)  RR 1.067 (95% CI 0.822-1.385)  No p-value | Standard care 92/589 (15.6%) | Not PO | + | ? | + |
| Norman 2021 United Kingdom, Belgium STOPPIT-2 | Twins | MCDA 20%  DCDA 80% CL<35mm | 503  I: 250  C: 253 | Pessary (Arabin) 62/250 (24.8%)  OR 0.90 (95% CI 0.52-1-57) p=0.64 | Standard care 66/253 (26.1%) | Not PO | + | ? | ? |

C; control, CI; confidence interval, CL; cervical length, DA; diamniotic, DCDA; dichorionic diamniotic, I; intervention, MC; monochorionic, MCDA; monochorionic diamniotic, OR; odds ratio, PO; primary outcome, PTB; preterm birth, RR; risk ratio, TVS; transvaginal sonography

* + No or minor problems

? Some problems

- Major problems

Prevention of preterm birth

STable 4.3.2.b. Intervention pessary

Outcome variable: Spontaneous preterm birth before 34 gestational weeks

| **Author, year Country**  **Trial acronym** | **Singletons/ Twins/ Triplets** | **Risk factor** | **Number of**  **randomized patients**  **n=** | **Results** | | **Comments** | **Directness *** | **Study limitations *** | **Precision *** |
| --- | --- | --- | --- | --- | --- | --- | --- | --- | --- |
|  |  |  |  | **Intervention** | **Control** |  |  |  |  |
|  |  |  |  |  |  |  |  |  |  |
| Dugoff 2018 USA PoPPS | Singletons | TVS CL <25 mm | 122  I: 61  C: 61 | Pessary (Biotech cup) 19/60 (31.7%)  RR 1.22 (95% CI 0.69-2.17) p=0.55 | No pessary 15/58 (25.9%) | Not PO  If CL <20 mm progesterone, I: 84% C: 91% | ? | ? | - |
| Goya 2012  Spain PECEP | Singletons | TVS CL <25 mm  11% in both groups had previous PTB | 385  I: 192  C: 193 | Pessary (Arabin) 12/190 (6%)  OR 0.18 (95% CI 0.08-0.37) p<0.0001 | Standard care 51/190 (27%) | PO | ? | ? | + |
| Hui 2013  China | Singletons | TVS CL <25 mm  Previous PTB 5.7 vs 10.9% | 108  I: 53  C: 55 | Pessary (Arabin) 5/53 (9.4%)  RR 1.04 (95% CI 0.94-1.12) p=0.46 | Digital examination at entry to simulate pessary insertion 3/55 (5.5%) | Not PO | + | ? | - |
| Nicolaides 2016b  9 countries | Singletons | TVS CL <25 mm  Previous PTB 15.1  vs 18% | 935  I: 466  C: 469 | Pessary (Arabin) 55/460 (12.0%)  OR 1.12 ( 95% CI 0.75-1.69)  p= 0.57 | Standard care 50/464 (10.8) | PO  If CL <15 mm progesterone, I: 204 (43.9%) C: 219  (46.9%) | + | ? | ? |
| Pacagnella 2022  Brazil | Singletons 92.4%  Twins 7.6% | All DA CL <30 mm | 936  I: 475  C: 461 | Pessary (Ingamed)+vaginal progesterone  29/461 (6.3%)  RR 0.68 (95% CI 0.43-1.08)  No p-value | Vaginal progesterone 41/435 (9.4%) | PO | ? | ? | ? |
| Saccone 2017c  Italy | Singletons | TVS CL ≤25 mm Prior cervical surgery I: 7 (4.7%)  C: 5 (3.3%) | 300  I: 150  C: 150 | Pessary (Arabin) 11/150 (7.3%)  RR 0.48 (95% CI 0.24-0.95) p=0.04 | Standard care 23/150 (15.3%) | PO  If CL <20 mm progesterone, I: 133 (88.7%) C: 125  (83.3%) | ? | ? | ? |
| Berghella 2017a USA PoPPT | Twins | TVS CL ≤30 mm DCDA 78%  MCDA 22%  Previous PTB 0 vs 13% | 46  I: 23  C: 23 | Pessary (Biotech cup) 7/23 (30%)  RR 1.0 (95%CI 0.42-2.40) p=1.0 | No pessary 7/23 (30%) | Not PO | + | ? | - |

* + No or minor problems

? Some problems

- Major problems

Prevention of preterm birth

STable 4.3.2.b. cont. Intervention pessary

Outcome variable: Spontaneous preterm birth before 34 gestational weeks

| **Author, year Country**  **Trial acronym** | **Singletons/ Twins/ Triplets** | **Risk factor** | **Number of**  **randomized patients**  **n=** | **Results** | | **Comments** | **Directness *** | **Study limitations *** | **Precision *** |
| --- | --- | --- | --- | --- | --- | --- | --- | --- | --- |
|  |  |  |  | **Intervention** | **Control** |  |  |  |  |
|  |  |  |  |  |  |  |  |  |  |
| Goya 2016  Spain  PECEP-Twins | Twins | TVS CL ≤25 mm Previous PTB  I: 16.7%  C: 17.6%  MC twins I: 19.1%  C: 17.6% | 137  I: 68  C: 66 | Pessary (Arabin) 11/68 (16.2%)  RR 0.41 (95% CI 0.22-0.76)  p= 0.003 | Standard care 26/66 (39.4%) | PO | + | ? | + |
| Nicolaides 2016a  12 countries | Twins | MC twins: I: 18.8%  C: 18.8%  Previous PTB 8.8 vs 14.3% | 1180  I: 590  C: 590 | Pessary (Arabin) 80/588 (13.6%)  RR 1.054 (95% CI 0.787-1.413)  No p-value | Standard care 76/589 (12.9%) | PO | + | ? | + |
| Norman 2021 United Kingdom, Belgium  STOPPIT-2 | Twins | MCDA 20%  DCDA 80% CL<35mm | 503  I: 250  C: 253 | Pessary (Arabin) 37/250 (14.8%)  OR 0.77 (95% CI 0.40-1-47) p=0.30 | Standard care 46/253 (18.2%) | PO (obstetric) | + | ? | ? |

C; control, CI; confidence interval, CL; cervical length, DA; diamniotic, DCDA; dichorionic diamniotic, I; intervention, MC; monochorionic, MCDA; monochorionic diamniotic, OR; odds ratio, PO; primary outcome, PTB; preterm birth, RR; risk ratio, TVS; transvaginal sonography

Prevention of preterm birth

STable 4.3.3.a. Intervention pessary

Outcome variable: Any preterm birth before 32 gestational weeks

* + No or minor problems

? Some problems

- Major problems

| **Author, year Country**  **Trial acronym** | **Singletons/ Twins/ Triplets** | **Risk factor** | **Number of**  **randomized patients**  **n=** | **Results** | | **Comments** | **Directness *** | **Study limitations *** | **Precision *** |
| --- | --- | --- | --- | --- | --- | --- | --- | --- | --- |
|  |  |  |  | **Intervention** | **Control** |  |  |  |  |
|  |  |  |  |  |  |  |  |  |  |
| Karbasian 2016  Iran | Singletons | TVS CL <25 mm | 146  I: 73  C: 73 | Pessary (Arabin)+vaginal progesterone  5/73 (6.8%) | Vaginal progesterone 6/73 (8.2%) | Not PO | + | ? | - |
| Nicolaides 2016b  9 countries | Singletons | TVS CL <25 mm  Previous PTB 15.1 vs 18% | 935  I: 466  C: 469 | Pessary (Arabin) 46/465 (9.9%)  OR 1.36 (95% CI 0.86-2.15) p=0.20 | Standard care 35/467 (7.5%) | Not PO  If CL <15 mm progesterone, I: 204 (43.9%) C: 219  (46.9%) | + | ? | ? |
| Pacagnella 2022  Brazil | Singletons 92.4%  Twins 7.6% | All DA CL <30 mm | 936  I: 475  C: 461 | Pessary (Ingamed)+vaginal progesterone Singletons  22/431 (5.1%)  RR 0.61 (95% CI 0.36-1.02)  p= 0.05  Multifetal 5/43 (11.6%)  RR 0.36 (95% CI 0.14-0.97)  p= 0.08 | Vaginal progesterone  Singletons 36/430 (8.4%)  Multifetal 9/28 (32.1%) | Not PO | ? | ? | ? |
| Saccone 2017c  Italy | Singleton | TVS CL ≤25 mm Prior cervical surgery I: 7 (4.7%)  C: 5 (3.3%) | 300  I: 150  C: 150 | Pessary (Arabin) 11/150 (7.3%)  RR 0.73 (95% CI 0.35-1.54) p=0.54 | Standard care 15/150 (10%) | Not PO  If CL <20 mm progesterone, I: 133 (88.7%) C: 125  (83.3%) | ? | ? | ? |
| Nicolaides 2016a  12 countries | Twins | MC twins: I: 18.8%  C: 18.8%  Previous PTB 8.8 vs 14.3% | 1180  I: 590  C: 590 | Pessary (Arabin) 52/588 (8.8%)  RR 0.983 (95% CI 0.682-1.416)  No p-value | Standard care 53/589 (9%) | Not PO | + | ? | + |
| Liem 2013a  The Netherlands ProTWIN | Twins Triplets (2%) | Triplets I: 2% (n=9)  C: 2% (n=9)  MC twins I: 22%  C: 24%  Previous PTB 7 vs 6% | 813  I: 403  C: 410 | Pessary (Arabin) 41/401 (10%)  RR 0.86 (95% CI 0.65-1.15)  No p-value | Standard care 49/407 (12%) | Not PO  I: 5 cerclage  C: 0 cerclage | + | ? | ? |

* + No or minor problems

? Some problems

- Major problems

Prevention of preterm birth

STable 4.3.3.a. cont. Intervention pessary

Outcome variable: Any preterm birth before 32 gestational weeks

| **Author, year Country**  **Trial acronym** | **Singletons/ Twins/ Triplets** | **Risk factor** | **Number of**  **randomized patients**  **n=** | **Results** | | **Comments** | **Directness *** | **Study limitations *** | **Precision *** |
| --- | --- | --- | --- | --- | --- | --- | --- | --- | --- |
|  |  |  |  | **Intervention** | **Control** |  |  |  |  |
|  |  |  |  |  |  |  |  |  |  |
| Norman 2021 United Kingdom,  Belgium STOPPIT-2 | Twins | MCDA 20%  DCDA 80%  CL <35 mm | 503  I: 250  C: 253 | Pessary (Arabin) 35/250 (14.0%)  OR 0.83 (95% CI 0.42-1.63) p=0.47 | Standard care 41/253 (16.2%) | Not PO | + | ? | ? |

C; control, CI; confidence interval, CL; cervical length, DA; diamniotic, DCDA; dichorionic diamniotic, I; intervention, MC; monochorionic, MCDA; monochorionic diamniotic, OR; odds ratio, PO; primary outcome, PTB; preterm birth, RR; risk ratio, TVS; transvaginal sonography

* + No or minor problems

? Some problems

- Major problems

Prevention of preterm birth

STable 4.3.3.b. Intervention pessary

Outcome variable: Spontaneous preterm birth before 32 gestational weeks

| **Author, year Country**  **Trial acronym** | **Singletons/ Twins/ Triplets** | **Risk factor** | **Number of**  **randomized patients**  **n=** | **Results** | | **Comments** | **Directness *** | **Study limitations *** | **Precision *** |
| --- | --- | --- | --- | --- | --- | --- | --- | --- | --- |
|  |  |  |  | **Intervention** | **Control** |  |  |  |  |
|  |  |  |  |  |  |  |  |  |  |
| Pacagnella 2022  Brazil | Singletons 92.4%  Twins 7.6% | All DA CL <30 mm | 936  I: 475  C: 461 | Pessary (Ingamed)+vaginal progesterone  15/461 (3.3%)  RR 0.52 (95% CI 0.28-0.96) | Vaginal progesterone  28/435 (6.4%) | PO | ? | ? | ? |
| Saccone 2017c  Italy | Singletons | TVS  CL ≤25 mm Prior cervical surgery  I: 7 (4.7%)  C: 5 (3.3%) | 300  I: 150  C: 150 | Pessary (Arabin) 10/150 (6.7%)  RR 0.71 (95% CI 0.33-1.56) p=0.52 | Standard care 14/150 (9.3%) | Not PO  If CL <20 mm progesterone, I: 133 (88.7%)  C: 125 (83.3%) | ? | ? | ? |
| Norman 2021 United Kingdom, Belgium STOPPIT-2 | Twins | MCDA 20%  DCDA 80%  CL <35 mm | 503  I: 250  C: 253 | Pessary (Arabin) 26/250 (10.4%)  OR 0.79 (95% CI 0.37-1.68) p=0.43 | Standard care 32/253 (12.6%) | Not PO | + | ? | ? |

C; control, CI; confidence interval, CL; cervical length, Cx; cervix, DA; diamniotic, DCDA; dichorionic diamniotic, I; intervention, MCDA; monochorionic diamniotic, OR; odds ratio, PO; primary outcome, RR; risk ratio, TVS; transvaginal sonography

* + No or minor problems

? Some problems

- Major problems

Prevention of preterm birth

STable 4.3.4.a. cont. Intervention pessary

Outcome variable: Any preterm birth before 28 gestational weeks

| **Author, year Country**  **Trial acronym** | **Singletons/ Twins/ Triplets** | **Risk factor** | **Number of**  **randomized patients**  **n=** | **Results** | | **Comments** | **Directness** | **Study limitations** | **Precision *** |
| --- | --- | --- | --- | --- | --- | --- | --- | --- | --- |
|  |  |  |  | **Intervention** | **Control** |  |  |  |  |
|  |  |  |  |  |  |  |  |  |  |
| Dugoff 2018 USA PoPPS | Singletons | TVS CL <25 mm | 122  I: 61  C: 61 | Pessary (Biotech cup) 12/60 (20.0%)  RR 0.83 (95% CI 0.42-1.64) p=0.66 | No pessary 14/58 (24.1%) | Not PO  If CL <20 mm progesterone, I: 84% C: 91% | ? | ? | - |
| Hui 2013  China | Singletons | TVS CL <25 mm  Previous PTB 5.7 vs 10.9% | 108  I: 53  C: 55 | Pessary (Arabin) 2/53 (3.8%)  RR 0.98 (95% CI 0.90-1.07) p=1.00 | Digital examination at entry to simulate pessary insertion 3/55 (5.5%) | Not PO | + | ? | - |
| Nicolaides 2016b  9 countries | Singletons | TVS CL <25 mm | 935  I: 466  C: 469 | Pessary (Arabin) 25/465 (5.4%)  OR 1.71 (95% CI 0.89-3.29) p=0.11 | Standard care 15/467 (3.2%) | Not PO  If CL <15 mm progesterone, I: 204 (43.9%) C: 219  (46.9%) | + | ? | ? |
| Pacagnella 2022  Brazil | Singletons 92.4%  Twins 7.6% | All DA CL <30 mm | 936  I: 475  C: 461 | Pessary (Ingamed)+vaginal progesterone Singletons  8/431 (1.9%)  RR 0.44 (95% CI 0.19-1.01)  p= 0.04  Multifetal 2/43 (4.7%)  RR 0.19 (95% CI 0.04-0.83)  p= 0.15 | Vaginal progesterone  Singletons 18/430 (4.2%)  Multifetal 7/28 (25.0%) | Not PO | ? | ? | ? |
| Saccone 2017c  Italy | Singletons | TVS CL ≤25 mm Prior cervical surgery I: 7 (4.7%)  C: 5 (3.3%) | 300  I: 150  C: 150 | Pessary (Arabin) 7/150 (4.7%)  RR 0.78 (95% CI 0.3-2.03) p=0.8 | Standard care 9/150 (6.0%) | Not PO  If CL <20 mm progesterone, I: 133 (88.7%) C: 125  (83.3%) | ? | ? | ? |
| Berghella 2017a USA PoPPT | Twins | TVS CL ≤30 mm DCDA 78%  MCDA 22%  Previous PTB 0 vs 13% | 46  I: 23  C: 23 | Pessary (Biotech cup) 4/23 (17%)  RR 1.00 (95% CI 0.28-3.52) p=1.00 | No pessary 4/23 (17%) | Not PO | + | ? | - |

Prevention of preterm birth

STable 4.3.4.a. cont. Intervention pessary

Outcome variable: Any preterm birth before 28 gestational weeks

* + No or minor problems

? Some problems

- Major problems

| **Author, year Country**  **Trial acronym** | **Singletons/ Twins/ Triplets** | **Risk factor** | **Number of**  **randomized patients**  **n=** | **Results** | | **Comments** | **Directness** | **Study limitations** | **Precision *** |
| --- | --- | --- | --- | --- | --- | --- | --- | --- | --- |
|  |  |  |  | **Intervention** | **Control** |  |  |  |  |
|  |  |  |  |  |  |  |  |  |  |
| Liem 2013a  The Netherlands ProTWIN | Twins Triplets | Triplets I: 2% (n=9)  C: 2% (n=9)  MC twins I: 22%  C: 24%  Previous PTB 7 vs 6% | 813  I: 403  C: 410 | Pessary (Arabin) 16/401 (4%)  RR 0.79 (95% CI 0.50-1.27)  No p-value | Standard care 21/407 (5%) | Not PO  I: 5 cerclage  C: 0 cerclage | + | ? | ? |
| Nicolaides 2016a  12 countries | Twins | MC twins: I: 18.8%  C: 18.8%  Previous PTB 8.8 vs 14.3% | 1180  I: 590  C: 590 | Pessary (Arabin) 19/588 (3.2%)  RR 1.269 (95% CI 0.651-2.473)  No p-value | Standard care 15/589 (2.5%) | Not PO | + | ? | + |
| Norman 2021 United Kingdom,  Belgium STOPPIT-2 | Twins | MCDA 20%  DCDA 80%  CL <35 mm | 503  I: 250  C: 253 | Pessary (Arabin) 17/250 (6.8%)  OR 0.67 (95% CI 0.27-1-64) p=0.25 | Standard care 24/253 (9.5%) | Not PO | + | ? | ? |

C; control, CI; confidence interval, CL; cervical length, Cx; cervix, DA; diamniotic, DCDA; dichorionic diamniotic, I; intervention, MC; monochorionic, MCDA; monochorionic diamniotic, OR; odds ratio, PO; primary outcome, PTB; preterm birth, RR; risk ratio, TVS; transvaginal sonography

* + No or minor problems

? Some problems

- Major problems

Prevention of preterm birth

STable 4.3.4.b. cont. Intervention pessary

Outcome variable: Spontaneous preterm birth before 28 gestational weeks

| **Author, year Country**  **Trial acronym** | **Singletonss/ Twins/ Triplets** | **Risk factor** | **Number of**  **randomized patients**  **n=** | **Results** | | **Comments** | **Directness *** | **Study limitations *** | **Precision *** |
| --- | --- | --- | --- | --- | --- | --- | --- | --- | --- |
|  |  |  |  | **Intervention** | **Control** |  |  |  |  |
|  |  |  |  |  |  |  |  |  |  |
| Dugoff 2018 USA PoPPS | Singletons | TVS CL <25 mm | 122  I: 61  C: 61 | Pessary (Biotech cup) 11/60 (18.3%)  RR 0.89 (95% CI 0.43-1.85) p=0.82 | No pessary 12/58 (20.7%) | Not PO  If CL <20 mm progesterone, I: 84% C: 91% | ? | ? | - |
| Goya 2012  Spain PECEP | Singletons | TVS CL <25 mm  11% in both groups had previous PTB | 385  I: 192  C: 193 | Pessary (Arabin) 4/190 (2%)  OR 0.23 (95% CI 0.06-0.74) p=0.0058 | Standard care 16/190 (8%) | Not PO | ? | ? | + |
| Pacagnella 2022  Brazil | Singletons 92.4%  Twins 7.6% | All DA CL <30 mm | 936  I:475 C:461 | Pessary (Ingamed)+vaginal progesterone  4/461 (0.9%)  RR 0.21 (95% CI 0.07-0.63)  No p-value | Vaginal progesterone 18/435 (4.1%) | PO | ? | ? | ? |
| Saccone 2017c  Italy | Singletons | TVS CL ≤25 mm Prior cervical surgery I: 7 (4.7%)  C: 5 (3.3%) | 300  I: 150  C: 150 | Pessary (Arabin) 6/150 (4.0%)  RR 0.67 (95% CI 0.24-1.83) p=0.6 | Standard care 9/150 (6.0%) | Not PO  If CL <20 mm progesterone, I: 133 (88.7%) C: 125  (83.3%) | ? | ? | ? |
| Berghella 2017a USA PoPPT | Twins | TVS CL ≤30 mm DCDA 78%  MCDA 22%  Previous PTB 0 vs 13% | 46  I: 23  C: 23 | Pessary (Biotech cup) 4/23 (17%)  RR 1.00 (95% CI 0.28-3.52) p=1.00 | No pessary 4/23 (17%) | Not PO | + | ? | - |
| Goya 2016  Spain  PECEP-Twins | Twins | TVS CL ≤25 mm Previous PTB  I: 16.7%  C: 17.6%  MC twins I: 19.1%  C: 17.6% | 137  I: 68  C: 66 | Pessary (Arabin) 4/68 (5.9%)  RR 0.43 (95% CI 0.14-1.33)  No p-value | Standard care 9/66 (13.6%) | Not PO | + | ? | + |

* + No or minor problems

? Some problems

- Major problems

Prevention of preterm birth

STable 4.3.4.b. cont. Intervention pessary

Outcome variable: Spontaneous preterm birth before 28 gestational weeks

| **Author, year Country**  **Trial acronym** | **Singletons/ Twins/ Triplets** | **Risk factor** | **Number of**  **randomized patients**  **n=** | **Results** | | **Comments** | **Directness *** | **Study limitations *** | **Precision *** |
| --- | --- | --- | --- | --- | --- | --- | --- | --- | --- |
|  |  |  |  | **Intervention** | **Control** |  |  |  |  |
|  |  |  |  |  |  |  |  |  |  |
| Norman 2021 United Kingdom,  Belgium STOPPIT-2 | Twins | MCDA 20%  DCDA 80%  CL <35 mm | 503  I: 250  C: 253 | Pessary (Arabin) 13/250 (5.2%)  OR 0.64 (95% CI 0.23-1.77) p=0.26 | Standard care 19/253 (7.5%) | Not PO | + | ? | ? |

C; control, CI; confidence interval, CL; cervical length, DA; diamniotic, DCDA; dichorionic diamniotic, I; intervention, MC; monochorionic, MCDA; monochorionic diamniotic, OR; odds ratio, PTB; preterm birth, RR; risk ratio, TVS; transvaginal sonography

* + No or minor problems

? Some problems

- Major problems

Prevention of preterm birth

STable 4.3.5 Intervention pessary

Outcome variable: Gestational age at delivery (weeks)

| **Author, year Country**  **Trial acronym** | **Singletons/ Twins/ Triplets** | **Risk factor** | **Number of**  **randomized patients**  **n=** | **Results** | | **Comments** | **Directness *** | **Study limitations**  ***** | **Precision *** |
| --- | --- | --- | --- | --- | --- | --- | --- | --- | --- |
|  |  |  |  | **Intervention Pessary** | **Control**  **No pessary/Standard care**  **Mean (SD)** |  |  |  |  |
|  |  |  |  |  |  |  |  |  |  |
| Dugoff 2018 USA PoPPS | Singletons | TVS CL <25 mm | 122  I: 61  C: 61 | Pessary (Biotech cup) Median (IQR) 37.2 (30.0-39.1)  p=0.99 | No pessary Median (IQR) 38.1 (27.8-39.4) | Not PO  If CL <20 mm progesterone, I: 84% C: 91% | ? | ? | - |
| Goya 2012  Spain PECEP | Singletons | TVS CL <25 mm  11% in both groups had previous PTB | 385  I: 192  C: 193 | Pessary (Arabin) Mean (SD) 37.7 (2.0)  p<0001 | Standard care Mean (SD) 34.9 (4.0) | Not PO | ? | ? | + |
| Hui 2013  China | Singletons | TVS CL <25 mm | 108  I: 53  C: 55 | Pessary (Arabin) Mean (SD) 38.1 (3.4)  p=0.68 | Digital examination at entry to simulate pessary insertion Mean (SD) 37.8 (3.9) | Not PO | + | ? | - |
| Karbasian 2016  Iran | Singletons | TVS CL <25 mm | 146  I: 73  C: 73 | Pessary (Arabin) + vaginal progesterone  Mean (SD) 37 ± 3.6  No p-value | Vaginal progesterone Mean (SD)  37.1 ± 4 | Not PO | + | ? | - |
| Nicolaides 2016b  9 countries | Singletons | TVS CL <25 mm | 935  I: 466  C: 469 | Pessary (Arabin) Median (IQR) 38.9 (37.0-40.0) p=0.4 | Standard care Median (IQR) 38.7 (37.1-39.9) | Not PO  If CL <15 mm progesterone, I: 204 (43.9%) C: 219  (46.9%) | + | ? | ? |
| Pacagnella 2022  Brazil | Singletons 92.4%  Twins 7.6% | All DA CL <30 mm | 936  I: 475  C: 461 | Pessary (Ingamed)+vaginal progesterone  Mean (SD) 37.4 (3.2) | Vaginal progesterone  Mean (SD) 36.9 (4.0) | Not PO | ? | ? | ? |
| Saccone 2017c  Italy | Singletons | TVS CL ≤25 mm Prior cervical surgery I: 7 (4.7%)  C: 5 (3.3%) | 300  I: 150  C: 150 | Pessary (Arabin) Mean 37.6 (3.44) p=0.001 | Standard care Mean (SD) 36.2 (4.37) | Not PO  If CL <20 mm progesterone, I: 133 (88.7%) C: 125  (83.3%)  SD calculated from data | ? | ? | ? |

* + No or minor problems

? Some problems

- Major problems

Prevention of preterm birth

STable 4.3.5 cont. Intervention pessary

Outcome variable: Gestational age at delivery (weeks)

| **Author, year Country**  **Trial acronym** | **Singletons/ Twins/ Triplets** | **Risk factor** | **Number of**  **randomized patients**  **n=** | **Results** | | **Comments** | **Directness *** | **Study limitations**  ***** | **Precision *** |
| --- | --- | --- | --- | --- | --- | --- | --- | --- | --- |
|  |  |  |  | **Intervention Pessary** | **Control**  **No pessary/Standard care**  **Mean (SD)** |  |  |  |  |
|  |  |  |  |  |  |  |  |  |  |
| Berghella 2017a  USA PoPPT | Twins | TVS CL ≤30 mm | 46  I: 23  C: 23 | Pessary (Biotech cup) Median (IQR)  35.9 (28.9-36.9) p=0.83 | No pessary Median (IQR) 35.0 (33.0-36.7) | Not PO | + | ? | - |
| Goya 2016  Spain  PECEP-Twins | Twins | TVS CL ≤25 mm Previous PTB  I: 16.7%  C: 17.6%  MC twins I: 19.1%  C: 17.6% | 137  I: 68  C: 66 | Pessary (Arabin)  35.3 (2.9 range) p=0.01 | Standard care  33.1 (3.9 range) | Not PO Unclear if median | + | ? | + |
| Liem 2013a  The Netherlands ProTWIN | Twins Triplets (2%) | Triplets I: 2% (n=9)  C: 2% (n=9)  MC twins I: 22%  C: 24% | 813  I: 403  C: 410 | Pessary (Arabin) Median (IQR) 36.7 (34.7-37.4)  HR 0.91 (95% CI 0.76-1.09)  No p-value | Standard care Median (IQR) 36.4 (34.3-37.6) | Not PO | + | ? | ? |
| Nicolaides 2016a  12 countries | Twins | MC twins: I: 18.8%  C: 18.8% | 1180  I: 590  C: 590 | Pessary (Arabin) Median (IQR) 36.6 (34.9-37.9)  No p-value | Standard care Median (IQR) 36.7 (35.0-37.9) | Not PO | + | ? | + |
| Norman 2021 United Kingdom, Belgium STOPPIT-2 | Twins | MCDA 20%  DCDA 80%  CL <35 mm | 503  I: 250  C: 253 | Pessary (Arabin) Mean (SD) 34.8 (3.7)  Mean difference  0.2 (-0.6-1.1) p=0.5 | Standard care Mean (SD) 34.5 (4.0) | Not PO | + | ? | ? |

C; control, CI; confidence interval, CL; cervical length, DA; diamniotic, DCDA; dichorionic diamniotic, HR; hazard ratio, I; intervention, IQR; interquartile range, MC; monochorionic, MCDA; monochorionic diamniotic, PO; primary outcome, PTB; preterm birth, SD; standard deviation, TVS; transvaginal sonography

* + No or minor problems

? Some problems

- Major problems

Prevention of preterm birth

STable 4.3.6 Intervention pessary

Outcome variable: Low birth weight (<2500g)

| **Author, year Country**  **Trial acronym** | **Singletons/ Twins/ Triplets** | **Risk factor** | **Number of**  **randomized patients**  **n=** | **Results** | | **Comments** | **Directness *** | **Study limitations *** | **Precision *** |
| --- | --- | --- | --- | --- | --- | --- | --- | --- | --- |
|  |  |  |  | **Intervention Pessary** | **Control Standard care** |  |  |  |  |
|  |  |  |  |  |  |  |  |  |  |
| Goya 2012  Spain PECEP | Singletons | TVS CL <25 mm  11% in both groups had previous PTB | 385  I: 192  C: 193 | Pessary (Arabin) 17/190 (9%)  OR 0.23 (95% CI 0.12-0.43) p=<0.0001 | Standard care 56/190 (29%) | Not PO | ? | ? | + |
| Karbasian 2016  Iran | Singletons | TVS CL <25 mm | 146  I: 73  C: 73 | Pessary+vaginal progesterone 17/71 (23.9%)  p=0.36 | Vaginal progesterone 13/73 (17.8%) | Not PO | + | ? | - |
| Nicolaides 2016b  9 countries | Singletons | TVS CL <25 mm | 935  I: 466  C: 469 | Pessary (Arabin) 96/465 (20.6%)  OR 1.15 (95% CI 0.83-1.59) p=0.39 | Standard care 86/467 (18.4%) | Not PO  If CL <15 mm progesterone, I: 204 (43.9%) C: 219  (46.9%) | + | ? | ? |
| Saccone 2017c  Italy | Singleton | TVS CL ≤25 mm Prior cervical surgery  I: 7 (4.7%)  C: 5 (3.3%) | 300  I: 150  C: 150 | Pessary (Arabin) 28/150 (18.7%)  RR 0.62 (95% CI 0.41-0.94) p=0.03 | Standard care 45/150 (30.0%) | Not PO  If CL <20 mm progesterone, I: 133 (88.7%) C: 125  (83.3%) | ? | ? | ? |
| Goya 2016  Spain  PECEP-Twins | Twins | TVS CL ≤25 mm Previous PTB  I: 16.7%  C: 17.6%  MC twins I: 19.1%  C: 17.6% | 137  I: 68  C: 66 | Pessary (Arabin) 47/136 (34.6%)  RR 0.72 (95% CI 0.54-0.97) p=0.01 | Standard care 62/130 (47.7%) | Not PO | + | ? | + |

* + No or minor problems

? Some problems

- Major problems

Prevention of preterm birth

STable 4.3.6 cont. Intervention pessary

Outcome variable: Low birth weight (<2500g)

| **Author, year Country**  **Trial acronym** | **Singletons/ Twins/ Triplets** | **Risk factor** | **Number of**  **randomized patients**  **n=** | **Results** | | **Comments** | **Directness *** | **Study limitations *** | **Precision *** |
| --- | --- | --- | --- | --- | --- | --- | --- | --- | --- |
|  |  |  |  | **Intervention Pessary** | **Control Standard care** |  |  |  |  |
|  |  |  |  |  |  |  |  |  |  |
| Nicolaides 2016a  12 countries | Twins | MC twins: I: 18.8%  C: 18.8% | 1180  I: 590  C: 590 | Pessary (Arabin) Pregnancy level 395/588 (67.2%)  RR 0.972 (95% CI 0.899-1.051)  No p-value Neonatal level 664/1176 (56.5%)  RR 0.993 (95% CI 0.925-1.065)  No p-value | Standard care Pregnancy level 407/589 (69.1%)  Neonatal level 670/1178 (56.9%) | Not PO | + | ? | + |
| Liem 2013a  The Netherlands ProTWIN | Twins Triplets | Triplets I: 2% (n=9)  C: 2% (n=9)  MC twins I: 22%  C: 24% | 813  I: 403  C: 410 | Pessary (Arabin) Pregnancy level 271/401 (68%)  RR 0.99 (95%CI 0.9-1.09)  No p-value Neonatal level 442/811 (55%)  RR 0.96 (95% CI 0.86-1.06)  No p-value | Standard care Pregnancy level 275/407 (68%)  Neonatal level 466/823 (57%) | Not PO | + | ? | ? |

C; control, CI; confidence interval, CL; cervical length, I; intervention, MC; monochorionic, OR; odds ratio, PO; primary outcome, RR; relative risk, PTB; preterm birth, TVS; transvaginal sonography

* + No or minor problems

? Some problems

- Major problems

Prevention of preterm birth

STable 4.3.7 Intervention pessary

Outcome variable: Very low birth weight (<1500g)

| **Author, year Country**  **Trial acronym** | **Singletons/ Twins/ Triplets** | **Risk factor** | **Number of**  **randomized patients**  **n=** | **Results** | | **Comments** | **Directness *** | **Study limitations *** | **Precision *** |
| --- | --- | --- | --- | --- | --- | --- | --- | --- | --- |
|  |  |  |  | **Intervention Pessary** | **Control Standard care** |  |  |  |  |
|  |  |  |  |  |  |  |  |  |  |
| Goya 2012  Spain PECEP | Singletons | TVS CL <25 mm  11% in both groups had previous PTB | 385  I: 192  C: 193 | Pessary (Arabin) 9/190 (5%)  OR 0.31 (95% CI 0.13-0.72) p=0.004 | Standard care 26/190 (14%) | Not PO | ? | ? | + |
| Nicolaides 2016b  9 countries | Singletons | TVS CL <25 mm | 935  I: 466  C: 469 | Pessary (Arabin) 39/465 (8.4%)  OR 1.44 (95% CI 0.87-2.37) p=0.16 | Standard care 28/467 (6.0%) | Not PO  If CL <15 mm progesterone, I: 204 (43.9%) C: 219  (46.9%) | + | ? | ? |
| Saccone 2017c  Italy | Singletons | TVS CL ≤25 mm Prior cervical surgery  I: 7 (4.7%)  C: 5 (3.3%) | 300  I: 150  C: 150 | Pessary (Arabin) 10/150 (6.7%)  RR 0.67 (95% CI 0.31-1.44) p=0.40 | Standard care 15/150 (10.0%) | Not PO  If CL <20 mm progesterone, I: 133 (88.7%) C: 125  (83.3%) | ? | ? | ? |
| Goya 2016  Spain  PECEP-Twins | Twins | TVS CL ≤25 mm Previous PTB  I: 16.7%  C: 17.6%  MC twins I: 19.1%  C: 17.6% | 137  I: 68  C: 66 | Pessary (Arabin) 13/136 (9.5%)  RR 0.73 (95% CI 0.37-1.44)  No p-value | Standard care 17/130 (13.1%) | Not PO | + | ? | + |

* + No or minor problems

? Some problems

- Major problems

Prevention of preterm birth

STable 4.3.7 cont. Intervention pessary

Outcome variable: Very low birth weight (<1500g)

| **Author, year Country**  **Trial acronym** | **Singletons/ Twins/ Triplets** | **Risk factor** | **Number of**  **randomized patients**  **n=** | **Results** | | **Comments** | **Directness *** | **Study limitations *** | **Precision *** |
| --- | --- | --- | --- | --- | --- | --- | --- | --- | --- |
|  |  |  |  | **Intervention Pessary** | **Control Standard care** |  |  |  |  |
|  |  |  |  |  |  |  |  |  |  |
| Nicolaides 2016a  12 countries | Twins | MC twins: I: 18.8%  C: 18.8% | 1180  I: 590  C: 590 | Pessary (Arabin) Pregnancy level 60/588 (10.2%)  RR 0.925 (95% CI 0.664-1.288)  No p-value Fetal level  100/1176 (8.5%)  RR 1.043 (95% CI 0.798-1.364)  No p-value | Standard care Pregnancy level 65/589 (11.0%)  Fetal level 96/1178 (8.1%) | Not PO | + | ? | + |
| Liem 2013a  The Netherlands | Twins Triplets | Triplets I: 2% (n=9)  C: 2% (n=9)  MC twins I: 22%  C: 24% | 813  I: 403  C: 410 | Pessary (Arabin) Pregnancy level 49/401 (12%)  RR 0.93 (95% CI 0.65-1.35)  No p-value Fetal level 82/811 (10%)  RR 0.95 (95% CI 0.65-1.41)  No p-value | Standard care Pregnancy level 53/407 (13%)  Fetal level 86/823 (10%) | Not PO | + | ? | ? |

C; control, CI; confidence interval, CL; cervical length, I; intervention, MC; monochorionic, OR; odds ratio, PO; primary outcome, PTB; preterm birth, RR; relative risk, TVS; transvaginal sonography

* + No or minor problems

? Some problems

- Major problems

Prevention of preterm birth

STable 4.3.8 Intervention pessary

Outcome variable: Perinatal mortality

| **Author, year Country**  **Trial acronym** | **Singletons/ Twins/ Triplets** | **Risk factor** | **Number of**  **randomized patients**  **n=** | **Results** | | **Comments** | **Directness *** | **Study limitations *** | **Precision *** |
| --- | --- | --- | --- | --- | --- | --- | --- | --- | --- |
|  |  |  |  | **Intervention Pessary** | **Control Standard care** |  |  |  |  |
|  |  |  |  |  |  |  |  |  |  |
| Karbasian 2016  Iran | Singletons | TVS CL <25 mm | 146  I: 73  C: 73 | Pessary+vaginal progesterone 2/71 (2.8%) | Vaginal progesterone 2/73 (2.7%) | Not PO  PNM includes NNM (not defined)  + IUFD (not defined) + stillbirth | + | ? | - |
| Nicolaides 2016b  9 countries | Singletons | TVS CL <25 mm | 935  I: 466  C: 469 | Pessary (Arabin) 15/465 (3.2%)  OR 1.38 (95% CI 0.63-3.04) p=0.42 | Standard care 11/467 (2.4%) | Not PO  PNM includes NNM (not defined)  + IUFD (not defined) + termination of pregnancy (I=0 C=1)  If CL <15 mm progesterone, I: 204 (43.9%) C: 219 (46.9%) | + | ? | ? |
| Pacagnella 2022  Brazil | Singletons 92.4%  Twins 7.6% | All DA CL <30 mm | 936  I: 475  C: 461 | Pessary (Ingamed)+vaginal progesterone  Fetal level 15/509 (2.9%) | Vaginal progesterone  Fetal level 31/468 (6.6%) | Not PO  PNM includes NNM ( not defined) + stillbirth (not defined) | ? | ? | ? |
| Saccone 2017c  Italy | Singletons | TVS CL ≤25 mm Prior cervical surgery  I: 7 (4.7%)  C: 5 (3.3%) | 300  I: 150  C: 150 | Pessary (Arabin) 2/150 (1.3%)  RR 0.50 (95% CI 0.09-2.69) p=0.68 | Standard care 4/150 (2.7%) | Not PO  PNM is defined as IUFD after 20 w or NNM (<28d)  If CL <20 mm progesterone, I: 133 (88.7%) C: 125 (83.3%) | ? | ? | ? |
| Nicolaides 2016a  12 countries | Twins | MC twins: I: 18.8%  C: 18.8% | 1180  I: 590  C: 590 | Pessary (Arabin) Maternal level 20/588 (3.4%)  RR 0.911 (95% CI 0.502-1.651)  No p-value  Neonatal level 29/1176 (2.5%)  RR 0.908 (95% CI 0.553-1.491)  No p-value | Standard care Maternal level 22/589 (3.7%)  Neonatal level 32/1178 (2.7%) | Not PO  PNM includes NNM (not defined)+ IUFD (not defined) | + | ? | + |
| Norman 2021 United Kingdom, Belgium STOPPIT-2 | Twins | MCDA 20%  DCDA 80%  CL <35 mm | 503  I: 250  C: 253 | Pessary (Arabin) Fetal level 4/500 (0.8%)  OR 0.49 (95% CI 0.07-3.25) p=0.33 | Standard care Fetal level 8/506 (1.6%) | Not PO  Fetal and neonatal death within 28d | + | ? | ? |

C; control, CI; confidence interval, CL; cervical length, DA; diamniotic, DCDA; dichorionic diamniotic, I; intervention, IUFD; intrauterine fetal demise, MC; monochorionic, MCDA; monochorionic diamniotic, NNM; neonatal mortality, OR; odds ratio, PNM; perinatal mortality, PO; primary outcome, RR; risk ratio, TVS; transvaginal sonograp

Prevention of preterm birth
STable 4.3.9 Intervention pessary
Outcome variable: Neonatal mortality <28d

* + No or minor problems

? Some problems

- Major problems

| **Author, year Country**  **Trial acronym** | **Singletons/ Twins/ Triplets** | **Risk factor** | **Number of**  **randomized patients**  **n=** | **Results** | | **Comments** | **Directness *** | **Study limitations *** | **Precision *** |
| --- | --- | --- | --- | --- | --- | --- | --- | --- | --- |
|  |  |  |  | **Intervention Pessary** | **Control**  **No pessary/Standard care** |  |  |  |  |
|  |  |  |  |  |  |  |  |  |  |
| Dugoff 2018  USA PoPPS | Singletons | TVS CL <25 mm | 122  I: 61  C: 61 | Pessary (Biotech cup) 3/60 (5%)  RR 0.48 (95% CI 0.13-1.84) p=0.31 | No pessary 6/58 (10.3%) | Not PO  <28d  If CL <20 mm progesterone, I: 84% C: 91% | ? | ? | - |
| Goya 2012  Spain PECEP | Singletons | TVS CL <25 mm  11% in both groups had previous PTB | 385  I:192 C: 193 | Pessary (Arabin) 0/190 (0%)  No p-value | Standard care 1/190 (<1%) | Not PO NNM not defined | ? | ? | + |
| Hui 2013  China | Singletons | TVS CL <25 mm | 108  I: 53 C:55 | Pessary (Arabin) 1/53 (1.9%)  RR 1.02 (95% CI 0.98-1.06) p=0.49 | Digital examination at entry to simulate pessary insertion 0/55 (0%) | Not PO NNM not defined | + | ? | - |
| Karbasian 2016  Iran | Singletons | TVS CL <25 mm | 146  I:73 C:73 | Pessary+vaginal progesterone 1/71 (1.4%)  p=1.0 | Vaginal progesterone 1/73 (1.3%) | Not PO NNM not defined | + | ? | - |
| Nicolaides 2016b  9 countries | Singletons | TVS CL <25 mm | 935  I: 466  C: 469 | Pessary (Arabin) 7/465 (1.5%)  OR 1.41 (95% CI 0.45-4.48) p=0.56 | Standard care 5/467 (1.1%) | Not PO NNM not defined  If CL <15 mm progesterone, I: 204  (43.9%) C: 219 (46.9%) | + | ? | ? |
| Pacagnella 2022  Brazil | Singletons 92.4%  Twins 7.6% | All DA CL <30 mm | 936  I:475 C:461 | Pessary (Ingamed)+vaginal progesterone  Fetal level 11/499 (2.2%)  RR 0.52 (95% CI 023-1.17) | Vaginal progesterone  Fetal level 19/450 (4.2%) | NNM not defined Not PO | ? | ? | ? |
| Saccone 2017c  Italy | Singletons | TVS CL ≤25 mm Prior cervical surgery I: 7 (4.7%)  C: 5 (3.3%) | 300  I: 150  C: 150 | Pessary (Arabin) 1/150 (0.7%)  RR 0.33 (95% CI 0.04-3.17) p=0.61 | Standard care 3/150 (2.0%) | Not PO  <28d  If CL <20 mm progesterone, I: 133 (88.7%) C: 125  (83.3%) | ? | ? | ? |

Prevention of preterm birth
STable 4.3.9 cont. Intervention pessary Outcome variable: Neonatal mortality <28d

* + No or minor problems

? Some problems

- Major problems

| **Author, year Country**  **Trial acronym** | **Singletons/ Twins/ Triplets** | **Risk factor** | **Number of**  **randomized patients**  **n=** | **Results** | | **Comments** | **Directness *** | **Study limitations *** | **Precision *** |
| --- | --- | --- | --- | --- | --- | --- | --- | --- | --- |
|  |  |  |  | **Intervention Pessary** | **Control**  **No pessary/Standard care** |  |  |  |  |
|  |  |  |  |  |  |  |  |  |  |
| Berghella 2017a USA PoPPT | Twins | TVS CL ≤30 mm | 46  I: 23  C: 23 | Pessary (Biotech cup) Neonatal level  4/46 (9%)  RR 1.33 (95% CI 0.32-5.63) p=1.0 | No pessary Neonatal level 3/46 (7%) | Not PO  <28d | + | ? | - |
| Goya 2016  Spain  PECEP-Twins | Twins | TVS CL ≤25 mm Previous PTB  I: 16.7%  C: 17.6%  MC twins I: 19.1%  C: 17.6% | 137  I: 68  C: 66 | Pessary (Arabin) Neonatal level 0/136  No p-value | Standard care Neonatal level 0/130 | Not PO NNM not defined | + | ? | + |
| Nicolaides 2016a  12 countries | Twins | MC twins: I: 18.8%  C: 18.8% | 1180  I: 590  C: 590 | Pessary (Arabin) Maternal level 13/588 (2.2%)  RR 1.447 (95% CI 0.623-3.359)  No p-value Neonatal level 17/1176 (1.4%)  RR 1.216 (95% CI 0.602-2.456)  No p-value | Standard care Maternal level 9/589 (1.5%)  Neonatal level 14/1178 (1.2%) | Not PO NNM not defined | + | ? | + |
| Liem 2013a  The Netherlands ProTWIN | Twins Triplets (2%) | Triplets I: 2% (n=9)  C: 2% (n=9)  MC twins I: 22%  C: 24% | 813  I: 403  C: 410 | Pessary (Arabin) Maternal level 16/401 (4%)  RR 0.90 (95% CI 0.46-1.77)  No p-value Neonatal level 23/811 (3%)  RR 0.83 (95% CI 0.41-1.68)  No p-value | Standard care Maternal level 18/407 (4%)  Neonatal level 28/823 (3%) | Not PO  NNM defined as death before discharge | + | ? | ? |

C; control, CI; confidence interval, CL; cervical length, DA; diamniotic, I; intervention, MC; monochorionic, NNM; neonatal mortality, OR; odds ratio, PO; primary outcome, PTB; preterm birth, RR; risk ratio, TVS; transvaginal sonography

Prevention of preterm birth

* + No or minor problems

? Some problems

- Major problems

STable 4.3.10 Intervention pessary

Outcome variable: Composite adverse neonatal outcome

| **Author, year Country**  **Trial acronym** | **Singletons/ Twins/ Triplets** | **Risk factor** | **Number of**  **randomized patients**  **n=** | **Results** | | **Comments** | **Directness *** | **Study limitations *** | **Precision *** |
| --- | --- | --- | --- | --- | --- | --- | --- | --- | --- |
|  |  |  |  | **Intervention** | **Control** |  |  |  |  |
|  |  |  |  |  |  |  |  |  |  |
| Dugoff 2018 USA PoPPS | Singletons | TVS CL <25 mm | 122  I: 61  C: 61 | Pessary (Biotech cup) 12/60 (20%)  RR 1.16 (95% CI 0.54-2.47) p=0.67 | No pessary 10/58 (17.2%) | Not PO  NEC, IVH (grade 3 or 4), RDS, BPD,  ROP, blood culture-proven sepsis or NNM.  If CL <20 mm progesterone, I: 84% C: 91% | ? | ? | - |
| Goya 2012  Spain PECEP | Singletons | TVS CL <25 mm  11% in both groups had previous PTB | 385  I: 192  C: 193 | Pessary (Arabin) 5/190 (3%)  OR 0.14 (95% CI 0.04-0.39) p<0.0001 | Standard care 30/190 (16%) | Not PO  NEC, IVH, RDS, ROP, treatment for sepsis | ? | ? | + |
| Nicolaides 2016b  9 countries | Singletons | TVS CL <25 mm | 935  I: 466  C: 469 | Pessary (Arabin) 30/450 (6.7%)  OR 1.18 (95% CI 0.69-2.03) p=0.55 | Standard care 26/456 (5.7%) | Not PO  IVH, RDS, NEC or ROP  If CL <15 mm progesterone, I: 204 (43.9%) C: 219 (46.9%) | + | ? | ? |
| Pacagnella 2022  Brazil | Singletons 92.4%  Twins 7.6% | All DA CL <30 mm | 936  I: 475  C: 461 | Pessary (Ingamed)+vaginal progesterone  Fetal level 98/503 (19.4%)  RR 0.9 (95% CI 0.69-1.17) | Vaginal progesterone  Fetal level 100/461 (21.7%) | PO  Periventricular leukomalacia, severe RDS, BPD, periventricular hemorrhage grade II or higher, NEC, sepsis, stillbirth, neonatal death. | ? | ? | ? |
| Saccone 2017c  Italy | Singletons | TVS CL ≤25 mm Prior cervical surgery  I: 7 (4.7%)  C: 5 (3.3%) | 300  I: 150  C: 150 | Pessary (Arabin) 22/150 (14.7%)  RR 0.46 (95% CI 0.29-0.72) p=0.01 | Standard care 48/150 (32%) | Not PO  NEC, IVH (grade 3 or 4), RDS, BPD,  ROP requiring therapy, blood culture- proven sepsis or NNM  If CL <20 mm progesterone, I: 133 (88.7%) C: 125 (83.3%) | ? | ? | ? |
| Berghella 2017a USA PoPPT | Twins | TVS CL ≤30 mm | 46  I: 23  C: 23 | Pessary (Biotech cup) Fetal level  21/46 (46%)  RR 1.62 (95% CI 0.92-2.82) p=0.13 | No pessary Fetal level 13/46 (28%) | Not PO  NEC, IVH (grade 3 or 4), RDS, BPD,  ROP, blood culture-proven sepsis or NNM | + | ? | - |

Prevention of preterm birth
STable 4.3.10 cont. Intervention pessary

* + No or minor problems

? Some problems

- Major problems

Outcome variable: Composite adverse neonatal outcome

| **Author, year Country**  **Trial acronym** | **Singletons/ Twins/ Triplets** | **Risk factor** | **Number of**  **randomized patients**  **n=** | **Results** | | **Comments** | **Directness *** | **Study limitations *** | **Precision *** |
| --- | --- | --- | --- | --- | --- | --- | --- | --- | --- |
|  |  |  |  | **Intervention** | **Control** |  |  |  |  |
|  |  |  |  |  |  |  |  |  |  |
| Goya 2016  Spain  PECEP-Twins | Twins | TVS CL ≤25 mm Previous PTB  I: 16.7%  C: 17.6%  MC twins I: 19.1%  C: 17.6% | 137  I: 68  C: 66 | Pessary (Arabin) Fetal level 8/136 (5.9%)  RR 0.64 (95% CI 0.27-1.5)  No p-value | Standard care Fetal level 12/130 (9.1%) | Not PO  NEC, IVH, RDS, BPD, ROP recuiring therapy, proven or suspected sepsis | + | ? | + |
| Liem 2013a  The Netherlands | Twins Triplets | Triplets I: 2% (n=9)  C: 2% (n=9)  MC twins I: 22%  C: 24% | 813  I: 403  C: 410 | Pessary (Arabin) Pregnancy level 53/401 (13%)  RR 0.98 (95% CI 0.69-1.39)  No p-value Fetal level 81/811 (10%)  RR 0.95 (95% CI 0.65-1.38)  No p-value | Standard care Pregnancy level 55/407 (14%)  Fetal level 87/823 (11%) | PO  IUFD, periventricular leukomalacia of grade 2 or worse, severe RDS of grade 2 or worse, BPD, IVH of grade 2b or worse, NEC, proven sepsis, NNM within 6 w after expected term date. | + | ? | ? |
| Nicolaides 2016a  12 countries | Twins | MC twins: I: 18.8%  C: 18.8% | 1180  I: 590  C: 590 | Pessary (Arabin) Pregnancy level 88/579 (15.2%)  RR 1.28 (95% CI 0.95-1.71)  No p-value Fetal level 115/1147 (10%)  RR 1.09 (95% CI 0.85-1.41)  No p-value | Standard care Pregnancy level 69/579 (11.9%)  Fetal level 105/1146 (9.2%) | IVH, RDS, ROP or NEC  Not PO | + | ? | + |
| Norman 2021 United Kingdom, Belgium STOPPIT-2 | Twins | MCDA 20%  DCDA 80%  CL <35 mm | 503  I: 250  C: 253 | Pessary (Arabin) Fetal level 67/500 (13.4%)  RR 0.88 (95% CI 0.6-1.31) p=0.52 | Standard care Fetal level 76/506 (15%) | PO (neonatal)  Stillbirth or neonatal death, periventricular leukomalacia, early respiratory morbidity, IVH, NEC, sepsis. | + | ? | ? |

BPD; bronchopulmonary dysplasia, C; control, CI; confidence interval, CL; cervical length, DA; diamniotic, DCDA; dichorionic diamniotic, I; intervention, IUFD; intrauterine foster death, IVH; intraventricular hemorrhage, MC; monochorionic, MCDA; monochorionic diamniotic, NEC; necrotizing enterocolitis, NNM; neonatal mortality, OR; odds ratio, PO; primary outcome, PTB; preterm birth, RDS; respiratory distress syndrome, ROP; retinopathy of prematurity, RR; risk ratio, TVS; transvaginal sonography

Prevention of preterm birth
STable 4.3.11 Intervention pessary

* + No or minor problems

? Some problems

- Major problems

Outcome variable: Respiratory distress syndrome (RDS)

| **Author, year Country**  **Trial acronym** | **Singletons/ Twins/ Triplets** | **Risk factor** | **Number of**  **randomized patients**  **n=** | **Results** | | **Comments** | **Directness *** | **Study limitations *** | **Precision *** |
| --- | --- | --- | --- | --- | --- | --- | --- | --- | --- |
|  |  |  |  | **Intervention** | **Control** |  |  |  |  |
|  |  |  |  |  |  |  |  |  |  |
| Dugoff 2018 USA PoPPS | Singletons | TVS CL <25 mm | 122  I: 61  C: 61 | Pessary (Biotech cup) 10/60 (16.7%)  RR 1.01 (95% CI 0.45-2.31) p=1.0 | No pessary 9/58 (15.5%) | Not PO  If CL <20 mm progesterone, I: 84% C: 91% | ? | ? | - |
| Goya 2012  Spain PECEP | Singletons | TVS CL <25 mm  11% in both groups had previous PTB | 385  I: 192  C: 193 | Pessary (Arabin) 5/190 (3%)  OR 0.20 (95% CI 0.06-0.55) p=0.0003 | Standard care 23/190 (12%) | Not PO | ? | ? | + |
| Hui 2013  China | Singletons | TVS CL <25 mm | 108  I: 53  C: 55 | Pessary (Arabin) 5/53 (9.4%)  RR 1.06 (95% CI 0.96-1.18) p=0.44 | Digital examination at entry to simulate pessary insertion  2/55 (3.8%) | Not PO | + | ? | - |
| Nicolaides 2016b  9 countries | Singletons | TVS CL <25 mm | 935  I: 466  C: 469 | Pessary (Arabin) 28/450 (6.2%)  OR 1.14 (95% CI 0.66-1.99)  p= 0.64 | Standard care 25/456 (5.5%) | Not PO  If CL <15 mm progesterone, I: 204  (43.9%) C: 219 (46.9%) | + | ? | ? |
| Pacagnella 2022  Brazil | Singletons 92.4%  Twins 7.6% | All DA CL <30 mm | 936  I: 475  C: 461 | Pessary (Ingamed)+vaginal progesterone  Fetal level 91/499 (18.2%)  RR 0.95 (95% CI 0.72-1.27) | Vaginal progesterone  Fetal level 86/450 (19.1%) | PO  Severe RDS | ? | ? | ? |
| Saccone 2017c  Italy | Singletons | TVS CL ≤25 mm Prior cervical surgery I: 7 (4.7%)  C: 5 (3.3%) | 300  I: 150  C: 150 | Pessary (Arabin) 14/150 (9.3%)  RR 0.45 (95% CI 0.25-0.81) p=0.01 | Standard care 31/150 (20.7%) | Not PO  If CL <20 mm progesterone, I: 133  (88.7%) C: 125 (83.3%) | ? | ? | ? |
| Berghella 2017a USA PoPPT | Twins | TVS CL ≤30 mm | 46  I: 23  C: 23 | Pessary (Biotech cup) Fetal level  11/46 (24%)  RR 1.38 (95% CI 0.61-3.10)  p= 0.61 | No pessary Fetal level 8/46 (17%) | Not PO | + | ? | - |

Prevention of preterm birth

* + No or minor problems

? Some problems

- Major problems

STable 4.3.11 cont. Intervention pessary

Outcome variable: Respiratory distress syndrome (RDS)

| **Author, year Country**  **Trial acronym** | **Singletons/ Twins/ Triplets** | **Risk factor** | **Number of**  **randomized patients**  **n=** | **Results** | | **Comments** | **Directness *** | **Study limitations *** | **Precision *** |
| --- | --- | --- | --- | --- | --- | --- | --- | --- | --- |
|  |  |  |  | **Intervention** | **Control** |  |  |  |  |
|  |  |  |  |  |  |  |  |  |  |
| Goya 2016  Spain  PECEP-Twins | Twins | TVS CL ≤25 mm Previous PTB  I: 16.7%  C: 17.6%  MC twins I: 19.1%  C: 17.6% | 137  I: 68  C: 66 | Pessary (Arabin) Fetal level 8/136 (5.9%)  RR 0.96 (95% CI 0.37-2.47)  No p-value | Standard care Fetal level 8/130 (6.1%) | Not PO | + | ? | + |
| Nicolaides 2016a  12 countries | Twins | MC twins: I: 18.8%  C: 18.8% | 1180  I: 590  C: 590 | Pessary (Arabin) Pregnancy level 84/579 (14.5%)  RR 1.25 (95% CI 0.93-1.69)  No p-value Fetal level  109/1147 (9,5%)  RR 1.09 (95% CI 0.84-1.41)  No p-value | Standard care Pregnancy level 67/579 (11.6%)  Fetal level 100/1146 (8.7%) | Not PO | + | ? | + |
| Liem 2013a  The Netherlands ProTWIN | Twins Triplets | Triplets I: 2% (n=9)  C: 2% (n=9)  MC twins I: 22%  C: 24% | 813  I: 403  C: 410 | Pessary (Arabin) Pregnancy level 27/401 (7%)  RR 1.52 (95% CI 0.85-2.72)  No p-value Fetal level 36/811 (4%)  RR 1.26 (95% CI 0.67-2.35)  No p-value | Standard care Pregnancy level 18/407 (4%)  Fetal level 29/823 (4%) | Not PO | + | ? | ? |

C; control, CI; confidence interval, CL; cervical length, DA; diamniotic, I; intervention, MC; monochorionic, OR; odds ratio, PO; primary outcome, PTB; preterm birth, RR; risk ratio, TVS; transvaginal sonography

* + No or minor problems

? Some problems

- Major problems

Prevention of preterm birth

STable 4.3.12. Intervention pessary

Outcome variable: Bronchopulmonary dysplasia (BPD)

| **Author, year Country**  **Trial acronym** | **Singletons/ Twins/ Triplets** | **Risk factor** | **Number of**  **randomized patients**  **n=** | **Results** | | **Comments** | **Directness*** | **Study limitations** | **Precision *** |
| --- | --- | --- | --- | --- | --- | --- | --- | --- | --- |
|  |  |  |  | **Intervention** | **Control** |  |  |  |  |
|  |  |  |  |  |  |  |  |  |  |
| Dugoff 2018 USA PoPPS | Singletons | TVS CL <25 mm | 122  I: 61  C: 61 | Pessary (Biotech cup) 5/60 (8.3%)  RR 0.87 (95% CI 0.27-2.83)  p=1 | No pessary 5/58 (8.6%) | Not PO  If CL <20 mm progesterone, I: 84% C: 91% | ? | ? | - |
| Pacagnella 2022  Brazil | Singletons 92.4%  Twins 7.6% | All DA CL <30 mm | 936  I: 475  C: 461 | Pessary (Ingamed)+vaginal progesterone  Fetal level 8/497 (1.6%)  RR 0.72 (95% CI 0.25-2.08) | Vaginal progesterone  Fetal level 10/450 (2.2%) | PO | ? | ? | ? |
| Saccone 2017c  Italy | Singletons | TVS CL ≤25 mm Prior cervical surgery I: 7 (4.7%)  C: 5 (3.3%) | 300  I: 150  C: 150 | Pessary (Arabin) 8/150 (5.3%)  RR 0.67 (95% CI 0.28-1.58) p=0.49 | Standard care 12/150 (8.0%) | Not PO  If CL <20 mm progesterone, I: 133  (88.7%) C: 125 (83.3%) | ? | ? | ? |
| Berghella 2017a USA PoPPT | Twins | TVS CL ≤30 mm | 46  I: 23  C: 23 | Pessary (Biotech cup) 4/46 (9%)  RR 0.80 (95% CI 0.23-2.79) p=1.0 | No pessary 5/46 (11%) | Not PO | + | ? | - |
| Liem 2013a  The Netherlands | Twins Triplets | Triplets I: 2% (n=9)  C: 2% (n=9)  MC twins I: 22%  C: 24% | 813  I: 403  C: 410 | Pessary (Arabin) Pregnancy level 2/401 (<1%)  RR 0.34 (95% CI 0.07-1.67)  Not p-value Fetal level 2/811 (<1%)  RR 0.23 (95% CI 0.04-1.17)  No p value | Standard care Pregnancy level 6/407 (1%)  Fetal level 9/823 (1%) | PO | + | ? | ? |
| Norman 2021 United Kingdom,  Belgium STOPPIT-2 | Twins | MCDA 20%  DCDA 80%  CL <35 mm | 503  I: 250  C: 253 | Pessary (Arabin) Fetal level 6/500 (1.2%)  OR 2.0 (95% CI 0.24-16.58) p=0.4 | Standard care 3/506 (0.6%) | Not PO | + | ? | ? |

C; control, CI; confidence interval, CL; cervical length, DA; diamniotic, DCDA; dichorionic diamniotic, I; intervention, MC; monochorionic, MCDA; monochorionic diamniotic, OR; odds ratio, PO; primary outcome, RR; risk ratio, TVS; transvaginal sonography

* + No or minor problems

? Some problems

- Major problems

Prevention of preterm birth

STable 4.3.13 Intervention pessary

Outcome variable: Intraventricular hemorrhage (IVH)

| **Author, year Country**  **Trial acronym** | **Singletons/ Twins/ Triplets** | **Risk factor** | **Number of**  **randomized patients**  **n=** | **Results** | | **Comments** | **Directness** | **Study limitations** | **Precision *** |
| --- | --- | --- | --- | --- | --- | --- | --- | --- | --- |
|  |  |  |  | **Intervention** | **Control** |  |  |  |  |
|  |  |  |  |  |  |  |  |  |  |
| Dugoff 2018 USA PoPPS | Singletons | TVS CL <25 mm | 122  I: 61  C: 61 | Pessary (Biotech cup) 4/60 (6.7%)  RR 1.83 (95% CI 0.35-9.60) p=0.68 | No pessary 2/58 (3.4%) | Not PO Grade 3 or 4  If CL <20 mm progesterone, I: 84% C: 91% | ? | ? | - |
| Goya 2012  Spain PECEP | Singletons | TVS CL <25 mm  11% in both groups had previous PTB | 385  I: 192  C: 193 | Pessary (Arabin) 0/190 (0%) p=0.5 | Standard care 2/190 (1%) | Not PO Grade 2 or more | ? | ? | + |
| Hui 2013  China | Singletons | TVS CL <25 mm | 108  I: 53  C: 55 | Pessary (Arabin) 0/53 (0%)  RR 0.98 (95% CI 0.95-1.02) p=1.0 | Digital examination at entry to simulate pessary insertion 1/55 (1.9%) | Not PO Not defined | + | ? | - |
| Nicolaides 2016b  9 countries | Singletons | TVS CL <25 mm | 935  I: 466  C: 469 | Pessary (Arabin) 9/450 (2%)  OR 3.08 (95% CI 0.83-11.46) p=0.09 | Standard care 3/456 (0.7%) | Not PO  Any grade of IVH  If CL <15 mm progesterone, I: 204 (43.9%) C: 219  (46.9%) | + | ? | ? |
| Saccone 2017c  Italy | Singleton | TVS CL ≤25 mm Prior cervical surgery I: 7 (4.7%)  C: 5 (3.3%) | 300  I: 150  C: 150 | Pessary (Arabin) 4/150 (2.7%)  RR 0.67 (95% CI 0.19-2.31) p=0.75 | Standard care 6/150 (4.0%) | Not PO Grade 3 or 4  If CL <20 mm progesterone, I: 133 (88.7%) C: 125  (83.3%) | ? | ? | ? |
| Berghella 2017a USA PoPPT | Twins | TVS CL ≤30 mm | 46  I: 23  C: 23 | Pessary (Biotech cup) Fetal level  2/46 (4%)  RR 2.0 (95% CI 0.19-21.3)  p=1 | No pessary Fetal level 1/46 (2%) | Not PO Grade 3 or 4 | + | ? | - |
| Goya 2016  Spain  PECEP-Twins | Twins | TVS CL ≤25 mm Previous PTB  I: 16.7%  C: 17.6%  MC twins I: 19.1%  C: 17.6% | 137  I: 68  C: 66 | Pessary (Arabin) Fetal level 0/136 (0%)  No p-value | Standard care Fetal level 4/130 (3%) | Not PO Grade 2 or more | + | ? | + |

Cont.

* + No or minor problems

? Some problems

- Major problems

Prevention of preterm birth

STable 4.3.13 cont. Intervention pessary

Outcome variable: Intraventricular hemorrhage (IVH)

| **Author, year Country**  **Trial acronym** | **Singletons/ Twins/ Triplets** | **Risk factor** | **Number of**  **randomized patients**  **n=** | **Results** | | **Comments** | **Directness** | **Study limitations** | **Precision *** |
| --- | --- | --- | --- | --- | --- | --- | --- | --- | --- |
|  |  |  |  | **Intervention** | **Control** |  |  |  |  |
|  |  |  |  |  |  |  |  |  |  |
| Liem 2013a  The Netherlands ProTWIN | Twins Triplets | Triplets I: 2% (n=9)  C: 2% (n=9)  MC twins I: 22%  C: 24% | 813  I: 403  C: 410 | Pessary (Arabin) Pregnancy level 6/401 (1%)  RR 1.22 (95% CI 0.37-3.98)  No p-value Fetal level 8/811 (1%)  RR 1.16 (95% CI 0.33-4.07)  No p-value | Standard care Pregnancy level 5/407 (1%)  Fetal level 7/823 (1%) | PO  Grade 2B or more | + | ? | ? |
| Nicolaides 2016a  12 countries | Twins | MC twins: I: 18.8%  C: 18.8% | 1180  I: 590  C: 590 | Pessary (Arabin) Pregnancy level 16/579 (2.8%)  RR 1.33 (95% CI 0.64-2.79)  No p-value Fetal level 18/1147 (1.6%)  RR 1.2 (95% CI 0.61-2.37)  No p-value | Standard care Pregnancy level 12/579 (2.1%)  Fetal level 15/1146 (1.3%) | Not PO Not defined | + | ? | + |
| Norman 2021 United Kingdom,  Belgium STOPPIT-2 | Twins | MCDA 20%  DCDA 80%  CL <35 mm | 503  I: 250  C: 253 | Pessary (Arabin) Fetal level 9/500 (1.8%) | Standard care Fetal level 6/506 (1.2%) | PO | + | ? | ? |

C; control, CI; confidence interval, CL; cervical length, DCDA; dichorionic diamniotic, I; intervention, IVH; intraventricular hemorrhage, MC; monochorionic, MCDA; monochorionic diamniotic, OR; odds ratio, PO; primary outcome, PTB; preterm birth, RR; risk ratio, TVS; transvaginal sonography

* + No or minor problems

? Some problems

- Major problems

Prevention of preterm birth

STable 4.3.14 Intervention pessary

Outcome variable: Necrotizing enterocolitis

| **Author, year Country**  **Trial acronym** | **Singletons/ Twins/ Triplets** | **Risk factor** | **Number of**  **randomized patients**  **n=** | **Results** | | **Comments** | **Directness *** | **Study limitations *** | **Precision *** |
| --- | --- | --- | --- | --- | --- | --- | --- | --- | --- |
|  |  |  |  | **Intervention** | **Control** |  |  |  |  |
|  |  |  |  |  |  |  |  |  |  |
| Dugoff 2018  USA PoPPS | Singletons | TVS CL <25 mm | 122  I: 61  C: 61 | Pessary (Biotech cup) 2/60 (3.3%)  RR 1.83 (95% CI 0.17-19.6)  p=1 | No pessary 1/58 (1.7%) | Not PO Not defined  If CL <20 mm progesterone, I: 84% C: 91% | ? | ? | - |
| Goya 2012  Spain PECEP | Singletons | TVS CL <25 mm  11% in both groups had previous PTB | 385  I: 192  C: 193 | Pessary (Arabin) 0/190 (0%) p=0.5 | Standard care 2/190 (1%) | Not PO Not defined | ? | ? | + |
| Nicolaides 2016b  9 countries | Singletons | TVS CL <25 mm | 935  I: 466  C: 469 | Pessary (Arabin) 6/450 (1.3%)  OR 2.04 (95% CI 0.51-8.21) p=0.32 | Standard care 3/456 (0.7%) | Not PO Not defined  If CL <15 mm progesterone, I: 204 (43.9%) C: 219 (46.9%) | + | ? | ? |
| Pacagnella 2022  Brazil | Singletons 92.4%  Twins 7.6% | All DA CL <30 mm | 936  I: 475  C: 461 | Pessary (Ingamed)+vaginal progesterone Fetal level  3/497 (0.6%)  RR 0.68 (95% CI 0.15-3.01)  No p-value | Vaginal progesterone  Fetal level 4/450 (0.9%) | PO | ? | ? | ? |
| Saccone 2017c  Italy | Singleton | TVS CL ≤25 mm Prior cervical surgery I: 7 (4.7%)  C: 5 (3.3%) | 300  I: 150  C: 150 | Pessary (Arabin) 3/150 (2.0%)  RR 0.75 (95% CI 0.17-3.29) p=0.99 | Standard care 4/150 (2.7%) | Not PO Not defined  If CL <20 mm progesterone, I: 133 (88.7%) C: 125 (83.3%) | ? | ? | ? |
| Berghella 2017a USA PoPPT | Twins | TVS CL ≤30 mm | 46  I: 23  C: 23 | Pessary (Biotech cup) Child level  1/46 (2%) p=1.0 | No pessary Child level 0/46 (0%) | Not PO Not defined | + | ? | - |
| Goya 2016  Spain  PECEP-Twins | Twins | TVS CL ≤25 mm Previous PTB  I: 16.7%  C: 17.6%  MC twins I: 19.1%  C: 17.6% | 137  I: 68  C: 66 | Pessary (Arabin) Child level 0/136 (0%)  No p-value | Standard care Child level 2/130 (1.5%) | Not PO Not defined | + | ? | + |

Cont.

* + No or minor problems

? Some problems

- Major problems

Prevention of preterm birth

STable 4.3.14 cont. Intervention pessary

Outcome variable: Necrotizing enterocolitis

| **Author, year Country**  **Trial acronym** | **Singletons/ Twins/ Triplets** | **Risk factor** | **Number of**  **randomized patients**  **n=** | **Results** | | **Comments** | **Directness *** | **Study limitations *** | **Precision *** |
| --- | --- | --- | --- | --- | --- | --- | --- | --- | --- |
|  |  |  |  | **Intervention** | **Control** |  |  |  |  |

| Liem 2013a  The Netherlands | Twins Triplets | Triplets I: 2% (n=9)  C: 2% (n=9)  MC twins I: 22%  C: 24% | 813  I: 403  C: 410 | Pessary (Arabin) Pregnancy level 8/401 (2%)  RR 1.35 (95% CI 0.47-3.88)  No p value Fetal level 8/811 (1%)  RR 1.16 (95% CI 0.39-3.43)  No p-value | Standard care Pregnancy level 6/407 (1%)  Fetal level 7/823 (1%) | PO  Not defined | + | ? | ? |
| --- | --- | --- | --- | --- | --- | --- | --- | --- | --- |
| Nicolaides 2016a  12 countries | Twins | MC twins: I: 18.8%  C: 18.8% | 1180  I: 590  C: 590 | Pessary (Arabin) Pregnancy level 6/579 (1%)  RR 1.0 (95% CI 0.32-3.08)  No p value Fetal level 8/1147 (0.7%)  RR 1.33 (95% CI 0.46-3.83)  No p-value | Standard care Pregnancy level 6/579 (1.0%)  Fetal level 6/1146 (0.5%) | Not PO Not defined | + | ? | + |
| Norman 2021 United Kingdom,  Belgium STOPPIT-2 | Twins | MCDA 20%  DCDA 80%  CL <35 mm | 503  I: 250  C: 253 | Pessary (Arabin) Fetal level 2/500 (0.4%)  OR 0.20 (95% CI 0.03-1-50) p=0.04 | Standard care Fetal level 10/506 (2.0%) | PO | + | ? | ? |

C; control, CI; confidence interval, CL; cervical length, DA; diamniotic, DCDA; dichorionic diamniotic, I; intervention, MC; monochorionic, MCDA; monochorionic diamniotic, OR; odds ratio, PO; primary outcome, PTB; preterm birth, RR; risk ratio, TVS; transvaginal sonography

* + No or minor problems

? Some problems

- Major problems

Prevention of preterm birth

STable 4.3.15 Intervention pessary

Outcome variable: Neonatal sepsis

| **Author, year Country**  **Trial acronym** | **Singletons/ Twins/ Triplets** | **Risk factor** | **Number of**  **randomized patients**  **n=** | **Results** | | **Comments** | **Directness *** | **Study limitations *** | **Precision *** |
| --- | --- | --- | --- | --- | --- | --- | --- | --- | --- |
|  |  |  |  | **Intervention** | **Control** |  |  |  |  |

| Dugoff 2018 USA PoPPS | Singletons | TVS CL <25 mm | 122  I: 61  C: 61 | Pessary (Biotech cup) 7/60 (11.6%)  RR 1.08 (95% CI 0.39-3.03) p=1.0 | No pessary 6/58 (10.3%) | Not PO Proven sepsis  If CL <20 mm progesterone, I: 84% C: 91% | ? | ? | - |
| --- | --- | --- | --- | --- | --- | --- | --- | --- | --- |
| Goya 2012  Spain PECEP | Singletons | TVS CL <25 mm  11% in both groups had previous PTB | 385  I: 192  C: 193 | Pessary (Arabin) 3/190 (2%)  OR 0.24 (95% CI 0.04-0.90) p=0.03 | Standard care 12/190 (6%) | Not PO  Number of treatment for sepsis | ? | ? | + |
| Hui 2013  China | Singletons | TVS CL <25 mm | 108  I: 53  C: 55 | Pessary (Arabin) 3/53 (5.7%)  RR 0.96 (95% CI 0.86-1.07) p=0.72 | Digital examination at entry to simulate pessary insertion 5/55 (9.4%) | Not PO Clinical sepsis | + | ? | - |
| Nicolaides 2016b  9 countries | Singletons | TVS CL <25 mm | 935  I: 466  C: 469 | Pessary (Arabin) 27/450 (6%)  OR 1.39 (95% CI 0.77-2.52) p=0.28 | Standard care 20/456 (4.4%) | Not PO  Number of treatment for sepsis If CL <15 mm progesterone, I: 204  (43.9%) C: 219 (46.9%) | + | ? | ? |
| Pacagnella 2022  Brazil | Singletons 92.4%  Twins 7.6% | All DA CL <30 mm | 936  I: 475  C: 461 | Pessary (Ingamed)+vaginal progesterone  Fetal level 9/497 (1.8%)  RR 1.36 (95% CI 0.44-4.16)  No p-value | Vaginal progesterone  Fetal level 6/450 (1.3%) | PO | ? | ? | ? |
| Saccone 2017c  Italy | Singletons | TVS CL ≤25 mm Prior cervical surgery I: 7 (4.7%)  C: 5 (3.3%) | 300  I: 150  C: 150 | Pessary (Arabin) 9/150 (6.0%)  RR 0.69 (95% CI 0.31-1.57) p=0.50 | Standard care 13/150 (8.7%) | Not PO  Blood-culture proven sepsis  If CL <20 mm progesterone, I: 133 (88.7%) C: 125 (83.3%) | ? | ? | ? |
| Berghella 2017a USA PoPPT | Twins | TVS CL ≤30 mm | 46  I: 23  C: 23 | Pessary (Biotech cup) Fetal level  5/46 (11%)  RR 2.5 (95% CI 0.51-12.2) p=0.43 | No pessary Fetal level 2/46 (4%) | Not PO Proven sepsis | + | ? | - |

Cont.

* + No or minor problems

? Some problems

- Major problems

Prevention of preterm birth

STable 4.3.15 cont. Intervention pessary

Outcome variable: Neonatal sepsis

| **Author, year Country**  **Trial acronym** | **Singletons/ Twins/ Triplets** | **Risk factor** | **Number of**  **randomized patients**  **n=** | **Results** | | **Comments** | **Directness *** | **Study limitations *** | **Precision *** |
| --- | --- | --- | --- | --- | --- | --- | --- | --- | --- |
|  |  |  |  | **Intervention** | **Control** |  |  |  |  |
|  |  |  |  |  |  |  |  |  |  |
| Goya 2016  Spain  PECEP-Twins | Twins | TVS CL ≤25 mm Previous PTB  I: 16.7%  C: 17.6%  MC twins I: 19.1%  C: 17.6% | 137  I: 68  C: 66 | Pessary (Arabin) Fetal level 4/136 (2.9%)  No p-value | Standard care Fetal level 6/130 (4.6%) | Not PO  Number of treatments for sepsis | *+* | *?* | *+* |
| Liem 2013a  The Netherlands ProTWIN | Twins Triplets | Triplets I: 2% (n=9)  C: 2% (n=9)  MC twins I: 22%  C: 24% | 813  I: 403  C: 410 | Pessary (Arabin) Pregnancy level 16/401 (4%)  RR 0.89 (95% CI 0.45-1.77)  No p-value Fetal level 19/811 (2%)  RR 0.77 (95% CI 0.38-1.55)  No p-value | Standard care Pregnancy level 18/407 (4%)  Fetal level 25/823 (3%) | PO | + | ? | ? |
| Nicolaides 2016a  12 countries | Twins | MC twins: I: 18.8%  C: 18.8% | 1180  I: 590  C: 590 | Pessary (Arabin) Pregnancy level 41/579 (7.1%)  RR 0.91 (95% CI 0.61-1.37)  No p-value Fetal level 66/1147 (5.8%)  RR 1.0 (95% CI 0.72-1.39)  No p-value | Standard care Pregnancy level 45/579 (7.8%)  Fetal level 66/1146 (5.8%) | Not PO  Number of treatments for sepsis | + | ? | + |
| Norman 2021 United Kingdom, Belgium  STOPPIT-2 | Twins | MCDA 20%  DCDA 80%  CL <35 mm | 503  I: 250  C: 253 | Pessary (Arabin) Fetal level 9/500 (1.8%)  No p-value | Standard care Fetal level 4/506 (0.8%) | PO | + | ? | ? |

C; control, CI; confidence interval, CL; cervical length, DA; diamniotic, DCDA; dichorionic diamniotic, I; intervention, MC; monochorionic, MCDA; monochorionic diamniotic, OR; odds ratio, PO; primary outcome, PTB; preterm birth, RR; risk ratio, TVS; transvaginal sonography

* + No or minor problems

? Some problems

- Major problems

| **Author, year Country**  **Trial acronym** | **Singletons/ Twins/ Triplets** | **Risk factor** | **Number of**  **randomized patients**  **n=** | **Results** | | **Comments** | **Directness *** | **Study limitations *** | **Precision *** |
| --- | --- | --- | --- | --- | --- | --- | --- | --- | --- |
|  |  |  |  | **Intervention** | **Control** |  |  |  |  |

| Dugoff 2018 USA PoPPS | Singletons | TVS CL <25 mm | 122  I: 61  C: 61 | Pessary (Biotech cup) 2/60 (3.3%)  RR 0.47 (95% CI 0.09-2.48) p=0.43 | No pessary 4/58 (6.9%) | Not PO  If CL <20 mm progesterone, I: 84% C: 91% | ? | ? | - |
| --- | --- | --- | --- | --- | --- | --- | --- | --- | --- |
| Goya 2012  Spain PECEP | Singletons | TVS CL <25 mm  11% in both groups had previous PTB | 385  I: 192  C: 193 | Pessary (Arabin)  0/190 (0%)  p=0.50 | Standard care 2/190 (1%) | Not PO | ? | ? | + |
| Nicolaides 2016b  9 countries | Singletons | TVS CL <25 mm | 935  I: 466  C: 469 | Pessary (Arabin) 5/450 (1.1%)  OR 5.11 (95% CI 0.59-43.93) p=0.14 | Standard care 1/456 (0.2%) | Not PO  If CL <15 mm progesterone, I: 204 (43.9%)  C: 219 (46.9%) | + | ? | ? |
| Saccone 2017c  Italy | Singletons | TVS CL ≤25 mm Prior cervical surgery I: 7 (4.7%)  C: 5 (3.3%) | 300  I: 150  C: 150 | Pessary (Arabin) 1/150 (0.7%)  RR 0.11 (95% CI 0.01 -0.87) p=0.02 | Standard care 9/150 (6.0%) | Not PO  If CL <20 mm progesterone, I: 133 (88.7%)  C: 125 (83.3%) | ? | ? | ? |
| Berghella 2017a  USA PoPPT | Twins | TVS CL ≤30 mm | 46  I: 23  C: 23 | Pessary (Biotech cup) Fetal level  1/46 (2%) p=1.0 | No pessary Fetal level 0/46 (0%) | Not PO | + | ? | - |
| Goya 2016  Spain  PECEP-Twins | Twins | TVS CL ≤25 mm Previous PTB  I: 16.7%  C: 17.6%  MC twins I: 19.1%  C: 17.6% | 137  I: 68  C: 66 | Pessary (Arabin) Fetal level 0/136 (0%)  No p-value | Standard care Fetal level 0/130 (0%) | Not PO | + | ? | + |

Prevention of preterm birth

STable 4.3.16 Intervention pessary

Outcome variable: Retinopathy of prematurity (ROP)

* + No or minor problems

? Some problems

- Major problems

Prevention of preterm birth

STable 4.3.17 Intervention pessary

Outcome variable: Admittance to neonatal intensive care unit

| **Author, year Country**  **Trial acronym** | **Singletons/ Twins/ Triplets** | **Risk factor** | **Number of**  **randomized patients**  **n=** | **Results** | | **Comments** | **Directness *** | **Study limitations *** | **Precision *** |
| --- | --- | --- | --- | --- | --- | --- | --- | --- | --- |
|  |  |  |  | **Intervention** | **Control** |  |  |  |  |
|  |  |  |  |  |  |  |  |  |  |
| Hui 2013  China | Singletons | TVS CL <25 mm | 108  I: 53  C: 55 | Pessary (Arabin) 21/53 (39.6%)  RR 1.13 (95% CI 0.85-1.50) p=0.54 | Digital examination at entry to simulate pessary insertion  17/55 (32.1%) | Not PO | + | ? | - |
| Karbasian 2016  Iran | Singletons | TVS CL <25 mm | 146  I: 73  C: 73 | Pessary (Arabin)+vaginal progesterone  5/71 (7%) p=0.49 | Vaginal progesterone 3/73 (4.1%) | Not PO | + | ? | - |
| Nicolaides 2016b  9 countries | Singletons | TVS CL <25 mm | 935  I: 466  C: 469 | Pessary (Arabin) 40/450 (8.9%)  OR 1.21 (95% CI 0.75-1.95) p=0.43 | Standard care 34/456 (7.5%) | Not PO  If CL <15 mm progesterone, I: 204 (43.9%) C: 219  (46.9%) | + | ? | ? |
| Pacagnella 2022  Brazil | Singletons 92.4%  Twins 7.6% | All DA CL <30 mm | 936  I: 475  C: 461 | Pessary (Ingamed)+vaginal progesterone  Fetal level 104/506 (20.6%)  RR 1.08 (95% CI 0.82-1.44) | Vaginal progesterone  Fetal level 88/464 (19.0%) | PO | ? | ? | ? |
| Saccone 2017c  Italy | Singletons | TVS CL ≤25 mm Prior cervical surgery I: 7 (4.7%)  C: 5 (3.3%) | 300  I: 150  C: 150 | Pessary (Arabin) 15/150 (10%)  RR 0.54 (95% CI 0.30-0.96) p=0.04 | Standard care 28/150 (18.7%) | Not PO  If CL <20 mm progesterone, I: 133 (88.7%) C: 125  (83.3%) | ? | ? | ? |
| Liem 2013a  The Netherlands | Twins Triplets | Triplets I: 2% (n=9)  C: 2% (n=9)  MC twins I: 22%  C: 24% | 813  I: 403  C: 410 | Pessary (Arabin) Pregnancy level 60/401 (15%)  RR 0.8 (95% CI 0.57-1.13)  No p-value Fetal level 102/811 (13%)  RR 0.83 (95% CI 0.60-1.15)  No p-value | Standard care Pregnancy level 76/407 (19%)  Fetal level 124/823 (15%) | PO | + | ? | ? |
| Norman 2021 United Kingdom, Belgium STOPPIT-2 | Twins | MCDA 20%  DCDA 80%  CL <35 mm | 503  I: 250  C: 253 | Pessary (Arabin) Fetal level 72/500 (14.4%)  OR 1.00 (95% CI 0.54-1.82) p=0.98 | Standard care Fetal level 72/506 (14.2%) | Not PO | + | ? | ? |

C; control, CI; confidence interval, CL; cervical length, DA; diamniotic, DCDA; dichorionic diamniotic, I; intervention, MC; monochorionic, MCDA; monochorionic diamniotic, OR; odds ratio, PO; primary outcome, RR; risk ratio, TVS; transvaginal sonography

Prevention of preterm birth

STable 4.3.18 Intervention pessary

Outcome variable: Long-term child outcome

* + No or minor problems

? Some problems

- Major problems

| **Author, year Country**  **Trial acronym** | **Singletons/ Twins/ Triplets** | **Risk factor** | **Number of**  **randomized patients**  **n=** | **Results** | | **Comments** | **Directness *** | **Study limitations *** | **Precision *** |
| --- | --- | --- | --- | --- | --- | --- | --- | --- | --- |
|  |  |  |  | **Intervention** | **Control** |  |  |  |  |
|  |  |  |  |  |  |  |  |  |  |
| Simons 2019 The Netherlands | Twins Triplets | MC 24.4%  Triplets 2.7% | I: 140  C: 118 | Pessary ASQ, delayed 41/277 (14.8%)  OR 1.54 (95% CI 0.83-2.85)  No p-value  SDQ, abnormal 19/279 (6.8%)  OR 1.37 (95% CI 0.66-2.82)  No p-value  Physical problem 12/277 (4.3%)  OR 1.28 (95% CI 0.57-2.91)  No p-value  Abnormal child outcome 64/281 (22.9%)  OR 1.58 (95% CI 0.94-2.65)  No p-value | No pessary ASQ, delayed 23/229 (10%)  SDQ, abnormal 10/229 (4.4%)  Physical problem 6/229 (2.6%)  Abnormal child outcome 37/233 (15.9%) | 4 year follow-up of the ProTWIN trial. In ProTWIN 813 women participated.  ASQ delayed - based on the mean scores, 1 SD in two or more domains or 2 SD in one domain below Dutch reference group.  SDQ abnormal – scores validated in Dutch population (normal, borderline, abnormal) Physical problem – 3 or more hospital admissions or 3 or more surgeries in the past 4 years.  Abnormal child outcome – at least one of defined above. | + | ? | ? |

C; control, CI; confidence interval, CL; cervical length, I; intervention, MC; monochorionic, OR; odds ratio, PO; primary outcome, PTB; preterm birth, RR; risk ratio, TVS; transvaginal sonography

Cont.

Prevention of preterm birth

STable 4.3.18 cont. Intervention pessary

Outcome variable: Long-term child outcome

* + No or minor problems

? Some problems

- Major problems

| **Author, year Country**  **Trial acronym** | **Singletons/ Twins/ Triplets** | **Risk factor** | **Number of**  **randomized patients**  **n=** | **Results** | | **Comments** | **Directness *** | **Study limitations *** | **Precision *** |
| --- | --- | --- | --- | --- | --- | --- | --- | --- | --- |
|  |  |  |  | **Intervention** | **Control** |  |  |  |  |
|  |  |  |  |  |  |  |  |  |  |
| Van´t Hooft 2018  Netherlands | Twins Triplets | MC 27%  Triplets 1.1% | I: 58  C: 31 | Pessary Bayley-III  (mean score ± SD) Cognitive n=113 101.1 ± 8.3  Mean difference -3.17 (95% CI -  6.1 to -0.2)  Adjusted mean difference -3.06 (95% CI -6.2-0.13)  No p-value  Language n=107 104.9 ± 7.9  Mean difference -0.05 (95% CI -  3.7-3.6)  Adjusted mean difference -0.94 (95% CI -4.7-2.9)  No p-value  Motor n=112 105.8 ± 8.5  Mean difference 0.6 (95% CI -2.9-  4.1)  Adjusted mean difference 0.35 (95% CI -3.4-4.1)  No p-value | No pessary Bayley-III  (mean score ± SD) Cognitive n=59 104.3 ± 7.1  Language n=55 105.0 ± 9.0  Motor n=59 105.2 ± 9.4 | 3 year follow-up of the ProTWIN trial. In ProTWIN 808 women participated.  In adjusted analyses, adjusted for multilevel analysis adjusting for dependence of twins or triplets and potential baseline confounders.  Also adjusted for parental education (high, middle, low), smoking, ethnic origin,  children being eldest in family, use of daycare, bilingual and breastfed >6 months. n/n, number of children in pessary/control group with known Bayley-III (subscale) measurement. | + | ? | ? |

ASQ: Age and Stages Questionnaire, Bayley-III; Bayley Scales of Infant and Toddler Development-third edition, C; control, CI; confidence interval, I; intervention, MC; monochorionic, OR; odds ratio, SD; standard deviation, SDQ: Strength and Difficulties Questionnaire

* + No or minor problems

? Some problems

- Major problems

Prevention of preterm birth

STable 4.3.19 Intervention pessary

Outcome variable: Maternal mortality

| **Author, year Country**  **Trial acronym** | **Singletons/ Twins/ Triplets** | **Risk factor** | **Number of**  **randomized patients**  **n=** | **Results** | | **Comments** | **Directness *** | **Study limitations *** | **Precision *** |
| --- | --- | --- | --- | --- | --- | --- | --- | --- | --- |
|  |  |  |  | **Intervention Pessary** | **Control Standard care** |  |  |  |  |
|  |  |  |  |  |  |  |  |  |  |
| Liem 2013a  The Netherlands ProTWIN | Twins Triplets (2%) | Triplets I: 2% (n=9)  C: 2% (n=9)  MC twins I: 22%  C: 24% | 813  I: 403  C: 410 | Pessary (Arabin) 1/401 (<1%)  No p-value | Standard care 0/407 | Not PO | + | ? | ? |

C; control, I; intervention, MC; monochorionic, PO; primary outcome

* + No or minor problems

? Some problems

- Major problems

Prevention of preterm birth

STable 4.3.20 Intervention pessary

Outcome variable: Hypertensive disorders in pregnancy

| **Author, year Country**  **Trial acronym** | **Singletons/ Twins/ Triplets** | **Risk factor** | **Number of**  **randomized patients**  **n=** | **Results** | | **Comments** | **Directness *** | **Study limitations *** | **Precision *** |
| --- | --- | --- | --- | --- | --- | --- | --- | --- | --- |
|  |  |  |  | **Intervention** | **Control** |  |  |  |  |
|  |  |  |  |  |  |  |  |  |  |
| Liem 2013a  The Netherlands ProTWIN | Twins Triplets | Triplets I: 2% (n=9)  C: 2% (n=9)  MC twins I: 22%  C: 24% | 813  I: 403  C: 410 | Pessary (Arabin) Hypertensive disorder 65/401 (16%)  RR 1.22 (95% CI 0.88-1.72)  No p-value  Eclampsia or HELLP syndrome  8/401 (2%)  RR 1.2 (95% CI 0.41-3.54)  No p-value | Standard care Hypertensive disorder 53/407 (13%)  Eclampsia or HELLP syndrome 7/407 (2%) | Not PO  No definition of hypertension. eclampsia or HELLP | + | ? | ? |

C; control, CI; confidence interval, I; intervention, HELLP; hemolysis, elevated liver enzymes, low platelet counts, MC; monochorionic, PO; primary outcome, RR; risk ratio

* + No or minor problems

? Some problems

- Major problems

Prevention of preterm birth

STable 4.3.21 Intervention pessary

Outcome variable: Chorioamnionitis

| **Author, year Country**  **Trial acronym** | **Singletons/ Twins/ Triplets** | **Risk factor** | **Number of**  **randomized patients**  **n=** | **Results** | | **Comments** | **Directness *** | **Study limitations *** | **Precision *** |
| --- | --- | --- | --- | --- | --- | --- | --- | --- | --- |
|  |  |  |  | **Intervention** | **Control** |  |  |  |  |
|  |  |  |  |  |  |  |  |  |  |
| Dugoff 2018 USA PoPPS | Singletons | TVS CL <25 mm | 122  I: 61  C: 61 | Pessary (Biotech cup) CA  7/60 (11.6%)  RR 1.63 (95% CI 0.5-5.28) p=0.53 | No pessary CA  4/58 (6.9%) | Not PO  If CL <20 mm progesterone, I: 84% C: 91% | ? | ? | - |
| Goya 2012  Spain PECEP | Singletons | TVS CL <25 mm  11% in both groups had previous PTB | 385  I: 192  C: 193 | Pessary (Arabin) CA  5/190 (3%)  OR 0.82 (95% CI 0.2-3.32) p=0.6 | Standard care CA  6/190 (3%) | Not PO | ? | ? | + |
| Karbasian 2016  Iran | Singletons | TVS CL <25 mm | 146  I: 73  C: 73 | Pessary+vaginal progesterone 1/71 (1.4%)  p=0.49 | Vaginal progesterone 0/73 (0%) | Not PO | + | ? | - |
| Saccone 2017c  Italy | Singletons | TVS CL ≤25 mm Prior cervical surgery I: 7 (4.7%)  C: 5 (3.3%) | 300  I: 150  C: 150 | Pessary (Arabin) CA  5/150 (3.3%)  RR 0.71 (95% CI 0.23-2.2)  p= 0.77 | Standard care CA  7/150 (4.7%) | Not PO  If CL <20 mm progesterone, I: 133 (88.7%) C: 125  (83.3%) | ? | ? | ? |
| Berghella 2017a  USA PoPPT | Twins | TVS CL ≤30 mm | 46  I: 23  C: 23 | Pessary (Biotech cup) CA  3/24 (12.5%)  p= 0.23 | No pessary CA  0/22 (0%) | Not PO | + | ? | - |
| Goya 2016  Spain  PECEP-Twins | Twins | TVS CL ≤25 mm Previous PTB  I: 16.7%  C: 17.6%  MC twins I: 19.1%  C: 17.6% | 137  I: 68  C: 66 | Pessary (Arabin) CA  2/68 (3%)  RR 0.97 (95% CI 0.14-6.7)  No p-value | Standard care CA  2/66 (2.9%) | Not PO | + | ? | + |

Cont.

Prevention of preterm birth

STable 4.3.21 cont. Intervention pessary

Outcome variable: Chorioamnionitis

* + No or minor problems

? Some problems

- Major problems

| **Author, year Country**  **Trial acronym** | **Singletons/ Twins/ Triplets** | **Risk factor** | **Number of**  **randomized patients**  **n=** | **Results** | | **Comments** | **Directness *** | **Study limitations *** | **Precision *** |
| --- | --- | --- | --- | --- | --- | --- | --- | --- | --- |
|  |  |  |  | **Intervention** | **Control** |  |  |  |  |
|  |  |  |  |  |  |  |  |  |  |
| Liem 2013a  The Netherlands | Twins Triplets | Triplets I: 2% (n=9)  C: 2% (n=9)  MC twins I: 22%  C: 24% | 813  I: 403  C: 410 | Pessary (Arabin) CA  13/401 (3%)  RR 0.93 (95% CI 0.43-2.01)  No p-value | Standard care CA  14/407 (3%) | Not PO  I: 5 cerclage  C: 0 cerclage | + | ? | ? |

C; control, CA; chorioamnionitis, CI; confidence interval, CL; cervical length, I; intervention, MC; monochorionic, OR; odds ratio, PO; primary outcome, PTB; preterm birth, RR; risk ratio, TVS; transvaginal sonography

* + No or minor problems

? Some problems

- Major problems

Prevention of preterm birth

STable 4.3.22 Intervention pessary

Outcome variable: Genitourinary infections

| **Author, year Country**  **Trial acronym** | **Singletons/ Twins/ Triplets** | **Risk factor** | **Number of**  **randomized patients**  **n=** | **Results** | | **Comments** | **Directness** | **Study limitations** | **Precision *** |
| --- | --- | --- | --- | --- | --- | --- | --- | --- | --- |
|  |  |  |  | **Intervention** | **Control** |  |  |  |  |
|  |  |  |  |  |  |  |  |  |  |
| Dugoff 2018 USA PoPPS | Singletons | TVS CL <25 mm | 122  I: 61  C: 61 | Pessary (Biotech cup) Any genitourinary infection  15/60 (25%)  RR 1.09 (95% CI 0.58-2.06) p=0.83 | No pessary  Any genitourinary infection 14/58 (24.1%) | Not PO  Any genitourinary infection includes urinary tract infection, bacterial vaginosis, chlamydia, gonorrhea, herpes simplex virus and trichomonas.  If CL <20 mm progesterone, I: 84% C: 91% | ? | ? | - |
| Hui 2013  China | Singletons | TVS CL <25 mm | 108  I: 53  C: 55 | Pessary (Arabin)  New vaginal infection 11/53 (20.8%)  RR 1.01 (95% CI 0.83-1.22) p=0.92 | Digital examination at entry to simulate pessary insertion New vaginal infection  11/58 (20%) | Not PO  Candida, bacterial vaginosis, group B streptococcus, Escherichia coli | + | ? | - |
| Pacagnella 2022  Brazil | Singletons 92.4%  Twins 7.6% | All DA CL <30 mm | 936  I: 475  C: 461 | Pessary (Ingamed)+vaginal progesterone  Urinary tract infection 47/475 (9.9%)  p=0.5 | Vaginal progesterone  Urinary tract infection 52/461 (11.3%) | Not PO | ? | ? | ? |
| Berghella 2017a USA PoPPT | Twins | TVS CL ≤30 mm | 46  I: 23  C: 23 | Pessary (Biotech cup)  Urinary tract infection 3/24 (12.5%) | No pessary  Urinary tract infection 1/22 (4.5%) | Not PO | + | ? | - |
| Liem 2013a  The Netherlands | Twins Triplets | Triplets I: 2% (n=9)  C: 2% (n=9)  MC twins I: 22%  C: 24% | 813  I: 403  C: 410 | Pessary (Arabin)  Urinary tract infection 4/401 (1%)  No p-value | Standard care  Urinary tract infection 0/407 (0%) | Not PO  I: 5 cerclage  C: 0 cerclage | + | ? | ? |

C; control, CI; confidence interval, CL; cervical length, DA; diamniotic, I; intervention, MC; monochorionic, PO; primary outcome, RR; risk ratio, TVS; transvaginal sonography

* + No or minor problems

? Some problems

- Major problems

Prevention of preterm birth

STable 4.3.23 Intervention pessary

Outcome variable: Vaginal discharge

| **Author, year Country**  **Trial acronym** | **Singletons/ Twins/ Triplets** | **Risk factor** | **Number of**  **randomized patients**  **n=** | **Results** | | **Comments** | **Directness *** | **Study limitations *** | **Precision *** |
| --- | --- | --- | --- | --- | --- | --- | --- | --- | --- |
|  |  |  |  | **Intervention** | **Control** |  |  |  |  |
|  |  |  |  |  |  |  |  |  |  |
| Dugoff 2018 USA PoPPS | Singletons | TVS CL <25 mm | 122  I: 61  C: 61 | Pessary (Biotech cup) 44/60 (73.3%)  RR 1.48 (95% CI 1.15-1.89) p=0.002 | No pessary 28/58 (48.3%) | Not PO  If CL <20 mm progesterone, I: 84% C: 91% | ? | ? | - |
| Goya 2012  Spain PECEP | Singletons | TVS CL <25 mm  11% in both groups had previous PTB | 385  I: 192  C: 193 | Pessary (Arabin) 190/190 (100%) p=0.002 | Standard care 87/190 (46%) | Not PO | ? | ? | + |
| Saccone 2017c  Italy | Singletons | TVS CL ≤25 mm Prior cervical surgery I: 7 (4.7%)  C: 5 (3.3%) | 300  I: 150  C: 150 | Pessary (Arabin) 130/150 (86.7%)  RR 1.88 (95% CI 1.57-2.27) p<0.001 | Standard care 69/150 (46%) | Not PO  If CL <20 mm progesterone, I: 133 (88.7%) C: 125  (83.3%) | ? | ? | ? |
| Berghella 2017a USA PoPPT | Twins | TVS CL ≤30 mm | 46  I: 23  C: 23 | Pessary (Biotech cup) 19/23 (83%)  RR 1.90 (95% CI 1.15-3.14) p=0.02 | No pessary 10/23 (43%) | Not PO | + | ? | - |
| Goya 2016  Spain  PECEP-Twins | Twins | TVS CL ≤25 mm Previous PTB  I: 16.7%  C: 17.6%  MC twins I: 19.1%  C: 17.6% | 137  I: 68  C: 66 | Pessary (Arabin) 68/68 (100%) p=0.01 | Standard care 35/66 (53%) | Not PO | + | ? | + |

C; control, CI; confidence interval, CL; cervical length, I; intervention, MC; monochorionic, PO; primary outcome, PTB; preterm birth, RR; risk ratio, TVS; transvaginal sonography

* + No or minor problems

? Some problems

- Major problems

Prevention of preterm birth

STable 4.3.24 Intervention pessary

Outcome variable: Maternal morbidity, preterm prelabor rupture of the membranes (PPROM)

| **Author, year Country**  **Trial acronym** | **Singletons/ Twins/ Triplets** | **Risk factor** | **Number of**  **randomized patients**  **n=** | **Results** | | **Comments** | **Directness *** | **Study limitations *** | **Precision *** |
| --- | --- | --- | --- | --- | --- | --- | --- | --- | --- |
|  |  |  |  | **Intervention** | **Control** |  |  |  |  |
|  |  |  |  |  |  |  |  |  |  |
| Dugoff 2018 USA PoPPS | Singletons | TVS CL <25 mm | 122  I: 61  C: 61 | Pessary (Biotech cup) 19/60 (31.7%)  RR 1.20 (95% CI 0.68-2.13) p=0.55 | No pessary 15/58 (25.9%) | Not PO PPROM <37w  If CL <20 mm progesterone, I: 84% C: 91% | ? | ? | - |
| Goya 2012  Spain PECEP | Singletons | TVS CL <25 mm  11% in both groups had previous PTB | 385  I: 192  C: 193 | Pessary (Arabin) 3/190 (2%)  OR 0.16 (95% CI 0.03-0.58) p=0.0013 | Standard care 17/190 (9%) | Not PO PPROM not defined | ? | ? | + |
| Hui 2013  China | Singletons | TVS CL <25 mm | 108  I: 53  C: 55 | Pessary (Arabin) 6/53 (11.3%)  RR 0.96 (95% CI 0.83-1.11)  p= 0.62 | Digital examination at entry to simulate pessary insertion 8/55 (14.5%) | Not PO PPROM not defined | + | ? | - |
| Pacagnella 2022  Brazil | Singletons 92.4%  Twins 7.6% | All DA CL <30 mm | 936  I: 475  C: 461 | Pessary (Ingamed)+vaginal progesterone  26/474 (5.5%)  RR 1.32 (95% CI 0.74-2.36) | Vaginal progesterone  19/458 (4.2%) | Not PO | ? | ? | ? |
| Saccone 2017c  Italy | Singletons | TVS CL ≤25 mm Prior cervical surgery I: 7 (4.7%)  C: 5 (3.3%) | 300  I: 150  C: 150 | Pessary (Arabin)  <34w 2/150 (1.3%)  RR 1.0 (95% CI 0.14-7.01) p>0.99 | Standard care  <34w 2/150 (1.3%) | Not PO PPROM <34w  If CL <20 mm progesterone, I: 133 (88.7%) C: 125  (83.3%) | ? | ? | ? |
| Berghella 2017a USA  PoPPT | Twins | TVS CL ≤30 mm | 46  I: 23  C: 23 | Pessary (Biotech cup) 4/23 (17%)  RR 0.67 (95% CI 0.17-2.05) p=0.72 | No pessary 6/23 (26%) | Not PO  PPROM not defined | + | ? | - |
| Goya 2016  Spain  PECEP-Twins | Twins | TVS CL ≤25 mm Previous PTB  I: 16.7%  C: 17.6%  MC twins I: 19.1%  C: 17.6% | 137  I: 68  C: 66 | Pessary (Arabin) 1/68 (1.5%)  RR 0.16 (95% CI 0.20-1.31)  No p-value | Standard care 6/66 (9.1%) | Not PO PPROM <34w | + | ? | + |

Prevention of preterm birth

STable 4.3.24 cont. Intervention pessary

Outcome variable: Maternal morbidity, preterm prelabor rupture of the membranes (PPROM)

Outcome variable: Maternal morbidity, preterm prelabor rupture of the membranes (PPROM)

* + No or minor problems

? Some problems

- Major problems

| **Author, year Country**  **Trial acronym** | **Singletons/ Twins/ Triplets** | **Risk factor** | **Number of**  **randomized patients**  **n=** | **Results** | | **Comments** | **Directness *** | **Study limitations *** | **Precision *** |
| --- | --- | --- | --- | --- | --- | --- | --- | --- | --- |
|  |  |  |  |  | |  |  |  |  |
|  |  |  |  | **Intervention** | **Control** |  |  |  |  |
|  |  |  |  |  |  |  |  |  |  |
| Liem 2013a  The Netherlands | Twins Triplets | Triplets I: 2% (n=9)  C: 2% (n=9)  MC twins I: 22%  C: 24% | 813  I: 403  C: 410 | Pessary (Arabin) 35/401 (9%)  RR 1.06 (95% CI 0.68-1.66)  No p-value | Standard care 34/407 (8%) | Not PO PPROM not defined | + | ? | ? |
| Norman  2021  United Kingdom,  Belgium STOPPIT-2 | Twins | MCDA 20%  DCDA 80%  CL <35 mm | 503  I: 250  C: 253 | Pessary (Arabin) 12/250 (4.8%)  OR 1.95 (95% CI 0.52-7.34) p=0.20 | Standard care 4/253 (1.6%) | Not PO | + | ? | ? |

C; control, CI; confidence interval, CL; cervical length, DA; diamniotic, DCDA; dichorionic diamniotic, I; intervention, MC; monochorionic, MCDA; monochorionic diamniotic, OR; odds ratio, PO; primary outcome, PPROM; preterm prelabor rupture of membranes, PTB; preterm birth, RR; risk ratio, TVS; transvaginal sonography
