# Supplementary material for: Progesterone, cerclage, pessary, or acetylsalicylic acid for prevention of preterm birth in singleton and multifetal pregnancies – A systematic review and meta-analyses
Source: Front Med (Lausanne). 2023 Feb 28;10:1111315. doi: 10.3389/fmed.2023.1111315 (PMC10015499; doi:10.3389/fmed.2023.1111315)
Supplement: Supplementary file 1 [file Data_Sheet_1.zip › Data Sheet 1_corrected/Appendix 4.4 Outcome tables_ASA.docx]

**Progesterone, cerclage, pessary, or acetylsalicylic acid for prevention of preterm birth in**

**singleton and multifetal pregnancies**

**Appendix 4.4 Outcome tables ASA in singleton pregnancies**

**Preterm birth**

STable 4.4.1.a Preterm birth <37 weeks

STable 4.4.1.b Spontaneous preterm birth <37 weeks

STable 4.4.2.a Any preterm birth <34 weeks

STable 4.4.2.b Spontaneous preterm birth <34 weeks

STable 4.4.3.a Any preterm birth <28 weeks

STable 4.4.3.b Spontaneous preterm birth <28 weeks

**Gestational age**

STable 4.4.4 Gestational age

**Perinatal mortality and neonatal morbidity**

STable 4.4.5 Perinatal mortality

STable 4.4.6 Composite adverse neonatal outcome

STable 4.4.7 Bronchopulmonary dysplasia

STable 4.4.8 Intraventricular hemorrhage

STable 4.4.9 Necrotizing enterocolitis

STable 4.4.10 Neonatal sepsis

STable 4.4.11 Retinopathy of prematurity

STable 4.4.12 Admission to neonatal intensive care unit and median days spent at NICU

**Maternal mortality and morbidity**

STable 4.4.13 Maternal mortality

STable 4.4.14 Maternal morbidity, hypertensive disorders in pregnancy

STable 4.4.15 Maternal morbidity, gestational diabetes

STable 4.4.16 Maternal morbidity, infections

STable 4.4.17 Maternal morbidity, bleeding

STable 4.4.18 Maternal morbidity, preterm prelabor rupture of the membranes

| **Author, year Country**  **Trial acronym** | **Singletons/ Twins/ Triplets** | **Risk factor** | **Number of**  **randomized patients**  **n=** | **Results** | | **Comments** | **Directness *** | **Study limitations *** | **Precision *** |
| --- | --- | --- | --- | --- | --- | --- | --- | --- | --- |
|  |  |  |  | **Intervention** | **Control** |  |  |  |  |

| Landman 2022  The Netherlands (34 centers) | Singletons | History of sPTB | I: 194  C: 193 | 80 mg oral ASA/d 41/194 (21.1%)  RR 0.83  (95% CI 0.58-1.20) p=0.323  Compliance >80%  24/125 (19.2%)  RR 0.77  (95% CI 0.48-1.25) p=0.291 | Placebo 49/193 (25.4%)  Compliance >80%  30/121 (24.8%) | PO  Compliance in the whole randomized cohort 63.3%. | + | + | - |
| --- | --- | --- | --- | --- | --- | --- | --- | --- | --- |

C; control, CI; confidence interval, I; intervention, PO; primary outcome, RR; risk ratio, sPTB; spontaneous preterm birth

| **Author, year Country**  **Trial acronym** | **Singletons/ Twins/ Triplets** | **Risk factor** | **Number of**  **randomized patients**  **n=** | **Results** | | **Comments** | **Directness *** | **Study limitations *** | **Precision *** |
| --- | --- | --- | --- | --- | --- | --- | --- | --- | --- |
|  |  |  |  | **Intervention** | **Control** |  |  |  |  |

| Landman 2022  The Netherlands (34 centers) | Singletons | History of sPTB | I: 194  C: 193 | 80 mg oral ASA/d 39/194 (20.1%)  RR 0.84  (95% CI 0.58-1.23) p=0.376 | Placebo 46/193 (23.8%) | Not PO | + | + | - |
| --- | --- | --- | --- | --- | --- | --- | --- | --- | --- |

C; control, CI; confidence interval, I; intervention, PO; primary outcome, RR; risk ratio, sPTB; spontaneous preterm birth

| **Author, year Country**  **Trial acronym** | **Singletons/ Twins/ Triplets** | **Risk factor** | **Number of**  **randomized patients**  **n=** | **Results** | | **Comments** | **Directness *** | **Study limitations *** | **Precision *** |
| --- | --- | --- | --- | --- | --- | --- | --- | --- | --- |
|  |  |  |  | **Intervention** | **Control** |  |  |  |  |

| Landman 2022  The Netherlands (34 centers) | Singletons | History of sPTB | I: 194  C: 193 | 80 mg oral ASA/d 18/194 (9.3%)  RR 1.05  (95% CI 0.56-1.98) p=0.872 | Placebo 17/193 (8.8%) | Not PO | + | + | - |
| --- | --- | --- | --- | --- | --- | --- | --- | --- | --- |

C; control, CI; confidence interval, I; intervention, PO; primary outcome, RR; risk ratio, sPTB; spontaneous preterm birth

| **Author, year Country**  **Trial acronym** | **Singletons/ Twins/ Triplets** | **Risk factor** | **Number of**  **randomized patients**  **n=** | **Results** | | **Comments** | **Directness *** | **Study limitations *** | **Precision *** |
| --- | --- | --- | --- | --- | --- | --- | --- | --- | --- |
|  |  |  |  | **Intervention** | **Control** |  |  |  |  |

| Landman 2022  The Netherlands (34 centers) | Singletons | History of sPTB | I: 194  C: 193 | 80 mg oral ASA/d 18/194 (9.3%)  RR 1.12  (95% CI 0.59-2.13) p=0.732 | Placebo 16/193 (8.3%) | Not PO | + | + | - |
| --- | --- | --- | --- | --- | --- | --- | --- | --- | --- |

C; control, CI; confidence interval, I; intervention, PO; primary outcome, RR; risk ratio, sPTB; spontaneous preterm birth

| **Author, year Country**  **Trial acronym** | **Singletons/ Twins/ Triplets** | **Risk factor** | **Number of**  **randomized patients**  **n=** | **Results** | | **Comments** | **Directness *** | **Study limitations *** | **Precision *** |
| --- | --- | --- | --- | --- | --- | --- | --- | --- | --- |
|  |  |  |  | **Intervention** | **Control** |  |  |  |  |

| Landman 2022  The Netherlands (34 centers) | Singletons | History of sPTB | I: 194  C: 193 | 80 mg oral ASA/d 7/194 (3.6%)  RR 1.39  (95% CI 0.45-4.31) p=0.566 | Placebo 5/193 (2.6%) | Not PO | + | + | - |
| --- | --- | --- | --- | --- | --- | --- | --- | --- | --- |

C; control, I; intervention, CI; confidence interval, PO; primary outcome, RR; risk ratio, sPTB; spontaneous preterm birth

| **Author, year Country**  **Trial acronym** | **Singletons/ Twins/ Triplets** | **Risk factor** | **Number of**  **randomized patients**  **n=** | **Results** | | **Comments** | **Directness *** | **Study limitations *** | **Precision *** |
| --- | --- | --- | --- | --- | --- | --- | --- | --- | --- |
|  |  |  |  | **Intervention** | **Control** |  |  |  |  |

| Landman 2022  The Netherlands (34 centers) | Singletons | History of sPTB | I: 194  C: 193 | 80 mg oral ASA/d 7/194 (3.6%)  RR 1.39  (95% CI 0.45-4.31) p=0.566 | Placebo 5/193 (2.6%) | Not PO | + | + | - |
| --- | --- | --- | --- | --- | --- | --- | --- | --- | --- |

C; control, I; intervention, CI; confidence interval, PO; primary outcome, RR; risk ratio, sPTB; spontaneous preterm birth

| **Author, year Country**  **Trial acronym** | **Singletons/ Twins/ Triplets** | **Risk factor** | **Number of**  **randomized patients**  **n=** | **Results** | | **Comments** | **Directness *** | **Study limitations *** | **Precision *** |
| --- | --- | --- | --- | --- | --- | --- | --- | --- | --- |
|  |  |  |  | **Intervention** | **Control** |  |  |  |  |

| Landman 2022  The Netherlands (34 centers) | Singletons | History of sPTB | I: 194  C: 193 | 80 mg oral ASA/d Median (Q1; Q2): 38+1 w  (37+1; 39+1)  (95% CI 37+6 to 38+3) p=0.964 | Placebo Median (Q1; Q2): 38+1  (36+6; 39+2)  (95% CI 37+6 to 38+4) | Not PO | + | + | ? |
| --- | --- | --- | --- | --- | --- | --- | --- | --- | --- |

C; control, CI; confidence interval, IQR; interquartile range, I; intervention, PO; primary outcome, sPTB; spontaneous preterm birth, w; weeks

| **Author, year Country**  **Trial acronym** | **Singletons/ Twins/ Triplets** | **Risk factor** | **Number of**  **randomized patients**  **n=** | **Results** | | **Comments** | **Directness *** | **Study limitations *** | **Precision *** |
| --- | --- | --- | --- | --- | --- | --- | --- | --- | --- |
|  |  |  |  | **Intervention** | **Control** |  |  |  |  |

| Landman 2022  The Netherlands (34 centers) | Singletons | History of sPTB | I: 194  C: 193 | 80 mg oral ASA/d Fetal death 4/194 (2.1%)  RR 1.99  (95% CI 0.37-10.74) p=0.685  Neonatal death 2/194 (1.0%)  No statistics  All mortality 6/194 (3.1%)  RR 2.99  (95% CI 0.61-14.60) P=0.284 | Placebo  Fetal death 2/193 (1.0%)  Neonatal death 0/193 (0%)  All mortality  2/193 (1.0%) | Not PO.  Fetal death is defined as death during  pregnancy (≥16 weeks) or during labor.  Neonatal death is defined as death occurring in the period after birth until discharge.  All mortality is defined as death of a fetus or neonate at any time between a gestational age (≥16 weeks) and discharge. | + | + | - |
| --- | --- | --- | --- | --- | --- | --- | --- | --- | --- |

C; control, CI; confidence interval, I; intervention, PO; primary outcome, RR; risk ratio, sPTB; spontaneous preterm birth

| **Author, year Country**  **Trial acronym** | **Singletons/ Twins/ Triplets** | **Risk factor** | **Number of**  **randomized patients**  **n=** | **Results** | | **Comments** | **Directness *** | **Study limitations *** | **Precision *** |
| --- | --- | --- | --- | --- | --- | --- | --- | --- | --- |
|  |  |  |  | **Intervention** | **Control** |  |  |  |  |

| Landman 2022  The Netherlands (34 centers) | Singletons | History of sPTB | I: 194  C: 193 | 80 mg oral ASA/d 9/194 (4.6%)  RR 1.79  (95% CI 0.61-5.25) p=0.288 | Placebo 5/193 (2.6%) | Not PO  Composite adverse neonatal outcome includes all perinatal deaths recorded during the study period which includes two deaths in gestational week 16 and two deaths in midtrimester 18+6 and 21+4 and extreme premature births 24+2 and 25+2. In the intervention group and one death in midtrimester, 22+5, in the placebo group.  Other adverse outcomes included were BPD, IVH, NEC, ROP, sepsis | + | + | - |
| --- | --- | --- | --- | --- | --- | --- | --- | --- | --- |

BPD; bronchopulmonary dysplasia, C; control, CI; confidence interval, I; intervention, IVH; intraventricular hemorrhage, NEC; necrotizing enterocolitis, PO; primary outcome, ROP; retinopathy of prematurity, RR; risk ratio, sPTB; spontaneous preterm birth

| **Author, year Country**  **Trial acronym** | **Singletons/ Twins/ Triplets** | **Risk factor** | **Number of**  **randomized patients**  **n=** | **Results** | | **Comments** | **Directness *** | **Study limitations *** | **Precision *** |
| --- | --- | --- | --- | --- | --- | --- | --- | --- | --- |
|  |  |  |  | **Intervention** | **Control** |  |  |  |  |

| Landman 2022  The Netherlands (34 centers) | Singletons | History of sPTB | I: 194  C: 193 | 80 mg oral ASA/d 1/194 (0.5%)  RR 0.33  (95% CI 0.04-3.16) p=0.372 | Placebo 3/193 (1.6%) | Not PO | + | + | - |
| --- | --- | --- | --- | --- | --- | --- | --- | --- | --- |

C; control, CI; confidence interval, I; intervention, PO; primary outcome, RR; risk ratio, sPTB; spontaneous preterm birth

| **Author, year Country**  **Trial acronym** | **Singletons/ Twins/ Triplets** | **Risk factor** | **Number of**  **randomized patients**  **n=** | **Results** | | **Comments** | **Directness *** | **Study limitations *** | **Precision *** |
| --- | --- | --- | --- | --- | --- | --- | --- | --- | --- |
|  |  |  |  | **Intervention** | **Control** |  |  |  |  |

| Landman 2022  The Netherlands (34 centers) | Singletons | History of sPTB | I: 194  C: 193 | 80 mg oral ASA/d 1/194 (0.5%)  No statistics | Placebo 0/193 (0%) | Not PO | + | + | - |
| --- | --- | --- | --- | --- | --- | --- | --- | --- | --- |

C; control, CI; confidence interval, I; intervention, PO; primary outcome, RR; risk ratio, sPTB; spontaneous preterm birth

| **Author, year Country**  **Trial acronym** | **Singletons/ Twins/ Triplets** | **Risk factor** | **Number of**  **randomized patients**  **n=** | **Results** | | **Comments** | **Directness *** | **Study limitations *** | **Precision *** |
| --- | --- | --- | --- | --- | --- | --- | --- | --- | --- |
|  |  |  |  | **Intervention** | **Control** |  |  |  |  |

| Landman 2022  The Netherlands (34 centers) | Singletons | History of sPTB | I: 194  C: 193 | 80 mg oral ASA/d 1/194 (0.5%)  No statistics | Placebo 0/193 (0%) | Not PO | + | + | ? |
| --- | --- | --- | --- | --- | --- | --- | --- | --- | --- |

C; control, CI; confidence interval, I; intervention, PO; primary outcome, RR; risk ratio, sPTB; spontaneous preterm birth

| **Author, year Country**  **Trial acronym** | **Singletons/ Twins/ Triplets** | **Risk factor** | **Number of**  **randomized patients**  **n=** | **Results** | | **Comments** | **Directness *** | **Study limitations *** | **Precision *** |
| --- | --- | --- | --- | --- | --- | --- | --- | --- | --- |
|  |  |  |  | **Intervention** | **Control** |  |  |  |  |

| Landman 2022  The Netherlands (34 centers) | Singletons | History of sPTB | I: 194  C: 193 | 80 mg oral ASA/d 4/194 (2.1%)  RR 1.99  (95% CI 0.37-10.74) p=0.424 | Placebo 2/193 (1.0%) | Not PO  Culture proven sepsis. Table S3 in the article states one more case of sepsis in the placebo group, no reason for exclusion is given. | + | + | - |
| --- | --- | --- | --- | --- | --- | --- | --- | --- | --- |

C; control, CI; confidence interval, I; intervention, PO; primary outcome, RR; risk ratio, sPTB; spontaneous preterm birth

| **Author, year Country**  **Trial acronym** | **Singletons/ Twins/ Triplets** | **Risk factor** | **Number of**  **randomized patients**  **n=** | **Results** | | **Comments** | **Directness *** | **Study limitations *** | **Precision *** |
| --- | --- | --- | --- | --- | --- | --- | --- | --- | --- |
|  |  |  |  | **Intervention** | **Control** |  |  |  |  |

| Landman 2022  The Netherlands (34 centers) | Singletons | History of sPTB | I: 194  C: 193 | 80 mg oral ASA/d 1/194 (0.5%)  RR 0.50  (95% CI 0.05-5.44) p=0.623 | Placebo 2/193 (1.0%) | Not PO | + | + | - |
| --- | --- | --- | --- | --- | --- | --- | --- | --- | --- |

C; control, CI; confidence interval, I; intervention, PO; primary outcome, RR; risk ratio, sPTB; spontaneous preterm birth

| **Author, year Country**  **Trial acronym** | **Singletons/ Twins/ Triplets** | **Risk factor** | **Number of**  **randomized patients**  **n=** | **Results** | | **Comments** | **Directness *** | **Study limitations *** | **Precision *** |
| --- | --- | --- | --- | --- | --- | --- | --- | --- | --- |
|  |  |  |  | **Intervention** | **Control** |  |  |  |  |

| Landman 2022  The Netherlands (34 centers) | Singletons | History of sPTB | I: 194  C: 193 | 80 mg oral ASA/d 13/194 (6.7%)  RR 1.18  (95% CI 0.54-2.56) p=0.683  Median (IQR)days 12 (4-46)  (95% CI 2-54) n=13  Median diff. 5 days p=0.560 | Placebo 11/193 (5.7%)  Median days spent (IQR) 7 (2-58)  (95% CI 2-82) n=11 | Not PO  Days spent at NICU calculated of those neonates with an admission until 3 months corrected age. | + | + | - |
| --- | --- | --- | --- | --- | --- | --- | --- | --- | --- |

C; control, CI; confidence interval, I; intervention, IQR; interquartile range, NICU; neonatal intensive care unit, PO; primary outcome, RR; risk ratio, sPTB; spontaneous preterm birth

| **Author, year Country**  **Trial acronym** | **Singletons/ Twins/ Triplets** | **Risk factor** | **Number of**  **randomized patients**  **n=** | **Results** | | **Comments** | **Directness *** | **Study limitations *** | **Precision *** |
| --- | --- | --- | --- | --- | --- | --- | --- | --- | --- |
|  |  |  |  | **Intervention** | **Control** |  |  |  |  |

| Landman 2022  The Netherlands (34 centers) | Singletons | History of sPTB | I: 194  C: 193 | 80 mg oral ASA/d 0/194 (0%)  No statistics | Placebo 0/193 (0%) | Not PO | + | + | - |
| --- | --- | --- | --- | --- | --- | --- | --- | --- | --- |

C; control, CI; confidence interval, I; intervention, PO; primary outcome, RR; risk ratio, sPTB; spontaneous preterm birth

| **Author, year Country**  **Trial acronym** | **Singletons/ Twins/ Triplets** | **Risk factor** | **Number of**  **randomized patients**  **n=** | **Results** | | **Comments** | **Directness *** | **Study limitations *** | **Precision *** |
| --- | --- | --- | --- | --- | --- | --- | --- | --- | --- |
|  |  |  |  | **Intervention** | **Control** |  |  |  |  |

| Landman 2022  The Netherlands (34 centers) | Singletons | History of sPTB | I: 194  C: 193 | 80 mg oral ASA/d GH 4/194 (2.1%)  RR 0.80  (95% CI 0.22-2.92) p=0.751  PE/HELLP 2/194 (1.0%)  RR 1.00  (95% CI 0.14-6.99) p=1.00  Eclampsia 0/194 (0%) No statistics | Placebo  GH 5/193 (2.6%)  PE/HELLP 2/193 (1.0%)  Eclampsia 0/193 (0%) | Not PO | + | + | ? |
| --- | --- | --- | --- | --- | --- | --- | --- | --- | --- |

C; control, CI; confidence interval, GH; gestational hypertension, HELLP; hemolysis, elevated liver enzymes, low platelets, I; intervention, PE; preeclampsia, PO; primary outcome, RR; risk ratio, sPTB; spontaneous preterm birth

| **Author, year Country**  **Trial acronym** | **Singletons/ Twins/ Triplets** | **Risk factor** | **Number of**  **randomized patients**  **n=** | **Results** | | **Comments** | **Directness *** | **Study limitations *** | **Precision *** |
| --- | --- | --- | --- | --- | --- | --- | --- | --- | --- |
|  |  |  |  | **Intervention** | **Control** |  |  |  |  |

| Landman 2022  The Netherlands (34 centers) | Singletons | History of sPTB | I: 194  C: 193 | 80 mg oral ASA/d 15/194 (7.7%)  RR 1.00  (95% CI 0.50-1.98) p=0.988 | Placebo 15/193 (7.8%) | Not PO | + | + | - |
| --- | --- | --- | --- | --- | --- | --- | --- | --- | --- |

C; control, CI; confidence interval, I; intervention, PO; primary outcome, RR; risk ratio, sPTB; spontaneous preterm birth

| **Author, year Country**  **Trial acronym** | **Singletons/ Twins/ Triplets** | **Risk factor** | **Number of**  **randomized patients**  **n=** | **Results** | | **Comments** | **Directness *** | **Study limitations *** | **Precision *** |
| --- | --- | --- | --- | --- | --- | --- | --- | --- | --- |
|  |  |  |  | **Intervention** | **Control** |  |  |  |  |

| Landman 2022  The Netherlands (34 centers) | Singletons | History of sPTB | I: 194  C: 193 | 80 mg oral ASA/d UTI/GI 6/194 (3.1%)  RR 0.40  (95% CI 0.16-1.00) p=0.051  BV 12/194 (6.2%)  RR 0.57  (95% CI 0.29-1.12) p=0.104 | Placebo UTI/GI 15/193 (7.8%)  BV 21/193 (10.9%) | Not PO | + | + | - |
| --- | --- | --- | --- | --- | --- | --- | --- | --- | --- |

BV; bacterial vaginosis, C; control, CI; confidence interval, GI; genital infection, I; intervention, PO; primary outcome, RR; risk ratio, sPTB; spontaneous preterm birth, UTI; urinary tract infection

admission to hospital)

| **Author, year Country**  **Trial acronym** | **Singletons/ Twins/ Triplets** | **Risk factor** | **Number of**  **randomized patients**  **n=** | **Results** | | **Comments** | **Directness *** | **Study limitations *** | **Precision *** |
| --- | --- | --- | --- | --- | --- | --- | --- | --- | --- |
|  |  |  |  | **Intervention** | **Control** |  |  |  |  |

| Landman 2022  The Netherlands (34 centers) | Singletons | History of sPTB | I: 194  C: 193  I: 106 C:116  I: 106 C:115  I: 194  C: 193 | 80 mg oral ASA/d Placental abruption 0/194 (0%)  No statistics  Vaginal bleeding 5/106 (4.7%)  RR 0.78  (95% CI 0.26-2.39) p=0.666  Other bleedings 17/106 (16.0%)  RR 1.54  (95% CI 0.77-3.01) p=0.222  Hospital admission 10/194 (5.2%)  RR 1.11  (95% CI 0.46-2.66) p=0.823 | Placebo  Placental abruption 2/193 (1.0%)  Vaginal bleeding 7/116 (6.0%)  Other bleedings 12/115 (10.4%)  Hospital admission 9/193 (4.7%) | Not PO  Vaginal bleeding and other bleeding are self-reported, thus altering the denominator due to response rates.  Types of other bleeding: anal bleeding, epitaxis, prolonged wound bleeding, gingival bleeding.  Admission to hospital due to vaginal bleeding during pregnancy.  Postpartum hemorrhage not reported | + | + | - |
| --- | --- | --- | --- | --- | --- | --- | --- | --- | --- |

C; control, CI; confidence interval, I; intervention, PO; primary outcome, RR; risk ratio, sPTB; spontaneous preterm birth

| **Author, year Country**  **Trial acronym** | **Singletons/ Twins/ Triplets** | **Risk factor** | **Number of**  **randomized patients**  **n=** | **Results** | | **Comments** | **Directness *** | **Study limitations *** | **Precision *** |
| --- | --- | --- | --- | --- | --- | --- | --- | --- | --- |
|  |  |  |  | **Intervention** | **Control** |  |  |  |  |

| Landman 2022  The Netherlands (34 centers) | Singletons | History of sPTB | I: 194  C: 193 | 80 mg oral ASA/d 9/194 (4.6%)  RR 0.50  (95% CI 0.23-1.08) p=0.077 | Placebo 18/193 (9.3%) | Not PO  Defined as PPROM <37 weeks of gestation. | + | + | - |
| --- | --- | --- | --- | --- | --- | --- | --- | --- | --- |

C; control, CI; confidence interval, I; intervention, PO; primary outcome, PPROM; preterm prelabor rupture of membranes, RR; risk ratio, sPTB; spontaneous preterm birth
