# Supplementary material for: Progesterone, cerclage, pessary, or acetylsalicylic acid for prevention of preterm birth in singleton and multifetal pregnancies – A systematic review and meta-analyses
Source: Front Med (Lausanne). 2023 Feb 28;10:1111315. doi: 10.3389/fmed.2023.1111315 (PMC10015499; doi:10.3389/fmed.2023.1111315)
Supplement: Supplementary file 1 [file Data_Sheet_1.zip › Data Sheet 1_corrected/Appendix 5.1 Results progesterone vs placebo in singleton pregnancies.docx]

**HTA-centrum**

**Progesterone, cerclage, pessary, or acetylsalicylic acid for prevention of preterm birth in singleton and multifetal pregnancies**

**Appendix 5.1 Results progesterone vs placebo in singleton pregnancies**

**Table of contents**

[Abbreviations1](#_Abbreviations)

# [STable 1 Risk of bias legend2](#_STable_1._Risk)

[Results per outcome progesterone vc placebo in singleton pregnancies2](#_Results_per_outcome_1)

[Preterm birth SFigures 1-10 (SFigures 1, 5, 7 corrected)2-11](#_Preterm_birth_in)

## [Gestational age, low birth weight, very low birth weight SFigures 11-1311-](#_SFigure_11._Outcome:)13

[Neonatal mortality and morbidity SFigures 14-2214-20](#_SFigure_14._Outcome:)

[Maternal morbidity SFigures 23-2720-](#_SFigure_23._Outcome:)23

[Subgroup analyses progesterone vs placebo in singleton pregnancies2](#_STable_2_shows)4

[**STable 2** Summary estimates from subgroup meta-analyses2](#_STable_2_shows)4

[Preterm birth SFigures 28-35 (SFigures 28-33 corrected)26-38](#_SFigure_28._Outcome:)

# Abbreviations

17-OHPC 17-alpha-hydroxyprogesterone caproate

BPD bronchopulmonary dysplasia

CI confidence interval

GDM gestational diabetes mellitus

HDP hypertensive disorders in pregnancy

HTA health technology assessment

ICP intrahepatic cholestasis in pregnancy

im intramuscular injection

IVH intraventricular hemorrhage

mm millimetre

NEC necrotizing enterocolitis

NICU neonatal intensive care unit

PPROM preterm prelabor rupture of membranes

PTB preterm birth

RCT randomized controlled trial

RD risk difference

RDS respiratory distress syndrome

ROP retinopathy of prematurity

RR relative risk/risk ratio

sPTB spontaneous preterm birth

# **STable 1**. **Risk of bias legend** to the colour plot within the following forests plots

1. Random sequence generation (selection bias)
2. Allocation concealment (selection bias)
3. Blinding of participants and personnel (performance bias)
4. Blinding of outcome assessment (detection bias)
5. Incomplete outcome data (attrition bias)
6. Selective reporting (reporting bias)
7. Conflict of interest bias

# Results per outcome

### **Preterm birth in singletons across gestational weeks**

**Any preterm birth <37 weeks** (Appendix 4.1, STable 4.1.1.a and SFigure 1)

A meta-analysis of 14 trials with low risk of bias, including 6303 women, showed a reduction in the rate of any preterm birth, RR 0.82 (95% CI 0.71 to 0.95). The crude event rate across trials was 30.2% without progesterone. The pooled weighted RD was -6.6 percentage points (95% CI -10.8 to -2.3).

SFigure 1. Outcome: Any preterm birth <37 weeks.


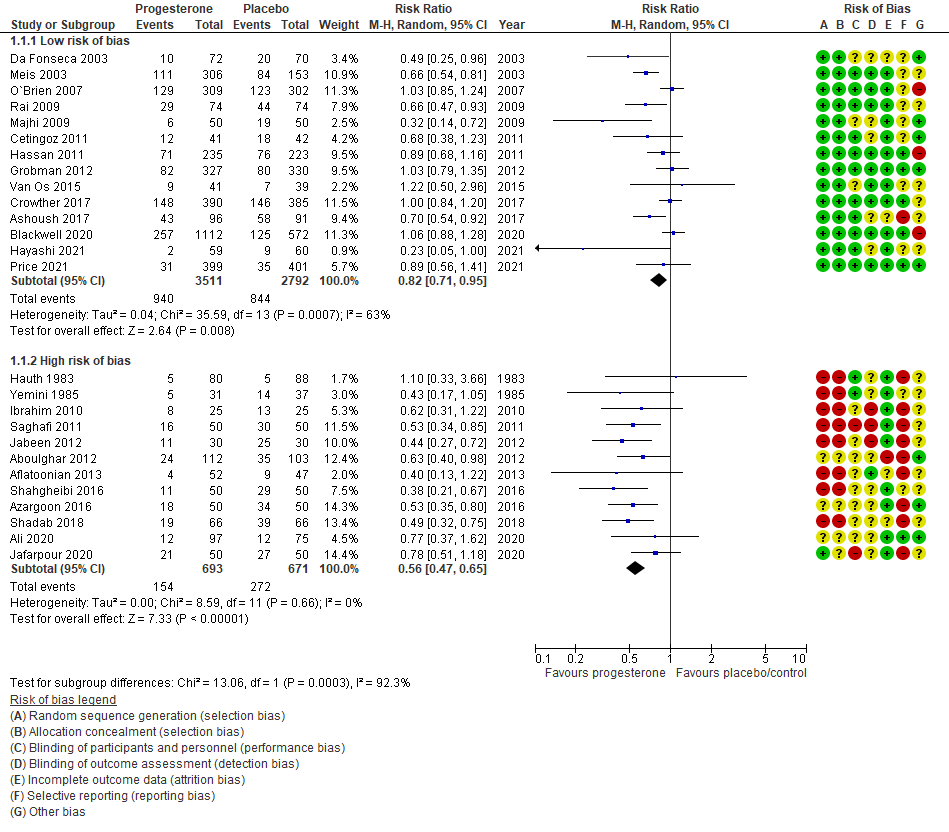


Conclusion: Progesterone compared with placebo reduces the risk of any preterm birth before 37 gestational weeks in women with a singleton pregnancy, neither considering administration route and dosage, nor type of risk factor for preterm birth (GRADE ⊕⊕⊕ ⊕).

**SFigure 1. Outcome: Any preterm birth <37 weeks**. Cetingoz et al. 2011 excluded from analysis (article retracted 2024).


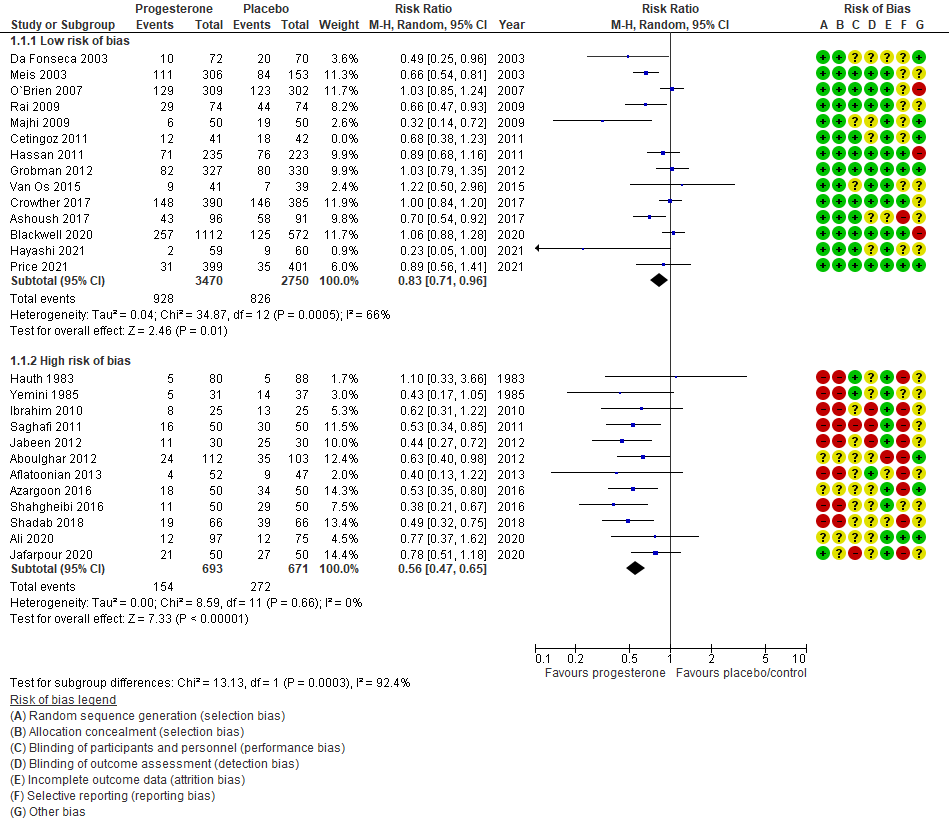


**Spontaneous preterm birth <37 weeks** (Appendix 4.1, STable 4.1.1.b and SFigure 2)

A meta-analysis of six trials with low risk of bias, including 3698 women, showed no difference in the rate of spontaneous preterm birth, RR 0.87 (95% CI 0.67 to 1.13). The crude event rate across trials was 17.4% without progesterone. The pooled weighted RD was -2.4 percentage points (95% CI -7.3 to 2.4).

**SFigure 2.** Outcome: Spontaneous preterm birth <37 weeks.


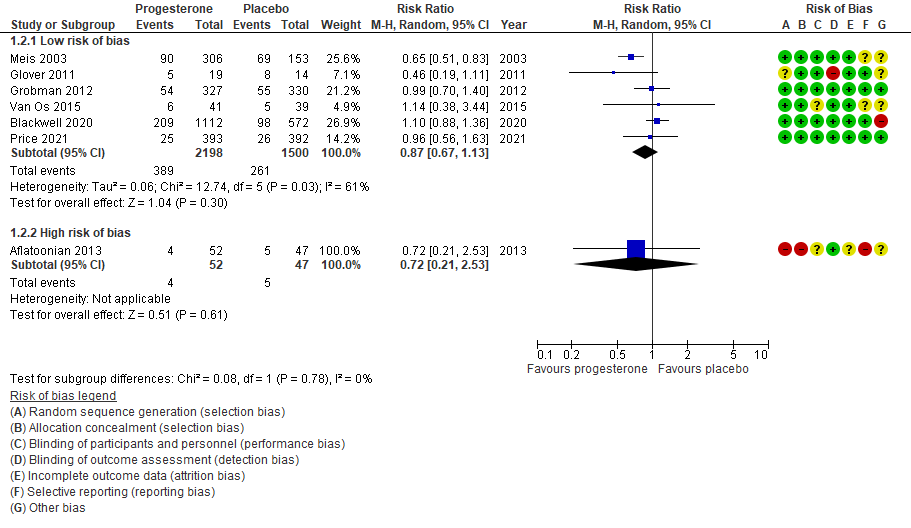


Conclusion: Progesterone compared with placebo probably result in no difference in the risk of spontaneous preterm birth before 37 gestational weeks in women with a singleton pregnancy, neither considering administration route and dosage, nor type of risk factor for preterm birth (GRADE ⊕⊕ ⊕🌕).

**Any preterm birth <35 weeks** (Appendix 4.1, STable 4.1.2.a and SFigure 3**)**

A meta-analysis of five trials with low risk of bias, including 3872 women, showed a significant reduction in the rate of any preterm birth, RR 0.80 (95% CI 0.68 to 0.93). The crude event rate across trials was 18.8% without progesterone. The pooled weighted RD was -4.1 percentage points (95% CI -7.8 to -0.5).

**SFigure 3.** Outcome: Any preterm birth <35 weeks.


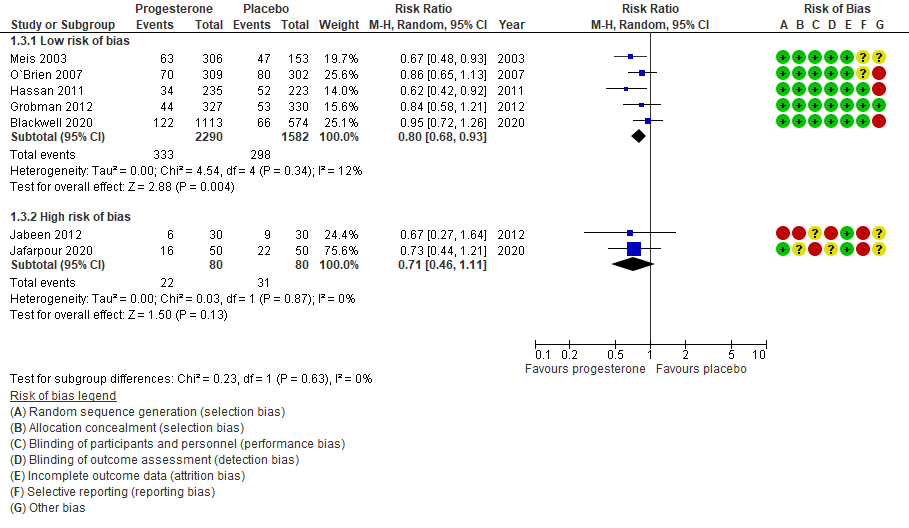


Conclusion: Progesterone compared with placebo probably reduces the risk of any preterm birth before 35 gestational weeks in women with a singleton pregnancy, neither considering administration route and dosage, nor type of risk factor for preterm birth (GRADE ⊕⊕⊕ 🌕).

**Spontaneous preterm birth <35 weeks** (Appendix 4.1, STable 4.1.2.b and SFigure 4**)**

One trial with low risk of bias, including 1687 women with a previous spontaneous preterm birth, compared 17-OHPC with placebo, showed no difference in the rate of spontaneous preterm birth, RR 0.94 (95% CI 0.68 to 1.30). The crude event rate across trials was 8.9% without progesterone. The RD was -0.5 percentage points (95% CI -3.4 to 2.3).

**SFigure 4.** Outcome: Spontaneous preterm birth <35 weeks.


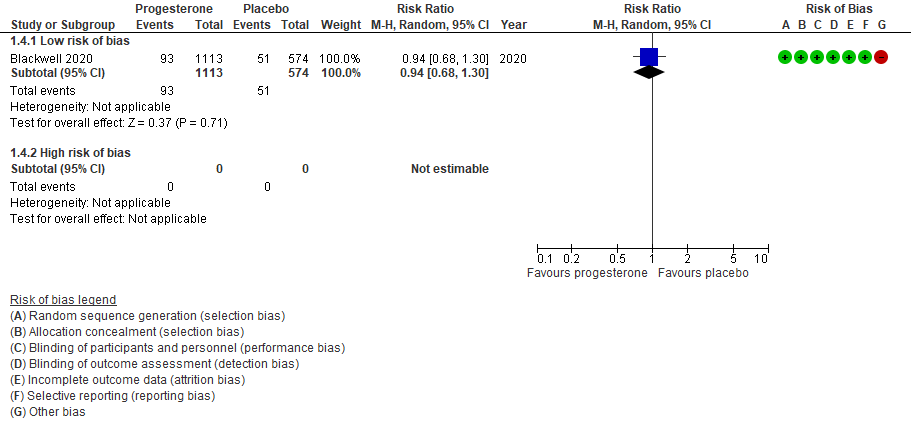


Conclusion: 17-OHPC compared with placebo probably results in no difference in the risk of spontaneous preterm birth before 35 gestational weeks in women with a singleton pregnancy and previous spontaneous preterm birth (GRADE ⊕⊕⊕🌕).

**Any preterm birth <34 weeks** (Appendix 4.1, STable 4.1.3.a and SFigure 5)

A meta-analysis of 16 trials with low risk of bias, including 7581 women, showed a reduction in the rate of any preterm birth, RR 0.78 (95% CI 0.68 to 0.89). The crude event rate across trials was 15.2% without progesterone. The pooled weighted RD was -3.5 percentage points (95% CI -5.8 to -1.2).

**SFigure 5.** Outcome: Any preterm birth <34 weeks.
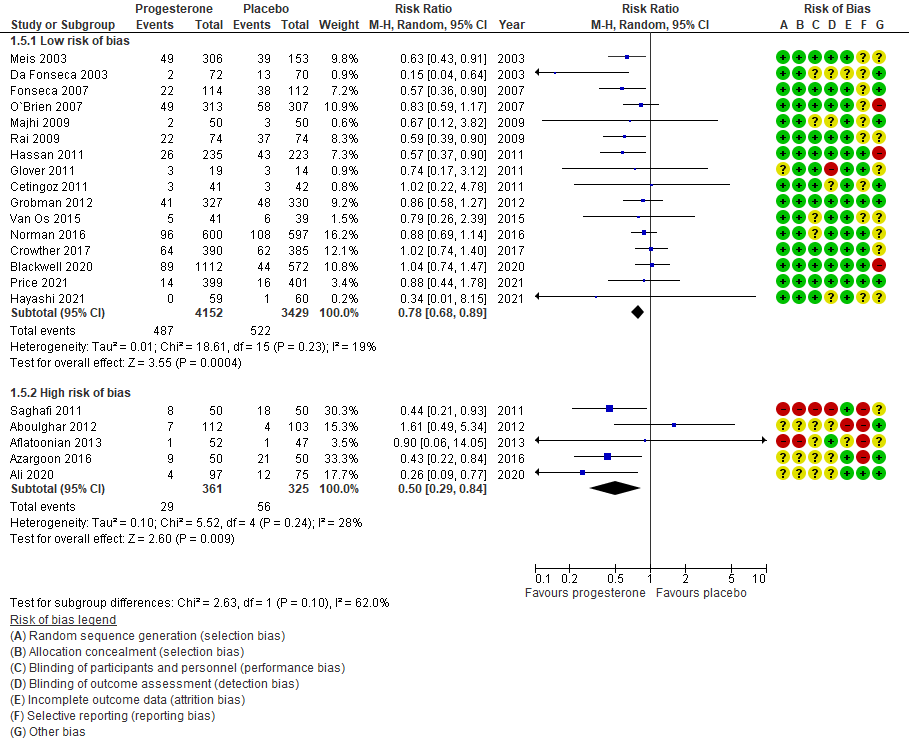


Results from Meis 2003, Fonseca 2007, O’Brien 2007, Hassan 2011, Glover 2011, Crowther 2017, Blackwell 2020 were retrieved from the IPD meta-analysis by Stewart et al. (EPPPIC, 2021).

Conclusion: Progesterone compared with placebo reduces the risk of any preterm birth before 34 gestational weeks in women with a singleton pregnancy, neither considering administration route and dosage, nor type of risk factor for preterm birth (GRADE ⊕⊕⊕⊕).

**SFigure 5.** Outcome: Any preterm birth <34 weeks. **Cetingoz et al. 2011 excluded from analysis** (article retracted 2024).


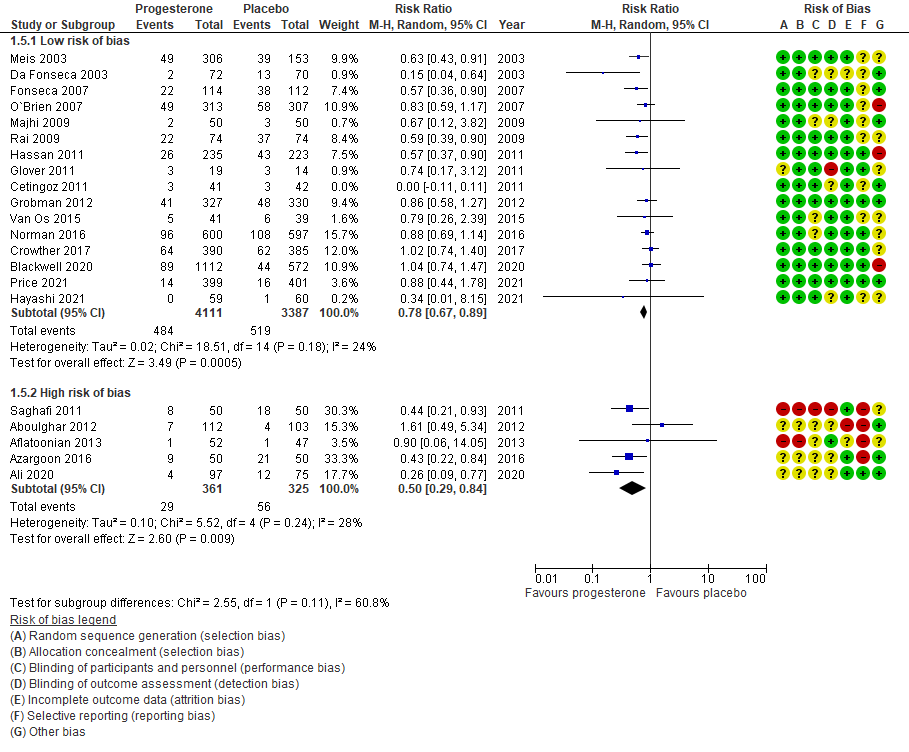


**Spontaneous preterm birth <34** weeks (Appendix 4.1, STable 4.1.3.b and SFigure 6)

A meta-analysis of two trials with low risk of bias, including 330 women (Fonseca et al., 2007 with 9.6% twins included) showed a significant reduction in the rate of spontaneous preterm birth; RR 0.57 (95% CI 0.38 to 0.86). A sensitivity analysis excluding Fonseca et al., 2007 due to inclusion of 9.6% twins, removed 92% of the sample size and resulted in a very imprecise estimate (RR 0.71 (95% CI 0.17 to 2.99). The crude event rate across trials was 28.6% without progesterone. The pooled weighted RD was -9.4 percentage points (95% CI -22.3 to 3.4).

**SFigure 6.** Outcome: Spontaneous preterm birth <34 weeks.


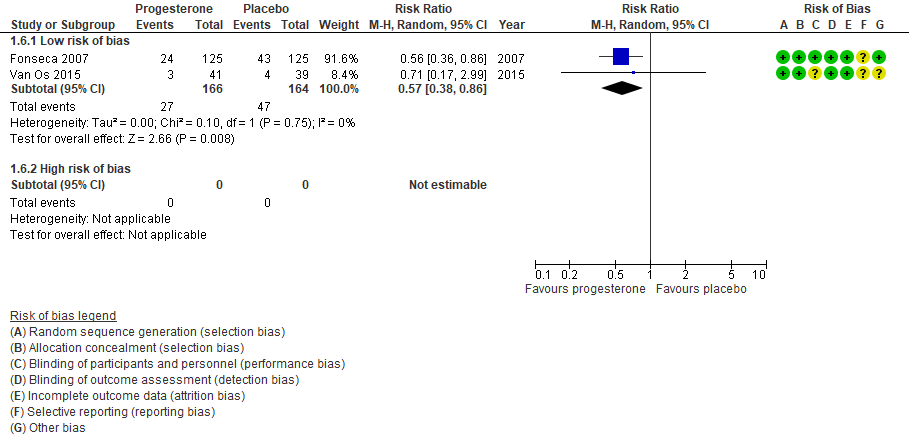


Conclusion**:** Vaginal progesterone, compared with placebo may reduce the risk of spontaneous preterm birth before 34 gestational weeks in women with a singleton pregnancy and short cervical length (GRADE ⊕⊕🌕🌕).

**Any preterm birth <33 weeks** (Appendix 4.1, STable 4.1.4.a and SFigure 7)

A meta-analysis of five trials with low risk of bias, including 974 women showed a significant reduction in the rate of preterm birth, RR 0.63 (95% CI 0.48 to 0.83). The crude event rate across trials was 22.5% without progesterone. The pooled weighted RD was -8.4 percentage points (95% CI -13.0 to -3.8).

**SFigure 7**. Outcome: Any preterm birth <33 weeks. All patients had a short cervical length according to inclusion criteria (Hassan and Fonseca) or constitute subgroups of the other trials.


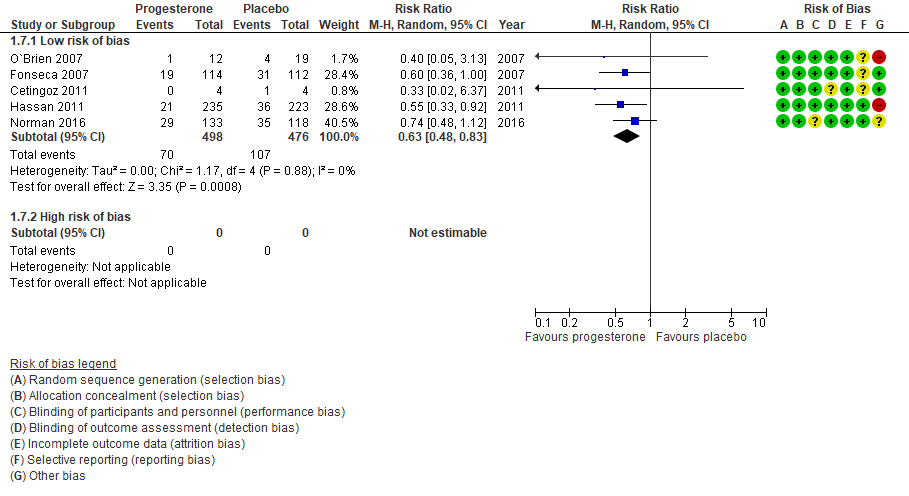


Results for singletons from O’Brien 2007, Fonseca 2007, Cetingoz 2011, and Norman 2016 were retrieved from the IPD meta-analysis by Romero et al., 2018.

Cut-off for cervical length was ≤25 mm in all trials but two, Fonseca 2007 used ≤15 mm and Hassan 2011 included 10-20 mm.

Conclusion: Vaginal progesterone, not considering dosage, compared with placebo probably reduces the risk of any preterm birth before 33 gestational weeks in women with a singleton pregnancy and short cervical length (GRADE ⊕⊕⊕ 🌕).

**SFigure 7**. Outcome: Any preterm birth <33 weeks. All patients had a short cervical length according to inclusion criteria (Hassan and Fonseca) or constitute subgroups of the other trials**. Cetingoz et al. 2011 excluded from analysis** (article retracted 2024).


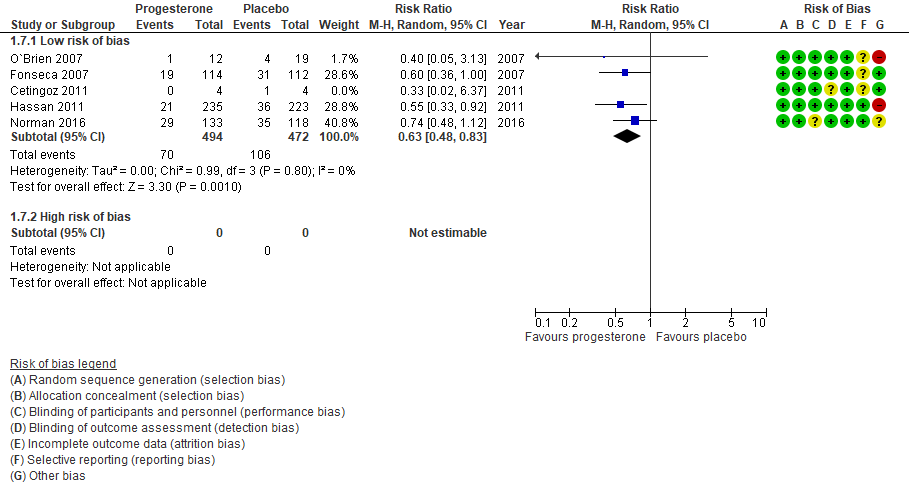


**Spontaneous preterm birth <33 weeks**

No trial reported on spontaneous preterm birth <33 weeks.

**Any preterm birth <32 weeks** (Appendix 4.1, STable 4.1.5.a and SFigure 8)

A meta-analysis of six trials with low risk of bias, including 3645 women showed no difference in the rate of any preterm birth, RR 0.71 (95% CI 0.50 to 1.01). The crude event rate across trials was 10.1% without progesterone. The pooled weighted RD was -4.6 percentage points (95% CI -9.2 to -0.1).

**SFigure 8.** Outcome: Any preterm birth <32 weeks.


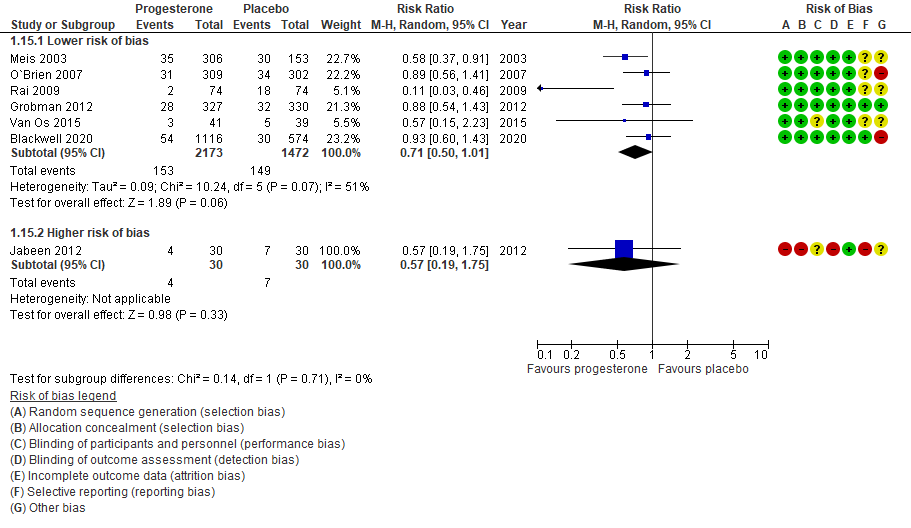


Conclusion: Progesterone compared with placebo may result in no difference in the risk of any preterm birth before 32 gestational weeks, although the CI may imply a reduced risk, in women with a singleton pregnancy, neither considering administration route and dosage, nor type of risk factor for preterm birth (GRADE ⊕⊕🌕🌕).

**Spontaneous birth <32 weeks** (Appendix 4.1, STable 4.1.5.b and SFigure 9)

A meta-analysis of two trials with low risk of bias, including 1770 women showed no difference in the rate of spontaneous preterm birth, RR 0.84 (95% CI 0.51 to 1.39). The crude event rate across trials was 4.1% without progesterone. The pooled weighted RD was -0.6 percentage points (95% CI -2.5 to 1.2).

**SFigure 9.** Outcome: Spontaneous preterm birth <32 weeks.


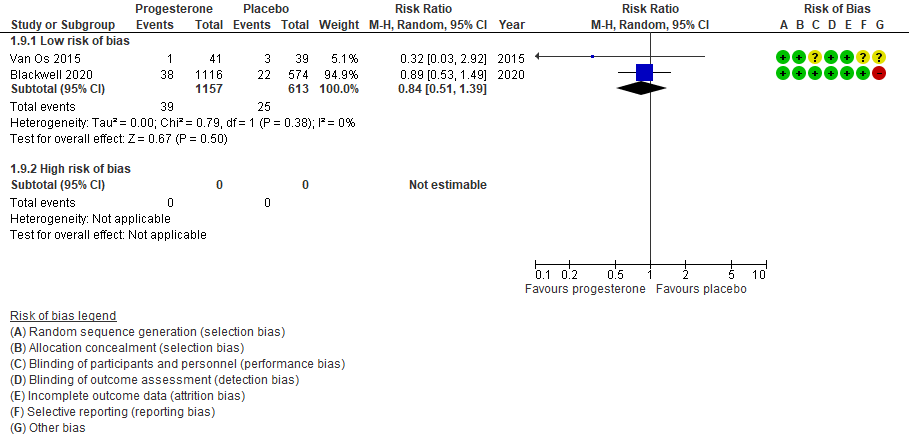


Conclusion: Progesterone compared with placebo may result in no difference in the risk of spontaneous preterm birth before 32 gestational weeks in women with a singleton pregnancy, neither considering administration route and dosage, nor type of risk factor for preterm birth (GRADE ⊕⊕🌕 🌕).

**Any preterm birth <28 weeks** (Appendix 4.1, STable 4.1.6.a and SFigure 10)

A meta-analysis of six trials with low risk of bias, including 2793 women showed a reduction in the rate of any preterm birth, RR 0.64 (95% CI 0.44 to 0.95). The crude event rate across trials was 4.5% without progesterone. The pooled weighted RD was -1.4 percentage points (95% CI -3.1 to 0.2).

**SFigure 10.** Outcome: Any preterm birth <28 weeks.


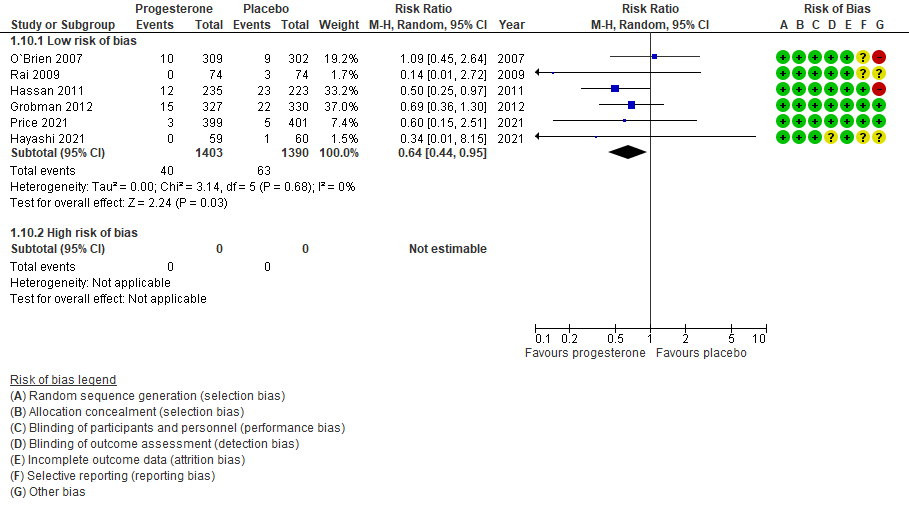


Conclusion: Progesterone compared with placebo probably reduces the risk of any preterm birth before 28 gestational weeks in women with a singleton pregnancy, neither considering administration route and dosage, nor type of risk factor for preterm birth (GRADE ⊕⊕⊕🌕).

**Spontaneous birth <28 weeks**

No trial reported on spontaneous preterm birth <28 weeks.

**Gestational age and birth weight in singletons**

**Gestational age** (Appendix 4.1, STable 4.1.7 and SFigure 11)

A meta-analysis of six trials with low risk of bias, including 1778 women showed no mean difference in gestational age, 0.60 (-0.27 to 1.47) weeks, corresponding to approximately four days longer (two days less to ten days longer) gestational length in the progesterone group.

# SFigure 11. Outcome: Gestational age at delivery (weeks).


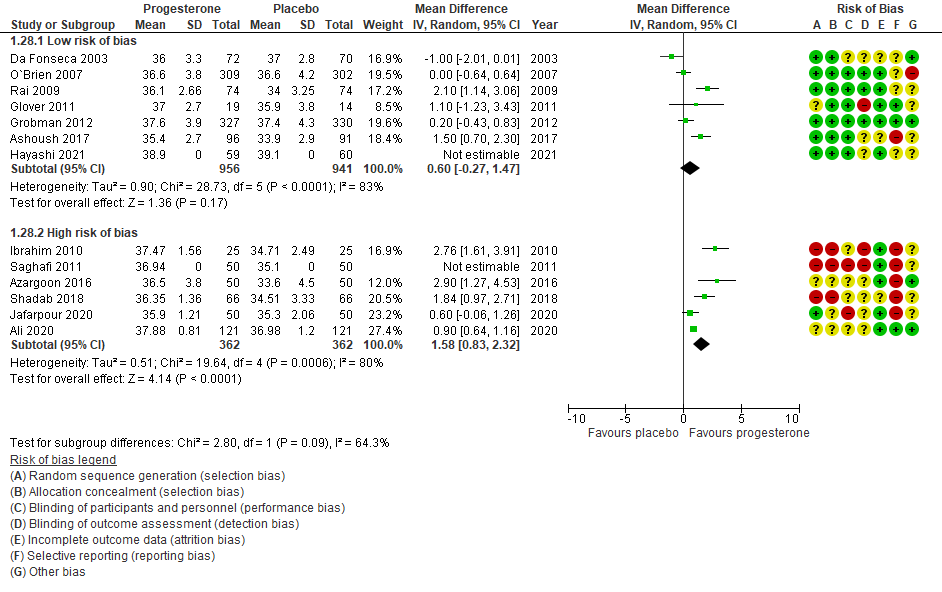


Conclusion: Progesterone compared with placebo may result in no difference in gestational age in women with a singleton pregnancy, neither considering administration route and dosage, nor type of risk factor for preterm birth (GRADE ⊕⊕🌕🌕).

**Low birth weight** (Appendix 4.1, STable 4.1.8 and SFigure 12)

A meta-analysis of seven trials with low risk of bias, including 2888 neonates showed a reduction in the rate of low birth weight, RR 0.81 (95% CI 0.69 to 0.96). A sensitivity analysis excluding Fonseca et al., 2007 (due to inclusion of 9.6% twins) did not alter the result (RR 0.78 (95% CI 0.66 to 0.94). The crude event rate across trials was 26.9% without progesterone. The pooled weighted RD was -6.2 percentage points (95% CI -12.2 to -0.1).

**SFigure 12.** Outcome: Low birth weight (<2500 g).


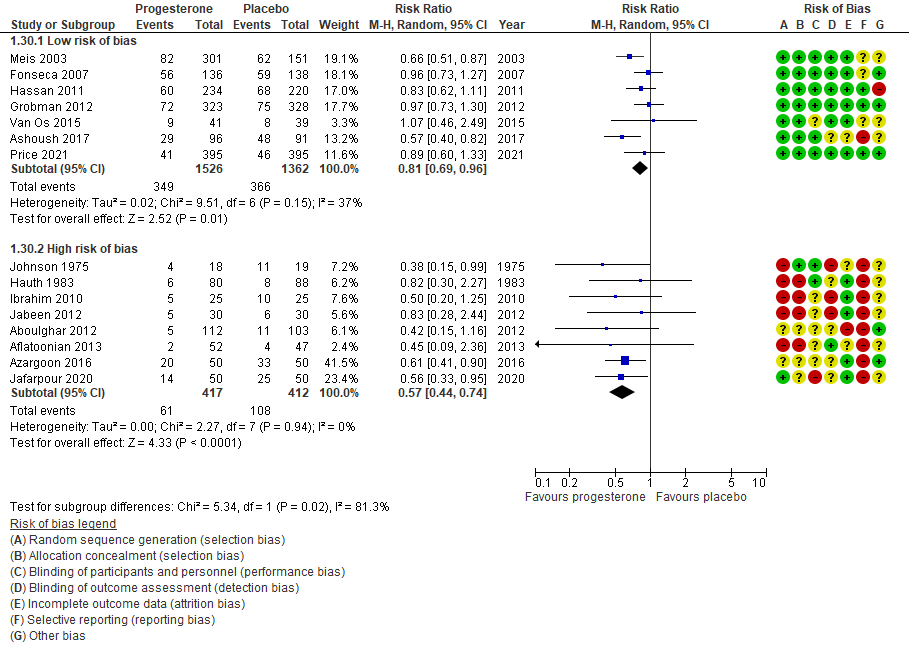


Johnson 1975 included 2.7% twins.

Conclusion: Progesterone compared with placebo probably reduces the risk of low birthweight in singletons, neither considering administration route and dosage, nor type of maternal risk factor for preterm birth (GRADE ⊕⊕⊕🌕).

**Very low birth weight** (Appendix 4.1, STable 4.1.9 and SFigure 13)

A meta-analysis of six trials with low risk of bias, including 2701 neonates showed a reduction in the risk of very low birth weight, RR 0.66 (95% CI 0.50 to 0.85). A sensitivity analysis excluding Fonseca et al., 2007 (due to inclusion of 9.6% twins) did not alter the result (RR 0.65 (95% CI 0.48 to 0.87). The crude event rate across trials was 9.3% without progesterone. The pooled weighted RD was -3.6 percentage points (95% CI -7.3 to 0.2).

**SFigure 13.** Outcome: Very low birth weight (<1500 g).


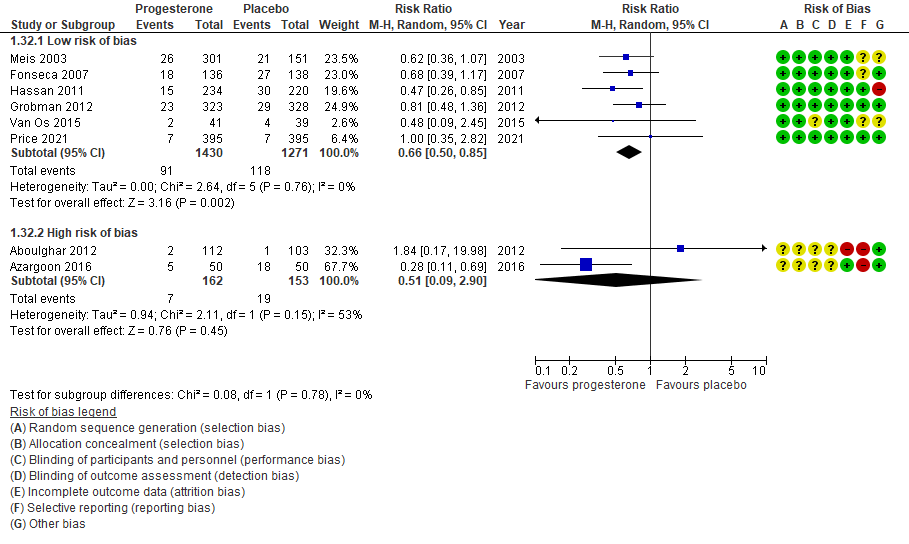


Conclusion: Progesterone compared with placebo reduces the risk of very low birth weight in singletons, neither considering administration route and dosage, nor type of maternal risk factor for preterm birth (GRADE ⊕⊕⊕⊕).

**Mortality and morbidity in neonates from singleton pregnancies**

**Perinatal mortality** (Appendix 4.1, STable 4.1.10)

Twelve trials reported perinatal mortality in singleton pregnancies. No meta-analysis was performed due to different or lack of definitions in the trials.

**Neonatal mortality <7 days**

No trial reported mortality <7 days.

**Neonatal mortality <28 days** (Appendix 4.1, STable 4.1.11 and SFigure 14)

A meta-analysis of 12 trials with low risk of bias, including 7169 neonates, showed a reduction in the rate of neonatal mortality <28 days, RR 0.60 (95% CI 0.39 to 0.92). The crude event rate across trials was 2.4% without progesterone. The pooled weighted RD was -0.7 percentage points (95% CI -1.7 to 0.4).

# SFigure 14. Outcome: Neonatal mortality <28 days.


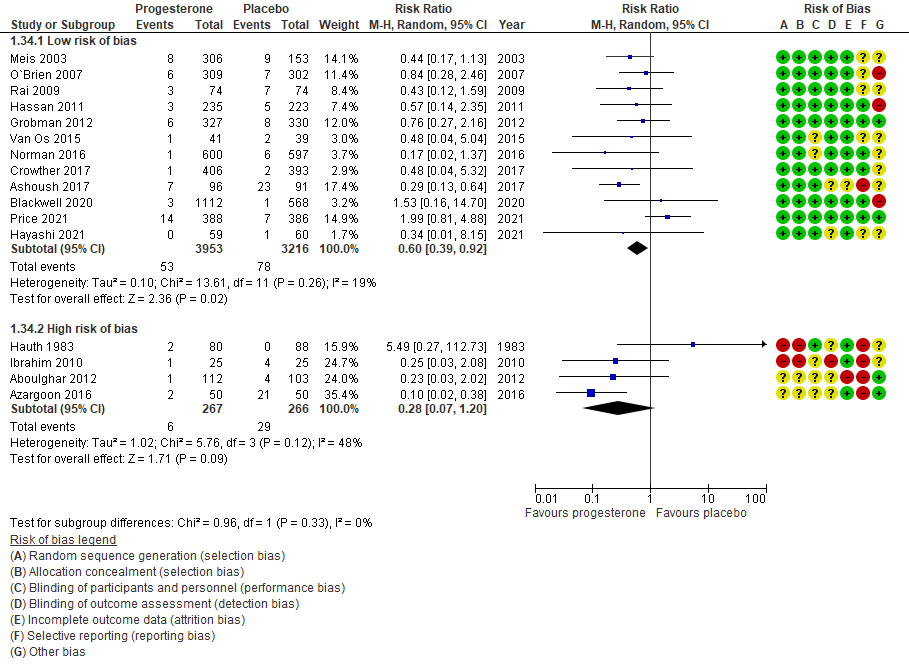


Conclusion: Progesterone compared with placebo may reduce the risk of neonatal mortality <28 days in singletons, neither considering administration route and dosage, nor type of maternal risk factor for preterm birth (GRADE ⊕⊕⊕🌕).

**Composite adverse neonatal outcome** (Appendix 4.1, STable 4.1.12 and SFigure 15)

A meta-analysis of five trials with low risk of bias, including 4742 neonates showed no difference in the rate of composite adverse neonatal outcome, RR 0.83 (95% CI 0.66 to 1.06). The crude event rate across trials was 14.3% without progesterone. The pooled weighted RD was -1.9 percentage points (95% CI -4.3 to 0.5).

Four trials included in the composite adverse outcome any of intrauterine fetal death, neonatal death, intraventricular hemorrhage, periventricular leukomalacia, necrotizing enterocolitis, bronchopulmonary dysplasia, respiratory distress syndrome, or confirmed sepsis. Grobman et al., 2012 and Crowther et al., 2017 also included retinopathy of prematurity and Crowther et al., 2017 also included low Apgar score <4 at 5 min and small for gestational age (<3^rd^ centile). Norman et al., 2016 defined the composite outcome as any of neonatal mortality, brain injury on ultrasound, or bronchopulmonary dysplasia.

**SFigure 15.** Outcome: Composite adverse neonatal outcome.


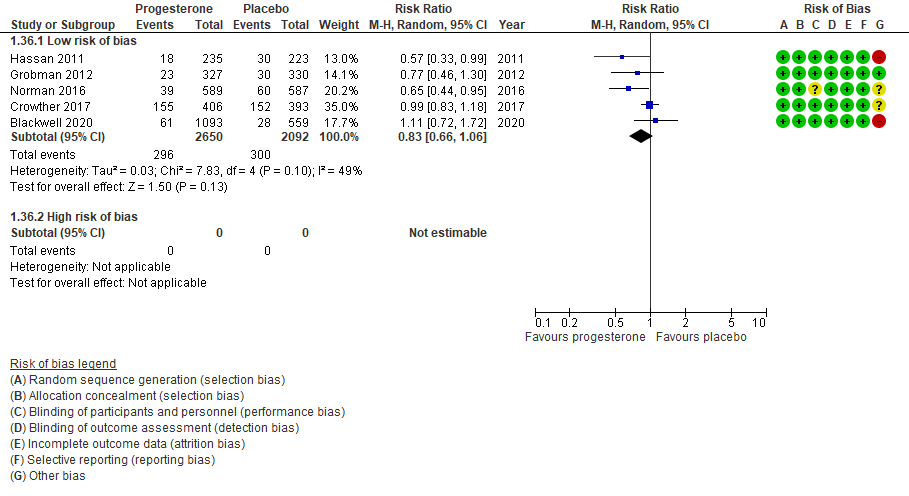


Conclusion: Progesterone compared with placebo, may result in no difference in the risk of a composite outcome of neonatal morbidity in singletons, neither considering administration route and dosage, nor type of maternal risk factor for preterm birth (GRADE ⊕⊕🌕🌕).

**Respiratory distress syndrome (RDS)** (Appendix 4.1, STable 4.1.13 and SFigure 16)

A meta-analysis of nine studies with low risk of bias, including 4636 neonates showed a significant reduction in the rate of RDS, RR 0.70 (95% CI 0.57 to 0.87). The crude event rate across trials was 10.0% without progesterone. The pooled weighted RD was -3.2 percentage points (95% CI -5.9 to -0.5).

**SFigure 16.** Outcome: Respiratory distress syndrome.


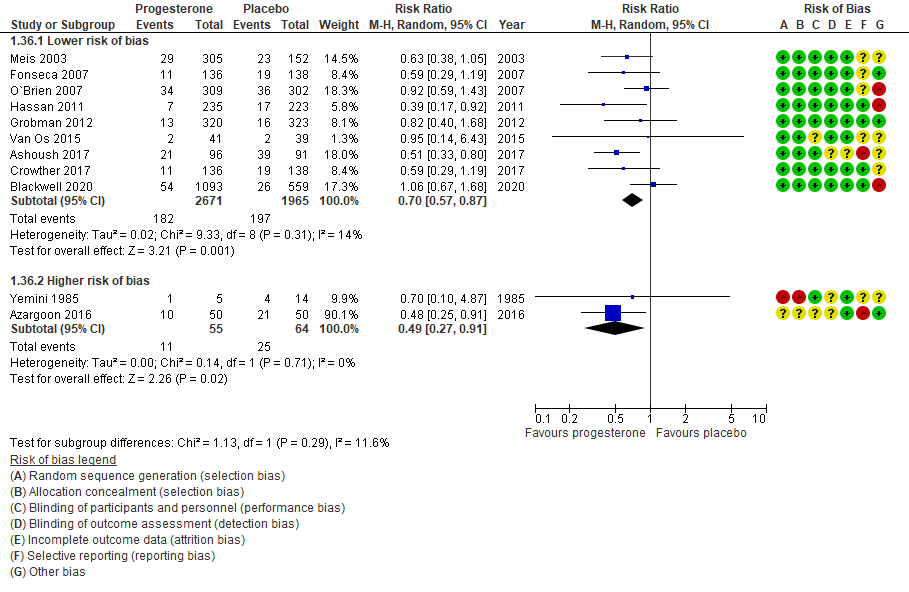


Conclusion: Progesterone compared with placebo probably reduces the risk of RDS in singletons, neither considering administration route and dosage, nor type of maternal risk factor for preterm birth (GRADE ⊕⊕⊕🌕).

**Bronchopulmonary dysplasia (BPD)** (Appendix 4.1, STable 4.1.14 and SFigure 17)

A meta-analysis of seven trials with low risk of bias, including 5233 neonates showed no difference in the rate of BPD, RR 0.89 (95% CI 0.58 to 1.37). The crude event rate across trials was 1.9% without progesterone. The pooled weighted RD was 0.1 percentage points (95% CI -0.4 to 0.7).

**SFigure 17.** Outcome: Bronchopulmonary dysplasia.


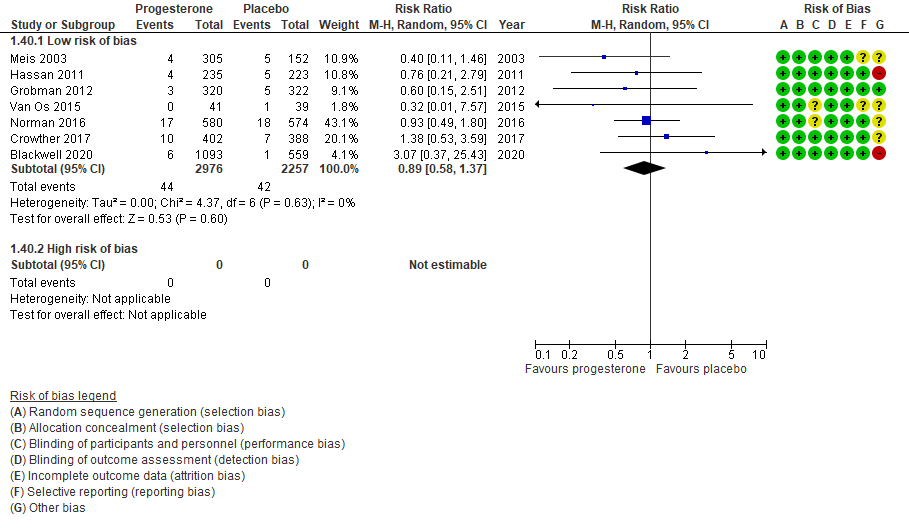


Conclusion: Progesterone compared with placebo may result in no difference in BPD in singletons, neither considering administration route and dosage, nor type of maternal risk factor for preterm birth (GRADE ⊕⊕🌕🌕).

**Intraventricular hemorrhage (IVH)** (Appendix 4.1, STable 4.1.15 and SFigure 18)

A meta-analysis of 10 trials with low risk of bias, including 6310 neonates showed no difference in the rate of IVH, RR 0.67 (95% CI 0.45 to 1.02). A sensitivity analysis excluding Fonseca et al., 2007 (due to inclusion of 9.6% twins), yielded a RR of 0.69 (95% CI 0.45 to 1.04). The crude event rate across trials was 1.8% without progesterone. The pooled weighted RD was -0.3 percentage points (95% CI -1.0 to 0.4).

**SFigure 18.** Outcome: Intraventricular hemorrhage.


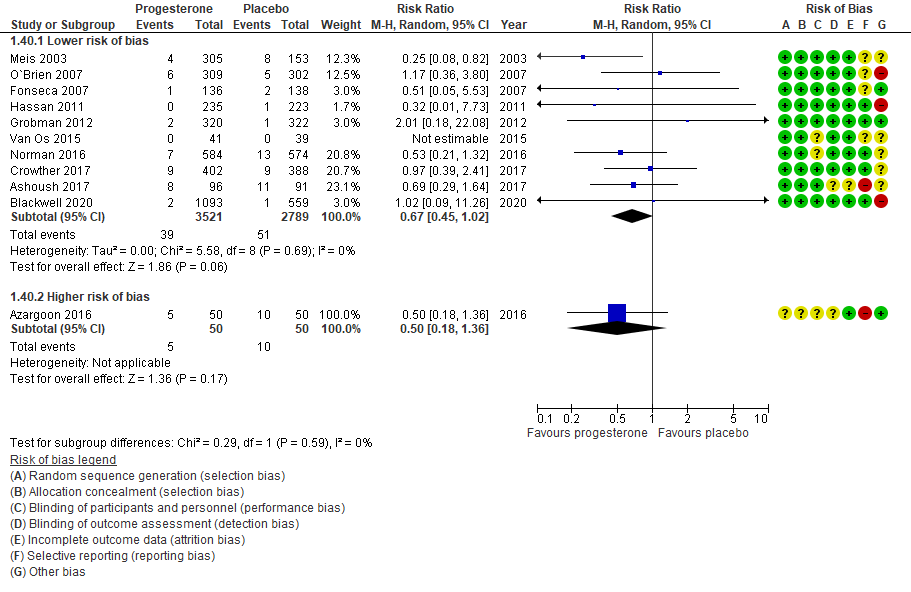
Conclusion: Progesterone compared with placebo may result in no difference in the risk of IVH in singletons, although the CI may imply a reduced risk, neither considering administration route and dosage, nor type of maternal risk factor for preterm birth
(GRADE ⊕⊕🌕🌕).

**Necrotizing enterocolitis (NEC)** (Appendix 4.1, STable 4.1.16 and SFigure 19)

A meta-analysis of 11 trials with low risk of bias, including 6406 neonates showed no difference in the rate of NEC, RR 0.80 (95% CI 0.51 to 1.23). A sensitivity analysis excluding Fonseca et al., 2007 (due to inclusion of 9.6% twins), yielded a RR of 0.81 (0.52 to 1.26). The crude event rate across trials was 1.6% without progesterone. The pooled weighted RD was -0.3 percentage points (95% CI -0.7 to 0.2).

**SFigure 19.** Outcome: Necrotizing enterocolitis.


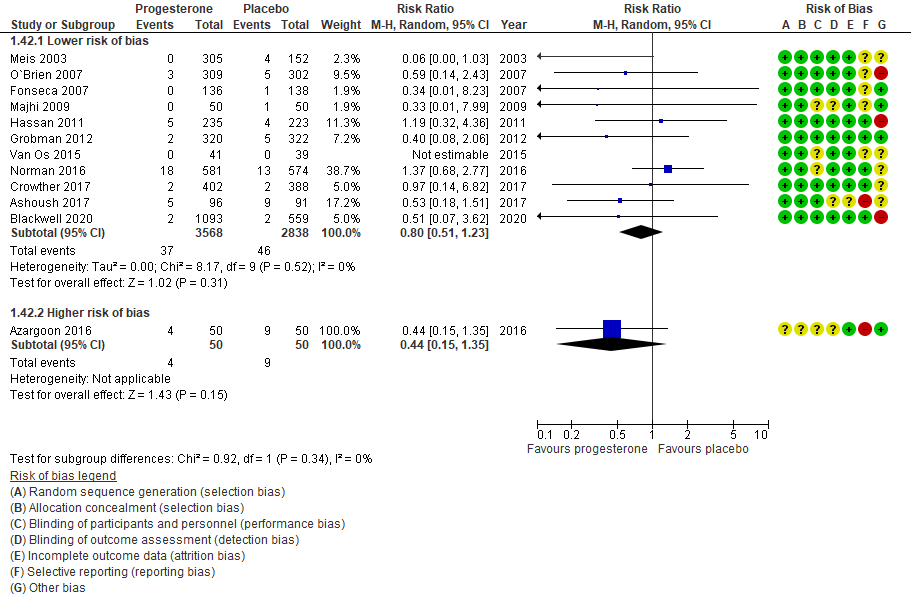


Conclusion: Progesterone compared with placebo may result in no difference in the risk of NEC in singletons, neither considering administration route and dosage, nor type of maternal risk factor for preterm birth (GRADE ⊕⊕🌕🌕).

**Neonatal sepsis** (Appendix 4.1, STable 4.1.17 and SFigure 20)

A meta-analysis of nine trials with low risk of bias, including 5563 neonates showed no difference in the rate of neonatal sepsis, RR 0.70 (95% CI 0.39 to 1.24). A sensitivity analysis excluding Fonseca et al., 2007 (due to inclusion of 9.6% twins), yielded a RR of 0.85 (95% CI 0.50 to 1.45). The crude event rate across trials was 3.1% without progesterone. The pooled weighted RD was -0.5 percentage points (95% CI -1.4 to 0.4).

**SFigure 20.** Outcome: Neonatal sepsis.


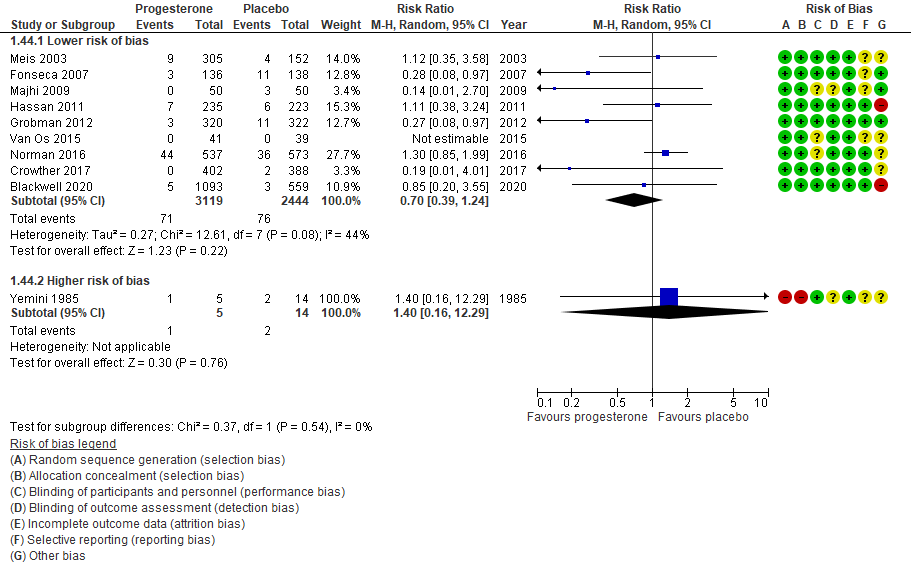


Conclusion: Progesterone compared with placebo may result in no difference in the risk of neonatal sepsis or other infection in singletons, neither considering administration route and dosage, nor type of maternal risk factor for preterm birth (GRADE ⊕⊕🌕🌕).

**Retinopathy of prematurity (ROP)** (Appendix 4.1, STable 4.1.18 and SFigure 21)

A meta-analysis of five trials with low risk of bias, including 3812 neonates showed no difference in the rate of ROP, RR 0.71 (95% CI 0.34 to 1.45). A sensitivity analysis excluding Fonseca et al., 2007 (due to inclusion of 9.6% twins), yielded a RR of 0.65 (95% CI 0.33 to 1.29). The crude event rate across trials was 1.5% without progesterone. The pooled weighted RD was -0.4 percentage points (95% CI -1.2 to 0.3).

**SFigure 21.** Outcome: Retinopathy of prematurity.


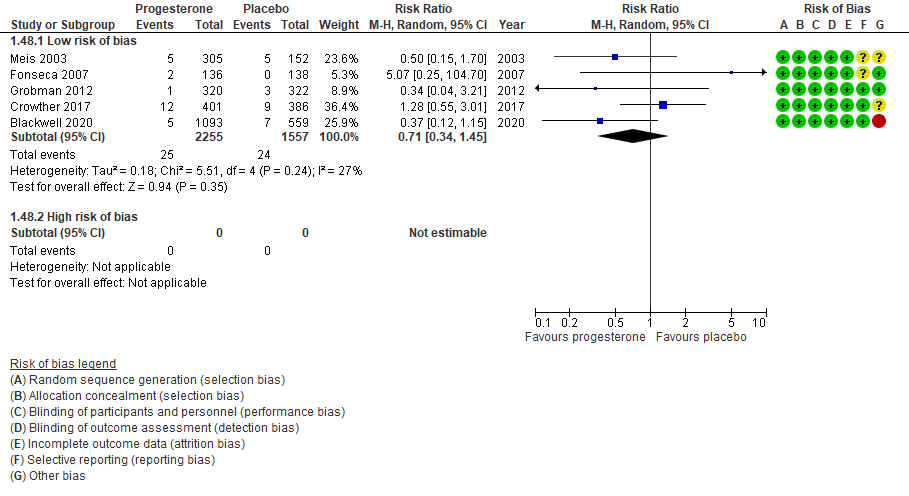


Conclusion: Progesterone compared with placebo may result in no difference in the risk of ROP in singletons, neither considering administration route and dosage, nor type of maternal risk factor for preterm birth (GRADE ⊕⊕🌕🌕).

**Admittance to neonatal intensive care unit (**Appendix 4.1, STable 4.1.19 and SFigure 22)

A meta-analysis of ten studies with low risk of bias, including 5272 neonates showed no difference in the rate of NICU admission, RR 0.77 (95% CI 0.60 to 1.00). A sensitivity analysis excluding Fonseca et al., 2007 (due to inclusion of 9.6% twins), yielded a RR of 0.76 (0.57 to 1.02). The crude event rate across trials was 17.7% without progesterone. The pooled weighted RD was -6.1 percentage points (95% CI -11.0 to -1.3).

**SFigure 22.** Outcome: Admittance to neonatal intensive care unit (NICU).


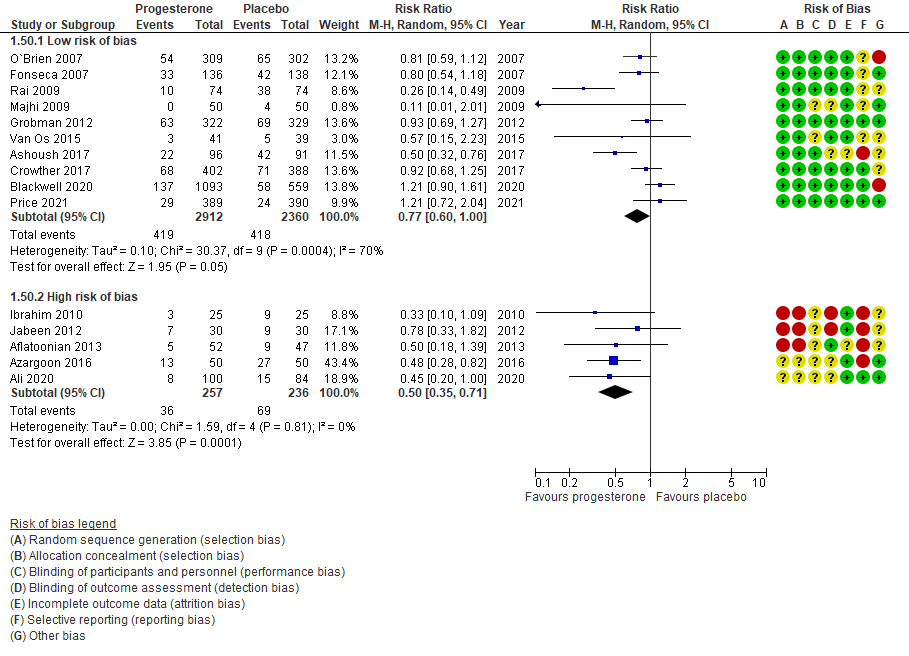


Conclusion: Progesterone compared with placebo may result in no difference in admittance to NICU of singletons, although the CI may imply a reduction, neither considering administration route and dosage, nor type of maternal risk factor for preterm birth
(GRADE ⊕⊕🌕🌕).

**Long-term child outcomes in singletons** (Appendix 4.1, STable 4.1.20)

Three trials (1715 children) examined long-term child outcome in singletons (Cuijpers et al., 2020 [follow up of Van Os et al., 2015, Triple P]), Norman et al., 2016 [OPPTIMUM], Northen et al., 2007 [follow up of Meis et al., 2003]). All three trials were included in a systematic review by Simons et al., 2020. Follow-up rate was between 71 and 80%. A meta-analysis of two reports (Cuijpers et al., 2020, n=57 children [unpublished data), Norman et al., 2018 [OPPTIMUM], n=833 children) showed no difference in neurodevelopment assessed by the Bayley-III Cognitive Composite score at two years between children exposed to progesterone versus placebo (Standardised Mean Difference -0.04, 95% CI -0.26 to 0.19) (Simons et al., 2020). Northen et al., 2007 used the Ages and Stages Questionnaire at 4 to 5 years of age and found no difference between the groups. General health, anthropometry and behaviour were similar between the groups.

Conclusion: Progesterone compared with placebo may result in no difference in cognitive development, general health or behaviour in singletons, neither considering administration route, nor type of maternal risk factor for preterm birth.

**Mortality and morbidity in women with singleton pregnancies**

**Maternal mortality** (Appendix 4.1, STable 4.1.21)

One trial from Zambia on an HIV-population reported one maternal death in the placebo group (1/401).

**Hypertensive disorders in pregnancy (HDP) (**Appendix 4.1 STable 4.1.22 and SFigure 23)

A meta-analysis of five trials with low risk of bias, including 4665 women showed no difference in the rate of HDP, RR 0.97 (95% CI 0.74 to 1.27). The crude event rate across trials was 4.0% without progesterone. The pooled weighted RD was 0.1 percentage points (95% CI -1.1 to 1.2).

# SFigure 23. Outcome: Hypertensive disorder in pregnancy.


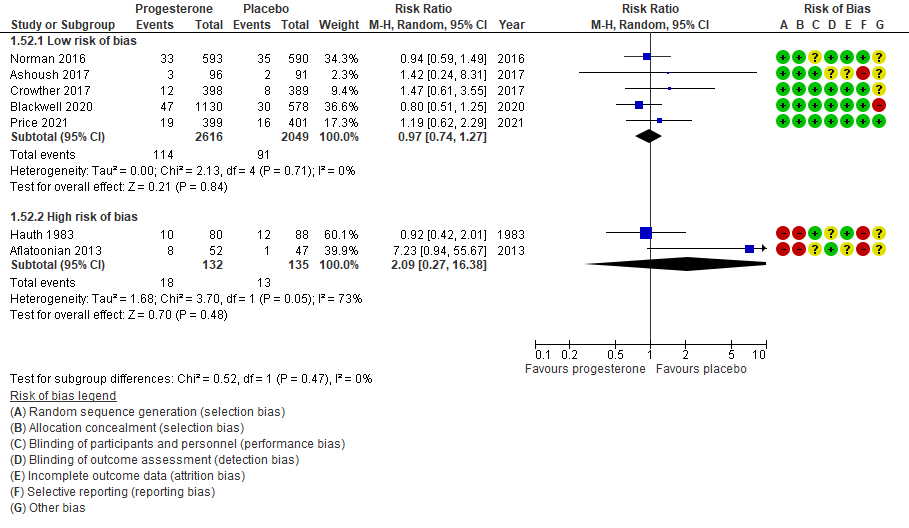


Conclusion: Progesterone compared with placebo probably results in no difference in the risk HDP in women with a singleton pregnancy, neither considering administration route and dosage, nor type of risk factor for preterm birth (GRADE ⊕⊕⊕🌕).

**Gestational diabetes mellitus (GDM) (**Appendix 4.1, STable 4.1.23 and SFigure 24)

A meta-analysis of four trials with low risk of bias, including 3519 women showed no difference in the rate of GDM, RR 0.83 (95% CI 0.60 to 1.15). The crude event rate across trials was 4.8% without progesterone. The pooled weighted RD was -0.8 percentage points (95% CI -2.1 to 0.5).

**SFigure 24.** Outcome: Gestational diabetes mellitus.


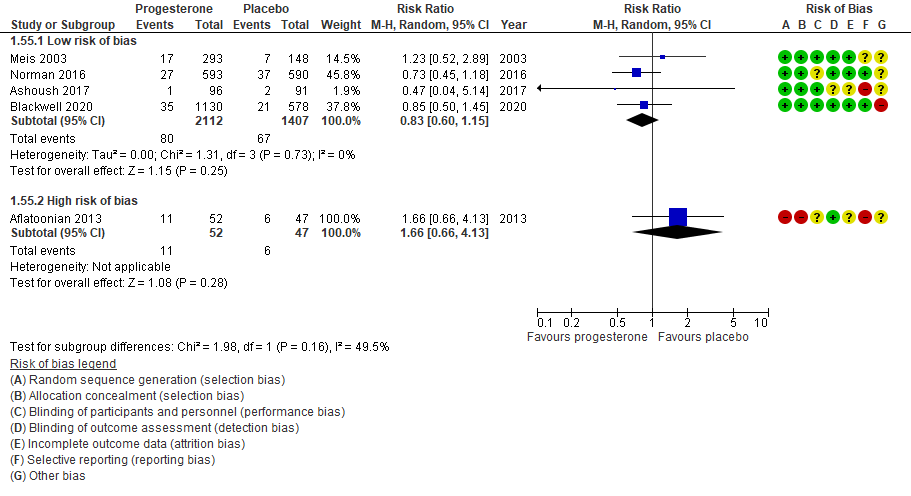


Data from Meis 2003 was retrieved from a secondary publication, Gyamfi 2009, reporting specifically on GDM.

Conclusion: Progesterone compared with placebo may result in no difference in the risk of GDM in women with a singleton pregnancy, neither considering administration route and dosage, nor type of risk factor for preterm birth (GRADE ⊕⊕🌕🌕).

**Intrahepatic cholestasis in pregnancy (ICP) (**Appendix 4.1, STable 4.1.24 and SFigure 25)

A meta-analysis of two trials with low risk of bias (one without events), including 1369 women showed no difference in the rate of ICP, RR 0.66 (95% CI 0.19 to 2.33). The crude event rate across trials was 0.9% without progesterone. The RD was -0.3 percentage points (95% CI -1.2 to 0.7).

**SFigure 25.** Outcome: Intrahepatic cholestasis.


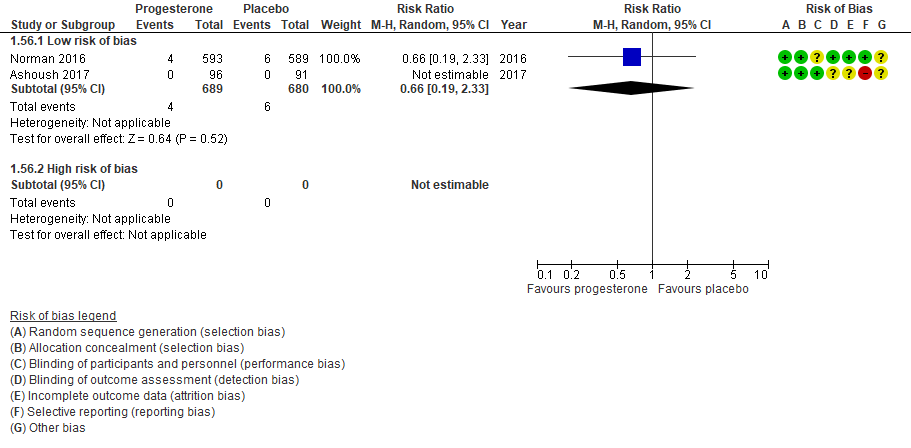


Conclusion: Progesterone compared with placebo may result in no difference in the risk of ICP in women with a singleton pregnancy, neither considering administration route and dosage, nor type of risk factor for preterm birth (GRADE ⊕⊕🌕🌕).

**Infections (chorioamnionitis) (**Appendix 4.1, STable 4.1.25 and SFigure 26)

A meta-analysis of six trials with low risk of bias, including 4021 women showed no difference in the rate of chorioamnionitis, RR 1.16 (95% CI 0.75 to 1.80). The crude event rate across trials was 2.0% without progesterone. The pooled weighted RD was 0.4 percentage points (95% CI -0.1 to 0.8).

**SFigure 26.** Outcome: Chorioamnionitis.


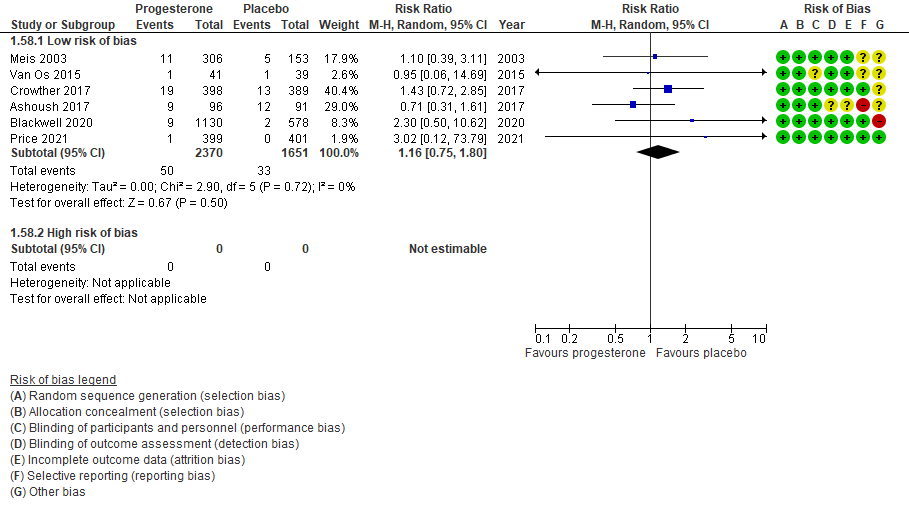


Conclusion: Progesterone compared with placebo may result in no difference in the risk of chorioamnionitis in women with a singleton pregnancy, neither considering administration route and dosage, nor type of risk factor for preterm birth (GRADE ⊕⊕🌕🌕).

**Preterm prelabor rupture of the membranes (PPROM) (**Appendix 4.1, STable 4.1.26 and SFigure 27)

A meta-analysis of six trials with low risk of bias, including 3648 women showed no difference in the rate of PPROM, RR 0.93 (95% CI 0.78 to 1.11). The crude event rate across trials was 11.4% without progesterone. The pooled weighted RD was -0.5 percentage points (95% CI -1.9 to 0.8).

**SFigure 27.** Outcome: Preterm prelabor rupture of membranes (PPROM).


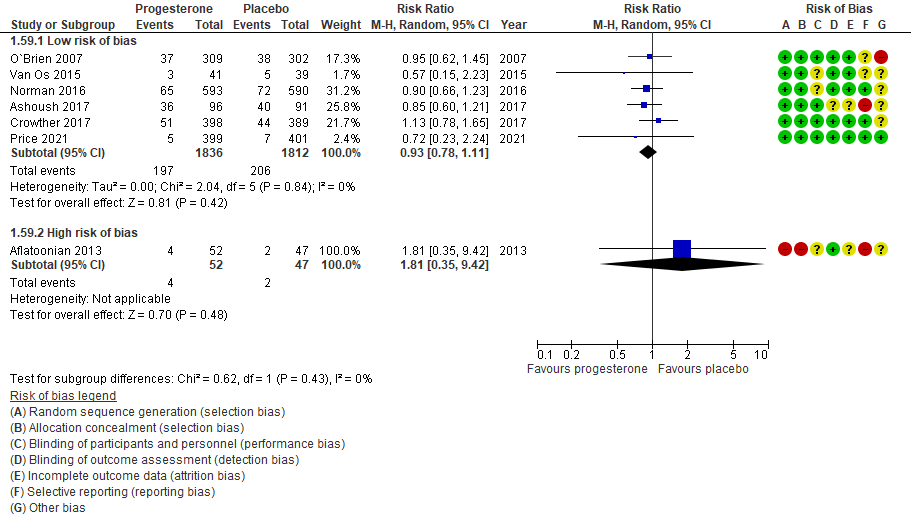


Conclusion: Progesterone compared with placebo results in no difference in the risk of PPROM in women with a singleton pregnancy, neither considering administration route and dosage, nor type of risk factor for preterm birth (GRADE ⊕⊕⊕⊕).

**Progesterone and adverse effects**

No trials or cohort studies reporting on cancer in the woman were identified from the separate search addressing the intervention progesterone and the outcome cancer in the woman

Subgroup analyses

Exploratory subgroup analyses among trials with low risk of bias were performed for administration route (vaginal progesterone, im 17-OHPC or oral progesterone) and pre-specified subgroup analyses for specific risk factors (history of spontaneous preterm birth or short cervical length) were performed among trials with low risk of bias. No trials reporting results in subgroups of patients with cervical surgical treatment for cervical intraepithelial neoplasia were identified. Also, within each stratum of administration route, subgroup analyses based on history of spontaneous preterm birth and short cervical length were conducted. Meta-analyses were performed for any preterm birth before 37 and 34 weeks.

### STable 2 shows the summary estimates from the meta-analyses.

STable 2. Summary estimates from subgroup meta-analyses exploring the effect of progesterone vs placebo according to administration route and specific risk factors in women with a singleton pregnancy.

| **Outcomes**  **Administration route**  **Risk factor** | **Number of RCTs**  **(women)** | **Relative effect**  **RR (95% CI)**  In bold if difference is statistically significant  RR <1 indicates favourable outcome of progesterone | **Absolute effect**  **(%)** |
| --- | --- | --- | --- |
| **Administration route** |  |  |  |
| Any PTB <37 weeks |  |  |  |
| 17-OHPC im | 4 (3600) | 0.89 (0.69 to 1.16) | 22.4 vs 22.3 |
| Vaginal progesterone | 8 (2416) | 0.85 (0.71 to 1.02) | 33.6 vs 37.1 |
| Oral progesterone | 2 (335) | **0.69 (0.55 to 0.85)** | 42.4 vs 61.8 |
| Any PTB <34 weeks |  |  |  |
| 17-OHPC im | 4 (3600) | 0.84 (0.66 to 1.06) | 9.0 vs 10.1 |
| Vaginal progesterone | 10 (3800) | **0.77 (0.62 to 0.94)** | 14.0 vs 17.8 |
| Oral progesterone | 2 (181) | **0.60 (0.40 to 0.90)** | 26.9 vs 45.5 |
| **Risk factor** |  |  |  |
| Any PTB <37 weeks |  |  |  |
| History of spontaneous preterm birth | 9 (4189) | **0.78 (0.65 to 0.94)** | 30.4 vs 36.6 |
| Short cervix | 4 (1314) | 0.93 (0.71 to 1.22) | 24.8 vs 26.4 |
| Any PTB <34 weeks |  |  |  |
| History of spontaneous preterm birth | 9 (4044) | **0.78 (0.62 to 0.98)** | 11.9 vs 15.7 |
| Short cervix | 5 (1540) | **0.68 (0.53 to 0.86)** | 12.1 vs 17.8 |
| **Administration route:**  **17-OHPC im**  **Risk factor:**  **history of preterm birth** |  |  |  |
| Any PTB <37 weeks | 2 (2143) | 0.84 (0.52 to 1.34) | 26.0 vs 28.8 |
| Any PTB <34 weeks | 2 (2143) | 0.81 (0.49 to 1.34) | 9.7 vs 11.4 |
| **Administration route:**  **17-OHPC im**  **Risk factor: short cervix** |  |  |  |
| Any PTB <37 weeks | 1 (657) | 1.03 (0.79 to 1.35) | 25.1 vs 24.2 |
| Any PTB <34 weeks | 1 (657) | 0.86 (0.58 to 1.27) | 12.5 vs 14.5 |
| **Administration route: vaginal**  **Risk factor:**  **history of preterm birth** |  |  |  |
| Any PTB <37 weeks | 5 (1711) | 0.79 (0.59 to 1.05) | 35.4 vs 38.4 |
| Any PTB <34 weeks | 5 (1720) | 0.81 (0.55 to 1.20) | 13.9 vs 16.3 |
| **Administration route: vaginal**  **Risk factor: short cervix** |  |  |  |
| Any PTB <37 weeks | 3 (657) | 0.81 (0.44 to 1.49) | 24.5 vs 28.6 |
| Any PTB <34 weeks | 4 (883) | **0.59 (0.43 to 0.80)** | 11.8 vs 20.3 |

Exploratory subgroup analyses based on trials with low risk of bias

# **SFigure 28.** Outcome: Any preterm birth <37 weeks according to administration route.


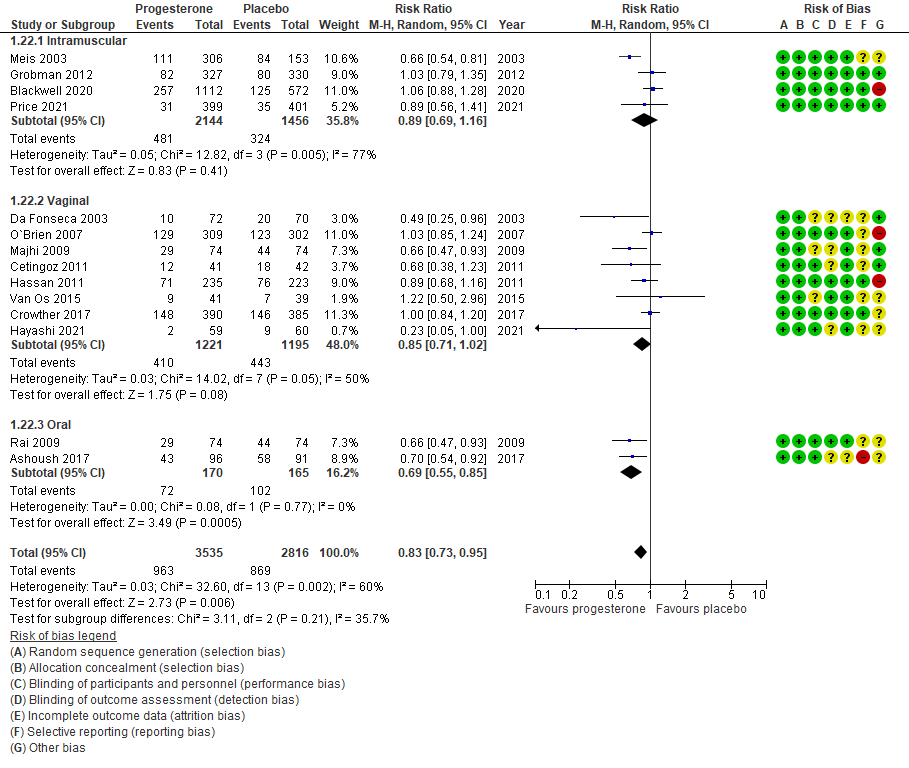


**SFigure 28**. **Outcome: Any preterm birth <37 weeks according to administration route.**

Cetingoz et al. 2011 excluded from analysis (article retracted 2024).


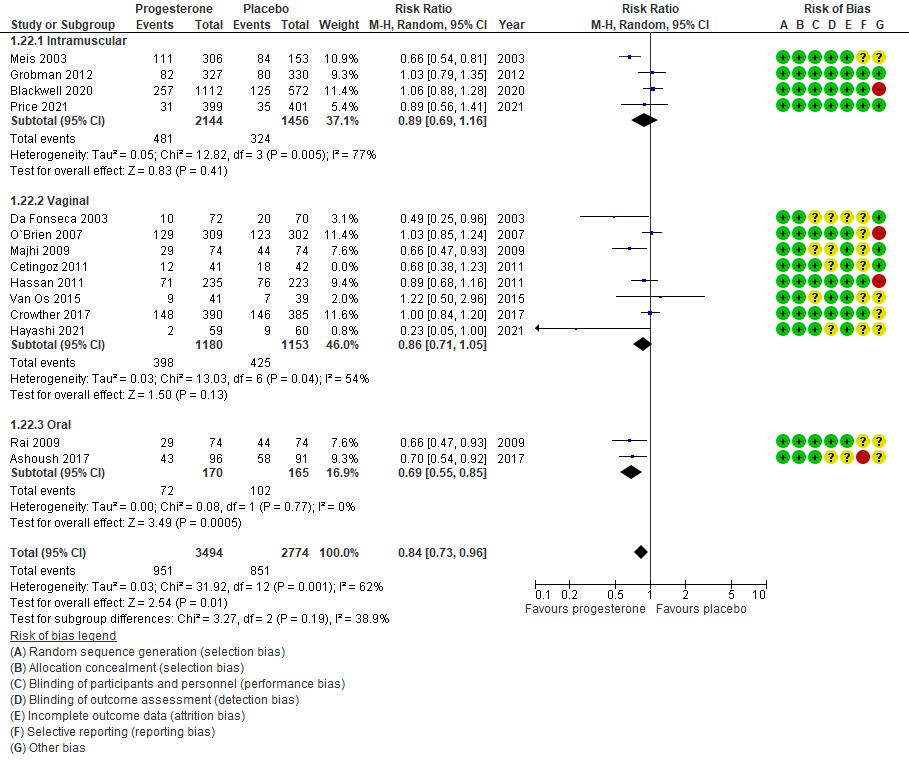


**SFigure 29.** Outcome: Any preterm birth <34 weeks according to administration route.


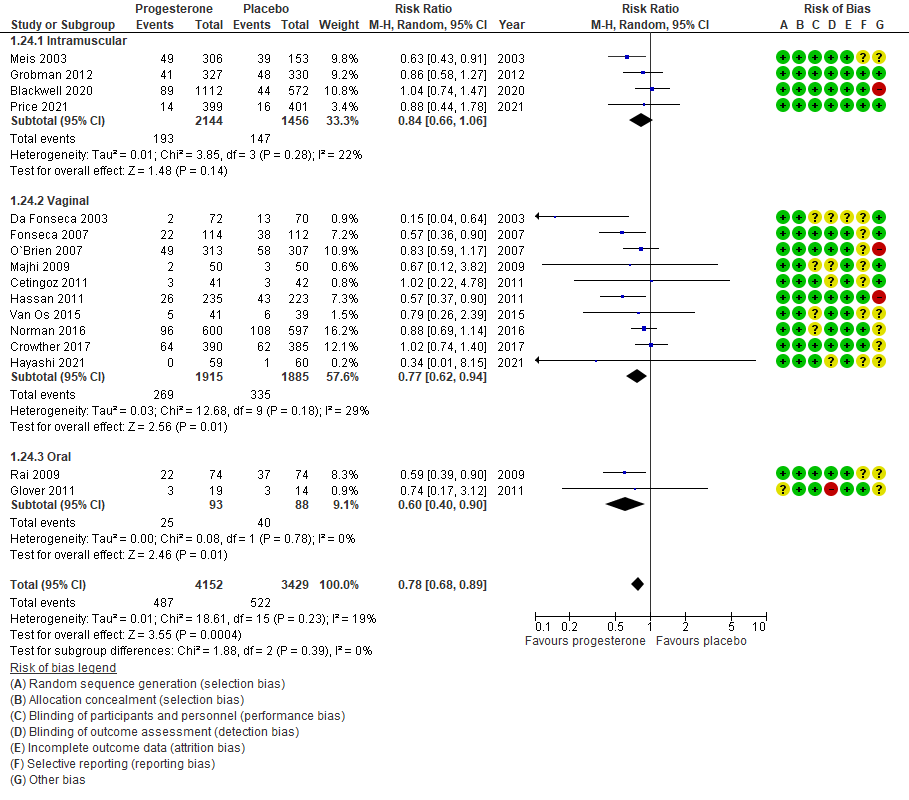


**SFigure 29**. **Outcome: Any preterm birth <34 weeks according to administration route.**

Cetingoz et al. 2011 excluded from analysis (article retracted 2024).


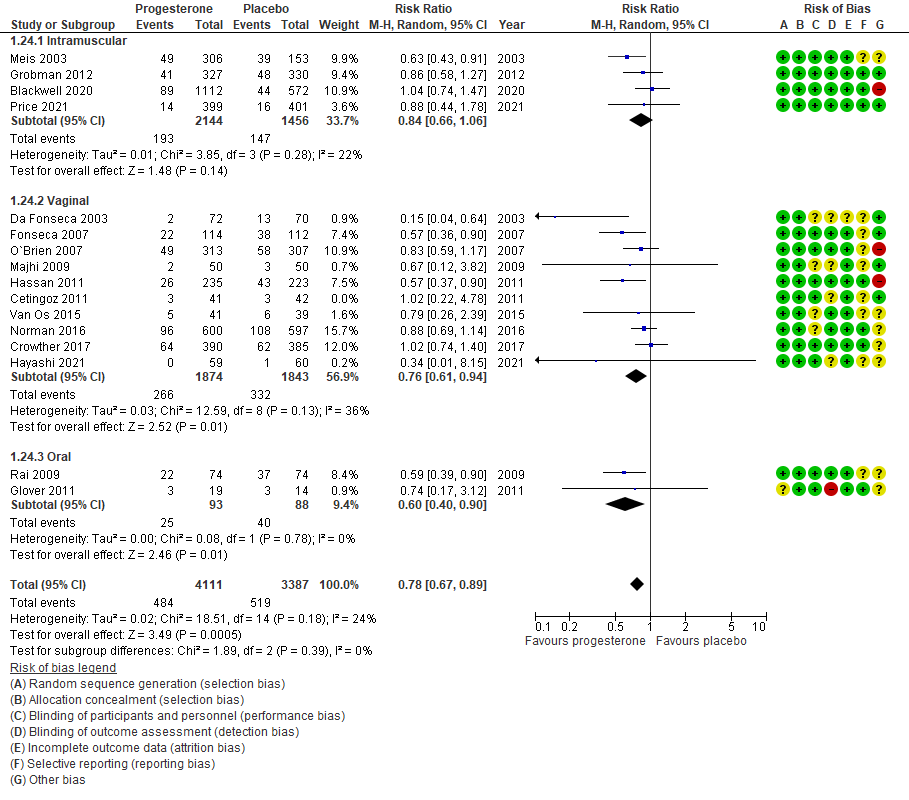


Prespecified subgroup analyses based on trials with low risk of bias

**SFigure 30.** Outcome: Any preterm birth <37 weeks according to risk factor.


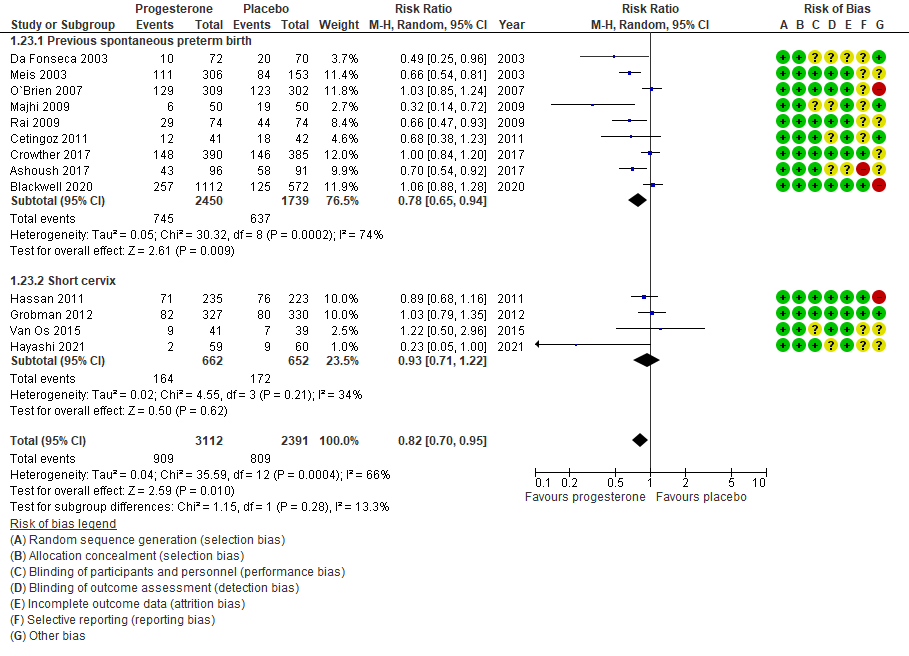


**SFigure 30.** Outcome: Any preterm birth <37 weeks according to risk factor. Cetingoz et al. 2011 excluded from analysis (article retracted 2024).


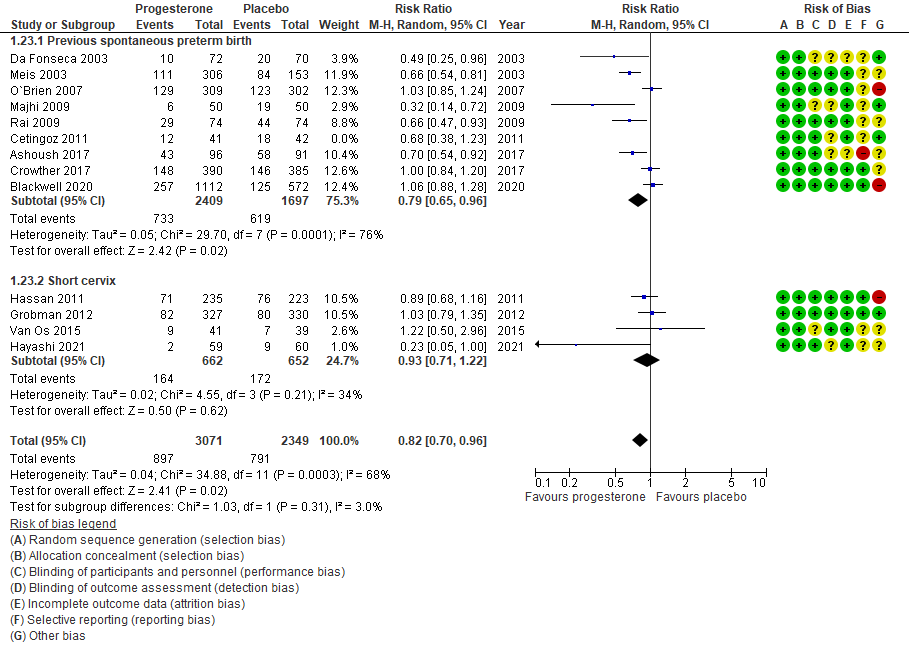


**SFigure 31.** Outcome: Any preterm birth <34 weeks according to risk factor.


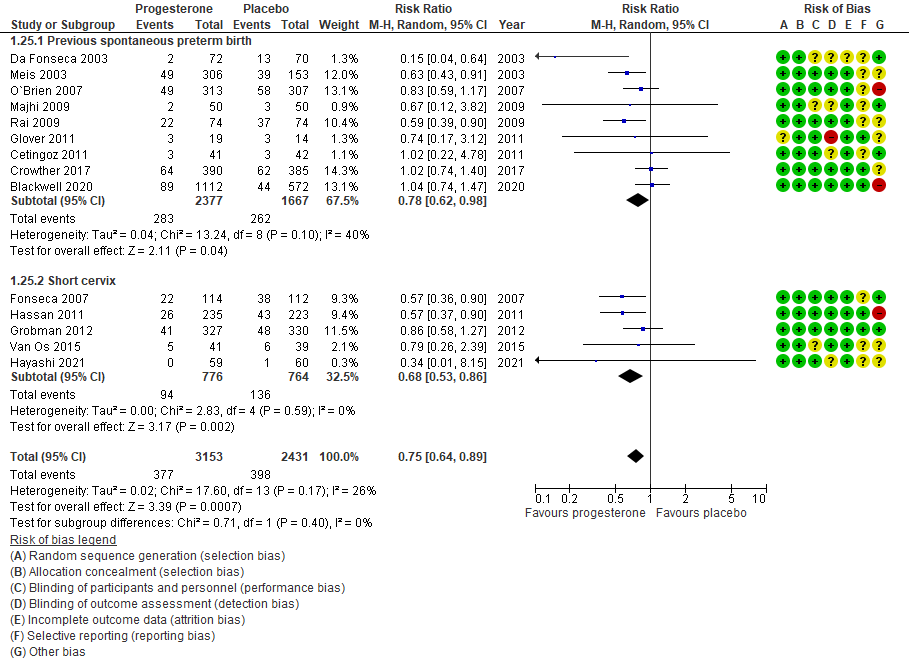


Cut-off cervical length: Fonseca 2007 ≤15 mm, Hassan 2011 10-20 mm, Grobman 2012 <30 mm, Van Os 2015 ≤30, and Hayashi 2021 included 25-<30 mm.

**SFigure 31.** Outcome: Any preterm birth <34 weeks according to risk factor. Cetingoz et al. 2011 excluded from analysis (article retracted 2024).


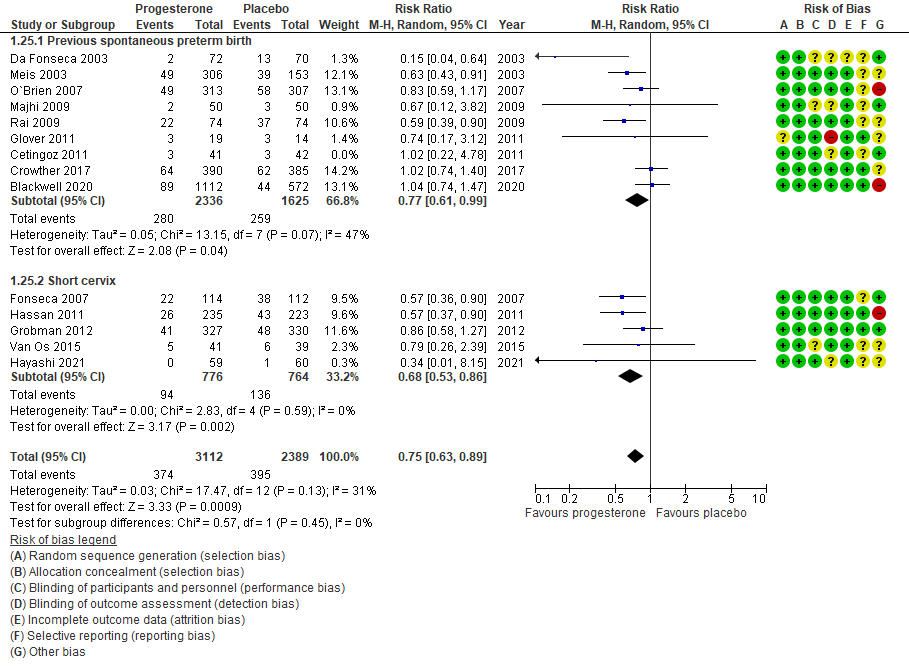


**SFigure 32.** Outcome: Any preterm birth <37 weeks among women with a previous spontaneous preterm birth, according to administration route.


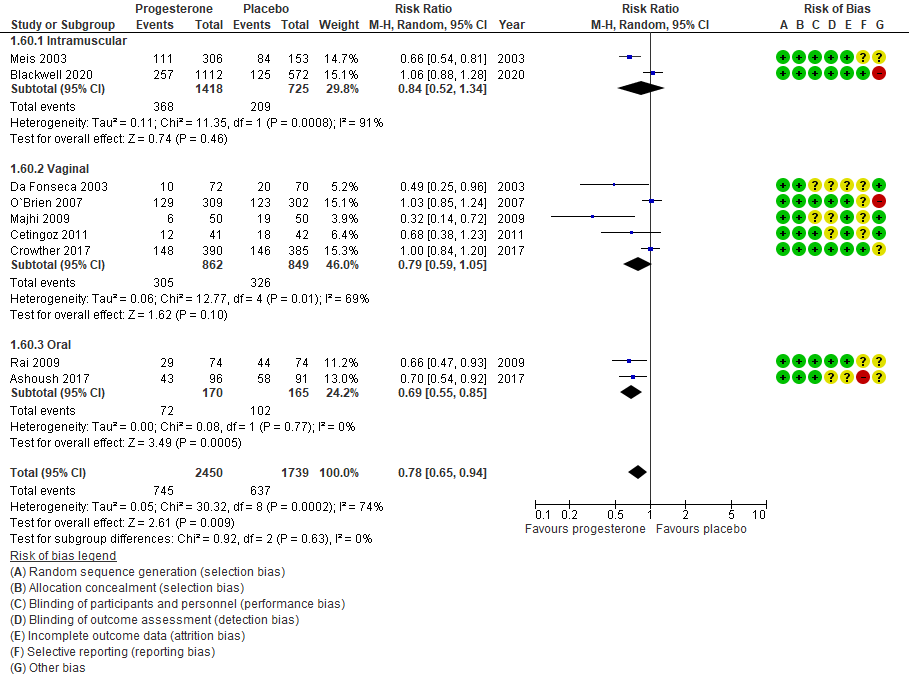


**SFigure 32.** Outcome: Any preterm birth <37 weeks among women with a previous spontaneous preterm birth, according to administration route. Cetingoz et al. 2011 excluded from analysis (article retracted 2024).


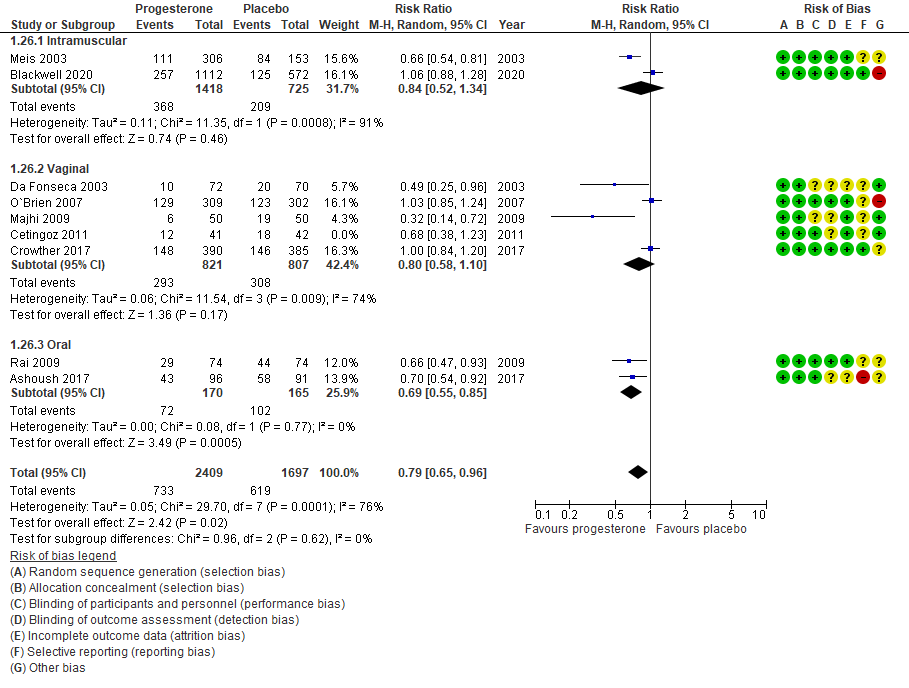


**SFigure 33.** Outcome: Any preterm birth <34 weeks among women with a previous spontaneous preterm birth, according to administration route.


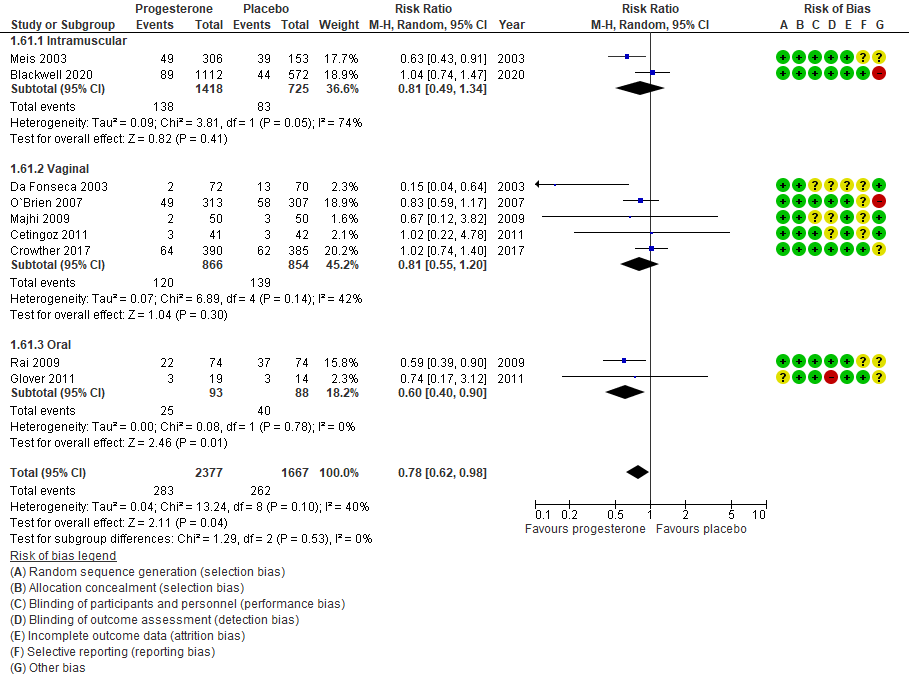


**SFigure 33.** Outcome: Any preterm birth <34 weeks among women with a previous spontaneous preterm birth, according to administration route. Cetingoz et al. 2011 excluded from analysis (article retracted 2024).


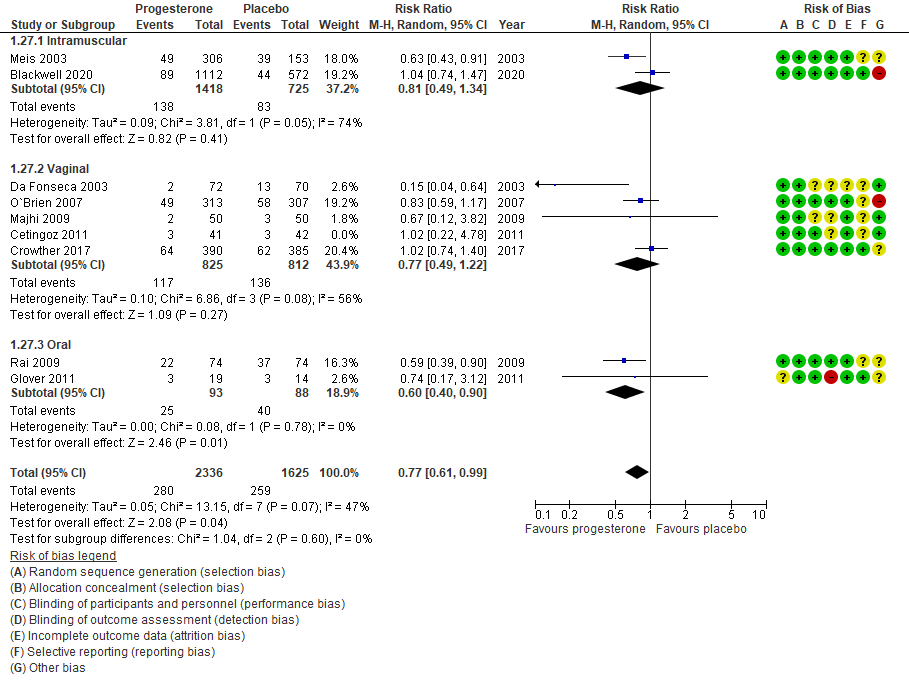


**SFigure 34.** Outcome: Any preterm birth <37 weeks among women with short cervical length, according to administration route.


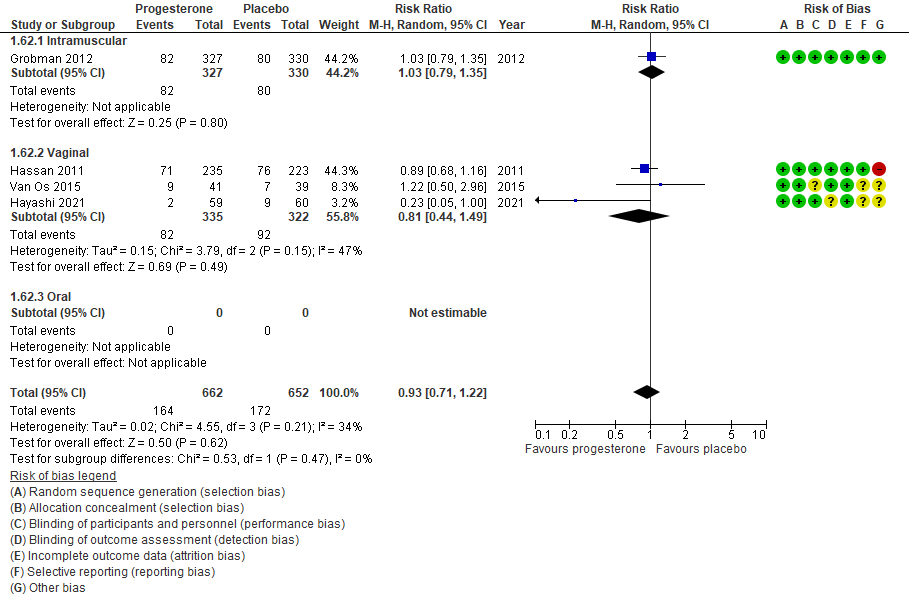


Cut-off cervical length: Grobman 2012 <30 mm, Hassan 2011 included 10-20 mm, Van Os 2015 ≤30 mm and Hayashi 2021 included 25-<30 mm.

**SFigure 35.** Outcome: Any preterm birth <34 weeks among women with short cervical length, according to administration route.


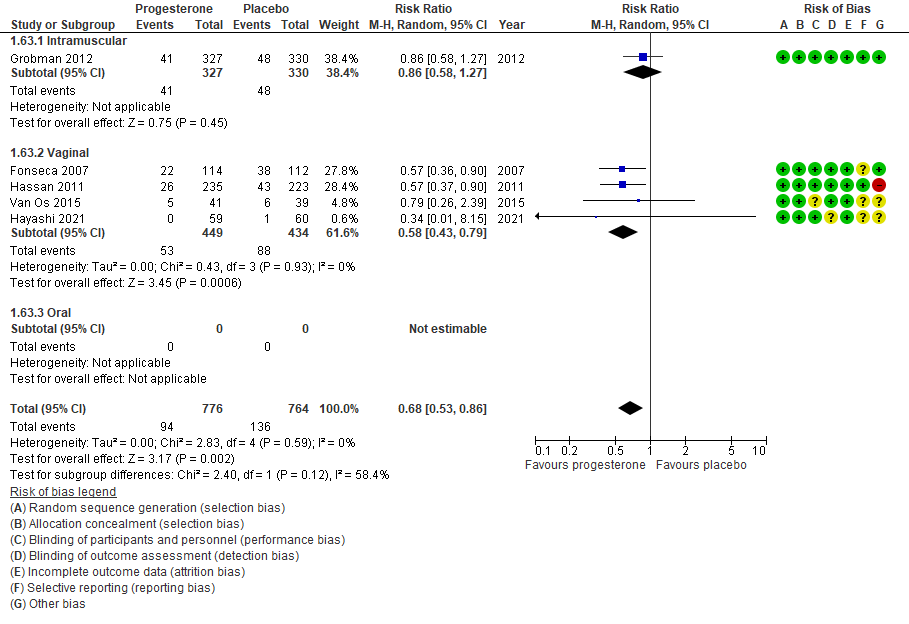


Cut-off for cervical length: Grobman 2012 <30 mm, Fonseca 2007 ≤15 mm, Hassan 2011 included 10-20 mm, Van Os 2015 ≤30 mm, and Hayashi 2021 included 25-<30 mm.
