# Supplementary material for: Progesterone, cerclage, pessary, or acetylsalicylic acid for prevention of preterm birth in singleton and multifetal pregnancies – A systematic review and meta-analyses
Source: Front Med (Lausanne). 2023 Feb 28;10:1111315. doi: 10.3389/fmed.2023.1111315 (PMC10015499; doi:10.3389/fmed.2023.1111315)
Supplement: Supplementary file 1 [file Data_Sheet_1.zip › Data Sheet 1_corrected/Appendix 5.2 Results Cerclage_singletons.docx]

**Progesterone, cerclage, pessary, or acetylsalicylic acid for prevention of preterm birth in singleton and multifetal pregnancies**

**Appendix 5.2 Results cerclage vs no cerclage in singleton pregnancies**

**Table of contents**

[[Abbreviations 2](#_Toc107218693)](#_Toc119661701)

[[STable 1. Risk of bias legend 3](#_Toc107218693)](#_Toc119661702)

[[Results per outcome cerclage vs no cerclage in singleton pregnancies 3](#_Toc107218693)](#_Toc119661703)

[Preterm birth SFigures 1-63-7](#_SFigure_1._Outcome:)

[Gestational age and birth weight SFigures 7-98-9](#_SFigure_7._Outcome:)

[Neonatal mortality and morbidity SFigures 10-199-14](#_SFigure_9._Outcome:)

[Maternal morbidity SFigures 20-2115](#_SFigure_20._Outcome:)

[Subgroup analyses 16](#_Subgroup_analyses_(SFigures)

[Preterm birth SFigures 22-2316](#_SFigure_22._Outcome:)

# Abbreviations

BPD bronchopulmonary dysplasia

CI confidence interval

ICP intrahepatic cholestasis in pregnancy

IVH intraventricular hemorrhage

mm millimetre

NEC necrotizing enterocolitis

PPROM preterm prelabor rupture of membranes

RD risk difference

RDS respiratory distress syndrome

ROP retinopathy of prematurity

RR relative risk/risk ratio

STable 1. Risk of bias legend **to the colour plot within the following forests plots**

1. Random sequence generation (selection bias)
2. Allocation concealment (selection bias)
3. Blinding of participants and personnel (performance bias)
4. Blinding of outcome assessment (detection bias)
5. Incomplete outcome data (attrition bias)
6. Selective reporting (reporting bias)
7. Conflict of interest bias

Results per outcome

Preterm birth **in singletons across gestational weeks**

**Any preterm birth <37 weeks** (Appendix 4.2, STable 4.2.1.a and SFigure 1)

A meta-analysis of four trials with low risk of bias, including 1919 women, showed a significant reduction in the rate of any preterm birth, RR 0.78 (95% CI 0.69 to 0.88). A sensitivity analysis excluding Macnaughton et al., 1993 due to inclusion of miscarriages in numerator and denominator changed the result only marginally (RR 0.72 [95% CI 0.61 to 0.86]). The crude event rate across trials was 37.6% without cerclage. The pooled weighted RD was -9.9 percentage points (95% CI -17.2 to -2.7).

# SFigure 1. Outcome: Any preterm birth <37 weeks.

**
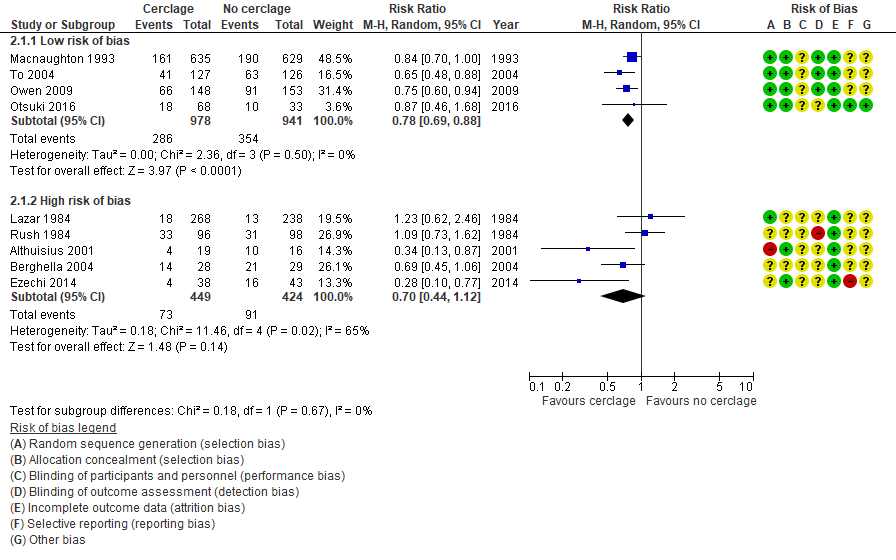
**

Conclusion: Cerclage compared with no cerclage probably reduces the risk of any preterm birth before 37 gestational weeks in women with a singleton pregnancy, not considering type of risk factor for preterm birth (GRADE ⊕⊕⊕ 🌕).

**Spontaneous preterm birth <37 weeks**

No trial reported spontaneous preterm birth <37 weeks.

**Any preterm birth <35 weeks** (Appendix 4.2, STable 4.2, STable 4.2.2.a and SFigure 2**)**
One trial with low risk of bias, including 301 women with a previous spontaneous preterm birth and short cervical length, showed no difference in the rate of any preterm birth, RR 0.76 (95% CI 0.56 to 1.03). The event rate was 41.8% without cerclage. The RD was -10.1 percentage points (95% CI -20.9 to 0.8).

**SFigure 2**. Outcome: Any preterm birth before 35 weeks.


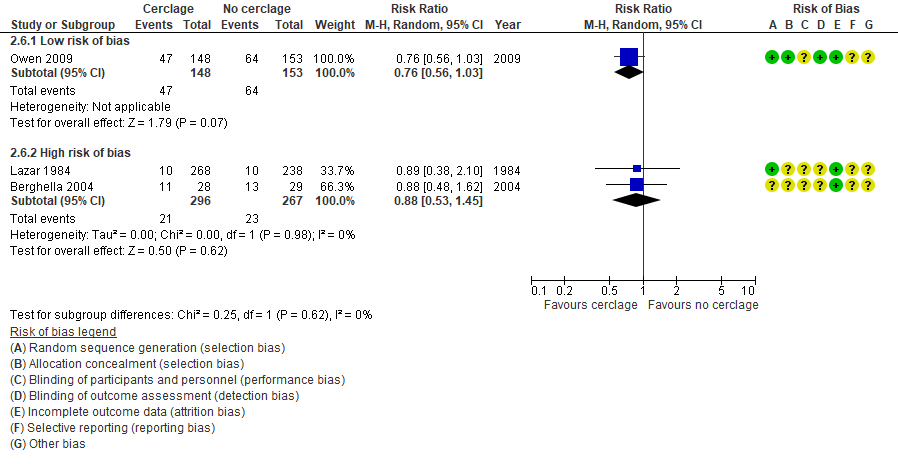


Conclusion: Cerclage compared with no cerclage may result in no difference in the risk of any preterm birth before 35 gestational weeks in women with a singleton pregnancy, previous spontaneous preterm birth, and short cervical length (GRADE ⊕⊕ 🌕🌕).

**Spontaneous preterm birth <35 weeks**

No trial reported spontaneous preterm birth <35 weeks.

**Any preterm birth <34 weeks** (Appendix 4.2, STable 4.2.3.a and SFigure 3)
A meta-analysis of four trials with low risk of bias, including 1919 women, showed a significant reduction in the rate of any preterm birth, RR 0.79 (95% CI 0.66 to 0.94). A sensitivity analysis excluding Macnaughton et al., 1993 due to inclusion of miscarriages in numerator and denominator changed the result marginally, but closer to non-significance (RR 0.77 [95% CI 0.60 to 0.99]). The crude event rate across trials was 22.3% without cerclage. The pooled weighted RD was -4.3 percentage points (95% CI -7.7 to -0.8).

**SFigure 3**. Outcome: Any preterm birth before 34 weeks in singletons.


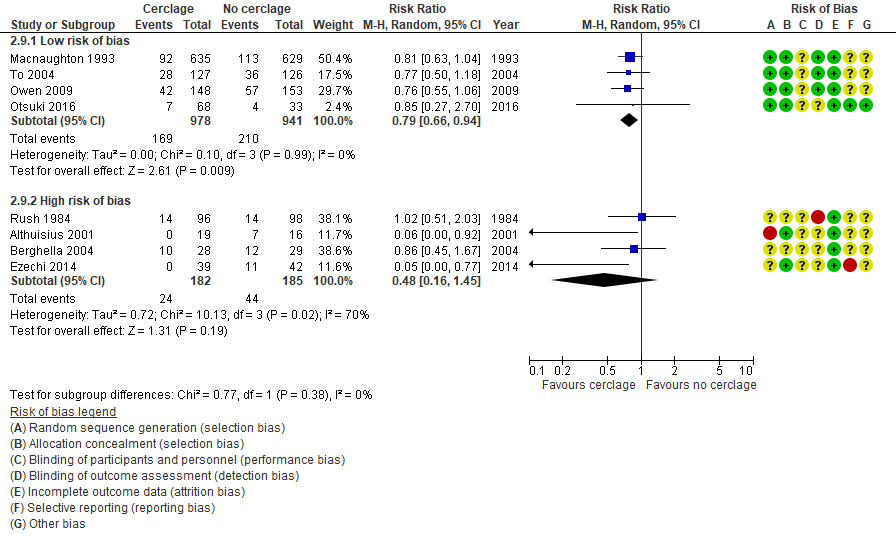


Conclusion: Cerclage compared with no cerclage probably reduces the risk of any preterm birth before 34 gestational weeks in women with a singleton pregnancy, not considering type of risk factor for preterm birth (GRADE ⊕⊕⊕🌕).

**Spontaneous preterm birth <34 weeks**

No trial reported spontaneous preterm birth <34 weeks.

**Any preterm birth <33 weeks** (Appendix 4.2, STable 4.2.4.a and SFigure 4)
A meta-analysis of two trials with low risk of bias, including 1517 women, showed a significant reduction in the rate of any preterm birth, RR 0.79 (95% CI 0.63 to 0.99). Sensitivity analysis excluding Macnaughton 1993 due to inclusion of miscarriages included in numerator and denominator: RR 0.84 (95% CI 0.54, 1.31). The crude event rate across trials was 18.3% without cerclage. The pooled weighted RD was -3.8 percentage points (95% CI -7.5 to -0.2).

**SFigure 4.** Outcome: Any preterm birth before 33 weeks in singletons.


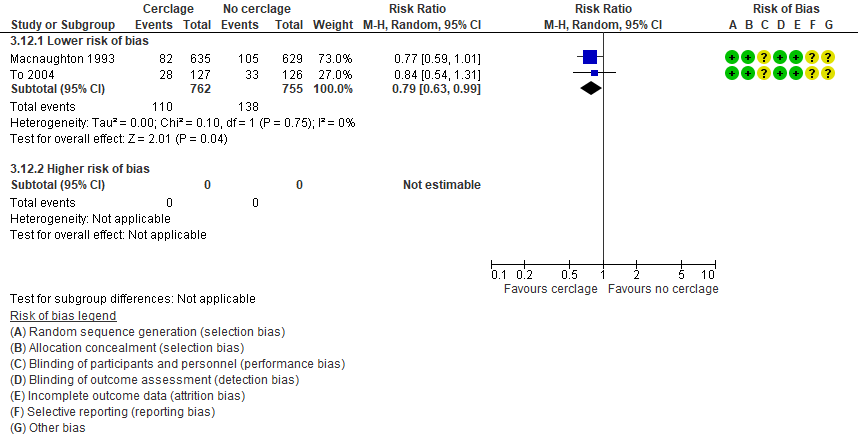


Conclusion: Cerclage compared with no cerclage probably reduces the risk of any preterm birth before 33 gestational weeks in women with a singleton pregnancy, not considering type of risk factor for preterm birth (GRADE ⊕⊕⊕🌕).

**Spontaneous preterm birth <33 weeks**

No trial reported spontaneous preterm birth <33 weeks.

**Any preterm birth <32 weeks** (Appendix 4.2, STable 4.2.5.a and SFigure 5)
One trial with low risk of bias, including 101 women with short cervical length, showed no difference in the rate of any preterm birth, RR 0.85 (95% CI 0.27 to 2.70). The event rate was 12.1% without cerclage. The RD was -1.8 percentage points (95% CI -15.1 to 11.5).

**SFigure 5**. Outcome: Any preterm birth before 32 weeks.


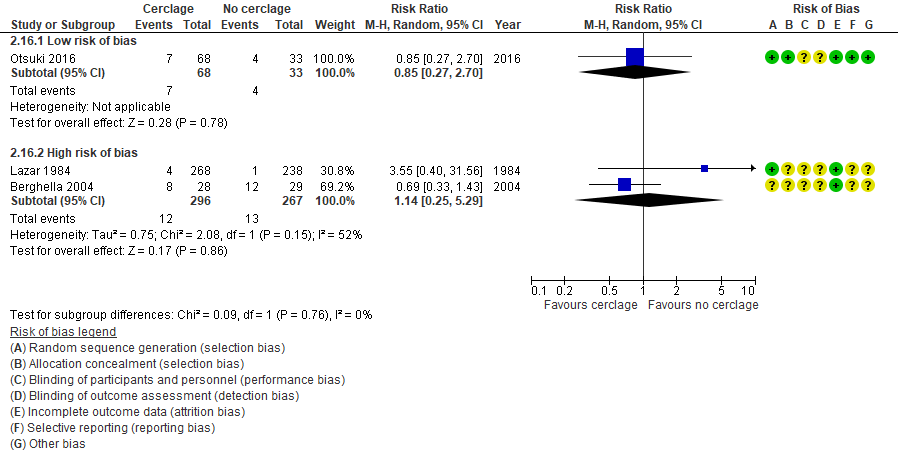


Conclusion: It is uncertain whether cerclage affects any preterm birth before 32 gestational weeks in women with a singleton pregnancy and short cervical length (GRADE ⊕🌕🌕🌕).

**Spontaneous preterm birth <32 weeks**

No trial reported spontaneous preterm birth <32 weeks.

**Any preterm birth <28 weeks** (Appendix 4.2, STable 4.2.6.a and SFigure 6)

A meta-analysis of four trials with low risk of bias, including 1915 women, showed no difference in the rate of any preterm birth, RR 0.77 (95% CI 0.60 to 1.00). Sensitivity analysis excluding Macnaughton 1993 due to inclusion of miscarriages in numerator and denominator: RR 0.73 (95% CI 0.49, 1.09). The crude event rate across trials was 12.2% without cerclage. The pooled weighted RD was -1.8 percentage points (95% CI -4.3 to 0.6).

**SFigure 6**. Outcome: Any preterm birth before 28 weeks in singleton pregnancies.


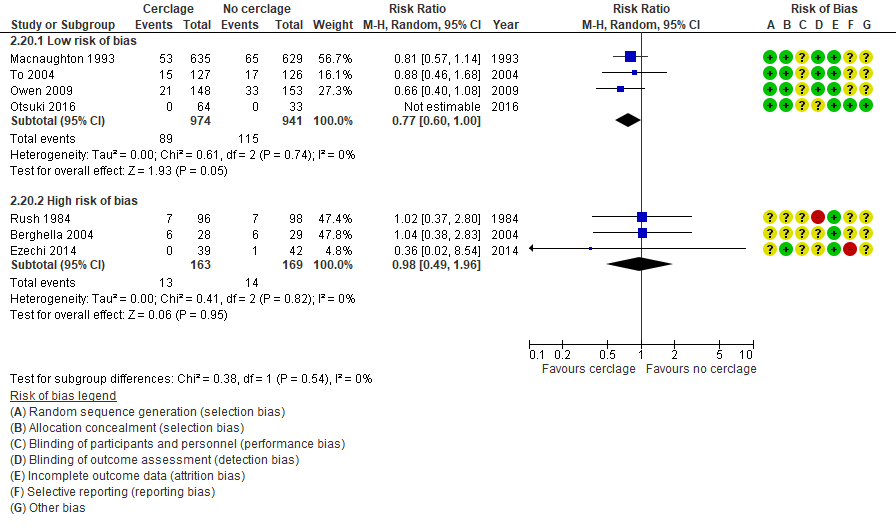


Conclusion: Cerclage compared with no cerclage probably results in no difference in the risk of any preterm birth before 28 gestational weeks not considering type of risk factor for preterm birth, although the CI for RR may imply a reduction (GRADE ⊕⊕⊕🌕).

**Spontaneous preterm birth <28 weeks**

No trial reported spontaneous preterm birth <28 weeks.

**Gestational age in singletons** (Appendix 4.2, STable 4.2.7 and SFigure 7)

One trial with low risk of bias, including 253 women with short cervical length, showed no mean difference in gestational age, 1.00 (-0.21 to 2.21) weeks, corresponding to seven days longer (one day less to 15 days longer) gestation in the cerclage group.

# SFigure 7. Outcome: Gestational age at delivery in singleton pregnancies.

**
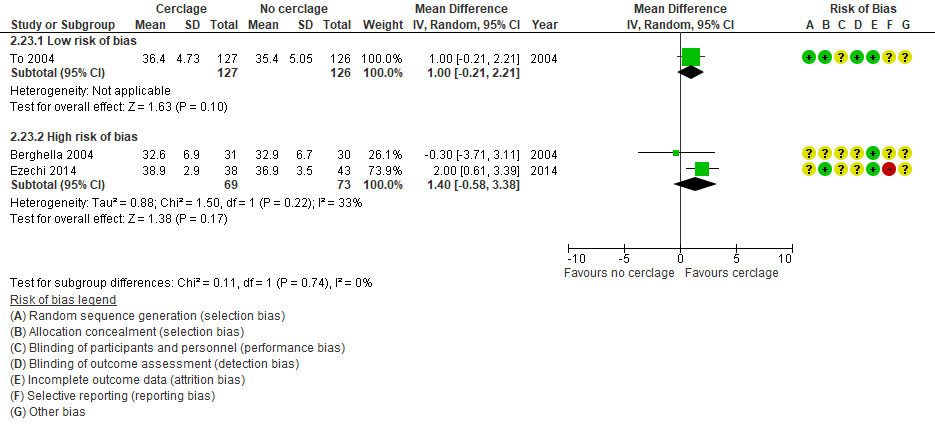
**

Conclusion: Cerclage compared with no cerclage may result in no difference in gestational age at delivery in women with a singleton pregnancy and short cervical length, although the CI may imply an increase (GRADE ⊕⊕🌕🌕).

**Low birth weight in singletons** (Appendix 4.2, STable 4.2.8 and SFigure 8)

One trial with low risk of bias, including 1320 neonates, showed no difference in the rate of low birth weight, RR 0.89 (95% CI 0.74 to 1.07). The event rate was 26.3% without cerclage. The RD was -2.7 percentage points (95% CI -7.6 to 1.7). However, the analysis included twins (2%) since data could not be separated between singletons and twins for this outcome.

**SFigure 8.** Outcome: Low birth weight (<2500g).


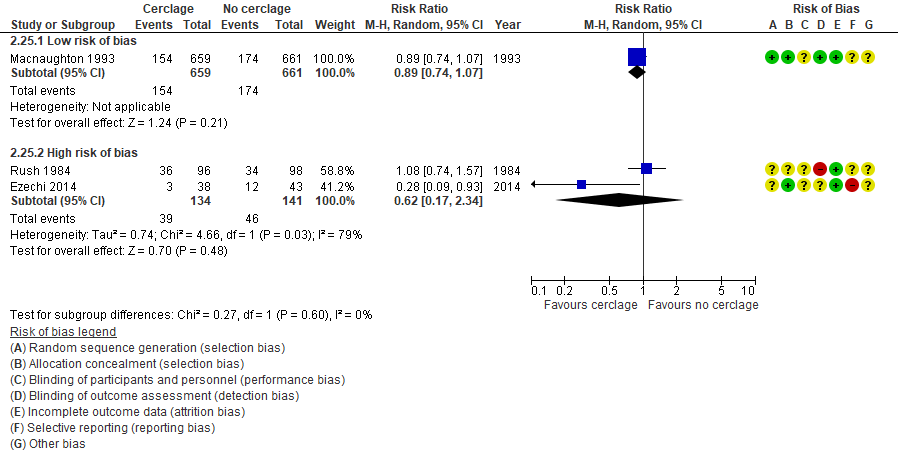


NB: Macnaughton 1993 includes 2% twins. Denominator includes miscarriages.

Conclusion: Cerclage compared with no cerclage may result in no difference in the risk of low birth weight in singletons not considering type of maternal risk factor for preterm birth (GRADE ⊕⊕🌕🌕).

**Very low birth weight in singletons** (Appendix 4.2, STable 4.2.9 and SFigure 9)
One trial with low risk of bias, including 1320 neonates, showed no difference in very low birth weight rate, RR 0.73 (95% CI 0.54 to 1.00). The event rate was 13.0% without cerclage. The RD was -3.5 percentage points (95% CI -6.9 to -0.04). However, the analysis included twins (2%) since data could not be separated between singletons and twins for this outcome.

# SFigure 9. Outcome: Very low birth weight (<1500g).


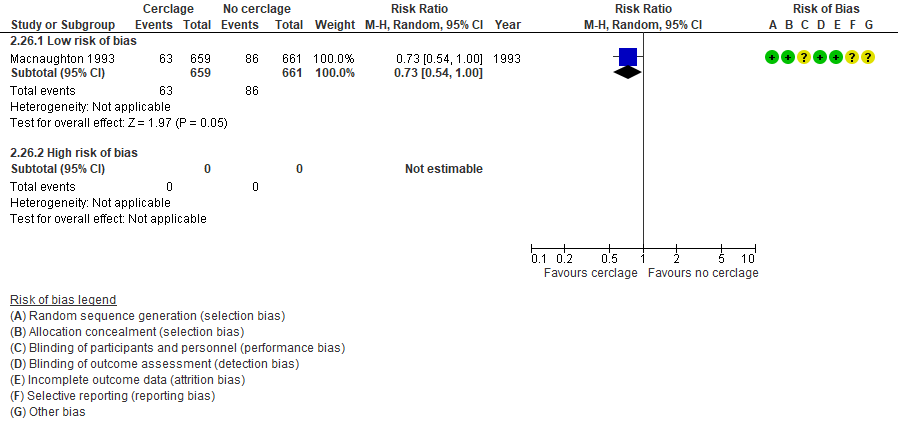


NB: Macnaughton 1993 includes 2% twins. Denominator includes miscarriages.

Conclusion: Cerclage compared with no cerclage may result in no difference in the risk of very low birth weight in singletons not considering the type of maternal risk factor for preterm birth, although the CI may imply a reduction (GRADE ⊕⊕🌕🌕).

**Mortality and morbidity in neonates from singleton pregnancies**

**Perinatal mortality** (Appendix 4.2, STable 4.2.10 and SFigure 10)

A meta-analysis of three trials with low risk of bias, including 1818 singletons, showed a significant reduction in perinatal mortality rate, 8.0% (73/910) versus 11.1% (101/908), RR 0.72 (95% CI 0.54 to 0.97). The pooled weighted RD was -2.9 percentage points (95% CI -5.6 to -0.2). One trial included miscarriages in both the numerator and denominator (Macnaughton et al., 1993). A sensitivity analysis excluding Macnaughton et al., 1993 lowered the pooled estimate further, but increased the width of the CI (RR 0.58 [95% CI 0.35 to 0.98]).

SFigure 10. Outcome: Perinatal mortality.


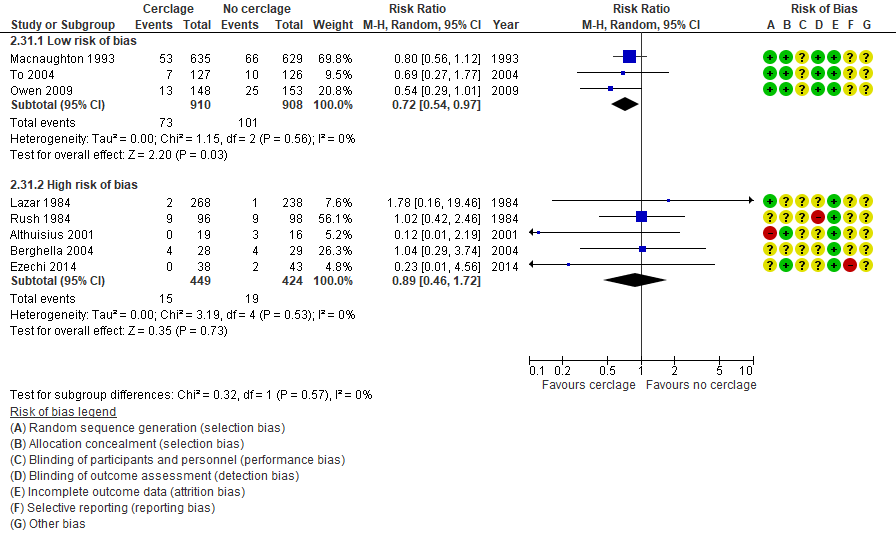


Conclusion: Cerclage compared with no cerclage may reduce the risk of perinatal mortality in singletons not considering the type of maternal risk factor for preterm birth (GRADE ⊕⊕🌕🌕).

**Neonatal mortality <7 days**

No study reported neonatal mortality <7 days.

**Neonatal mortality <28 days** (Appendix 4.2, STable 4.2.12 and SFigure 11)

A meta-analysis of three trials with low risk of bias, including 1674 neonates, showed no difference in neonatal mortality rate <28 days, RR 0.62 (95% CI 0.31 to 1.25). A sensitivity analysis excluding Macnaughton 1993 due to inclusion of miscarriages in denominator, numerator ‘liveborn died’: RR 0.73 (95% CI 0.23, 2.33). The crude event rate across trials was 2.4% without cerclage. The pooled weighted RD was -0.9 percentage points (95% CI -2.2 to 0.4).

**SFigure 11**. Outcome: Neonatal mortality <28 days.


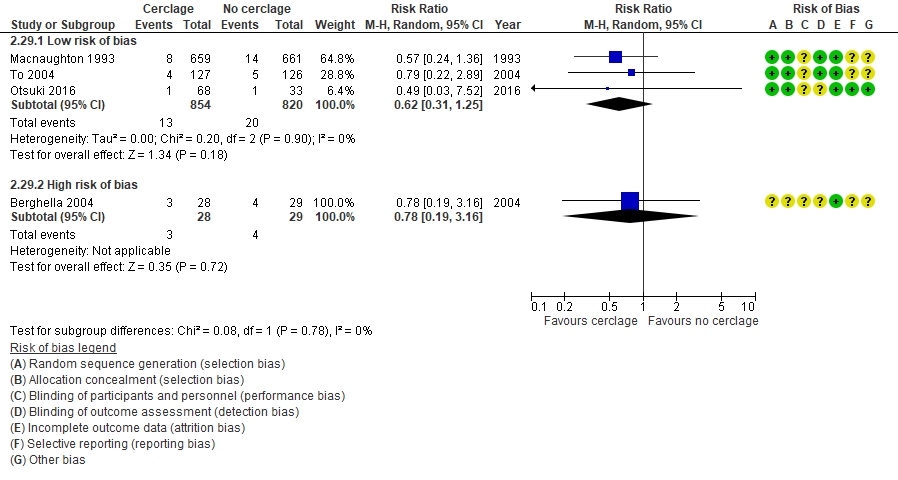


NB: Macnaughton 1993 includes 2% twins.

Conclusion: Cerclage compared with no cerclage may result in no difference in the risk of neonatal mortality <28 days in neonates not considering the type of maternal risk factor for preterm birth (GRADE ⊕⊕🌕🌕).

**Composite adverse neonatal outcome** (Appendix 4.2, STable 4.2.13 and SFigure 12)

A meta-analysis of two trials with low risk of bias, including 554 neonates, showed no difference in composite adverse neonatal outcome rate, RR 1.02 (95% CI 0.60 to 1.72). The crude event rate across trials was 9.0% without cerclage. The pooled weighted RD was 0.5 percentage points (95% CI -4.1 to 5.1). However, composite adverse neonatal outcome (data from the systematic review of Alfirevic et al., 2017) was not defined (and there were no definitions in the original trials) but named serious neonatal morbidity; therefore, mortality was probably not included.

**SFigure 12**. Outcome: Composite adverse neonatal outcome.


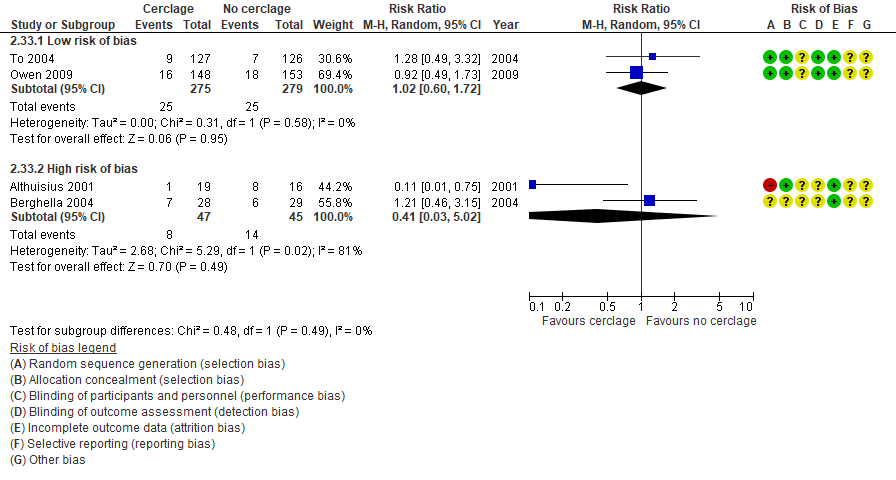


Conclusion: Cerclage compared with no cerclage may result in no difference in the risk of composite adverse neonatal outcomes in neonates, with the maternal risk factor short cervical length (GRADE ⊕⊕🌕🌕).

**Respiratory distress syndrome (RDS)** (Appendix 4.2, STable 4.2.14 and SFigure 13)

One trial with low risk of bias, including 300 neonates, showed no difference in RDS rate, RR 1.03 (95% CI 0.49 to 2.14). The event rate was 8.6% without cerclage. The RD was 0.2 percentage points (95% CI -6.1 to 6.6).

**SFigure 13**. Outcome: Respiratory distress syndrome.


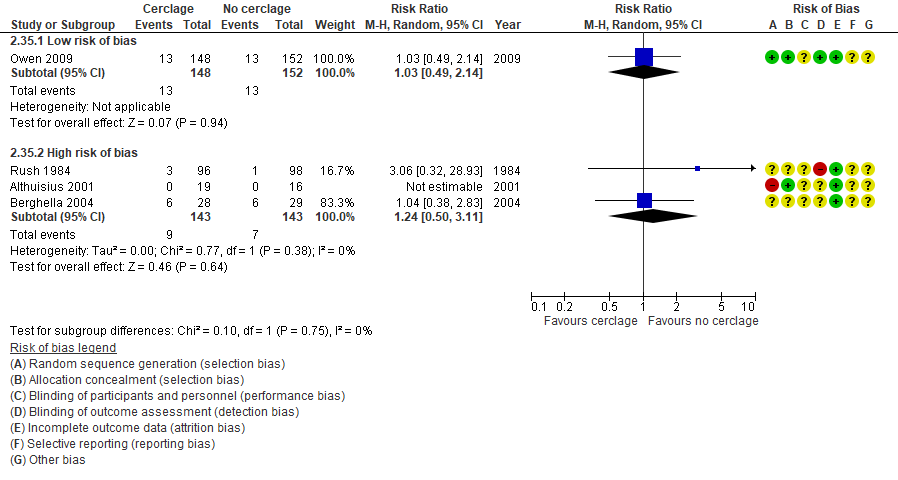


Conclusion: It is uncertain whether cerclage affects the risk of RDS in neonates with the maternal risk factors of previous spontaneous preterm birth and short cervical length (GRADE ⊕🌕🌕🌕).

**Bronchopulmonary dysplasia (BPD)** (Appendix 4.2, STable 4.2.15 and SFigure 14)

One trial with low risk of bias, including 244 neonates showed no difference in BPD rate, RR 0.98 (95% CI 0.25 to 3.84). The event rate was 3.3% without cerclage. The pooled weighted RD was -0.1 percentage points (95% CI -4.5 to 4.4).

**SFigure 14**. Outcome: Bronchopulmonary dysplasia.


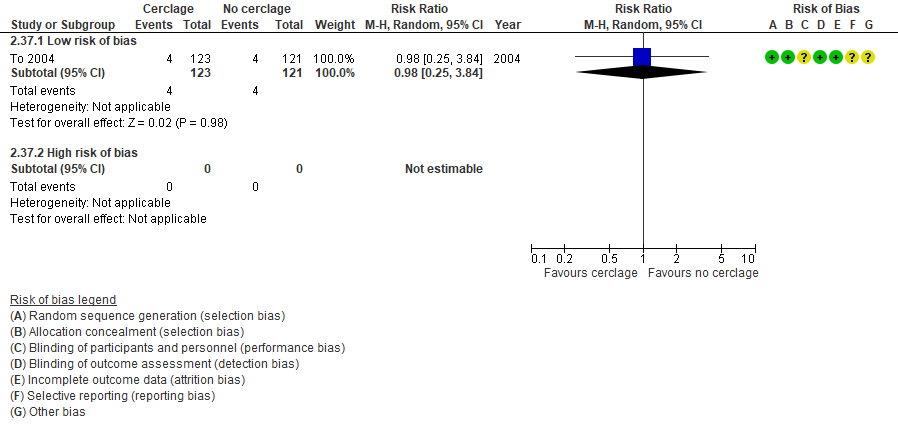


Conclusion: It is uncertain whether cerclage affects the risk of BPD in neonates with the maternal risk factor short cervical length (GRADE ⊕🌕🌕🌕).

**Intraventricular hemorrhage (IVH)** (Appendix 4.2, STable 4.2.16 and SFigure 15)

A meta-analysis of two trials with low risk of bias, including 544 neonates, showed no difference in the rate of IVH, RR 0.35 (95% CI 0.05 to 2.29). The crude event rate across trials was 1.5% without cerclage. The pooled weighted RD was -1.1 percentage points (95% CI -2.9 to 0.6).

# SFigure 15. Outcome: Intraventricular hemorrhage.


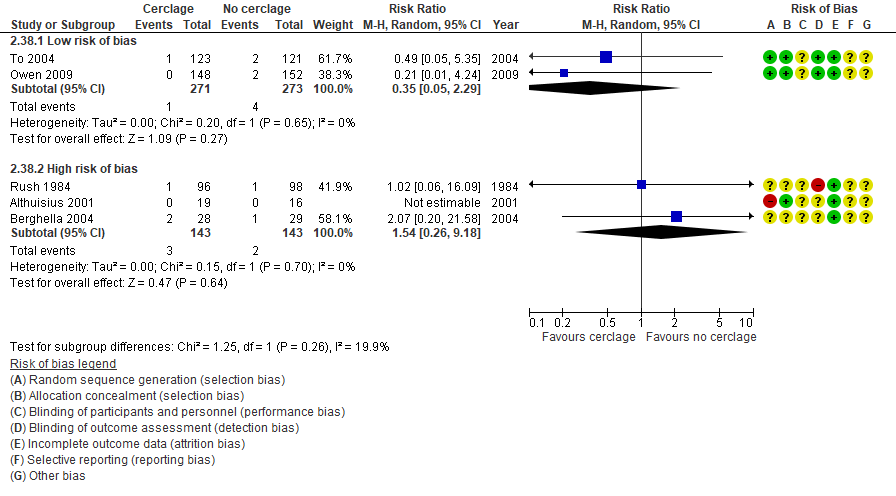


Conclusion: It is uncertain whether cerclage affects the risk of IVH in neonates, with the maternal risk factor short cervical length (GRADE ⊕🌕🌕🌕).

**Necrotizing enterocolitis (NEC)** (Appendix 4.2, STable 4.2.17 and SFigure 16)

One trial with low risk of bias, including 300 neonates, showed no difference in the rate of NEC, RR 1.03 (95% CI 0.15 to 7.20). The event rate was 1.3% without cerclage. The pooled weighted RD was 0.04 percentage points (95% CI -2.6 to 2.6).

**SFigure 16**. Outcome: Necrotizing enterocolitis.


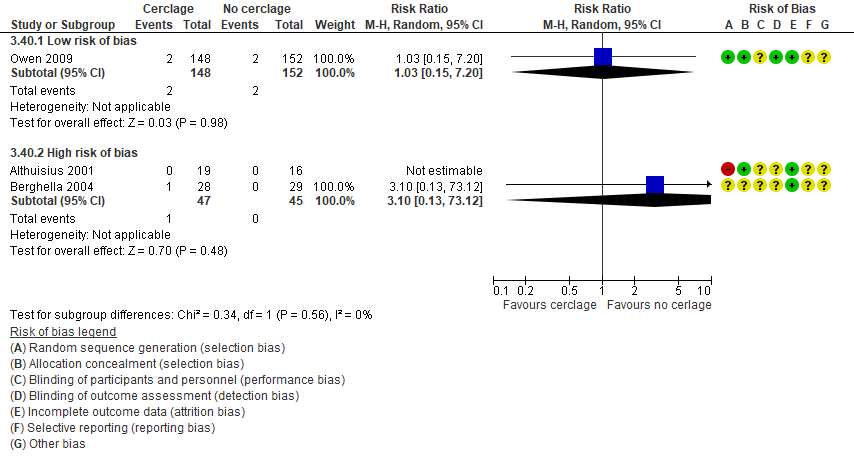


Conclusion: It is uncertain whether cerclage affects the risk of NEC in neonates with the maternal risk factors previous spontaneous preterm birth and short cervical length (GRADE ⊕🌕🌕🌕).

**Neonatal sepsis** (Appendix 4.2, STable 4.2.18 and SFigure 17)

One trial with low risk of bias, including 244 neonates, showed no difference in the rate of neonatal sepsis, RR 2.46 (95% CI 0.49 to 12.43). The event rate was 1.7% without cerclage. The RD was 2.4 percentage points (95% CI -1.8 to 6.6).

**SFigure 17**. Outcome: Neonatal sepsis.


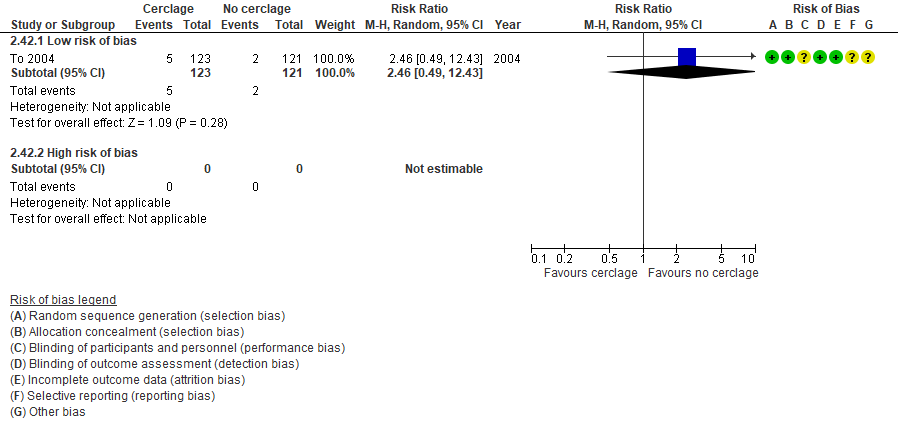


Conclusion: It is uncertain whether cerclage affects the risk of neonatal sepsis in neonates with the maternal risk factor short cervical length (GRADE ⊕🌕🌕🌕).

**Retinopathy of prematurity (ROP)** (Appendix 4.2, STable 4.2.19 and SFigure 18)

A meta-analysis of two trials with low risk of bias, including 544 neonates, showed no difference in the rate of ROP, RR 0.47 (95% CI 0.13 to 1.67). The crude event rate across trials was 2.9% without cerclage. The pooled weighted RD was -2.0 percentage points (95% CI -4.3 to 0.4).

**SFigure 18**. Outcome: Retinopathy of prematurity.


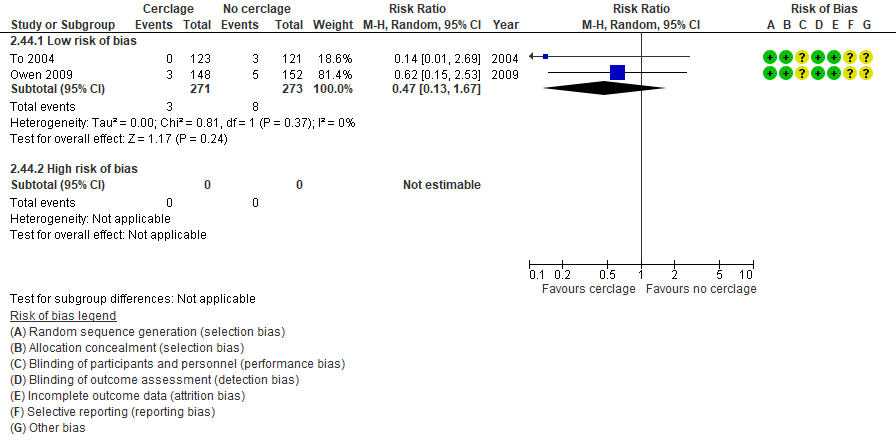


Conclusion: It is uncertain whether cerclage affects the risk of ROP in neonates, with the maternal risk factor short cervical length (GRADE ⊕🌕🌕🌕).

**Admittance to neonatal intensive care unit** (Appendix 4.2, STable 4.2.20 and SFigure 19)

No trial with low risk of bias reported on admittance to the neonatal intensive care unit.

**SFigure 19**. Outcome: Admittance to neonatal intensive care unit.

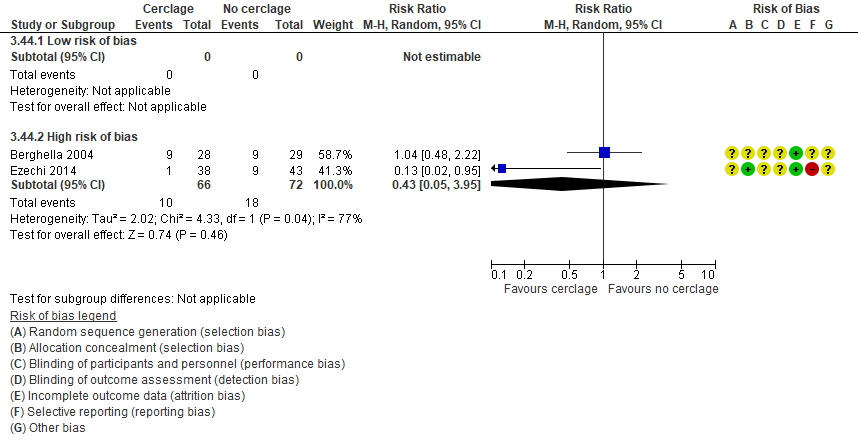


**Long-term child outcomes**

No trial reported long-term child outcomes.

**Mortality and morbidity in women with singleton pregnancies**

**Maternal mortality and morbidity**

No trial reported maternal mortality, hypertensive disorders in pregnancy, gestational diabetes mellitus, or intrahepatic cholestasis.

**Infection (maternal pyrexia) (**Appendix 4.2, STable 4.2.21 and SFigure 20)

Two trials with low risk of bias, reported fever antepartum (RR 4.96 [95% CI 0.59 to 41.86]) and postpartum (RR 2.01 [95% CI 0.99 to 4.07]) respectively. No pooled estimate was calculated due to the clinical heterogeneity.

# **SFigure 20**. Outcome: Maternal pyrexia.


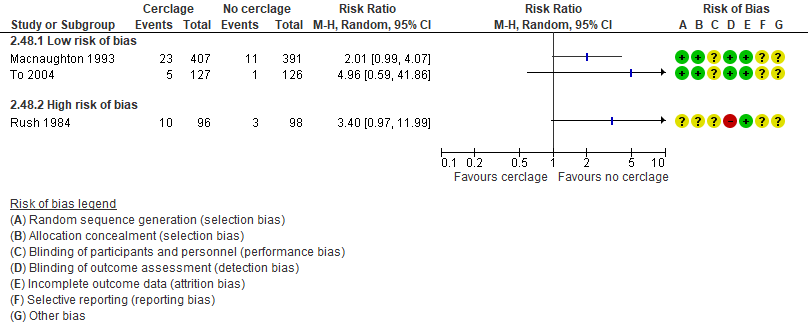
Macnaughton 1993 reports fever postpartum. To 2004 reports fever antepartum. Rush 1984 reports fever postpartum.

Conclusion: It is uncertain whether cerclage increases the risk of maternal pyrexia in women with a singleton pregnancy, not considering type of risk factor for preterm birth (GRADE ⊕🌕🌕🌕).

**Preterm prelabor rupture of the membranes (PPROM) (**Appendix 4.2, STable 4.2.22 and SFigure 21)

A meta-analysis of two trials with low risk of bias, including 1517 women, showed no difference in the rate of PPROM, RR 1.57 (95% CI 0.45 to 5.50). The crude event rate across trials was 2.5% without cerclage. The pooled weighted RD was 1.1 percentage points (95% CI -4.6 to 6.9).

**SFigure 21**. Outcome: Preterm prelabor rupture of membranes (PPROM).

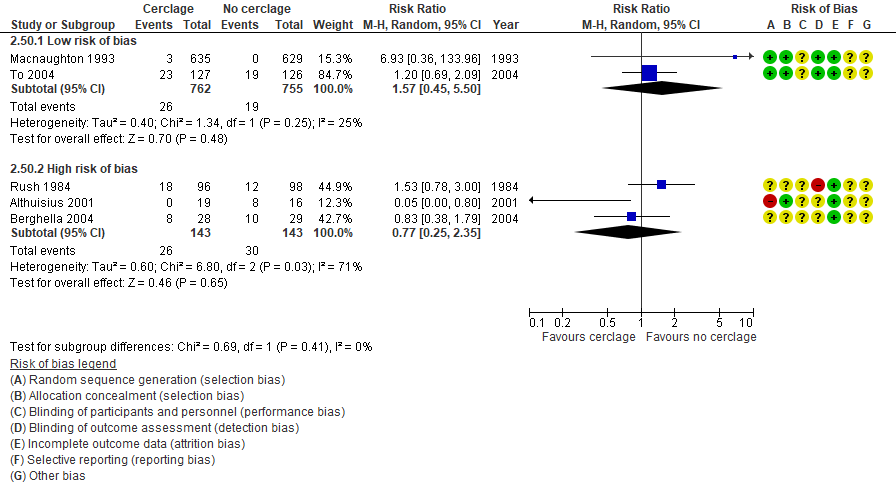


Conclusion: Cerclage compared with no cerclage may result in no difference in PPROM in women with a singleton pregnancy, not considering the type of risk factor for preterm birth (GRADE ⊕⊕🌕🌕).

# **Subgroup analyses**

Pre-specified subgroup analyses for the specific risk factor short cervical length were performed (SFigures 22-23). The meta-analyses of three trials with a total of 655 women, showed a significant reduction in the rate of any preterm birth <37 weeks, RR 0.72 (95% CI 0.61 to 0.86), and <34 weeks, RR 0.77 (95% CI 0.60 to 0.99).

# **SFigure 22**. Outcome: Any preterm birth <37 weeks among women with short cervical length.


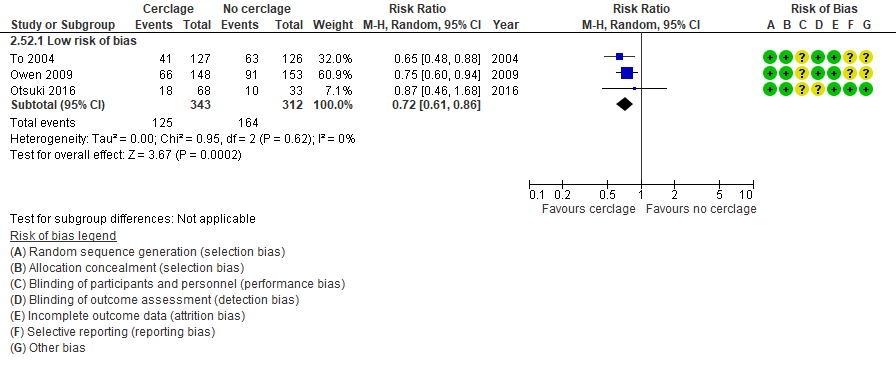


Cut-off cervical length: To 2004 ≤15 mm, Owen 2009 <25 mm, Otsuki 2016 <25 mm.

**SFigure 23**. Outcome: Any preterm birth <34 weeks among women with short cervical length.


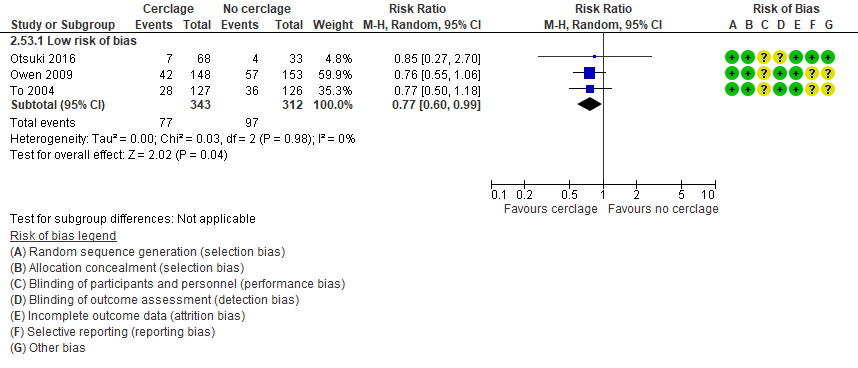


Cut-off cervical length: To 2004 ≤15 mm, Owen 2009 <25 mm, Otsuki 2016 <25 mm.

Only one trial had the specific inclusion criteria of previous spontaneous preterm birth (Owen et al., 2009) and no trial reported results in subgroups of patients with cervical surgical treatment for cervical intraepithelial neoplasia.
