# Supplementary material for: Progesterone, cerclage, pessary, or acetylsalicylic acid for prevention of preterm birth in singleton and multifetal pregnancies – A systematic review and meta-analyses
Source: Front Med (Lausanne). 2023 Feb 28;10:1111315. doi: 10.3389/fmed.2023.1111315 (PMC10015499; doi:10.3389/fmed.2023.1111315)
Supplement: Supplementary file 1 [file Data_Sheet_1.zip › Data Sheet 1_corrected/Appendix 5.3 Results Pessary_singletons.docx]

**Region Västra Götaland, HTA-centrum**

**Regional HTA]**

**Health Technology Assessment**

**HTA report 2022:129**

**Progesterone, cerclage, pessary, or acetylsalicylic acid for prevention of preterm birth in singleton and multifetal pregnancies**

**Appendix 5.3 Results pessary vs no pessary in singleton pregnancies**

Table of contents

[Abbreviations2](#_Abbreviations)

[Table S1 Risk of bias legend3](#_Table_S1._Risk)

[Results per outcome pessary vs no pessary in singleton pregnancies3](#_Results_per_outcome)

[Preterm birth SFigure 1-73-6](#_SFigure_1._Outcome:)

[Gestational age and birth weight SFigures 8-106-](#_SFigure_8._Outcome:)7

[Neonatal mortality and morbidity SFigures 11-207-1](#_SFigure_11._Outcome:)1

[Maternal morbidity SFigures 21-2411-13](#_SFigure_21._Outcome:)

# Abbreviations

BPD bronchopulmonary dysplasia

CI confidence interval

IVH intraventricular haemorrhage

NEC necrotizing enterocolitis

NICU neonatal intensive care unit

PPROM preterm prelabor rupture of membranes

RD risk difference

RDS respiratory distress syndrome

ROP retinopathy of prematurity

RR relative risk/risk ratio

# **Table S1.** **Risk of bias legend to** the colour plot within the following forests plots

1. Random sequence generation (selection bias)
2. Allocation concealment (selection bias)
3. Blinding of participants and personnel (performance bias)
4. Blinding of outcome assessment (detection bias)
5. Incomplete outcome data (attrition bias)
6. Selective reporting (reporting bias)
7. Conflict of interest bias

# Results per outcome

**Preterm birth in singletons** **across gestational weeks**

**Any preterm birth <37 weeks** (Appendix 4.3, STable 4.3.1.a and SFigure 1)

A meta-analysis of five trials, including 1531 women, showed no difference in the rate of any preterm birth, RR 0.87 (95% CI 0.73 to 1.03). The crude event rate across trials was 28.3% without pessary. The pooled weighted RD was -3.7% percentage points (95% CI -8.5 to 1.0).

# **SFigure 1**. Outcome: Any preterm birth <37 weeks.


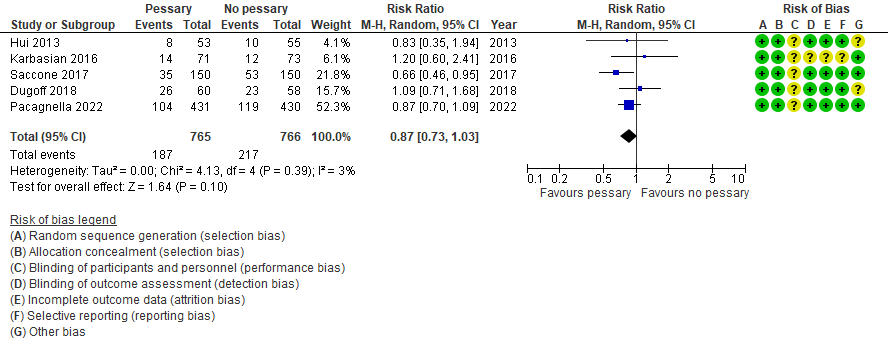


Conclusion: Pessary compared with no pessary probably results in no difference in the risk of any preterm birth before 37 gestational weeks, in women with a singleton pregnancy and short cervical length (GRADE ⊕⊕⊕🌕 ).

**Spontaneous preterm birth <37 weeks** (Appendix 4.3, STable 4.3.1.b and SFigure 2)

A meta-analysis of four trials, including 1694 women, showed no difference in the rate of spontaneous preterm birth, RR 0.67 (95% CI 0.41 to 1.09). The crude event rate across trials was 31.8% without pessary. The pooled weighted RD was -12.6% percentage points (95% CI -30.6 to 5.4).

**SFigure 2.** Outcome: Spontaneous preterm birth <37 weeks.


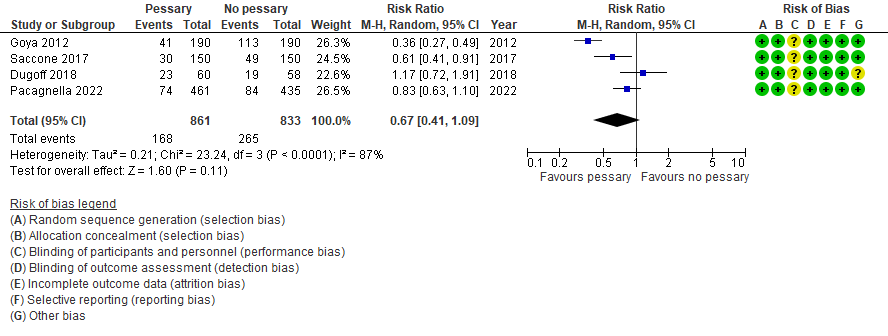


Conclusion: Pessary compared with no pessary may result in no difference in the risk of spontaneous preterm birth before 37 gestational weeks, in women with a singleton pregnancy and short cervical length (GRADE ⊕⊕🌕 🌕).

**Any preterm birth <35 weeks**

No trial reported spontaneous preterm birth <35 weeks.

**Spontaneous preterm birth <35 weeks**

No trial reported spontaneous preterm birth <35 weeks.

**Any preterm birth <34 weeks** (Appendix 4.3, STable 4.3.2.a and SFigure 3)

A meta-analysis of seven trials, including 2843 women, showed no difference in the rate of any preterm birth, RR 0.78 (95% CI 0.49 to 1.23). The crude event rate across trials was 14.8% without pessary. The pooled weighted RD was -3.5 % percentage points (95% CI -9.7 to 2.8).

**SFigure 3.** Outcome: Any preterm birth <34 weeks.


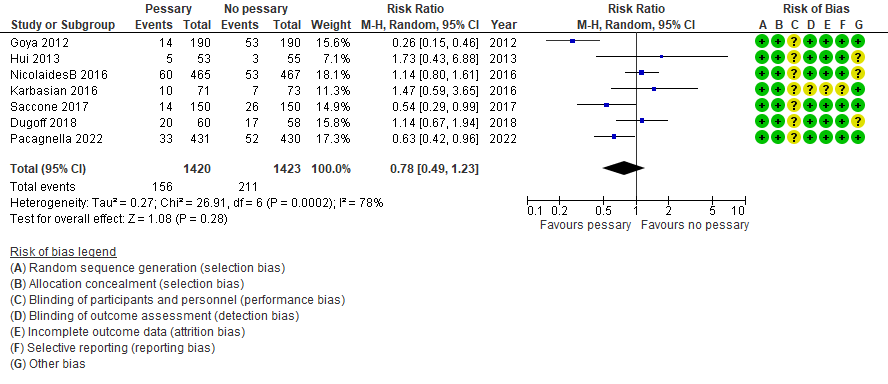


Conclusion: Pessary compared with no pessary may result in no difference in the risk of any preterm birth before 34 gestational weeks, in women with a singleton pregnancy and short cervical length (GRADE ⊕⊕🌕 🌕).

**Spontaneous preterm birth <34 weeks** (Appendix 4.3, STable 4.3.2.b and SFigure 4)

A meta-analysis of six trials, including 2726 women, showed no difference in the rate of spontaneous preterm birth, RR 0.71 (95% CI 0.41 to 1.21). The crude event rate across trials was 13.5% without pessary. The pooled weighted RD was -4.3 % percentage points (95% CI (-10.8 to 2.3).

**SFigure 4.** Outcome: Spontaneous preterm birth <34 weeks.


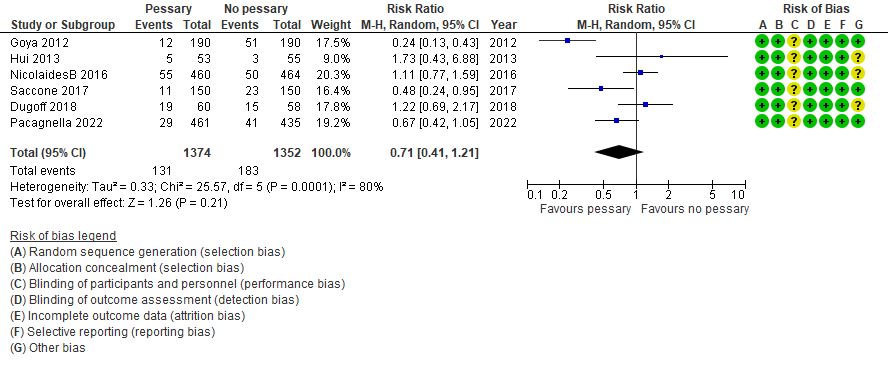


Conclusion: Pessary compared with no pessary may result in no difference in spontaneous preterm birth before 34 gestational weeks, in women with a singleton pregnancy and short cervical length (GRADE ⊕⊕🌕 🌕).

**Any preterm birth <33 weeks**

No trial reported any preterm birth <33 weeks.

**Spontaneous preterm birth <33 weeks**

No trial reported on spontaneous preterm birth <33 weeks.

**Any preterm birth <32 weeks** (Appendix 4.3, STable 4.3.3.a and SFigure 5)

A meta-analysis of four trials, including 2239 women, showed no difference in the rate of any preterm birth, RR 0.87 (95% CI 0.56 to 1.34). The crude event rate across trials was 8.2% without pessary. The pooled weighted RD was -1.0 % percentage points (95% CI -4.3 to 2.3).

**SFigure 5.** Outcome: Any preterm birth <32 weeks.


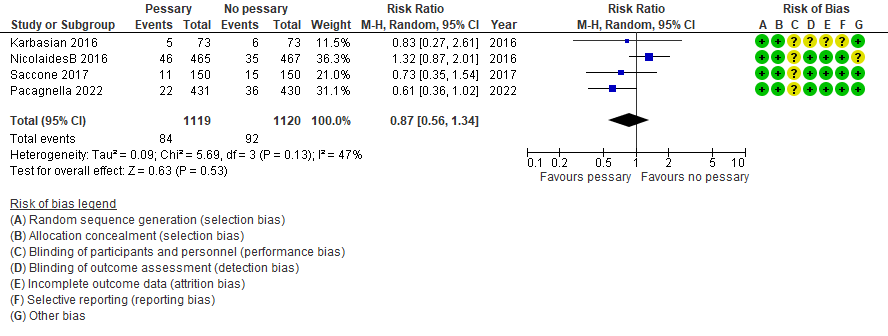


Conclusion: Pessary compared with no pessary may result in no difference in the risk of any preterm birth before 32 gestational weeks, in women with a singleton pregnancy and short cervical length (GRADE ⊕⊕🌕 🌕).

**Spontaneous preterm birth <32 weeks** (Appendix 4.3, STable 4.3.3.b)

One trial, including 300 women, showed no difference in the rate of spontaneous preterm birth; RR 0.71 (95% CI 0.33 to 1.56). The crude event rate was 9.3% without pessary. The RD was -2.7 % percentage points (95% CI -8.8 to 3.5).

Conclusion: Pessary compared with no pessary may result in no difference in the risk of spontaneous preterm birth before 32 gestational weeks, in women with a singleton pregnancy and short cervical length (GRADE ⊕⊕🌕 🌕).

**Any preterm birth <28 weeks** (Appendix 4.3, STable 4.3.4.a and SFigure 6)

A meta-analysis of five trials, including 2319 women, showed no difference in the rate of any preterm birth, RR 0.86 (95% CI 0.52 to 1.42). The crude event rate across trials was 5.1% without pessary. The pooled weighted RD was -0.6 % percentage points (95% CI -3.1 to 1.9).

**SFigure 6.** Outcome: Any preterm birth <28 weeks.


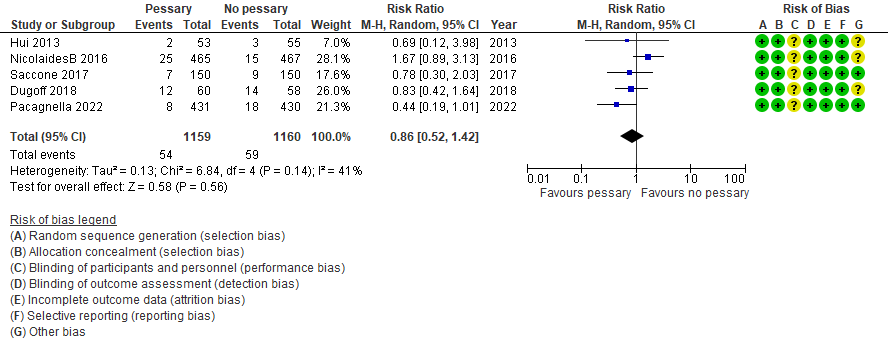


Conclusion: Pessary compared with no pessary may result in no difference in the risk of any preterm birth before 28 gestational weeks, in women with a singleton pregnancy and short cervical length

(GRADE ⊕⊕🌕 🌕).

**Spontaneous preterm birth <28 weeks** (Appendix 4.3, STable 4.3.4.b and SFigure 7)

A meta-analysis of four trials, including 1694 women, showed a significant difference in the rate of spontaneous preterm birth, RR 0.45 (95% CI 0.22 to 0.93). The crude event rate across trials was 6.6% without pessary. The pooled weighted RD was -3.6 % percentage points (95% CI -5.3 to -1.8).

**SFigure 7.** Outcome: Spontaneous preterm birth <28 weeks.


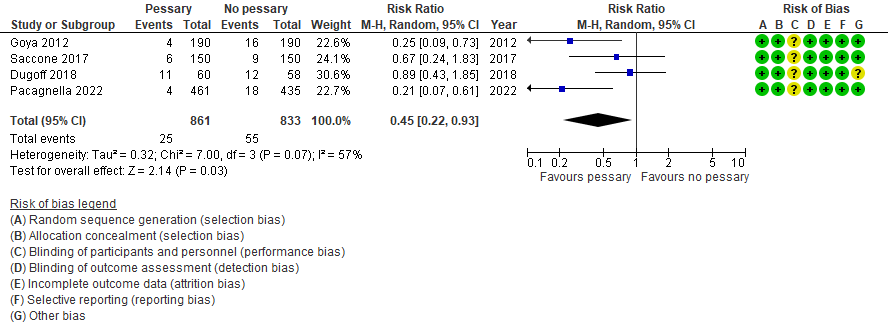


Conclusion: Pessary compared with no pessary may reduce the risk of spontaneous preterm birth before 28 gestational weeks, in women with a singleton pregnancy and short cervical length (GRADE ⊕⊕🌕 🌕).

**Gestational age and birth weight in singletons**

**Gestational age** (Appendix 4.3, STable 4.3.5 and SFigure 8)

A meta-analysis of seven trials, including 2932 women showed no mean difference in gestational age, 0.78 (-0.08 to 1.64) weeks, corresponding to approximately 5.5 days longer (0.5 day less to 11.5 days longer) gestational length in the pessary group.

# SFigure 8. Outcome: Gestational age at delivery (weeks).


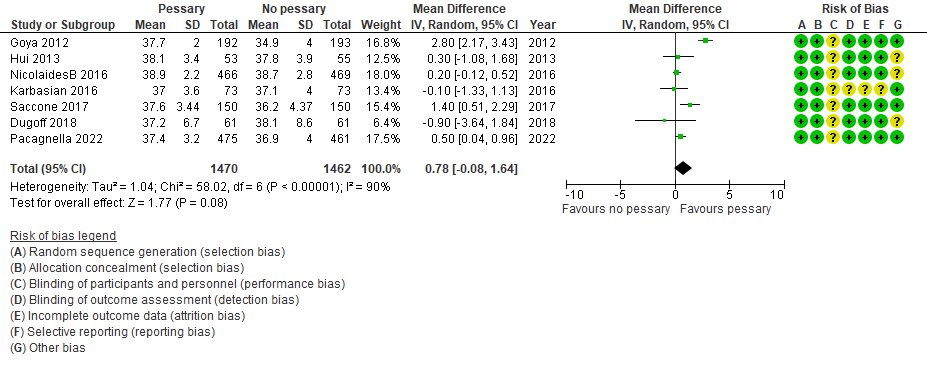


Conclusion: Pessary compared with no pessary probably results in no difference in gestational age at delivery, in women with a singleton pregnancy and short cervical length (GRADE⊕⊕⊕🌕 ).

**Low birth weight in singletons (**Appendix 4.3, STable 4.3.6 and SFigure 9)

A meta-analysis of four trials, including 1756 women with a singleton pregnancy, showed no difference in the rate of low birth weight, RR 0.73 (95% CI 0.39 to 1.35). The crude event rate across trials was 25.6% without pessary. The pooled weighted RD was -6.2% percentage points (95% CI -18.8 to 6.4).

**SFigure 9.** Outcome: Low birth weight (<2500 g).


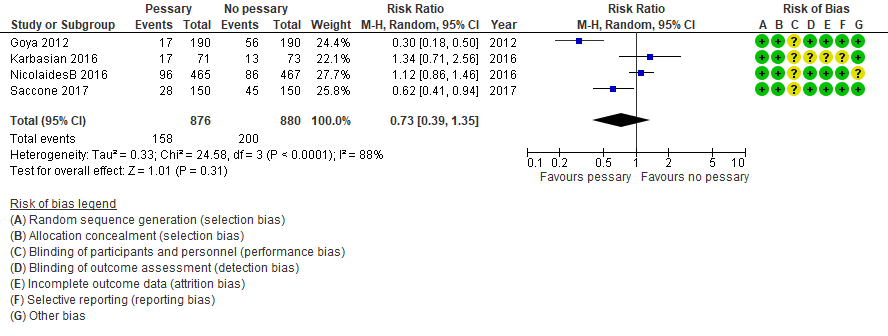


Conclusion: Pessary compared with no pessary may result in no difference in the risk of low birth weight (<2500 g), in singletons from women with short cervical length (GRADE ⊕⊕🌕 🌕).

**Very low birth weight in singletons (**Appendix 4.3, STable 4.3.7 and SFigure 10)

A meta-analysis of three trials, including 1612 women with a singleton pregnancy, showed no difference in the rate of low birth weight, RR 0.71 (95% CI 0.30 to 1.68). The crude event rate across trials was 8.6% without pessary. The pooled weighted RD was -3.0 % percentage points (95% CI -10.1 to 4.1).

**SFigure 10.** Outcome: Very low birth weight (<1500 g).

**
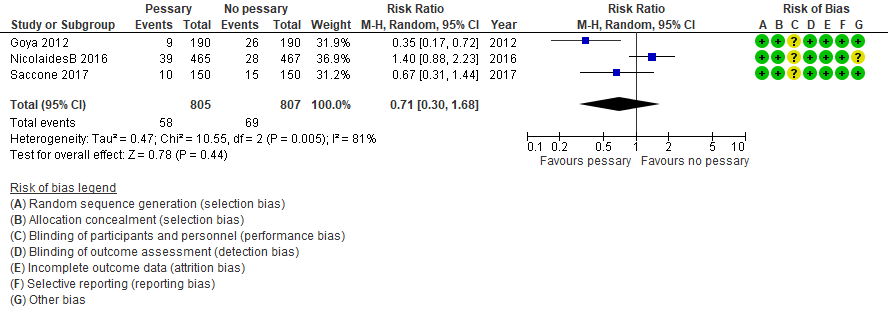
**

Conclusion: Pessary compared with no pessary may result in no difference in the risk of very low birth weight (<1500 g), in singletons from women with short cervical length (GRADE ⊕⊕🌕 🌕).

**Mortality and morbidity in neonates from singleton pregnancies**

**Perinatal mortality (**Appendix 4.3, STable 4.3.8 and SFigure 11)

A meta-analysis of four trials, including 2353 neonates showed no difference in the rate of perinatal mortality, RR 0.73 (95% CI 0.36 to 1.46). The crude event rate across trials was 4.1% without pessary. The pooled weighted RD was -1.1 % percentage points (95% CI -3.5 to 1.3).

# SFigure 11. Outcome: Perinatal mortality.


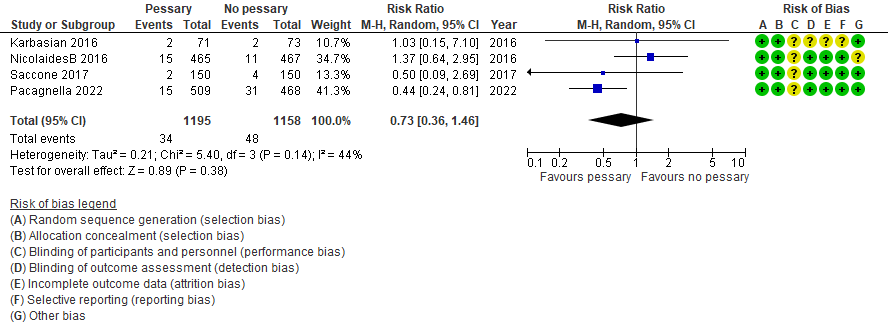


Conclusion: Pessary compared with no pessary may result in no difference in perinatal mortality in singletons from women with short cervical length (GRADE ⊕⊕🌕 🌕).

**Neonatal mortality < 7 days**

No trial reported neonatal mortality <7 days.

**Neonatal mortality < 28 days (**Appendix 4.3, STable 4.3.9 and SFigure 12)

A meta-analysis of seven trials, including 2931 women with a singleton pregnancy, showed no difference in the rate of neonatal mortality <28 days, RR 0.66 (95% CI 0.39 to 1.10). The crude event rate across trials was 2.4% without pessary. The pooled weighted RD was -0.5 % percentage points (95% CI -1.4 to 0.5).

**SFigure 12.** Outcome: Neonatal mortality <28 days.


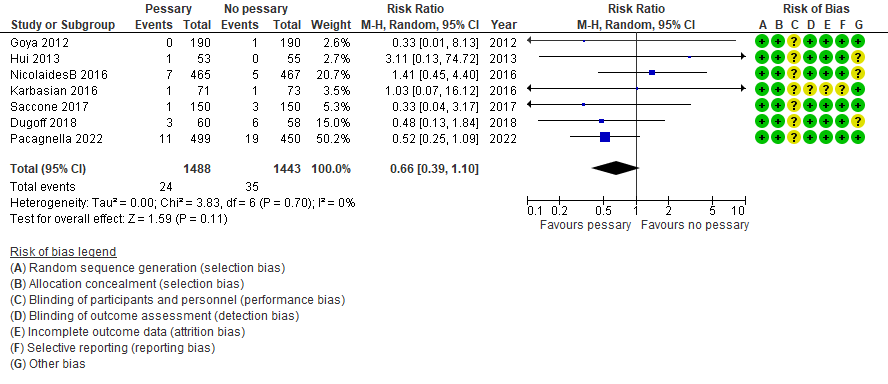


Conclusion: Pessary compared with no pessary may result in no difference in neonatal mortality <28 days, in singletons from women with short cervical length (GRADE ⊕⊕🌕 🌕).

**Composite adverse neonatal outcome (**Appendix 4.3, STable 4.3.10 and SFigure 13)

A meta-analysis of five trials, including 2668 neonates showed no difference in the rate of composite neonatal morbidity, RR 0.67 (95% CI 0.40 to 1.13). The crude event rate across trials was 16.3% without pessary. The pooled weighted RD was -5.9 % percentage points
(95% CI -13.2 to 1.4).

A sensitivity analysis of two trials with low risk of bias, including 1286 neonates showed no difference in the rate of composite neonatal morbidity when excluding trials including neonatal mortality, RR of 0.46 (95% CI 0.07 to 3.21).

The composite adverse neonatal outcome included any of intrauterine fetal death, neonatal death, intraventricular haemorrhage, periventricular leukomalacia, necrotizing enterocolitis, bronchopulmonary dysplasia, respiratory distress syndrome, retinopathy of prematurity, or confirmed sepsis.

**SFigure 13.** Outcome: Composite adverse neonatal outcome with or without mortality.


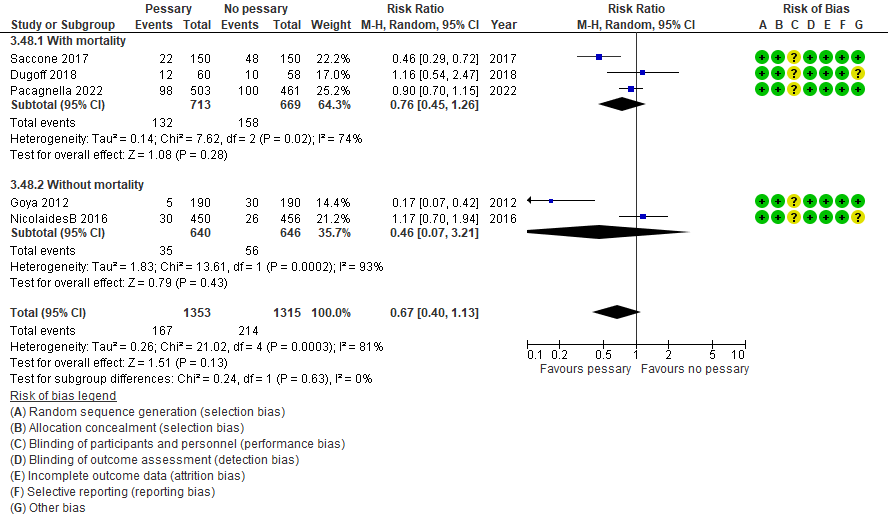


Conclusion: Pessary, compared with no pessary may result in no difference in a composite adverse neonatal outcome in singletons from women with short cervical length (GRADE ⊕⊕🌕 🌕).

**Respiratory distress syndrome (RDS) (**Appendix 4.3, STable 4.3.11 and SFigure 14)

A meta-analysis of six trials, including 2761 neonates showed no difference in the rate of RDS, RR of 0.77 (95% CI 0.48 to 1.23). The crude event rate across trials was 13.0% without pessary. The pooled weighted RD was -2.7 % percentage points (95% CI -7.7 to 2.2).

**SFigure 14.** Outcome: Respiratory distress syndrome.

**
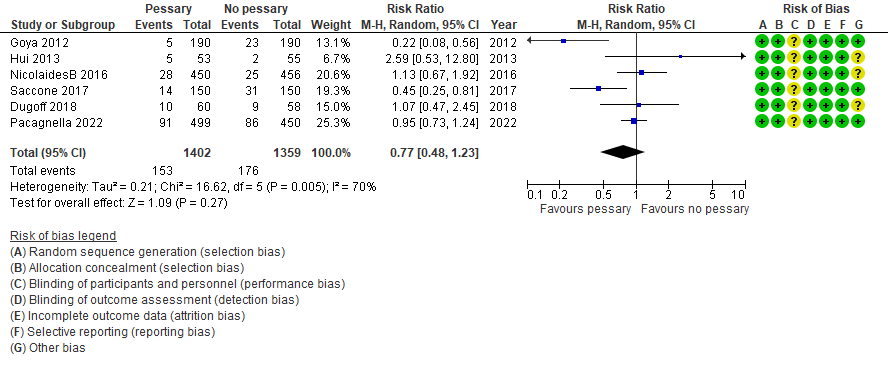
**

Conclusion: Pessary, compared with no pessary may result in no difference in RDS in singletons from women with short cervical length (GRADE ⊕⊕🌕 🌕).

**Bronchopulmonary dysplasia (BPD) (**Appendix 4.3, STable 4.3.12 and SFigure 15)

A meta-analysis of three trials, including 1365 neonates showed no difference in the rate of BPD, RR of 0.75 (95% CI 0.43 to 1.30). The crude event rate across trials was 4.1% without pessary. The pooled weighted RD was -0.8 % percentage points (95% CI -2.4 to 0.9).

**SFigure 15.** Outcome: Bronchopulmonary dysplasia.

**
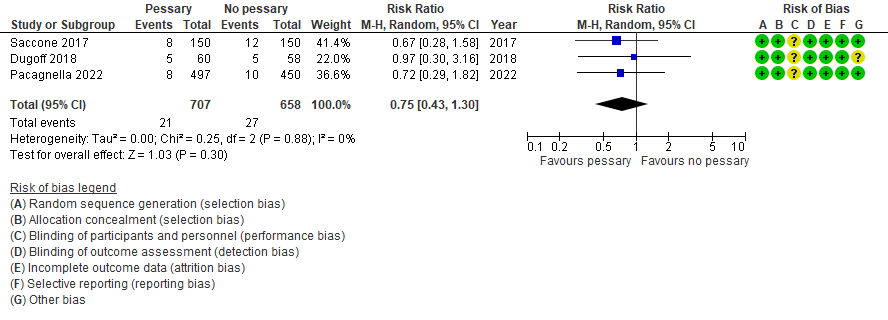
**

Conclusion: Pessary, compared with no pessary may result in no difference in BPD in singletons from women short cervical length (GRADE ⊕⊕🌕 🌕).

**Intraventricular hemorrhage (IVH) (**Appendix 4.3, STable 4.3.13 and SFigure 16)

A meta-analysis of five trials, including 1812 women showed no difference in the rate of IVH, RR 1.17 (95% CI 0.48 to 2.81). The crude event rate across trials was 1.5% without pessary. The pooled weighted RD was -0.0 % percentage points (95% CI -1.6 to 1.5).

**SFigure 16.** Outcome: Intraventricular haemorrhage.

**
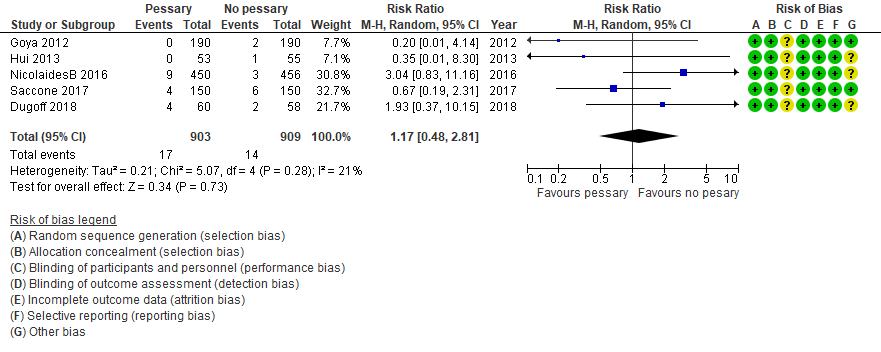
**

Conclusion: Pessary, compared with no pessary may result in no difference in IVH in singletons from women short cervical length (GRADE ⊕⊕🌕 🌕).

**Necrotizing enterocolitis (NEC) (**Appendix 4.3, STable 4.3.14 and SFigure 17)

A meta-analysis of five trials, including 2651 neonates showed no difference in the rate of NEC, RR 1.00 (95% CI 0.47 to 2.15). The crude event rate across trials was 1.1% without pessary. The pooled weighted RD was -0.1 % percentage points (95% CI -0.8 to 0.6).

**SFigure 17.** Outcome: Necrotizing enterocolitis.

**
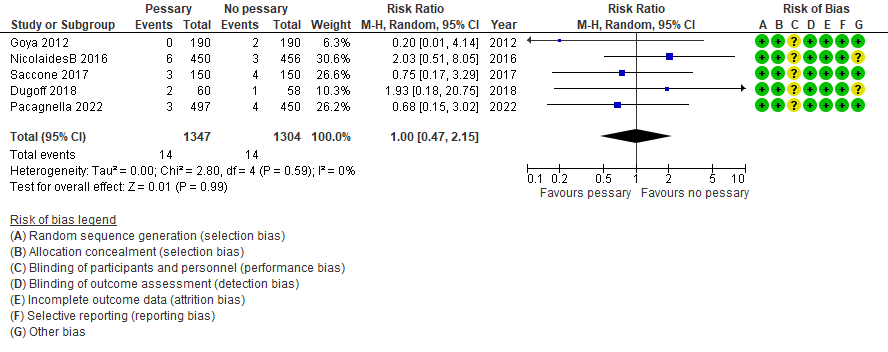
**

Conclusion: Pessary, compared with no pessary may result in no difference in NEC in singletons from women with short cervical length (GRADE ⊕⊕🌕 🌕).

**Neonatal sepsis (**Appendix 4.3, STable 4.3.15 and SFigure 18)

A meta-analysis of six trials, including 2759 neonates showed no difference in the rate of neonatal sepsis, RR 0.89 (95% CI 0.55 to 1.43). The crude event rate across trials was 4.6% without pessary. The pooled weighted RD was -0.7 % percentage points (95% CI -2.9 to 1.5).

**SFigure 18.** Outcome: Neonatal sepsis.

**
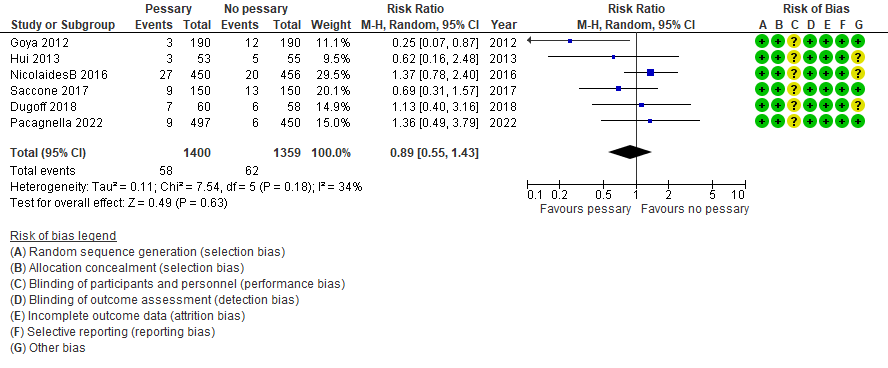
**

Conclusion: Pessary, compared with no pessary may result in no difference in neonatal sepsis in singletons from women with short cervical length (GRADE ⊕⊕🌕 🌕).

**Retinopathy of prematurity (ROP) (**Appendix 4.3, STable 4.3.16 and SFigure 19)

A meta-analysis of four trials, including 1704 neonates showed no difference in the rate of ROP, 0.51 (95% CI 0.10 to 2.60). The crude event rate across trials was 1.9% without pessary. The pooled weighted RD was -1.6 % percentage points (95% CI -4.5 to 1.4).

**SFigure 19.** Outcome: Retinopathy of prematurity.

**
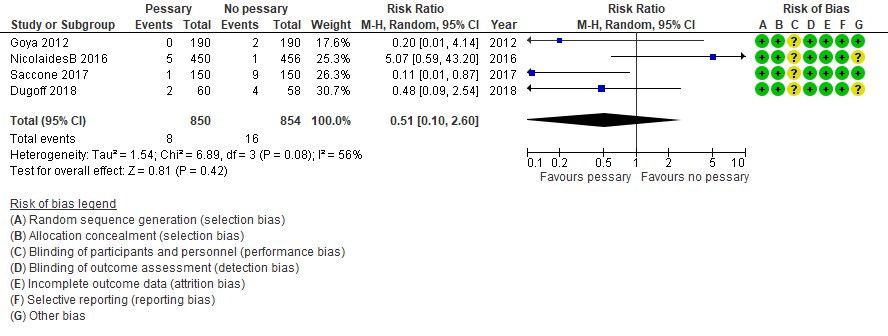
**

Conclusion: Pessary, compared with no pessary may result in no difference in ROP in singletons from women with short cervical length (GRADE ⊕⊕🌕 🌕).

**Admittance to neonatal intensive care unit (**Appendix 4.3, STable 4.3.17 and SFigure 20)

A meta-analysis of five trials, including 2428 neonates showed no difference in the rate of NICU admission, RR 1.04 (95% CI 0.78 to 1.38). The crude event rate across trials was 14.2% without pessary. The pooled weighted RD was 0.4 % percentage points (95% CI -3.4 to 4.2).

**SFigure 20.** Outcome: Admittance to neonatal intensive care unit (NICU).

**
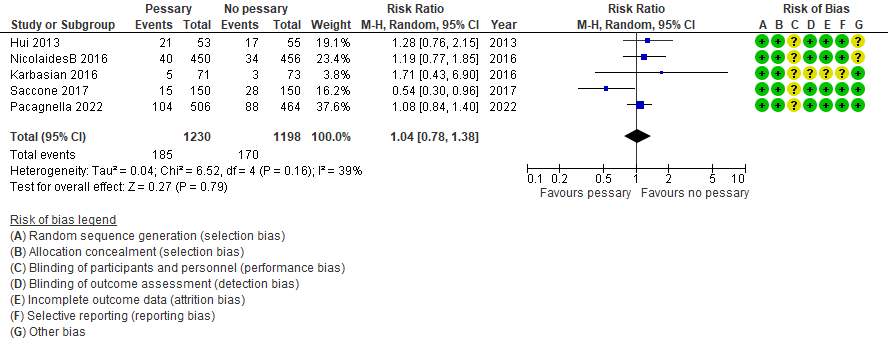
**

Conclusion: Pessary, compared with no pessary may result in no difference in admittance to NICU for singletons from women with short cervical length (GRADE ⊕⊕🌕 🌕).

**Long-term child outcomes in singletons**

No trial reported on long term child outcome in singleton pregnancies.

**Mortality and morbidity in women with singleton pregnancies**

**Maternal mortality <28d**

No trial reported maternal mortality <28 days.

**Hypertensive disorders in pregnancy**

No trial reported hypertensive disorders in pregnancy.

**Gestational diabetes mellitus**

No trial reported gestational diabetes mellitus.

**Cholestasis of pregnancy**

No trial reported cholestasis of pregnancy.

**Chorioamnionitis** (Appendix 4.3, STable 4.3.21 and SFigure 21)

A meta-analysis of four trials, including 942 women showed no difference in the rate of chorioamnionitis, RR 1.04 (95% CI 0.54 to 2.00). The crude event rate across trials was 3.6% without pessary. The pooled weighted RD was 0.1 % percentage points (95% CI -2.0 to 2.3).

# SFigure 21. Outcome: Chorioamnionitis.

**
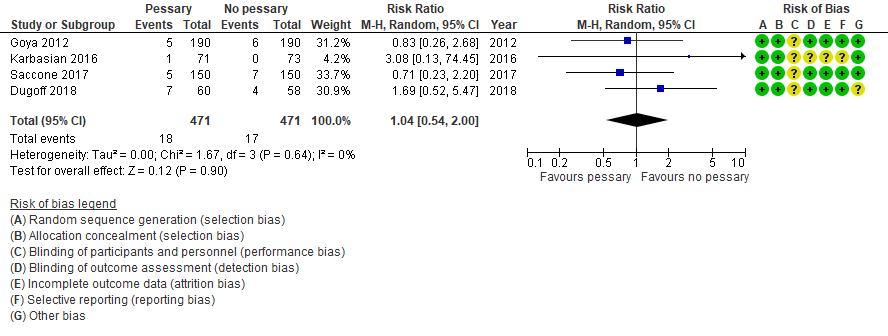
**

Conclusion: Pessary, compared with no pessary may result in no difference in chorioamnionitis in women with a singleton pregnancy and short cervical length (GRADE ⊕⊕🌕 🌕).

**Genitourinary infections (**Appendix 4.3, STable 4.3.22 and SFigure 22)

A meta-analysis of three trials, including 1165 women showed no difference in the rate of genitourinary infections, RR 0.94 (95% CI 0.70 to 1.26). The crude event rate across trials was 13.3% without pessary. The pooled weighted RD was -1.1 % percentage points (95% CI -4.8 to 2.6).

**SFigure 22.** Outcome: Genitourinary infection.

**
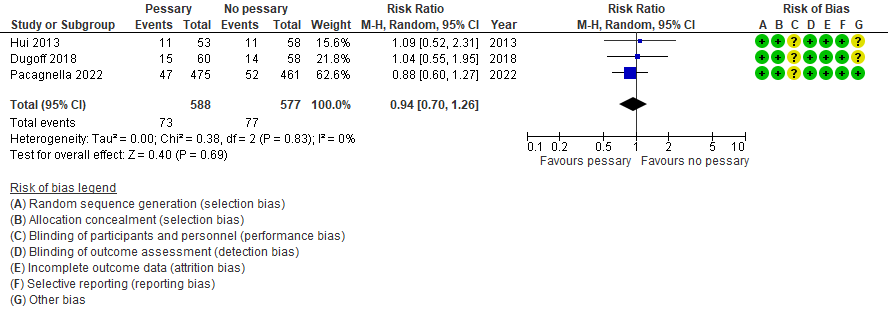
**

Conclusion: Pessary, compared with no pessary may result in no difference in genitourinary infections in women with a singleton pregnancy and short cervical length (GRADE ⊕⊕🌕 🌕).

**Vaginal discharge (**Appendix 4.3, STable 4.3.23 and SFigure 23)

A meta-analysis of three trials, including 798 women showed an increase in the frequency of vaginal discharge, RR 1.91 (95% CI 1.60 to 2.28). The crude event rate across trials was 46.2% without pessary. The pooled weighted RD was 41.5 % percentage points (95% CI 26.3 to 56.8).

**SFigure 23.** Outcome: Vaginal discharge.

**
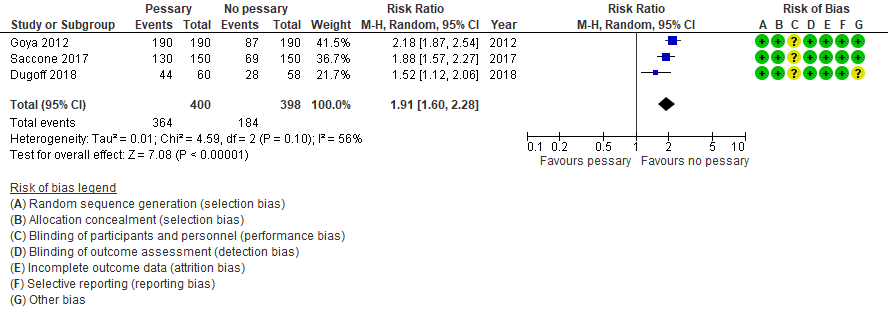
**

Conclusion: Pessary, compared with no pessary probably increases the risk of vaginal discharge in women with a singleton pregnancy and short cervical length (GRADE ⊕⊕⊕🌕 ).

**Preterm prelabor rupture of the membranes (PPROM) (**Appendix 4.3, STable 4.3.24 and SFigure 24)

A meta-analysis of five trials, including 1838 women showed no difference in the rate of PPROM, RR 0.83 (95% CI 0.44 to 1.56). The crude event rate across trials was 6.7% without pessary. The pooled weighted RD was -1.4 % percentage points (95% CI -5.1 to 2.3).

**SFigure 24.** Outcome: Preterm prelabor rupture of membranes.

**
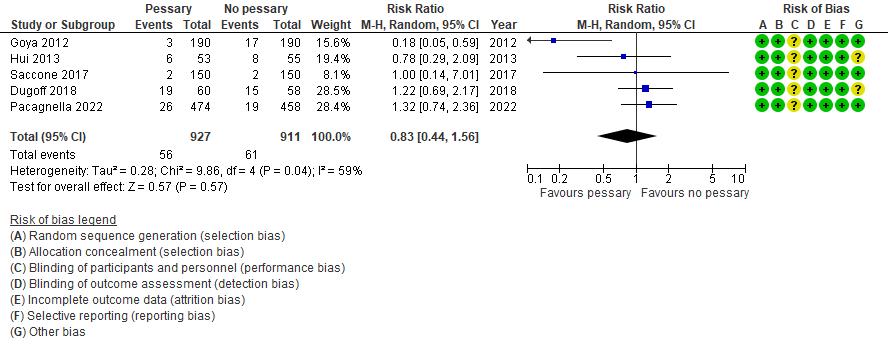
**

Conclusion: Pessary, compared with no pessary may result in no difference in PPROM in women with a singleton pregnancy and short cervical length (GRADE ⊕⊕🌕 🌕).
