# Supplementary material for: Progesterone, cerclage, pessary, or acetylsalicylic acid for prevention of preterm birth in singleton and multifetal pregnancies – A systematic review and meta-analyses
Source: Front Med (Lausanne). 2023 Feb 28;10:1111315. doi: 10.3389/fmed.2023.1111315 (PMC10015499; doi:10.3389/fmed.2023.1111315)
Supplement: Supplementary file 1 [file Data_Sheet_1.zip › Data Sheet 1_corrected/Appendix 5.4 Results ASA for_singletons.docx]

**Region Västra Götaland, HTA-centrum**

**Regional**

**HTA report 2022:130**

**Progesterone, cerclage, pessary, or acetylsalicylic acid for prevention of preterm birth in singleton and multifetal pregnancies**

**Appendix 5.4 Results Acetylsalicylic acid (ASA) versus placebo**

**Table of contents**

[Abbreviations 1](#_Toc119669329)

[Results per singleton pregnancies 2](#_Toc119669330)

# **Abbreviations**

ASA acetylsalicylic acid

CI confidence interval

RD risk difference

RR relative risk/risk ratio

# **Results per outcome**

**Preterm birth in singletons across gestational weeks**

**Any preterm birth <37 weeks** (Appendix 4.4, STable 4.4.1.a)

The outcome occurred in 21.2% in the ASA group vs. 25.4% in the placebo group, resulting in RR 0.83 (95% CI 0.58 to 1.20) and RD -4.25 (95% CI -12.7 to 4.2). In a prespecified subgroup analysis including only women with good medication adherence (≥80%), (n=246), the rate of preterm birth <37 gestational weeks was lower in the ASA group (19.2%) but remained similar in the placebo group (24.8%), resulting in RR 0.77 (95% CI 0.48 to 1.25).

Conclusion: ASA compared with placebo may result in no difference in the risk of any preterm birth before 37 gestational weeks in women with a singleton pregnancy and a history of previous spontaneous preterm birth (GRADE ⊕⊕🌕 🌕).

**Spontaneous preterm birth <37 weeks** (Appendix 4.4, STable 4.4.1.b)

The outcome occurred in 20.1% in the ASA group vs. 23.8% in the placebo group, resulting in RR 0.84 (95% CI 0.58 to 1.23) and RD -3.7 (95% CI -12.0 to 4.5).

Conclusion: ASA compared with placebo may result in no difference in the risk of spontaneous preterm birth before 37 gestational weeks in women with a singleton pregnancy and a history of previous spontaneous preterm birth (GRADE ⊕⊕🌕 🌕).

**Any preterm birth <34 weeks** (Appendix 4.4, STable 4.4.2.a)

The outcome occurred in 9.3% in the ASA group vs. 8.8% in the placebo group, resulting in RR 1.05 (95% CI 0.56 to 1.98) and RD 0.5 (95% CI -5.2 to 6.2).
Conclusion: ASA compared with placebo may result in no difference in the risk of any preterm birth before 34 gestational weeks in women with a singleton pregnancy and a history of previous spontaneous preterm birth (GRADE ⊕⊕🌕 🌕).

**Spontaneous preterm birth <34 weeks** (Appendix 4.4, STable 4.4.2.b)

The outcome occurred in 9.3% in the ASA group vs. 8.3% in the placebo group, resulting in RR 1.12 (95% CI 0.59 to 2.13) and RD 1.0 (95% CI -4.6 to 6.6).
Conclusion: ASA compared with placebo may result in no difference in the risk of spontaneous preterm birth before 34 gestational weeks in women with a singleton pregnancy and a history of previous spontaneous preterm birth (GRADE ⊕⊕🌕 🌕).

**Any preterm birth <28 weeks** (Appendix 4.4, STable 4.4.3.a)

The outcome occurred in 3.6% in the ASA group vs. 2.6% in the placebo group, resulting in RR 1.39 (95% CI 0.45 to 4.31) and RD 1.0 (95% CI -2.4 to 4.5).
Conclusion: ASA compared with placebo may result in no difference in the risk of any preterm birth before 28 gestational weeks in women with a singleton pregnancy and a history of previous spontaneous preterm birth (GRADE ⊕⊕🌕 🌕).

**Spontaneous preterm birth <28 weeks** (Appendix 4.4, STable 4.4.3.b)

The outcome occurred in 3.6% in the ASA group vs. 2.6% in the placebo group, resulting in RR 1.39 (95% CI 0.45 to 4.31) and RD 1.0 (95% CI -2.4 to 4.5).
Conclusion: ASA compared with placebo may result in no difference in the risk of spontaneous preterm birth before 28 gestational weeks in women with a singleton pregnancy and a history of previous spontaneous preterm birth (GRADE ⊕⊕🌕 🌕).

**Gestational length** (Appendix 4.4, STable 4.4.4)

Median gestational length (Q1; Q2) was 38+1 weeks in both the ASA group (37+1; 39+1) and the placebo group (36+6; 39+2). The certainty of evidence was downgraded one level due to serious imprecision.

Conclusion: ASA compared with placebo probably results in no difference in gestational length in women with a singleton pregnancy and a history of previous spontaneous preterm birth
(GRADE ⊕⊕⊕ 🌕).

**Mortality and morbidity in neonates from singleton pregnancies**

**Perinatal mortality** (Appendix 4.4, STable 4.4.5)

The outcome occurred in six (3.1%) cases in the ASA group vs. two (1.0%) cases in the placebo group, resulting in RR 2.99 (95% CI 0.61 to 14.60) and RD 2.1 (95% CI -0.8 to 4.9).
Conclusion: It is uncertain if ASA compared with placebo results in any difference in the risk of perinatal death in neonates from women with a singleton pregnancy and a history of previous spontaneous preterm birth (GRADE ⊕🌕🌕 🌕).

**Composite adverse neonatal outcome** (Appendix 4.4, STable 4.4.6)

The outcome occurred in 4.6% in the ASA group vs. 2.6% in the placebo group, resulting in RR1.79 (95% CI 0.61 to 5.25) and RD 2.0 (95% CI -1.7 to 5.8). The composite adverse outcome included any intrauterine fetal death after 16 weeks of gestation, neonatal death, bronchopulmonary dysplasia, intraventricular haemorrhage, necrotizing enterocolitis, confirmed sepsis, or retinopathy of prematurity.
Conclusion: It is uncertain if ASA compared with placebo results in any difference in the risk of composite adverse neonatal outcome in neonates from women with a singleton pregnancy and a history of previous spontaneous preterm birth (GRADE ⊕🌕🌕 🌕).

**Bronchopulmonary dysplasia** (Appendix 4.4, STable 4.4.7)

The outcome occurred in one (0.5%) neonate in the ASA group vs. three (1.6%) neonates in the placebo group, resulting in RR 0.33 (95% CI 0.04 to 3.16) and RD -1.0 (95% CI -3.0 to 1.0).
Conclusion: It is uncertain if ASA compared with placebo results in any difference in bronchopulmonary dysplasia in neonates from women with a singleton pregnancy and a history of previous spontaneous preterm birth (GRADE ⊕🌕🌕 🌕).

**Intraventricular haemorrhage** (Appendix 4.4, STable 4.4.8)

In the placebo group, the outcome occurred in one neonate in the ASA group vs. none in the placebo group.

Conclusion: It is uncertain if ASA compared with placebo results in any difference in I intraventricular haemorrhage in neonates from women with a singleton pregnancy and a history of previous spontaneous preterm birth (GRADE ⊕🌕🌕 🌕).

**Necrotizing enterocolitis** (Appendix 4.4, STable 4.4.9)

The outcome occurred in one neonate in the ASA group vs. none in the placebo group.
Conclusion: It is uncertain if ASA compared with placebo results in any difference in necrotizing enterocolitis in neonates from women with a singleton pregnancy and a history of previous spontaneous preterm birth (GRADE ⊕🌕🌕 🌕).

**Neonatal sepsis** (Appendix 4.4, STable 4.4.10)

The outcome occurred in four (2.1%) neonates in the ASA group vs. two (1.0%) neonates in the placebo group, resulting in RR 1.99 (95% CI 0.37 to 10.74) and RD 1.0 (95% CI -1.4 to 3.5).
Conclusion: It is uncertain if ASA compared with placebo results in any difference in neonatal sepsis in neonates from women with a singleton pregnancy and a history of previous spontaneous preterm birth (GRADE ⊕🌕🌕 🌕).

**Retinopathy of prematurity (ROP)** (Appendix 4.4, STable 4.4.11)

The outcome occurred in one (0.5%) neonate in the ASA group vs. two (1.0%) neonates in the placebo group, resulting in RR 0.50 (95% CI 0.05 to 5.44) and RD 0.5 (95% CI -2.3 to 1.2).
Conclusion: It is uncertain if ASA compared with placebo results in any difference in retinopathy of prematurity in neonates from women with a singleton pregnancy and a history of previous spontaneous preterm birth (GRADE ⊕🌕🌕 🌕).

**Admittance to neonatal intensive care unit** (Appendix 4.4, STable 4.4.12)

The outcome occurred in 6.7% in the ASA group vs. 5.7% in the placebo group, resulting in RR 1.18 (95% CI 0.54 to 2.56) and RD 1.0 (95% CI -3.8 to 5.8). The median days admitted was twelve days in the ASA group vs. seven days in the placebo group.

Conclusion: ASA compared with placebo may result in no difference in admittance to neonatal intensive care unit in neonates from women with a singleton pregnancy and a history of previous spontaneous preterm birth (GRADE ⊕⊕🌕 🌕).

**Mortality and morbidity in women with singleton pregnancies**

**Maternal mortality** (Appendix 4.4, STable 4.4.13)

No deaths were reported in this trial (GRADE ⊕🌕🌕 🌕).

**Hypertensive disorders in pregnancy** (Appendix 4.4, STable 4.4.14)

Gestational hypertension occurred in 2.1% in the ASA group vs. 2.6% in the placebo group, resulting in RR 0.80 (95% CI 0.22 to 2.92) and RD -0.5 (95% CI -3.5 to 2.5). Preeclampsia occurred in two cases each in the ASA and placebo groups, resulting in RR 1.00 (95% CI 0.14 to 6.99) and RD -0.0 (95% CI -2.0 to 2.0). Eclampsia did not occur in any of the groups.
Conclusion: It is uncertain if ASA compared with placebo results in any difference in gestational hypertension, preeclampsia, or eclampsia, in women with a singleton pregnancy and a history of previous spontaneous preterm birth (GRADE ⊕🌕 🌕 🌕).

**Gestational diabetes mellitus** (Appendix 4.4, STable 4.4.15)

The outcome occurred in 7.7% in the ASA group vs. 7.8% in the placebo group, resulting in RR 1.0 (95% CI 0.50 to 1.98) and RD -0.0 (95% CI -5.4 to 5.3).
Conclusion: ASA compared with placebo may result in no difference in gestational diabetes mellitus in women with a singleton pregnancy and a history of previous spontaneous preterm birth (GRADE ⊕⊕ 🌕 🌕).

**Infection** (Appendix 4.4, STable 4.4.16)

Urinary tract infection or genital infection treated with antibiotics occurred in 3.1% in the ASA group vs. 7.8% in the placebo group, resulting in RR 0.40 (95% CI 0.16 to 1.00) and RD -4.7 (95% CI -9.2 to -0.2).
Conclusion: It is uncertain if ASA compared with placebo results in any difference in urinary tract infection or genital infections in women with a singleton pregnancy and a history of previous spontaneous preterm birth (GRADE ⊕🌕 🌕 🌕).

**Bleeding** (Appendix 4.4, STable 4.4.17)

Vaginal bleeding occurred in 4.7% in the ASA group vs. 6.0% in the placebo group, resulting in RR 0.78 (95% CI 0.26 to 2.39) and RD -1.3(95% CI -7.2 to 4.6). Other bleedings (defined as anal bleeding, epistaxis, prolonged wound bleeding, gingival bleeding) occurred in 16.0% in the ASA group vs. 10.4% in the placebo group, resulting in RR 1.54 (95% CI 0.77 to 3.01) and RD 5.7 (95% CI -3.2 to 14.6).

Conclusion: It is uncertain if ASA compared with placebo results in any difference in bleeding in women with a singleton pregnancy and a history of previous spontaneous preterm birth
(GRADE ⊕🌕 🌕 🌕).

**Preterm prelabor rupture of the membranes** (Appendix 4.4, STable 4.4.18)

The outcome occurred in 4.6% in the ASA group vs. 9.3% in the placebo group, resulting in RR 0.50 (95% CI 0.23 to 1.08) and RD -4.7 (95% CI -9.8 to 0.4).
Conclusion: ASA compared with placebo may result in no difference in the risk of preterm prelabor rupture of the membranes in women with a singleton pregnancy and a history of previous spontaneous preterm birth
(GRADE ⊕⊕🌕 🌕).
