# Supplementary material for: Progesterone, cerclage, pessary, or acetylsalicylic acid for prevention of preterm birth in singleton and multifetal pregnancies – A systematic review and meta-analyses
Source: Front Med (Lausanne). 2023 Feb 28;10:1111315. doi: 10.3389/fmed.2023.1111315 (PMC10015499; doi:10.3389/fmed.2023.1111315)
Supplement: Supplementary file 1 [file Data_Sheet_1.zip › Data Sheet 1_corrected/Appendix 6.1 Results progesterone vs placebo in multifetal pregnancies .docx]

**Region -centrum**

**Progesterone, cerclage, pessary, or acetylsalicylic acid for prevention of preterm birth in singleton and multifetal pregnancies**

**Appendix 6.1 Results progesterone vs placebo in multifetal pregnancies**

**Table of contents**

[**Abbreviations1**](#_Abbreviations)

[**STable 1.** Risk of bias legend](#_STable_1._Risk)2

[**Results per outcome progesterone vs placebo in multifetal pregnancies**](#_Results_per_outcome)2

[Preterm birth SFigures 1-11 (SFigures 1, 5, 7 corrected)](#_SFigure_1._Outcome:)2-12

[Gestational age and birth weight SFigures 12-14](#_SFigure_12._Outcome:)12-13

[Neonatal mortality and morbidity SFigures 15-2314-1](#_SFigure_15._Outcome:)9

[Maternal morbidity SFigures 24-281](#_SFigure_24._Outcome:)9-22

[**Subgroup analyses progesterone vs placebo in multifetal pregnancies**](#_Subgroup_analyses_progesterone)23

[**STable 2.** Summary estimates from subgroup meta-analyses exploring the effect of progesterone vs placebo in women with a multifetal pregnancy, according to administration route and specific risk factors for preterm birth.](#_STable_2._Summary)23

[Preterm birth SFigures 29-33](#_SFigure_29._Outcome:)24-25

# **Abbreviations**

17-OHPC 17-alpha-hydroxyprogesterone caproate

BPD bronchopulmonary dysplasia

CI confidence interval

GDM gestational diabetes mellitus

HDP hypertensive disorders in pregnancy

ICP intrahepatic cholestasis in pregnancy

im intramuscular injection

IVH intraventricular hemorrhage

mg milligram

mm millimetre

NEC necrotizing enterocolitis

NICU neonatal intensive care unit

PPROM preterm prelabor rupture of membranes

PTB preterm birth

RD risk difference

RDS respiratory distress syndrome

ROP retinopathy of prematurity

RR relative risk/risk ratio

sPTB spontaneous preterm birth

# **STable 1.** **Risk of bias legend to the colour plot within the following forests plots**

1. Random sequence generation (selection bias)
2. Allocation concealment (selection bias)
3. Blinding of participants and personnel (performance bias)
4. Blinding of outcome assessment (detection bias)
5. Incomplete outcome data (attrition bias)
6. Selective reporting (reporting bias)
7. Conflict of interest bias

# **Results per outcome**

**Preterm birth in multifetal pregnancies across gestational weeks**

**Any preterm birth <37 weeks** (Appendix 4.1, STable 4.1.1.a and SFigure 1)

A meta-analysis of ten trials with low risk of bias, including 4517 women, showed no difference in the rate of any preterm birth, RR 1.01 (95% CI 0.95 to 1.08). The crude event rate across trials was 57.2% without progesterone. The pooled weighted RD was -0.7 percentage points (95% CI -3.4 to 4.8).

# **SFigure 1.** Outcome: Any preterm birth <37 weeks.


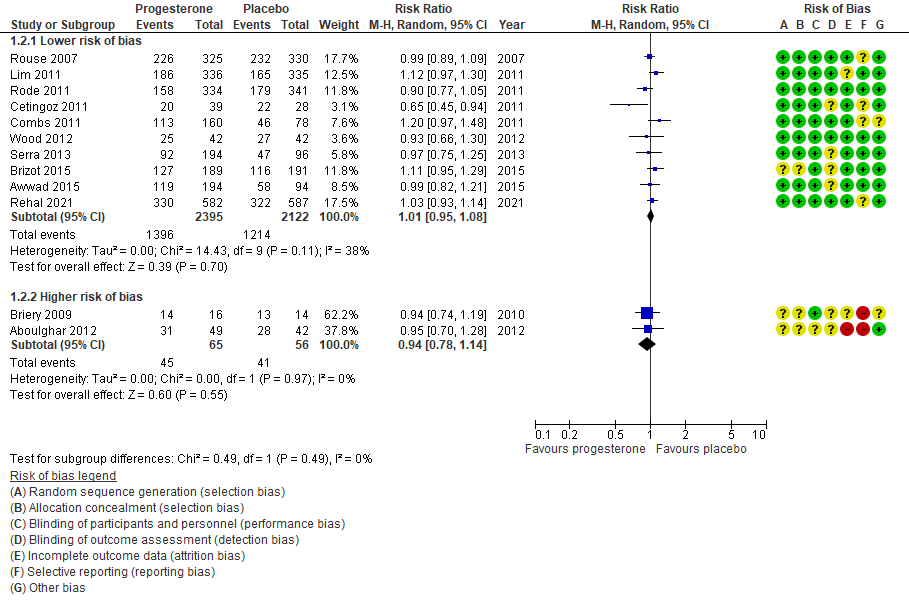


Conclusion: Progesterone compared with placebo probably results in little or no difference in the risk of any preterm birth before 37 gestational weeks in women with a multifetal pregnancy, neither considering administration route and dosage, nor additional risk factor(s) for preterm birth (GRADE ⊕⊕⊕ 🌕).

**SFigure 1.** Outcome: Any preterm birth <37 weeks. **Cetingoz et al. 2011 excluded from analysis** (article retracted 2024).


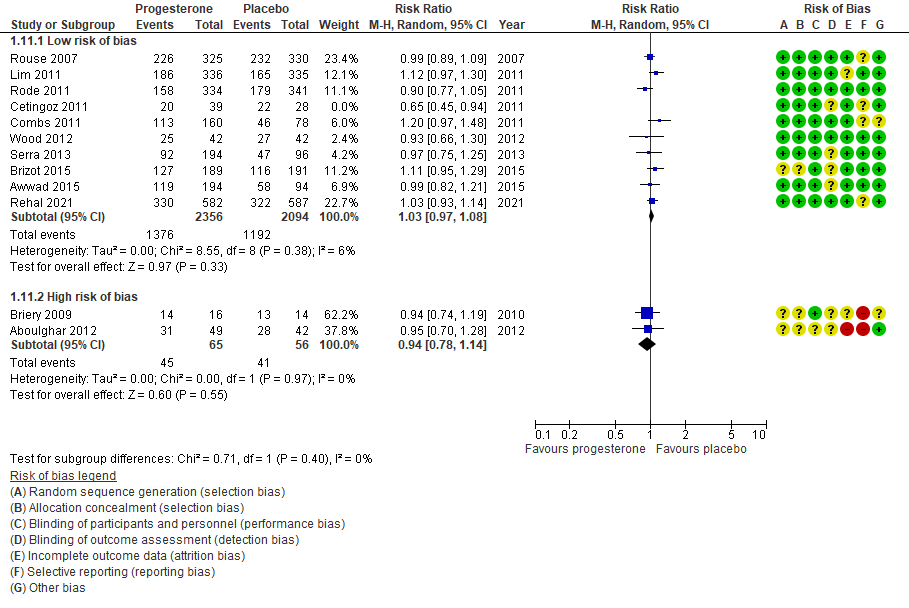


**Spontaneous preterm birth <37 weeks** (Appendix 4.1, STable 4.1.1.b and SFigure 2)

A meta-analysis of three trials with low risk of bias, including 1189 women, showed no difference in the rate of spontaneous preterm birth, RR 1.08 (95% CI 0.94 to 1.24). The crude event rate across trials was 37.0% without progesterone. The pooled weighted RD was 3.4 percentage points (95% CI -2.2 to 9.0).

**SFigure 2.** Outcome: Spontaneous preterm birth <37 weeks.


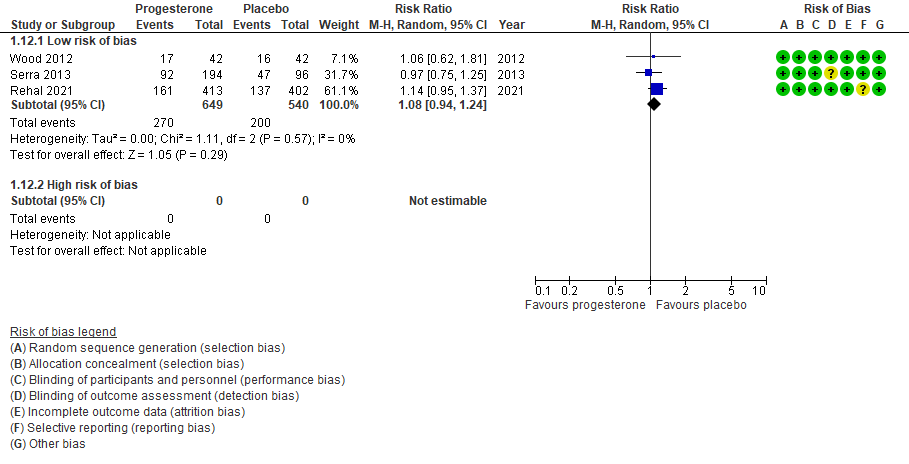


Conclusion: Vaginal progesterone compared with placebo probably results in no difference in the risk of spontaneous preterm birth before 37 gestational weeks in women with a multifetal pregnancy, neither considering dosage, nor additional risk factor(s) for preterm birth
(GRADE ⊕⊕⊕🌕).

**Any preterm birth <35 weeks** (Appendix 4.1, STable 4.1.2.a and SFigure 3**)**

A meta-analysis of four trials with low risk of bias, including 954 women, showed no difference in the rate of any preterm birth, RR 1.00 (95% CI 0.90 to 1.11). The crude event rate across trials was 46.0% without progesterone. The pooled weighted RD was 1.2 percentage points (95% CI -4.6 to 7.0).

**SFigure 3.** Outcome: Any preterm birth <35 weeks.


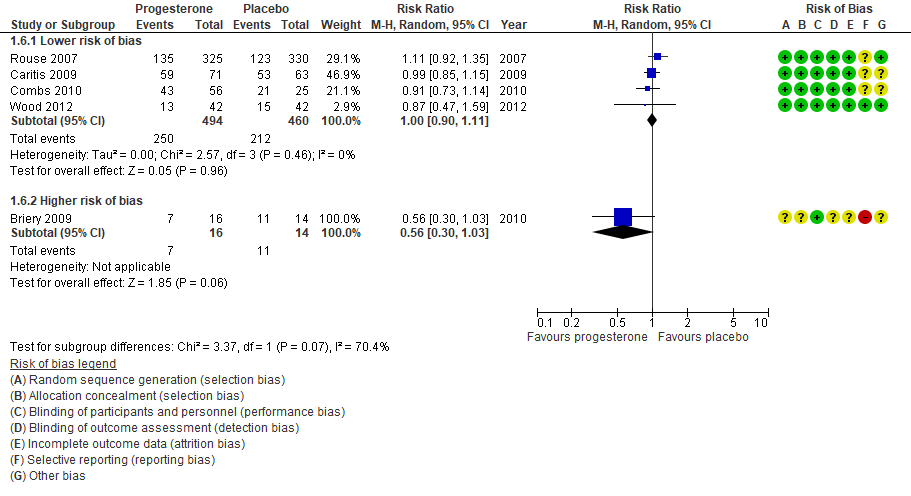


Conclusion: Progesterone compared with placebo results in little or no difference in the risk of any preterm birth before 35 gestational weeks in women with a multifetal pregnancy, neither considering administration route and dosage, nor additional risk factor(s) for preterm birth (GRADE ⊕⊕⊕ ⊕ ).

**Spontaneous preterm birth <35 weeks** (Appendix 4.1, STable 4.1.2.b and SFigure 4**)**

A meta-analysis of two trials with low risk of bias, including 788 women, compared 17-OHPC with placebo, showed no difference in the rate of spontaneous preterm birth, RR 1.17 (95% CI 0.96 to 1.44). The crude event rate across trials was 28.8% without progesterone. The pooled weighted RD was 5.1 percentage points (95% CI -1.3 to 11.5).

**SFigure 4.** Outcome: Spontaneous preterm birth <35 weeks.


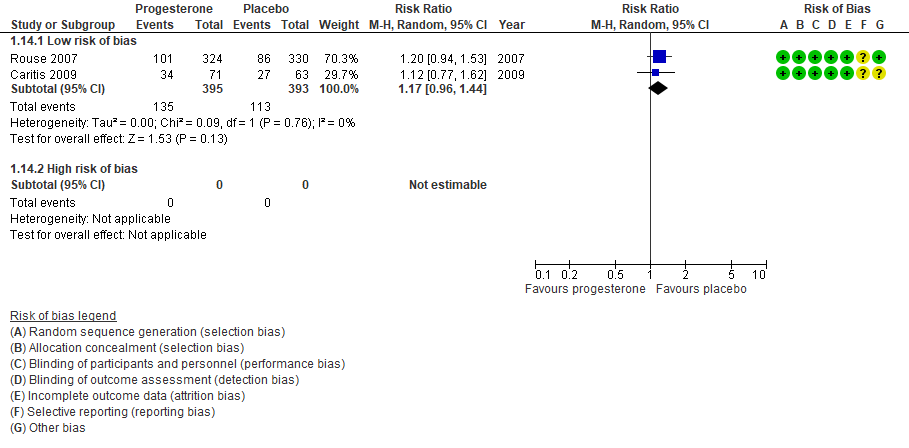


Conclusion: 17-OHPC, compared with placebo probably results in no difference in the risk of spontaneous preterm birth before 35 gestational weeks in women with a multifetal pregnancy, not considering additional risk factor(s) for preterm birth (GRADE ⊕⊕⊕ 🌕).

**Any preterm birth <34 weeks** (Appendix 4.1, STable 4.1.3.a and SFigure 5)

A meta-analysis of 15 trials with low risk of bias, including 5257 women, showed no difference in the rate of any preterm birth, RR 1.02 (95% CI 0.92 to 1.12). The crude event rate across trials was 21.6% without progesterone. The pooled weighted RD was 0.6 percentage points (95% CI -1.6 to 2.7).

**SFigure 5.** Outcome: Any preterm birth <34 weeks.


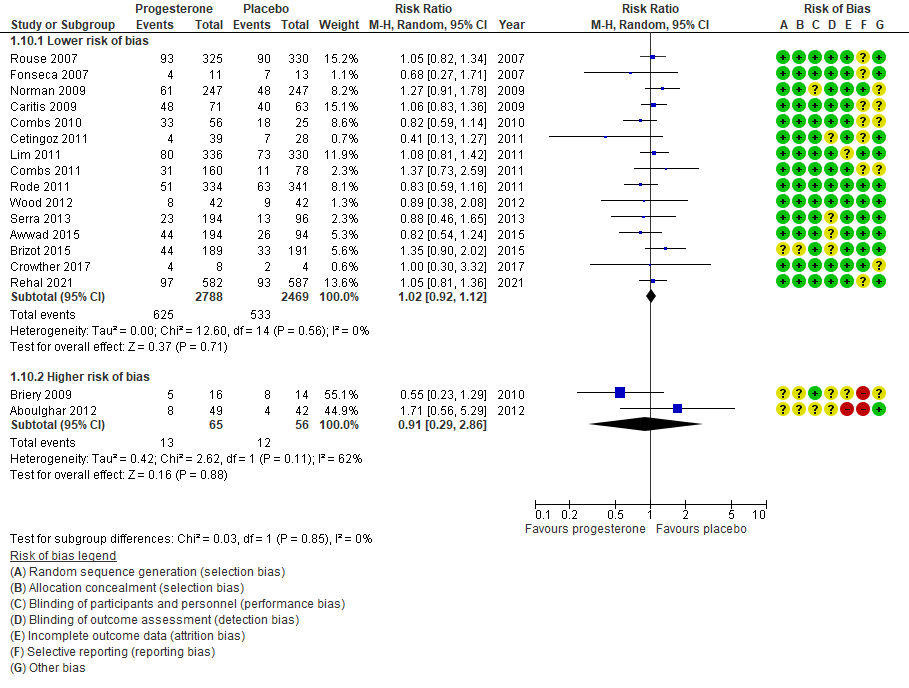


Results from Rouse 2007, Fonseca 2007, Caritis 2009, Lim 2011, Wood 2012, Awwad 2015, Crowther 2017 were retrieved from the IPD meta-analysis by Stewart et al. (EPPPIC, 2021).

Conclusion: Progesterone compared with placebo results in no difference in the risk of any preterm birth before 34 gestational weeks in women with a multifetal pregnancy, neither considering administration route and dosage, nor additional risk factor(s) for preterm birth (GRADE ⊕⊕⊕⊕).

**SFigure 5.** Outcome: Any preterm birth <34 weeks. **Cetingoz et al. 2011 excluded from analysis** (article retracted 2024).


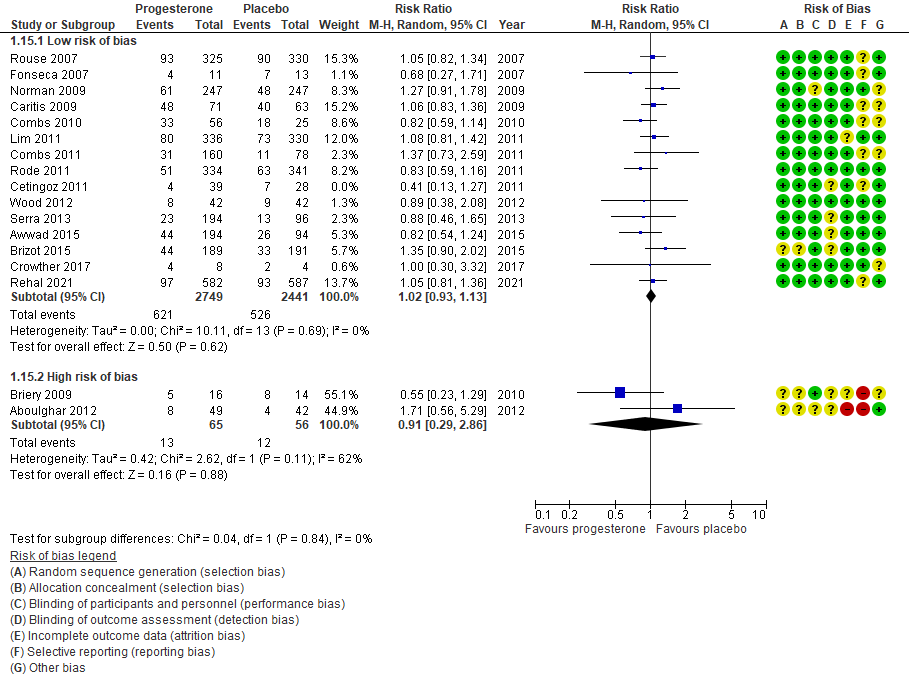


**Spontaneous preterm birth <34** weeks (Appendix 4.1, STable 4.1.3.b and SFigure 6)

A meta-analysis of four trials with low risk of bias, including 2372 women showed no difference in the rate of spontaneous preterm birth, RR 1.05 (95% CI 0.82 to 1.35). The crude event rate across trials was 11.7% without progesterone. The pooled weighted RD was 0.7 percentage points (95% CI -1.6 to 3.6).

**SFigure 6.** Outcome: Spontaneous preterm birth <34 weeks.


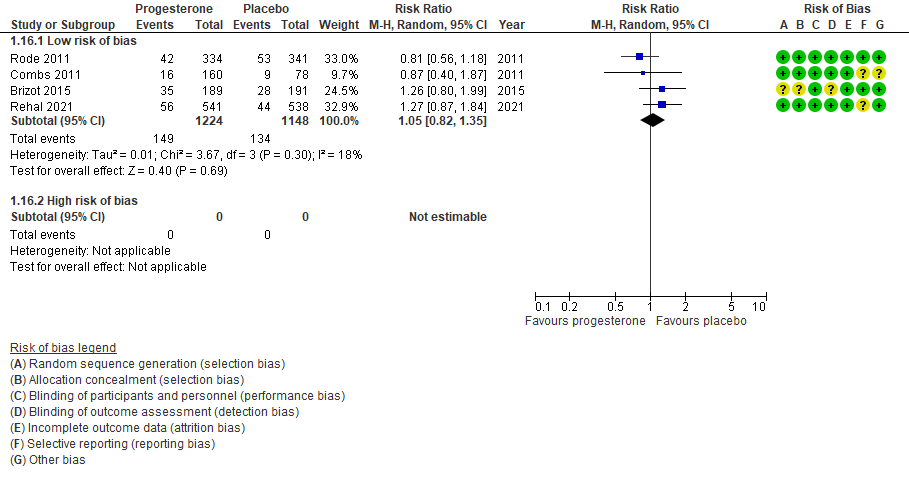


Conclusion: Progesterone compared with placebo results in no difference in the risk of spontaneous preterm birth before 34 gestational weeks in women with a multifetal pregnancy, neither considering administration route and dosage, nor additional risk factor(s) for preterm birth (GRADE ⊕⊕⊕⊕).

**Any preterm birth <33 weeks** (Appendix 4.1, STable 4.1.4.a and SFigure 7)

A meta-analysis of six trials with low risk of bias, including 95 women with short cervical length and a twin pregnancy showed no difference in the rate of preterm birth, RR 0.70 (95% CI 0.43 to 1.16). The crude event rate across trials was 55.8% without progesterone. The pooled weighted RD was 29.4 percentage points (95% CI -58.9 to 0.1).

**SFigure 7.** Outcome: Any preterm birth <33 weeks. All patients had a twin pregnancy and short cervical length according to inclusion criteria (Fonseca) or constitute subgroups of the other trials.


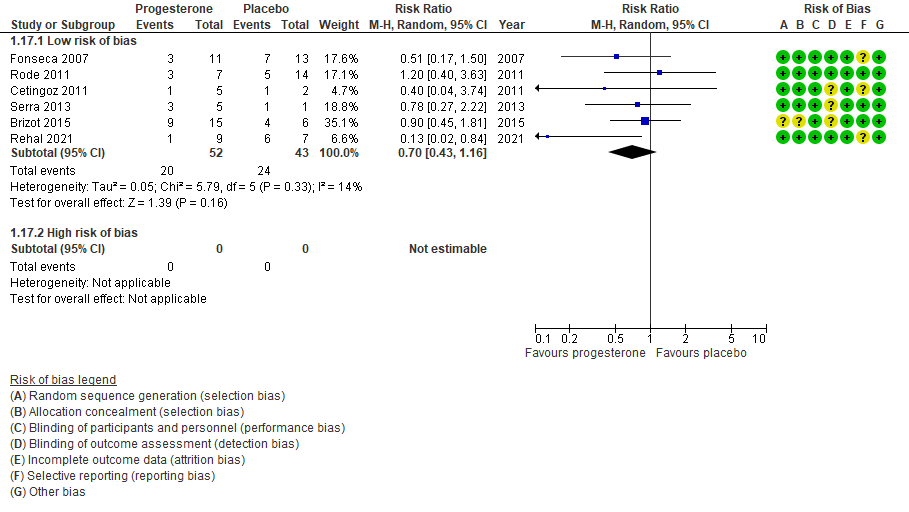


Results were retrieved from the IPD meta-analyses by Romero et al., 2017 and Romero et al., 2022.

Dosage/administration: 200 mg vaginal progesterone/d in all but one trial, Cetingoz 2011 used 100 mg.

Cut-off cervical length: ≤25 mm in all but one trial, Fonseca 2007 used ≤15 mm.

Conclusion: Vaginal progesterone, not considering dosage, compared with placebo may result in no difference in any preterm birth before 33 gestational weeks in women with a twin pregnancy and short cervical length (GRADE ⊕⊕🌕🌕).

**SFigure 7.** Outcome: Any preterm birth <33 weeks. **Cetingoz et al. 2011 excluded from analysis** (article retracted 2024).


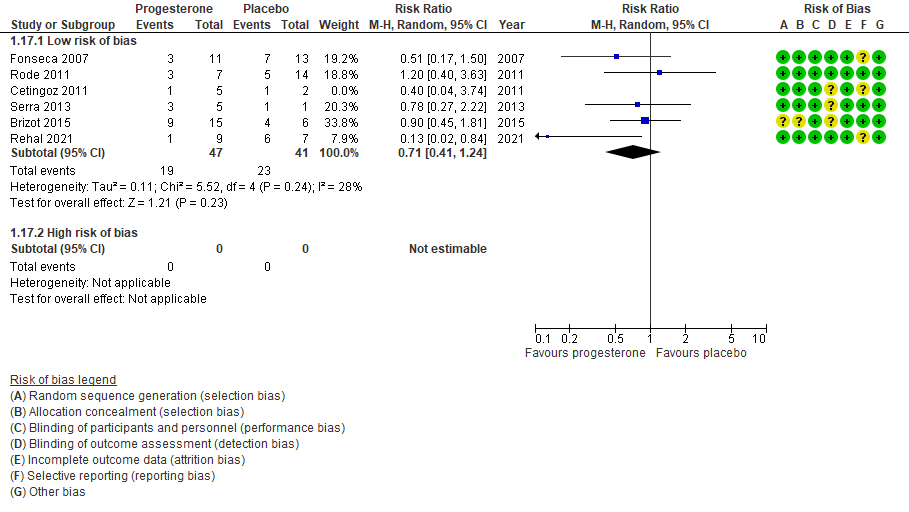


**Spontaneous preterm birth <33 weeks**

No trial reported spontaneous preterm birth <33 weeks.

**Any preterm birth <32 weeks** (Appendix 4.1, STable 4.1.5.a and SFigure 8)

A meta-analysis of ten trials with low risk of bias, including 4581 women showed no difference in the rate of any preterm birth, RR 1.00 (95% CI 0.81 to 1.23). The crude event rate across trials was 11.8% without progesterone. The pooled weighted RD was 0.3 percentage points (95% CI -2.1 to 2.8).

**SFigure 8.** Outcome: Any preterm birth <32 weeks.


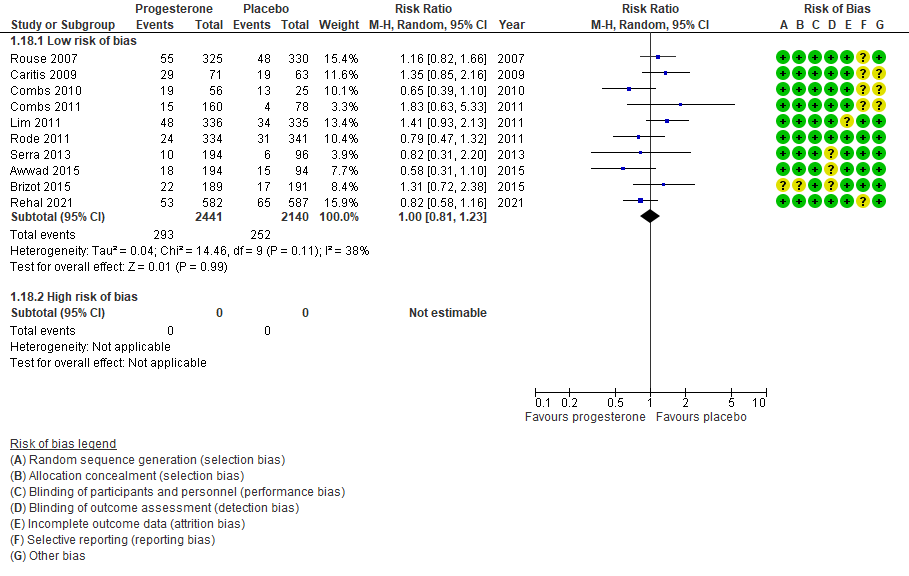


Conclusion: Progesterone compared with placebo results in no difference in the risk of any preterm birth before 32 gestational weeks in women with a multifetal pregnancy, neither considering administration route and dosage, nor additional risk factor(s) for preterm birth (GRADE ⊕⊕⊕⊕).

**Spontaneous birth <32 weeks** (Appendix 4.1, STable 4.1.5.b and SFigure 9)

One trial with low risk of bias, including 1100 women with a twin pregnancy showed no difference in the rate of spontaneous preterm birth, RR 1.03 (95% CI 0.59 to 1.77). The crude event rate across trials was 4.4% without progesterone. The pooled weighted RD was 0.1 percentage points (95% CI -2.3 to 2.6).

**SFigure 9.** Outcome: Spontaneous preterm birth <32 weeks.


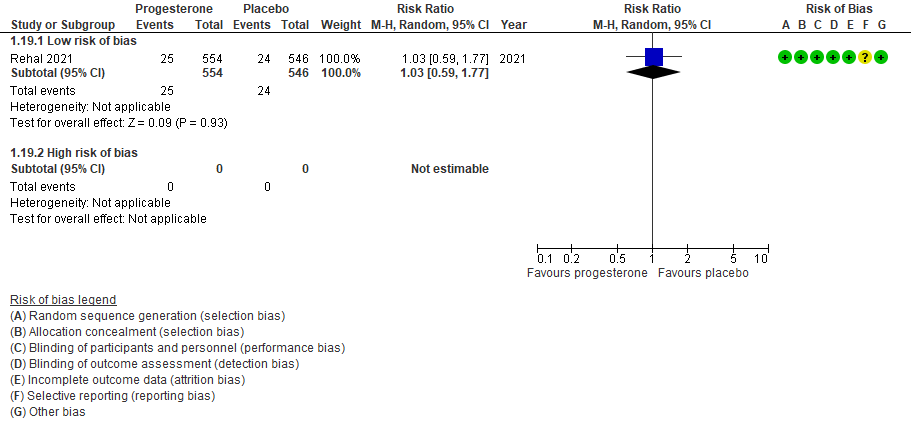


Conclusion: Vaginal progesterone compared with placebo may result in no difference in the risk of spontaneous preterm birth before 32 gestational weeks in women with a twin pregnancy, not considering additional risk factor(s) for preterm birth (GRADE ⊕⊕🌕 🌕).

**Any preterm birth <28 weeks** (Appendix 4.1, STable 4.1.6.a and SFigure 10)

A meta-analysis of ten trials with low risk of bias, including 4578 women showed no difference in the rate of any preterm birth, RR 0.99 (95% CI 0.76 to 1.29). The crude event rate across trials was 4.9% without progesterone. The pooled weighted RD was 0.2 percentage points (95% CI -0.9 to 1.3).

**SFigure 10**. Outcome: Any preterm birth <28 weeks.


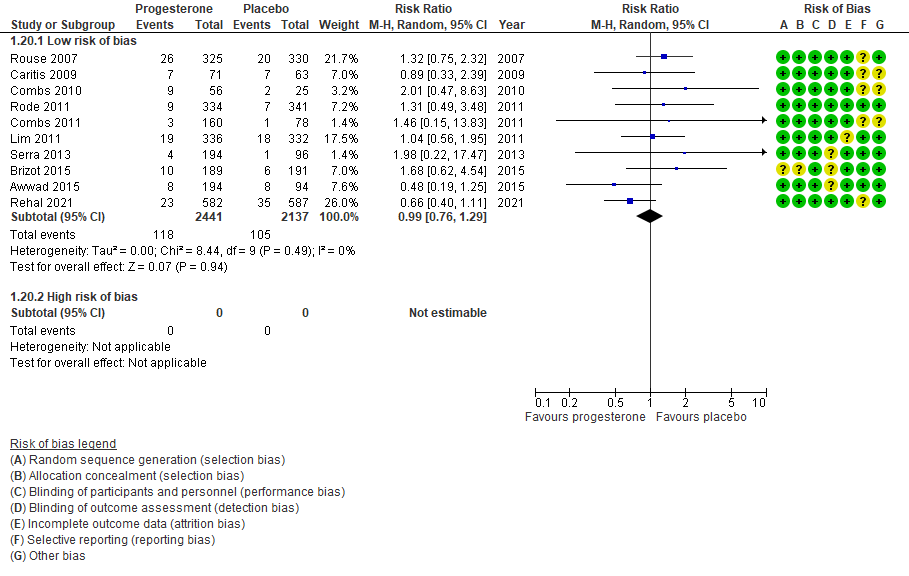


Conclusion: Progesterone compared with placebo results in no difference in the risk of any preterm birth before 28 gestational weeks in women with a multifetal pregnancy, neither considering administration route and dosage, nor additional risk factor(s) for preterm birth (GRADE ⊕⊕⊕⊕).

**Spontaneous birth <28 weeks** (Appendix 4.1, STable 4.1.6.b and SFigure 11)

One trial with low risk of bias, including 1126 women with a twin pregnancy showed no difference in the rate of spontaneous preterm birth, RR 1.13 (95% CI 0.41 to 3.09). The crude event rate across trials was 1.2% without progesterone. The pooled weighted RD was 0.2 percentage points (95% CI -1.2 to 1.5).

**SFigure 11.** Outcome: Spontaneous preterm birth <28 weeks.


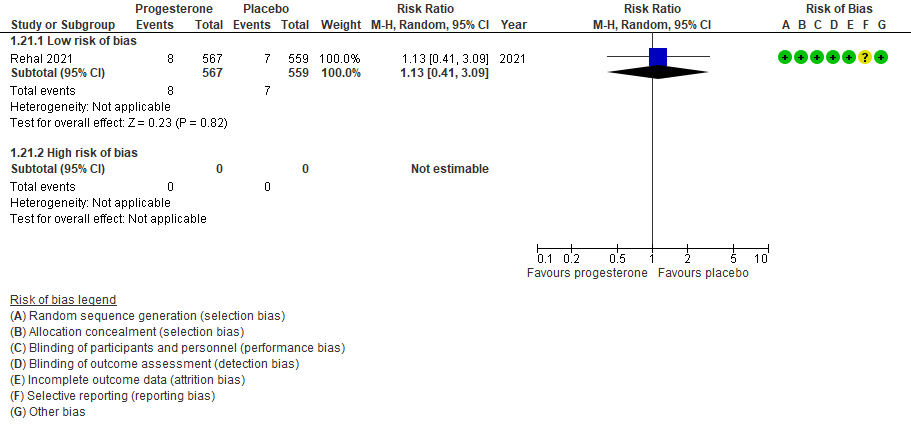


Conclusion: Vaginal progesterone compared with placebo may result in no difference in the risk of spontaneous preterm birth before 28 gestational weeks in women with a twin pregnancy, not considering additional risk factor(s) for preterm birth (GRADE ⊕⊕🌕 🌕).

**Gestational age and birth weight in multifetal pregnancies**

**Outcome gestational age** (Appendix 4.1, STable 4.1.7 and SFigure 12)

A meta-analysis of ten trials with low risk of bias, including 3903 women showed no mean difference in gestational age, -0.18 (-0.37 to 0.02) weeks, corresponding to approximately one day less (three to zero days less) gestational length in the progesterone group.

# **SFigure 12**. Outcome: Gestational age at delivery (weeks).


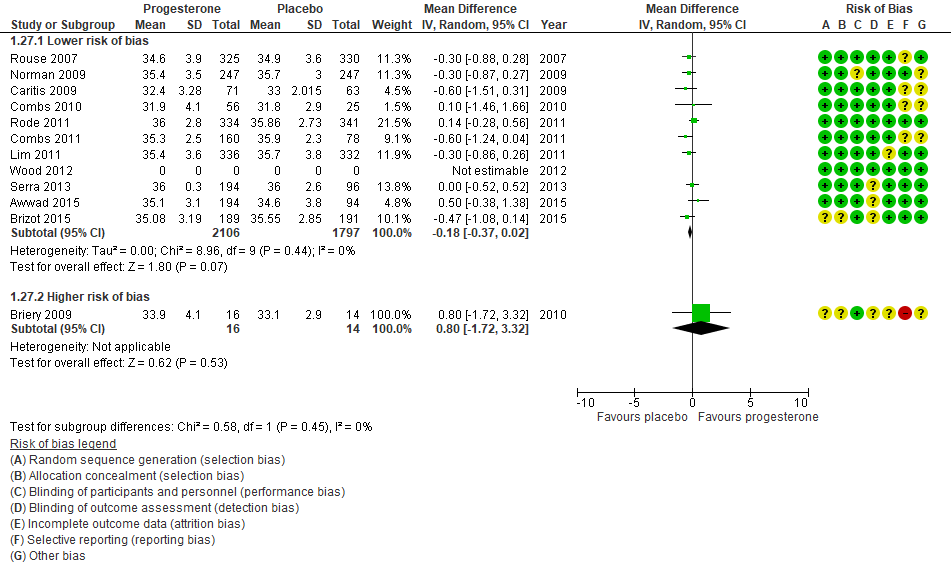


Conclusion: Progesterone compared with placebo results in little or no difference in gestational age, in women with a multifetal pregnancy, neither considering administration route and dosage, nor additional risk factor(s) for preterm birth (GRADE ⊕⊕⊕⊕).

**Outcome low birth weight (<2500 g)** (Appendix 4.1, STable 4.1.8, SFigure 13)

A meta-analysis of eight trials with low risk of bias, including 6711 neonates showed no difference in the rate of low birth weight, RR 0.97 (95% CI 0.91 to 1.04). The crude event rate across trials was 60.0% without progesterone. The pooled weighted RD was -1.6 percentage points (95% CI -5.8 to 2.7).

**SFigure 13**. Outcome: Low birth weight (<2500 g).


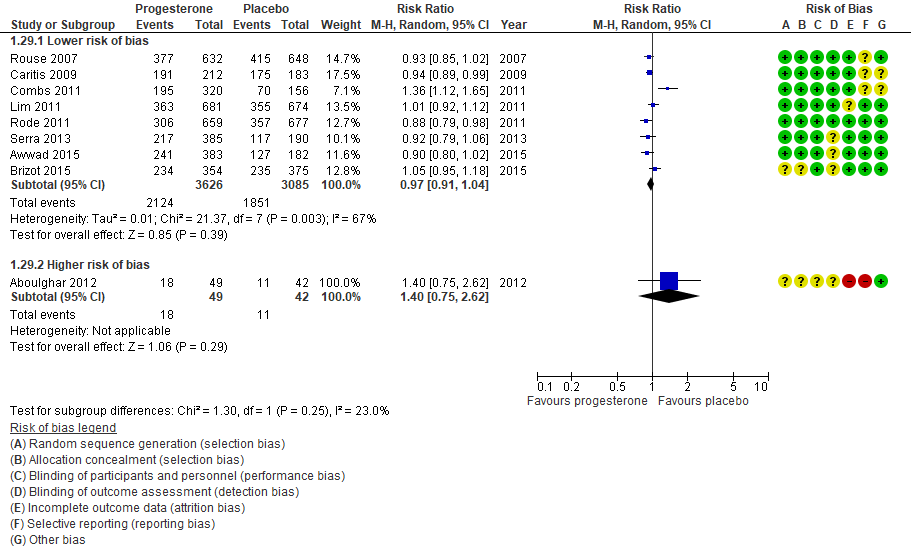


Conclusion: Progesterone compared with placebo results in little or no difference in the risk of low birth weight in neonates from a multifetal pregnancy, neither considering administration route and dosage, nor additional maternal risk factor(s) for preterm birth (GRADE ⊕⊕⊕⊕).

**Very low birth weight (<1500 g)** (Appendix 4.1, STable 4.1.9 and SFigure 14)

A meta-analysis of five trials with low risk of bias, including 8950 neonates showed no difference in the rate of very low birth weight, RR 1.07 (95% CI 0.84 to 1.36). The crude event rate across trials was 9.1% without progesterone. The pooled weighted RD was 1.1 percentage points (95% CI -1.4 to 3.6).

**SFigure 14.** Outcome: Very low birth weight (<1500 g).


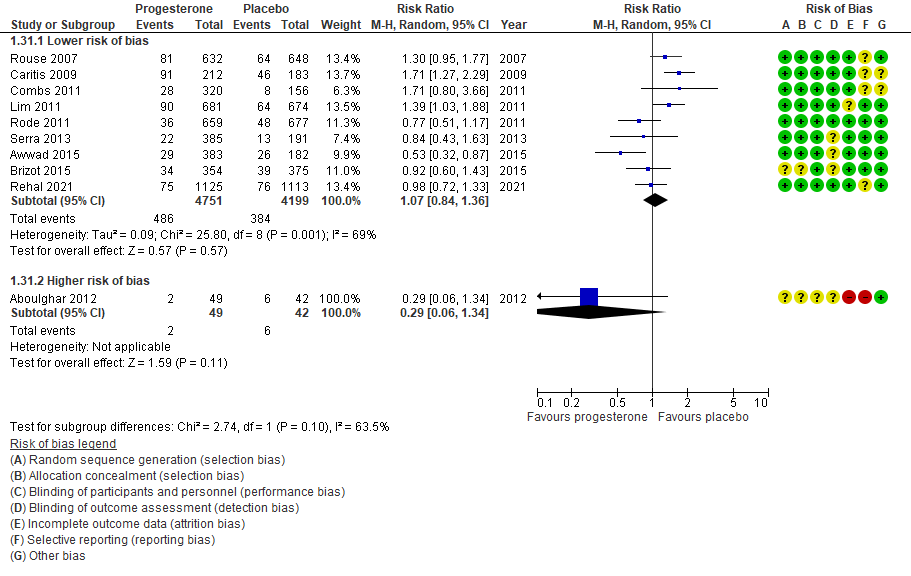


Conclusion: Progesterone compared with placebo probably results in no difference in the risk of very low birth weight in neonates from a multifetal pregnancy, neither considering administration route and dosage, nor additional maternal risk factor(s) for preterm birth (GRADE⊕⊕⊕🌕).

**Mortality and morbidity in neonates from multifetal pregnancies**

**Perinatal mortality** (Appendix 4.1, STable 4.1.10)

Twelve trials reported perinatal mortality in multifetal pregnancies. No meta-analysis on perinatal mortality was performed due to different or lack of definitions in the trials.

**Neonatal mortality <7 days**

No trial reported neonatal mortality <7 days.

**Neonatal mortality <28 days** (Appendix 4.1, STable 4.1.11 and SFigure 15)

A meta-analysis of ten studies with low risk of bias, including 6869 neonates, showed no difference in the rate of neonatal mortality <28 days, RR 0.96 (95% CI 0.60 to 1.53). The crude event rate across trials was 1.7% without progesterone. The pooled weighted RD was 0.0 percentage points (95% CI -0.6 to 0.7).

# **SFigure 15.** Outcome: Neonatal mortality <28 days.


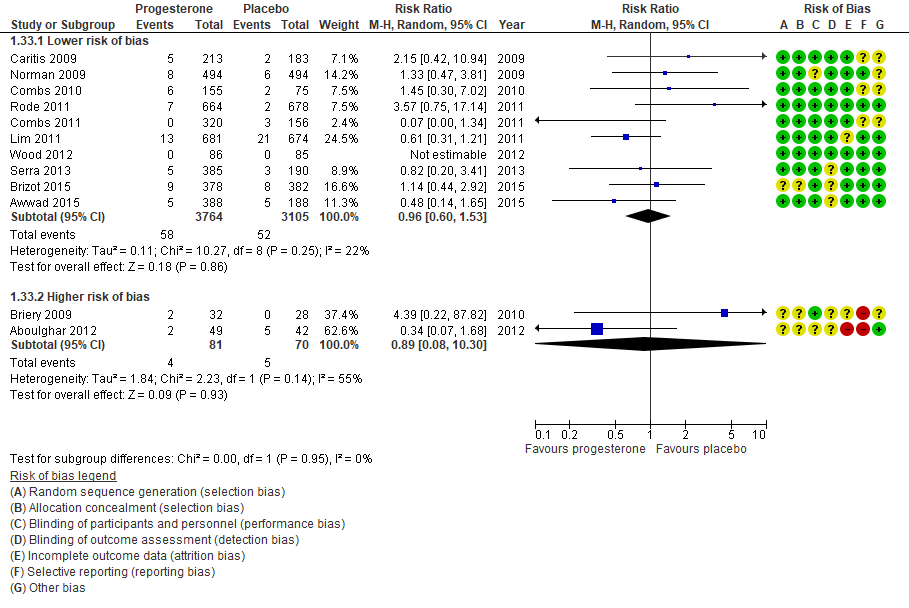


Conclusion: Progesterone compared with placebo probably results in no difference in the risk of neonatal mortality <28 days in neonates from a multifetal pregnancy, neither considering administration route and dosage, nor additional maternal risk factor(s) for preterm birth (GRADE⊕⊕⊕🌕 ).

**Composite adverse neonatal outcome** (Appendix 4.1, STable 4.1.12 and SFigure 16)

A meta-analysis of seven trials with low risk of bias, including 4230 neonates showed no difference in the rate of composite adverse neonatal outcome, RR 0.98 (95% CI 0.80 to 1.21). The crude event rate across trials was 18.2% without progesterone. The pooled weighted RD was -0.5 percentage points (95% CI -4.5 to 3.5).

All studies included intraventricular haemorrhage, necrotizing enterocolitis, respiratory distress syndrome, pneumonia, or confirmed sepsis in the composite adverse outcome. Awwad et al. (2015), Serra et al. (2013), Caritis et al. (2009), and Combs et al. (2010, 2011) included periventricular leukomalacia. Awwad et al. (2015) and Serra et al. (2013) included patent ductus arteriosus. Awwad et al. (2015) and Serra et al. (2013) did not include mortality in the composite adverse neonatal outcome, while the other trials did.

**SFigure 16.** Outcome: Composite adverse neonatal outcome.


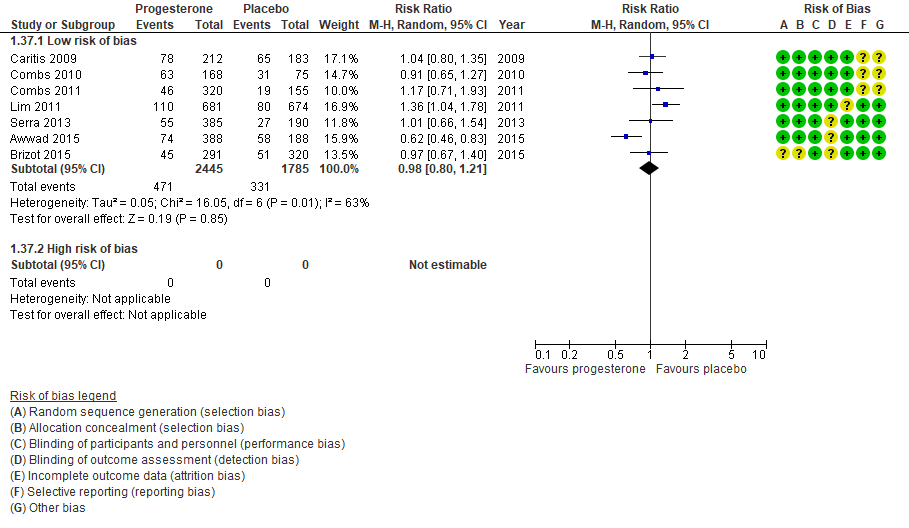


Conclusion: Progesterone compared with placebo may result in no difference in a composite adverse neonatal outcome in neonates from a multifetal pregnancy, neither considering administration route and dosage, nor additional maternal risk factor(s) for preterm birth (GRADE ⊕⊕🌕🌕).

**Respiratory distress syndrome (RDS)** (Appendix 4.1, STable 4.1.13 and SFigure 17)

A meta-analysis of eight trials with low risk of bias, including 5142 neonates showed no difference in the rate of RDS, RR 0.97 (95% CI 0.77 to 1.22). The crude event rate across trials was 13.8 without progesterone. The pooled weighted RD was -0.5 percentage points (95% CI -4.0 to 2.9).

**SFigure 17.** Outcome: Respiratory distress syndrome.


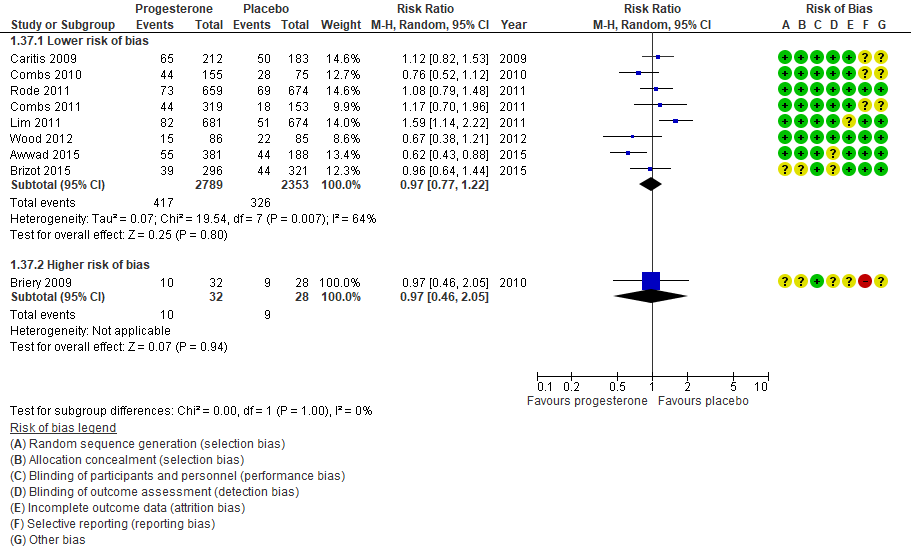


Conclusion: Progesterone compared with placebo probably results in no difference in the risk of RDS in neonates from a multifetal pregnancy, neither considering administration route and dosage, nor additional maternal risk factor(s) for preterm birth (GRADE ⊕⊕⊕🌕).

**Bronchopulmonary dysplasia (BPD)** (Appendix 4.1, STable 4.1.14 and SFigure 18)

A meta-analysis of six trials with low risk of bias, including 3175 neonates showed no difference in the rate of BPD, RR 0.87 (95% CI 0.50 to 1.51). The crude event rate across trials was 3.7% without progesterone. The pooled weighted RD was -0.2 percentage points (95% CI -2.7 to 2.3).

**SFigure 18**. Outcome: Bronchopulmonary dysplasia.


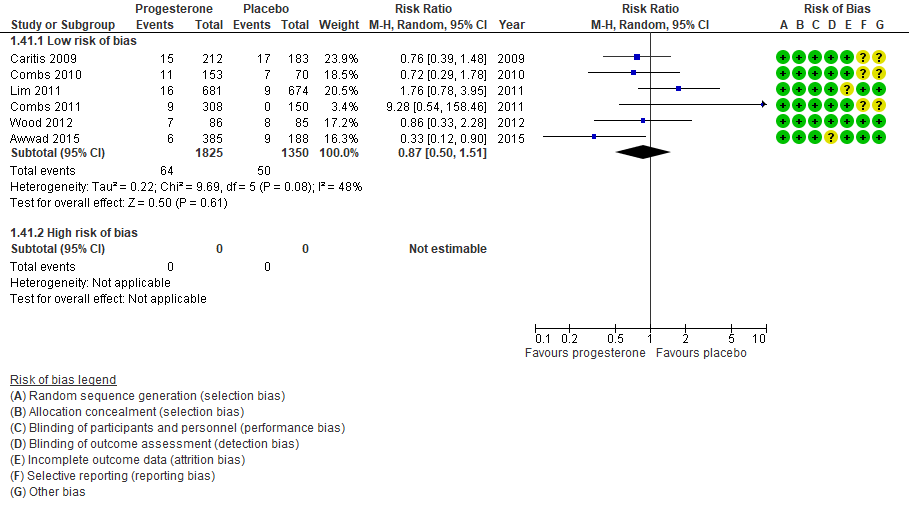


Conclusion: Progesterone compared with placebo may result in no difference in the risk of BPD in neonates from a multifetal pregnancy, neither considering administration route and dosage, nor additional maternal risk factor(s) for preterm birth (GRADE ⊕⊕🌕🌕).

**Intraventricular hemorrhage (IVH)** (Appendix 4.1, STable 4.1.15 and SFigure 19)

A meta-analysis of seven studies with low risk of bias, including 4559 neonates showed no difference in the rate of IVH, RR 1.38 (0.76 to 2.51). The crude event rate across trials was 0.8% without progesterone. The pooled weighted RD was 0.4 percentage points (95% CI -0.1 to 0.9).

**SFigure 19**. Outcome: Intraventricular hemorrhage.


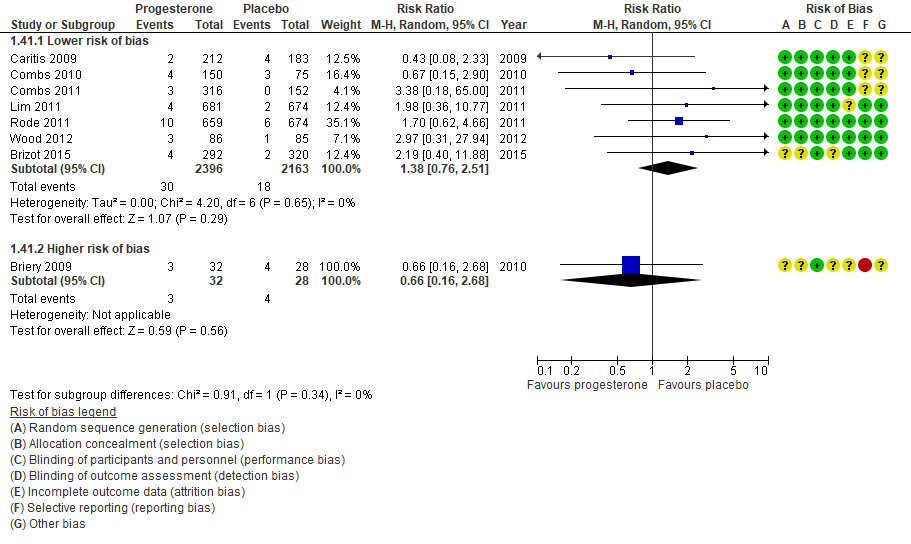


Conclusion: Progesterone compared with placebo may result in no difference in the risk of IVH in neonates from a multifetal pregnancy, neither considering administration route and dosage, nor additional maternal risk factor(s) for preterm birth (GRADE ⊕⊕🌕🌕).

**Necrotizing enterocolitis (NEC)** (Appendix 4.1, STable 4.1.16 and SFigure 20)

A meta-analysis of eight trials with low risk of bias, including 5137 neonates showed no difference in the rate of NEC, RR 0.73 (95% CI 0.41 to 1.30). The crude event rate across trials was 1.1% without progesterone. The pooled weighted RD was -0.1 percentage points (95% CI -0.5 to 0.2).

**SFigure 20.** Outcome: Necrotizing enterocolitis.


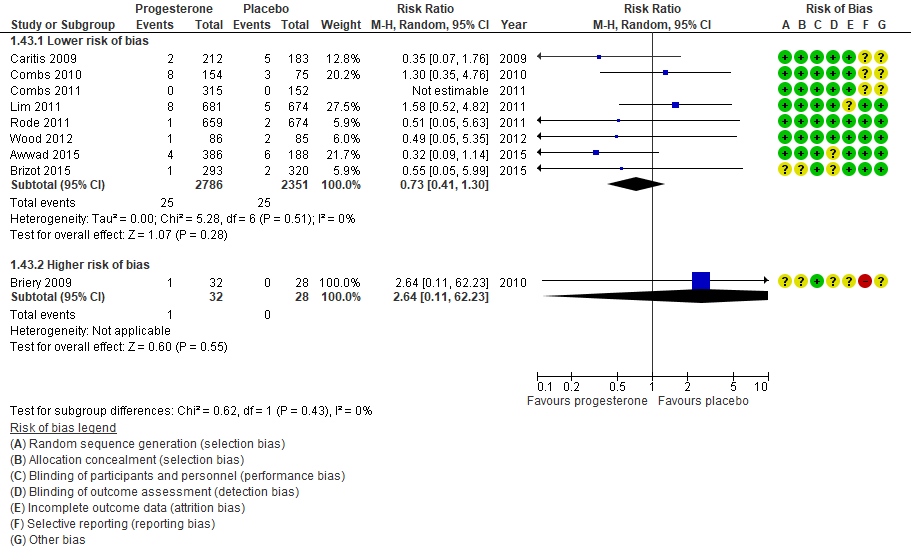


Conclusion: Progesterone compared with placebo may result in no difference in the risk of NEC in neonates from a multifetal pregnancy, neither considering administration route and dosage, nor additional maternal risk factor(s) for preterm birth (GRADE ⊕⊕🌕🌕).

**Neonatal sepsis** (Appendix 4.1, STable 4.1.17 and SFigure 21)

A meta-analysis of seven trials with low risk of bias, including 4968 neonates showed no difference in the rate of neonatal sepsis, RR 1.02 (95% CI 0.53 to 1.95). The crude event rate across trials was 3.5% without progesterone. The pooled weighted RD was 0.0 percentage points (95% CI -2.0 to 2.0).

**SFigure 21.** Outcome: Neonatal sepsis.


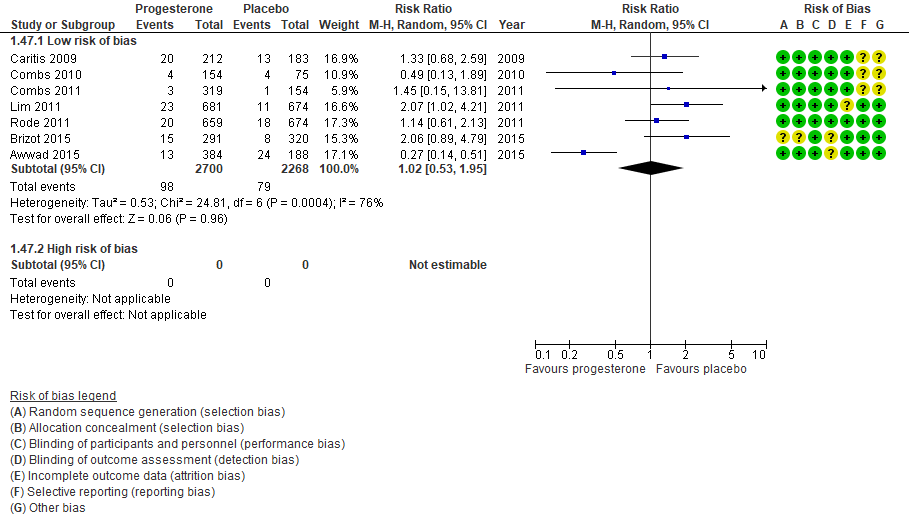


Conclusion: Progesterone compared with placebo may result in no difference in the risk of neonatal sepsis or other infection in neonates from a multifetal pregnancy, neither considering administration route and dosage, nor additional maternal risk factor(s) for preterm birth (GRADE ⊕⊕🌕🌕).

**Retinopathy of prematurity (ROP)** (Appendix 4.1, STable 4.1.18 and SFigure 22)

A meta-analysis of six trials with low risk of bias, including 3565 neonates showed no difference in the rate of ROP, RR 0.61 (95% CI 0.29 to 1.28). The crude event rate across trials was 1.5% without progesterone. The pooled weighted RD was -0.2 percentage points (95% CI -1.3 to 0.8).

**SFigure 22.** Outcome: Retinopathy of prematurity.


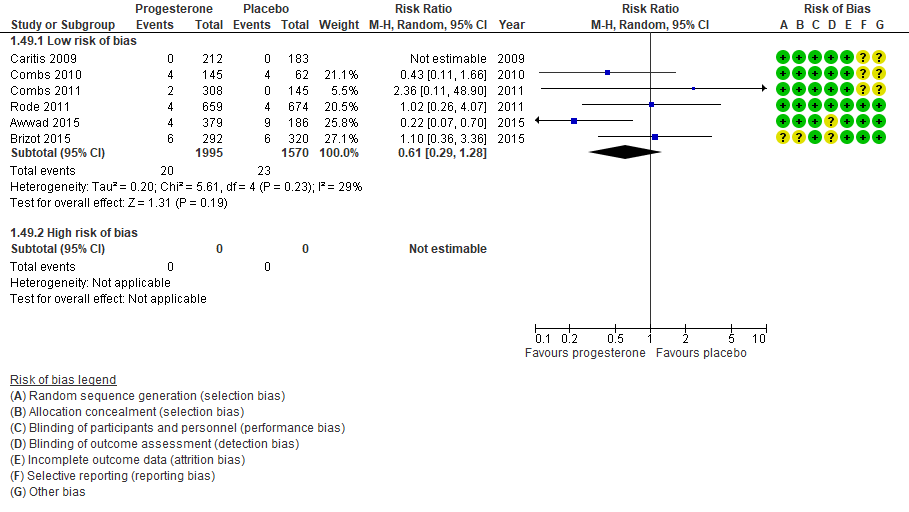


Conclusion: Progesterone compared with placebo may result in no difference in the risk of ROP in neonates from a multifetal pregnancy, neither considering administration route and dosage, nor additional maternal risk factor(s) for preterm birth (GRADE ⊕⊕🌕🌕).

**Admittance to neonatal intensive care unit (NICU) (**Appendix 4.1, STable 4.1.19 and SFigure 23)

A meta-analysis of five trials with low risk of bias, including 4968 neonates showed no difference in the rate of NICU admission, RR 1.00 (95% CI 0.79 to 1.27). The crude event rate across trials was 31.0% without progesterone. The pooled weighted RD was -0.4 percentage points (95% CI -6.6 to 5.9).

**SFigure 23.** Outcome: Admittance to neonatal intensive care unit (NICU).


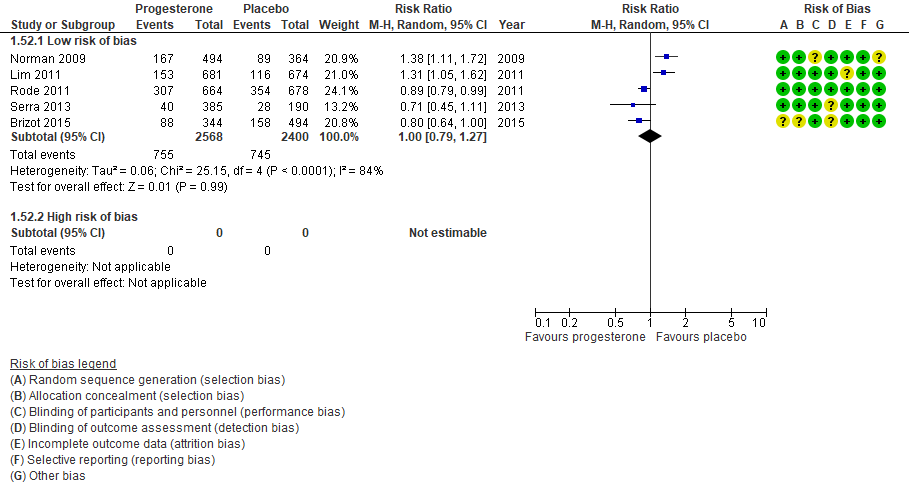


Conclusion: Progesterone compared with placebo probably result in no difference in admittance to NICU in neonates from a multifetal pregnancy, neither considering administration route and dosage, nor additional maternal risk factor(s) for preterm birth (GRADE ⊕⊕⊕🌕).

**Long-term child outcomes in multifetal pregnancies** (Appendix 4.1, STable 4.1.20**)**

Three trials (3030 children) examined long-term child outcomes in twins (Rode et al., 2011, PREDICT-study, follow up at 6- and 18-months of age, Vedel et al., 2016, follow up to eight years of age [follow up of Rode et al., 2011, PREDICT-study], McNamara et al., 2015, follow up at three to six years of age [follow up of Norman et al., 2009, STOPPIT-study]). Rode et al. (2011) and Vedel et al. (2016) measured the neurodevelopment at different ages with Ages and Stages Questionnaire (ASQ) and McNamara et al. (2015) used Child Developmental Inventory (CDI) score. Follow-up rate was between 44% and 80%. Cognitive development, general health, anthropometry and behaviour were similar between the groups. No meta-analysis was performed because of the heterogeneity of the studies.

Conclusion: Vaginal progesterone compared with placebo may result in no difference in cognitive development, general health, or behaviour in twins, neither considering dosage, nor additional maternal risk factor(s) for preterm birth.

**Maternal mortality and morbidity in women with a multifetal pregnancy**

**Maternal mortality**

No trial reported maternal mortality.

**Hypertensive disorders in pregnancy (HDP) (**Appendix 4.1, STable 4.1.22 and SFigure 24)

A meta-analysis of ten trials with low risk of bias, including 4502 women showed no difference in the rate of HDP, RR 1.02 (95% CI 0.84 to 1.25). The crude event rate across trials was 10.3% without progesterone. The pooled weighted RD was 0.6 percentage points (95% CI -1.1 to 2.3).

# **SFigure 24.** Outcome: Hypertensive disorder in pregnancy.


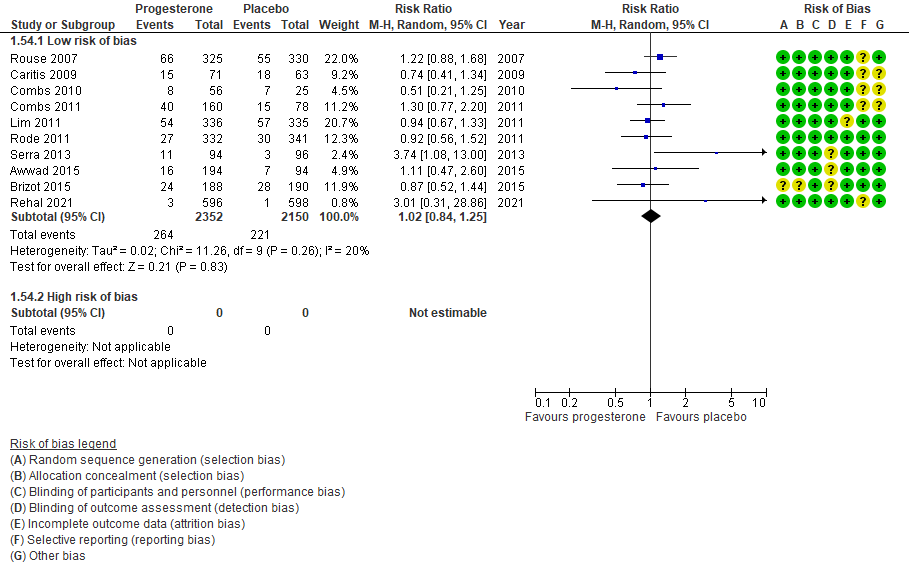


Conclusion: Progesterone compared with placebo results in no difference in HDP in women with a multifetal pregnancy, neither considering administration route and dosage, nor additional risk factor(s) for preterm birth (GRADE ⊕⊕⊕⊕).

**Gestational diabetes mellitus (GDM) (**Appendix 4.1, STable 4.1.23 and SFigure 25)

A meta-analysis of eight trials with low risk of bias, including 3268 women showed no difference in the rate of GDM, RR 1.02 (95% CI 0.77 to 1.35). The crude event rate across trials was 5.5% without progesterone. The pooled weighted RD was 0.1 percentage points (95% CI -1.4 to 1.5).

**SFigure 25**. Outcome: Gestational diabetes mellitus.


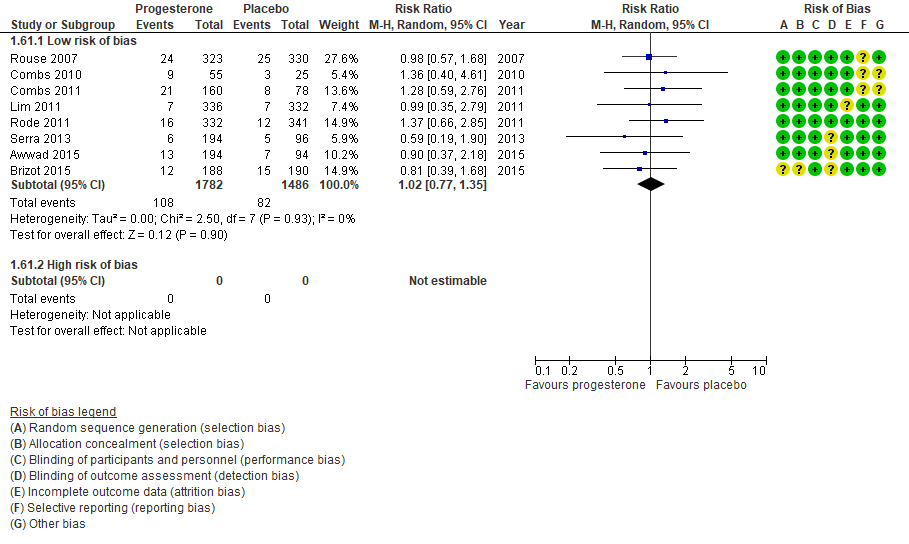


Data from Rouse 2007 was retrieved from a secondary publication, Gyamfi 2009, reporting specifically on GDM.

Conclusion: Progesterone compared with placebo probably result in no difference in the risk of GDM in women with a multifetal pregnancy, neither considering administration route and dosage, nor additional risk factor(s) for preterm birth (GRADE ⊕⊕⊕🌕).

**Intrahepatic cholestasis in pregnancy (ICP) (**Appendix 4.1, STable 4.1.24 and SFigure 26)

A meta-analysis of four trials with low risk of bias, including 2535 women showed no difference in the rate of ICP, RR 0.63 (95% CI 0.22 to 1.86). The crude event rate across trials was 2.3% without progesterone. The pooled weighted RD was -0.4 percentage points (95% CI -3.1 to 2.3).

**SFigure 26.** Outcome: Cholestasis.


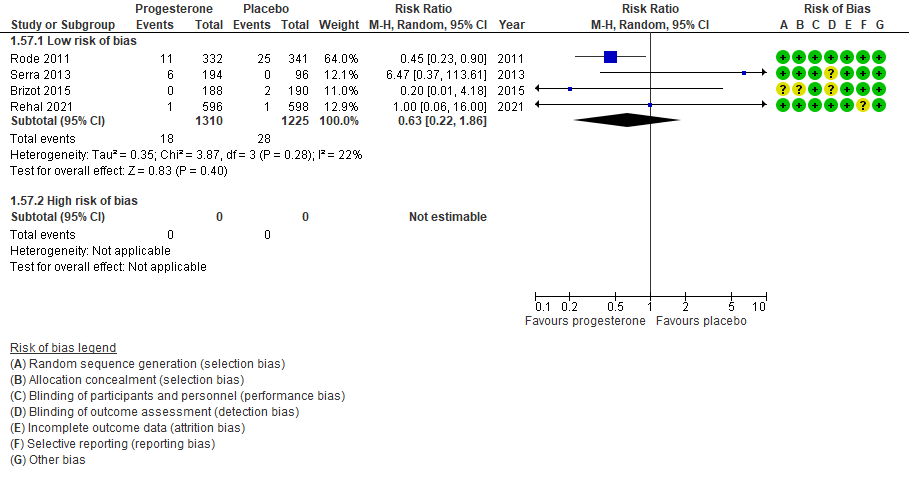


Conclusion: Progesterone compared with placebo may result in no difference in the risk of ICP in women with a multifetal pregnancy, neither considering administration route and dosage, nor additional risk factor(s) for preterm birth (GRADE ⊕⊕🌕🌕).

**Infections (chorioamnionitis) (**Appendix 4.1, STable 4.1.25 and SFigure 27)

A meta-analysis of five trials with low risk of bias, including 1773 women showed no difference in the rate of chorioamnionitis, RR 1.29 (95% CI 0.77 to 2.15). The crude event rate across trials was 2.8% without progesterone. The pooled weighted RD was 0.9 percentage points (95% CI -0.5 to 2.3).

**SFigure 27**. Outcome: Infection.


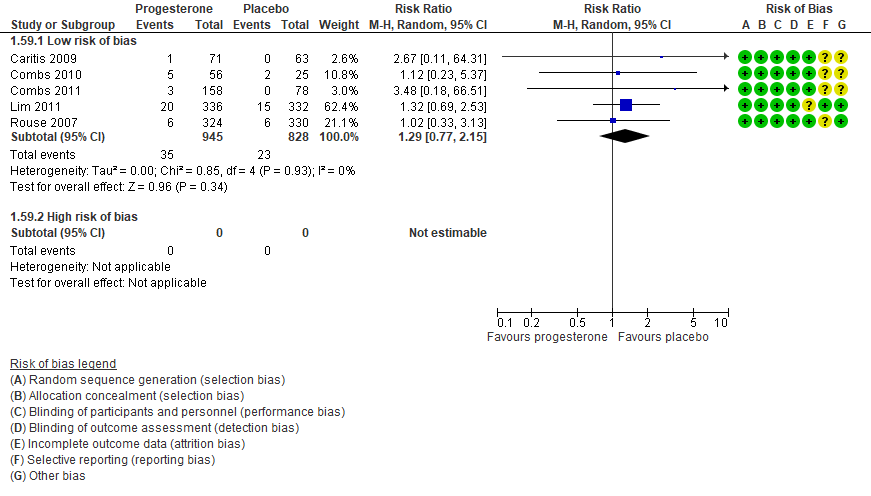


Conclusion: 17-OHPC compared with placebo may result in no difference in the risk of any infection in women with a multifetal pregnancy, neither considering additional risk factor(s) for preterm birth (GRADE ⊕⊕🌕🌕).

**Preterm prelabor rupture of the membranes (PPROM) (**Appendix 4.1, STable 4.1.26 and SFigure 28)

A meta-analysis of four trials with low risk of bias, including 1279 women showed no difference in the rate of PPROM, RR 1.09 (95% CI 0.73 to 1.63). The crude event rate across trials was 7.3% without progesterone. The pooled weighted RD was 0.4 percentage points (95% CI -2.2 to 3.0).

**SFigure 28.** Outcome: Preterm prelabor rupture of membranes (PPROM).


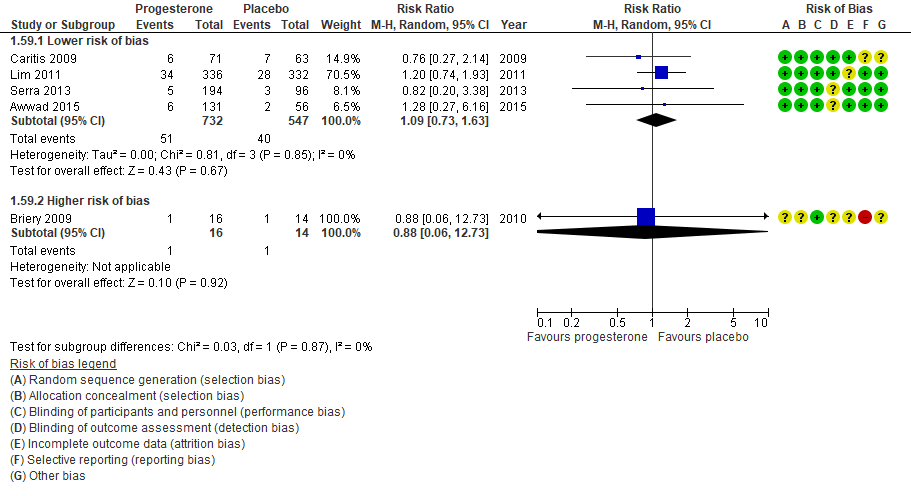


Conclusion: Progesterone compared with placebo probably results in no difference in the risk of PPROM in women with a multifetal pregnancy, neither considering administration route and dosage, nor additional risk factor(s) for preterm birth (GRADE ⊕⊕⊕🌕).

**Progesterone and adverse effects**

No trials or cohort studies reporting on cancer in the woman were identified from the separate search addressing the intervention progesterone and the outcome cancer in women with a multifetal pregnancy.

#

# **Subgroup analyses**

## **Exploratory subgroup analyses based on trials with low risk of bias**

Exploratory subgroup analyses among trials with low risk of bias were performed for administration route (vaginal progesterone or im 17-OHPC), and for specific risk factors (history of preterm birth or short cervical length). Meta-analyses were performed for any preterm birth before 37 and 34 weeks (SFigures 29-33). STable 2 shows the summary estimates from the meta-analyses.

# **STable 2.** Summary estimates from subgroup meta-analyses exploring the effect of progesterone vs placebo in women with a multifetal pregnancy, according to administration route and specific risk factors for preterm birth.

| **Outcomes**  **Administration route**  **Risk factor** | **Number of RCTs**  **(women)** | **Relative effect**  **RR (95% CI)**  In bold if difference is statistically significant  RR <1 indicates favourable outcome of progesterone | **Absolute effect**  **(%)** |
| --- | --- | --- | --- |
| **Administration route** |  |  |  |
| Any PTB <37 weeks |  |  |  |
| 17-OHPC im | 4 (1852) | 1.05 (0.96 to 1.15) | 63.4 vs 59.9 |
| Vaginal progesterone | 6 (2665) | 0.97 (0.87 to 1.08) | 54.5 vs 55.5 |
| Any PTB <34 weeks |  |  |  |
| 17-OHPC im | 6 (2062) | 1.01 (0.89 to 1.14) | 28.8 vs 28.0 |
| Vaginal progesterone | 9 (3195) | 1.03 (0.88 to 1.20) | 18.0 vs 17.8 |
| **Risk factor** |  |  |  |
| Any PTB <37 weeks |  |  |  |
| History of spontaneous preterm birth | NR |  |  |
| Short cervix | 2 (48) | 0.92 (0.71 to 1.20) | 83.9 vs 94.1 |
| Any PTB <34 weeks |  |  |  |
| History of spontaneous preterm birth | 2 (94) | 0.73 (0.36 to 1.50) | 29.5 vs 40.0 |
| Short cervix | 3 (106) | 0.87 (0.62 to 1.22) | 50.0 vs 51.8 |

#

# **SFigure 29.** Outcome: Any preterm birth <37 weeks according to administration route.


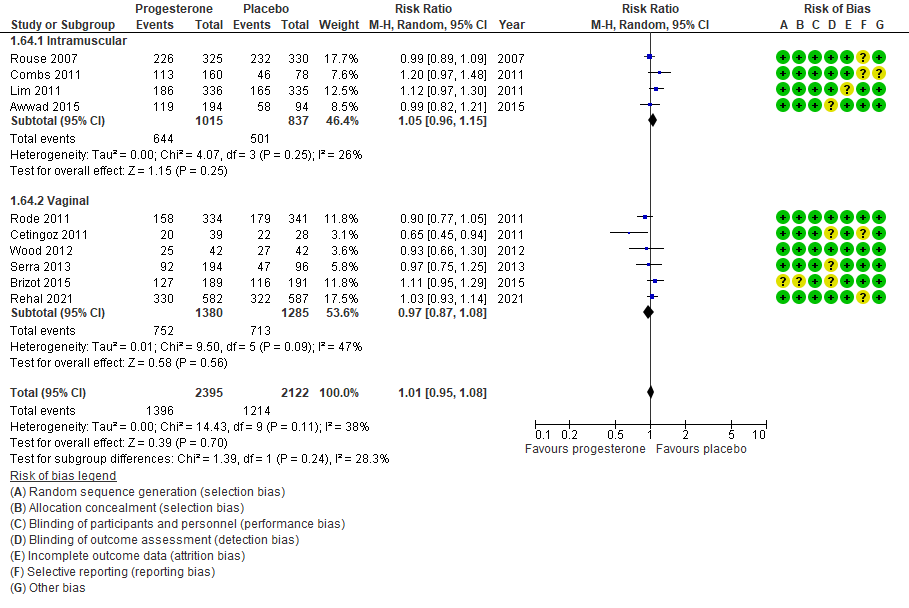


**SFigure 30.** Outcome: Any preterm birth <34 weeks according to administration route.


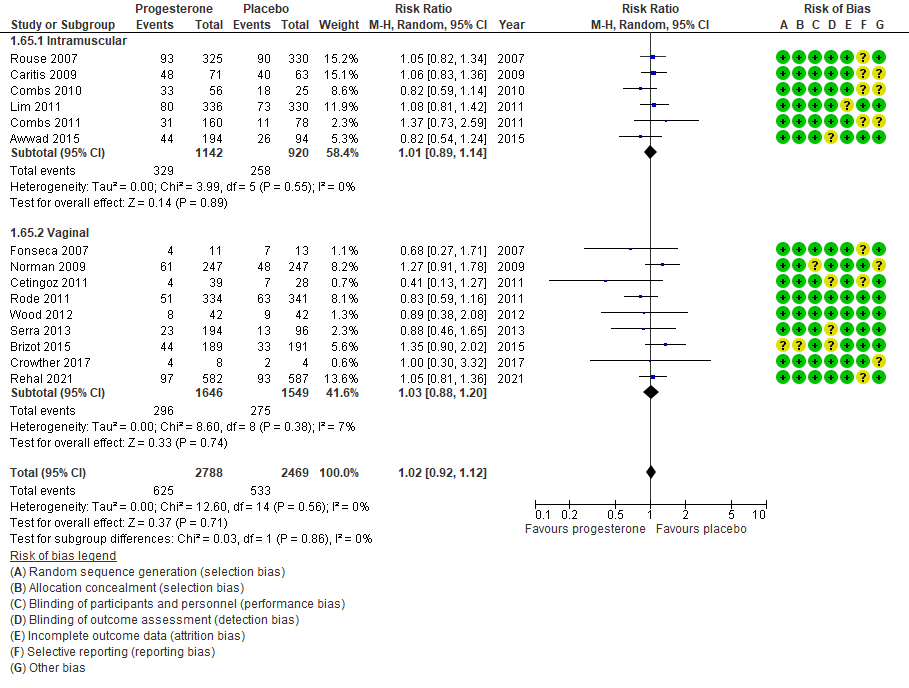


Results from Rouse 2007, Fonseca 2007, Caritis 2009, Lim 2011, Wood 2012, Awwad 2015, Crowther 2017 were retrieved from the IPD meta-analysis by Stewart et al., 2021 (EPPPIC).

**SFigure 31.** Outcome: Any preterm birth <37 weeks in women with short cervical length.


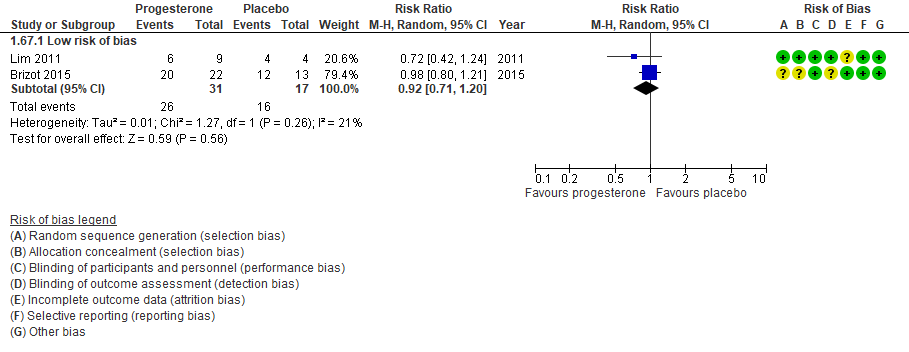


Dosage/administration. Cut-off cervical length: Lim 2011: 250 mg i.m. 17-OHCP/w. <25 mm

Brizot 2015: 200 mg vaginal progesterone/d. ≤25 mm

**SFigure 32.** Outcome: Any preterm birth <34 weeks in women with short cervical length and vaginal progesterone administration.


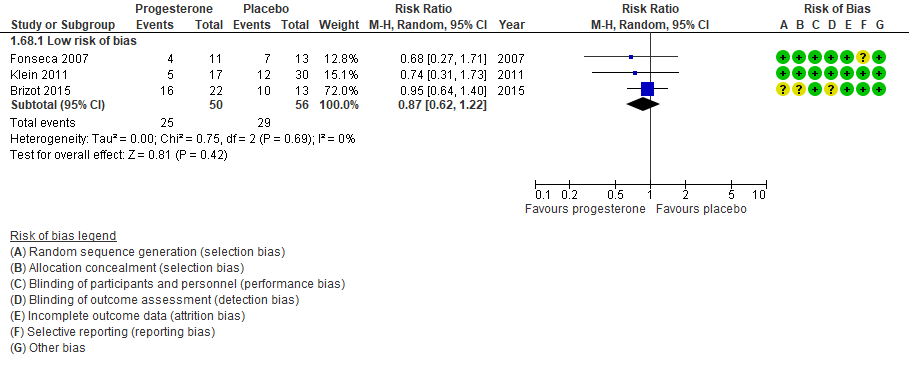


Dosage/administration. 200 mg vaginal progesterone/d in all 3 trials.

Cut-off cervical length: Fonseca 2007: ≤15 mm, Brizot 2015: ≤25 mm, Klein 2011: ≤30 mm

**SFigure 33.** Outcome: Any preterm birth <34 weeks in women with previous preterm birth.


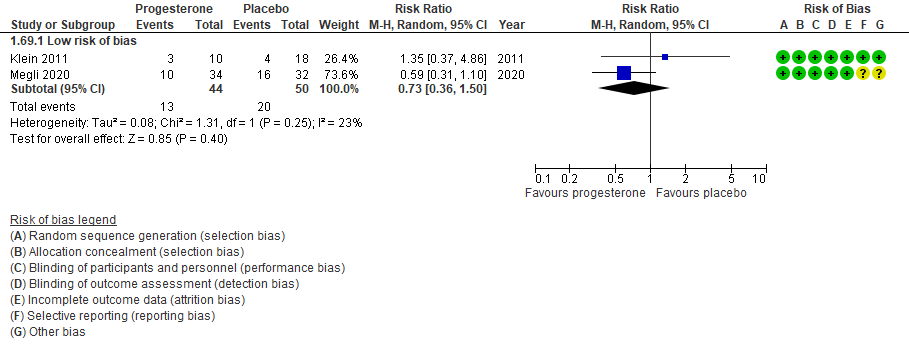


Dosage/administration: Klein 2011: 200 mg vaginal progesterone/d

Megli 2020: 250 mg i.m. 17-OHPC/w
