# Supplementary material for: Progesterone, cerclage, pessary, or acetylsalicylic acid for prevention of preterm birth in singleton and multifetal pregnancies – A systematic review and meta-analyses
Source: Front Med (Lausanne). 2023 Feb 28;10:1111315. doi: 10.3389/fmed.2023.1111315 (PMC10015499; doi:10.3389/fmed.2023.1111315)
Supplement: Supplementary file 1 [file Data_Sheet_1.zip › Data Sheet 1_corrected/Appendix 6.2 Results Cerclage_multifetal.docx]

**Progesterone, cerclage, pessary, or acetylsalicylic acid for prevention of preterm birth in singleton and multifetal pregnancies**

**Appendix 6.2 Results cerclage vs no cerclage in multifetal pregnancies**

**Table of contents**

[**Abbreviations**](#_Abbreviations)**2**

[**STable 1.** Risk of bias legend](#_STable_1._Risk)**3**

[**Results per outcome cerclage vs no cerclage in multifetal pregnancies**](#_Results_per_outcome)**3**

[Preterm birth SFigures 1-9](#_SFigure_1._Outcome:)3-7

[Gestational age and birth weight SFigure 10](#_SFigure_10._Outcome:) 7-8

[Neonatal mortality and morbidity SFigures 11-19](#_SFigure_11._Outcome:) 9-13

[Maternal morbidity SFigures 20-21](#_SFigure_20._Outcome:) 13-14

# **Abbreviations**

BPD bronchopulmonary dysplasia

CI confidence interval

IVH intraventricular hemorrhage

NEC necrotizing enterocolitis

NICU neonatal intensive care unit

PPROM preterm prelabor rupture of membranes

RD risk difference

RDS respiratory distress syndrome

ROP retinopathy of prematurity

RR relative risk/risk ratio

# **STable 1.** **Risk of bias legend to the colour plot within the following forests plots**

1. Random sequence generation (selection bias)
2. Allocation concealment (selection bias)
3. Blinding of participants and personnel (performance bias)
4. Blinding of outcome assessment (detection bias)
5. Incomplete outcome data (attrition bias)
6. Selective reporting (reporting bias)
7. Conflict of interest bias

# **Results per outcome**

**Preterm birth in multifetal pregnancies across gestational weeks**

**Any preterm birth <37 weeks** (Appendix 4.2, STable 4.2.1.a and SFigure 1)

One trial with low risk of bias, including 28 women with a twin pregnancy, showed no difference in the rate of any preterm birth, RR 1.33 (95% CI 0.71 to 2.51). The event rate was 50.0% without cerclage. The RD was 16.7 percentage points (95% CI -19.6 to 52.9).

# **SFigure 1**. Outcome: Any preterm birth before 37 weeks.


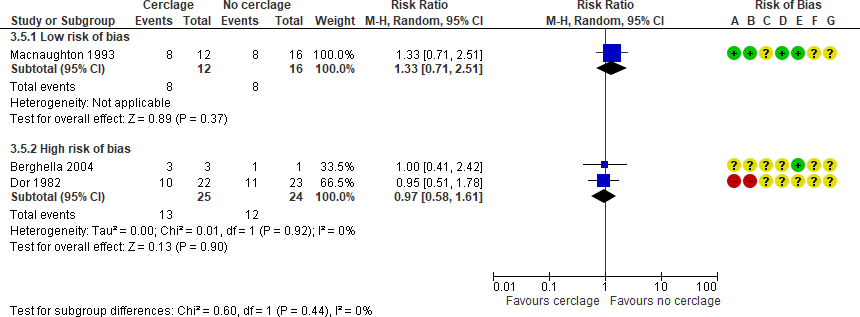


NB: Macnaughton 1993 includes miscarriages in numerator and denominator.

Conclusion: It is uncertain whether cerclage affects the risk of any preterm birth before 37 gestational weeks in women with a twin pregnancy with or without additional risk factor(s) for preterm birth (GRADE ⊕🌕🌕🌕).

**Spontaneous preterm birth <37 weeks**

No trial reported spontaneous preterm birth <37 weeks.

**Any preterm birth <35 weeks** (Appendix 4.2, STable 4.2.2.a and SFigure 2**)**

No trial with low risk of bias reported spontaneous preterm birth <35 weeks.

**SFigure 2**. Outcome: Any preterm birth before 35 weeks.


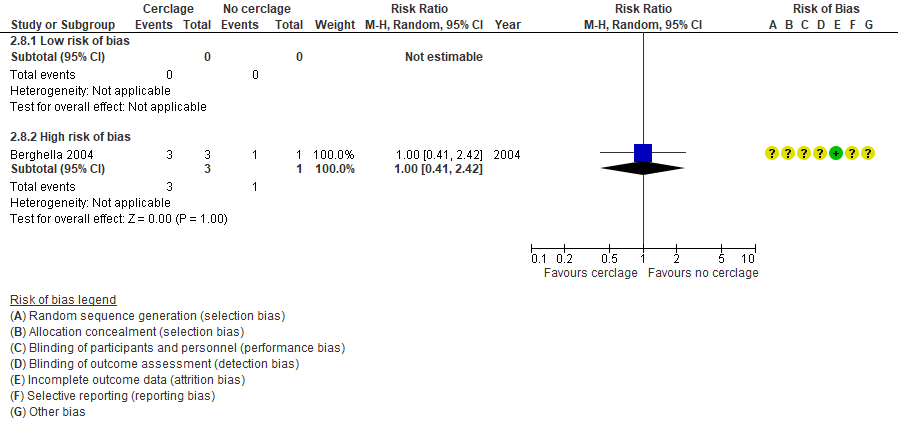


**Spontaneous preterm birth <35 weeks**

No trial reported spontaneous preterm birth <35 weeks.

**Any preterm birth <34 weeks** (Appendix 4.2, STable 4.2.3.a and SFigure 3)

No trial with low risk of bias reported any preterm birth <34 weeks.

**SFigure 3**. Outcome: Any preterm birth before 34 weeks.


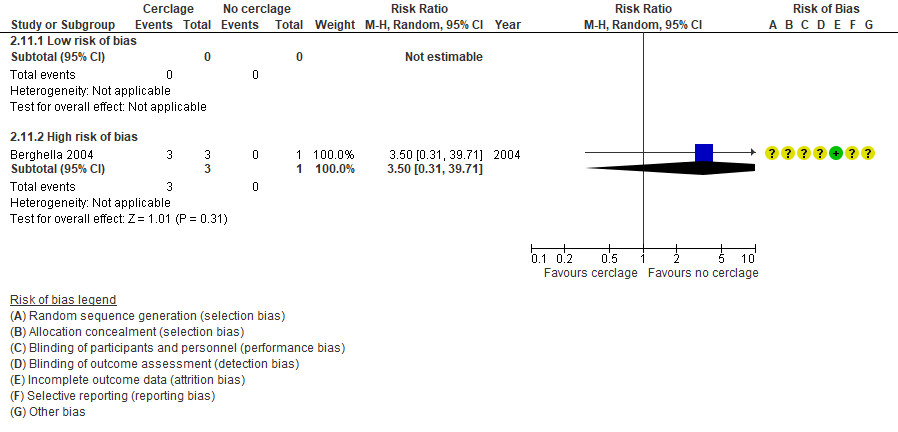


**Spontaneous preterm birth <34 weeks** (Appendix 4.2, STable 4.2.3.b and SFigure 4)

One trial with low risk of bias, including 30 women with a twin pregnancy, showed a reduced rate of spontaneous preterm birth, RR 0.72 (95% CI 0.52 to 0.99). The event rate was 100% without cerclage. The RD was -29.4 percentage points (95% CI -52.8 to -6.0).

**SFigure 4**. Outcome: Spontaneous preterm birth before 34 weeks.


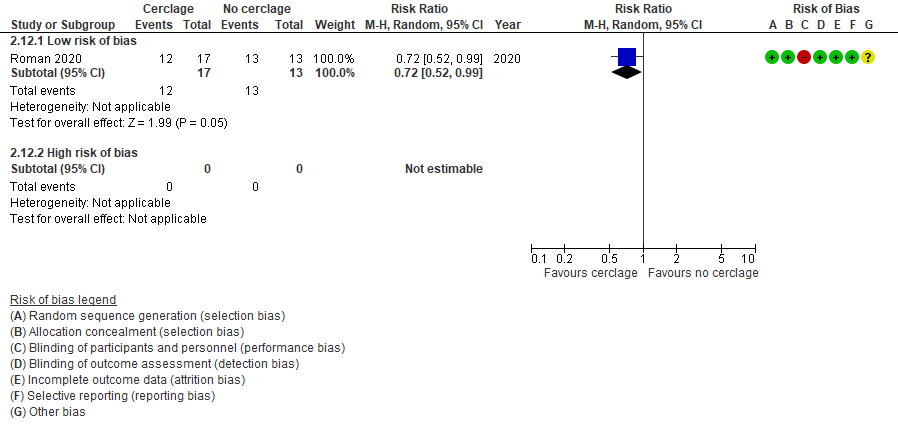


Conclusion: It is uncertain whether cerclage reduces the risk for spontaneous preterm birth before 34 gestational weeks in asymptomatic women with a twin pregnancy, dilated cervix, and visible membranes (GRADE ⊕🌕🌕🌕).

**Any preterm birth <33 weeks** (Appendix 4.2, STable 4.2.4.a and SFigure 5)

One trial with low risk of bias, including 28 women with a twin pregnancy, showed no difference in the rate of any preterm birth, RR 0.27 (95% CI 0.04 to 1.99). The event rate was 31.3% without cerclage. The RD was -22.9 percentage points (95% CI -50.5 to 4.7).

**SFigure 5**. Outcome: Any preterm birth before 33 weeks.


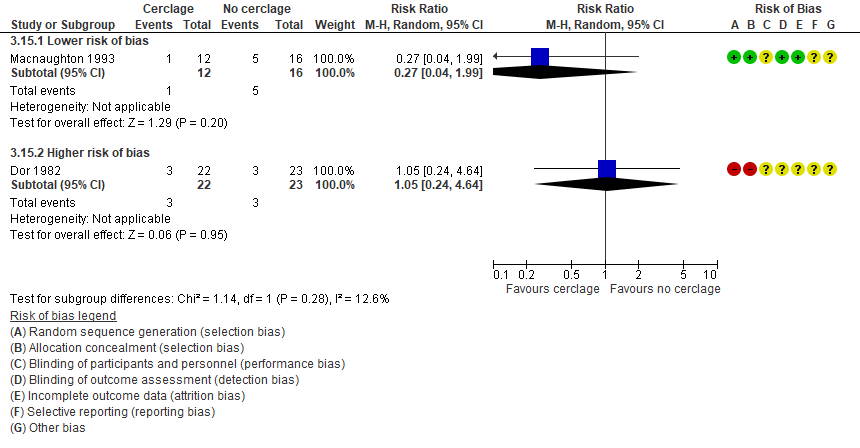


NB: Macnaughton 1993 includes miscarriages in numerator and denominator.

Conclusion: It is uncertain whether cerclage affects the risk of any preterm birth before 33 gestational weeks in women with a twin pregnancy with or without additional risk factor(s) for preterm birth (GRADE ⊕🌕🌕🌕).

**Spontaneous preterm birth <33 weeks**

No trial reported spontaneous preterm birth <33 weeks.

**Any preterm birth <32 weeks** (Appendix 4.2, STable 4.2.5.a and SFigure 6)

No trial with low risk of bias reported any preterm birth <32 weeks.

**SFigure 6**. Outcome: Any preterm birth before 32 weeks.


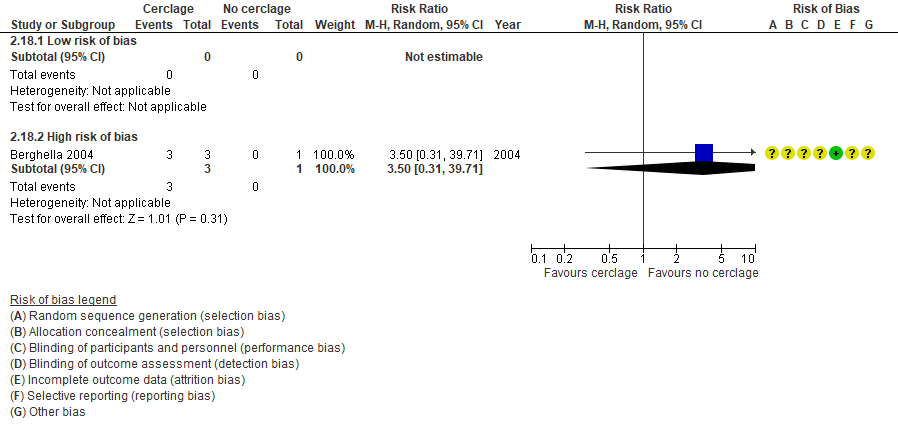


**Spontaneous preterm birth <32 weeks** (Appendix 4.2, STable 4.2.5.b and SFigure 7)

One trial with low risk of bias, including 30 women with a twin pregnancy, showed a reduced rate of spontaneous preterm birth, RR 0.66 (95% CI 0.46 to 0.95). The event rate was 100% without cerclage. The RD was -35.3 percentage points (95% CI -59.2 to -11.1).

**SFigure 7**. Outcome: Spontaneous preterm birth before 32 weeks.


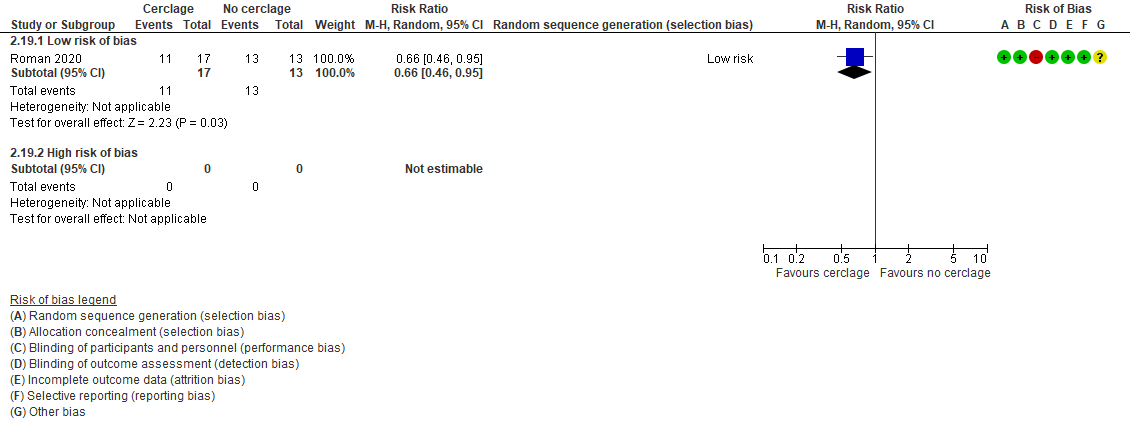


Conclusion: It is uncertain whether cerclage reduces the risk for spontaneous preterm birth before 32 gestational weeks, in asymptomatic women with a twin pregnancy, dilated cervix, and visible membranes (GRADE ⊕🌕🌕🌕).

**Any preterm birth <28 weeks** (Appendix 4.2, STable 4.2.6.a and SFigure 8)

No trial with low risk of bias reported any preterm birth <28 weeks.

**SFigure 8**. Outcome: Any preterm birth before 28 weeks.


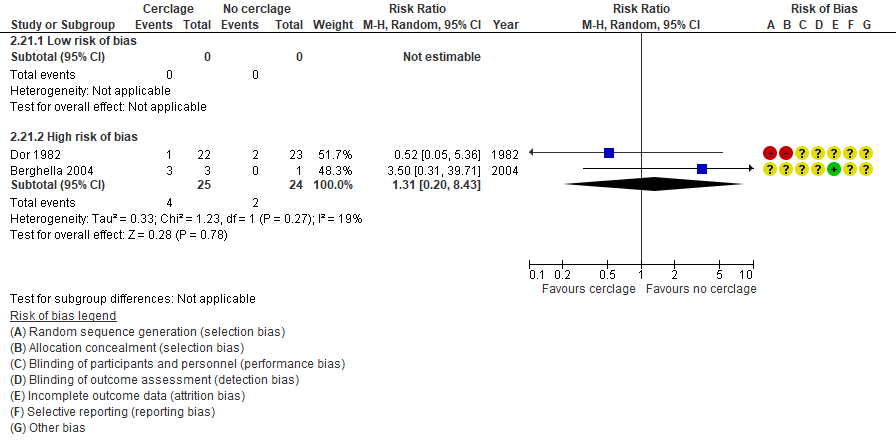


**Spontaneous preterm birth <28 weeks** (Appendix 4.2, STable 4.2.6.b and SFigure 9)

One trial with low risk of bias, including 30 women with a twin pregnancy, showed a reduced rate of spontaneous preterm birth, RR 0.49 (95% CI 0.26 to 0.90). The event rate was 84.6% without cerclage. The RD was -43.4 percentage points (95% CI -74.0 to -12.9).

**SFigure 9**. Outcome: Spontaneous preterm birth before 28 weeks.


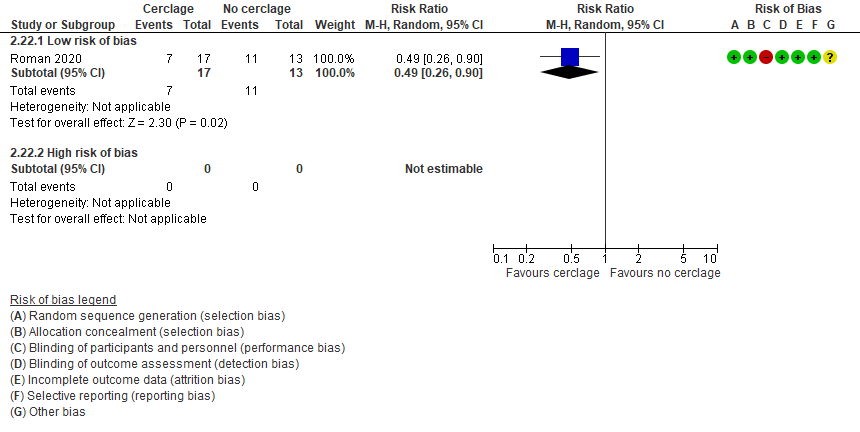


Conclusion: It is uncertain whether cerclage reduces the risk of spontaneous preterm birth before 28 gestational weeks, in asymptomatic women with a twin pregnancy, dilated cervix, and visible membranes (GRADE ⊕🌕🌕🌕).

**Gestational age and birth weight in multifetal pregnancies**

**Gestational age**

No trial reported gestational age.

**Low birth weight**

No trial reported low birth weight.

**Very low birth weight in** (Appendix 4.2, STable 4.2.9 and SFigure 10)

One trial with low risk of bias, including 60 neonates, showed a reduced rate of very low birth weight, RR 0.67 (95% CI 0.50 to 0.89). The event rate was 92.3% without cerclage. The RD

was -30.5 percentage points (95% CI -49.8 to -11.3).

# **SFigure 10**. Outcome: Very low birth weight (<1500g).


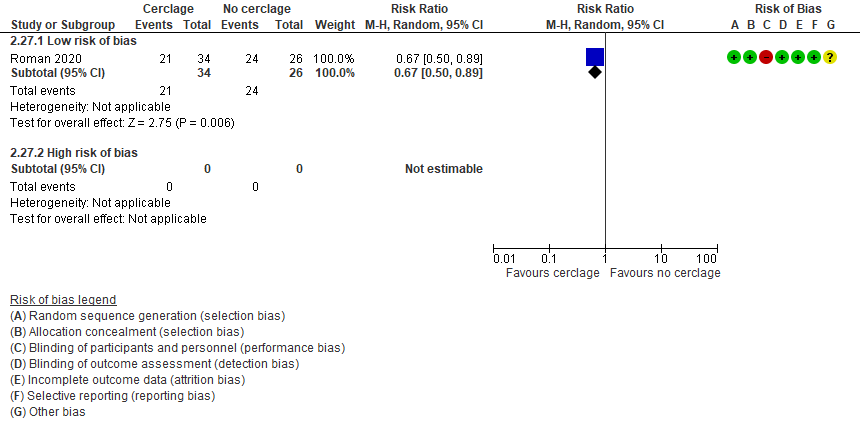


Conclusion: Cerclage may reduce the risk of very low birth weight, in neonates from asymptomatic women with a twin pregnancy, dilated cervix, and visible membranes (GRADE ⊕⊕🌕🌕).

**Mortality and morbidity in neonates from multifetal pregnancies**

**Perinatal mortality** (Appendix 4.2, STable 4.2.10 and SFigure 11)

Two trials with low risk of bias reported perinatal mortality in twin pregnancies. Due to the heterogeneity of the trials, no meta-analysis was performed. The Roman et al. trial, including 60 neonates from women with a twin pregnancy with a very high risk for preterm birth, showed a reduced risk of perinatal mortality, RR 0.23 (95% CI 0.11 to 0.49). The event rate was 76.9% without cerclage. The RD was -59.3 percentage points (95% CI -79.9 to -38.6). There was no intrauterine fetal death. The Macnaughton et al. trial, including 56 neonates, showed no difference in perinatal mortality rate, RR 1.33 (95% CI 0.20 to 8.80). The event rate was 6.3% without cerclage. The RD was 2.1 percentage points (95% CI -11.8 to 16.0).

# **SFigure 11**. Outcome: Perinatal mortality.


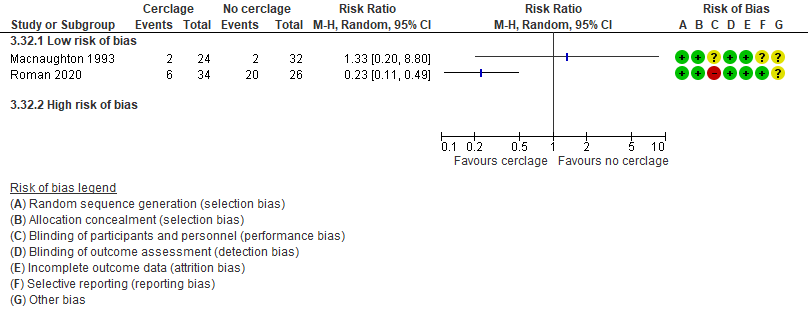


NB: Macnaughton 1993 includes all miscarriages in numerator and denominator.

Conclusions:

-Cerclage may reduce the risk of perinatal mortality in asymptomatic women with twin pregnancies and a dilated cervix, and visible membranes (GRADE ⊕⊕🌕🌕) (based on Roman et al., 2020).

-It is uncertain whether cerclage affects the risk of perinatal mortality in twins with or without additional risk factor(s) for preterm birth (GRADE ⊕🌕🌕🌕) (based on Macnaughton et al., 1993).

**Neonatal mortality <7 days** (Appendix 4.2, STable 4.2.11 and SFigure 12)

No trial with low risk of bias reported on neonatal mortality <7 days.

**SFigure 12**. Outcome: Neonatal mortality <7days in twin pregnancies.


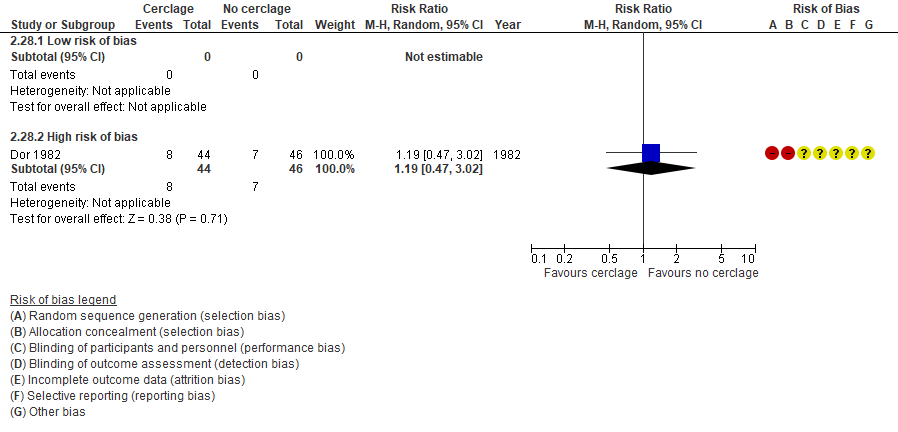


**Neonatal mortality <28 days** (Appendix 4.2, STable 4.2.12 and SFigure 13)

One trial with a low risk of bias, including 60 neonates, showed a reduced rate of neonatal mortality, RR 0.23 (95% CI 0.11 to 0.49). The event rate was 76.9% without cerclage. The RD was -59.3 percentage points (95% CI -79.9 to -38.6).

**SFigure 13**. Outcome: Neonatal mortality <28 days.


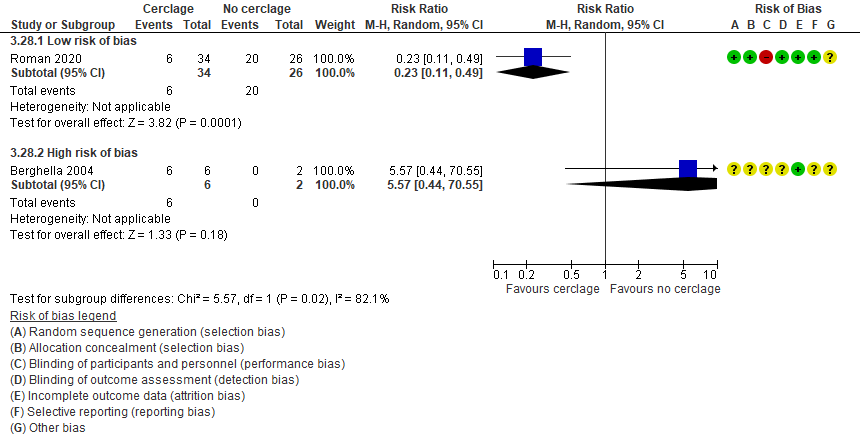


Conclusion: Cerclage may reduce the risk of neonatal mortality <28 days in neonates from asymptomatic women with a twin pregnancy, dilated cervix, and visible membranes (GRADE ⊕⊕🌕🌕).

**Composite adverse neonatal outcome** (Appendix 4.2, STable 4.2.13 and SFigure 14)

One trial with low risk of bias, including 36 neonates, showed no difference in composite adverse neonatal outcome rate, RR 0.93 (95% CI 0.38 to 2.27). Mortality was not included in the composite adverse outcome. The event rate was 50.0% without cerclage. The RD was -3.3 percentage points (95% CI -47.1 to 40.5).

**SFigure 14**. Composite adverse neonatal outcome.


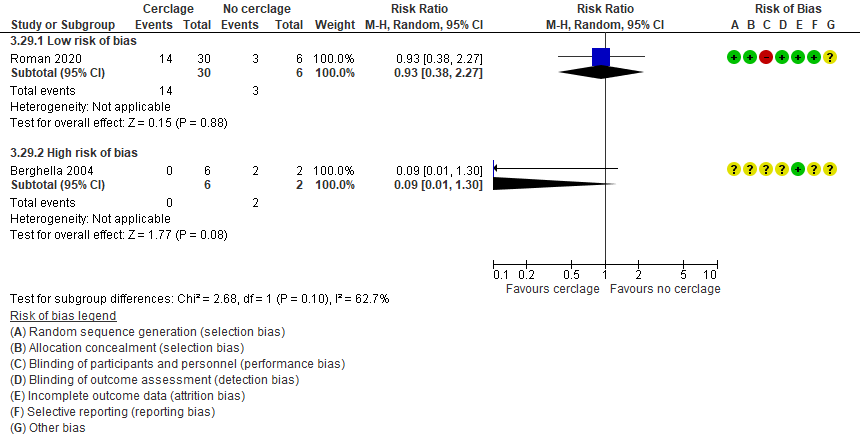


Conclusion: It is uncertain whether cerclage compared with no cerclage, results in a reduced risk of composite adverse neonatal outcome, in neonates from asymptomatic women with a twin pregnancy, dilated cervix, and visible membranes (GRADE ⊕🌕🌕🌕).

**Respiratory distress syndrome (RDS)** (Appendix 4.2, STable 4.2.14 and SFigure 15)

One trial with low risk of bias, including 36 neonates, showed no difference in RDS rate, RR 1.40 (95% CI 0.42 to 4.62). The event rate was 33.3% without cerclage. The RD was 13.3 percentage points (95% CI -28.4 to 55.1).

**SFigure 15**. Outcome: Respiratory distress syndrome.


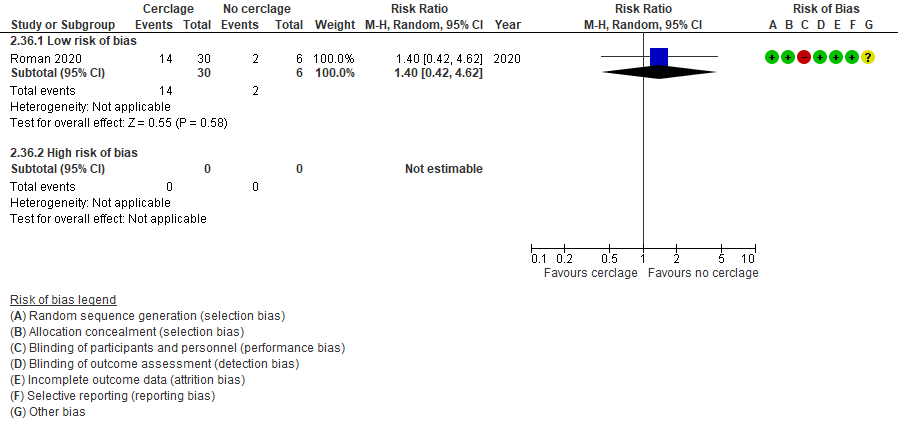


Conclusion: It is uncertain whether cerclage affects the risk of RDS in neonates from asymptomatic women with a twin pregnancy, dilated cervix, and visible membranes (GRADE ⊕🌕🌕🌕).

**Bronchopulmonary dysplasia (BPD)**

No trial reported BPD.

**Intraventricular hemorrhage (IVH)** (Appendix 4.2, STable 4.2.16 and SFigure 16)

One trial with low risk of bias, including 36 neonates, showed no difference in the rate of IVH, RR 0.80 (95% CI 0.11 to 5.96). The event rate was 16.7% without cerclage. The RD was -3.3 percentage points (95% CI -35.5 to 28.9).

**SFigure 16**. Outcome: Intraventricular hemorrhage.


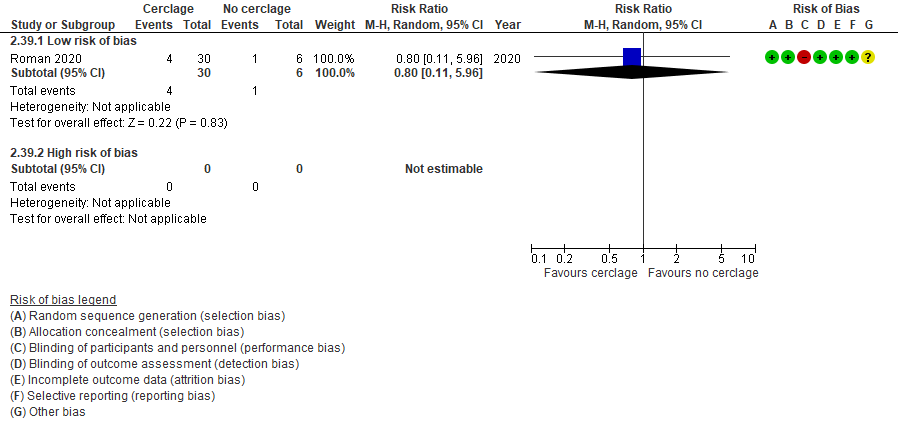


Conclusion: It is uncertain whether cerclage affects the risk of IVH in neonates from asymptomatic women with a twin pregnancy, dilated cervix, and visible membranes (GRADE ⊕🌕🌕🌕).

**Necrotizing enterocolitis (NEC)** (Appendix 4.2, STable 4.2.17)

One trial with low risk of bias, including 36 neonates reported zero events in both groups.

Conclusion: It is uncertain whether cerclage affects the risk of NEC in neonates from asymptomatic women with a twin pregnancy, dilated cervix, and visible membranes (GRADE ⊕🌕🌕🌕).

**Neonatal sepsis** (Appendix 4.2, STable 4.2.18 and SFigure 17)

One trial with low risk of bias, including 36 neonates, showed no difference in the rate of neonatal sepsis, RR 0.40 (95% CI 0.04 to 3.74). The event rate was 16.7% without cerclage. The RD was -10.1 percentage points (95% CI -41.1 to 21.1).

**SFigure 17**. Outcome: Neonatal sepsis.


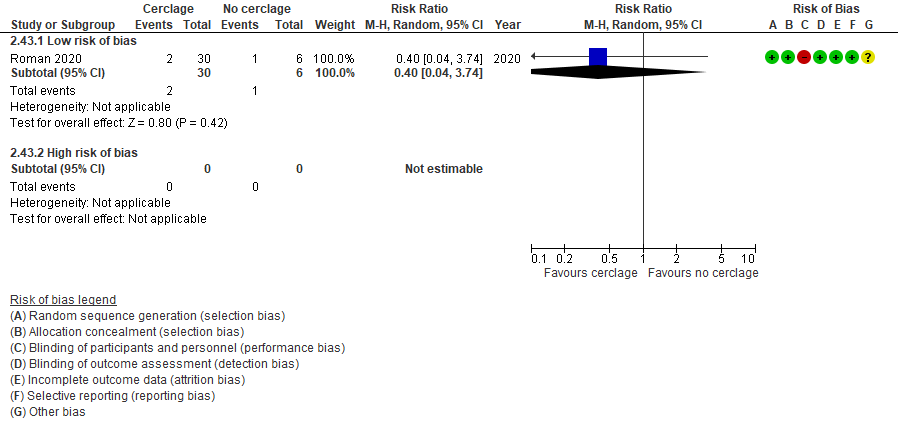


Conclusion: It is uncertain whether cerclage affects the risk of neonatal sepsis, in neonates from asymptomatic women with a twin pregnancy, dilated cervix, and visible membranes (GRADE ⊕🌕🌕🌕).

**Retinopathy of prematurity (ROP)** (Appendix 4.2, STable 4.2.19 and SFigure 18)

One trial with low risk of bias, including 36 neonates, showed no difference in the rate of ROP, RR 1.00 (95% CI 0.14 to 7.10). The event rate was 16.7% without cerclage. The RD was 0.0 percentage points (95% CI -32.7 to 32.7).

**SFigure 18**. Outcome: Retinopathy of prematurity.


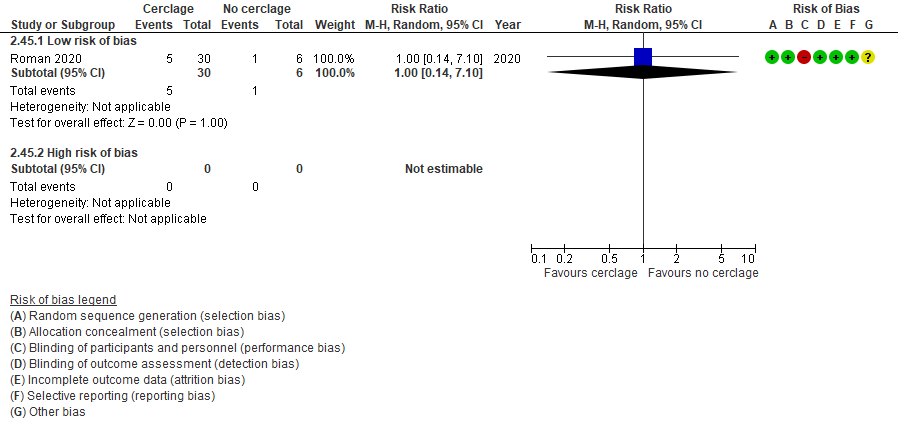


Conclusion: It is uncertain whether cerclage affects the risk of ROP in neonates from asymptomatic women with a twin pregnancy, dilated cervix, and visible membranes (GRADE ⊕🌕🌕🌕).

**Admittance to neonatal intensive care unit** (Appendix 4.2, STable 4.2.20 and SFigure 19)

One trial with a low risk of bias, including 36 neonates, showed no difference in the admittance rate to NICU, RR 0.78 (95% CI 0.58 to 1.05). The event rate was 100% without cerclage. The RD was -27.7 percentage points (95% CI -51.4 to -2.0).

**SFigure 19**. Outcome: Admittance to neonatal intensive care unit.


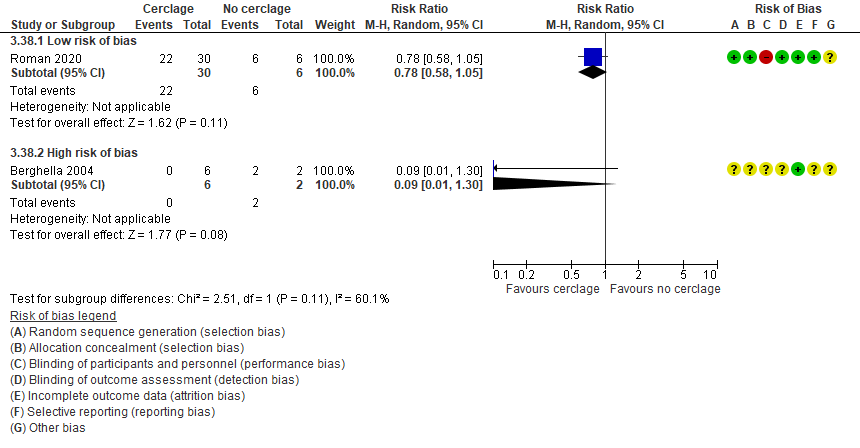


Conclusion: Cerclage compared with no cerclage, may result in no difference in the risk of NICU admittance in neonates from asymptomatic women with a twin pregnancy, dilated cervix, and visible membranes (GRADE ⊕⊕🌕🌕).

**Long-term child outcomes**

No trial reported long-term child outcomes.

**Mortality and morbidity in women with multifetal pregnancies**

**Maternal mortality and morbidity**

No trial reported maternal mortality, hypertensive disorders in pregnancy, gestational diabetes, or intrahepatic cholestasis.

**Infection (clinical chorioamnionitis) (**Appendix 4.2, STable 4.2.21 and SFigure 20)

One trial with low risk of bias, including 30 women with a twin pregnancy, showed no difference in the rate of clinical chorioamnionitis, RR 0.51 (95% CI 0.10 to 2.62). The event rate was 23.1% without cerclage. The RD was -11.3 percentage points (95% CI -38.9 to 16.2).

# **SFigure 20**. Outcome: Chorioamnionitis.


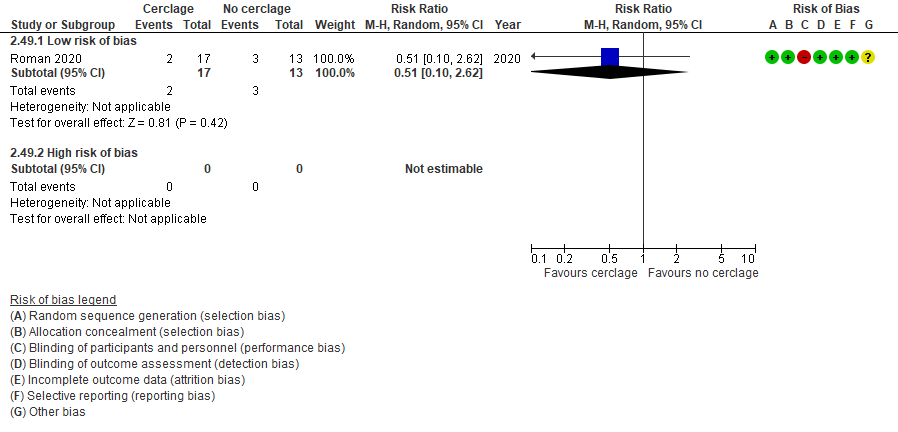


Conclusion: It is uncertain whether cerclage affects the risk of clinical chorioamnionitis in asymptomatic women with a twin pregnancy and dilated cervix, and visible membranes (GRADE ⊕🌕🌕🌕).

**Preterm prelabor rupture of the membranes (PPROM) (**Appendix 4.2, STable 4.2.22 and SFigure 21)

One trial with low risk of bias, including 30 women with a twin pregnancy, showed no difference in the risk of PPROM, RR 1.68 (95% CI 0.78 to 3.64). The event rate was 38.5% without cerclage. The RD was 26.2 percentage points (95% CI -8.6 to 61.1).

**SFigure 21**. Outcome: Preterm prelabor rupture of the membranes (PPROM).


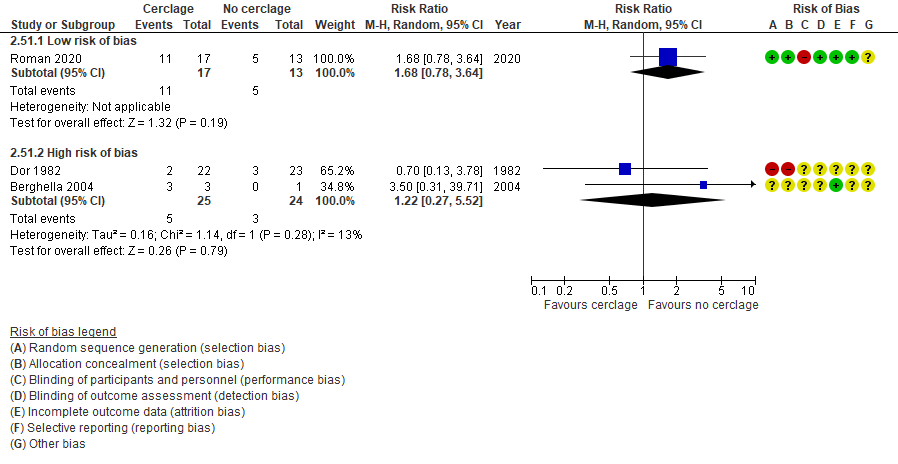


Conclusion: It is uncertain whether cerclage affects the rate of PPROM in asymptomatic women with a twin pregnancy, dilated cervix, and visible membranes (GRADE ⊕🌕🌕🌕).
