# Supplementary material for: Progesterone, cerclage, pessary, or acetylsalicylic acid for prevention of preterm birth in singleton and multifetal pregnancies – A systematic review and meta-analyses
Source: Front Med (Lausanne). 2023 Feb 28;10:1111315. doi: 10.3389/fmed.2023.1111315 (PMC10015499; doi:10.3389/fmed.2023.1111315)
Supplement: Supplementary file 1 [file Data_Sheet_1.zip › Data Sheet 1_corrected/Appendix 6.3 Results Pessary_multifetal.docx]

**Region Västra Götaland, HTA-centrum**

**Regional**

**HTA report 2022:129**

**Progesterone, cerclage, pessary, or acetylsalicylic acid for prevention of preterm birth in singleton and multifetal pregnancies**

**Appendix 6.3 Results pessary vs no pessary in multifetal pregnancies**

**Table of contents**

[Abbreviations1](#_Abbreviations)

[STable 1. Risk of bias legend2](#_STable_1._Risk)

[Results per outcome pessary vs no pessary in multifetal pregnancies2](#_Results_per_outcome)

[Preterm birth SFigures 1-7 2-5](#_SFigure_1._Outcome:)

[Gestational age and birth weight SFigures 8-10 5-6](#_SFigure_8._Outcome:)

Neonatal mortality and morbidity SFigures 11-20 6-11

[Maternal mortality and morbidity SFigures 21-24 11-1](#_SFigure_21._Outcome:)3

[Subgroup analyses 1](#_Subgroup_analyses)4

[Preterm birth SFigures 25-261](#_SFigure_25._Outcome:)4

# Abbreviations

BPD bronchopulmonary dysplasia

CDI child developmental inventory

CI confidence interval

HDP hypertensive disorders in pregnancy

IVH intraventricular haemorrhage

mm millimetre

NEC necrotizing enterocolitis

NICU neonatal intensive care unit

PPROM preterm prelabor rupture of membranes

RD risk difference

RDS respiratory distress syndrome

ROP retinopathy of prematurity

RR relative risk/risk ratio

# STable 1. Risk of bias legend to the colour plot within the following forests plots

1. Random sequence generation (selection bias)
2. Allocation concealment (selection bias)
3. Blinding of participants and personnel (performance bias)
4. Blinding of outcome assessment (detection bias)
5. Incomplete outcome data (attrition bias)
6. Selective reporting (reporting bias)
7. Conflict of interest bias

# Results per outcome

**Preterm birth in multifetal pregnancies across gestational weeks**

**Any preterm birth <37 weeks** (Appendix 4.3, STable 4.3.1.a and SFigure 1)

A meta-analysis of four trials, including 1428 women, showed no difference in the rate of any preterm birth, RR 0.97 (95% CI 0.89 to 1.04). The crude event rate across trials was 61.6% without pessary. The pooled weighted RD was -2.0% percentage points (95% CI -7.0 to 2.9).

# SFigure 1. Outcome: Any preterm birth <37 weeks.

**
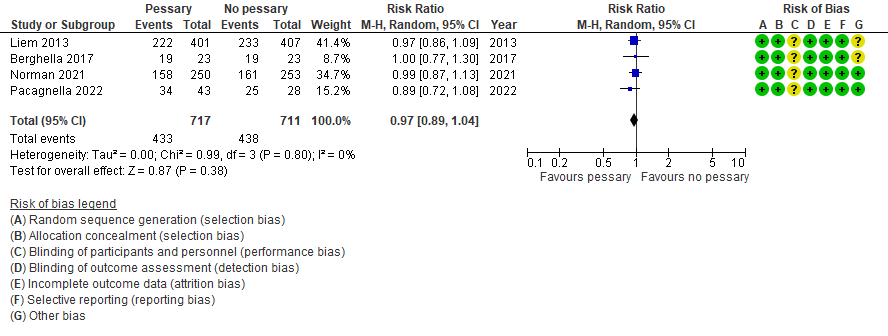
**

Conclusion: Pessary compared with no pessary probably results in no difference in the risk of any preterm birth before 37 gestational weeks in women with a multifetal pregnancy, not considering additional risk factor(s) for preterm birth (GRADE ⊕⊕⊕🌕 ).

**Spontaneous preterm birth <37 weeks** (Appendix 4.3, STable 4.3.1.b and SFigure 2)

A meta-analysis of three trials, including 683 women with short cervical length, showed no difference in the rate of spontaneous preterm birth, RR 0.93 (95% CI 0.78 to 1.10). The crude event rate across trials was 36.8% without pessary. The pooled weighted RD was -3.5 % percentage points (95% CI-10.0 to 3.1).

**SFigure 2.** Outcome: Spontaneous preterm birth <37 weeks.


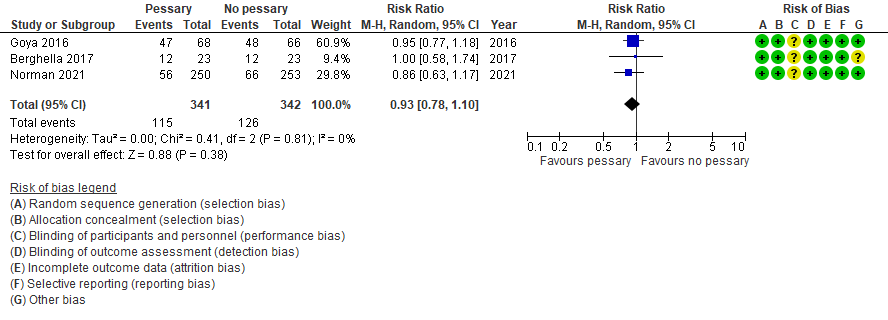


Conclusion: Pessary compared with no pessary probably results in no difference in the risk of spontaneous preterm birth before 37 gestational weeks in women with a twin pregnancy and short cervical length (GRADE ⊕⊕⊕🌕 ).

**Any preterm birth <35 weeks**

No trial reported spontaneous preterm birth <35 weeks.

**Spontaneous preterm birth <35 weeks**

No trial reported spontaneous preterm birth <35 weeks.

**Any preterm birth <34 weeks** (Appendix 4.3, STable 4.3.2.a and SFigure 3)

A meta-analysis of five trials, including 1931 women, showed no difference in the rate of any preterm birth, RR 0.86 (95% CI 0.65 to 1.15). The crude event rate across trials was 21.4% without pessary. The pooled weighted RD was -4.7 % percentage points (95% CI -12.8 to 3.5).

**SFigure 3.** Outcome: Any preterm birth <34 weeks.


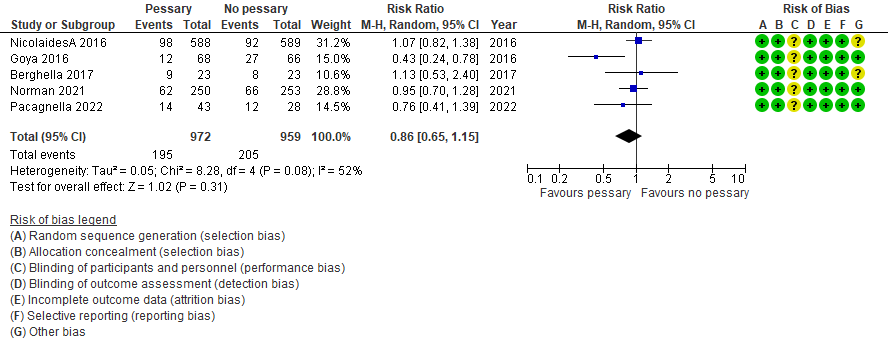


Conclusion: Pessary compared with no pessary may result in no difference in the risk of any preterm birth before 34 gestational weeks, in women with a twin pregnancy, not considering additional risk factor(s) for preterm birth (GRADE ⊕⊕🌕 🌕).

**Spontaneous preterm birth <34 weeks** (Appendix 4.3, STable 4.3.2.b and SFigure 4)

A meta-analysis of four trials, including 1860 women, showed no difference in the rate of spontaneous preterm birth RR 0.80 (95% CI 0.54 to 1.17). The crude event rate across trials was 16.6% without pessary. The pooled weighted RD was -5.2 % percentage points (95% CI -13.5 to 3.2).

**SFigure 4.** Outcome: Spontaneous preterm birth <34 weeks.


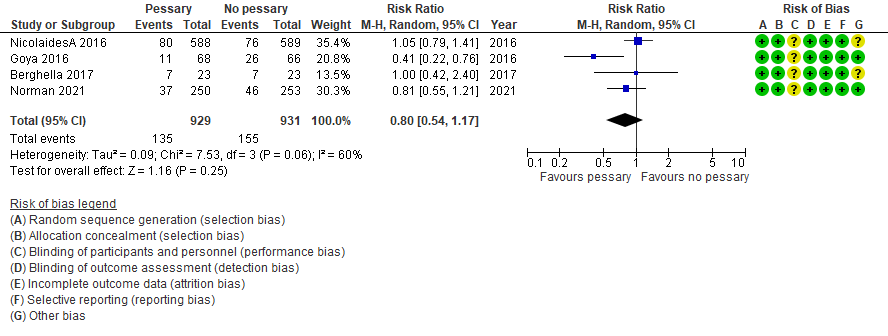


Conclusion: Pessary compared with no pessary may result in no difference in the risk of spontaneous preterm birth before 34 gestational weeks, in women with a twin pregnancy, not considering additional risk factor(s) for preterm birth (GRADE ⊕⊕🌕 🌕).

**Any preterm birth <33 weeks**

No trial reported any preterm birth <33 weeks.

**Spontaneous preterm birth <33 weeks**

No trial reported spontaneous preterm birth <33 weeks.

**Any preterm birth <32 weeks** (Appendix 4.3, STable 4.3.3.a and SFigure 5)

A meta-analysis of four trials, including 2559 women, showed no difference in the rate of any preterm birth, RR 0.85 (95% CI 0.67 to 1.09). The crude event rate across trials was 11.9% without pessary. The pooled weighted RD was -1.6 % percentage points (95% CI -4.9 to 1.6).

**SFigure 5.** Outcome: Any preterm birth <32 weeks.

**
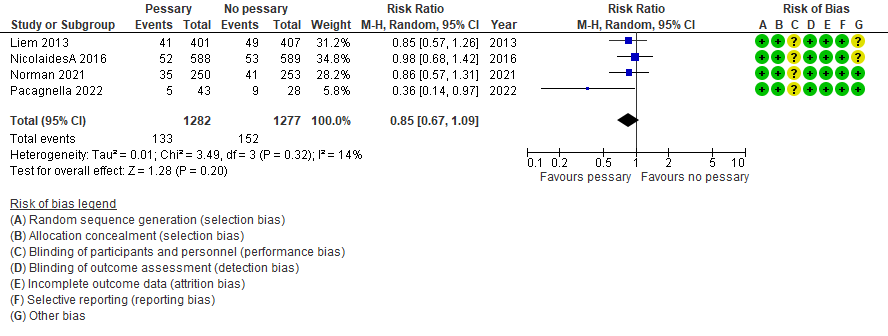
**

Conclusion: Pessary compared with no pessary probably results in no difference in the risk of any preterm birth before 32 gestational weeks, in women with a multifetal pregnancy, not considering additional risk factor(s) for preterm birth (GRADE ⊕⊕⊕🌕 ).

**Spontaneous preterm birth <32 weeks**(Appendix 4.3, STable 4.3.3.b)

One trial, including 503 women, showed no difference in the rate of spontaneous preterm birth, RR 0.82 (95% CI 0.51 to 1.33). The crude event rate was 12.6% without pessary. The RD was -2.3 percentage points (95% CI -7.8 to 3.3).

Conclusion: Pessary compared with no pessary may result in no difference in the risk of spontaneous preterm birth before 32 gestational weeks, in women with a multifetal pregnancy with a short cervical length (GRADE ⊕⊕🌕 🌕).

**Any preterm birth <28 weeks (**Appendix 4.3, STable 4.3.4.a and SFigure 6)

A meta-analysis of five trials, including 2605 women, showed no difference in the rate of any preterm birth, RR 0.79 (95% CI 0.52 to 1.22). The crude event rate across trials was 5.5% without pessary. The pooled weighted RD was -1.3 % percentage points (95% CI -4.3 to 1.7).

**SFigure 6.** Outcome: Any preterm birth <28 weeks.

**
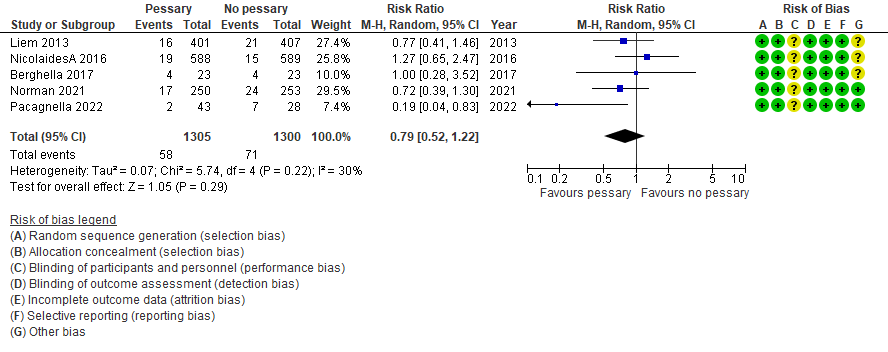
**

Conclusion: Pessary compared with no pessary may result in no difference in the risk of any preterm birth before 28 gestational weeks, in women with a multifetal pregnancy, not considering additional risk factor(s) for preterm birth (GRADE ⊕⊕🌕 🌕).

**Spontaneous birth <28 weeks (**Appendix 4.3, STable 4.3.4.b and SFigure 7)

A meta-analysis of three trials, including 683 women with short cervical length, showed no difference in the rate of spontaneous preterm birth, RR 0.67 (95% CI 0.39 to 1.13). The crude event rate across trials was 9.4% without pessary. The pooled weighted RD was -3.1 % percentage points (95% CI -6.9 to 0.8).

**SFigure 7.** Outcome: Spontaneous preterm birth <28 weeks.


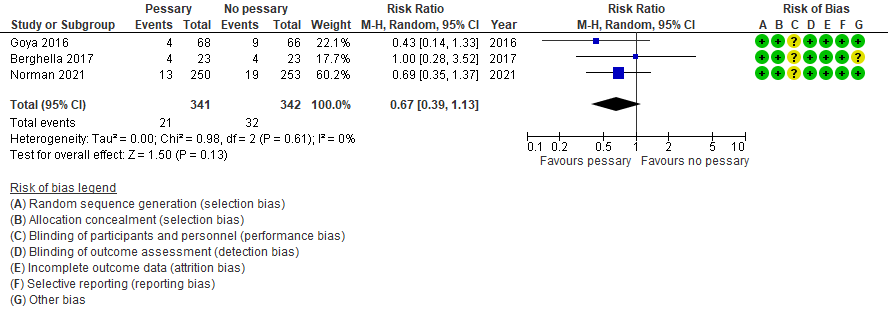


Conclusion: Pessary compared with no pessary may result in no difference in the risk of spontaneous preterm birth before 28 gestational weeks in twins and short cervical length (GRADE ⊕⊕🌕 🌕).

**Gestational age and birth weight in multifetal pregnancies**

**Gestational age (**Appendix 4.3, STable 4.3.5 and SFigure 8)

A meta-analysis of five trials, including 2656 women showed no mean difference in gestational age, 0.57 (-0.04 to 1.18) weeks, corresponding to approximately four days longer (0.3 day less to eight days longer) gestational length in the pessary group.

# SFigure 8. Outcome: Gestational age at delivery (weeks).


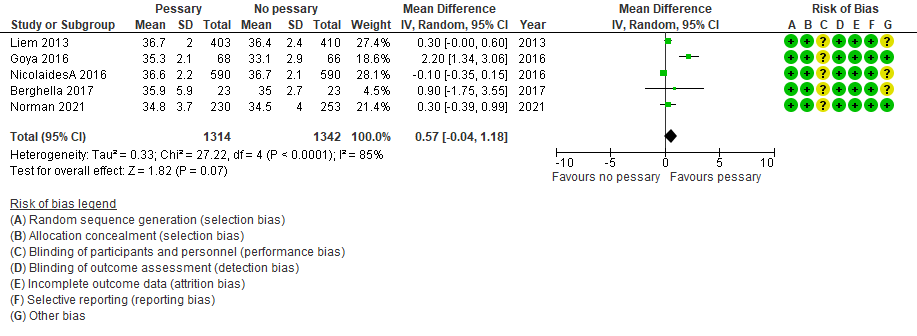


Conclusion: Pessary compared with no pessary probably results in no difference in length of gestation in women with a multifetal pregnancy, not considering additional risk factor(s) for preterm birth (GRADE ⊕⊕⊕🌕 ).

**Low birth weight (**Appendix 4.3, STable 4.3.6 and SFigure 9)

A meta-analysis of three trials, including 4254 women showed no difference in the rate of low birth weight, RR 0.95 (95% CI 0.87 to 1.05). The crude event rate across trials was 56.2% without pessary. The pooled weighted RD was -2.8 % percentage points (95% CI -7.6 to 2.0).

**SFigure 39.** Outcome: Low birth weight (<2500 g).


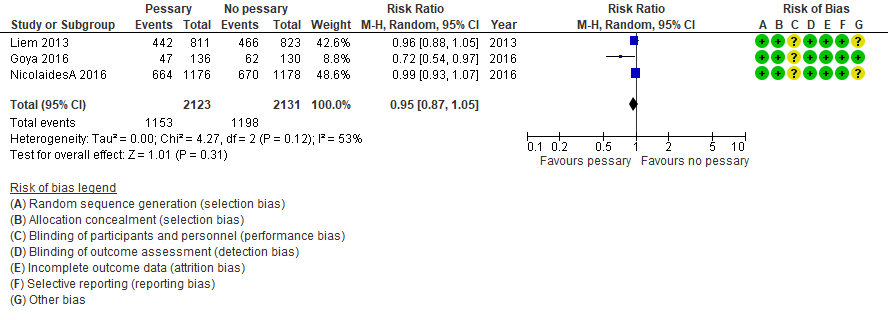


Conclusion: Pessary compared with no pessary probably results in no difference in the risk of low birth weight (<2500g) in neonates from a twin pregnancy, not considering additional risk factor(s) for preterm birth (GRADE ⊕⊕⊕🌕 ).

**Very low birth weight (**Appendix 4.3, STable 4.3.7 and SFigure 10)

A meta-analysis of three trials, including 4254 women showed no difference in the rate of low birth weight, RR 0.98 (95% CI 0.81 to 1.19). The crude event rate across trials was 9.3% without pessary. The pooled weighted RD was -0.1 % percentage points (95% CI -1.8 to 1.7).

**SFigure 10.** Outcome: Very low birth weight (<1500 g).

**
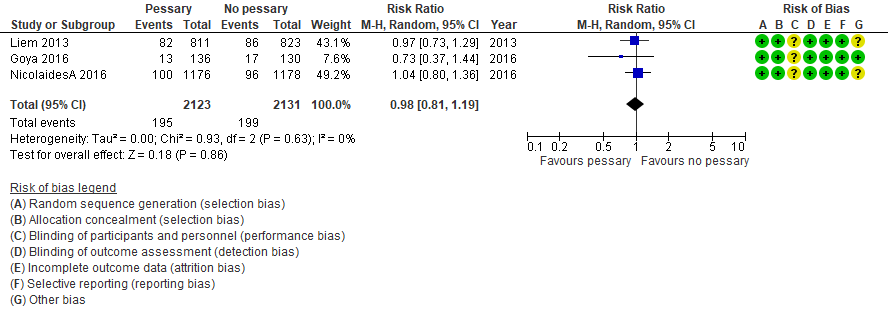
**

Conclusion: Pessary compared with no pessary probably results in no difference in the risk of very low birth weight (<1500g) in neonates from a twin pregnancy, not considering additional risk factor(s) for preterm birth (GRADE ⊕⊕⊕🌕 ).

**Mortality and morbidity in neonates from multifetal pregnancies**

**Perinatal mortality (**Appendix 4.3, STable 4.3.8 and SFigure 11)

A meta-analysis of two trials, including 2183 neonates, showed no difference in the rate of perinatal mortality, RR 0.81 (95% CI 0.48 to 1.38). The definition of perinatal mortality includes intrauterine death and neonatal mortality. Intrauterine death and neonatal mortality were not defined in Nicolaides et al. (2016a), but in Norman et al. (2021) defined as fetal and neonatal death within 28 days. The crude event rate across trials was 2.7% without pessary. The pooled weighted RD was -0.7 % percentage points (95% CI -1.8 to 0.5).

**SFigure 11**. Outcome: Perinatal mortality.


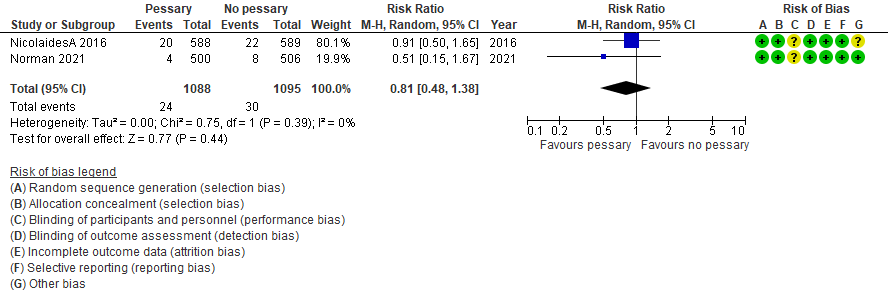


Conclusion: Pessary compared with no pessary may result in no difference in perinatal mortality in perinates from a twin pregnancy, not considering additional maternal risk factor(s) for preterm birth (GRADE ⊕⊕🌕 🌕).

**Neonatal mortality (**Appendix 4.3, STable 4.3.9 and SFigure 12)

A meta-analysis of four trials, including 4346 neonates, showed no difference in the rate of neonatal mortality <28 days, RR 0.99 (95% CI 0.65 to 1.49). The crude event rate across trials was 2.1% without pessary. The pooled weighted RD was 0.1 % percentage points (95% CI -0.6 to 0.8).

**SFigure 12.** Outcome: Neonatal mortality <28 days.


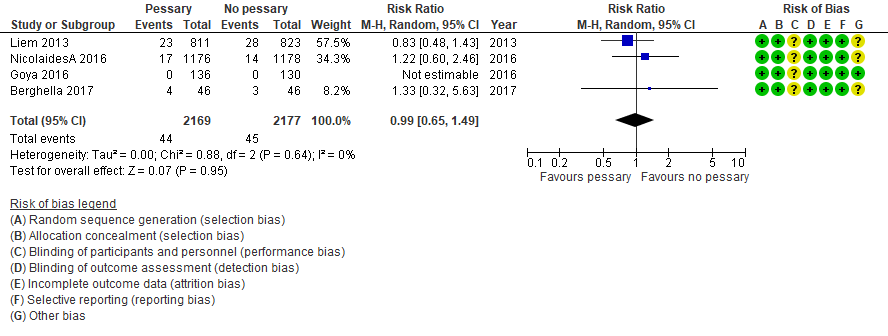


Conclusion: Pessary compared to no pessary may result in no difference in neonatal mortality <28 days in neonates from a multifetal pregnancy, not considering additional maternal risk factor(s) for preterm birth (GRADE ⊕⊕🌕 🌕).

**Composite adverse neonatal outcome (**Appendix 4.3, STable 4.3.10 and SFigure 13)

A meta-analysis of five trials, including 5291 neonates showed no difference in the rate of composite neonatal morbidity, RR 1.01 (95% CI 0.84 to 1.21). The crude event rate across trials was 12.8% without pessary. The pooled weighted RD was -0.3 % percentage points (95% CI -2.4 to 1.9).

A sensitivity analysis of two trials with low risk of bias, including 2559 neonates showed no difference in the rate of composite adverse neonatal morbidity when excluding trials including neonatal mortality, RR of 0.98 (95% CI 0.64 to 1.50).

The composite adverse neonatal outcome included any of intrauterine fetal death, neonatal death, intraventricular haemorrhage, periventricular leukomalacia, necrotizing enterocolitis, bronchopulmonary dysplasia, respiratory distress syndrome, retinopathy of prematurity, or confirmed sepsis.

**SFigure 13.** Outcome: Composite adverse neonatal outcome with or without mortality.


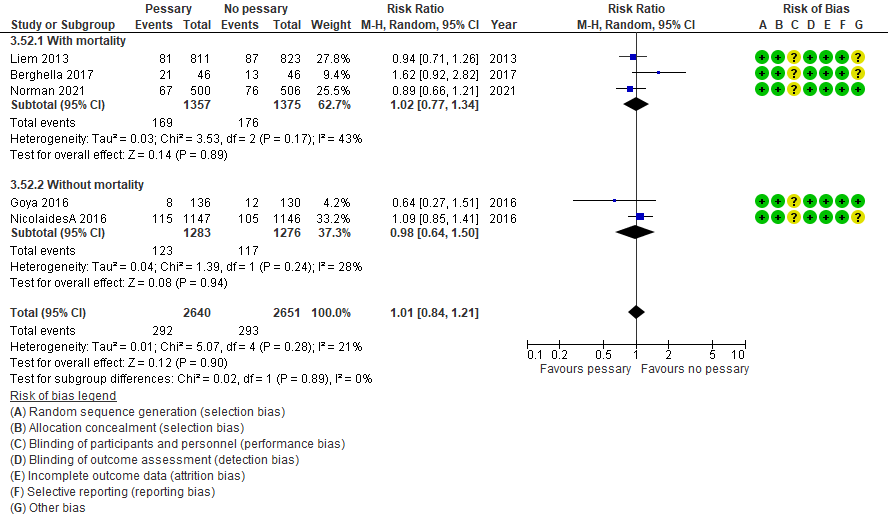


Conclusion: Pessary, compared with no pessary probably results in no difference in a composite adverse neonatal outcome in neonates from a multifetal pregnancy, not considering additional maternal risk factor(s) for preterm birth (GRADE ⊕⊕⊕🌕 ).

**Respiratory distress syndrome (RDS) (**Appendix 4.3, STable 4.3.11 and SFigure 14)

A meta-analysis of four trials, including 4285 neonates showed no difference in the rate of RDS, RR of 1.13 (95% CI 0.91 to 1.40). The crude event rate across trials was 6.8% without pessary. The pooled weighted RD was 0.8 % percentage points (95% CI -0.6 to 2.3).

**SFigure 14.** Outcome: Respiratory distress syndrome

**
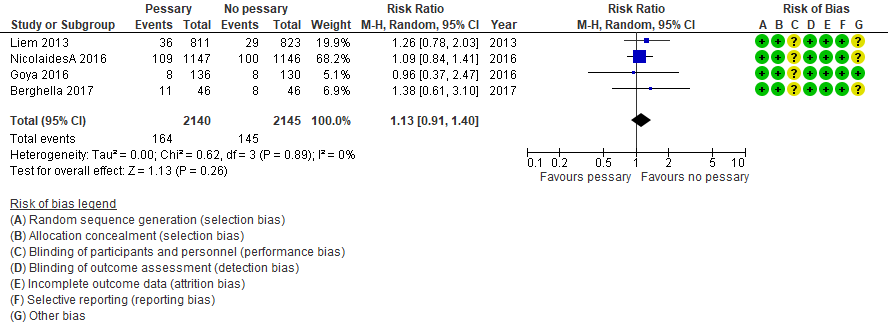
**

Conclusion: Pessary, compared with no pessary probably results in no difference in RDS in neonates from a multifetal pregnancy, not considering additional maternal risk factor(s) for preterm birth (GRADE ⊕⊕⊕🌕 ).

**Bronchopulmonary dysplasia (BPD) (**Appendix 4.3, STable 4.3.12 and SFigure 15)

A meta-analysis of three trials, including 2732 neonates showed no difference in the rate of BPD, RR of 0.74 (95% CI 0.23 to 2.43). The crude event rate across trials was 1.2% without pessary. The pooled weighted RD was -0.2 % percentage points (95% CI -1.5 to 1.0).

**SFigure 15.** Outcome: Bronchopulmonary dysplasia.

**
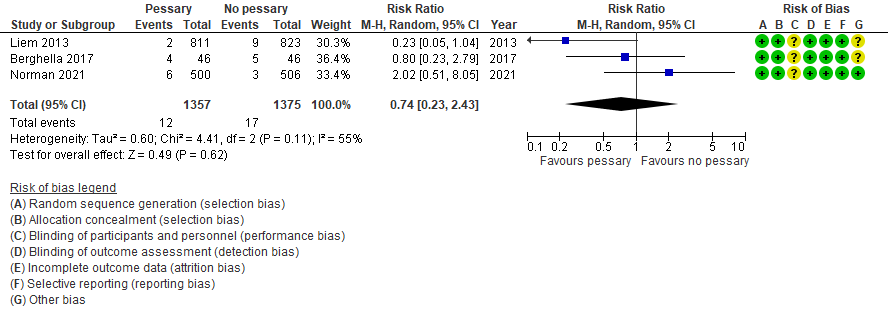
**

Conclusion: Pessary, compared with no pessary may result in no difference in BPD in neonates from a multifetal pregnancy, not considering additional maternal risk factor(s) for preterm birth (GRADE ⊕⊕🌕 🌕).

**Intraventricular hemorrhage (IVH) (**Appendix 4.3, STable 4.3.13 and SFigure 16)

A meta-analysis of five trials, including 5291 women showed no difference in the rate of IVH, RR 1.20 (0.74 to 1.93). The crude event rate across trials was 1.2% without pessary. The pooled weighted RD was 0.2 % percentage points (95% CI -0.5 to 0.8).

**SFigure 16.** Outcome: Intraventricular hemorrhage.

**
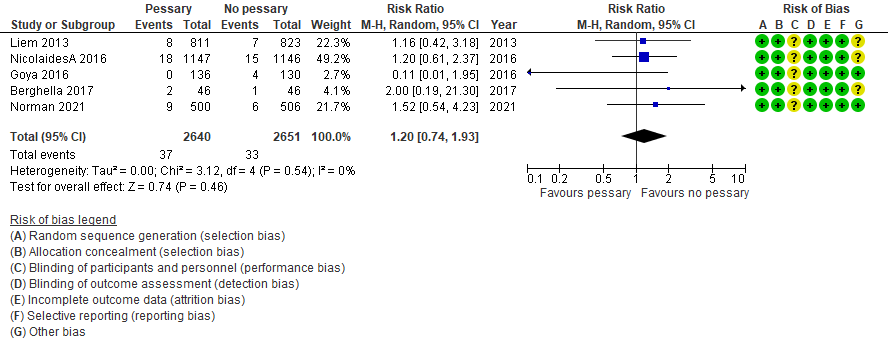
**

Conclusion: Pessary, compared with no pessary may result in no difference in IVH in neonates from a multifetal pregnancy, not considering additional maternal risk factor(s) for preterm birth

(GRADE ⊕⊕🌕 🌕).

**Necrotizing enterocolitis (NEC) (**Appendix 4.3, STable 4.3.14 and SFigure 17)

A meta-analysis of five trials, including 5291 neonates showed no difference in the rate of NEC, RR 0.78 (95% CI 0.33 to 1.86). The crude event rate across trials was 0.9% without pessary. The pooled weighted RD was -0.3 % percentage points (95% CI -1.2 to 0.5).

**SFigure 17.** Outcome: Necrotizing enterocolitis.

**
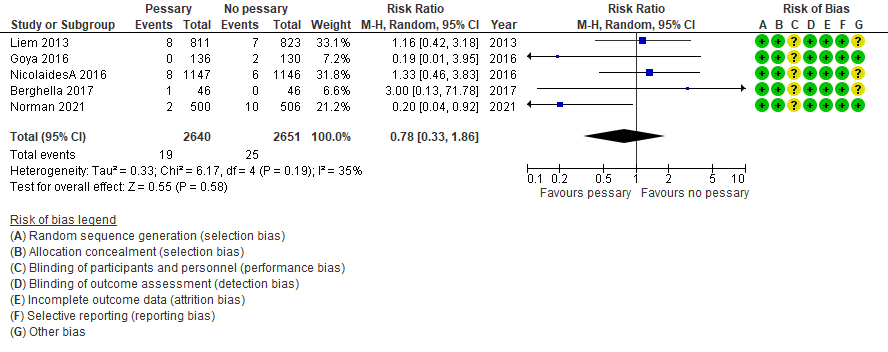
**

Conclusion: Pessary, compared with no pessary may result in no difference in the rate of NEC in neonates from a multifetal pregnancy, not considering additional maternal risk factor(s) for preterm birth (GRADE ⊕⊕🌕 🌕).

**Neonatal sepsis (**Appendix 4.3, STable 4.3.15 and SFigure 18)

A meta-analysis of five trials, including 5291 neonates showed no difference in the rate of neonatal sepsis, RR 1.00 (95% CI 0.73 to 1.37). The crude event rate across trials was 3.9% without pessary. The pooled weighted RD was 0.1 % percentage points (95% CI -0.9 to 1.2).

**SFigure 18.** Outcome: Neonatal sepsis.

**
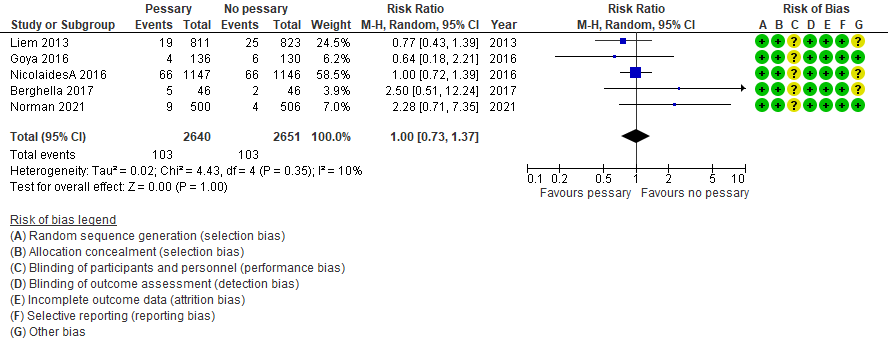
**

Conclusion: Pessary, compared with no pessary probably result in no difference in neonatal sepsis in neonates from a multifetal pregnancy, not considering additional maternal risk factor(s) for preterm birth (GRADE ⊕⊕⊕🌕 ).

**Retinopathy of prematurity (ROP) (**Appendix 4.3, STable 4.3.16 and SFigure 19)

A meta-analysis of three trials, including 2651 neonates showed an increase in the rate of ROP in pessary users, 3.84 (95% CI 1.19 to 12.42). The crude event rate across trials was 0.2% without pessary. The pooled weighted RD was 0.7 % percentage points (95% CI 0.1 to 1.3). Very serious imprecision affected certainty of evidence.

**SFigure 19.** Outcome: Retinopathy of prematurity.

**
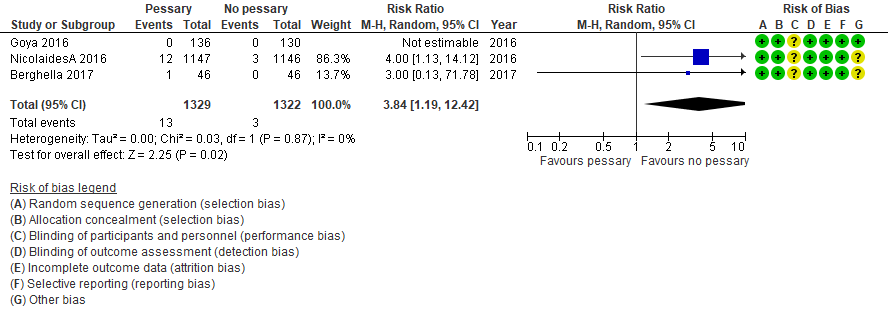
**

Conclusion: It is uncertain whether pessary increases the risk of ROP in neonates from a multifetal pregnancy, not considering additional maternal risk factor(s) for preterm birth (GRADE ⊕🌕🌕🌕).

**Admittance to neonatal intensive care unit (NICU) (**Appendix 4.3, STable 4.3.17 and SFigure 20)

A meta-analysis of two trials, including 2640 neonates showed no difference in the rate of NICU admission, RR 0.90 (95% CI 0.74 to 1.09). The crude event rate across trials was 14.7% without pessary. The pooled weighted RD was -1.5 % percentage points (95% CI -4.1 to 1.2).

**SFigure 20.** Outcome: Admittance to neonatal intensive care unit (NICU).

**
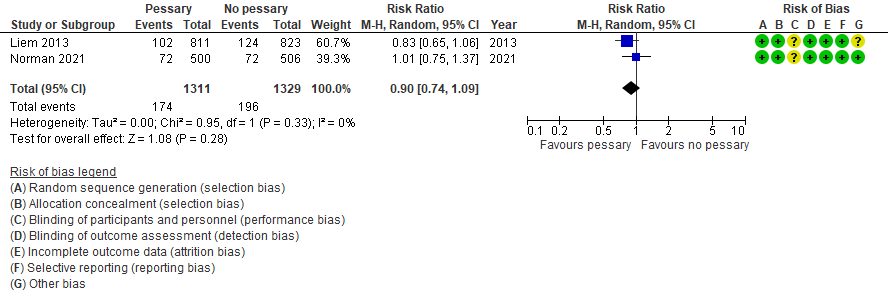
**

Conclusion: Pessary, compared with no pessary probably results in no difference in admittance to NICU in neonates from a multifetal pregnancy, not considering additional maternal risk factor(s) for preterm birth (GRADE ⊕⊕⊕🌕 ).

**Long-term child outcomes in multifetal** (Appendix 4.3, STable 4.3.18)

Two articles (714 children) examined long-term child outcome in twins and triplets (Van`t Hooft et al., 2018, Simons et al., 2019). Both articles are a follow up of the ProTWIN trial (Liem et al., 2013). Follow-up rate was 45% (Simons et al., 2019) and 83% (van 't Hooft et al., 2018). A meta-analysis was not feasible due to different outcomes. Van´t Hooft et al. showed no difference in neurodevelopment assessed by the Bayley-III Cognitive Composite score at 3 years, neither were there any differences in cognitive, language or motor development. Simons et al. (2019) concluded no improvement in development, behavioral, or physical outcomes of surviving children after four years.

**Mortality and morbidity in women with multifetal pregnancies**

**Maternal mortality <28 d (**Appendix 4.3, STable 4.3.19)

One trial from the Netherlands reported one maternal death in the intervention group (1/401).

The treatment was a cerclage instead of a pessary, and death occurred later due to chorioamnionitis.

**Hypertensive disorders in pregnancy (HDP) (**Appendix 4.3, STable 4.3.20)

One trial, including 808 women showed no difference in the rate of HDP, RR 1.24 (95% CI 0.89 to 1.74). The crude event rate across trials was 13.0% without pessary. The RD was 3.2 % percentage points (95% CI -1.7 to 8.1).

Conclusion: Pessary, compared with no pessary may result in no difference in HDP in women with a multifetal pregnancy, not considering additional risk factor(s) for preterm birth (GRADE ⊕⊕🌕 🌕 ).

**Gestational diabetes mellitus**

No trial reported gestational diabetes mellitus.

**Cholestasis of pregnancy**

No trial reported cholestasis of pregnancy.

**Chorioamnionitis** (Appendix 4.3, STable 4.3.21 and SFigure 21)

A meta-analysis of three trials, including 988 women showed no difference in the rate of chorioamnionitis, RR 1.05 (95% CI 0.53 to 2.06). The crude event rate across trials was 3.2% without pessary. The pooled weighted RD was 0.6 % percentage points (95% CI -3.1 to 4.2).

# SFigure 21. Outcome: Chorioamnionitis.

**
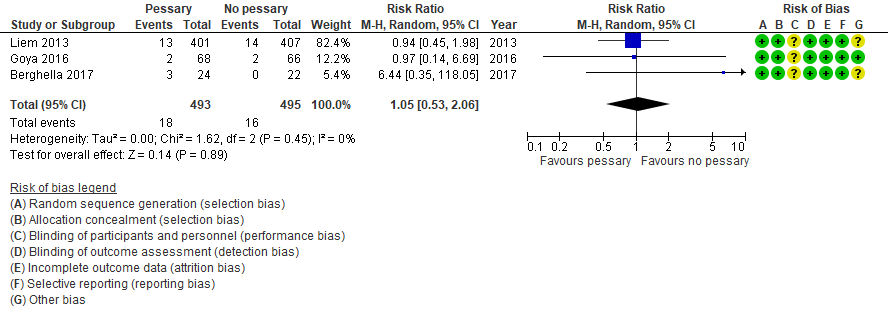
**

Conclusion: Pessary, compared with no pessary may result in no difference in chorioamnionitis in women with multifetal pregnancy, not considering additional risk factor(s) for preterm birth (GRADE ⊕⊕🌕 🌕).

**Genitourinary infections (**Appendix 4.3, STable 4.3.22 and SFigure 22)

A meta-analysis of two trials, including 854 women showed no difference in the rate of genitourinary infections, RR 4.24 (95% CI 0.74 to 24.39). The crude event rate across trials was 0.2% without pessary. The pooled weighted RD was 1.4 % percentage points (95% CI -2.5 to 5.4).

**SFigure 22.** Outcome: Genitourinary infection.

**
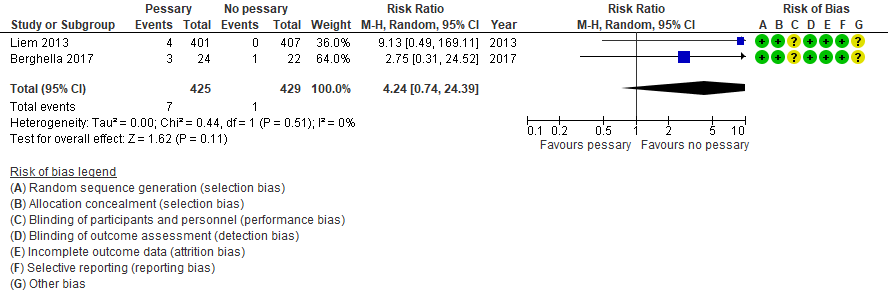
**

Conclusion: Its uncertain whether pessary, compared with no pessary results in any difference in genitourinary infections in women with multifetal pregnancy, not considering additional risk factor(s) for preterm birth (GRADE ⊕🌕🌕🌕).

**Vaginal discharge (**Appendix 4.3, STable 4.3.23 and SFigure 23)

A meta-analysis of two trials, including 180 women showed an increased rate of vaginal discharge, RR 1.88 (95% CI 1.53 to 2.31). The crude event rate across trials was 50.6% without pessary. The pooled weighted risk RD was 45.5 % percentage points
(95% CI 34.6 to 56.5).

**SFigure 23.** Outcome: Vaginal discharge.

**
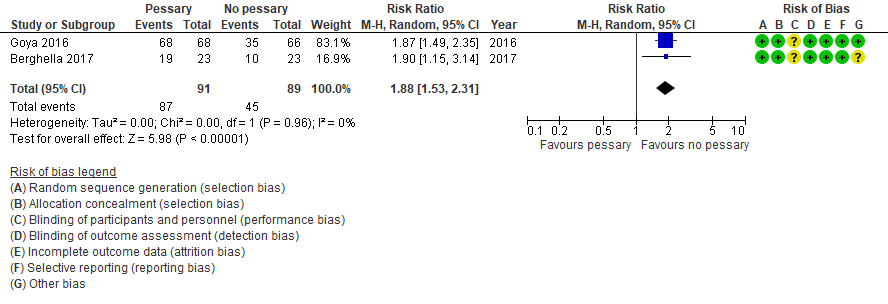
**

Conclusion: Pessary, compared with no pessary probably increases the risk of vaginal discharge in women with a twin pregnancy, not considering additional risk factor(s) for preterm birth (GRADE ⊕⊕⊕🌕 ).

**Preterm prelabor rupture of the membranes (PPROM)** **(**Appendix 4.3, STable 4.3.24 and SFigure 24)

A meta-analysis of four trials, including 1491 women showed no difference in the rate of PPROM, RR 0.99 (95% CI 0.44 to 2.21). The crude event rate across trials was 6.7% without pessary. The pooled weighted risk RD was -0.6 % percentage points (95% CI -5.3 to 4.2).

**SFigure 24.** Outcome: Preterm prelabor rupture of membranes.

**
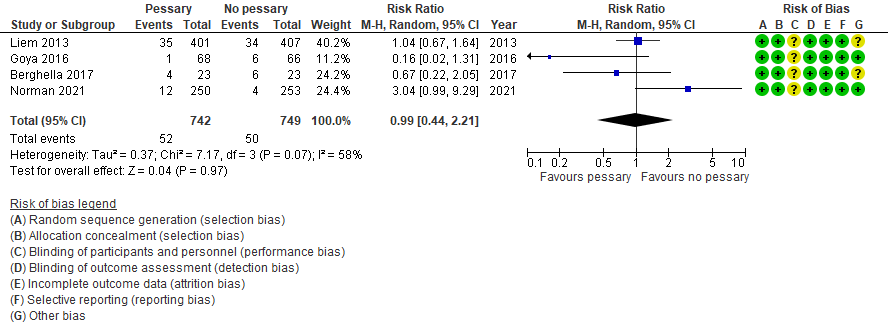
**

Conclusion: Pessary, compared with no pessary may result in no difference in PPROM in women with a multifetal pregnancy, not considering additional risk factor(s) for preterm birth
(GRADE ⊕⊕🌕 🌕).

# Subgroup analyses

Pre-specified subgroup analyses according to risk factors, of which only short cervical length was applicable, were conducted (SFigures 25 and 26)

.

# SFigure 25. Outcome: Any preterm birth <37 weeks according to the risk factor short cervical length.


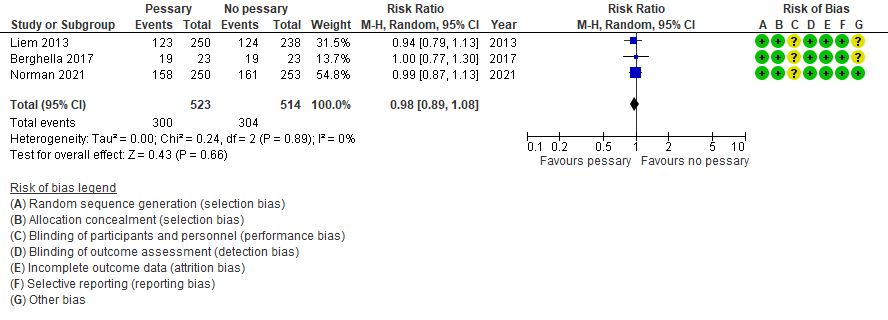


Cut-off cervical length: Liem 2013a ≤38 mm, Berghella 2017a <30 mm, Norman 2021 <35 mm.

**SFigure 26.** Outcome: Any preterm birth <34 weeks according to the risk factor short cervical length.


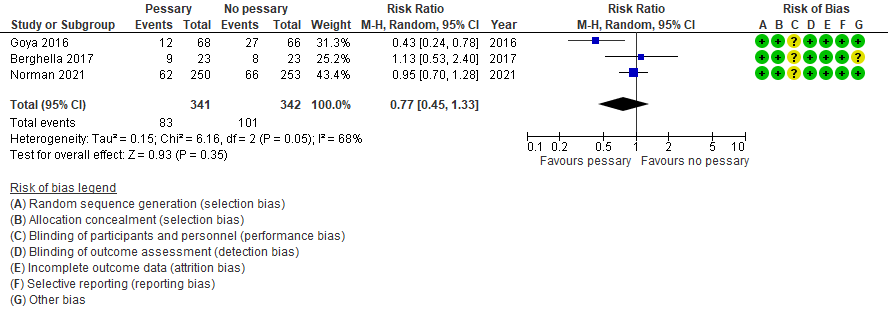


Cut-off cervical length: Goya 2016 ≤25 mm, Berghella 2017a <30 mm, Norman 2021 <35 mm.
